# Supplementary material for: Sulfated glycosaminoglycans inhibit LCMV entry and modulate antiviral immunity and pathology
Source: EMBO Mol Med. 2026 Feb 23;18(4):1235–64. doi: 10.1038/s44321-026-00387-8 (PMC13083911; doi:10.1038/s44321-026-00387-8)
Supplement: Supplementary file 1 — Appendix [file 44321_2026_387_MOESM1_ESM.pdf]

***Appendix***

***Sulfated glycosaminoglycans inhibit LCMV entry and modulate antiviral immunity and pathology***

Michal Gorzkiewicz[1,2†], Soha Noseir[1†], Mandar Vengurlekar[1], Mitrajit Ghosh[1], Ichiro Katahira[1], Džiuljeta Abromavičiūtė[1], Ulla Gerling-Driessen[3], Lorand Bonda[4], Nick Rähse[5], Marco Lapsien[5], Sabrina Bockholt[6], Ann Kathrin Bergmann[7], Konstantina Kostadinovska[1], Hafssa Fraii[1], Karl S. Lang[8], Lisa Oestereich[6,9], Holger Gohlke[5,10], Laura Hartmann[3], Philipp A. Lang[1*]

1. Department of Molecular Medicine II, Medical Faculty and University Hospital, Heinrich Heine University Düsseldorf, Universitätsstr. 1, 40225 Düsseldorf, Germany

2. Department of General Biophysics, Faculty of Biology and Environmental Protection, University of Lodz, 141/143 Pomorska St., 90-236 Lodz, Poland

3. Institute for Macromolecular Chemistry, University of Freiburg, Stefan-Meier-Str. 31, 79104 Freiburg, Germany

4. Department of Organic and Macromolecular Chemistry, Heinrich Heine University Düsseldorf, Universitätsstr. 1, 40225 Düsseldorf, Germany

5. Institute for Pharmaceutical and Medicinal Chemistry, Heinrich Heine University Düsseldorf, Universitätsstr. 1, 40225 Düsseldorf, Germany

6. Bernhard Nocht Institute for Tropical Medicine, Bernhard-Nocht-Str. 74, 20359 Hamburg, Germany

7. Electron Microscopy Core Facility, Medical Faculty and University Hospital, Heinrich Heine University, Universitätsstr. 1, 40225 Düsseldorf, Germany

8. Institute of Immunology, University Hospital Essen, Hufelandstr. 55, 45122 Essen, Germany

9. German Center for Infection Research, Partner Site Hamburg-Lübeck-Borstel-Riems

10. Institute of Bio- and Geosciences (IBG-4: Bioinformatics), Forschungszentrum Jülich GmbH, Wilhelm-Johnen-Straße, 52428 Jülich, Germany

† These authors contributed equally to this work

**Table of contents:**


- Appendix Supplementary Information
  - Dextran sulfation – elemental analysis and NMR spectrum……………………………….3
  - Synthesis of Man70 and Man70-sulf polymers……………………………………………4
  - Appendix Table S1: Outline of MD simulation steps performed…………………………..9
  - Appendix Table S2: Exact p values for all statistical analyses presented in the figures......10
- Appendix Figures

**Appendix Supplementary Information**

## 1. Dextran sulfation – elemental analysis and NMR spectrum

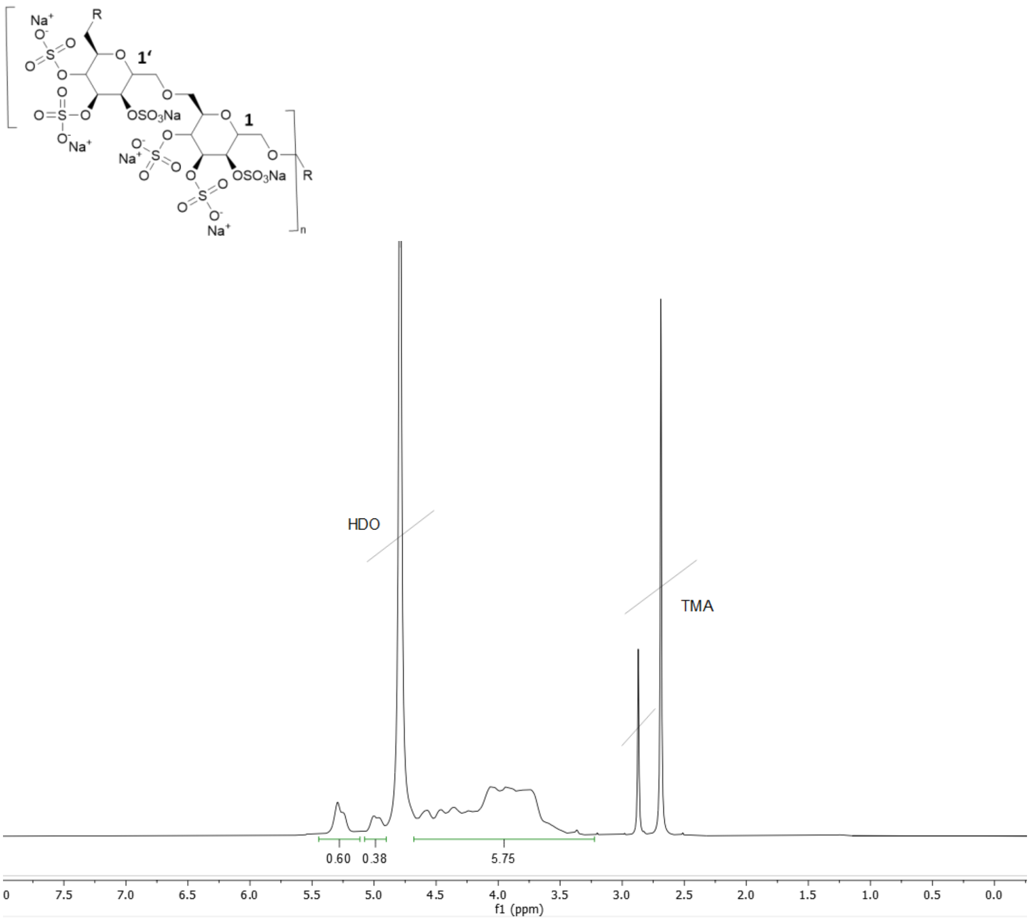


***$^{1}$H-NMR (400 MHz, $D_2O$)***

δ [ppm] 5.45-5.11 ppm (m, 0.6H,1); 5.07-4.90 ppm (m, 0.4H, 1‘); 5.67-3.22 ppm (m, 6H).

The ratios of carbon, hydrogen, nitrogen and sulfur were determined using a Vario Micro Cube provided by Analysensysteme GmbH. The measurements were carried out by the Institute for Pharmaceutical and Medicinal Chemistry, Heinrich Heine University Düsseldorf.

theoretical values (sulfated dextran): %C=18.41; %H=2.06; %S=19.66

measured values (sulfated dextran): %C=15.53; %H=2.74; %S=15.87

## 2. Synthesis of Man70 and Man70-sulf polymers

### UV-light source

Samples were irradiated with a UV-LED Spot P standard (405 nm) from Opsytec Dr. Gröbel GmbH.

### Irradiation intensities

Irradiation intensities were determined with a FieldMaxII-TO Laser Power Meter from Coherent.

### $^1$H-NMR

$^1$H-NMR spectra were recorded at RT with a Bruker AVANCE III 300 (for 300 MHz) and 600 (for 600 MHz). The chemical shifts were reported relative to solvent peaks (chloroform and water) as internal standards and reported as δ in parts per million (ppm). Multiplicities were abbreviated as s for singlet, d for doublet, t for triplet and m for multiplet.

### Size Exclusion Chromatography - Multi-angle Light Scattering ($H_2O$-SEC-MALS)

SEC analysis was conducted with an Agilent 1200 series HPLC system and three aqueous SEC columns provided by Polymer Standards Service (PSS). The columns included two Suprema Lux analytical columns (8 mm diameter and 5 μm particle size) and one precolumn (50 mm, 2 × 160 Å of 300 mm and 1000 Å of 300 mm). The eluent was a buffer system consisting of MilliQ water and 30% acetonitrile with 50 mM, $NaH_2PO_4$, 150 mM NaCl and 250 ppm $NaN_3$ with a pH=7.0 (via addition of 50 ml of 3 M aqueous sodium hydroxide solution) filtered with inline 0.1 μm membrane filter and running at 0.8 ml per minute. Multi-angle light scattering was recorded via mimDAWN TREOS and differential refractive index spectra with Optilab rEX, both supplied by Wyatt Technologies EU. Data analysis was performed with Astra 5 software and a dn/dc value of 0.156 for each polymer.

### Monomer synthesis

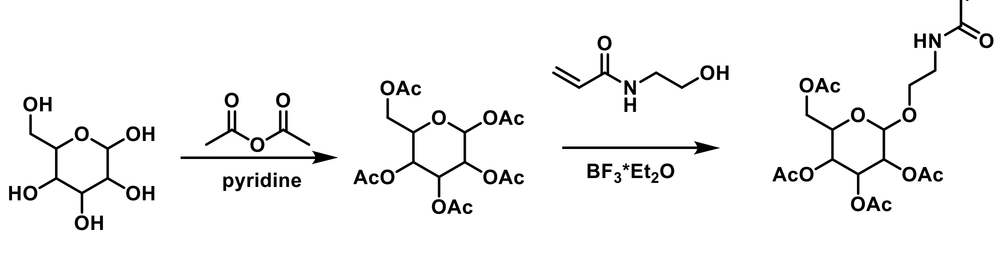


M2

The acetylated mannoseacrylamide monomer was synthesized by dissolving D-mannose in a 1:1 (*v*/*v*) mixture of pyridine/acetic anhydride (20 g/ml) and stirring at RT overnight. After diluting with ethylacetate, the mixture was extracted three times with 1 M HCl solution. Evaporation of ethylacetate resulted in 1,2,3,4,6-penta-O-acetyl-α-D-mannopyranose. Pentaacetylated mannose (1.0 eq.) and N-hydroxyethylacrylamide (1.2 eq.) were dissolved in DCM (2 mmol/ml) and flushed with argon gas for 10 minutes. $BF_3$*$Et_2O$ (10.0 eq.) was added through a syringe and the mixture was stirred at RT overnight. The reaction solution was washed three times with brine and the organic phase was dried with $MgSO_4$. The solvent was removed, which resulted in pure acetylated mannose monomer (AcO-ManAAm) with a relative purity of 98% and a yield of 78%.

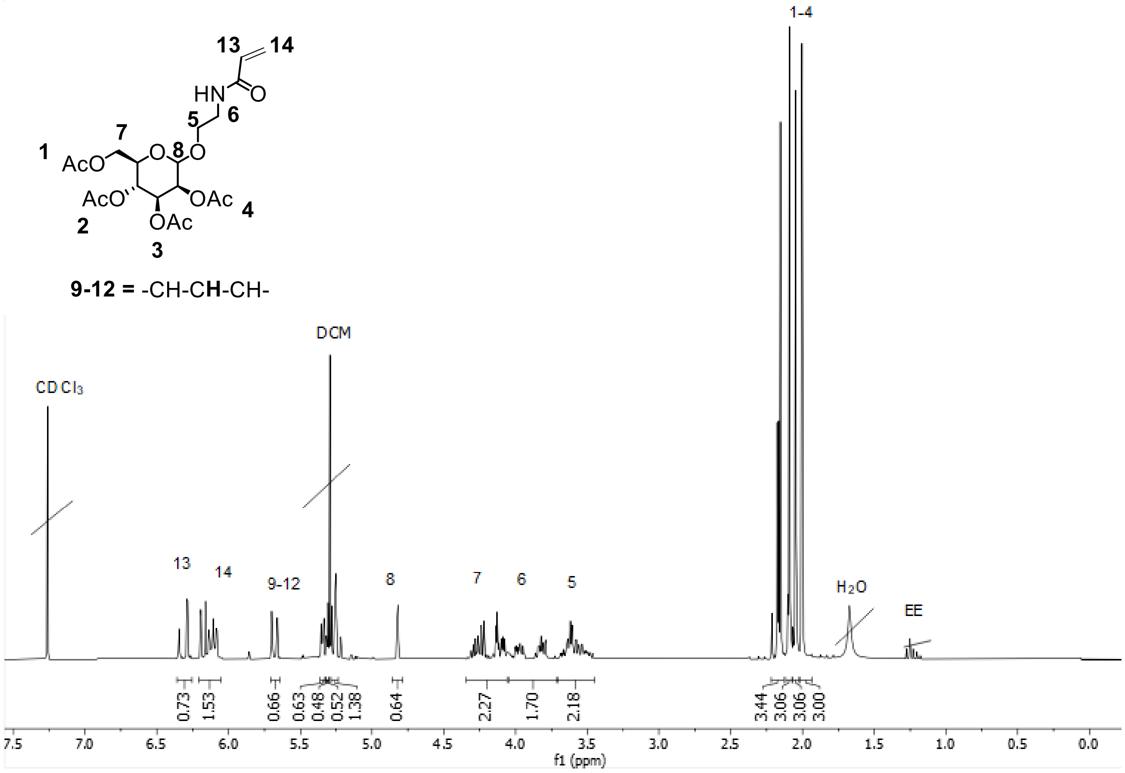


***$^1$H NMR (300 MHz, $CDCl_3$)***

**$^1$H-NMR** (300 MHz, $CDCl_3$): δ (ppm) 2.00-2.16 (s, 12H, C**H**$_3$ H1-4), 3.46-3.61 (m, 2H, C**H**$_2$ H5), 3.79-4.02 (m, 2H, C**H**$_2$, H6), 4.06-4.23 (m, 2H, C**H**$_2$, H7), 4.82 (s, 1H, C**H**, H8), 5.22-5.69 (m, 4H, C**H**, H9-12), 6.15 (dd, $^2$J=10.2 Hz, $^3$J=17.1 Hz, 2H, C**H**$_2$, H14), 6.32 (dd, $^2$J=1.2 Hz, $^3$J=17.1 Hz, 1H C**H**, H13)

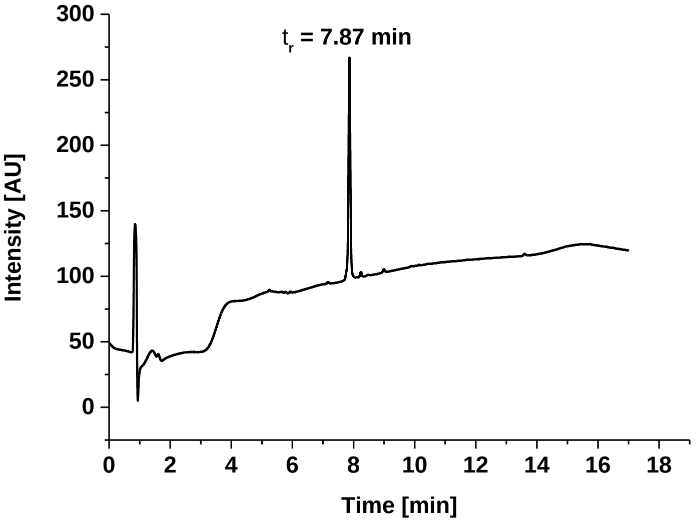


***RP-HPLC***

(A: 95% $H_2O$/ 5% MeCN/ 0.1% Formic Acid; 100% A → 50% A in 30 min)

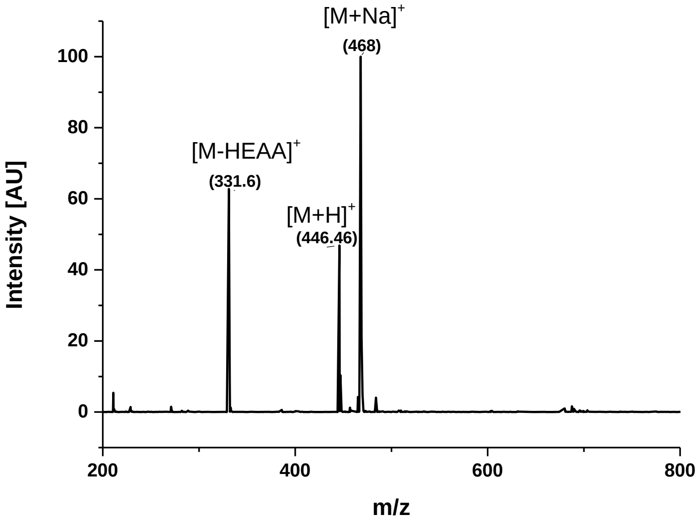


***ESI-MS***

m/z calculated for $C_{19}H_{27}NO_{11}$ $[M+H]^+$ 446.16 and $[M+Na]^+$ 468.15; found $[M+H]^+$ 446.46 and $[M+H]^+$ 468.

**Polymer synthesis**

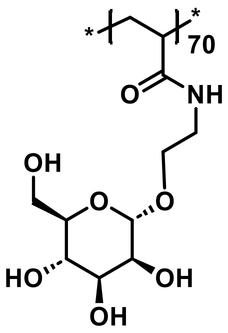


222.7 mg of monomer (0.5 mmol) and TPO (1.4 mol%, 0.007 mmol) were dissolved in DMF (10 wt.%) and the solution was flushed with argon for 10 min. and irradiated with UV-light (405 nm wavelength, with an intensity 45.2 mW/cm$^2$). After an hour, the irradiation was stopped and 5 ml NaOMe (0.2 M) in MeOH was added to the polymer solution and stirred for one hour at RT. Solid matter that has already precipitated and the residual solution was precipitated in diethyl ether. The precipitated polymer was dissolved in $H_2O$, dialyzed against distilled water (three cycles, 2 kDa) and subsequently lyophilized.

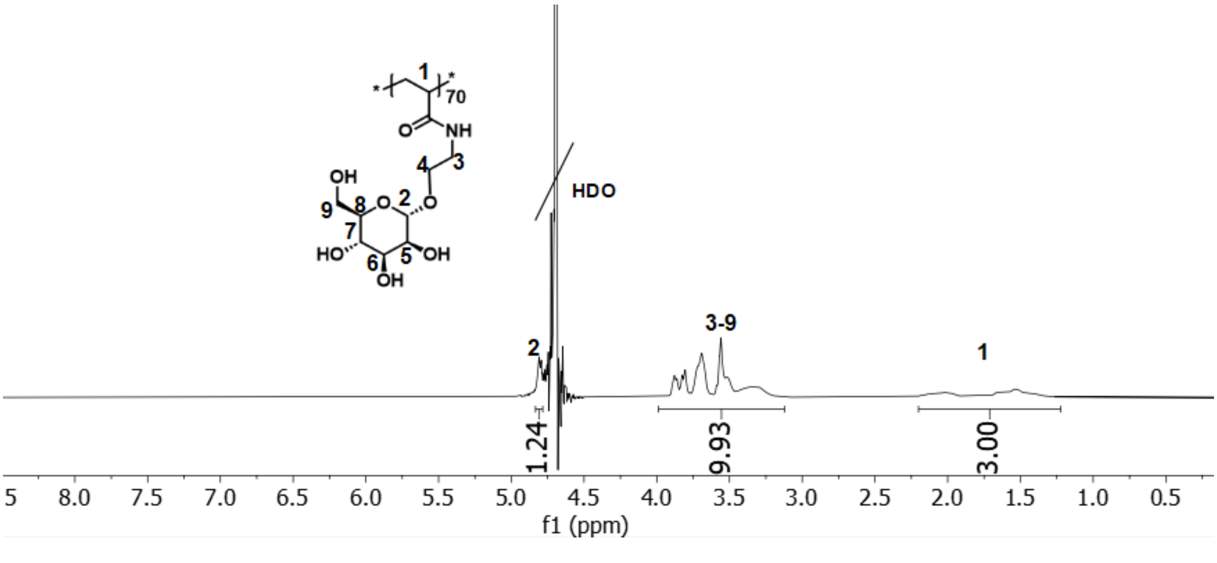


***$^1$H NMR spectrum (600 MHz, $D_2O$)***

$^1$H-NMR (600 MHz, $D_2O$) δ (ppm) 4.85-4.77 (m, **2,** $D_2O$ overlapping), 3.98-3.11 (m, **3-9**), 2.19-1.22 (m, **1**).

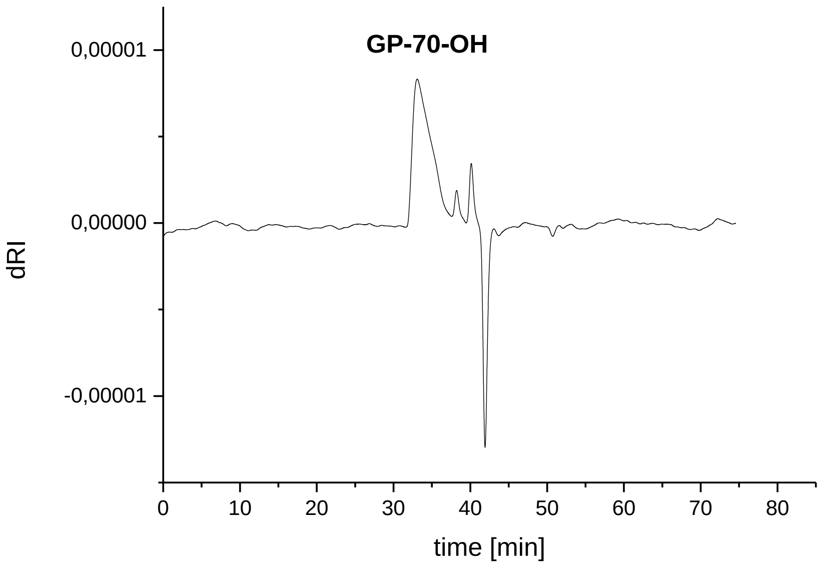


***$H_2O$-SEC spectrum***

Mn=19.4 kDa; Mw=25 kDa; Mw/Mn=1.29

Sulfation was performed according to the protocol in Soria-Martinez et al. 2020.

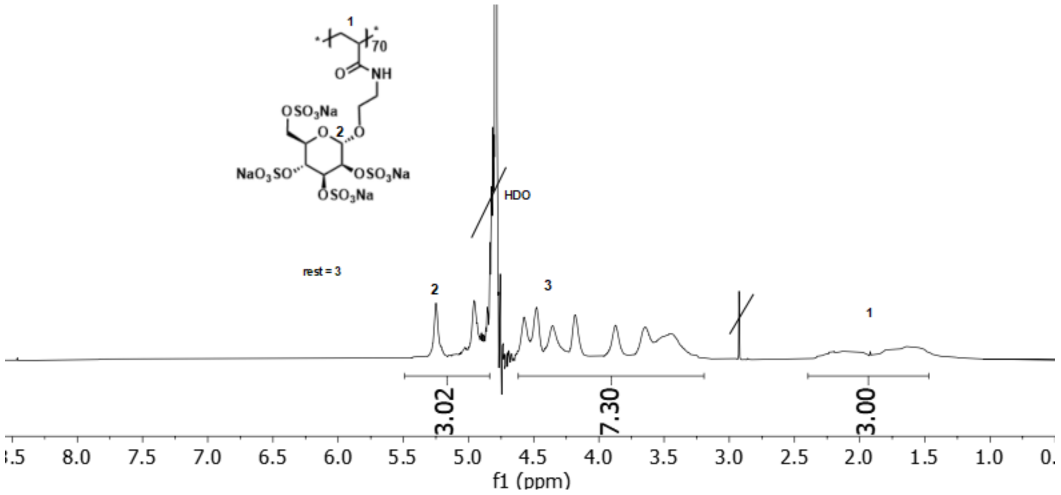


***$^1$H NMR spectrum (600 MHz, $D_2O$)***

$^1$H-NMR (600 MHz, $D_2O$) δ (ppm) 5.30-5.18 (m, **2**), 5.09-3.22 (m, **3**, $D_2O$ overlapping), 2.38-1.38 (m, **1**).

## Elemental analysis

The ratios of carbon, hydrogen, nitrogen and sulfur were determined using a Vario Micro Cube provided by Analysensysteme GmbH. The measurements were carried out by the Institute for Pharmaceutical and Medicinal Chemistry, Heinrich Heine University Düsseldorf.

theoretical values (n=70): %C=19.27; %H=2.21; %N=2.04; %S=18.71

measured values (n=70): %C=17.23; %H=3.34; %N=1.74; %S=15.51

*Appendix Table S1: Outline of MD simulation steps performed. Simulation length and restraints during minimization, thermalization, equilibration, and production steps of MD simulation of the LCMV-GP systems are showcased.*

| **Process** | **No. steps** | **Method** | **Restrained atoms and force constant (kcal mol$^{-1}$ Å$^{-2}$)** | | | |
|---|---|---|---|---|---|---|
| | | | *PBB*[a] | *PSC*[b] | *GSG*[c] | *DSG*[d] |
| Minimization | 2500/2500 | Steepest descent/Conjugate gradient | 10.0 | 5.0 | 10.0 | 2.5 |
| **Process** | **Simulation time [ps]** | **Ensemble/Time step [fs]** | **Restrained atoms and force constant (kcal mol$^{-1}$ Å$^{-2}$)** | | | |
| | | | *PBB*[a] | *PSC*[b] | *GSG*[c] | *DSG*[d] |
| Thermalization | 50.0 | NVT/1.0 | 10.0 | 5.0 | 10.0 | 2.5 |
| Equilibration 1 | 100.0 | NPT/2.0 | 5.0 | 2.5 | 5.0 | 2.5 |
| Equilibration 2 | 100.0 | NPT/2.0 | 2.5 | 1.0 | 2.5 | 2.5 |
| Equilibration 3 | 100.0 | NPT/4.0 | 1.0 | 0.5 | 1.0 | 2.5 |
| Equilibration 4 | 100.0 | NPT/4.0 | 0.5 | 0.1 | 0.5 | 2.5 |
| Equilibration 5 | 100.0 | NPT/4.0 | 0.1 | - | 0.1 | - |
| Equilibration 6 | 150.0 | NPT/4.0 | 0.05 | - | 0.05 | - |
| Equilibration 7 | 300.0 | NPT/4.0 | - | - | - | - |
| Production | $1.5 \cdot 10^6$ | NPT/4.0 | - | - | - | - |

[a]protein backbone atoms
[b]protein side-chain heavy atoms
[c]glycan atoms
[d]ligand (dextran) atoms

*Appendix Table S2: Exact p values for all statistical analyses presented in the figures*

| **Figure 1** | **p values** |
|---|---|
| **A** | |
| 0.05 | 0.9998 |
| 0.5 | 0.3085 |
| 5 | <0.0001 |
| 50 | <0.0001 |
| 500 | <0.0001 |
| 5000 | <0.0001 |
| **B** | |
| 0.05 | >0.9999 |
| 0.5 | 0.9995 |
| 5 | <0.0001 |
| 50 | <0.0001 |
| 500 | <0.0001 |
| 5000 | <0.0001 |
| **C** | |
| 0.05 | 0.9982 |
| 0.5 | 0.4424 |
| 5 | <0.0001 |
| 50 | <0.0001 |
| 500 | <0.0001 |
| 5000 | <0.0001 |
| **D** | |
| GP | 0.0668 |
| NP | 0.037 |
| Z | 0.0243 |
| **E** | |
| GP | 0.0061 |
| NP | <0.0001 |
| Z | 0.0078 |
| **G** | |
| 5 | 0.0003 |
| 50 | <0.0001 |
| 500 | <0.0001 |
| 5000 | <0.0001 |
| **H** | |
| 0.05 | 0.9948 |
| 0.5 | 0.0024 |
| 5 | <0.0001 |
| 50 | <0.0001 |
| 500 | <0.0001 |
| 5000 | <0.0001 |
| **I** | |
| 0.05 | 0.9949 |
| 0.5 | 0.0419 |
| 5 | <0.0001 |
| 50 | <0.0001 |
| 500 | <0.0001 |
| 5000 | <0.0001 |
| **J** | |
| 0.05 | 0.0092 |
| 0.5 | <0.0001 |
| 5 | <0.0001 |
| 50 | <0.0001 |
| 500 | <0.0001 |
| 5000 | <0.0001 |

| **Figure 2** | **p values** |
|---|---|
| **B** | |
| 5 | |
| vehicle vs. dextran | 0.9998 |
| vehicle vs. dextran sulfate 5 kDa | <0.0001 |
| vehicle vs. dextran sulfate 9-20 kDa | <0.0001 |
| vehicle vs. chondroitin sulfate A | 0.9973 |
| vehicle vs. chondroitin sulfate SC | 0.9997 |
| vehicle vs. ulvan | 0.9994 |
| vehicle vs. hyaluronic acid 15 kDa | 0.9997 |
| vehicle vs. hyaluronic acid 50 kDa | 0.9996 |
| 50 | |
| vehicle vs. dextran | 0.9997 |
| vehicle vs. dextran sulfate 5 kDa | <0.0001 |
| vehicle vs. dextran sulfate 9-20 kDa | <0.0001 |
| vehicle vs. chondroitin sulfate A | 0.9999 |
| vehicle vs. chondroitin sulfate SC | >0.9999 |
| vehicle vs. ulvan | 0.1435 |
| vehicle vs. hyaluronic acid 15 kDa | 0.9998 |
| vehicle vs. hyaluronic acid 50 kDa | 0.9996 |
| 500 | |
| vehicle vs. dextran | 0.9998 |
| vehicle vs. dextran sulfate 5 kDa | <0.0001 |
| vehicle vs. dextran sulfate 9-20 kDa | <0.0001 |
| vehicle vs. chondroitin sulfate A | 0.9997 |
| vehicle vs. chondroitin sulfate SC | 0.9997 |
| vehicle vs. ulvan | <0.0001 |
| vehicle vs. hyaluronic acid 15 kDa | 0.9997 |
| vehicle vs. hyaluronic acid 50 kDa | >0.9999 |
| 5000 | |
| vehicle vs. dextran | 0.0145 |
| vehicle vs. dextran sulfate 5 kDa | <0.0001 |
| vehicle vs. dextran sulfate 9-20 kDa | <0.0001 |
| vehicle vs. chondroitin sulfate A | <0.0001 |
| vehicle vs. chondroitin sulfate SC | <0.0001 |
| vehicle vs. ulvan | <0.0001 |
| vehicle vs. hyaluronic acid 15 kDa | 0.9999 |
| vehicle vs. hyaluronic acid 50 kDa | 0.0051 |
| **E** | |
| 0.05 | 0.8119 |
| 0.5 | 0.0763 |
| 5 | <0.0001 |
| 50 | <0.0001 |
| 500 | <0.0001 |
| 5000 | <0.0001 |
| **F** | |
| 0.05 | 0.9082 |
| 0.5 | 0.0616 |
| 5 | <0.0001 |
| 50 | <0.0001 |
| 500 | <0.0001 |
| 5000 | <0.0001 |
| **H** | |
| 0.005 | 0.9996 |
| 0.05 | 0.9999 |
| 0.5 | 0.5332 |
| 5 | 0.013 |
| 50 | <0.0001 |
| 500 | <0.0001 |
| **I** | |
| **LASV** | |
| 0.05 | |

| **Figure 3** | **p values** |
|---|---|
| **C** | |
| D 1 min. vs. D 5 min. | 0.7742 |
| D 1 min. vs. D 15 min. | 0.0025 |
| DS 1 min. vs. DS 5 min. | 0.9534 |
| DS 1 min. vs. DS 15 min. | 0.813 |
| **D** | |
| **NP** | |
| 5 min. | |
| vehicle vs. dextran | 0.9975 |
| vehicle vs. dextran sulfate | 0.9342 |
| 15 min. | |
| vehicle vs. dextran | 0.9522 |
| vehicle vs. dextran sulfate | 0.8677 |
| 45 min. | |
| vehicle vs. dextran | 0.849 |
| vehicle vs. dextran sulfate | 0.4643 |
| 150 min. | |
| vehicle vs. dextran | 0.7653 |
| vehicle vs. dextran sulfate | 0.0002 |
| **Z** | |
| 5 min. | |
| vehicle vs. dextran | 0.9907 |
| vehicle vs. dextran sulfate | 0.936 |
| 15 min. | |
| vehicle vs. dextran | 0.9709 |
| vehicle vs. dextran sulfate | 0.9551 |
| 45 min. | |
| vehicle vs. dextran | 0.9416 |
| vehicle vs. dextran sulfate | 0.5676 |
| 150 min. | |
| vehicle vs. dextran | 0.4544 |
| vehicle vs. dextran sulfate | 0.0002 |
| **E** | |
| vehicle vs. 5 µg/ml | 0.0002 |
| vehicle vs. 50 µg/ml | <0.0001 |
| vehicle vs. 500 µg/ml | <0.0001 |
| vehicle filtered vs. 5 µg/ml filtered | 0.0002 |
| vehicle filtered vs. 50 µg/ml filtered | <0.0001 |
| vehicle filtered vs. 500 µg/ml filtered | <0.0001 |
| **G** | |
| medium control vs. D 5 µg/ml | >0.9999 |
| medium control vs. D 50 µg/ml | 0.7194 |
| medium control vs. D 500 µg/ml | 0.9984 |
| medium control vs. DS 5 µg/ml | 0.0004 |
| medium control vs. DS 50 µg/ml | 0.0002 |
| medium control vs. DS 500 µg/ml | <0.0001 |

| | |
|---|---|
| Dextran vs. Heparin | 0.3034 |
| Dextran vs. Dextran sulfate | 0.0341 |
| 0.5 | |
| Dextran vs. Heparin | 0.1975 |
| Dextran vs. Dextran sulfate | 0.9789 |
| 5 | |
| Dextran vs. Heparin | 0.9789 |
| Dextran vs. Dextran sulfate | 0.1693 |
| 50 | |
| Dextran vs. Heparin | <0.0001 |
| Dextran vs. Dextran sulfate | <0.0001 |
| 500 | |
| Dextran vs. Heparin | <0.0001 |
| Dextran vs. Dextran sulfate | <0.0001 |
| 5000 | |
| Dextran vs. Heparin | <0.0001 |
| Dextran vs. Dextran sulfate | <0.0001 |
| **LUJV** | |
| 0.05 | |
| Dextran vs. Heparin | 0.331 |
| Dextran vs. Dextran sulfate | 0.9699 |
| 0.5 | |
| Dextran vs. Heparin | <0.0001 |
| Dextran vs. Dextran sulfate | <0.0001 |
| 5 | |
| Dextran vs. Heparin | <0.0001 |
| Dextran vs. Dextran sulfate | <0.0001 |
| 50 | |
| Dextran vs. Heparin | <0.0001 |
| Dextran vs. Dextran sulfate | <0.0001 |
| 500 | |
| Dextran vs. Heparin | <0.0001 |
| Dextran vs. Dextran sulfate | <0.0001 |
| 5000 | |
| Dextran vs. Heparin | 0.1223 |
| Dextran vs. Dextran sulfate | 0.1223 |
| **JUNV** | |
| 0.05 | |
| Dextran vs. Heparin | 0.0012 |
| Dextran vs. Dextran sulfate | 0.0162 |
| 0.5 | |
| Dextran vs. Heparin | <0.0001 |
| Dextran vs. Dextran sulfate | <0.0001 |
| 5 | |
| Dextran vs. Heparin | <0.0001 |
| Dextran vs. Dextran sulfate | <0.0001 |
| 50 | |
| Dextran vs. Heparin | <0.0001 |
| Dextran vs. Dextran sulfate | <0.0001 |
| 500 | |
| Dextran vs. Heparin | <0.0001 |
| Dextran vs. Dextran sulfate | <0.0001 |
| 5000 | |
| Dextran vs. Heparin | <0.0001 |
| Dextran vs. Dextran sulfate | <0.0001 |
| **MACV** | |
| 0.05 | |
| Dextran vs. Heparin | <0.0001 |
| Dextran vs. Dextran sulfate | 0.0021 |
| 0.5 | |
| Dextran vs. Heparin | <0.0001 |
| Dextran vs. Dextran sulfate | <0.0001 |
| 5 | |
| Dextran vs. Heparin | <0.0001 |

| | |
|---|---|
| Dextran vs. Dextran sulfate | <0.0001 |
| 50 | |
| Dextran vs. Heparin | <0.0001 |
| Dextran vs. Dextran sulfate | <0.0001 |
| 500 | |
| Dextran vs. Heparin | <0.0001 |
| Dextran vs. Dextran sulfate | <0.0001 |
| 5000 | |
| Dextran vs. Heparin | 0.0004 |
| Dextran vs. Dextran sulfate | 0.0003 |

| **Figure 4** | **p values** |
|---|---|
| **A** | |
| **rWT** | |
| 0.05 | 0.9998 |
| 0.5 | 0.1269 |
| 5 | <0.0001 |
| 50 | <0.0001 |
| 500 | <0.0001 |
| 5000 | <0.0001 |
| **H155Y** | |
| 0.05 | 0.9915 |
| 0.5 | 0.3394 |
| 5 | <0.0001 |
| 50 | <0.0001 |
| 500 | <0.0001 |
| 5000 | <0.0001 |
| **B** | |
| **rWT** | |
| 0.05 | 0.995 |
| 0.5 | 0.6268 |
| 5 | 0.0683 |
| 50 | <0.0001 |
| 500 | <0.0001 |
| 5000 | <0.0001 |
| **H155Y** | |
| 0.05 | 0.9996 |
| 0.5 | 0.6401 |
| 5 | 0.0281 |
| 50 | <0.0001 |
| 500 | <0.0001 |
| 5000 | <0.0001 |

| **Figure 5** | **p values** |
|---|---|
| **B** | |
| Spleen | <0.0001 |
| Liver | <0.0001 |
| **D** | <0.0001 |
| **E** | <0.0001 |
| **G** | <0.0001 |
| **H** | |
| **Spleen** | |
| usp18 | 0.000431 |
| ifit3 | 0.002888 |
| ifi47 | 0.002005 |
| mx1 | 0.0621 |
| socs1 | 0.659358 |
| lcmv gp | 0.000034 |
| lcmv np | 0.006962 |
| lcmv z | 0.00778 |
| **Liver** | |
| usp18 | <0.000001 |
| ifit3 | 0.000561 |
| ifi47 | 0.000213 |
| mx1 | <0.000001 |
| socs1 | 0.017255 |
| lcmv gp | 0.000001 |
| lcmv np | 0.000039 |
| lcmv z | 0.000017 |

| **Figure 6** | **p values** |
|---|---|
| **A** | |
| **rWT** | |
| 0.05 | 0.6914 |
| 0.5 | 0.0238 |
| 5 | <0.0001 |
| 50 | <0.0001 |
| 500 | <0.0001 |
| 5000 | <0.0001 |
| **H155Y** | |
| 0.05 | >0.9999 |
| 0.5 | 0.8024 |
| 5 | 0.978 |
| 50 | 0.0003 |
| 500 | <0.0001 |
| 5000 | <0.0001 |
| **D** | |
| LCMV vs. control | <0.0001 |
| LCMV vs. 5000 D | 0.6133 |
| LCMV vs. 500 D | 0.9131 |
| LCMV vs. 50 D | 0.2996 |
| LCMV vs. 5 D | 0.2784 |
| LCMV vs. 0.5 D | 0.2132 |
| LCMV vs. 0.05 D | 0.0598 |
| LCMV vs. 5000 DS. | <0.0001 |
| LCMV vs. 500 DS. | <0.0001 |
| LCMV vs. 50 DS. | <0.0001 |
| LCMV vs. 5 DS. | <0.0001 |
| LCMV vs. 0.5 DS. | 0.0002 |
| LCMV vs. 0.05 DS. | 0.0869 |
| **F** | |
| **Blood** | |
| gp33 | 0.001743 |
| np396 | 0.000151 |
| **Spleen** | |
| gp33 | 0.000522 |
| np396 | 0.000624 |
| **Liver** | |
| gp33 | 0.052095 |
| np396 | 0.000178 |
| **G** | |
| **Blood** | |
| **SLEC** | |
| gp33 | 0.000081 |
| np396 | 0.034561 |
| **MPEC** | |
| gp33 | 0.045839 |
| np396 | 0.226609 |
| **Spleen** | |
| **SLEC** | |
| gp33 | 0.000157 |
| np396 | 0.030693 |
| **MPEC** | |

| | |
|---|---|
| gp33 | 0.007076 |
| np396 | 0.226515 |
| **Liver** | |
| **SLEC** | |
| gp33 | 0.019861 |
| np396 | 0.059209 |
| **MPEC** | |
| gp33 | 0.039338 |
| np396 | 0.003445 |
| **H** | |
| **Blood** | |
| u.s. | 0.9715 |
| gp33 | <0.0001 |
| np396 | 0.2128 |
| **Spleen** | |
| u.s. | 0.6611 |
| gp33 | <0.0001 |
| np396 | 0.0964 |
| **Liver** | |
| u.s. | 0.0159 |
| gp33 | 0.0008 |
| np396 | 0.0208 |
| **I** | |
| AST | <0.0001 |
| ALT | 0.0264 |
| **J** | |
| Blood | 0.0002 |
| Spleen | 0.0002 |
| Liver | 0.0448 |
| Kidney | <0.0001 |
| Lung | <0.0001 |
| Brain | <0.0001 |

| **Figure 7** | **p values** |
|---|---|
| **B** | |
| **Blood** | |
| gp33 | 0.010221 |
| np396 | 0.007607 |
| **C** | |
| **Blood** | |
| **SLEC** | |
| gp33 | 0.554172 |
| np396 | 0.020695 |
| **MPEC** | |
| gp33 | 0.04359 |
| np396 | 0.123576 |
| **D** | |
| **IFNγ** | |
| u.s. | >0.9999 |
| gp33 | <0.0001 |
| np396 | 0.8408 |
| **TNFα** | |
| u.s. | 0.6656 |
| gp33 | 0.0011 |
| np396 | 0.0211 |
| **E** | |
| **Spleen** | 0.0265 |
| **Liver** | <0.0001 |
| **F** | |
| **Spleen** | |
| GP | 0.02117 |
| NP | 0.043988 |
| Z | 0.066773 |
| **Liver** | |

| **Figure EV1** | **p values** |
|---|---|
| **B** | |
| GP | 0.0079 |
| NP | 0.0253 |
| Z | 0.0431 |
| **C** | |
| GP | 0.032 |
| NP | 0.0018 |
| Z | 0.0158 |
| **D** | |
| GP | 0.0024 |
| NP | 0.0004 |
| Z | 0.0028 |
| **E** | |
| GP | 0.043 |
| NP | 0.0107 |
| Z | 0.0386 |
| **F** | |
| 0.005 | 0.053 |
| 0.05 | 0.2544 |
| 0.5 | 0.9998 |
| 5 | 0.0021 |
| 50 | <0.0001 |
| 500 | <0.0001 |
| **G** | |
| **TCRV** | |
| 0.05 | |
| Dextran vs. Heparin | <0.0001 |
| Dextran vs. Dextran sulfate | <0.0001 |
| 0.5 | |
| Dextran vs. Heparin | 0.0496 |

| **Figure EV2** | **p values** |
|---|---|
| **B** | |
| LCMV vs. control | <0.0001 |
| LCMV vs. 5000 D | 0.9993 |
| LCMV vs. 500 D | 0.9991 |
| LCMV vs. 50 D | 0.9993 |
| LCMV vs. 5 D | 0.7647 |
| LCMV vs. 0.5 D | 0.988 |
| LCMV vs. 0.05 D | 0.9864 |
| LCMV vs. 5000 DS. | <0.0001 |
| LCMV vs. 500 DS. | <0.0001 |
| LCMV vs. 50 DS. | <0.0001 |
| LCMV vs. 5 DS. | >0.9999 |
| LCMV vs. 0.5 DS. | 0.9999 |
| LCMV vs. 0.05 DS. | 0.8926 |
| **C** | |
| LCMV vs. control | <0.0001 |
| LCMV vs. 5000 D | 0.8129 |
| LCMV vs. 500 D | 0.9117 |
| LCMV vs. 50 D | 0.5298 |
| LCMV vs. 5 D | 0.6084 |
| LCMV vs. 0.5 D | 0.4202 |
| LCMV vs. 0.05 D | 0.1045 |
| LCMV vs. 5000 DS. | <0.0001 |
| LCMV vs. 500 DS. | <0.0001 |
| LCMV vs. 50 DS. | <0.0001 |
| LCMV vs. 5 DS. | <0.0001 |
| LCMV vs. 0.5 DS. | 0.0005 |
| LCMV vs. 0.05 DS. | 0.1086 |
| **E** | |
| **CD44+/CD62L- cells (% of CD8+)** | |

| | |
|---|---|
| GP | 0.044757 |
| NP | 0.070202 |
| Z | 0.027656 |
| **H** | <0.0001 |
| **J** | <0.0001 |
| **K** | <0.0001 |
| **L** | |
| AST | <0.0001 |
| ALT | 0.0013 |

| | |
|---|---|
| Dextran vs. Dextran sulfate | 0.0012 |
| 5 | |
| Dextran vs. Heparin | 0.0179 |
| Dextran vs. Dextran sulfate | 0.0004 |
| 50 | |
| Dextran vs. Heparin | 0.0012 |
| Dextran vs. Dextran sulfate | 0.0008 |
| 500 | |
| Dextran vs. Heparin | 0.0125 |
| Dextran vs. Dextran sulfate | <0.0001 |
| 5000 | |
| Dextran vs. Heparin | 0.4579 |
| Dextran vs. Dextran sulfate | 0.0488 |

| | |
|---|---|
| control vs. D 50 µg/ml | 0.9972 |
| control vs. D 5 µg/ml | 0.4522 |
| control vs. D 0.5 µg/ml | 0.257 |
| control vs. D 0.05 µg/ml | 0.5232 |
| control vs. DS 50 µg/ml | 0.005 |
| control vs. DS 5 µg/ml | 0.0067 |
| control vs. DS 0.5 µg/ml | 0.9997 |
| control vs. DS 0.05 µg/ml | 0.946 |
| **Proliferation** | |
| control vs. D 50 µg/ml | 0.9995 |
| control vs. D 5 µg/ml | 0.9742 |
| control vs. D 0.5 µg/ml | 0.7619 |
| control vs. D 0.05 µg/ml | 0.9062 |
| control vs. DS 50 µg/ml | 0.0402 |
| control vs. DS 5 µg/ml | 0.174 |
| control vs. DS 0.5 µg/ml | 0.9995 |
| control vs. DS 0.05 µg/ml | 0.9978 |

| **Figure EV3** | **p values** |
|---|---|
| **A** | |
| **Blood** | |
| gp33 | 0.010306 |
| np396 | 0.029381 |
| **B** | |
| **Blood** | |
| **SLEC** | |
| gp33 | 0.007987 |
| np396 | 0.035223 |
| **MPEC** | |
| gp33 | 0.003479 |
| np396 | 0.024352 |
| **C** | |
| **TIM3** | |
| gp33 | 0.093419 |
| np396 | 0.024428 |
| **PD1** | |
| gp33 | 0.018826 |
| np396 | 0.017549 |
| **D** | |
| **TIM3** | |
| gp33 | 0.077927 |
| np396 | 0.86458 |
| **PD1** | |
| gp33 | 0.01259 |
| np396 | 0.985352 |
| **2B4** | |
| gp33 | 0.028664 |
| np396 | 0.903941 |
| **LAG3** | |
| gp33 | 0.068885 |
| np396 | 0.6622 |
| **E** | |
| **TIM3** | |
| gp33 | 0.045702 |
| np396 | 0.101209 |
| **PD1** | |
| gp33 | 0.004841 |
| np396 | 0.01011 |
| **2B4** | |
| gp33 | 0.000106 |
| np396 | 0.873268 |
| **LAG3** | |
| gp33 | 0.001238 |
| np396 | 0.531151 |

| **Figure EV4** | **p values** |
|---|---|
| **C** | |
| **IFNγ** | |
| **Liver** | |
| u.s. | 0.4092 |
| gp33 | 0.0013 |
| np396 | 0.2457 |
| **D** | |
| **2B4** | |
| gp33 | 0.015353 |
| np396 | 0.15563 |
| **LAG3** | |
| gp33 | 0.043854 |
| np396 | 0.185319 |
| **F** | |
| **TIM3** | |
| gp33 | 0.451681 |
| np396 | 0.000214 |
| **PD1** | |
| gp33 | 0.699569 |
| np396 | 0.000726 |

| **Figure EV5** | **p values** |
|---|---|
| **E** | |
| **SLEC** | |
| gp33 | 0.087398 |
| np396 | 0.028301 |
| **I** | |
| **PD1** | |
| gp33 | 0.033453 |
| np396 | 0.031212 |
| **J** | |
| **IFNγ** | |
| u.s. | >0.9999 |
| gp33 | 0.0187 |
| np396 | 0.9928 |
| **TNFα** | |
| u.s. | 0.9999 |
| gp33 | 0.0189 |
| np396 | 0.9996 |
| **K** | |
| **IFNγ** | |
| u.s. | >0.9999 |
| gp33 | 0.0127 |
| np396 | 0.2164 |
| **TNFα** | |
| u.s. | >0.9999 |
| gp33 | 0.0083 |
| np396 | 0.9934 |
| **M** | 0.006 |

| | |
|---|---|
| **F** | |
| **TIM3** | |
| gp33 | 0.008564 |
| np396 | 0.175917 |
| **PD1** | |
| gp33 | 0.000185 |
| np396 | 0.2116 |
| **2B4** | |
| gp33 | 0.000104 |
| np396 | 0.539531 |
| **LAG3** | |
| gp33 | 0.000123 |
| np396 | 0.749617 |
| **G** | |
| **IFNγ** | |
| u.s. | 0.9948 |
| gp33 | 0.0009 |
| np396 | 0.9352 |
| **TNFα** | |
| u.s. | 0.8726 |
| gp33 | 0.006 |
| np396 | 0.9744 |
| **H** | |
| AST | 0.0004 |
| ALT | 0.0045 |
| **I** | <0.0001 |
| **J** | |
| **GP** | |
| Spleen | 0.02553 |
| Liver | 0.151962 |
| Kidney | 0.016243 |
| Lung | 0.048768 |
| Brain | 0.032155 |
| **NP** | |
| Spleen | 0.05941 |
| Liver | 0.109799 |
| Kidney | 0.014495 |
| Lung | 0.038128 |
| Brain | 0.038958 |
| **Z** | |
| Spleen | 0.042736 |
| Liver | 0.061301 |
| Kidney | 0.041851 |
| Lung | 0.080331 |
| Brain | 0.041486 |

| **Figure S1** | **p values** |
|---|---|
| **A** | |
| GP | 0.0863 |
| NP | 0.0216 |
| Z | 0.0043 |
| **B** | |
| GP | 0.0331 |
| NP | 0.0051 |
| Z | 0.0149 |

| **Figure S3** | **p values** |
|---|---|
| **A** | |
| **rWT** | |
| 0.05 | 0.8262 |
| 0.5 | 0.8669 |
| 5 | <0.0001 |
| 50 | <0.0001 |
| 500 | <0.0001 |

| **Figure S6** | **p values** |
|---|---|
| | |
| **B** | <0.0001 |
| **C** | |
| AST | 0.0084 |
| ALT | 0.0388 |

| **Figure S8** | **p values** |
|---|---|
| **A** | |
| **CD62L** | |
| gp33 | 0.207392 |
| np396 | 0.09015 |
| **KLRG1** | |
| gp33 | 0.159939 |
| np396 | 0.018433 |
| **CD62L %** | |
| gp33 | 0.357445 |
| np396 | 0.032347 |

| **Figure S9** | **p values** |
|---|---|
| **A** | |
| **CD44** | |
| gp33 | 0.216247 |
| np396 | 0.002275 |
| **B** | |
| **CD44** | |
| gp33 | 0.011274 |
| np396 | 0.14507 |
| **CD44 %** | |
| gp33 | 0.016145 |
| np396 | 0.212965 |
| **C** | |
| **IL7R** | |
| gp33 | 0.554344 |
| np396 | 0.003872 |

| | |
|---|---|
| 5000 | <0.0001 |
| **H155Y** | |
| 0.05 | 0.9997 |
| 0.5 | <0.0001 |
| 5 | <0.0001 |
| 50 | <0.0001 |
| 500 | <0.0001 |
| 5000 | <0.0001 |
| **B** | |
| **rWT** | |
| 0.05 | >0.9999 |
| 0.5 | 0.9998 |
| 5 | 0.9727 |
| 50 | 0.0006 |
| 500 | <0.0001 |
| 5000 | <0.0001 |
| **H155Y** | |
| 0.05 | 0.9792 |
| 0.5 | 0.9715 |
| 5 | 0.8537 |
| 50 | 0.0224 |
| 500 | <0.0001 |
| 5000 | <0.0001 |
| **C** | |
| **rWT** | |
| 0.05 | 0.9568 |
| 0.5 | 0.7827 |
| 5 | <0.0001 |
| 50 | <0.0001 |
| 500 | <0.0001 |
| 5000 | <0.0001 |
| **H155Y** | |
| 0.05 | 0.5271 |
| 0.5 | 0.001 |
| 5 | <0.0001 |
| 50 | <0.0001 |
| 500 | <0.0001 |
| 5000 | <0.0001 |
| **D** | |
| **rWT** | |
| 0.05 | 0.9964 |
| 0.5 | 0.9999 |
| 5 | 0.5986 |
| 50 | <0.0001 |
| 500 | <0.0001 |
| 5000 | <0.0001 |
| **H155Y** | |
| 0.05 | 0.8406 |
| 0.5 | 0.1453 |
| 5 | 0.0314 |
| 50 | <0.0001 |
| 500 | <0.0001 |
| 5000 | <0.0001 |
| **E** | |
| 0.05 | 0.9997 |
| 0.5 | <0.0001 |
| 5 | <0.0001 |
| 50 | <0.0001 |
| 500 | <0.0001 |
| 5000 | <0.0001 |
| **F** | |
| 0.05 | 0.9979 |
| 0.5 | 0.7305 |
| 5 | 0.3195 |

| | |
|---|---|
| **B** | |
| **CD62L** | |
| gp33 | 0.368999 |
| np396 | 0.018025 |
| **CD44** | |
| gp33 | 0.000668 |
| np396 | 0.005641 |
| **KLRG1** | |
| gp33 | 0.000053 |
| np396 | 0.556574 |
| **IL7R** | |
| gp33 | 0.041374 |
| np396 | 0.006485 |
| **CD44 %** | |
| gp33 | 0.000209 |
| np396 | 0.00054 |
| **KLRG1 %** | |
| gp33 | 0.001004 |
| np396 | 0.630468 |
| **IL7R %** | |
| gp33 | 0.369797 |
| np396 | 0.040908 |
| **C** | |
| **CD62L** | |
| gp33 | 0.00063 |
| np396 | 0.001069 |
| **CD44** | |
| gp33 | 0.000109 |
| np396 | 0.184084 |
| **KLRG1** | |
| gp33 | 0.288934 |
| np396 | 0.014379 |
| **IL7R** | |
| gp33 | 0.000093 |
| np396 | 0.00007 |
| **CD62L %** | |
| gp33 | 0.000292 |
| np396 | 0.000024 |
| **CD44 %** | |
| gp33 | 0.000037 |
| np396 | 0.893235 |
| **KLRG1 %** | |
| gp33 | 0.047221 |
| np396 | 0.000421 |
| **IL7R %** | |
| gp33 | 0.024891 |
| np396 | 0.000001 |
| **D** | |
| **CD62L** | |
| gp33 | 0.924225 |
| np396 | 0.007051 |
| **CD44** | |
| gp33 | 0.000298 |
| np396 | 0.001153 |
| **KLRG1** | |
| gp33 | 0.009592 |
| np396 | 0.000024 |
| **IL7R** | |
| gp33 | 0.578138 |
| np396 | 0.087422 |
| **CD62L %** | |
| gp33 | 0.192494 |
| np396 | 0.014914 |
| **CD44 %** | |

| | |
|---|---|
| 50 | <0.0001 |
| 500 | <0.0001 |
| 5000 | <0.0001 |
| **G** | |
| 0.05 | >0.9999 |
| 0.5 | 0.9665 |
| 5 | 0.9776 |
| 50 | 0.0213 |
| 500 | <0.0001 |
| 5000 | <0.0001 |
| **H** | |
| 0.05 | 0.6638 |
| 0.5 | >0.9999 |
| 5 | 0.7957 |
| 50 | 0.0005 |
| 500 | <0.0001 |
| 5000 | <0.0001 |
| **I** | |
| 0.05 | 0.9985 |
| 0.5 | 0.9997 |
| 5 | 0.6958 |
| 50 | 0.153 |
| 500 | <0.0001 |
| 5000 | <0.0001 |
| **J** | |
| 0.05 | 0.9916 |
| 0.5 | 0.9771 |
| 5 | 0.9908 |
| 50 | 0.9924 |
| 500 | <0.0001 |
| 5000 | <0.0001 |

| | |
|---|---|
| gp33 | 0.000217 |
| np396 | 0.009475 |
| **KLRG1 %** | |
| gp33 | 0.000057 |
| np396 | 0.000074 |
| **IL7R %** | |
| gp33 | 0.116266 |
| np396 | 0.16243 |

# Appendix Figure S1

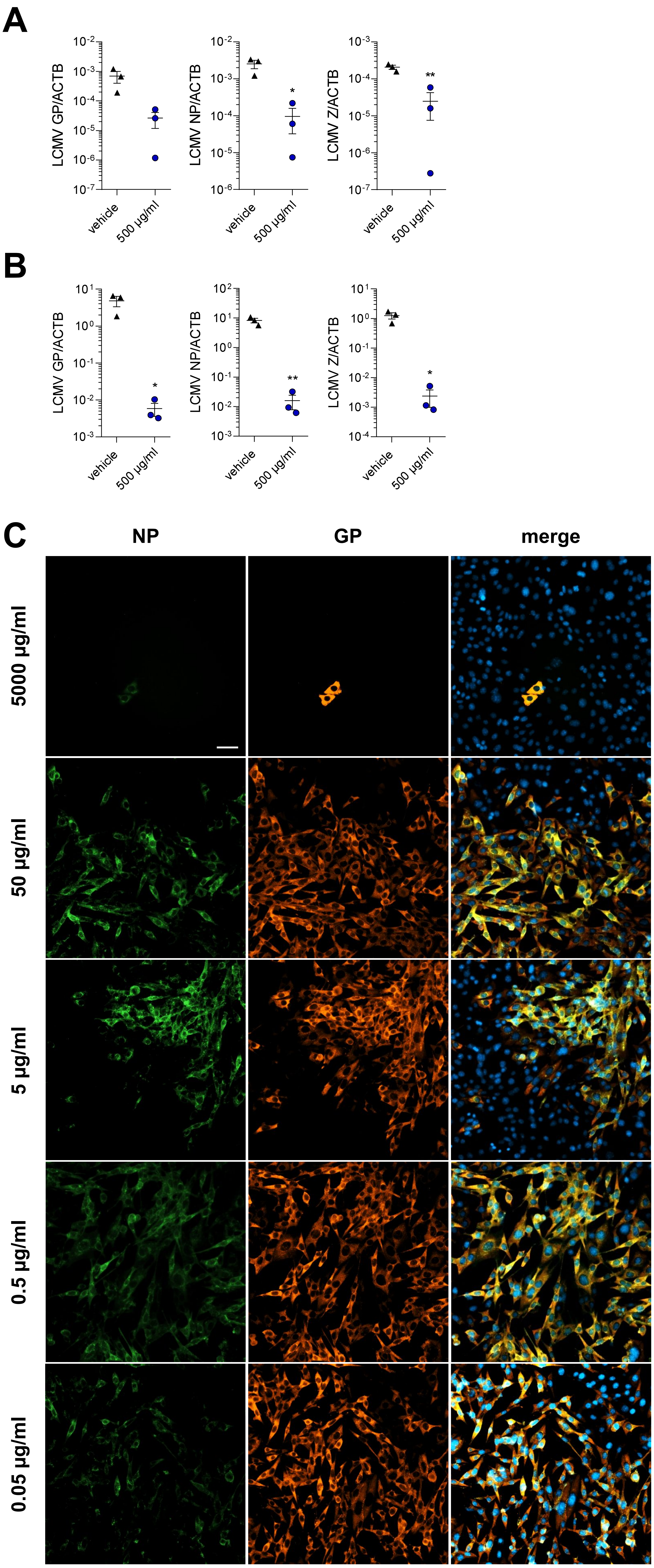

**Appendix Figure S1. Heparin decreases LCMV cell entry and replication.**

A-B. MC57G cells were treated with heparin for 1 h. Next, cells were infected with LCMV WE (MOI 0.01) for 3 h. After the infection, cells were washed three times with PBS and fresh medium containing heparin was readded to the cells. The cells were collected after 8 (A) and 24 h (B) for RNA isolation and RT-PCR. Data presented as number of cognate mRNA copies per copy of mRNA for reference housekeeping gene, mean±SEM, n=3. $^*p<0.05$, $^{**}p<0.01$ compared to vehicle control. Statistical significance was assessed by Student's *t*-test.

C. MC57G cells were treated with heparin for 1 h. Next, cells were infected with LCMV WE (MOI 0.1) for 3 h. After the infection, cells were washed three times with PBS and fresh medium containing heparin was readded to the cells. After 24 h, the cells were stained for LCMV NP and GP (one representative set of n=4 is shown; scale bar=50 µm).

# Appendix Figure S2

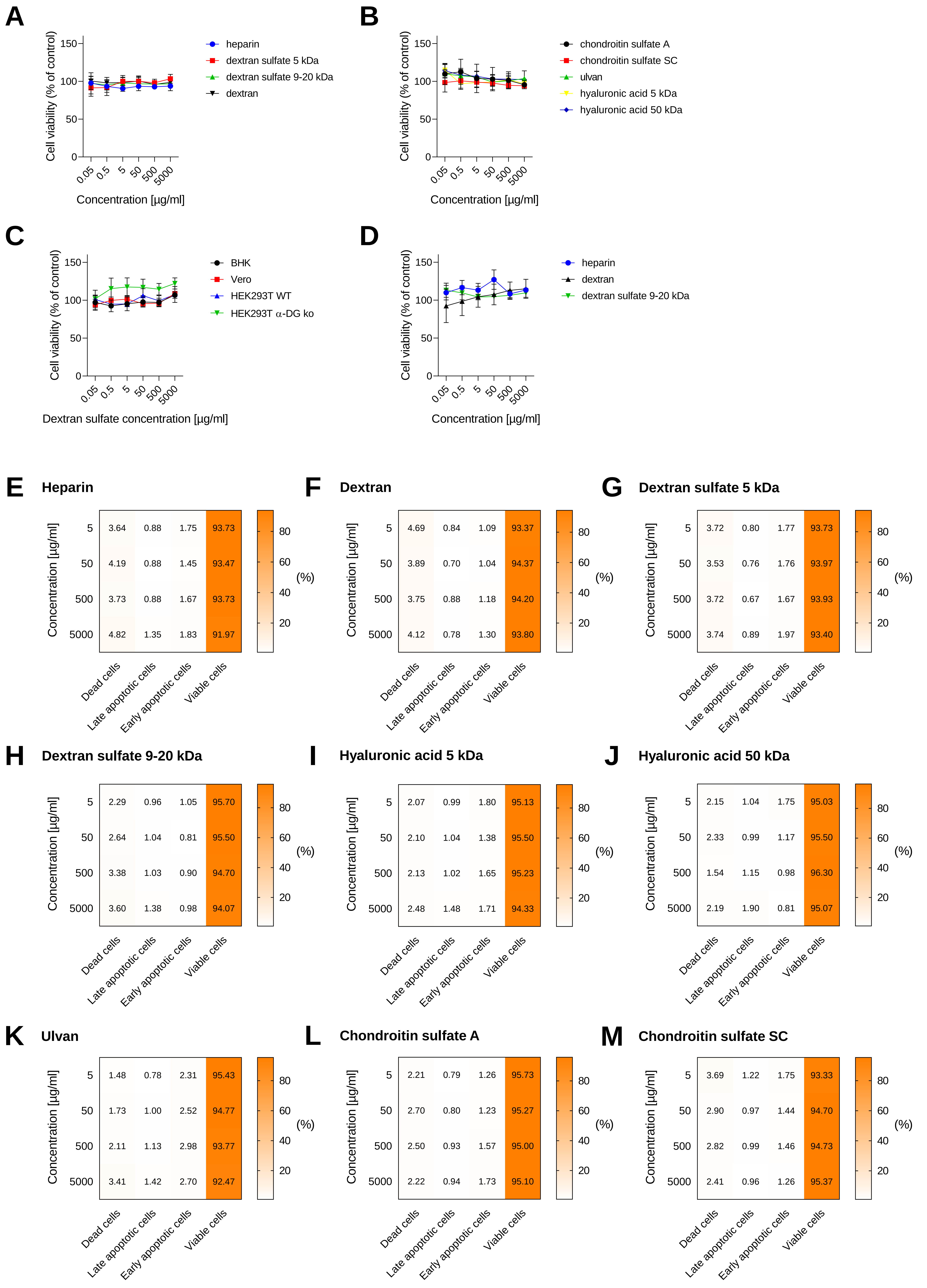

**Appendix Figure S2: Cytotoxicity analysis**

Cells were seeded into 96-well plates (20.000 cells/well) and after 24 h of incubation, treated with tested compounds. After 24 or 48 h, resazurin assay or annexin/7-AAD staining was performed.

Resazurin assay: A-B. MC57G, 24 h. C. BHK, Vero, HEK293T WT and HEK293T α-DG ko cells treated with dextran sulfate 9-20 kDa, 24 h. C. MC57G, 48 h. Data presented as mean±SEM, n=3.

Annexin/7-AAD staining (MC57G, 24 h): E. heparin; F. dextran; G. dextran sulfate 5 kDa; H. dextran sulfate 9-20 kDa; I. hyaluronic acid 5 kDa; J. hyaluronic acid 50 kDa; K. ulvan; L. chondroitin sulfate A; M. chondroitin sulfate SC. Data presented as heatmaps representing mean, n=3.

# Appendix Figure S3

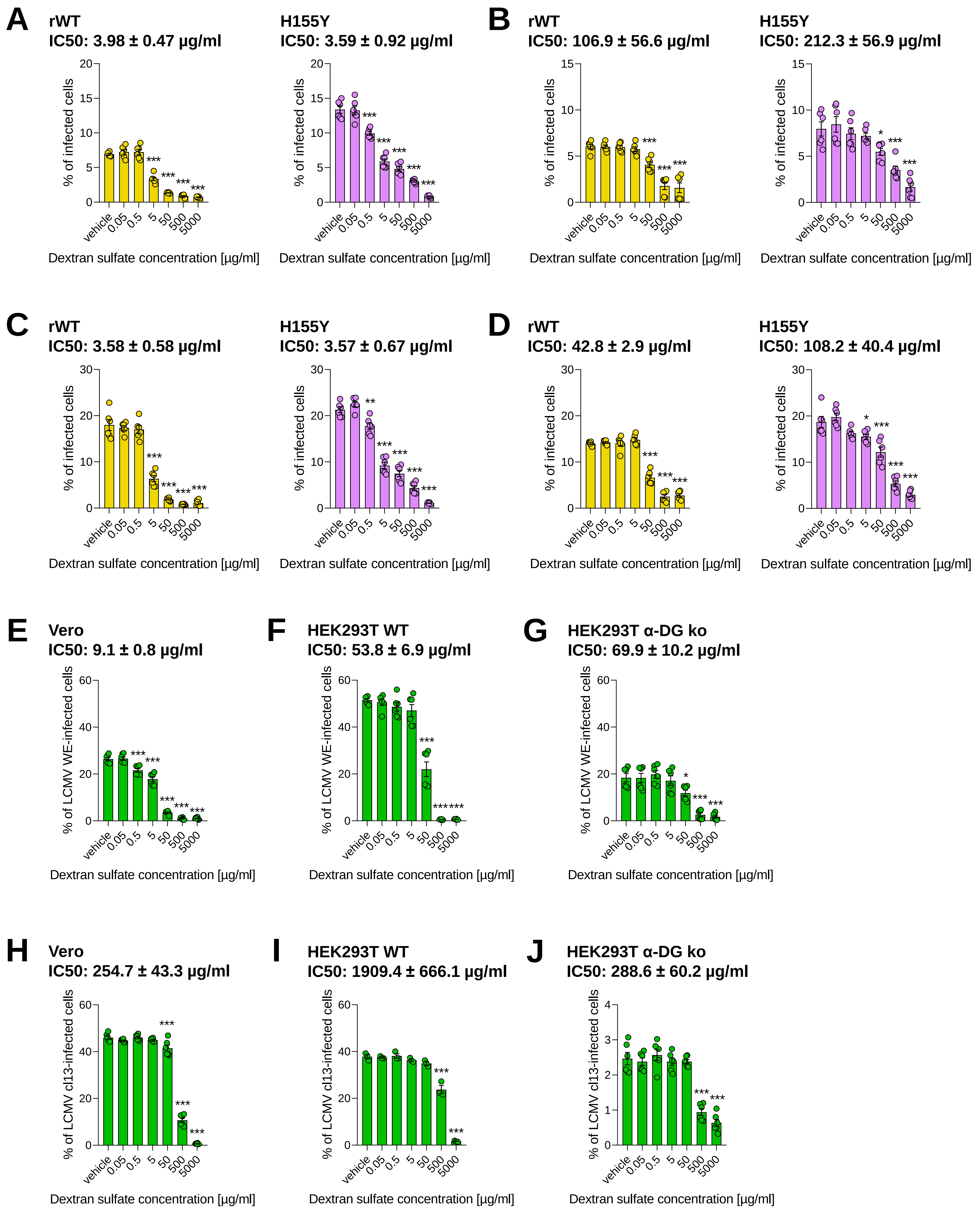

**Appendix Figure S3: Dextran sulfate inhibits cell entry of LCMV viruses with different affinity to α-dystroglycan in time- and concentration-dependent manner.**

A-D. MC57G (A, C) and BHK (D, B) cells were treated with dextran sulfate for 1 h. Next, cells were incubated with rWT (yellow) or H155Y (purple) viruses (MOI 0.5) for 1 h at 4 °C. To initiate infection, cells were moved to 37 °C for 2.5 (A, B) and 5 h (C, D) before adding monensin to block the Golgi apparatus and intracellular protein transport. Cells were then stained for viability and LCMV NP according to the standard protocol. Data presented as mean±SEM, n=6. *$p<0.05$, **$p<0.01$ ***$p<0.001$ compared to vehicle control. Statistical significance was assessed by one-way ANOVA.

E-J. Vero (E, H) and HEK293T (WT (F, I) and α-DG ko (G, J)) were treated with dextran sulfate for 1 h. Next, cells were incubated with LCMV WE (E-G) or clone 13 (Cl13, H-J) viruses (MOI 0.5) for 1 h at 4 °C. To initiate infection, cells were moved to 37 °C for 24 h before adding monensin to block the Golgi apparatus and intracellular protein transport. Cells were then stained for viability and LCMV NP according to the standard protocol. Data presented as mean±SEM, n=3-6. *$p<0.05$, ***$p<0.001$ compared to vehicle control. Statistical significance was assessed by one-way ANOVA.

# Appendix Figure S4

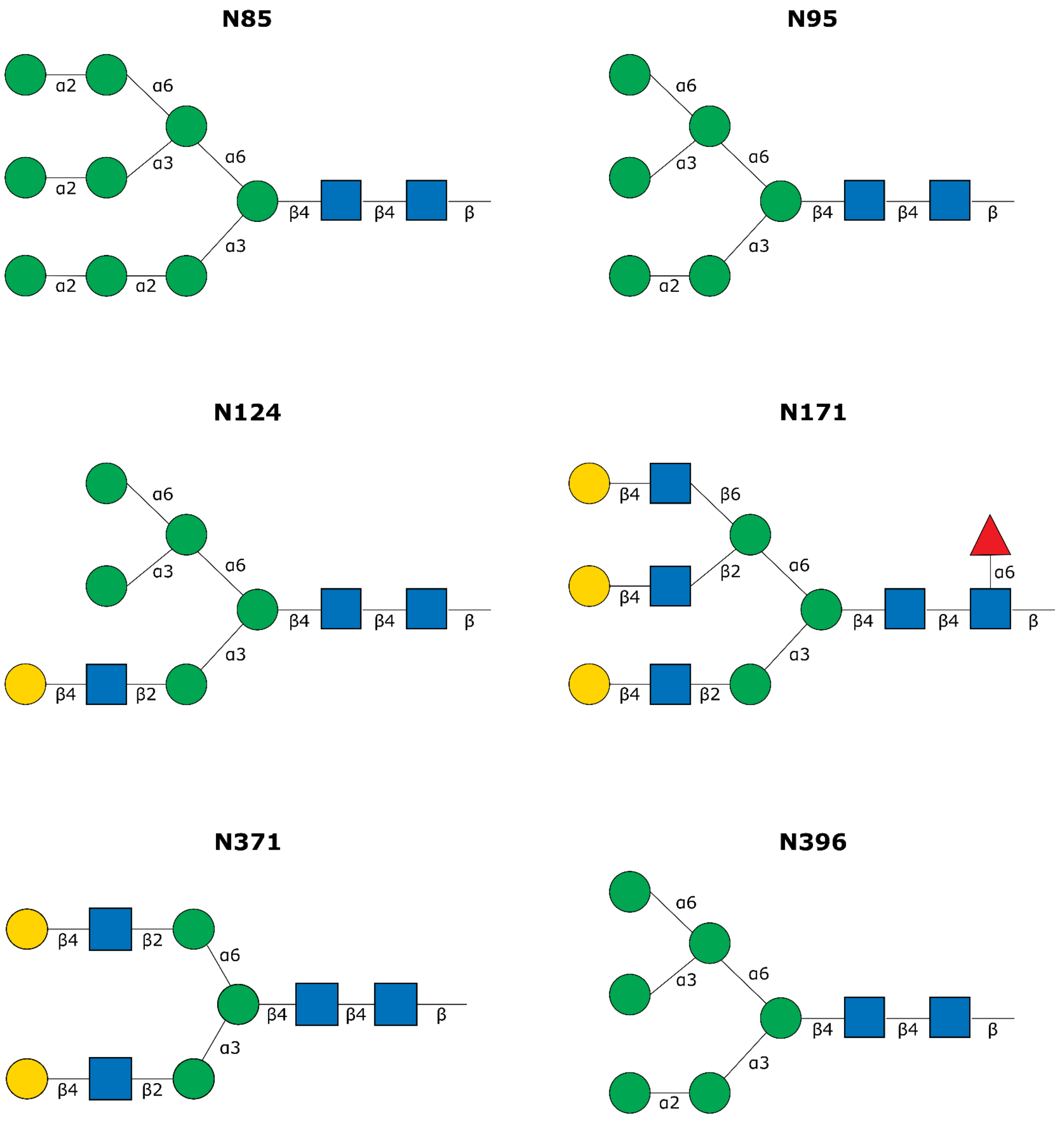

**Appendix Figure S4: Glycan compositions used for LCMV GP glycosylation.**

Glycans were adopted from experimentally derived compositions for the LASV-GP (Re and Mizuguchi 2021). The symbol nomenclature for glycans of each glycan chain is shown along with the index of the amino acid residue it is attached to.

# Appendix Figure S5

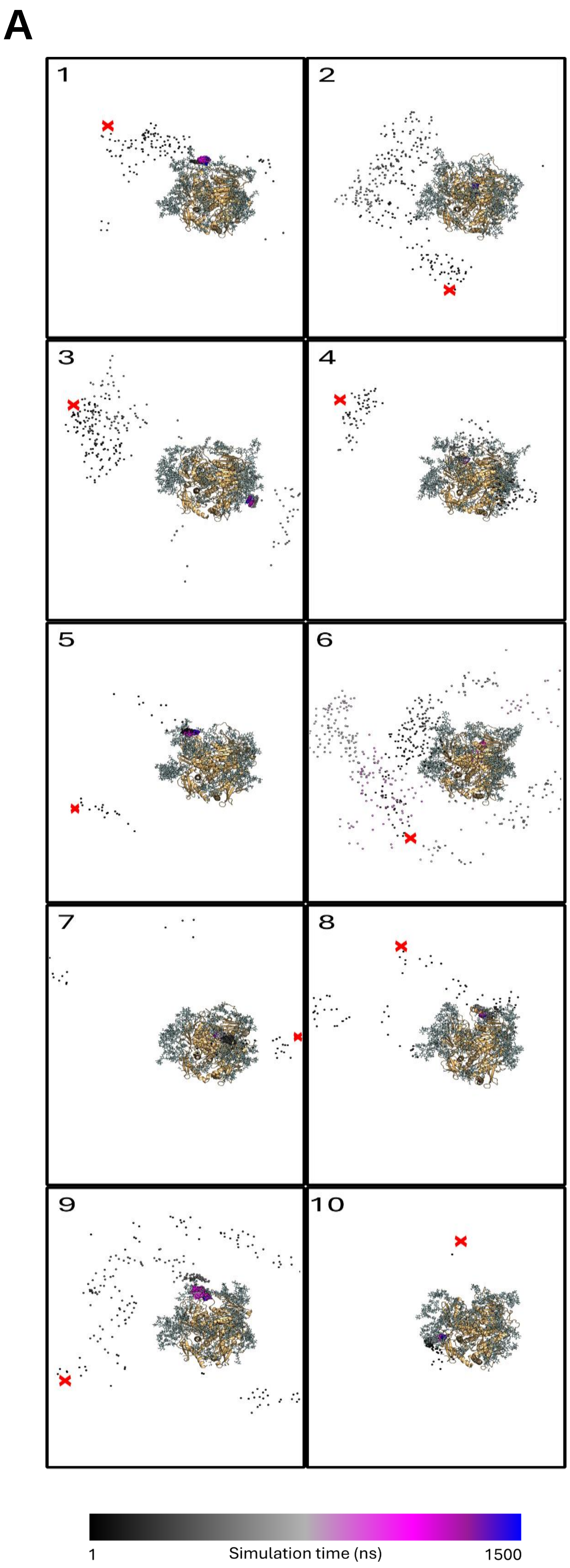


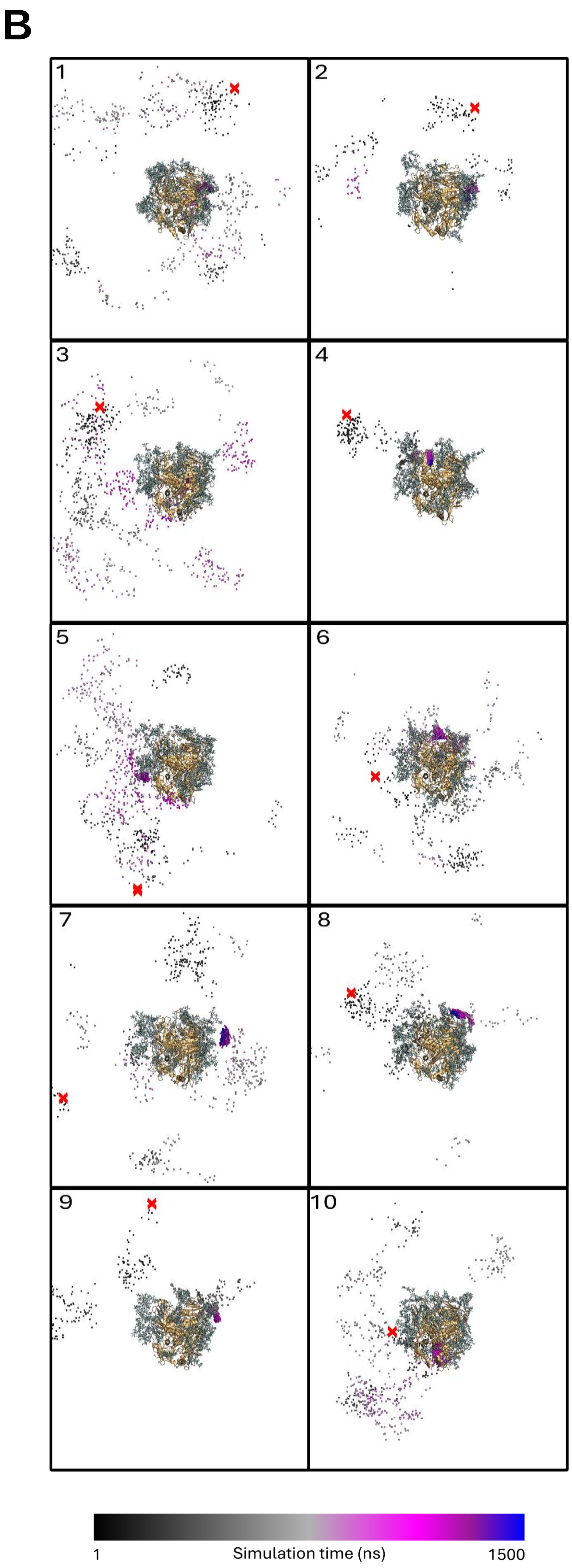

**Appendix Figure S5: Trajectories of the non-sulfated (A) and sulfated (B) dextran polymer.**

The path taken by the dextran molecule during each simulation, visualized as spheres color-coded by the simulation time (gradient: black [1 ns] to blue [1500 ns]). Each sphere represents the center of mass (COM) position of the ligand for every fifth trajectory frame, overlaid on the protein's average conformation (golden cartoon). Glycans (grey) are shown with stick representation. The starting frame of each trajectory is marked by a red cross. Coordinates of protein residues of each replica were fit to a reference state to enable comparison.

# Appendix Figure S6

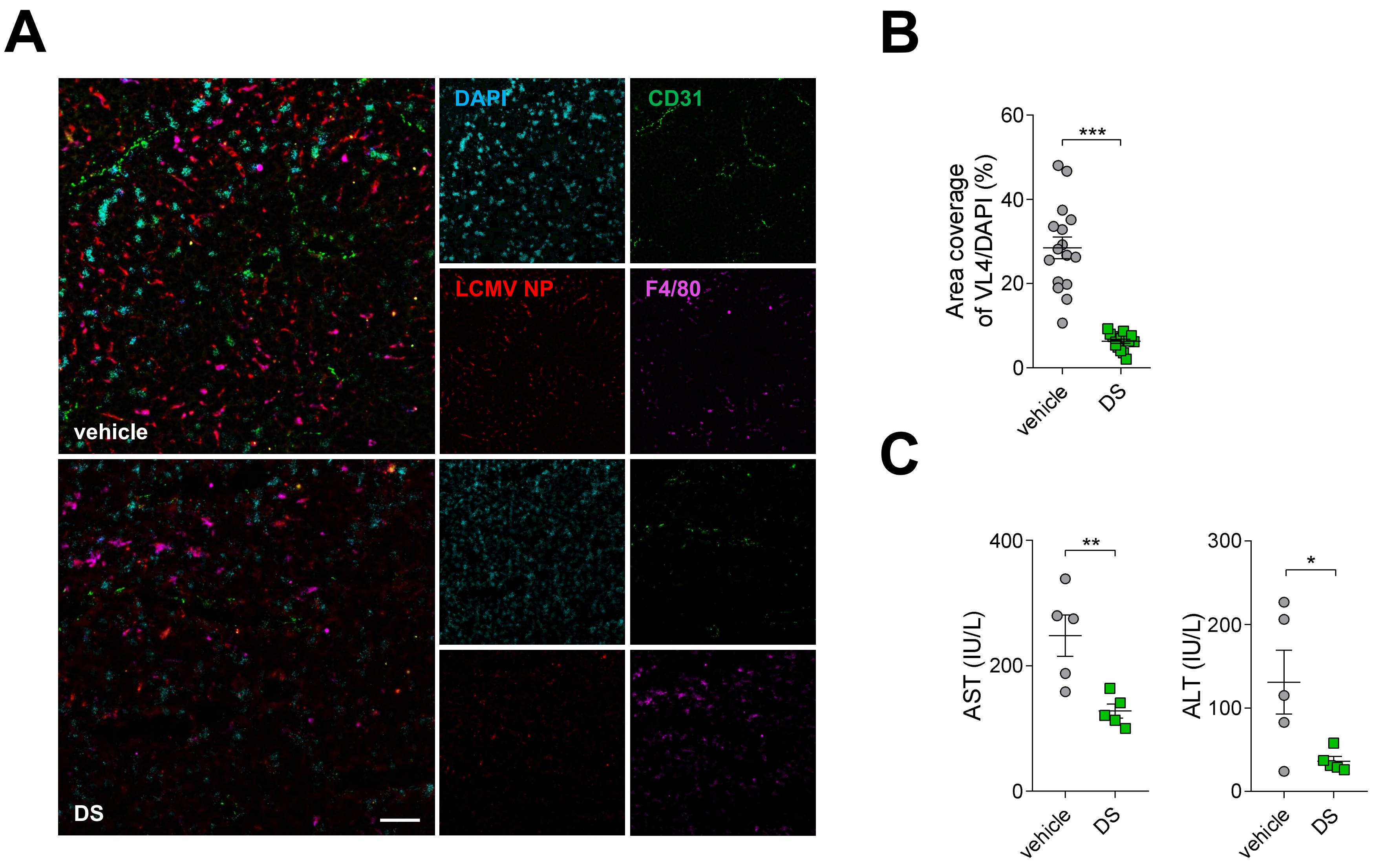

**Appendix Figure S6: Dextran sulfate does not cause liver damage.**

LCMV WE ($5 \times 10^6$ pfu) was pretreated with dextran sulfate (DS, 500 µg/ml) for 30 min., and then injected i.v. into C57BL/6J mice ($10^6$ pfu per mouse), while control mice were infected with LCMV WE. Mice were re-injected i.v. with dextran sulfate (100 µg per mouse) 6 h p.i., and control mice were injected with PBS. Analyses were carried out on day 1 p.i..

A-B. Liver tissue samples were stained for LCMV NP (clone VL4), CD31, and F4/80 (A); representative pictures of n=4 mice per condition are shown (ROI=4), scale bar=50 µm. LCMV NP staining normalized to DAPI (B) was quantified. Data presented as mean±SEM, ***p<0.001. Statistical significance was assessed by Student's *t*-test. Analysis performed in ImageJ.

C. ALT and AST activity in the serum of control and DS-treated mice was determined. Data presented as mean±SEM, n=5 mice per condition, *p<0.05, **p<0.01. Statistical significance was assessed by Student's *t*-test.

# Appendix Figure S7

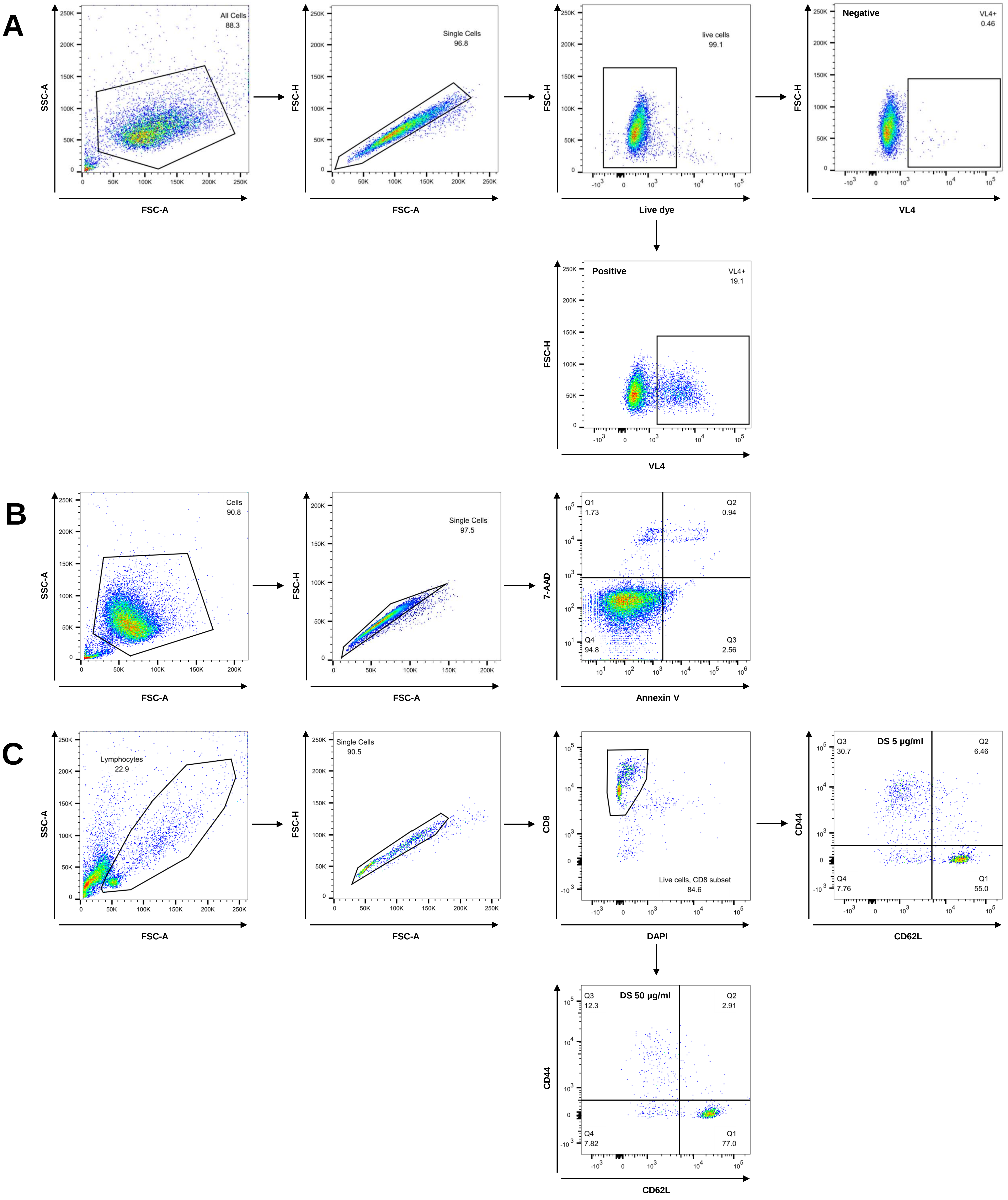

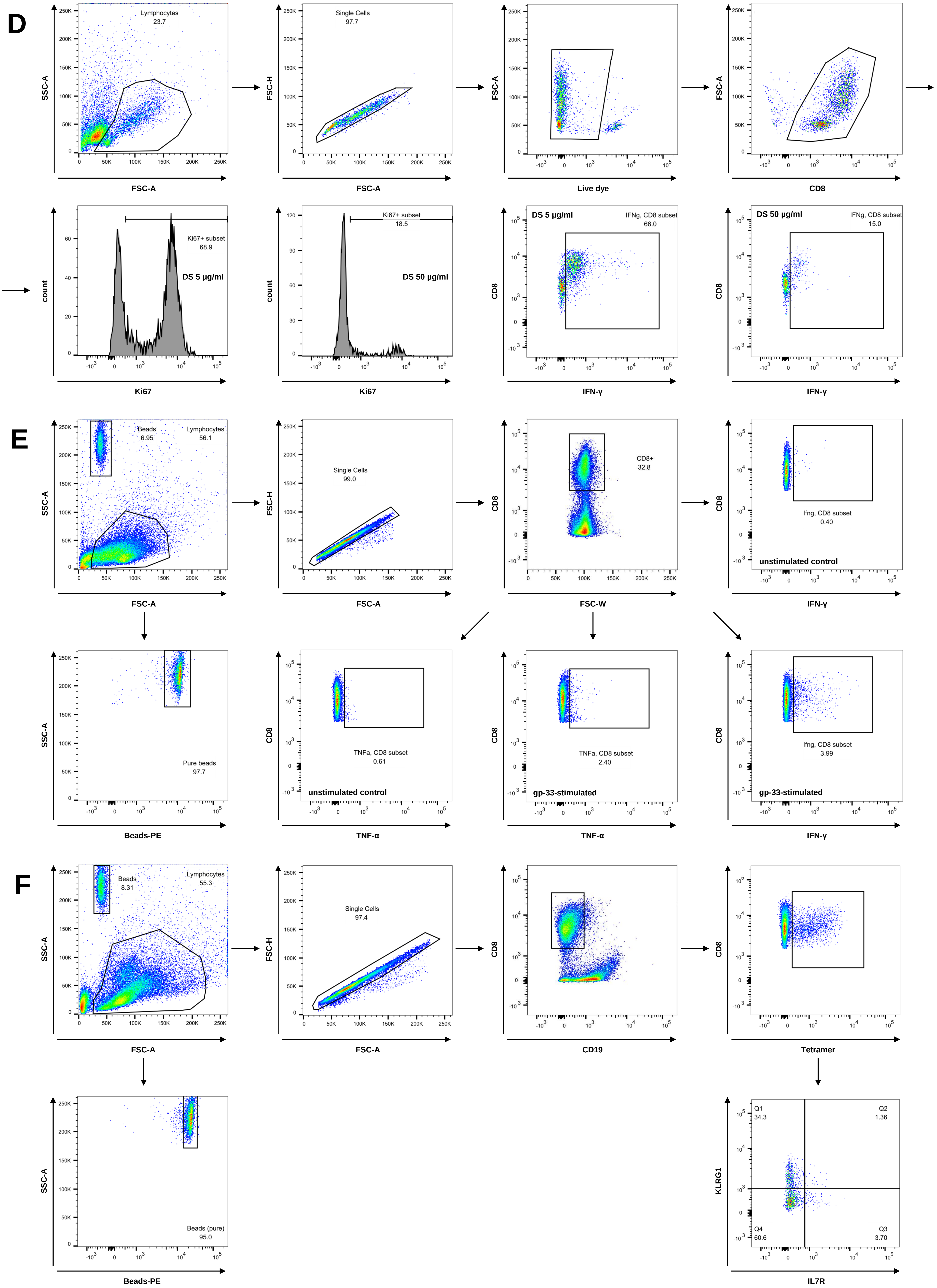
D
Lymphocytes
23.7
SSC-A
FSC-A
Single Cells
97.7
FSC-H
FSC-A
FSC-A
Live dye
FSC-A
CD8
Ki67+ subset
68.9
DS 5 µg/ml
count
Ki67
Ki67+ subset
18.5
DS 50 µg/ml
count
Ki67
DS 5 µg/ml
IFNg, CD8 subset
66.0
CD8
IFN-γ
DS 50 µg/ml
IFNg, CD8 subset
15.0
CD8
IFN-γ
E
Beads
6.95
Lymphocytes
56.1
SSC-A
FSC-A
Single Cells
99.0
FSC-H
FSC-A
CD8+
32.8
CD8
FSC-W
Ifng, CD8 subset
0.40
unstimulated control
CD8
IFN-γ
Pure beads
97.7
SSC-A
Beads-PE
TNFa, CD8 subset
0.61
unstimulated control
CD8
TNF-α
TNFa, CD8 subset
2.40
gp-33-stimulated
CD8
TNF-α
Ifng, CD8 subset
3.99
gp-33-stimulated
CD8
IFN-γ
F
Beads
8.31
Lymphocytes
55.3
SSC-A
FSC-A
Single Cells
97.4
FSC-H
FSC-A
CD8
CD19
CD8
Tetramer
Beads (pure)
95.0
SSC-A
Beads-PE
Q1
34.3
Q2
1.36
Q4
60.6
Q3
3.70
KLRG1
IL7R

**Appendix Figure S7: Gating strategies**

A. Entry assay. B. Apoptosis assay. C. T cell proliferation – effector populations. D. BMDCs:T cells co-culture – intracellular cytokine staining and Ki67 staining. E. Intracellular cytokine staining in samples collected during *in vivo* experiments. F. Tetramer staining in samples collected during *in vivo* experiments.

# Appendix Figure S8

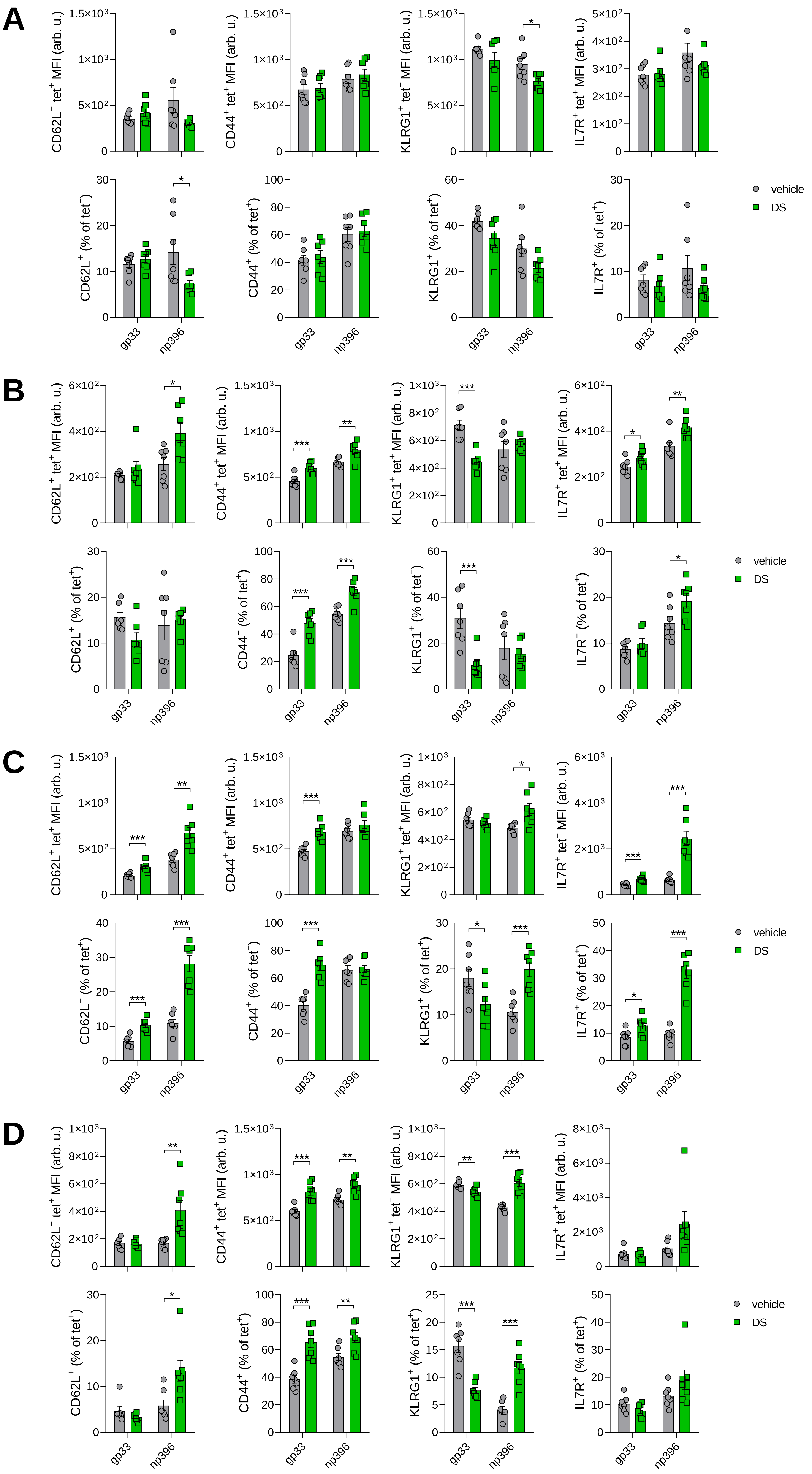

**Appendix Figure S8: Surface activation marker expression on tet$^{+}$ T cells after treatment with dextran sulfate at the beginning of infection.**

The expression of CD44, CD62L, IL7R and KLRG1 has been reported as frequency of tet$^{+}$ T cells and MFI: A. Blood on day 8 p.i. B. Blood on day 12 p.i. C. Spleen on day 12 p.i. D. Liver on day 12 p.i. Data presented as mean±SEM, n=7 mice per condition, *p<0.05, **p<0.01, ***p<0.001. Statistical significance was assessed by Student's t-test.

# Appendix Figure S9

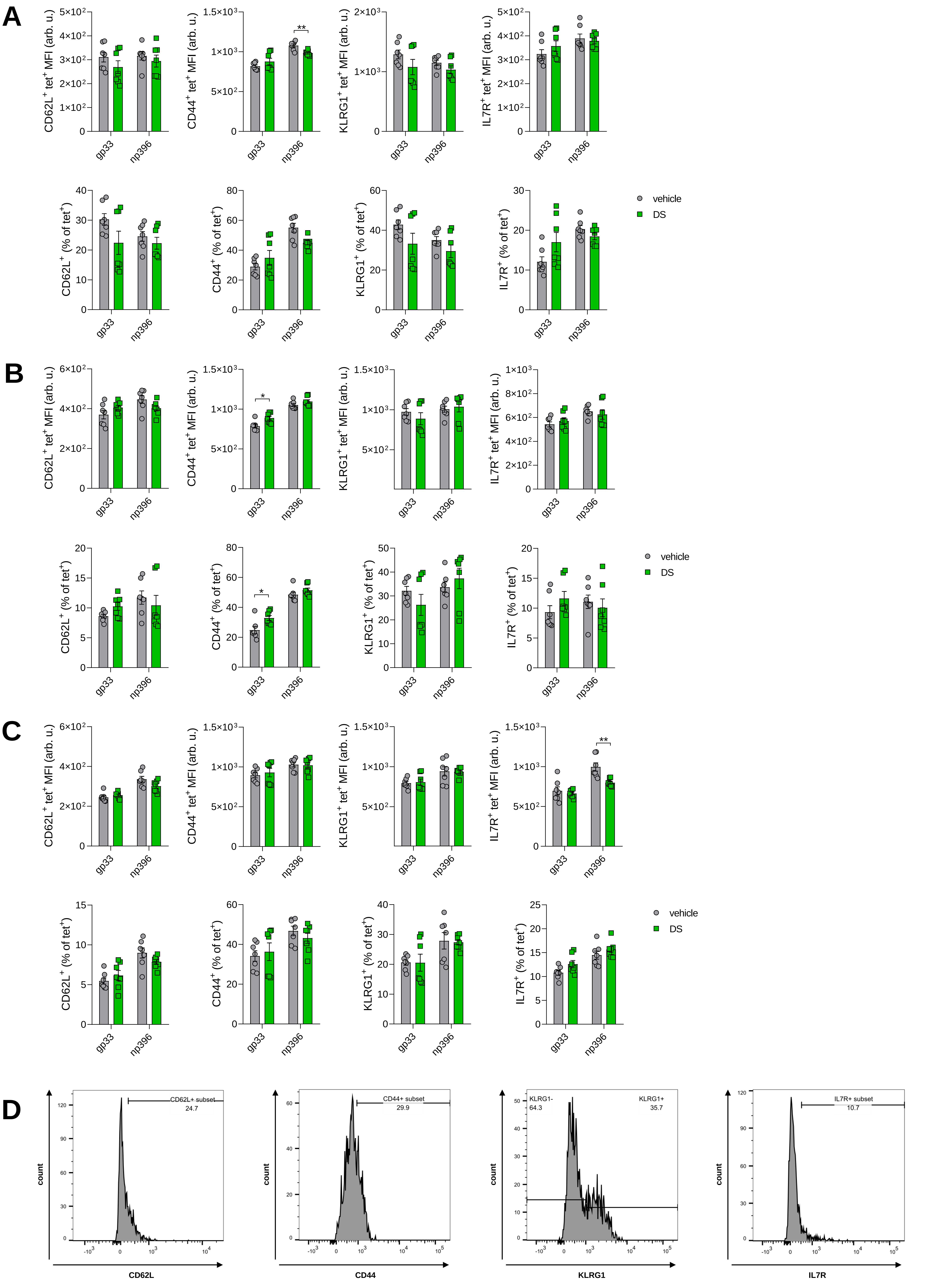

**Appendix Figure S9: Surface activation marker expression on tet$^+$ T cells after treatment with dextran sulfate in the course of acute infection.**

The expression of CD44, CD62L, IL7R and KLRG1 has been reported as frequency of tet$^+$ T cells and MFI: A. Blood on day 8 p.i. B. Spleen on day 8 p.i. C. Liver on day 8 p.i. Data presented as mean±SEM, n=7 mice per condition, *$p<0.05$, **$p<0.01$. Statistical significance was assessed by Student's t-test. D. Representative histograms for CD44, CD62L, IL7R and KLRG1.
