# Supplementary material for: Sulfated glycosaminoglycans inhibit LCMV entry and modulate antiviral immunity and pathology
Source: EMBO Mol Med. 2026 Feb 23;18(4):1235–64. doi: 10.1038/s44321-026-00387-8 (PMC13083911; doi:10.1038/s44321-026-00387-8)
Supplement: Supplementary file 3 — Source data Fig. 1 [file 44321_2026_387_MOESM3_ESM.zip › Fig. 1/1F/n=4.docx]

| **n=4** | **GP** | **NP** | **merge** |
| --- | --- | --- | --- |
| **negative control 1** | 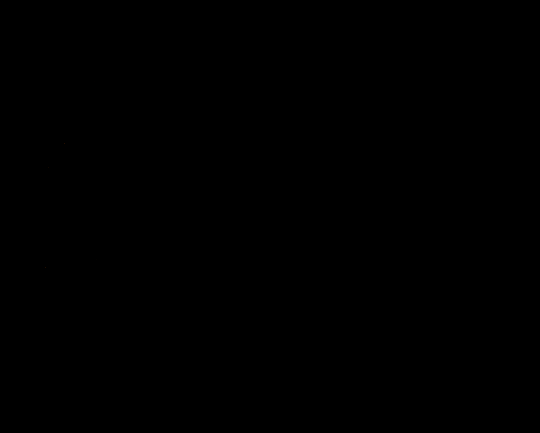 | 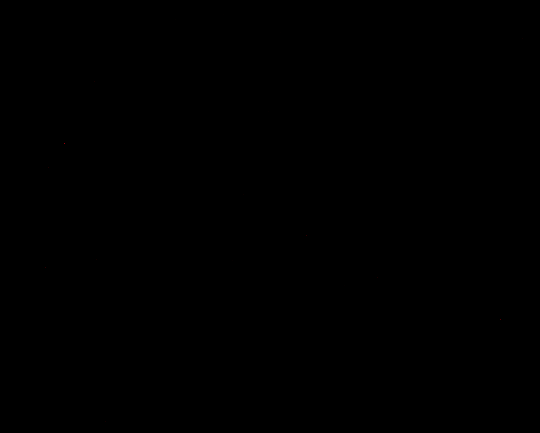 | 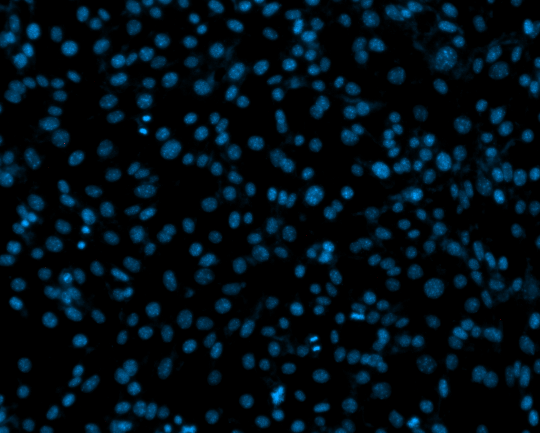 |
| **negative control 2** | 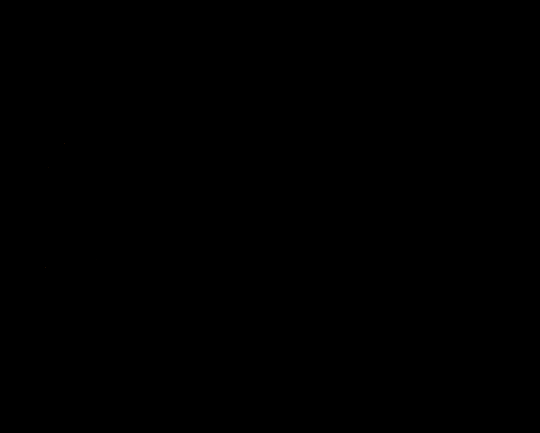 | 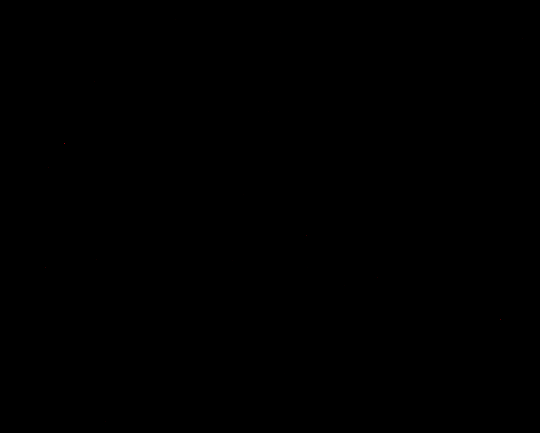 | 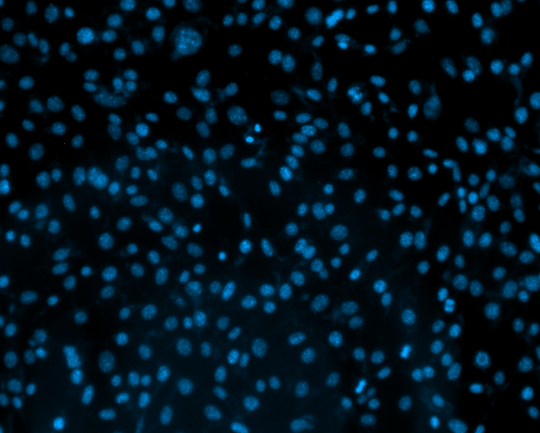 |
| **negative control 3** | 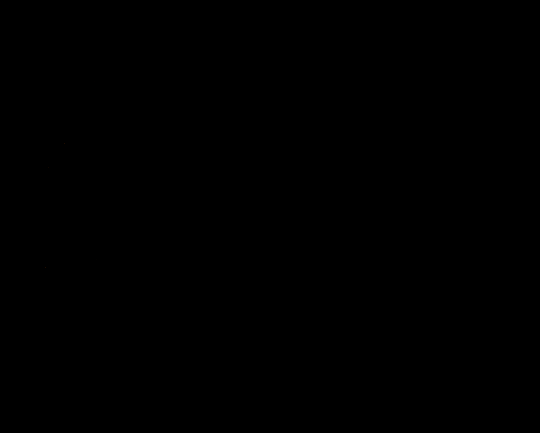 | 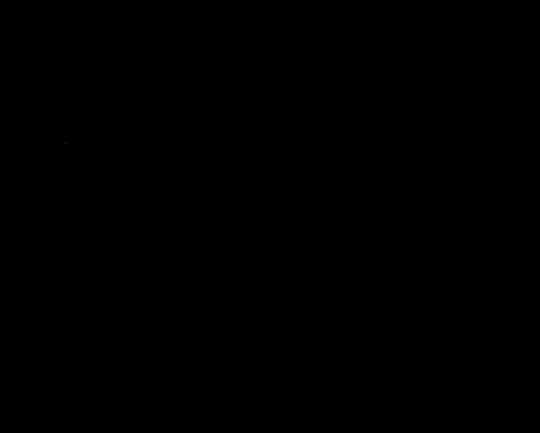 | 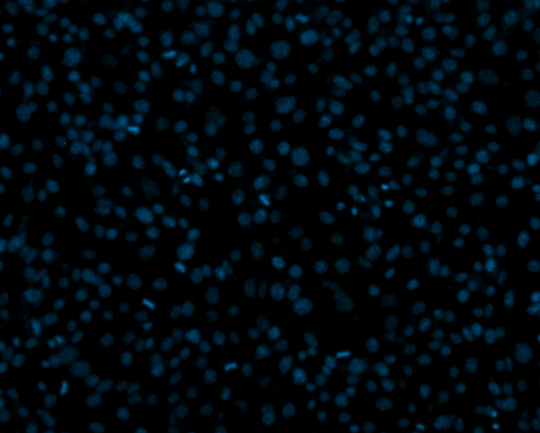 |
| **negative control 4** | 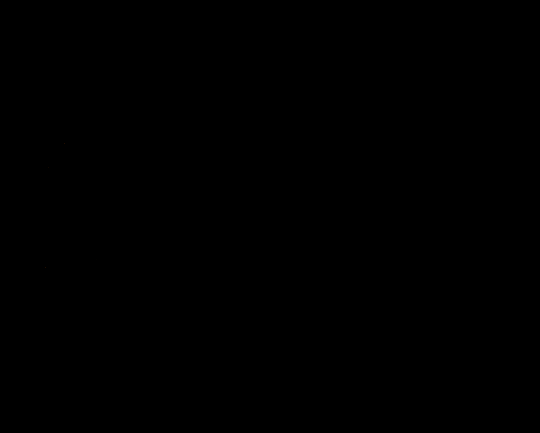 | 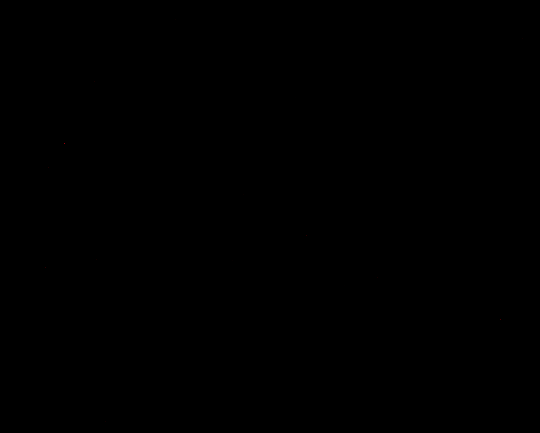 | 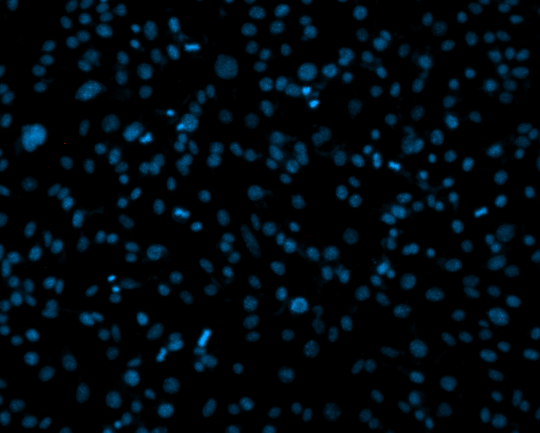 |
| **positive control 1** | 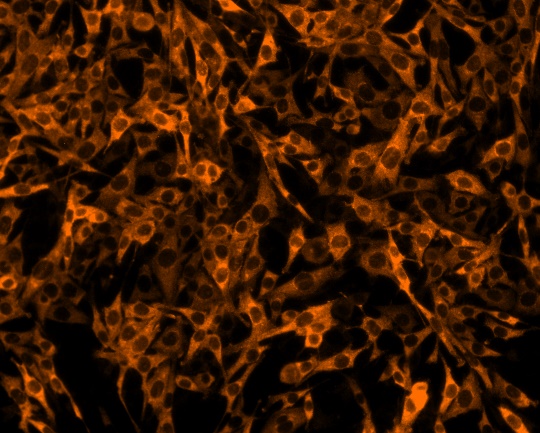 | 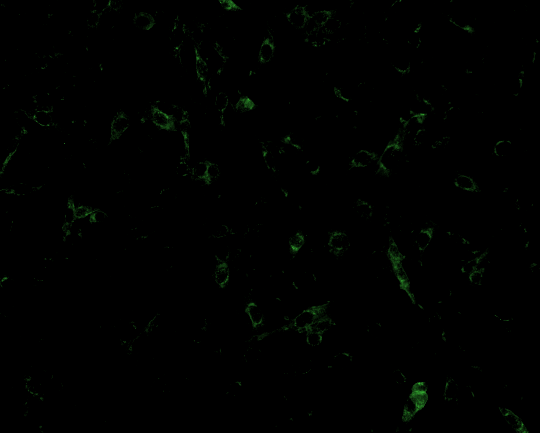 | 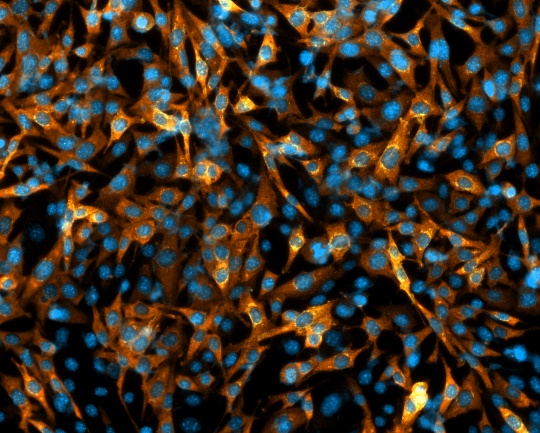 |
| **positive control 2** | 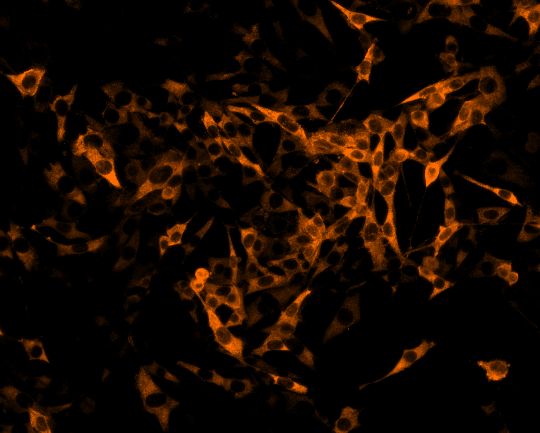 | 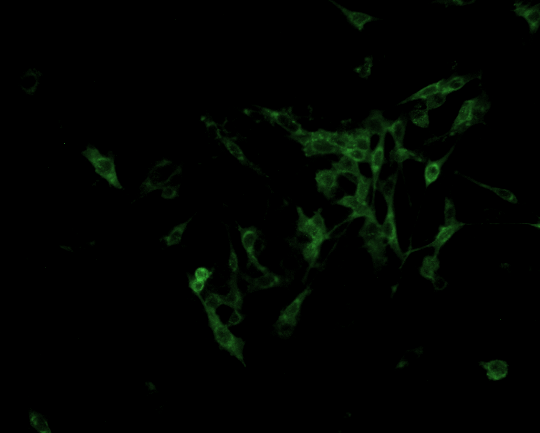 | 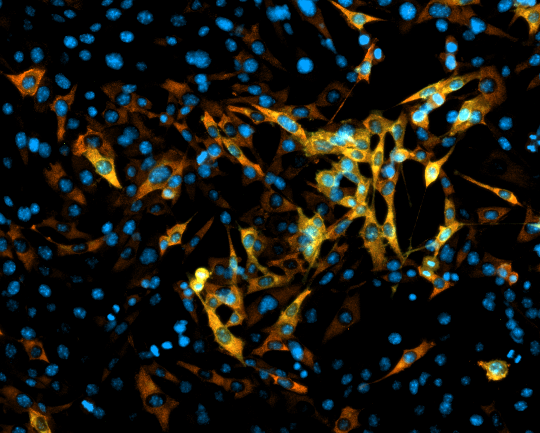 |
| **positive control 3** | 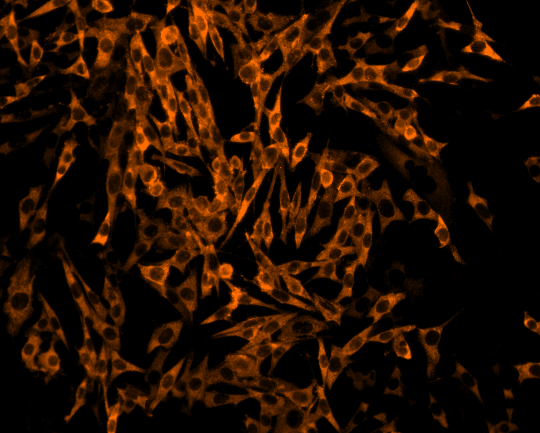 | 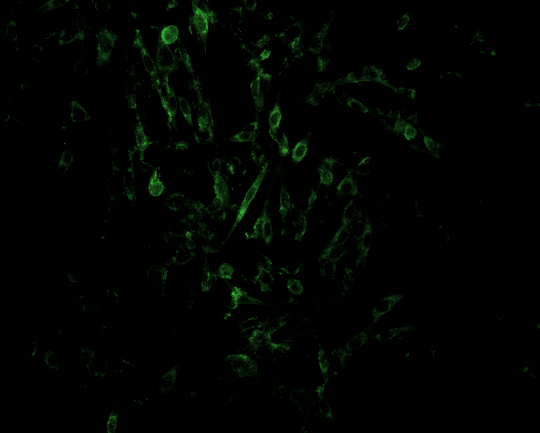 | 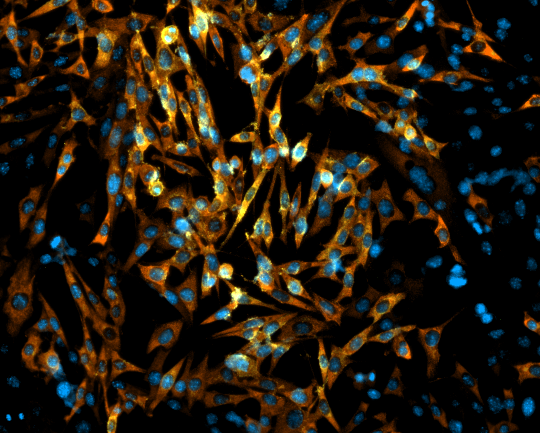 |
| **positive control 4** | 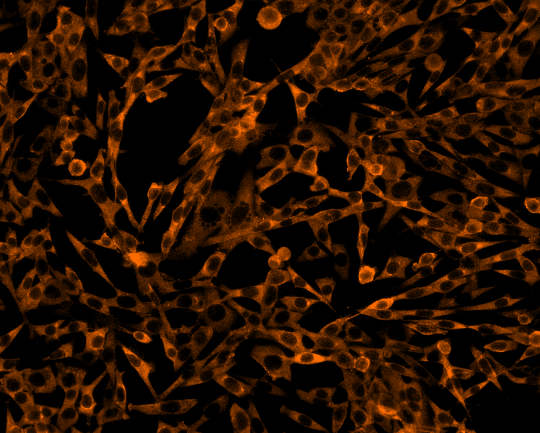 | 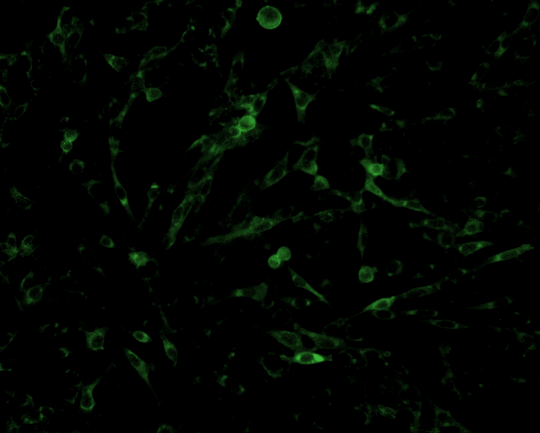 | 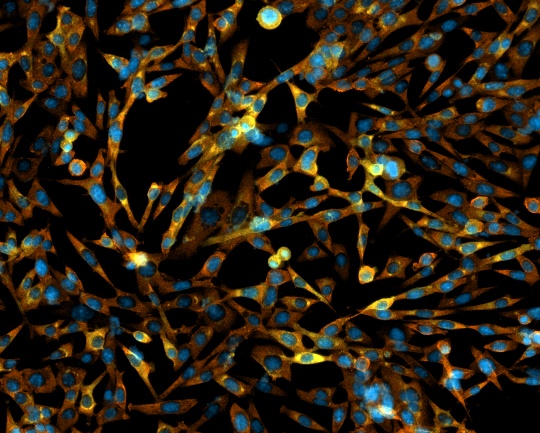 |
| **heparin 5000 µg/ml 1** | 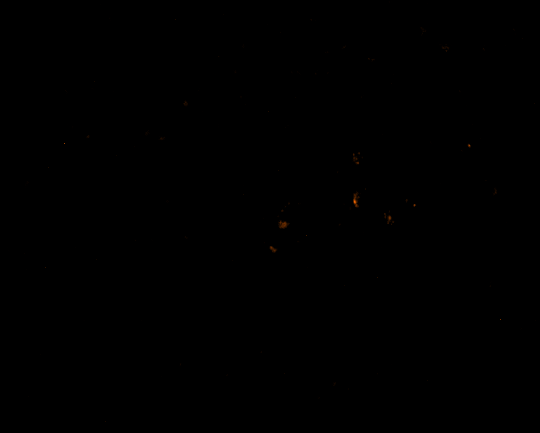 | 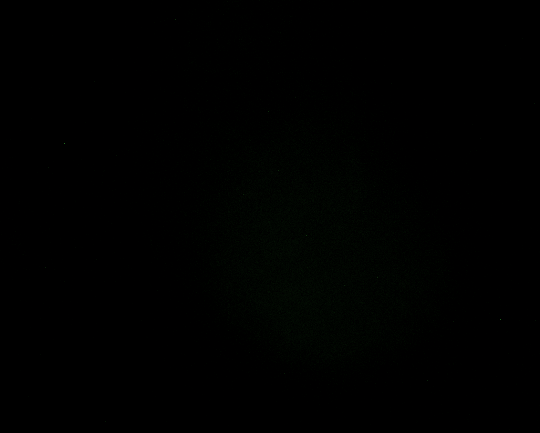 | 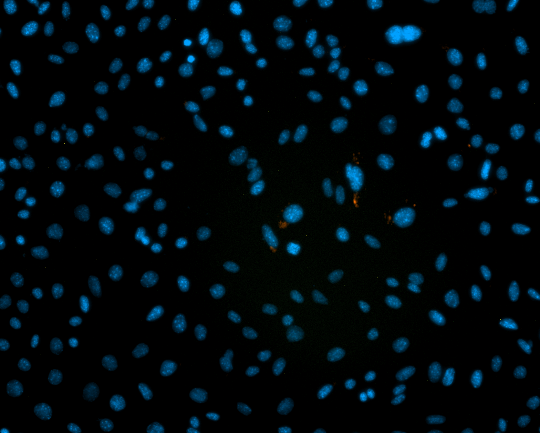 |
| **heparin 5000 µg/ml 2** | 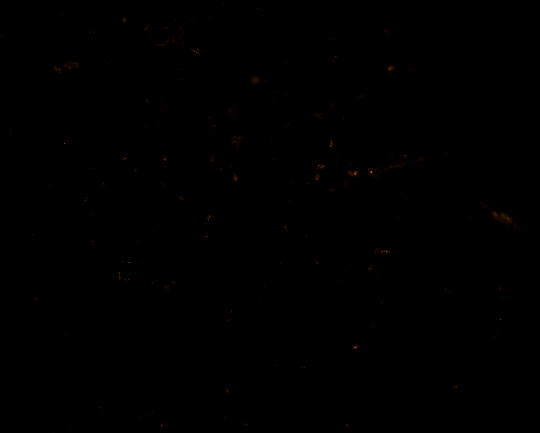 | 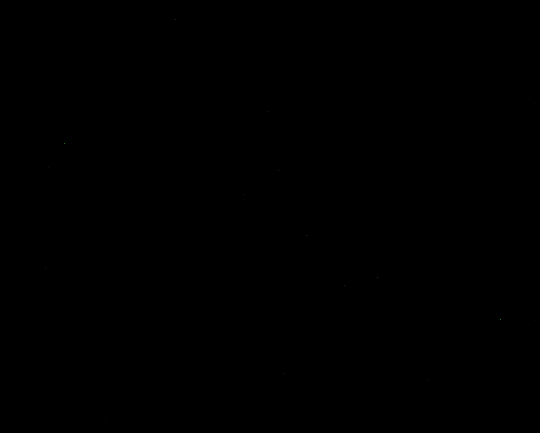 | 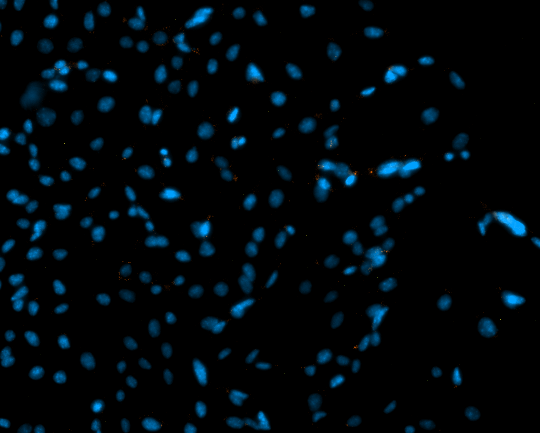 |
| **heparin 5000 µg/ml 3** | 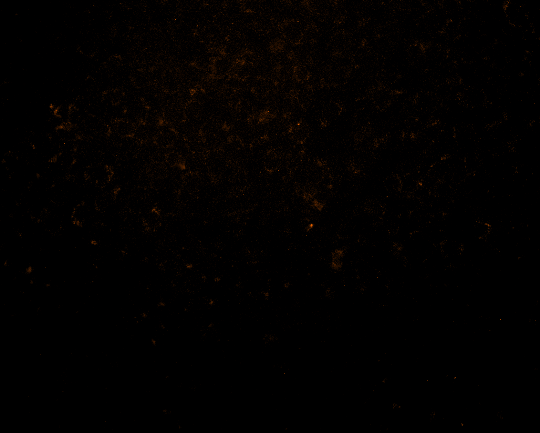 | 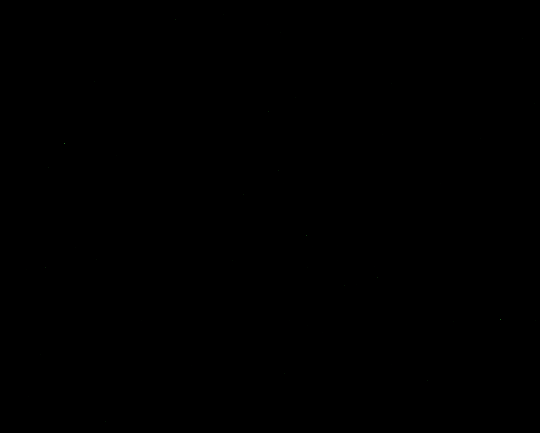 | 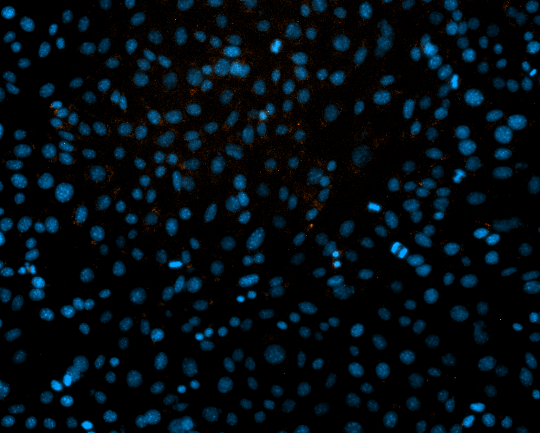 |
| **heparin 5000 µg/ml 4** | 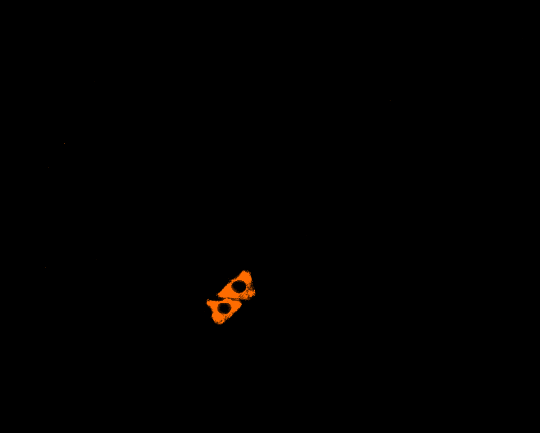 | 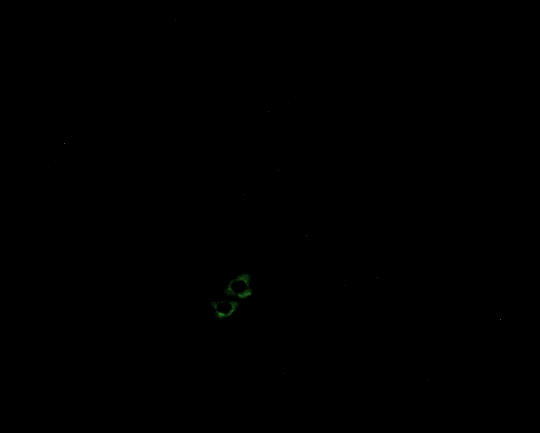 | 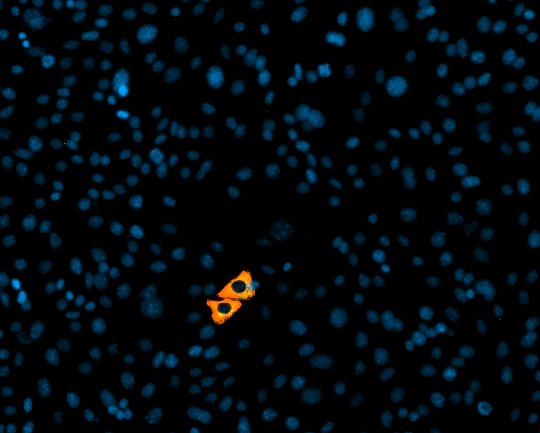 |
| **heparin 500 µg/ml 1** | 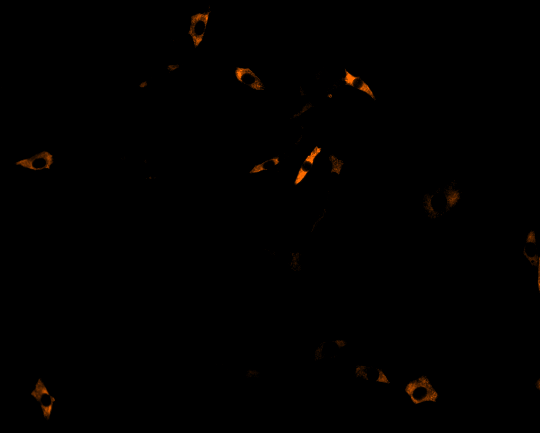 | 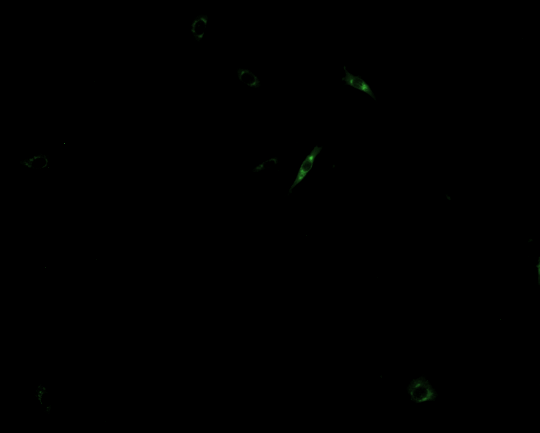 | 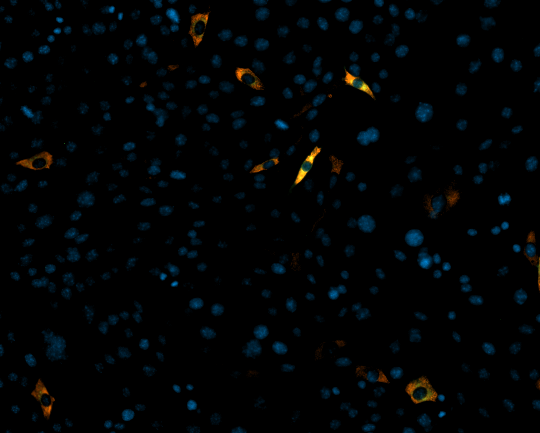 |
| **heparin 500 µg/ml 2** | 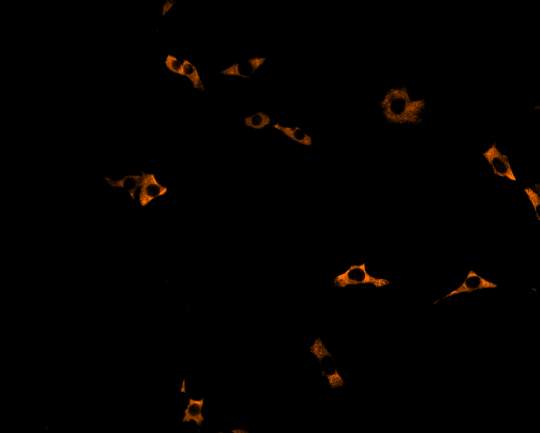 | 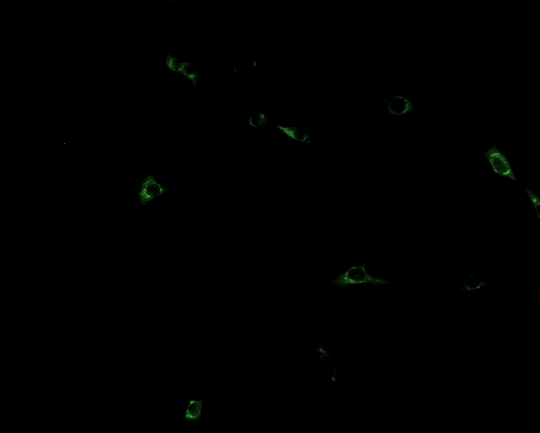 | 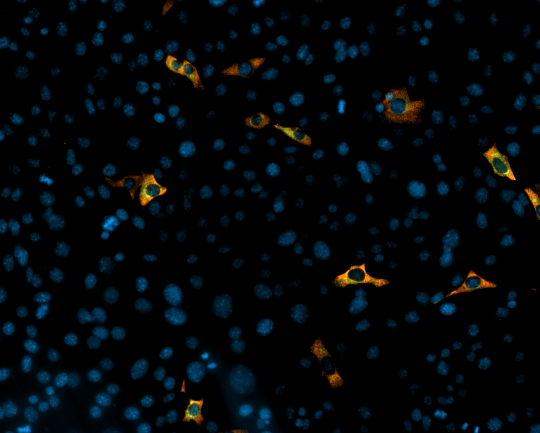 |
| **heparin 500 µg/ml 3** | 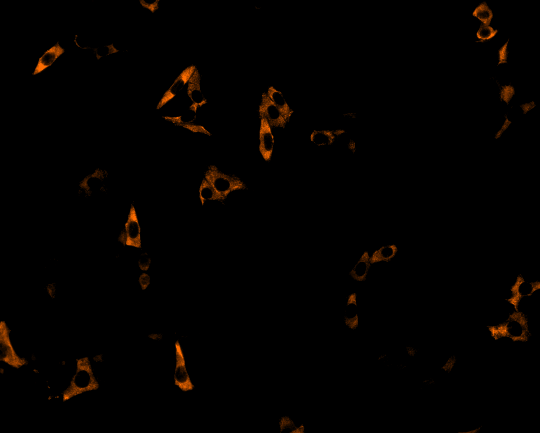 | 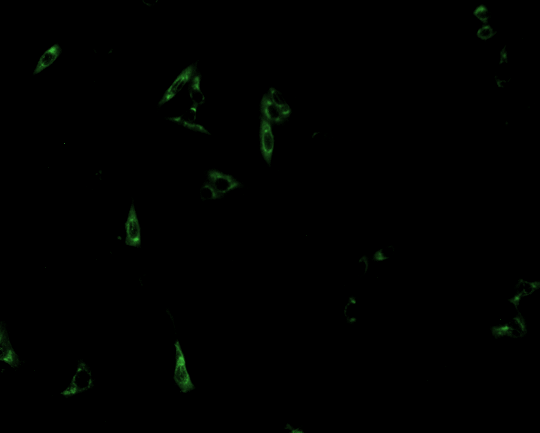 | 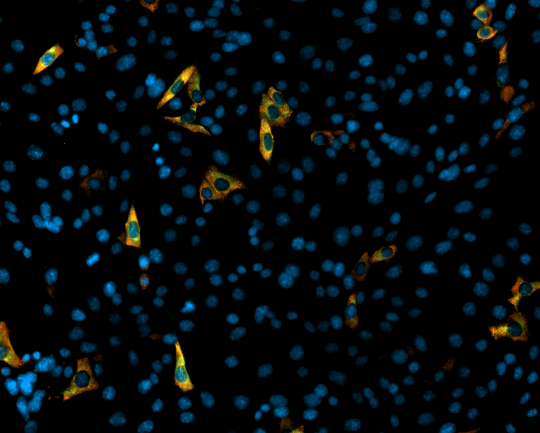 |
| **heparin 500 µg/ml 4** | 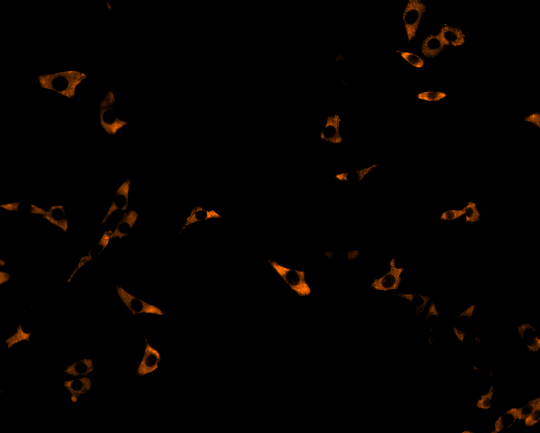 | 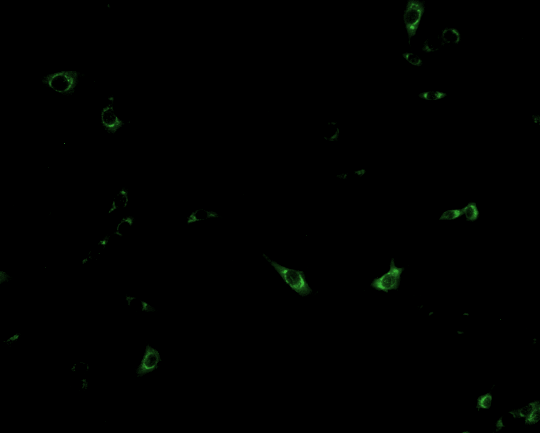 | 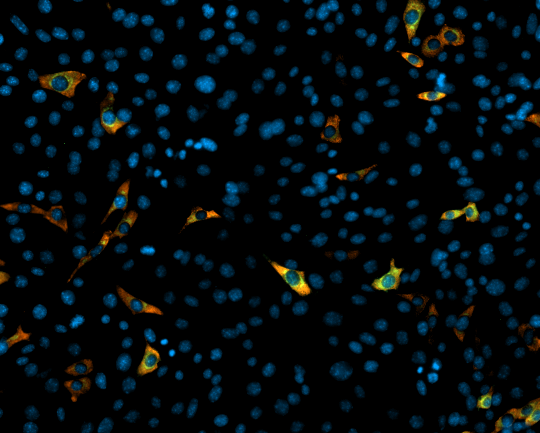 |
| **heparin 50 µg/ml 1** | 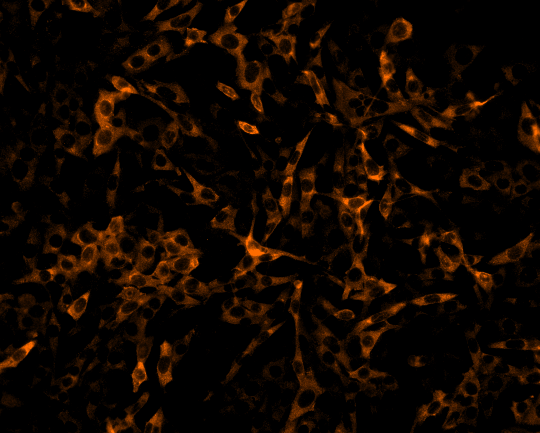 | 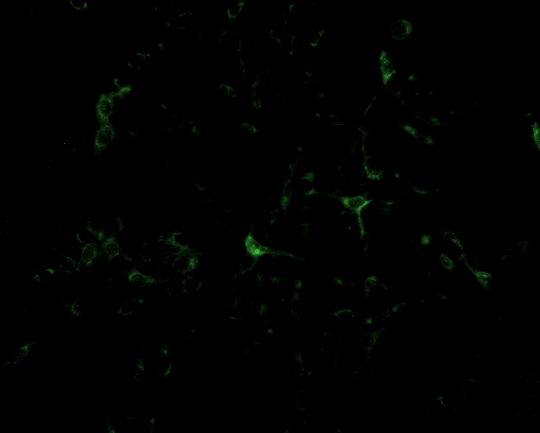 | 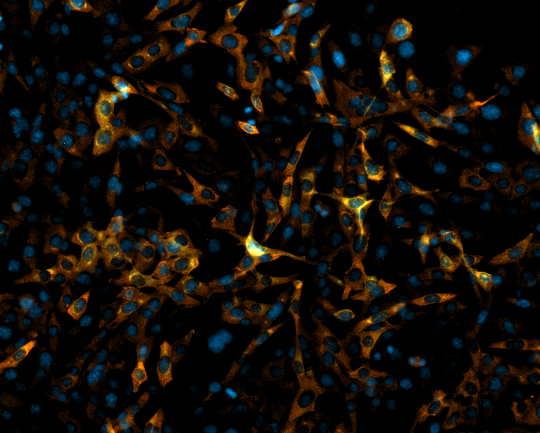 |
| **heparin 50 µg/ml 2** | 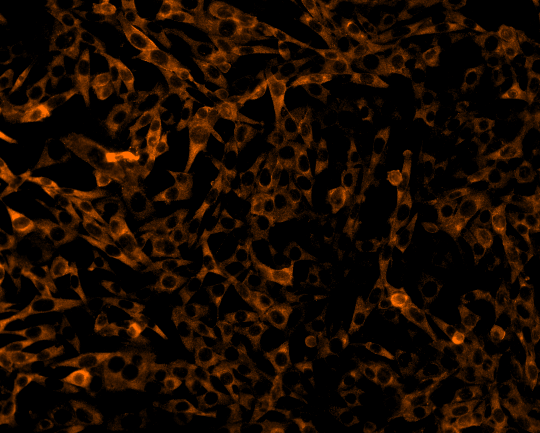 | 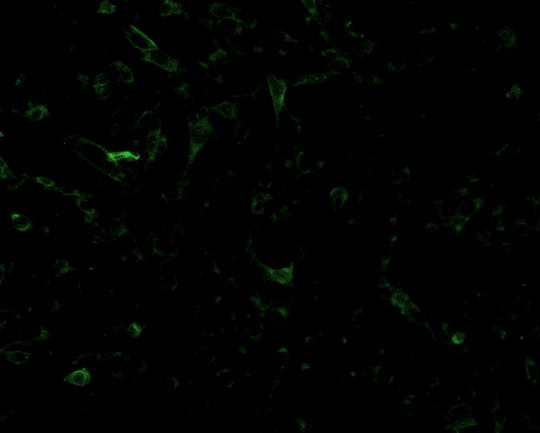 | 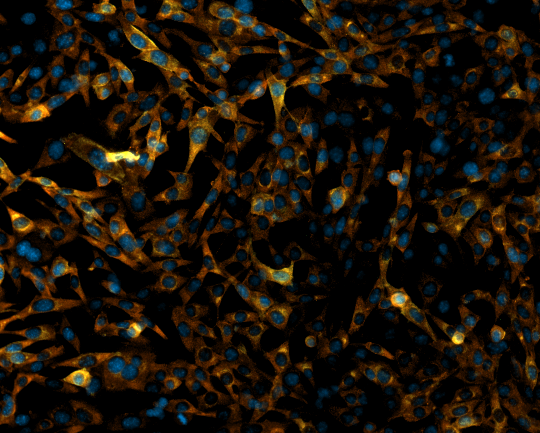 |
| **heparin 50 µg/ml 3** | 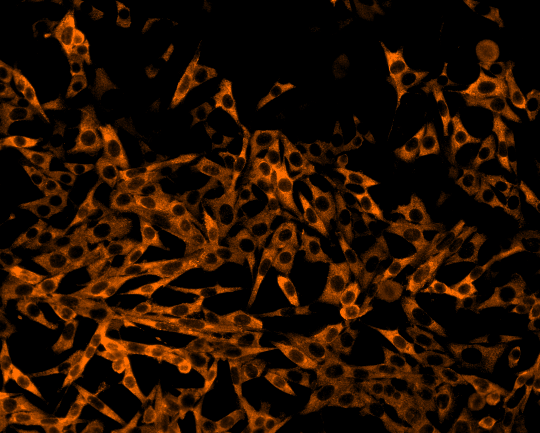 | 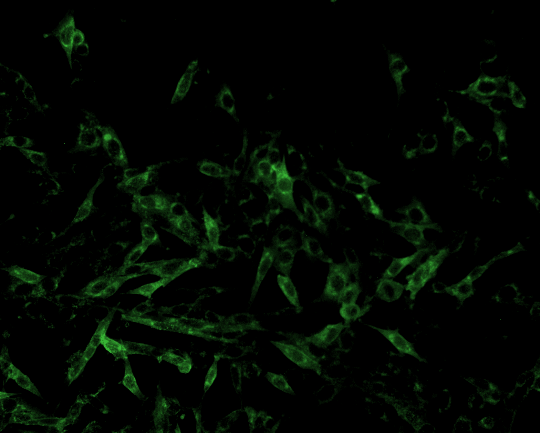 | 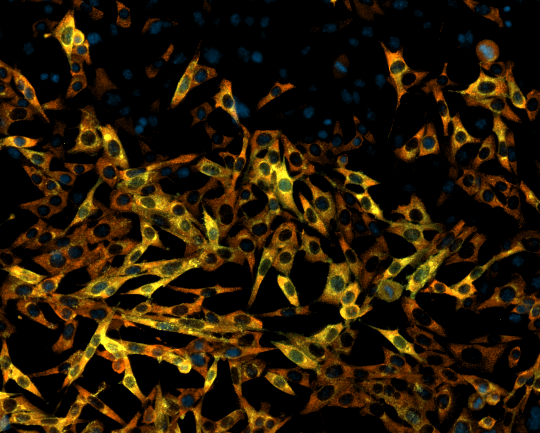 |
| **heparin 50 µg/ml 4** | 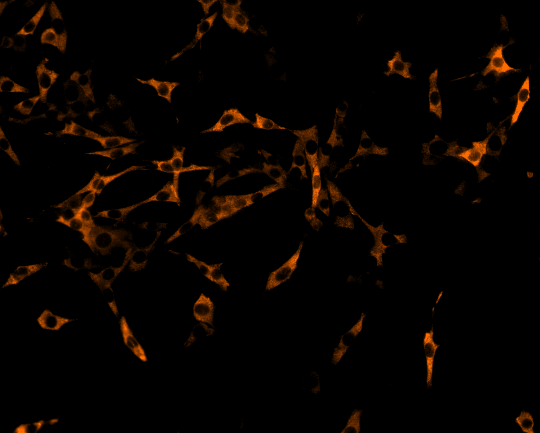 | 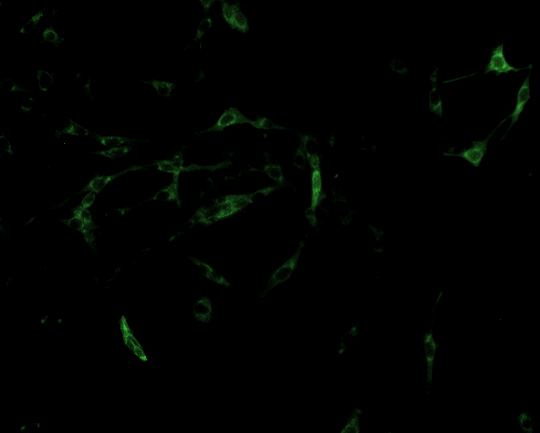 | 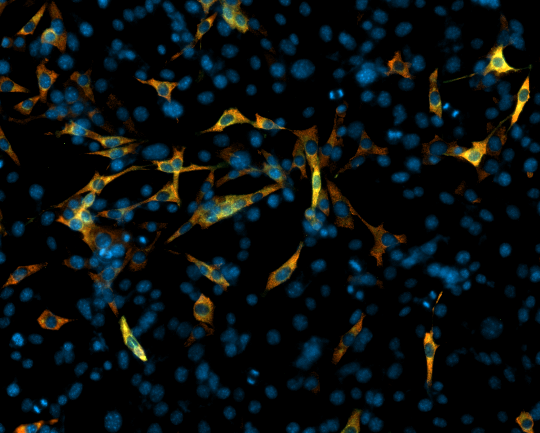 |
| **heparin 5 µg/ml 1** | 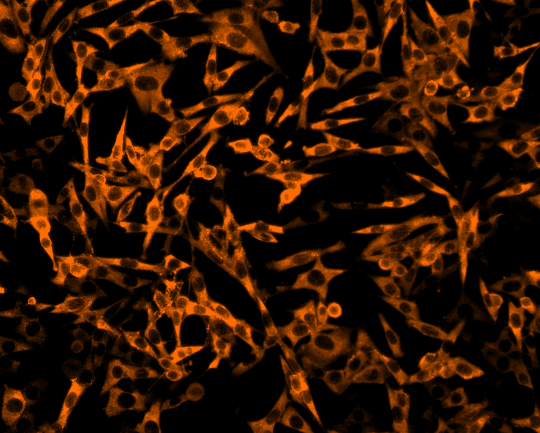 | 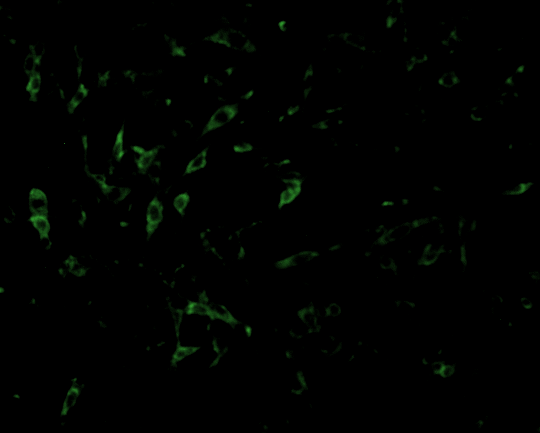 | 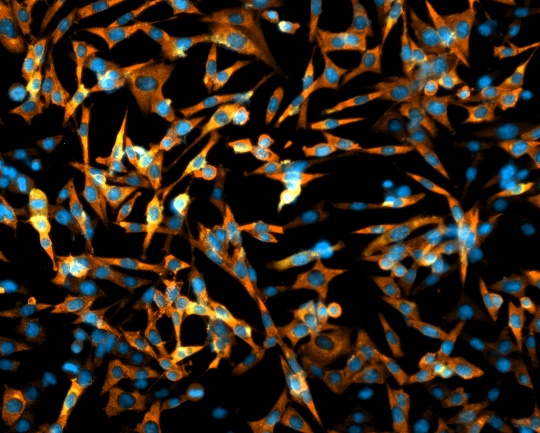 |
| **heparin 5 µg/ml 2** | 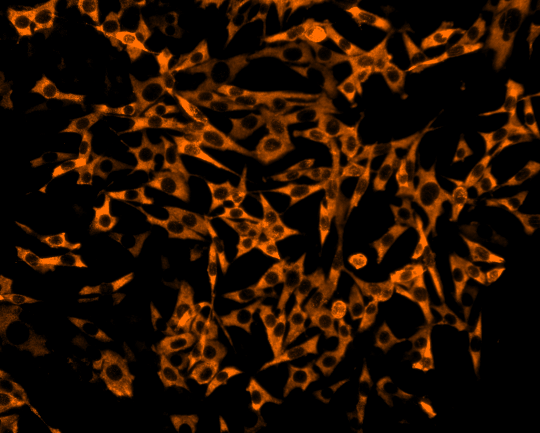 | 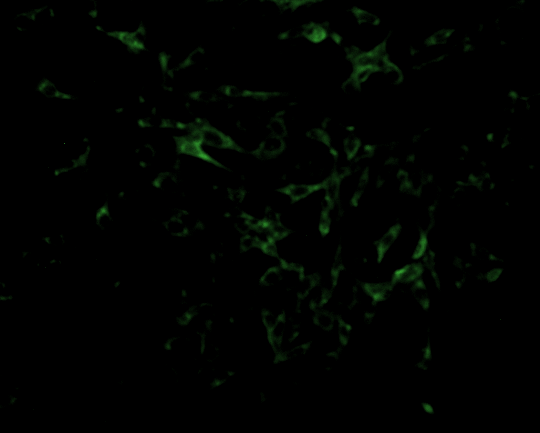 | 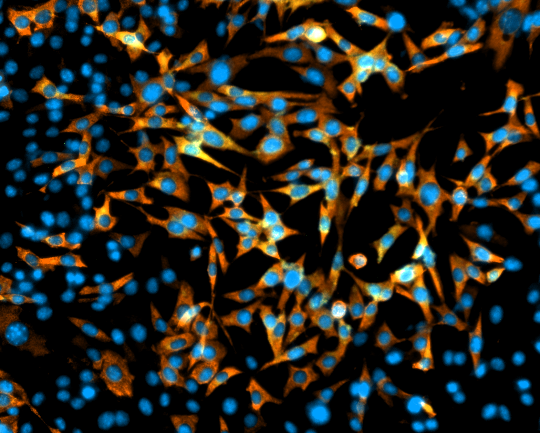 |
| **heparin 5 µg/ml 3** | 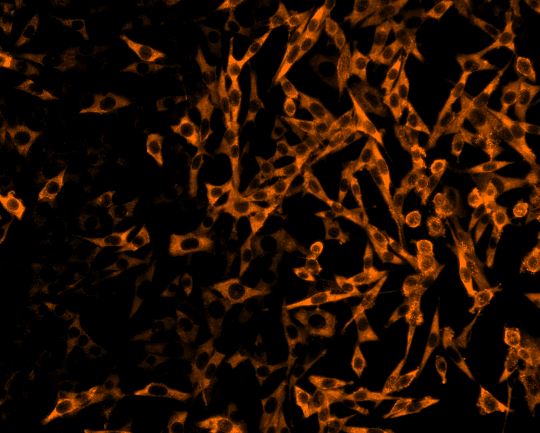 | 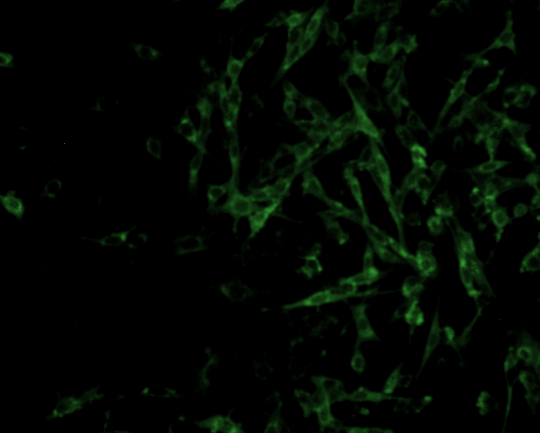 | 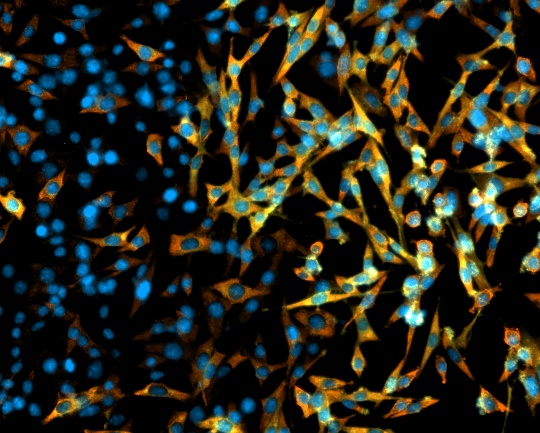 |
| **heparin 5 µg/ml 4** | 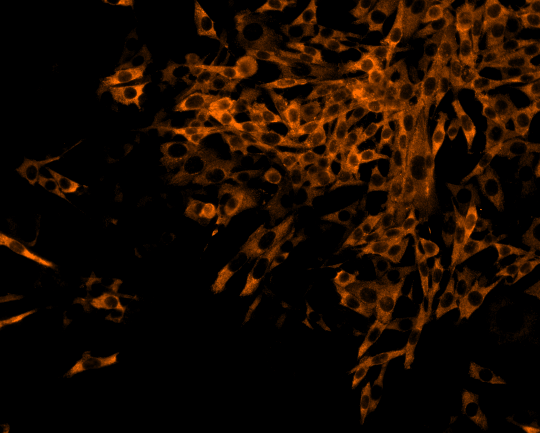 | 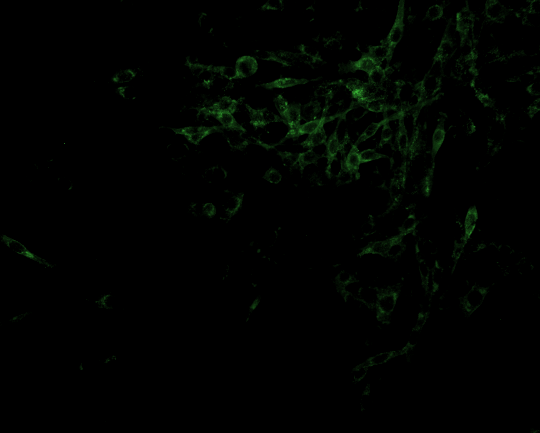 | 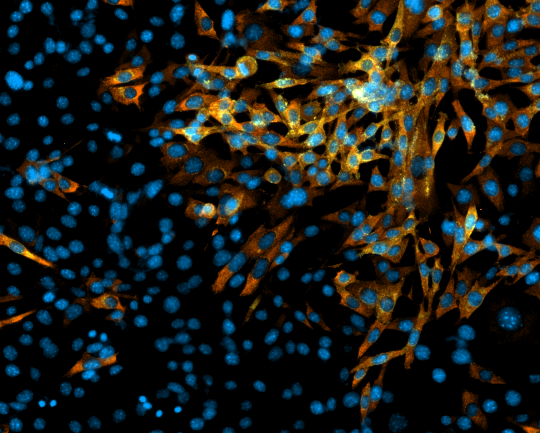 |
| **heparin 0.5 µg/ml 1** | 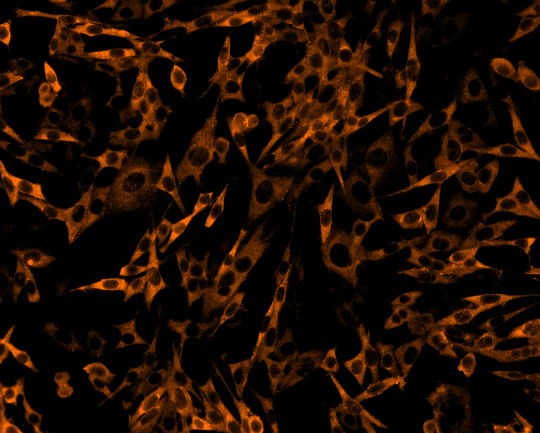 | 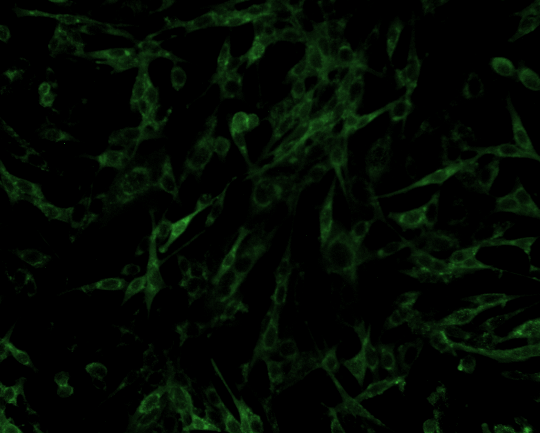 | 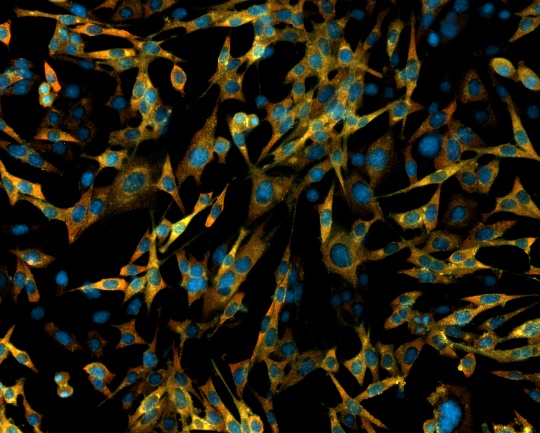 |
| **heparin 0.5 µg/ml 2** | 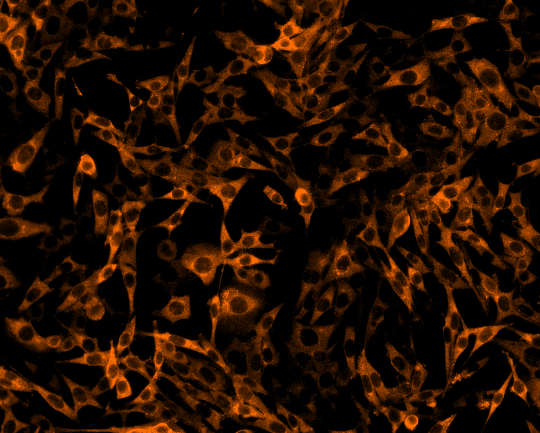 | 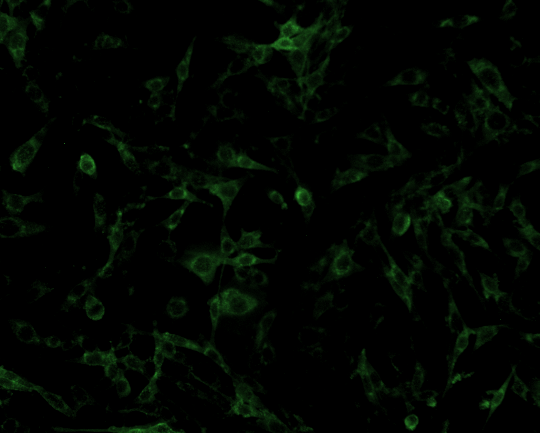 | 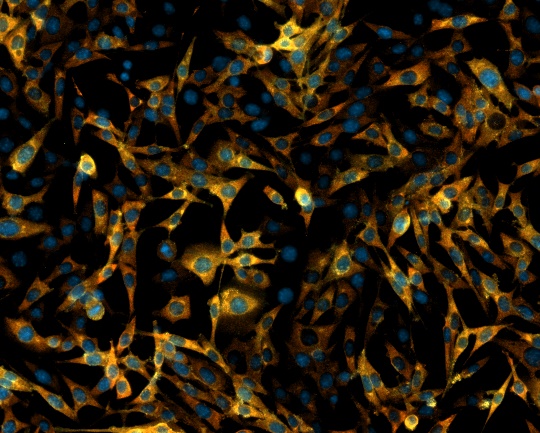 |
| **heparin 0.5 µg/ml 3** | 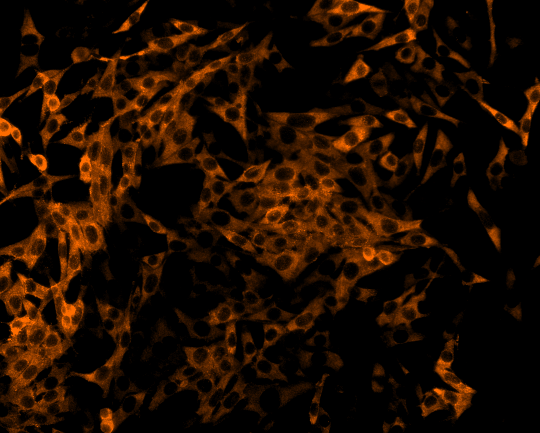 | 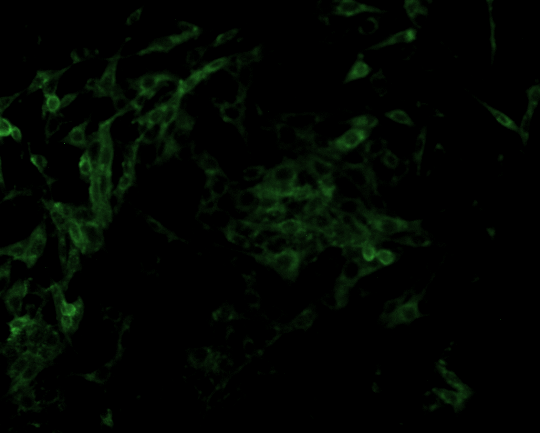 | 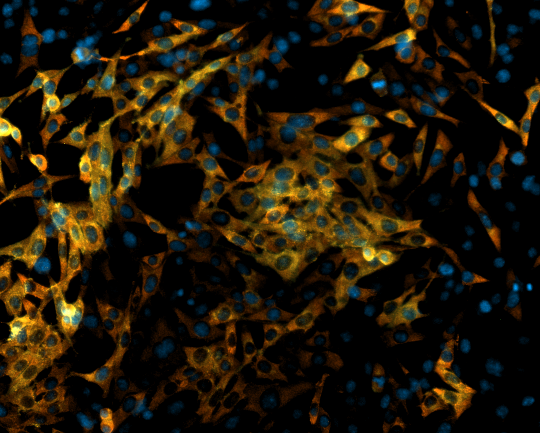 |
| **heparin 0.5 µg/ml 4** | 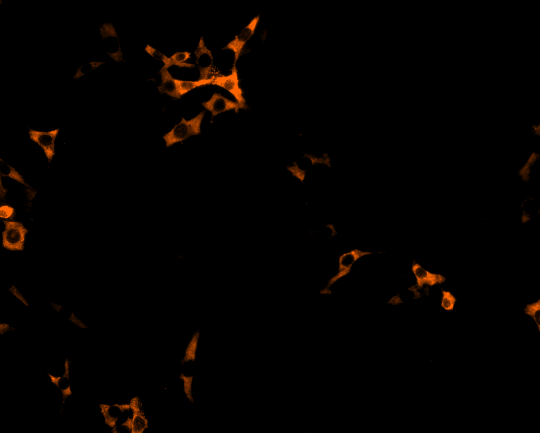 | 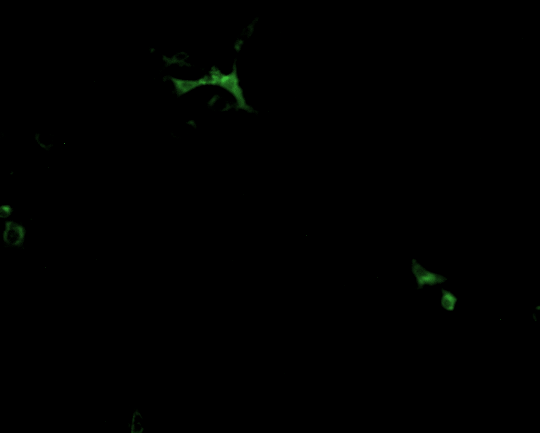 | 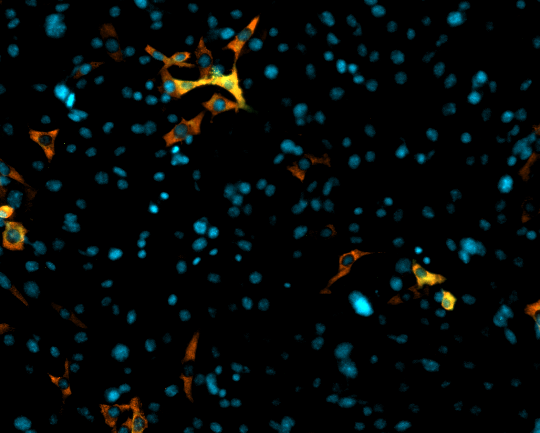 |
| **heparin 0.05 µg/ml 1** | 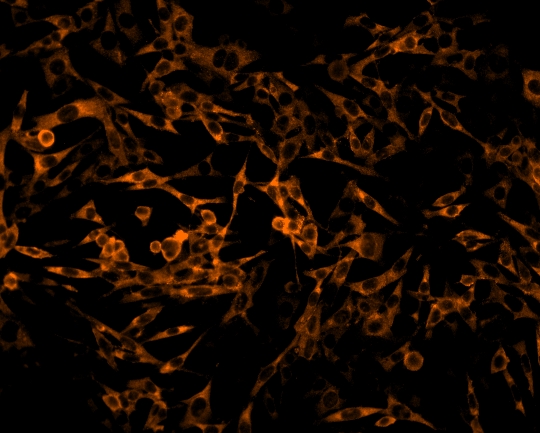 | 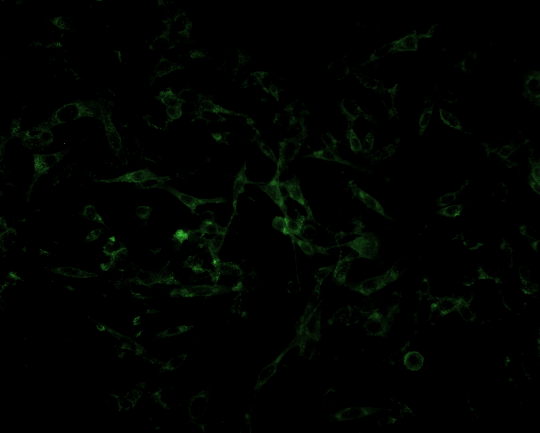 | 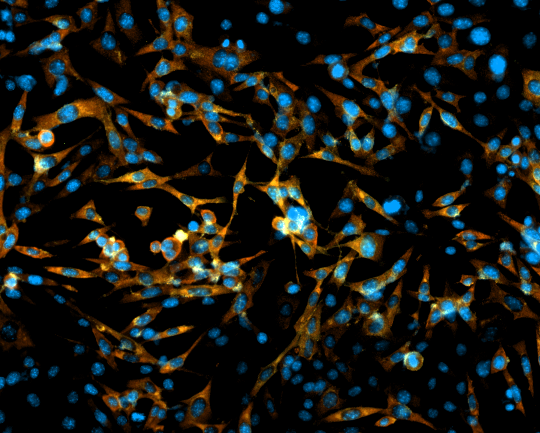 |
| **heparin 0.05 µg/ml 1** | 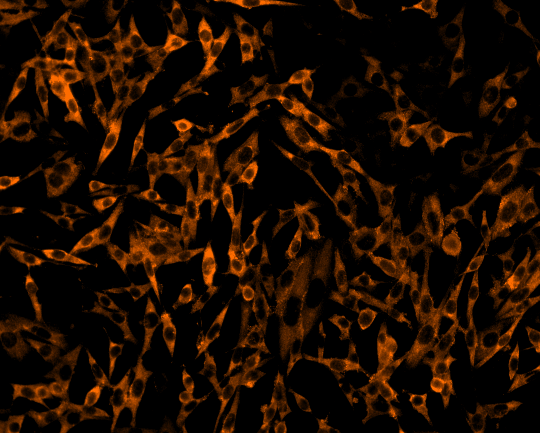 | 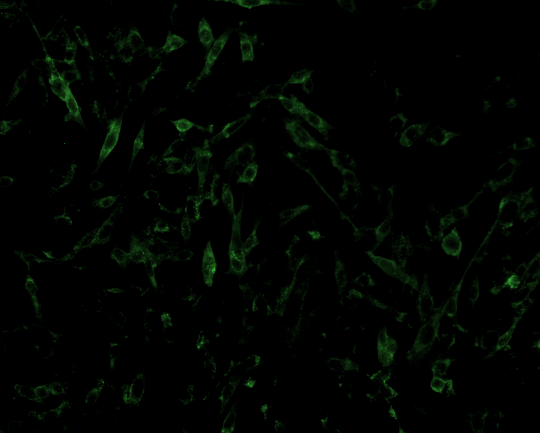 | 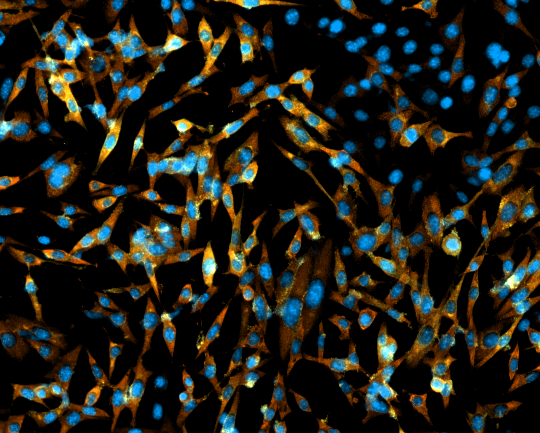 |
| **heparin 0.05 µg/ml 1** | 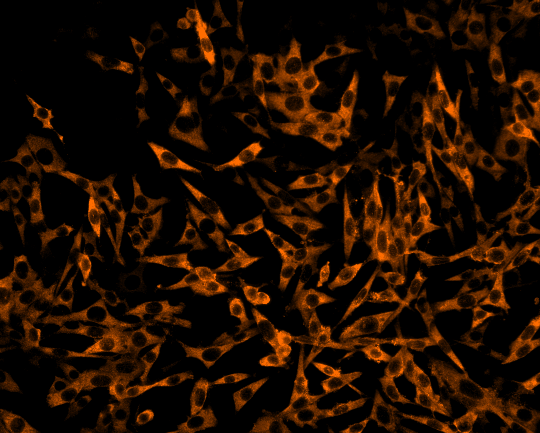 | 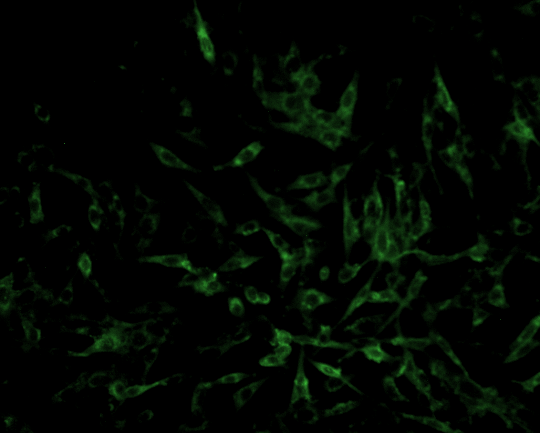 | 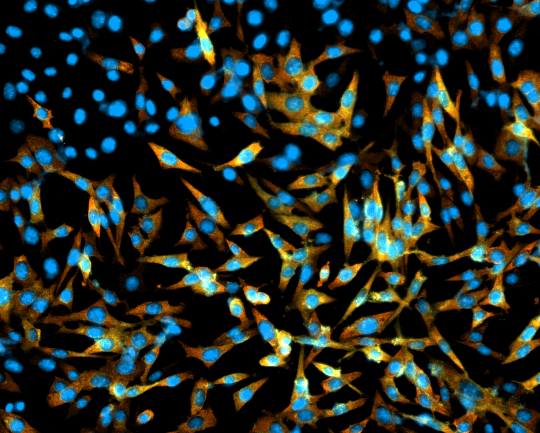 |
| **heparin 0.05 µg/ml 1** | 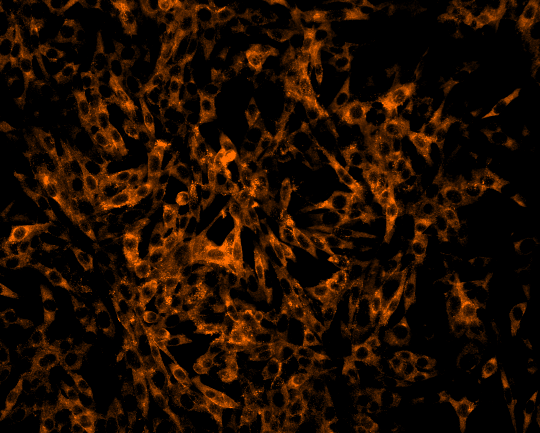 | 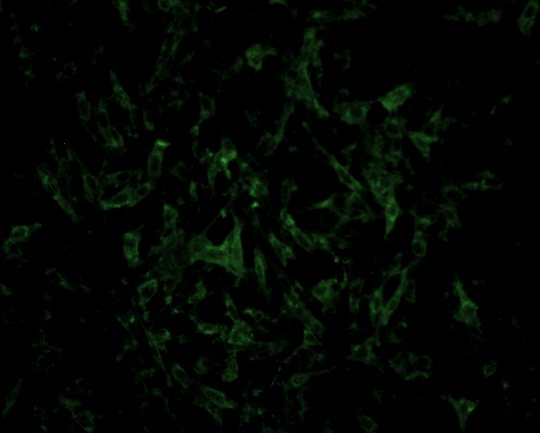 | 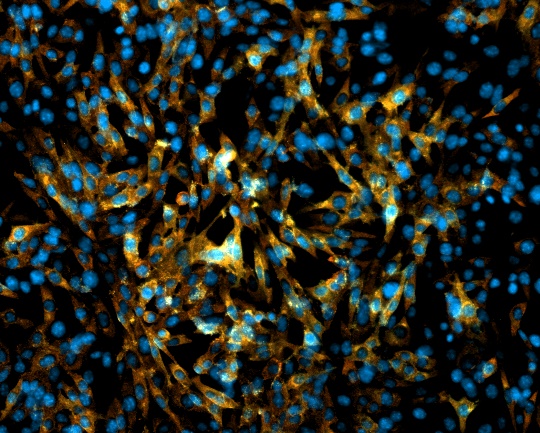 |
