# Supplementary figures and images for: Sulfated glycosaminoglycans inhibit LCMV entry and modulate antiviral immunity and pathology
Source: EMBO Mol Med. 2026 Feb 23;18(4):1235–64. doi: 10.1038/s44321-026-00387-8 (PMC13083911; doi:10.1038/s44321-026-00387-8)

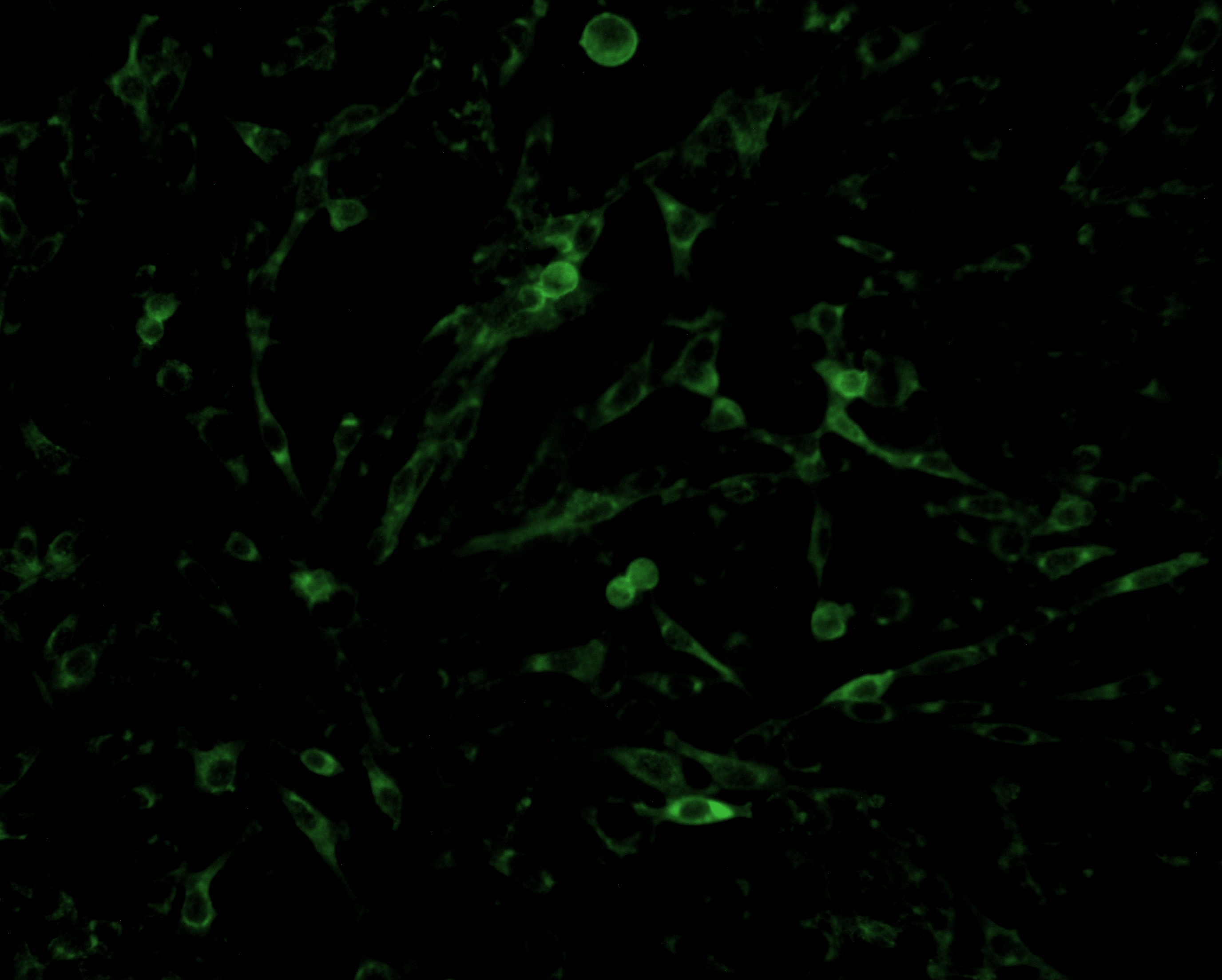

Supplement: Supplementary file 3 — Source data Fig. 1 [file 44321_2026_387_MOESM3_ESM.zip › Fig. 1/1F/Fig. 1F/PC_11-Image Export-05_c2x0-2752y0-2208_NP.tif]

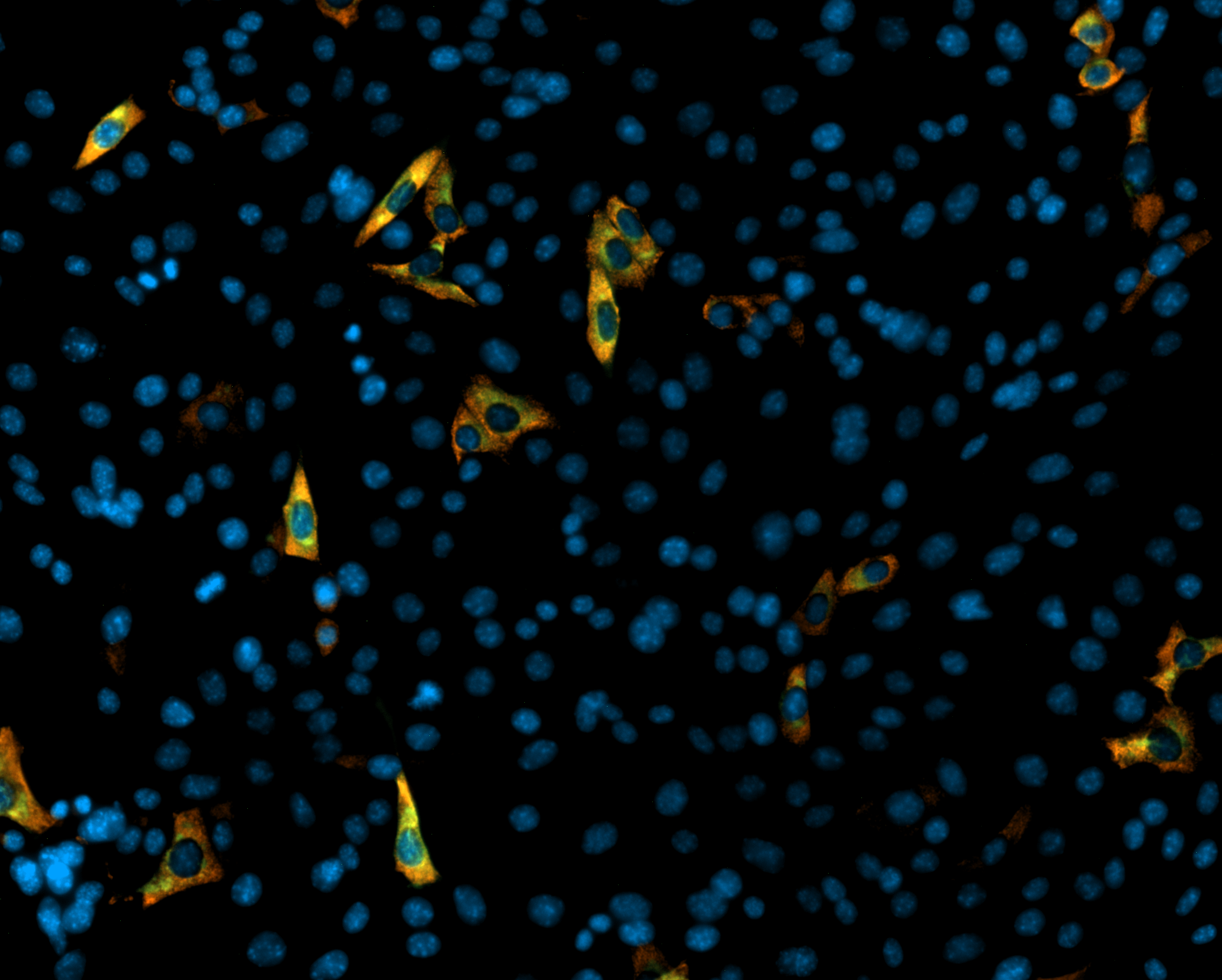

Supplement: Supplementary file 3 — Source data Fig. 1 [file 44321_2026_387_MOESM3_ESM.zip › Fig. 1/1F/Fig. 1F/H2_6-Image Export-22_c0-3x0-2752y0-2208_merge.tif]

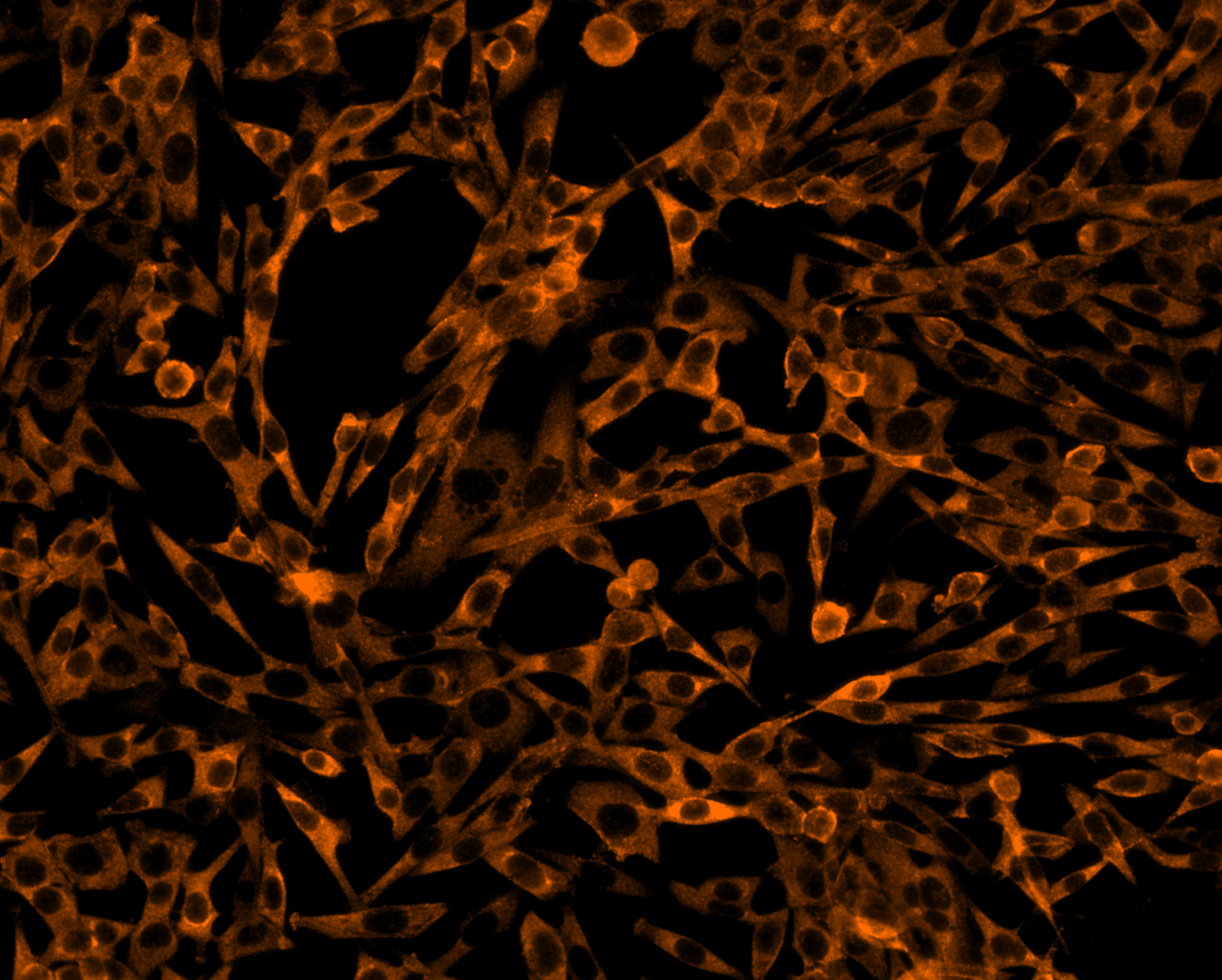

Supplement: Supplementary file 3 — Source data Fig. 1 [file 44321_2026_387_MOESM3_ESM.zip › Fig. 1/1F/Fig. 1F/PC_11-Image Export-05_c1x0-2752y0-2208_GP.tif]

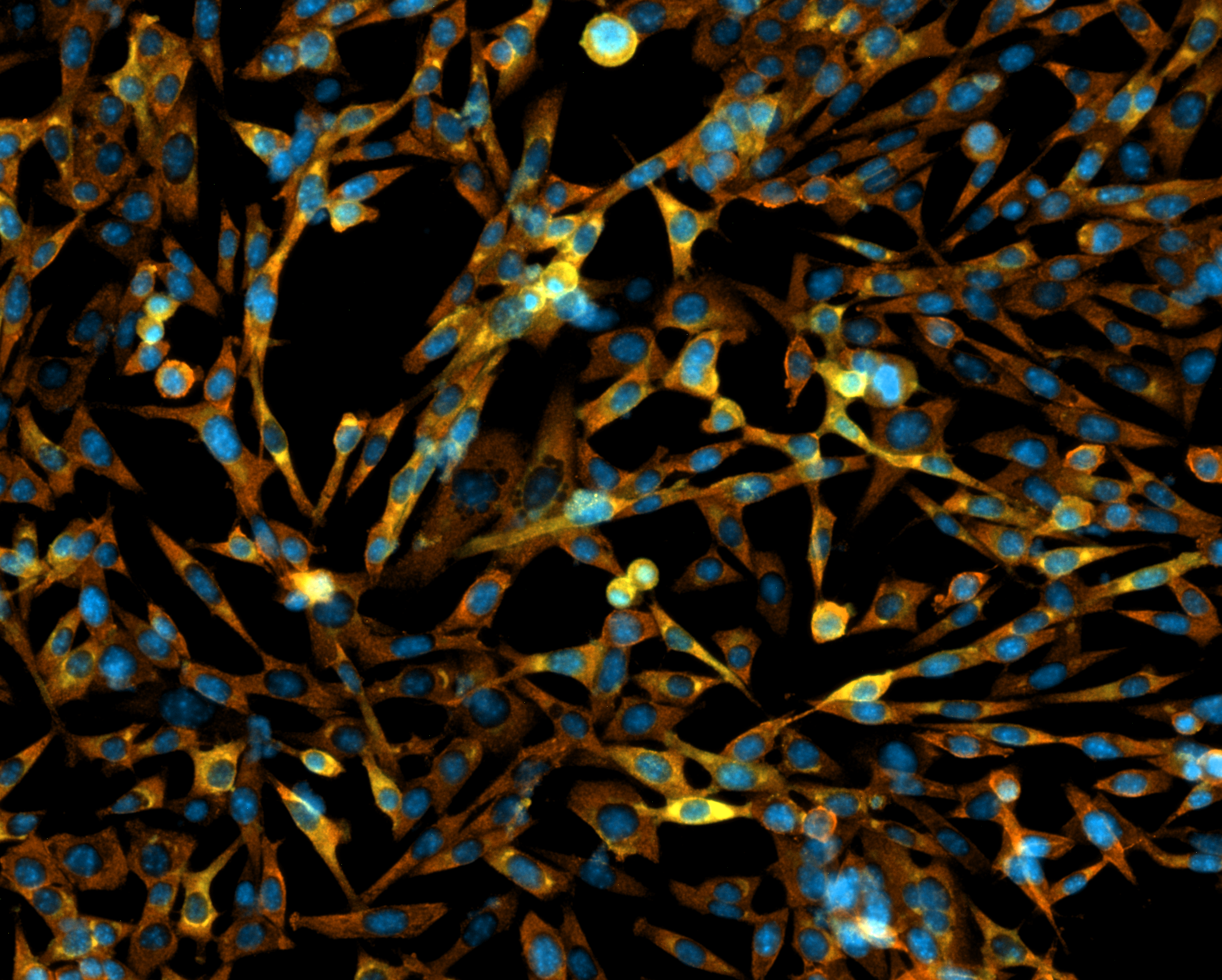

Supplement: Supplementary file 3 — Source data Fig. 1 [file 44321_2026_387_MOESM3_ESM.zip › Fig. 1/1F/Fig. 1F/PC_11-Image Export-05_c0-3x0-2752y0-2208_merge.tif]

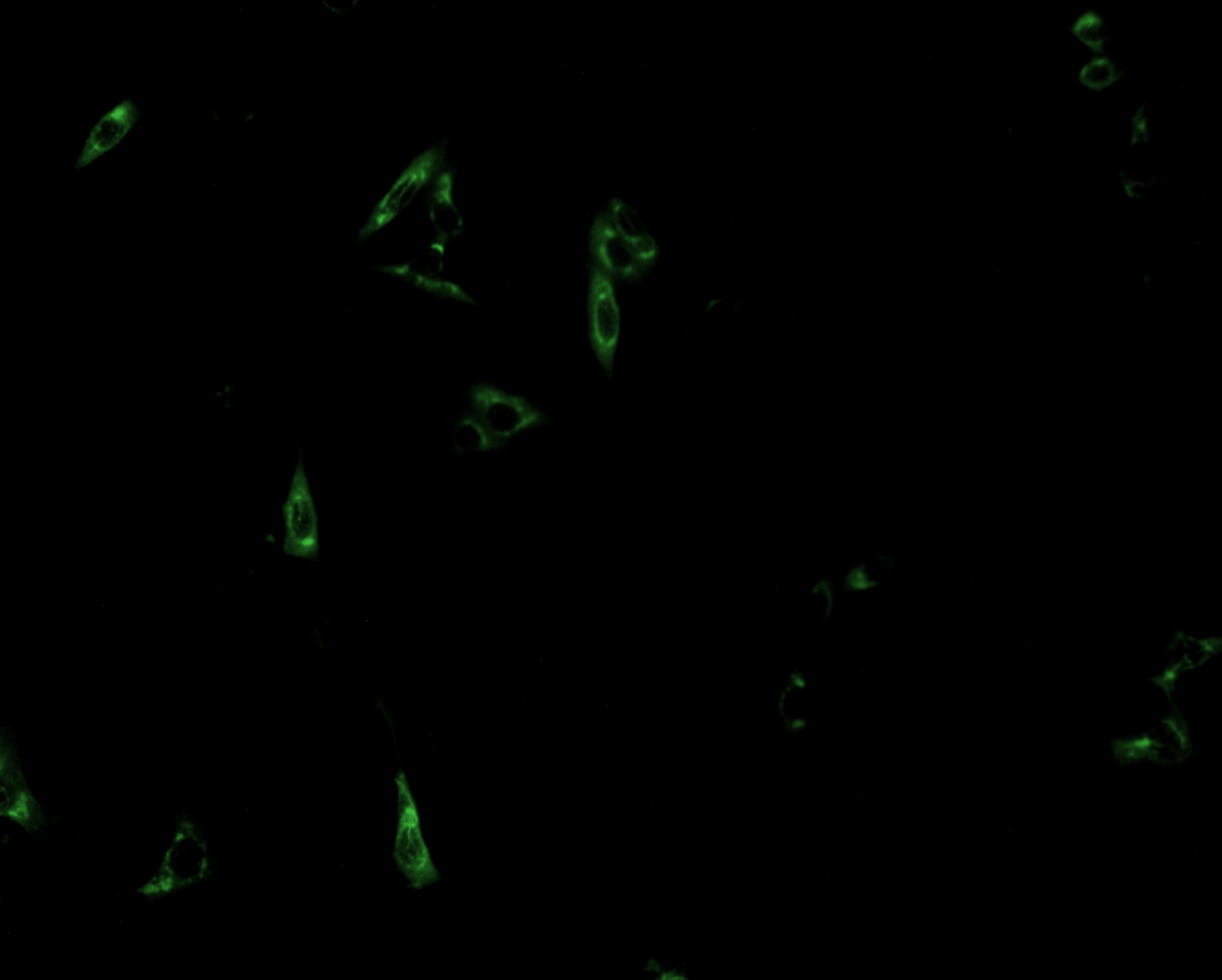

Supplement: Supplementary file 3 — Source data Fig. 1 [file 44321_2026_387_MOESM3_ESM.zip › Fig. 1/1F/Fig. 1F/H2_6-Image Export-22_c2x0-2752y0-2208_NP.tif]

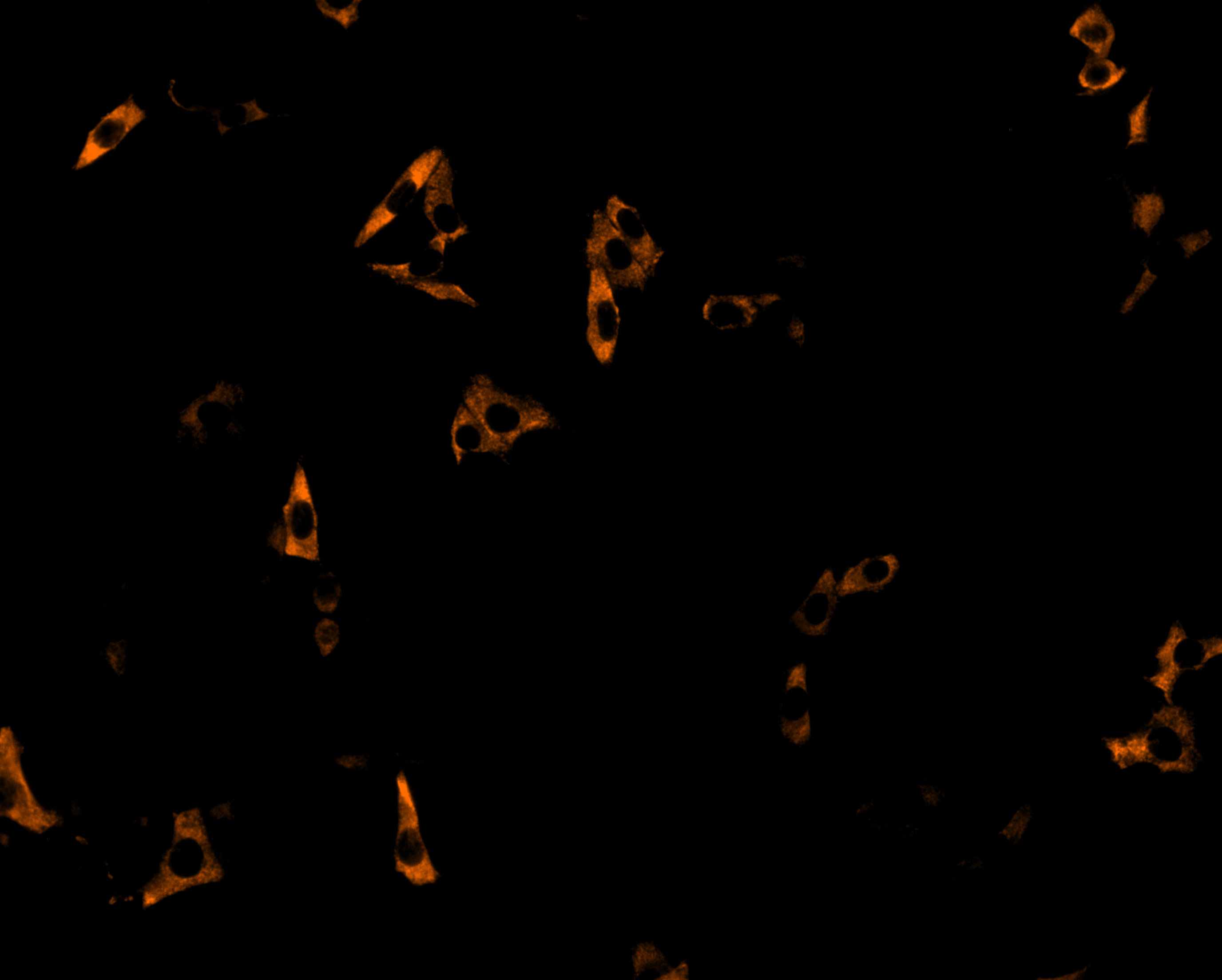

Supplement: Supplementary file 3 — Source data Fig. 1 [file 44321_2026_387_MOESM3_ESM.zip › Fig. 1/1F/Fig. 1F/H2_6-Image Export-22_c1x0-2752y0-2208_GP.tif]

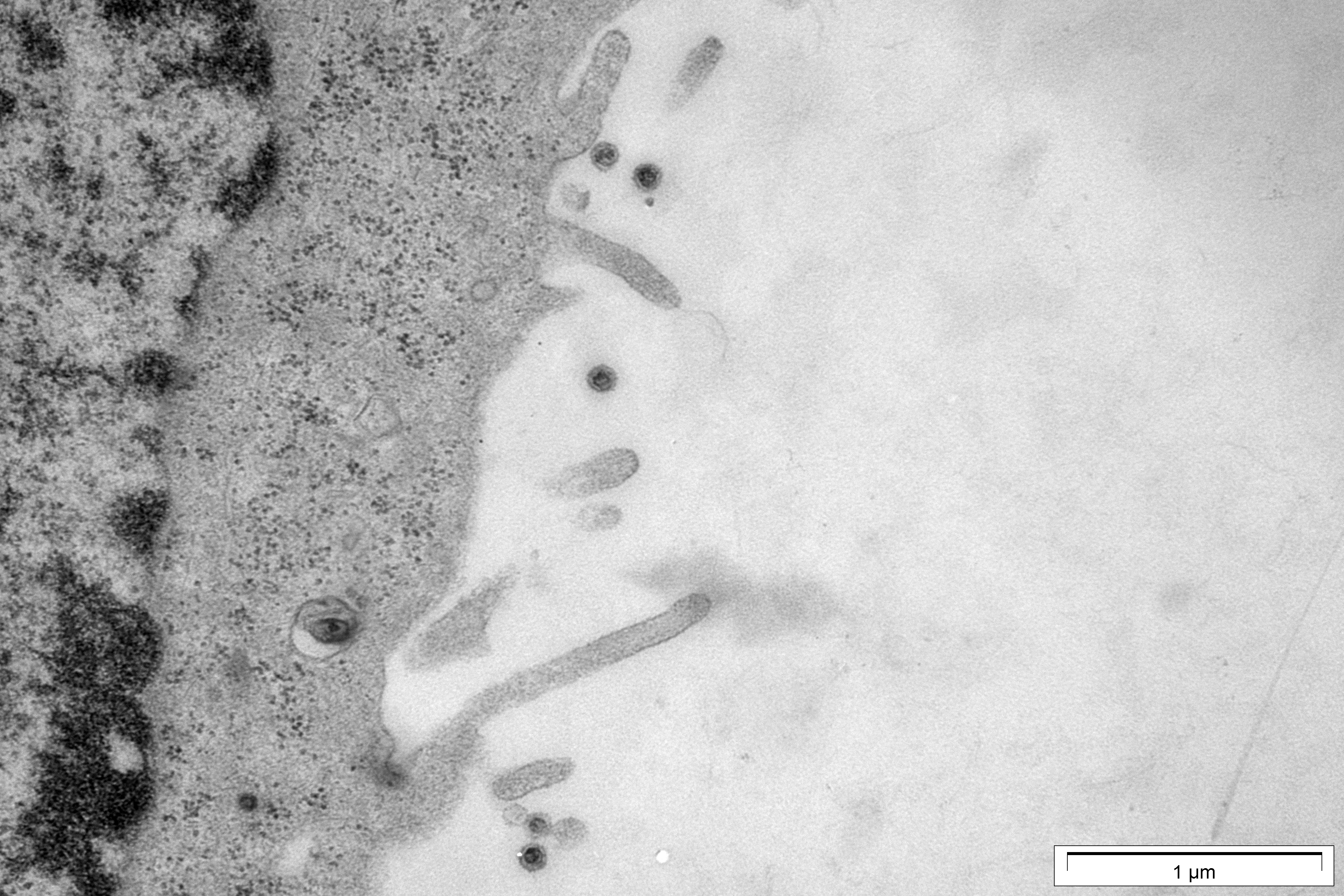

Supplement: Supplementary file 5 — Source data Fig. 3 [file 44321_2026_387_MOESM5_ESM.zip › Fig. 3/Fig. 3A-B/Fig. 3B/22-10-28-DexSuf-1min_30k_08.tif]

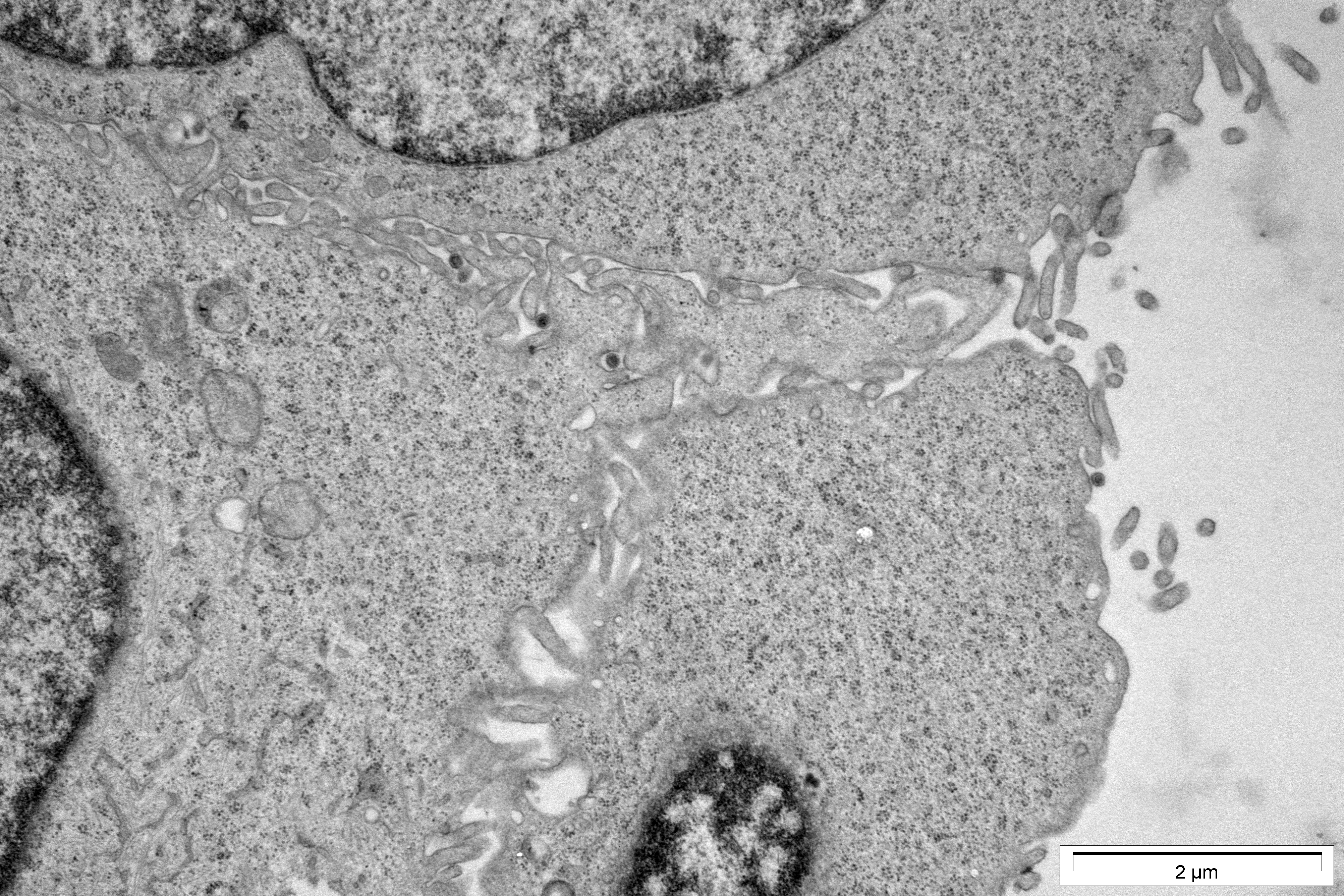

Supplement: Supplementary file 5 — Source data Fig. 3 [file 44321_2026_387_MOESM5_ESM.zip › Fig. 3/Fig. 3A-B/Fig. 3A/22-09-16-Dextran-1min_15k_01.tif]

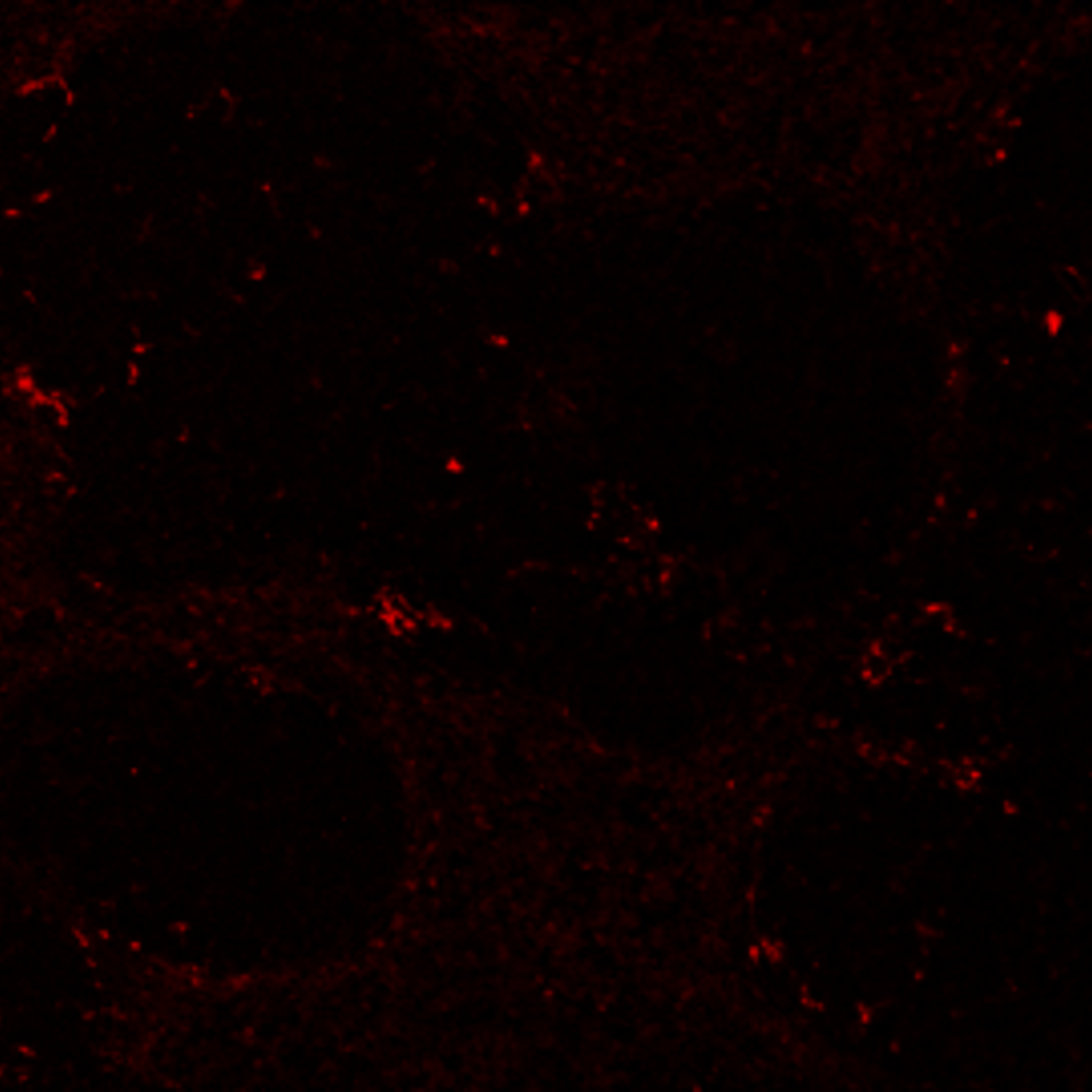

Supplement: Supplementary file 7 — Source data Fig. 5 [file 44321_2026_387_MOESM7_ESM.zip › Fig. 5/Fig. 5C/Fig.5C ds lcmv np.tif]

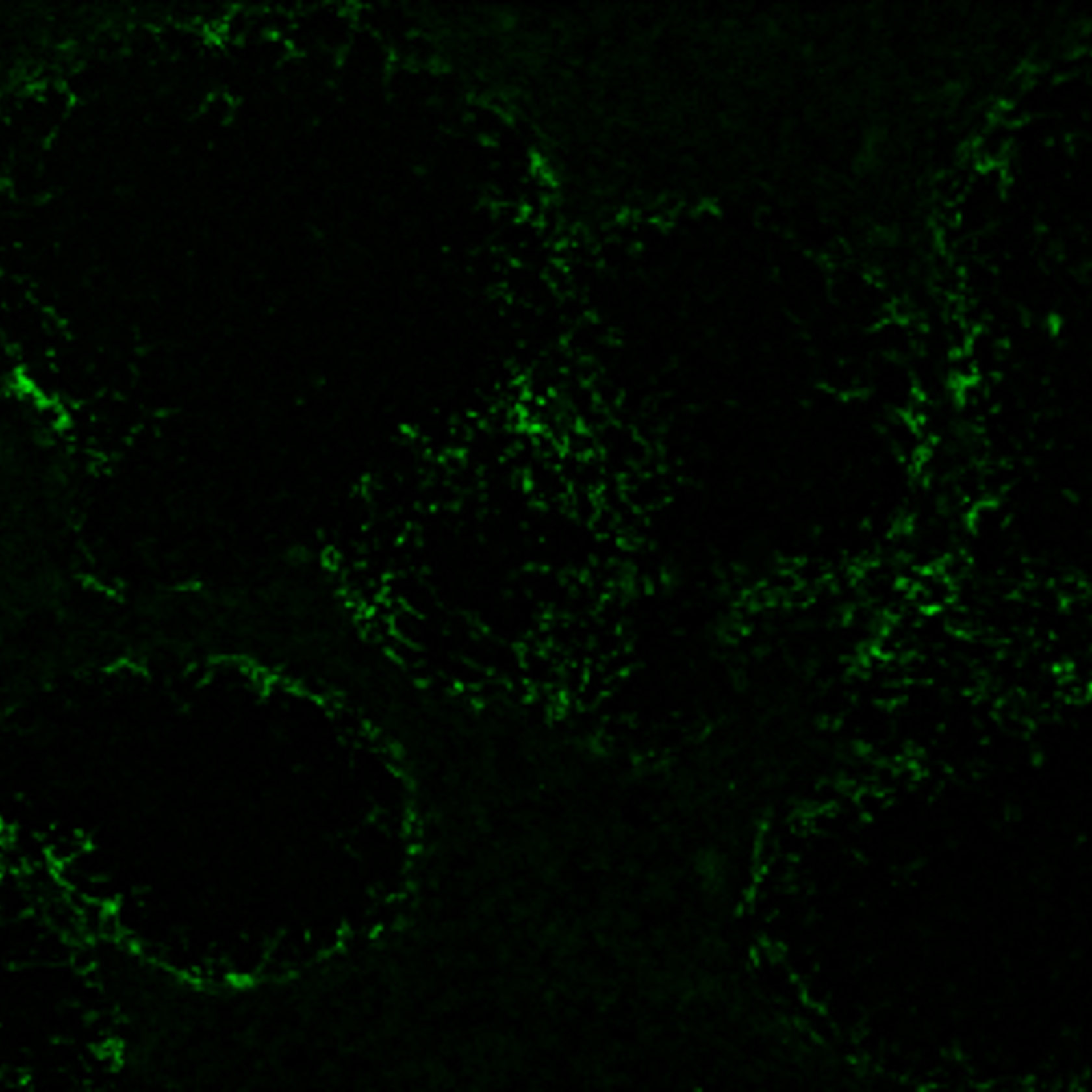

Supplement: Supplementary file 7 — Source data Fig. 5 [file 44321_2026_387_MOESM7_ESM.zip › Fig. 5/Fig. 5C/Fig.5C ds cd169.tif]

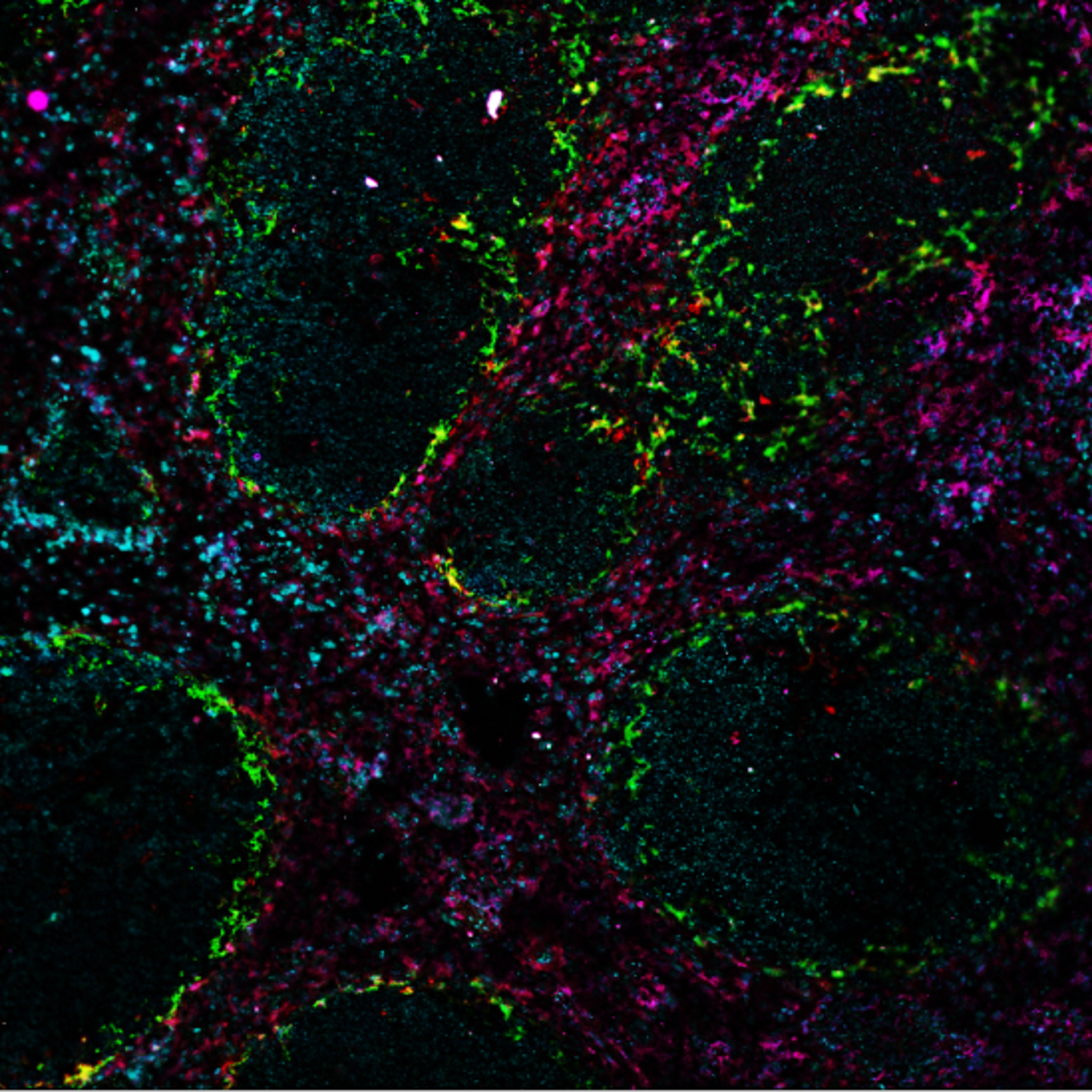

Supplement: Supplementary file 7 — Source data Fig. 5 [file 44321_2026_387_MOESM7_ESM.zip › Fig. 5/Fig. 5C/Fig.5C vehicle merged.tif]

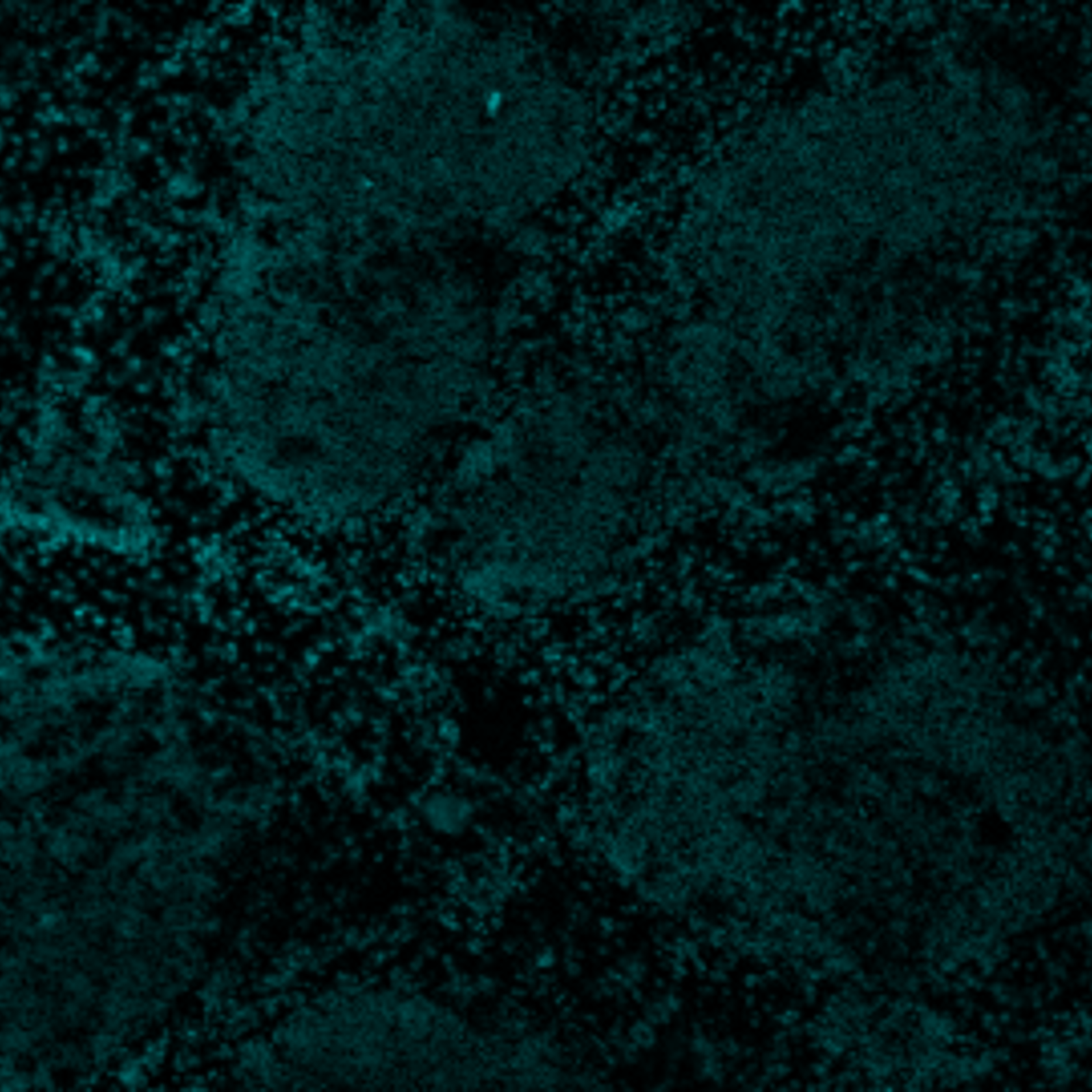

Supplement: Supplementary file 7 — Source data Fig. 5 [file 44321_2026_387_MOESM7_ESM.zip › Fig. 5/Fig. 5C/Fig.5C vehicle DAPI.tif]

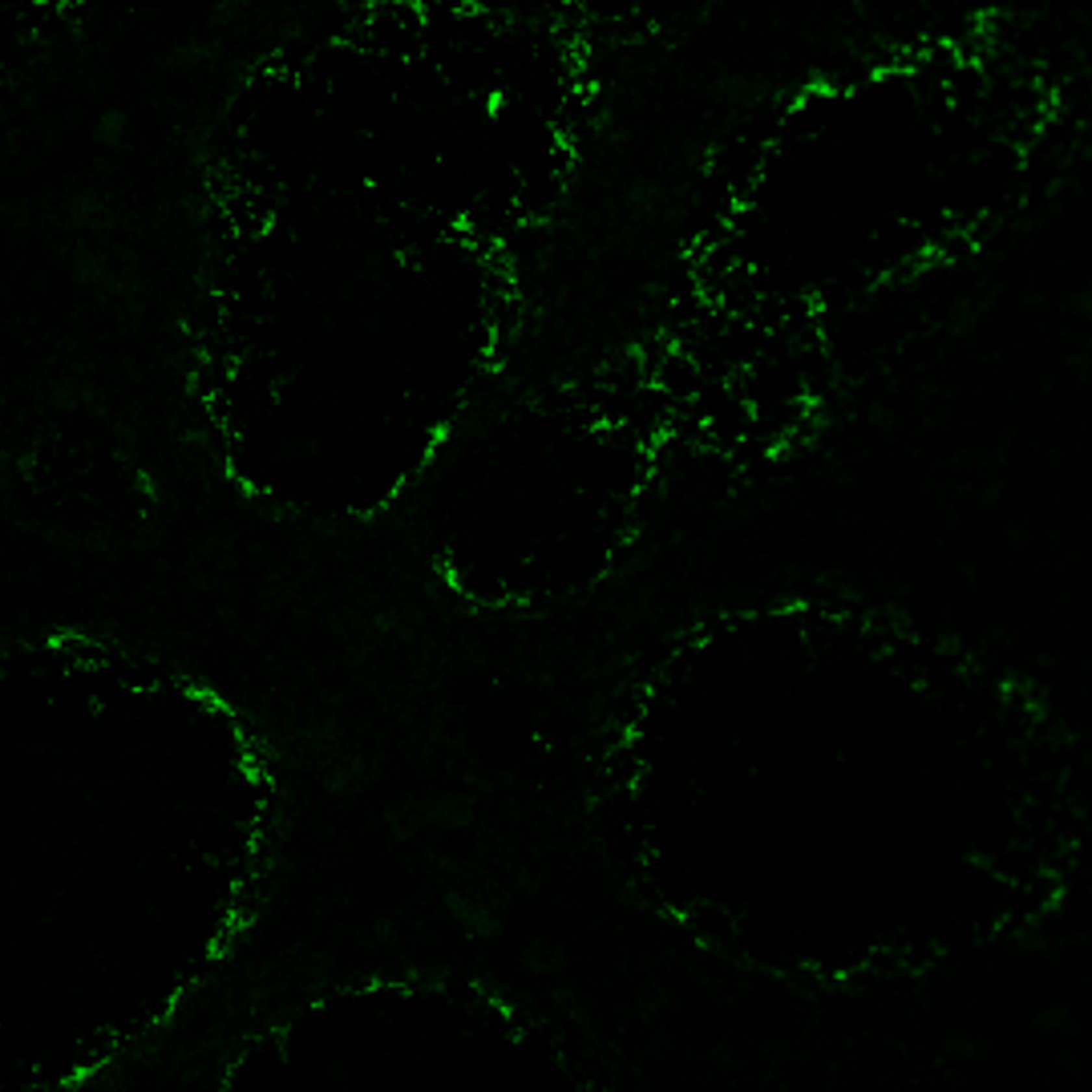

Supplement: Supplementary file 7 — Source data Fig. 5 [file 44321_2026_387_MOESM7_ESM.zip › Fig. 5/Fig. 5C/Fig.5C vehicle cd169.tif]

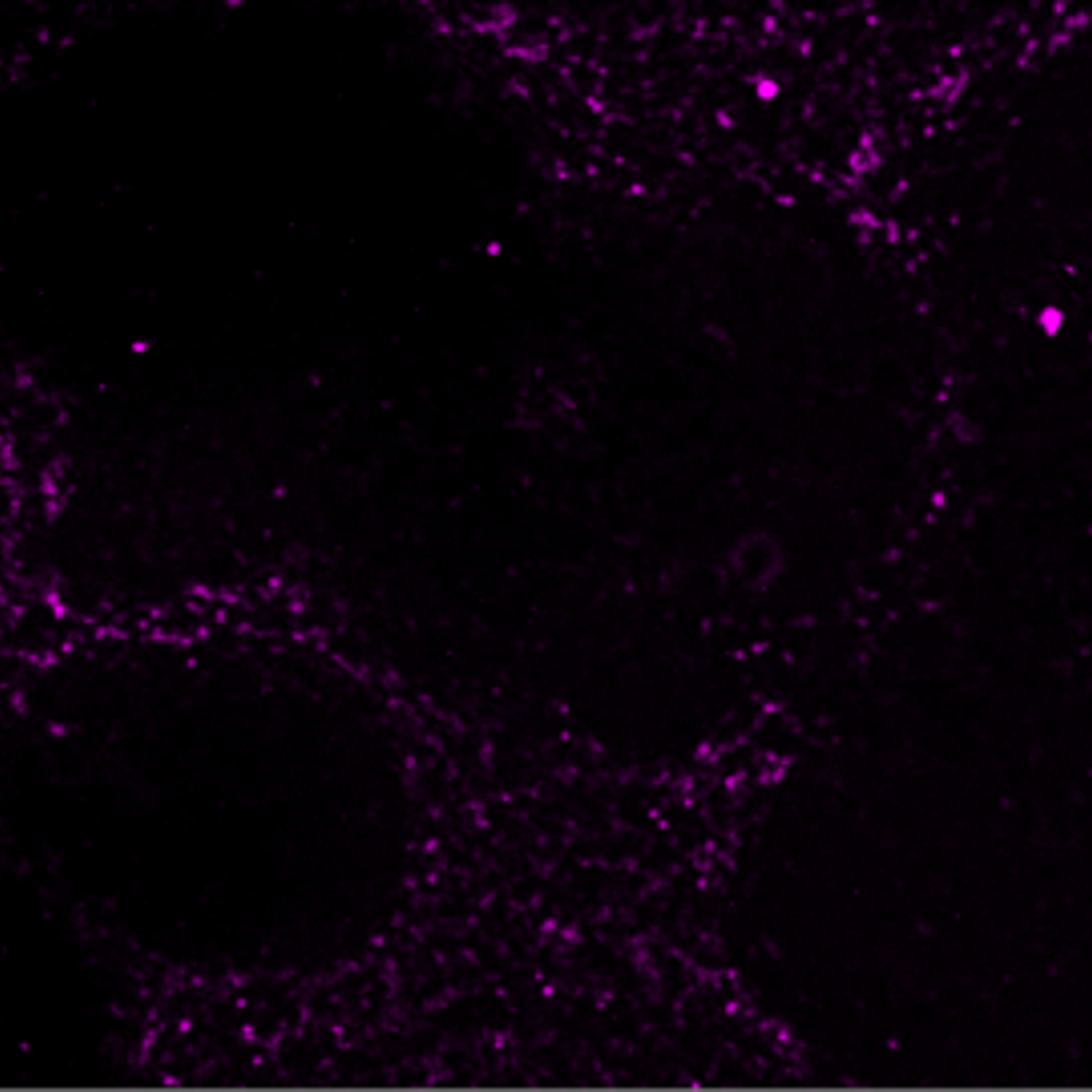

Supplement: Supplementary file 7 — Source data Fig. 5 [file 44321_2026_387_MOESM7_ESM.zip › Fig. 5/Fig. 5C/Fig.5C ds f480.tif]

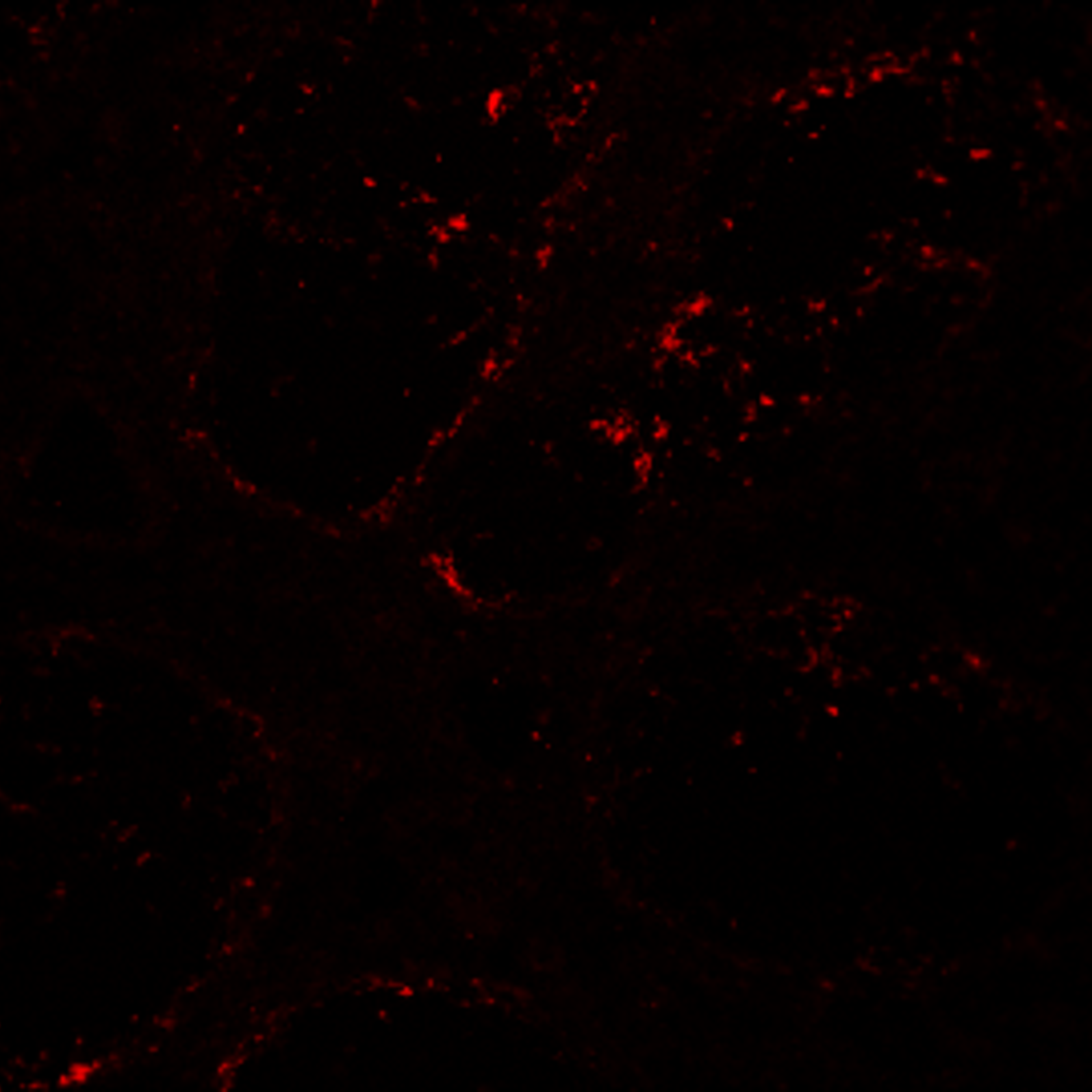

Supplement: Supplementary file 7 — Source data Fig. 5 [file 44321_2026_387_MOESM7_ESM.zip › Fig. 5/Fig. 5C/Fig.5C vehicle lcmv np.tif]

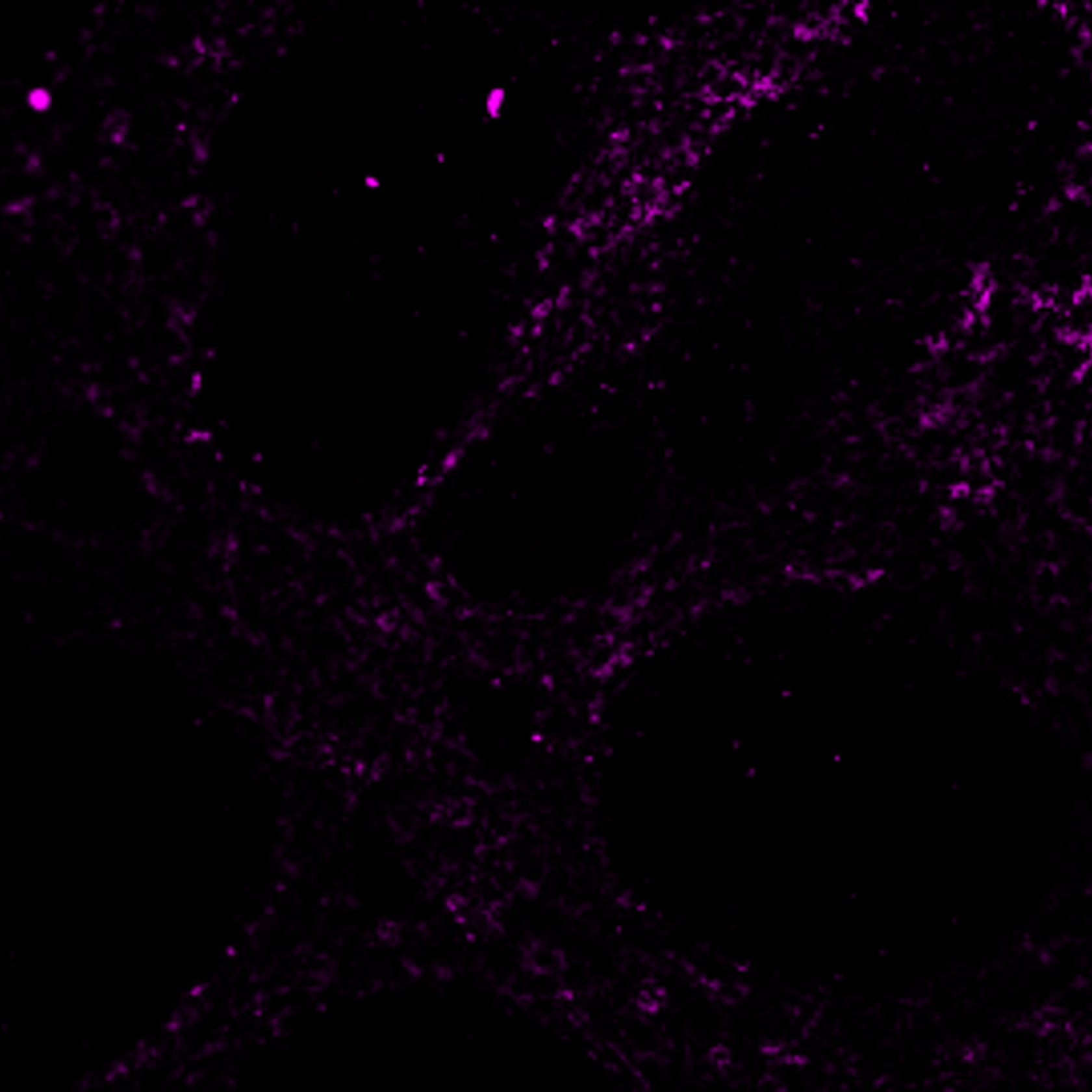

Supplement: Supplementary file 7 — Source data Fig. 5 [file 44321_2026_387_MOESM7_ESM.zip › Fig. 5/Fig. 5C/Fig.5C vehicle f480.tif]

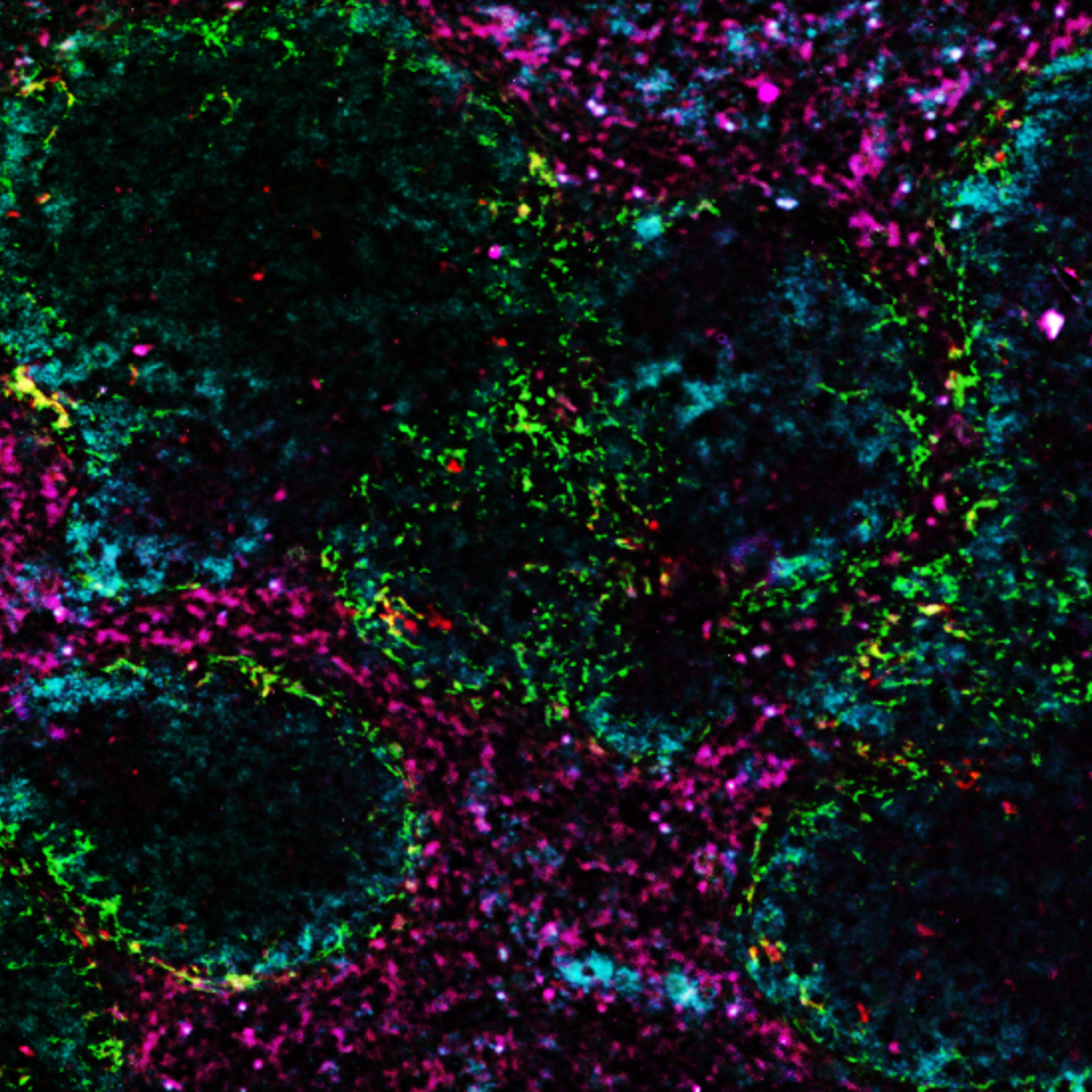

Supplement: Supplementary file 7 — Source data Fig. 5 [file 44321_2026_387_MOESM7_ESM.zip › Fig. 5/Fig. 5C/Fig.5C ds merged.tif]

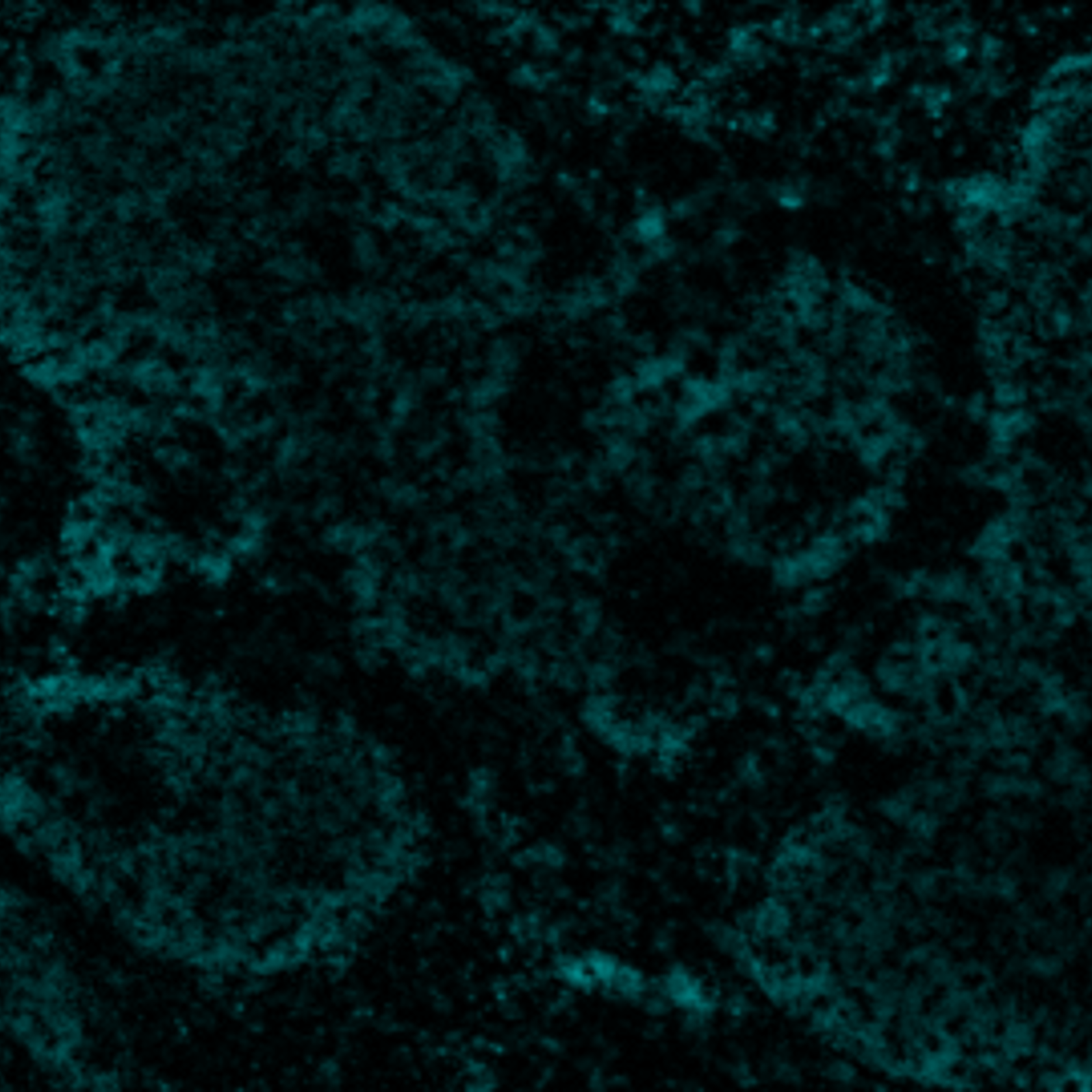

Supplement: Supplementary file 7 — Source data Fig. 5 [file 44321_2026_387_MOESM7_ESM.zip › Fig. 5/Fig. 5C/Fig.5C ds DAPI.tif]

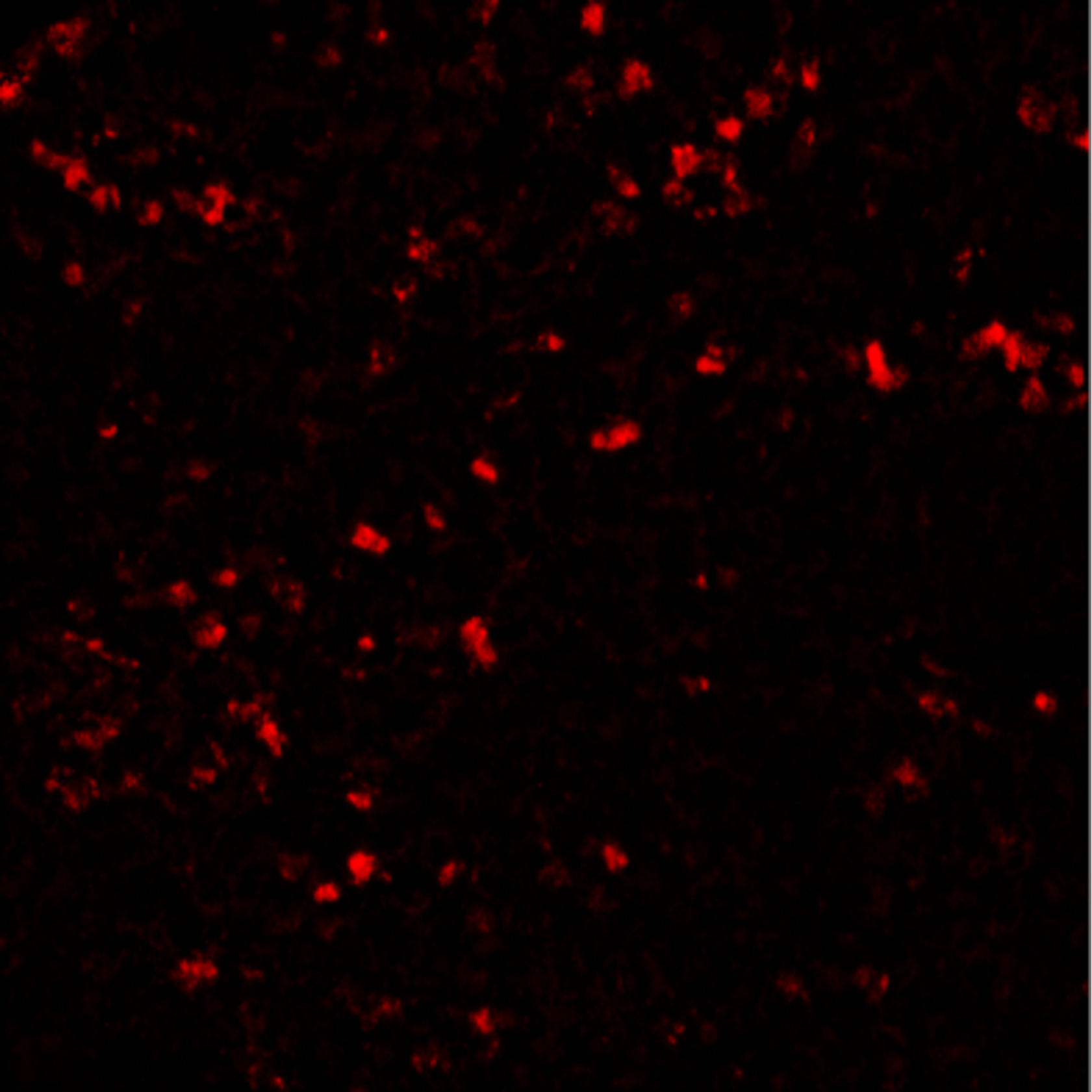

Supplement: Supplementary file 9 — Source data Fig. 7 [file 44321_2026_387_MOESM9_ESM.zip › Fig. 7/Fig. 7G/Fig.7G vehicle lcmv np.tif]

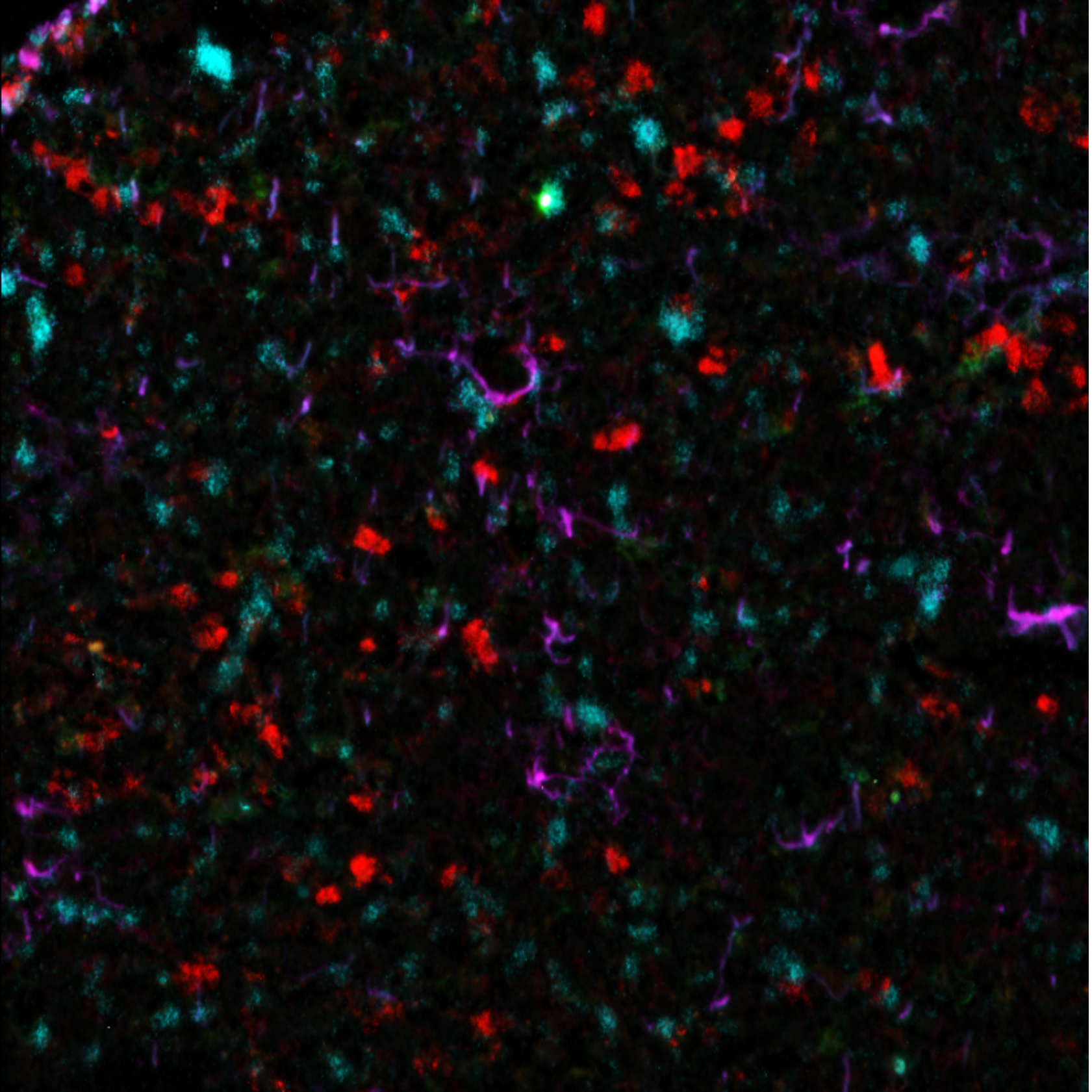

Supplement: Supplementary file 9 — Source data Fig. 7 [file 44321_2026_387_MOESM9_ESM.zip › Fig. 7/Fig. 7G/Fig.7G vehicle merged.tif]

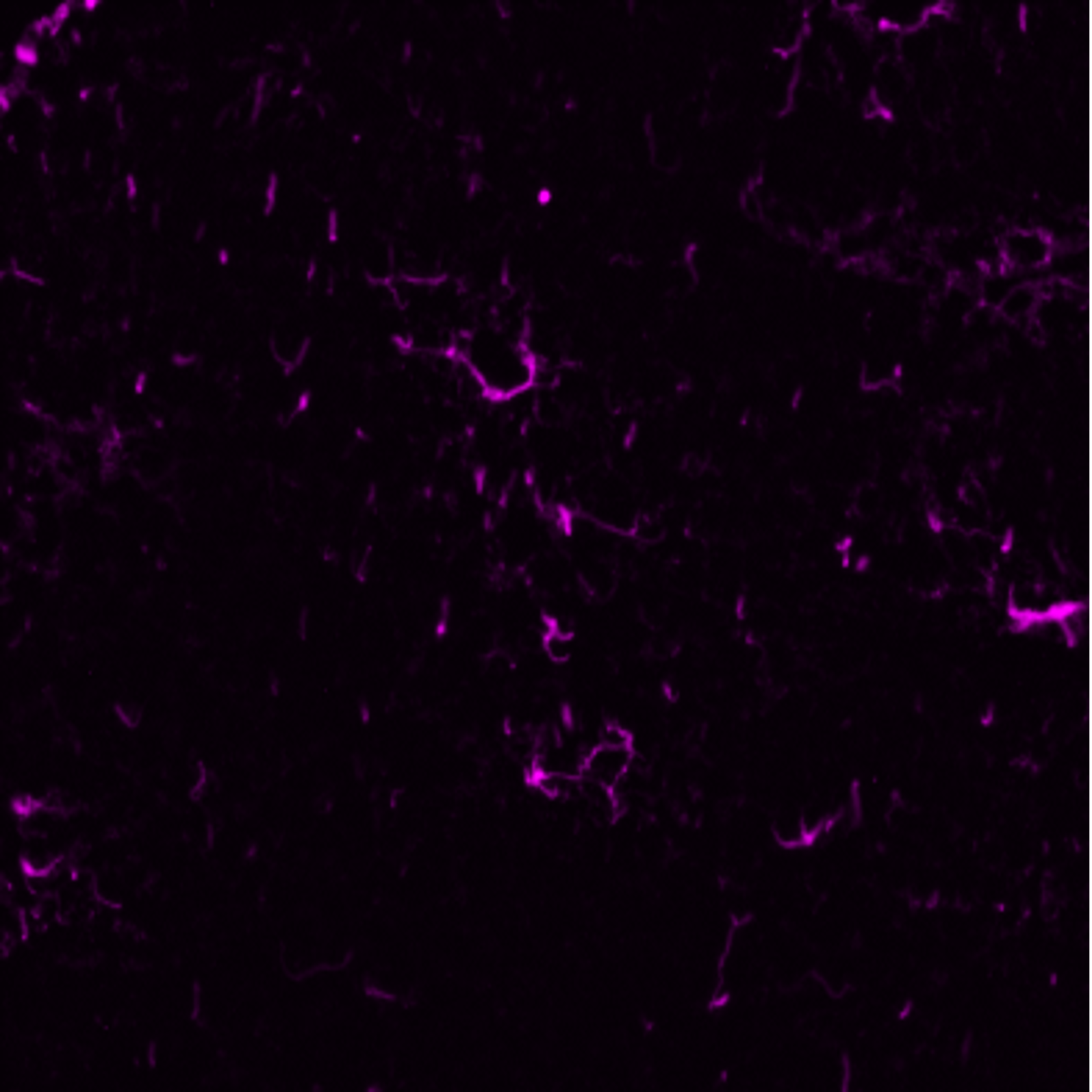

Supplement: Supplementary file 9 — Source data Fig. 7 [file 44321_2026_387_MOESM9_ESM.zip › Fig. 7/Fig. 7G/Fig.7G vehicle col1.tif]

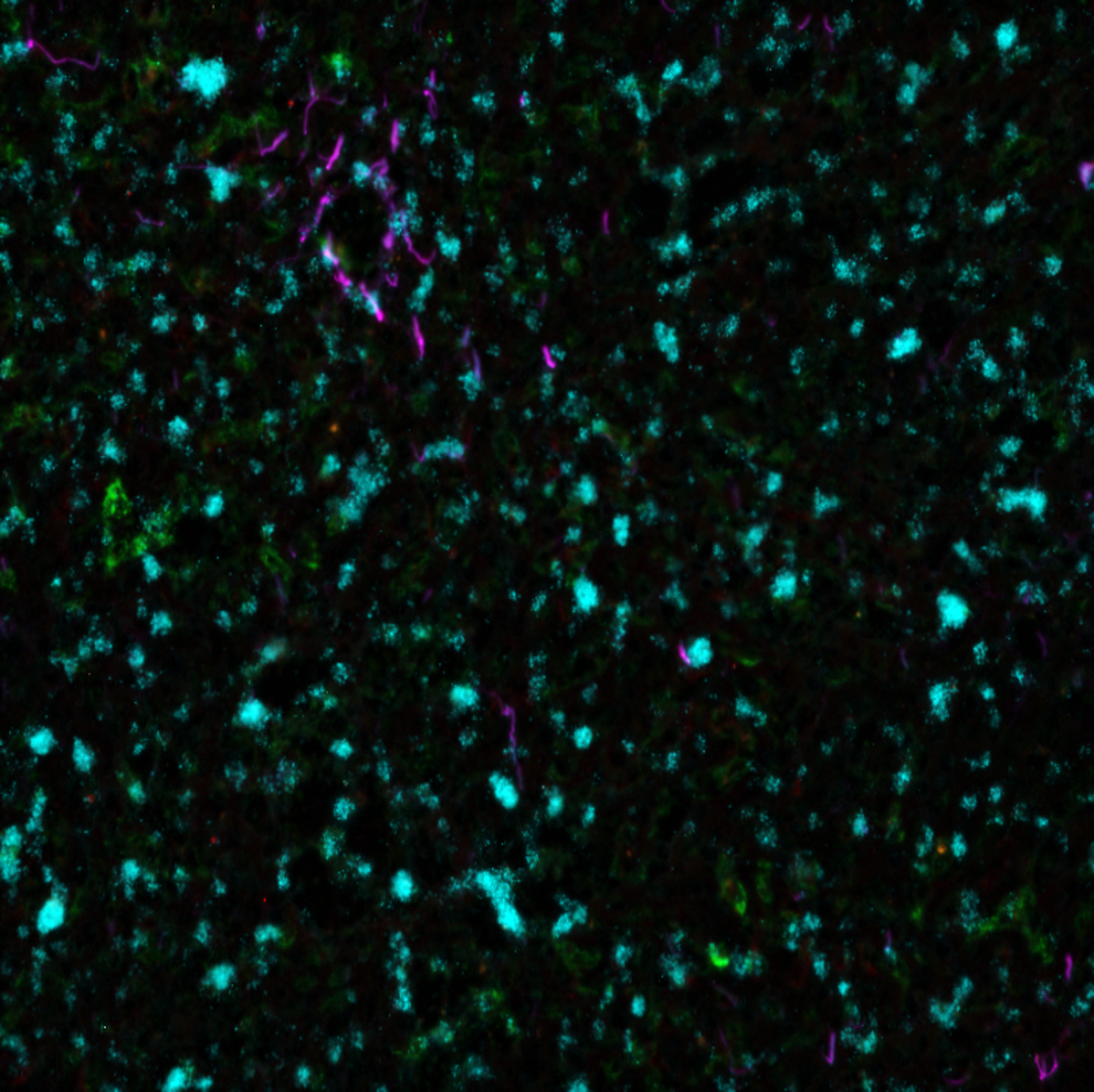

Supplement: Supplementary file 9 — Source data Fig. 7 [file 44321_2026_387_MOESM9_ESM.zip › Fig. 7/Fig. 7G/Fig.7G ds merged.tif]

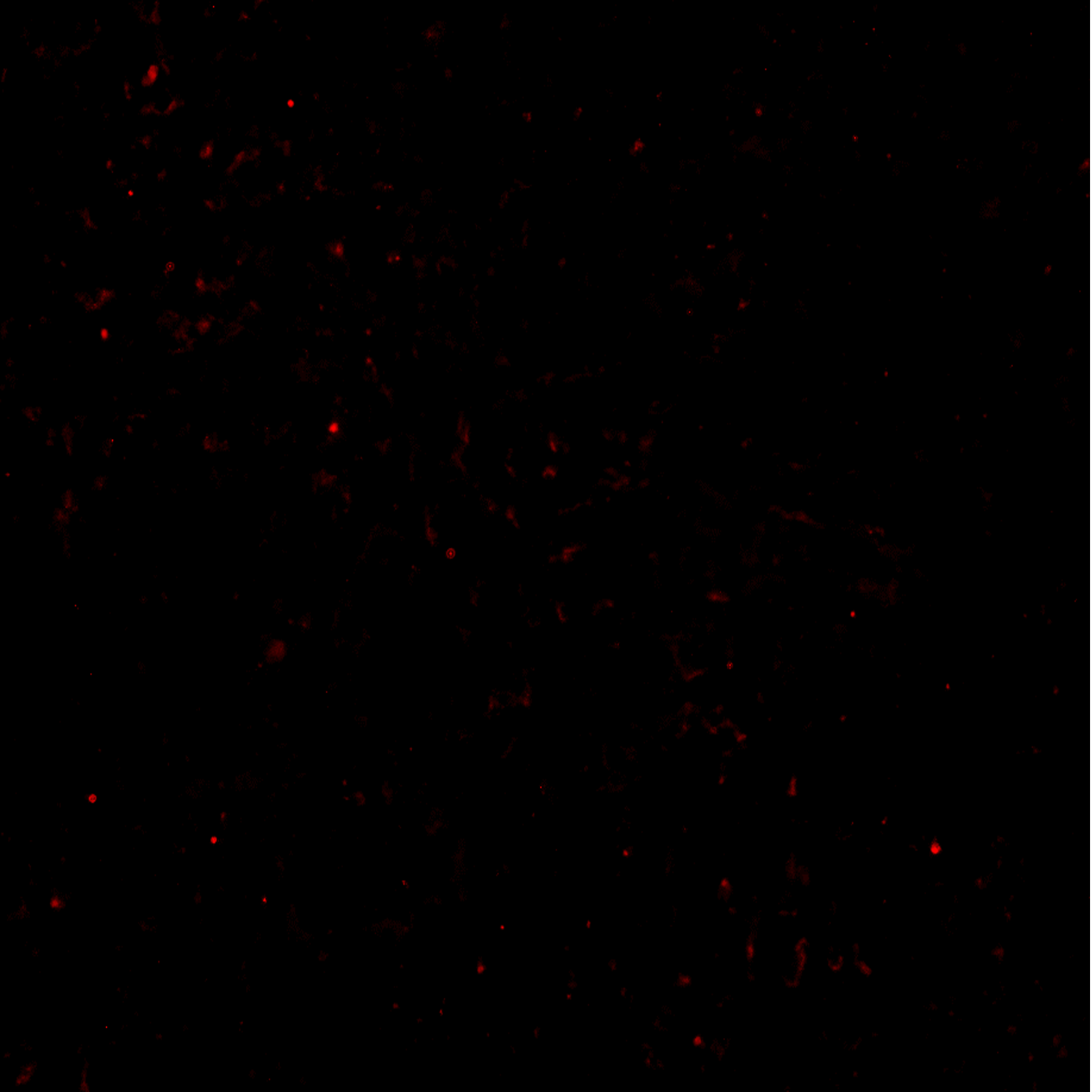

Supplement: Supplementary file 9 — Source data Fig. 7 [file 44321_2026_387_MOESM9_ESM.zip › Fig. 7/Fig. 7G/Fig.7G ds lcmv np.tif]

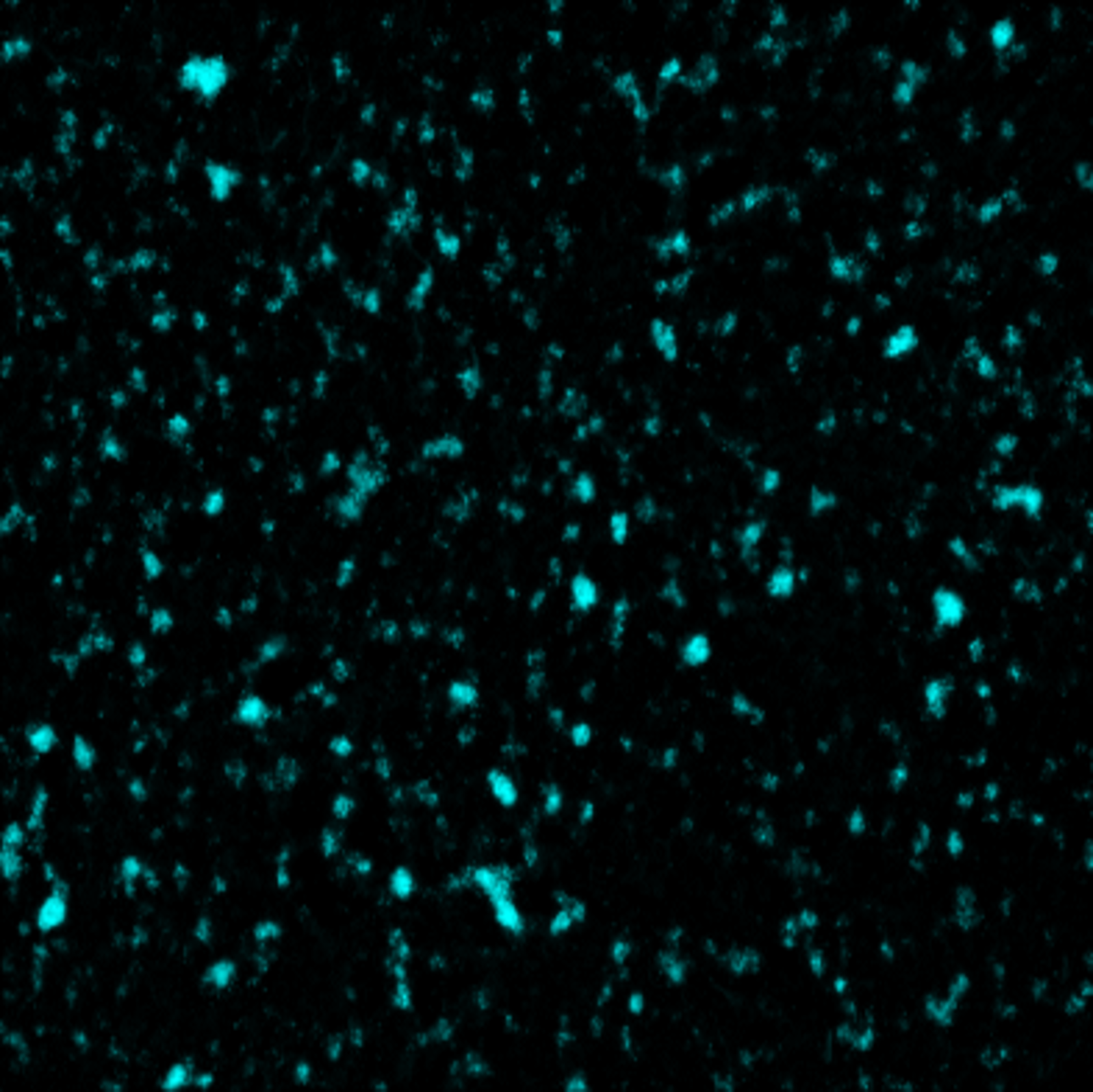

Supplement: Supplementary file 9 — Source data Fig. 7 [file 44321_2026_387_MOESM9_ESM.zip › Fig. 7/Fig. 7G/Fig.7G ds DAPI.tif]

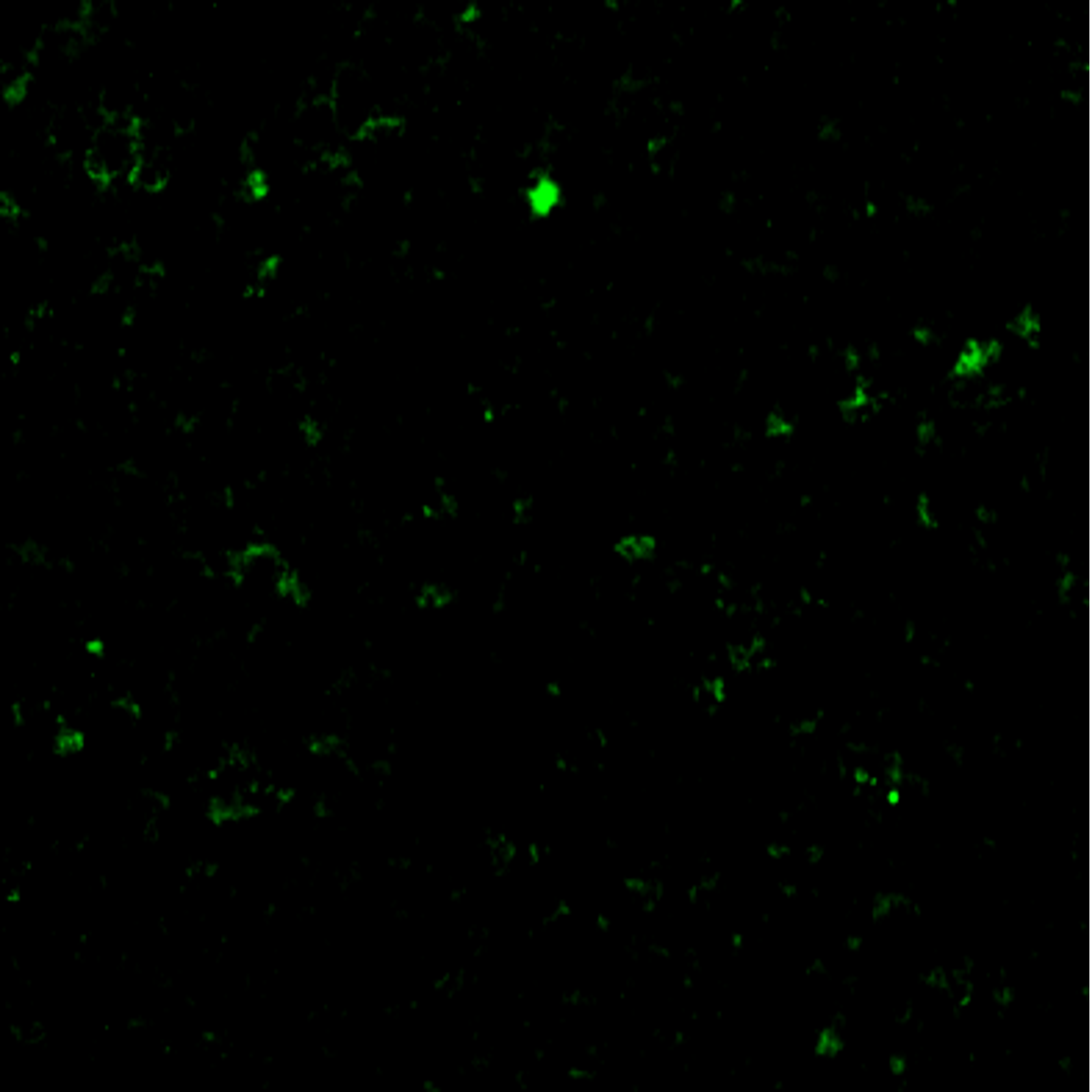

Supplement: Supplementary file 9 — Source data Fig. 7 [file 44321_2026_387_MOESM9_ESM.zip › Fig. 7/Fig. 7G/Fig.7G vehicle f480.tif]

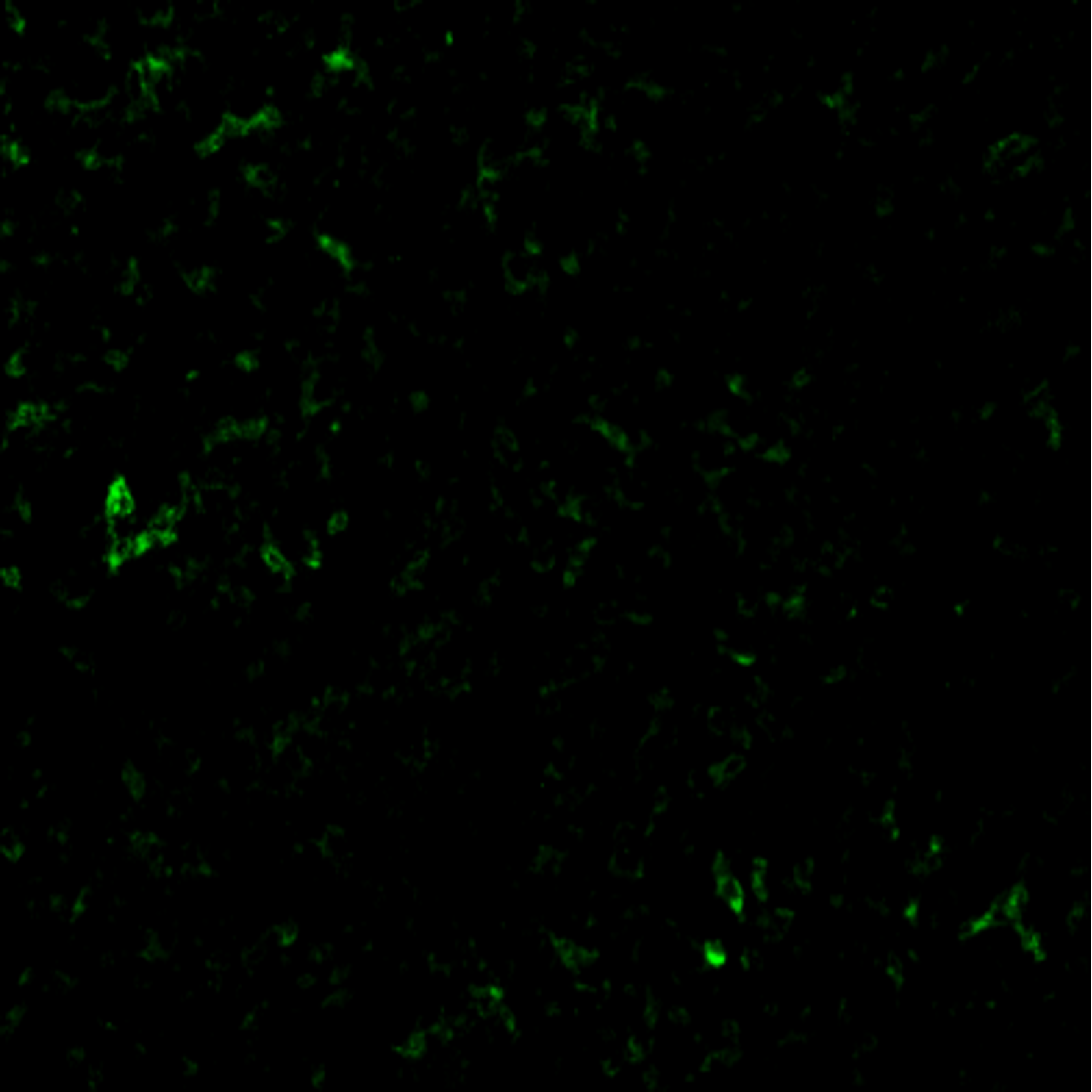

Supplement: Supplementary file 9 — Source data Fig. 7 [file 44321_2026_387_MOESM9_ESM.zip › Fig. 7/Fig. 7G/Fig.7G ds f480.tif]

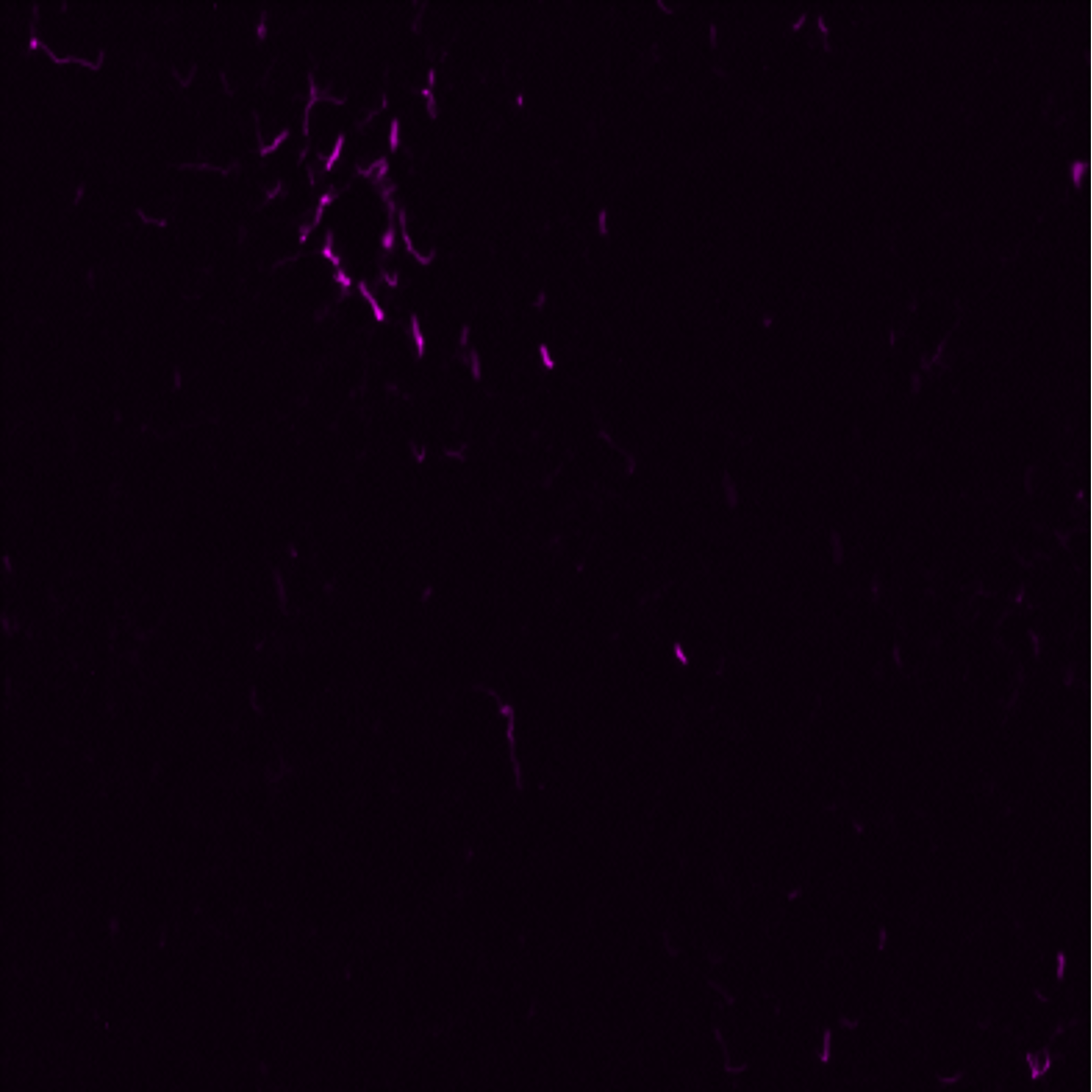

Supplement: Supplementary file 9 — Source data Fig. 7 [file 44321_2026_387_MOESM9_ESM.zip › Fig. 7/Fig. 7G/Fig.7G ds col1.tif]

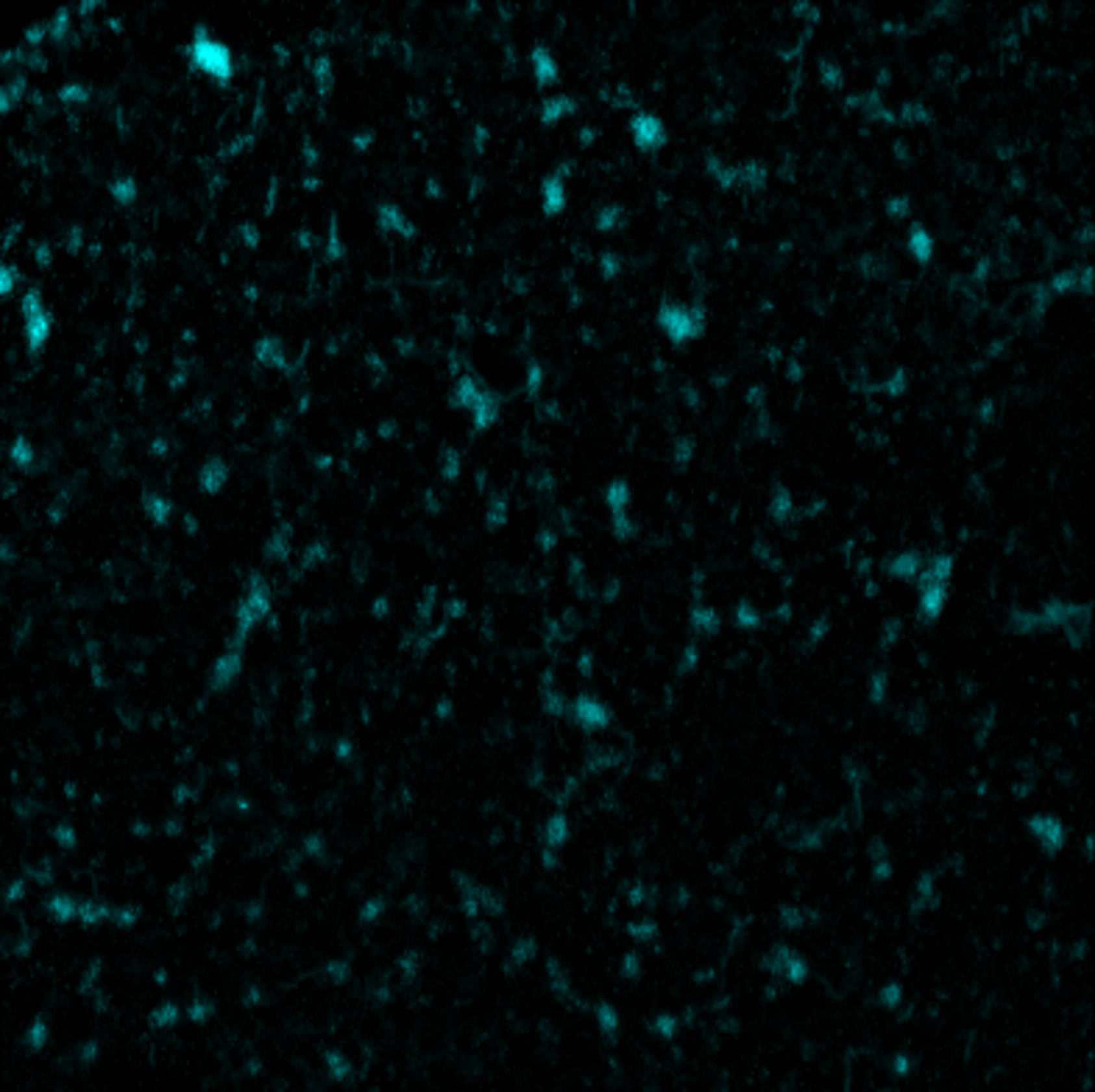

Supplement: Supplementary file 9 — Source data Fig. 7 [file 44321_2026_387_MOESM9_ESM.zip › Fig. 7/Fig. 7G/Fig.7G vehicle DAPI.tif]

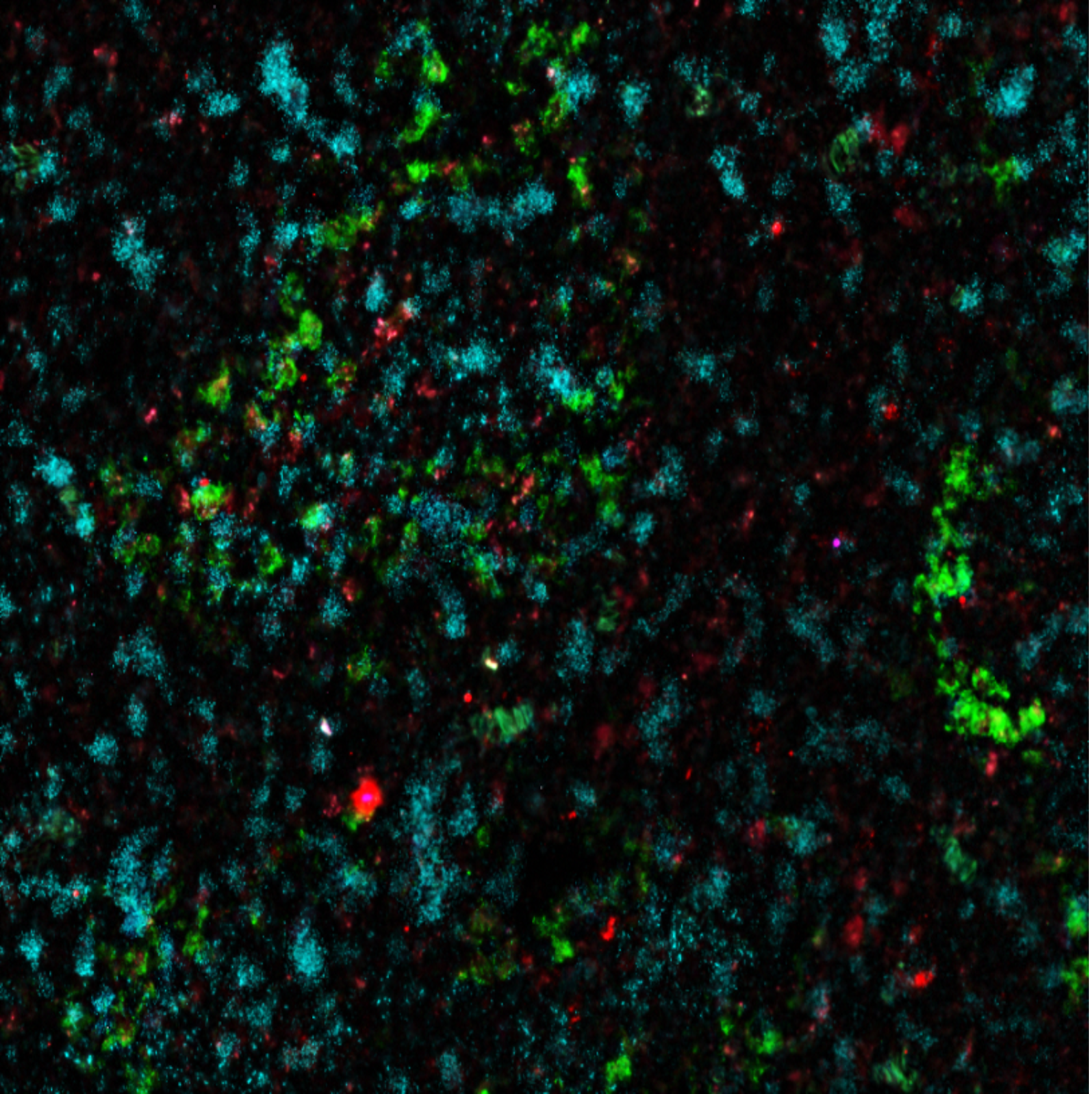

Supplement: Supplementary file 9 — Source data Fig. 7 [file 44321_2026_387_MOESM9_ESM.zip › Fig. 7/Fig. 7I/Fig.7I vehicle merged.tif]

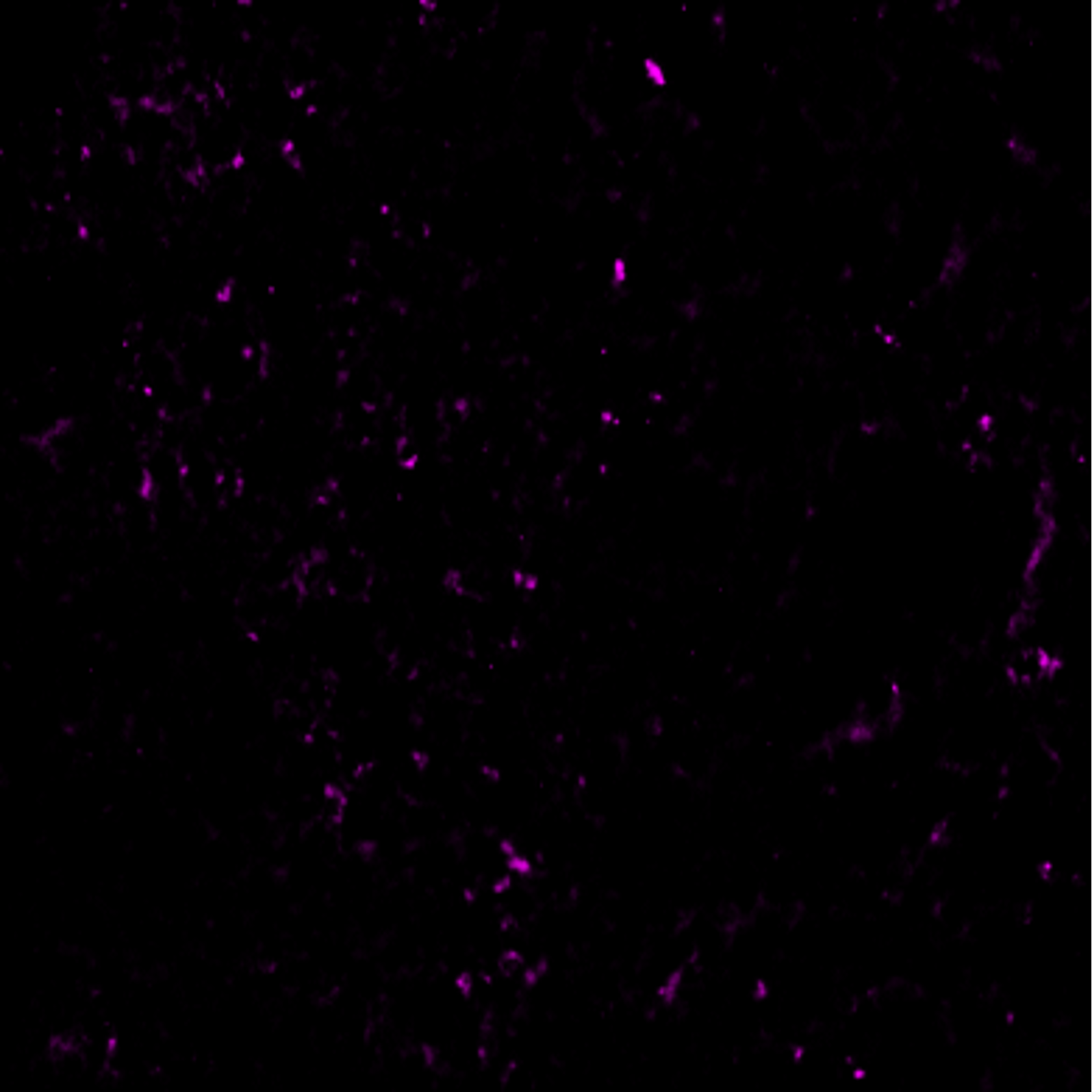

Supplement: Supplementary file 9 — Source data Fig. 7 [file 44321_2026_387_MOESM9_ESM.zip › Fig. 7/Fig. 7I/Fig.7I ds f480.tif]

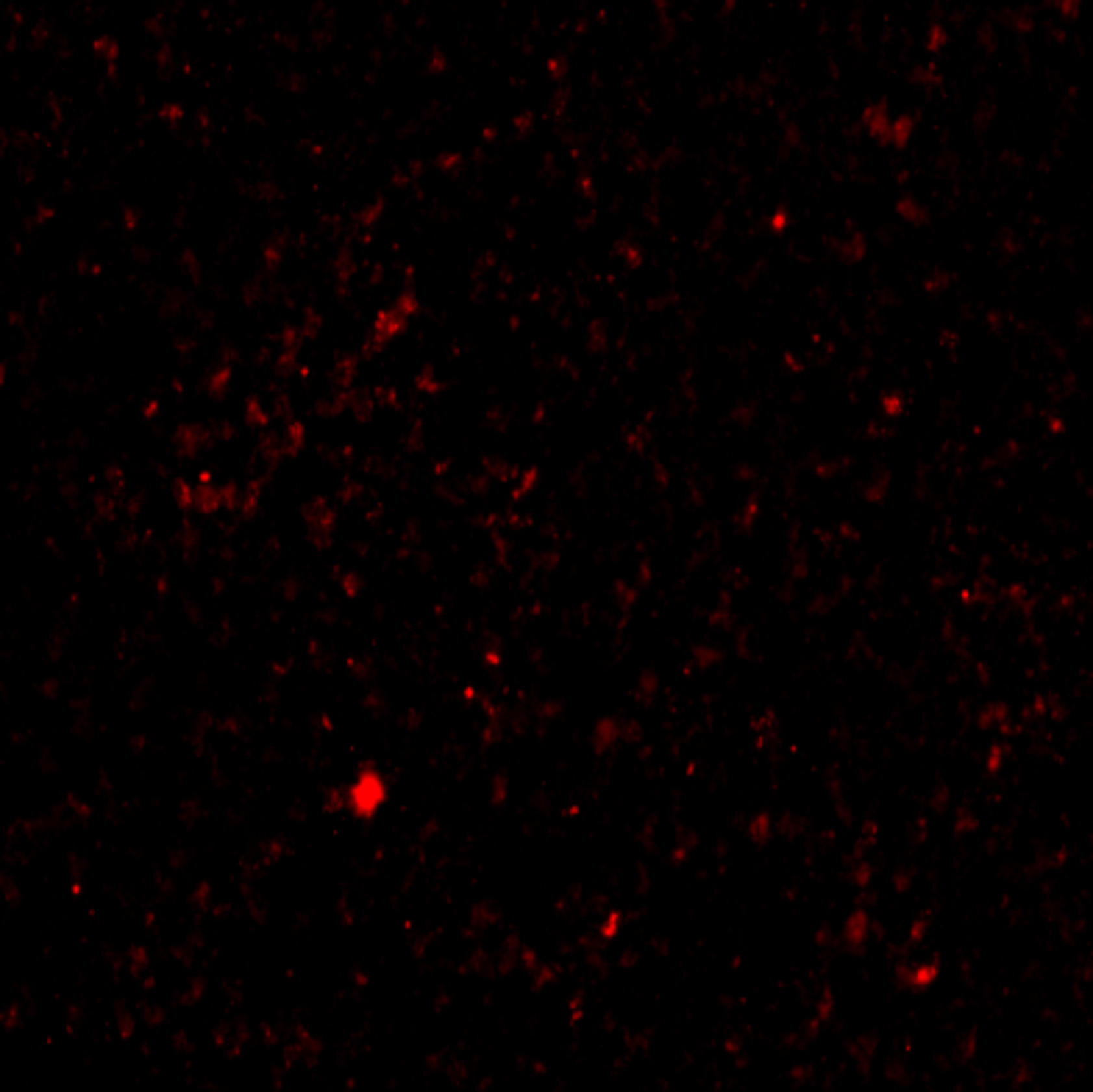

Supplement: Supplementary file 9 — Source data Fig. 7 [file 44321_2026_387_MOESM9_ESM.zip › Fig. 7/Fig. 7I/Fig.7I vehicle lcmv np.tif]

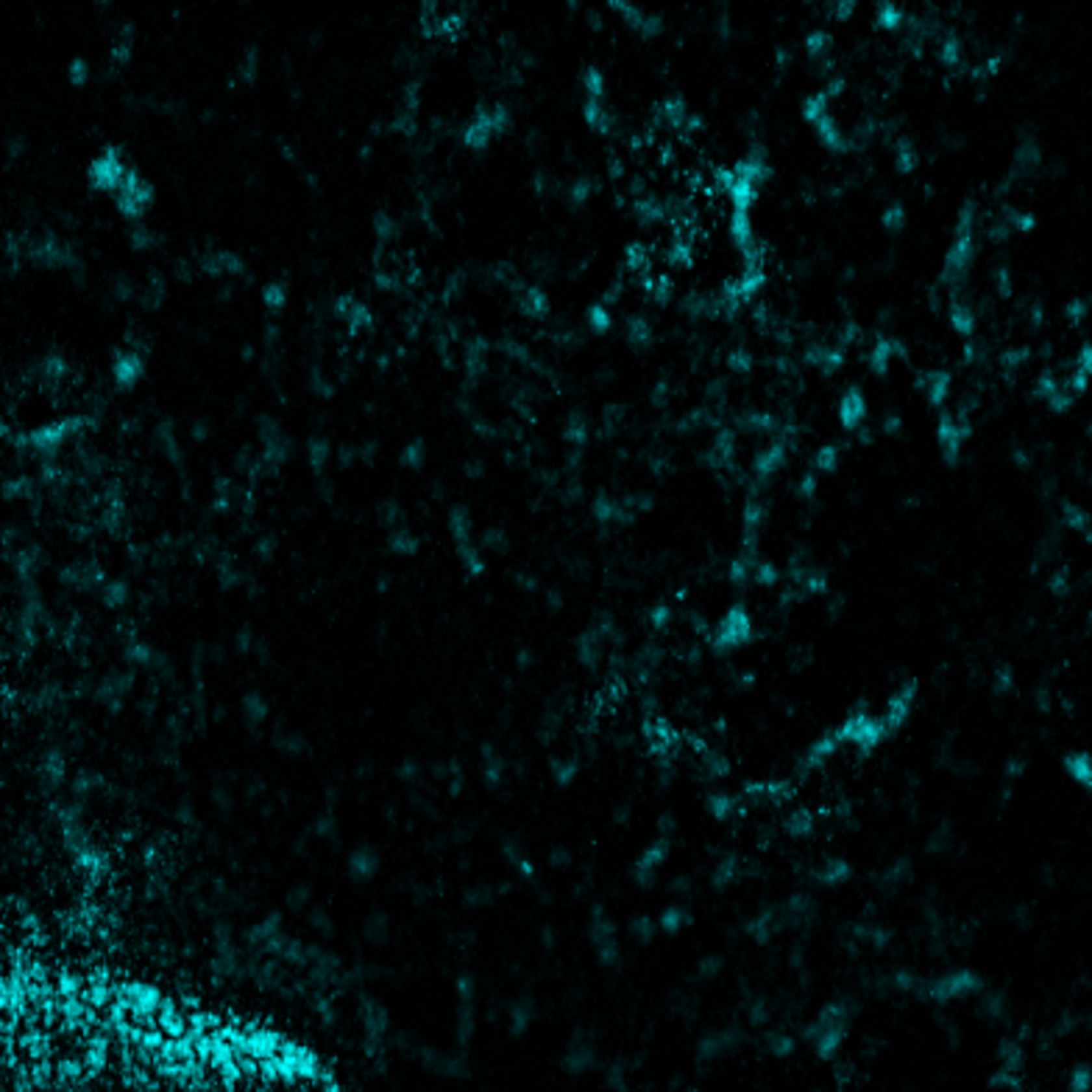

Supplement: Supplementary file 9 — Source data Fig. 7 [file 44321_2026_387_MOESM9_ESM.zip › Fig. 7/Fig. 7I/Fig.7I ds DAPI.tif]

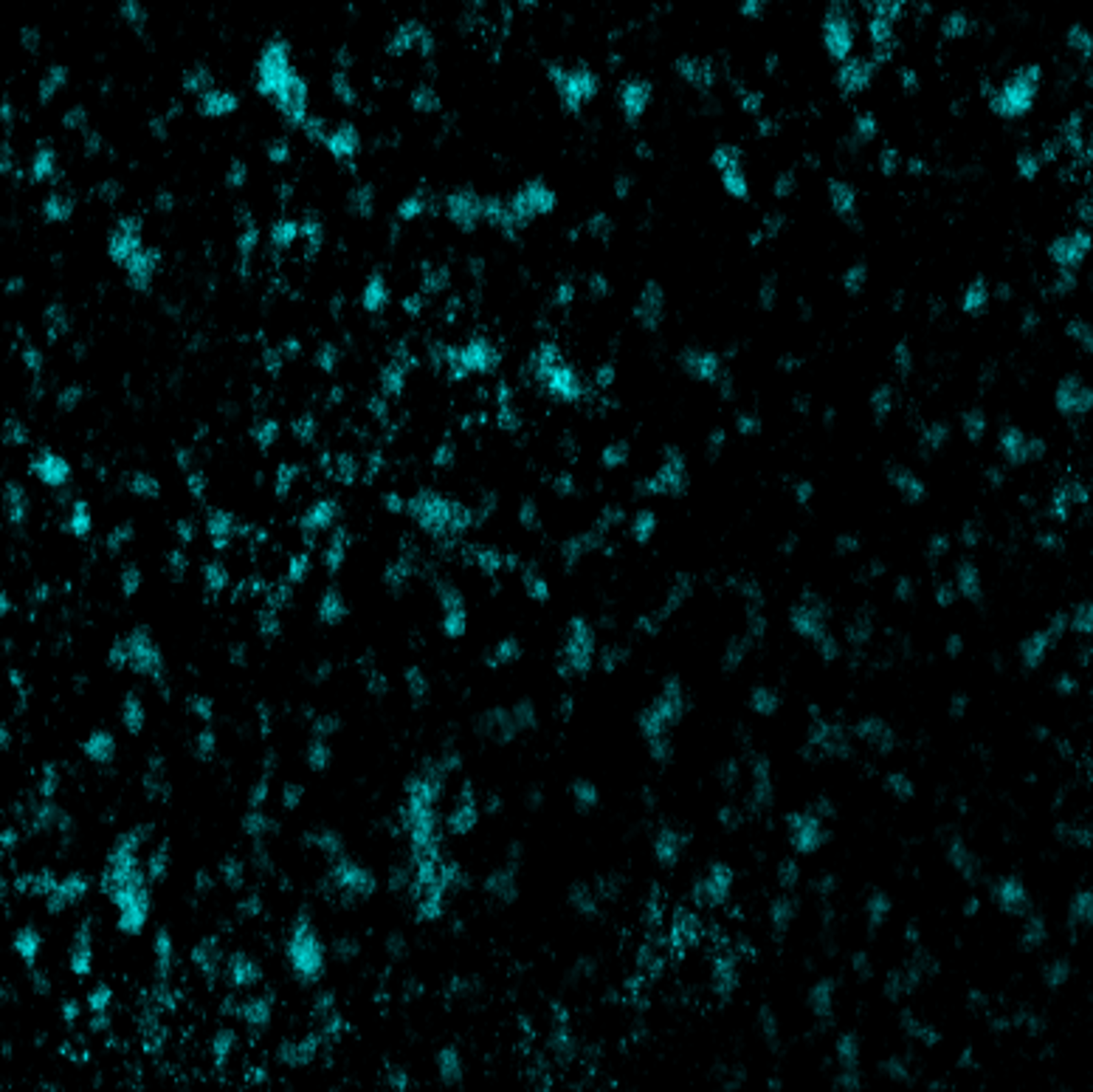

Supplement: Supplementary file 9 — Source data Fig. 7 [file 44321_2026_387_MOESM9_ESM.zip › Fig. 7/Fig. 7I/Fig.7I vehicle DAPI.tif]

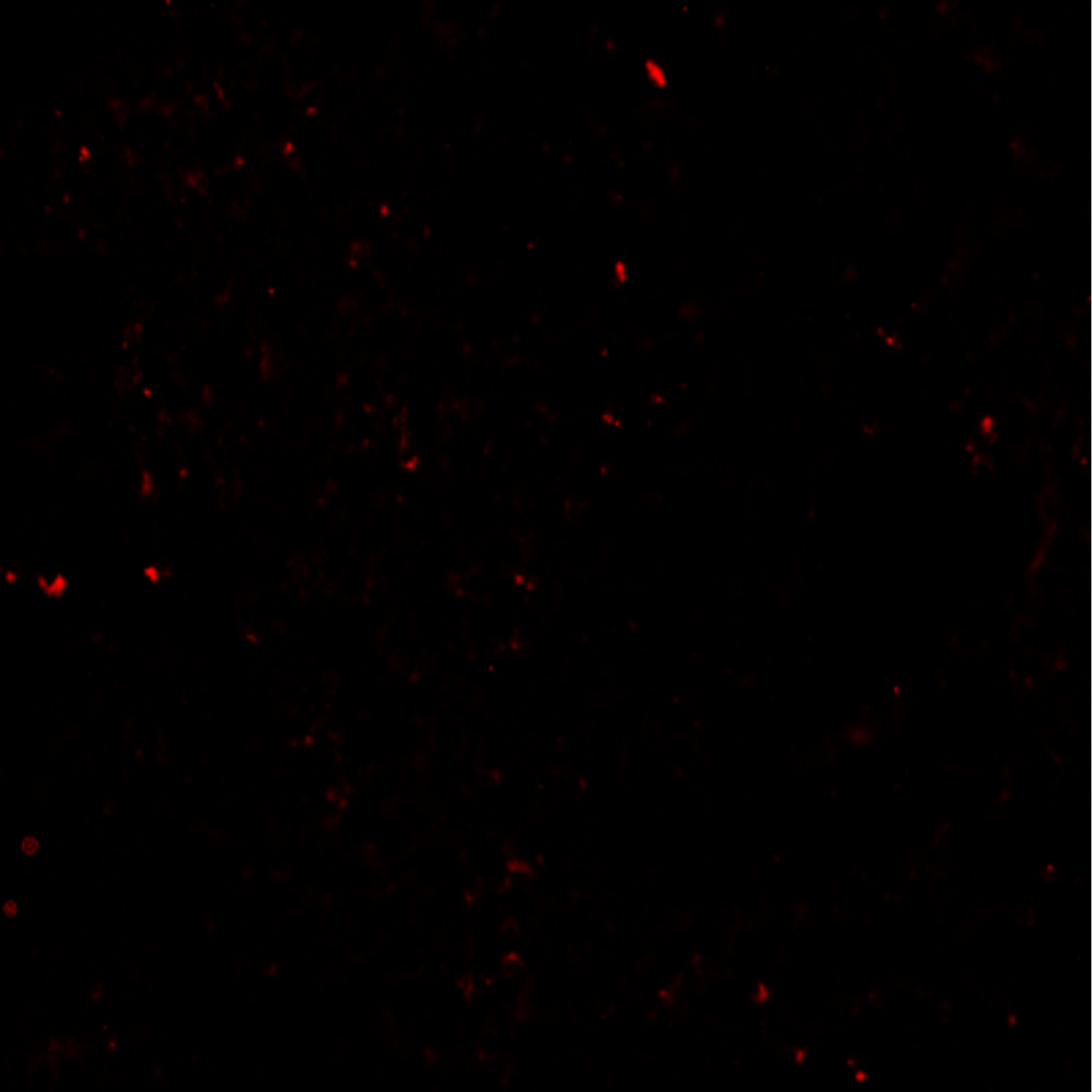

Supplement: Supplementary file 9 — Source data Fig. 7 [file 44321_2026_387_MOESM9_ESM.zip › Fig. 7/Fig. 7I/Fig.7I ds lcmv np.tif]

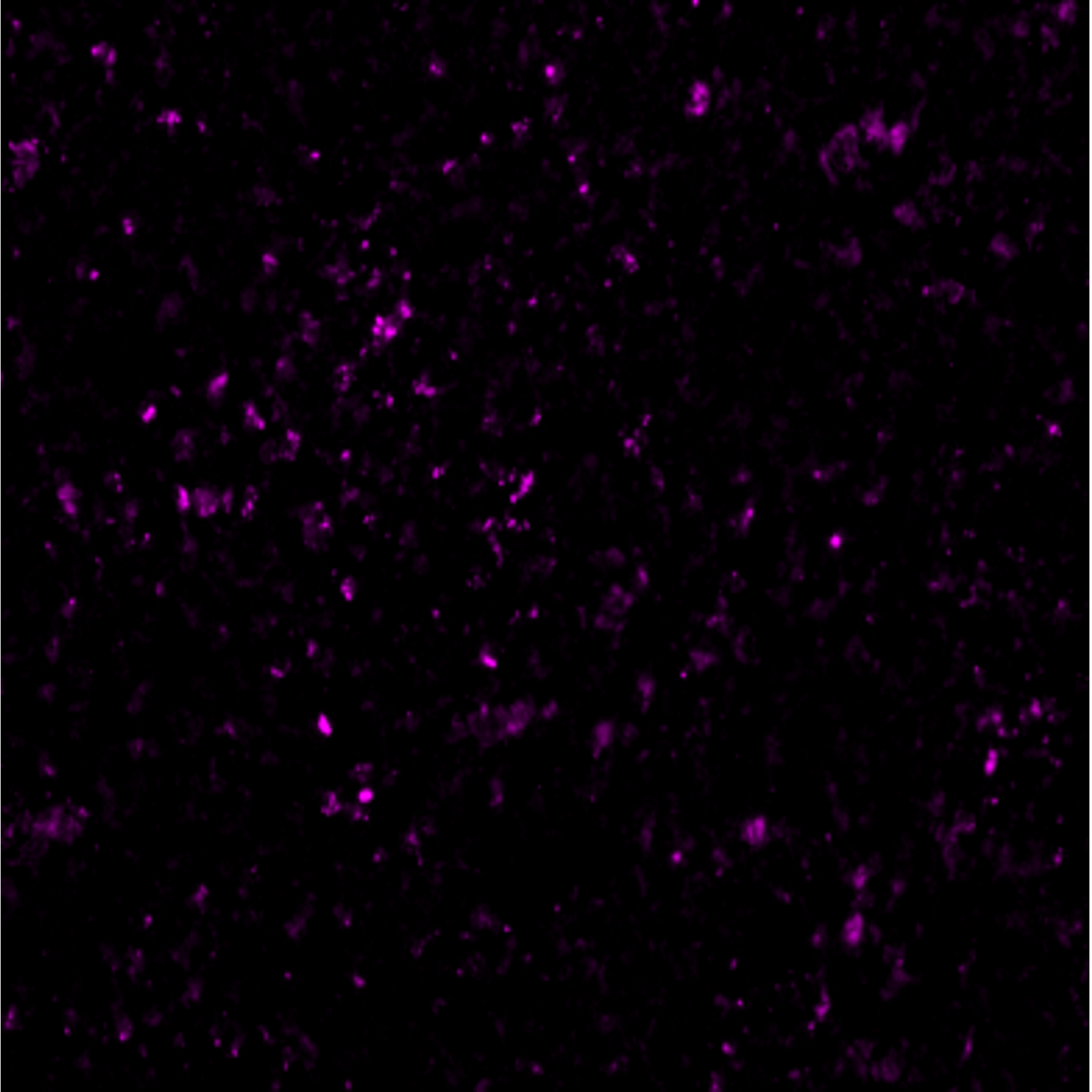

Supplement: Supplementary file 9 — Source data Fig. 7 [file 44321_2026_387_MOESM9_ESM.zip › Fig. 7/Fig. 7I/Fig.7I vehicle f480.tif]

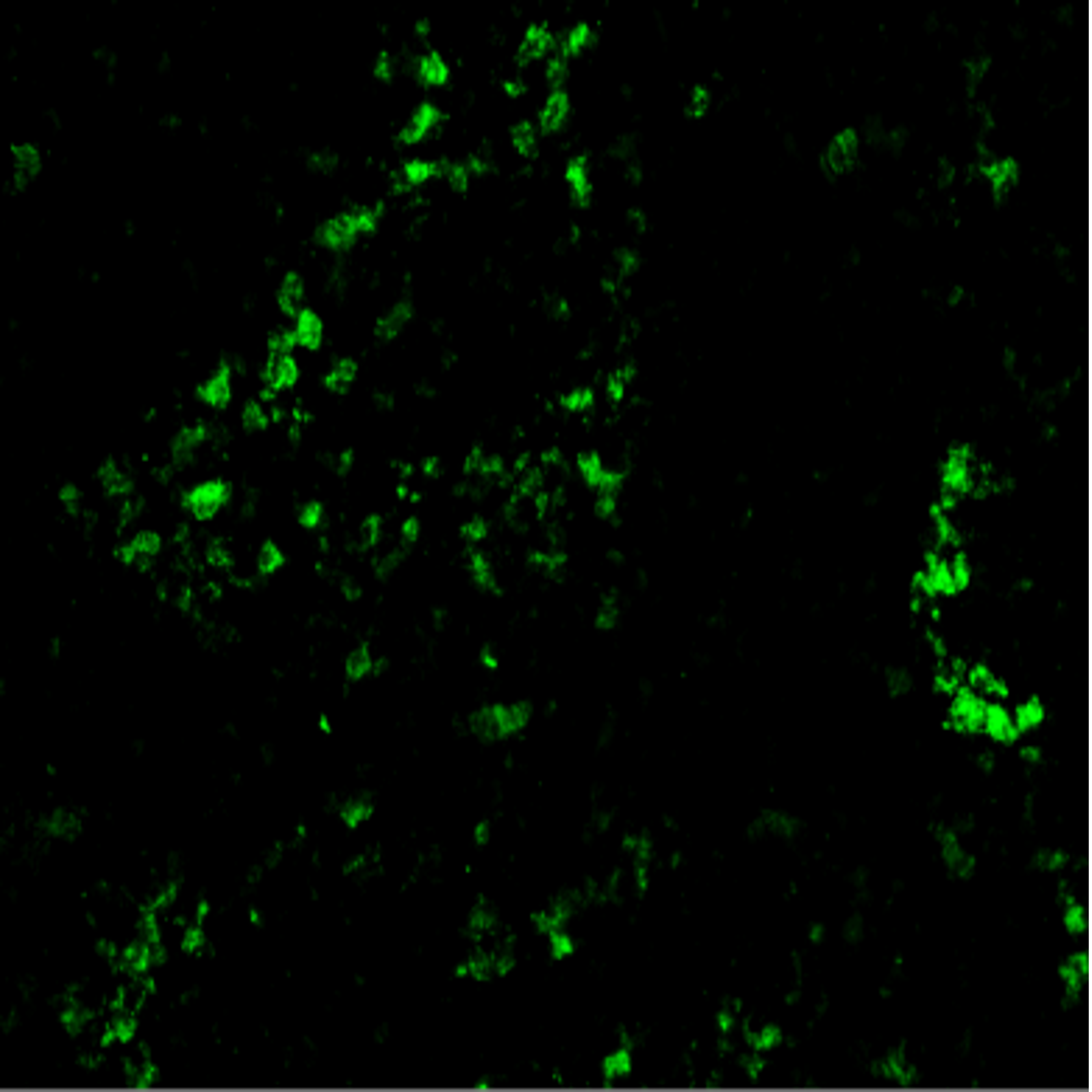

Supplement: Supplementary file 9 — Source data Fig. 7 [file 44321_2026_387_MOESM9_ESM.zip › Fig. 7/Fig. 7I/Fig.7I vehicle cd169.tif]

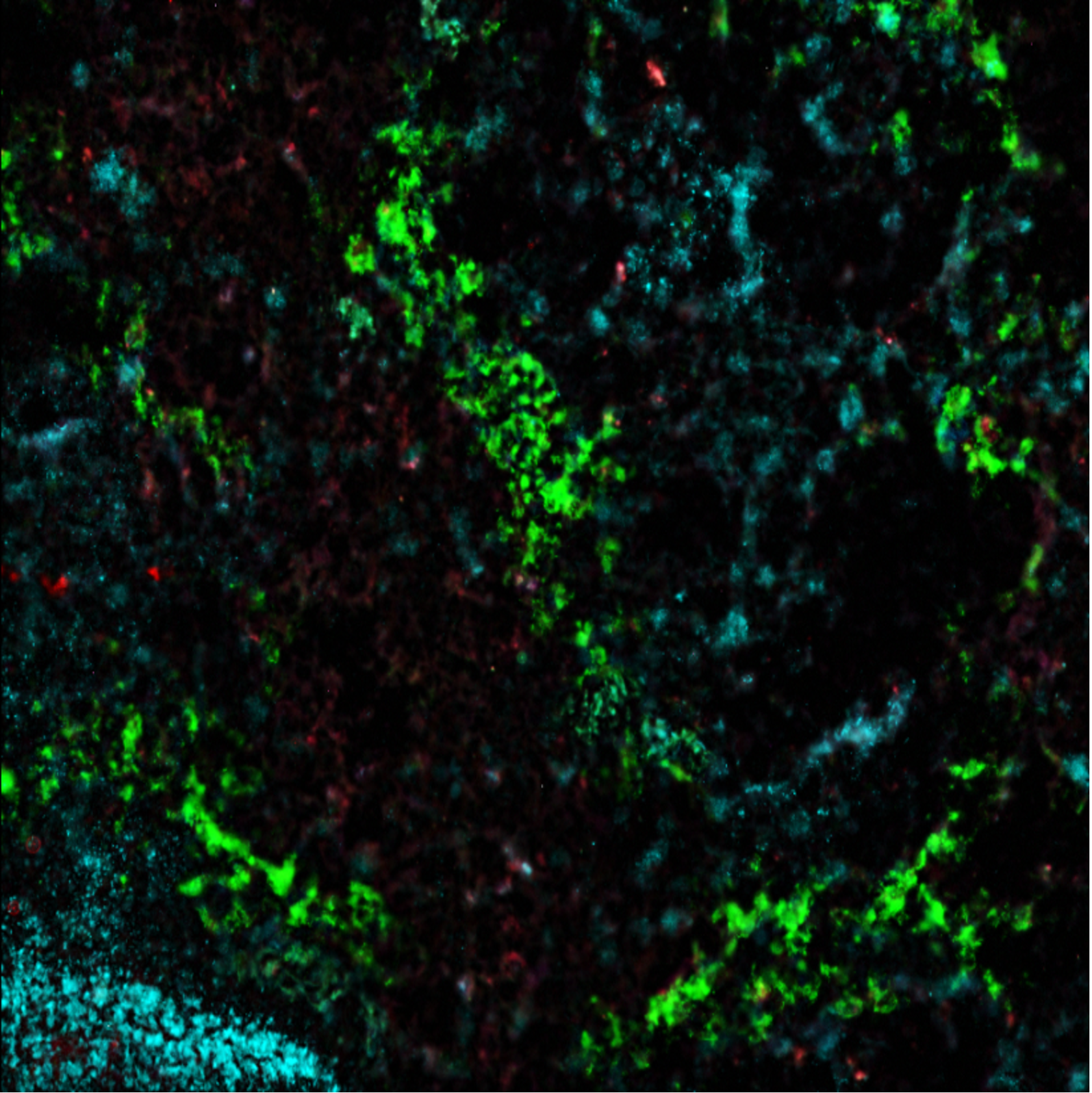

Supplement: Supplementary file 9 — Source data Fig. 7 [file 44321_2026_387_MOESM9_ESM.zip › Fig. 7/Fig. 7I/Fig.7I ds merged.tif]

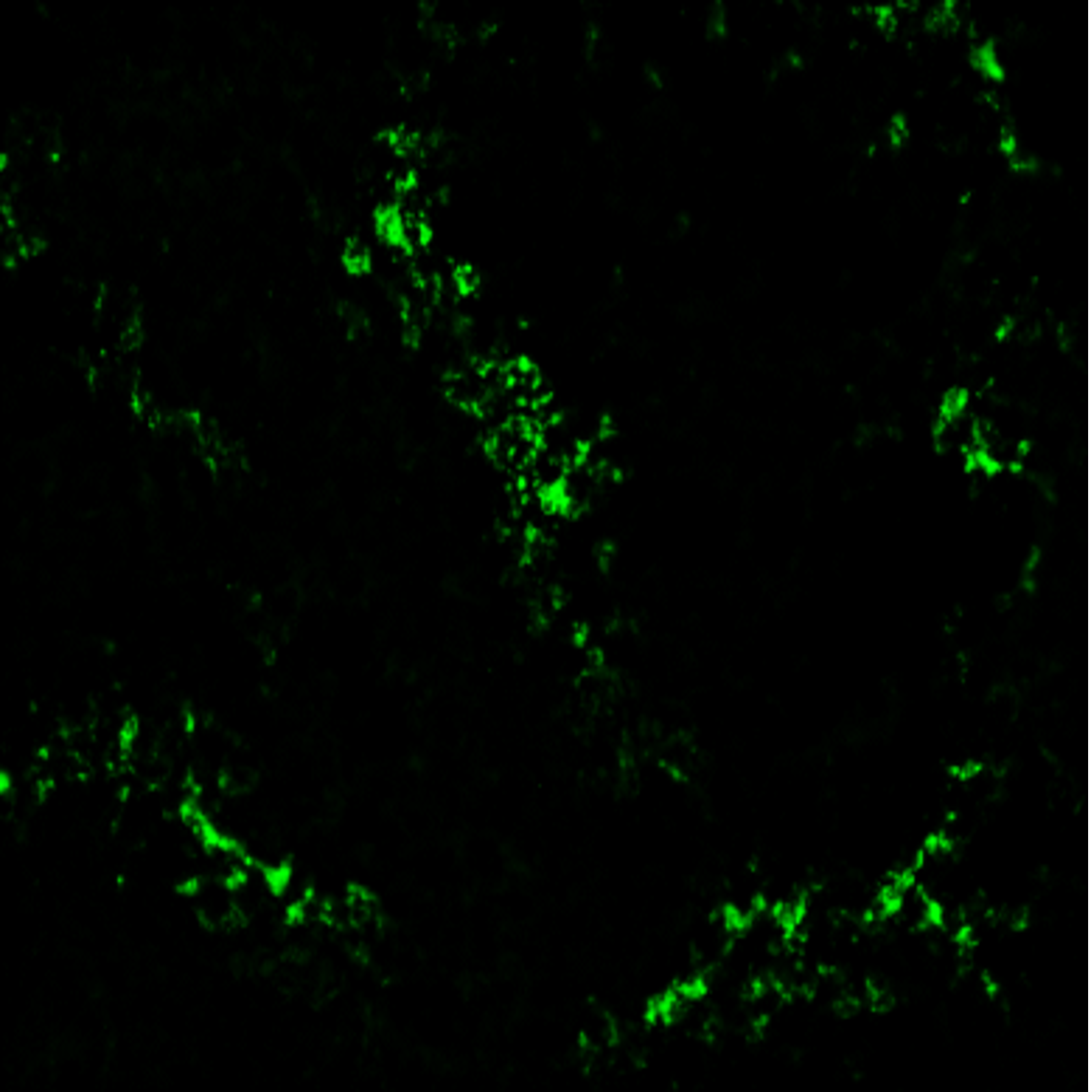

Supplement: Supplementary file 9 — Source data Fig. 7 [file 44321_2026_387_MOESM9_ESM.zip › Fig. 7/Fig. 7I/Fig.7I ds cd169.tif]

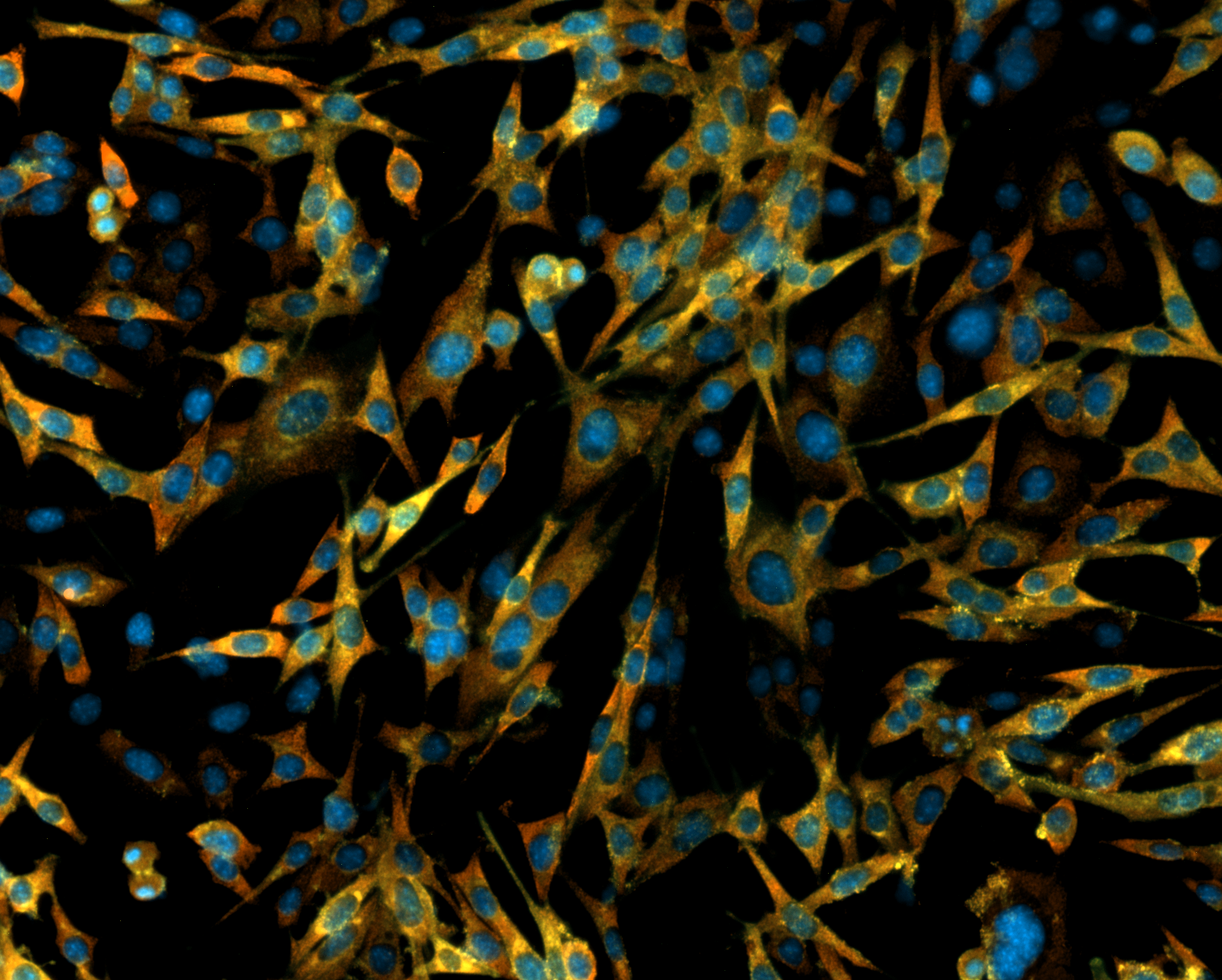

Supplement: Supplementary file 15 — Appendix Figure Source Data [file 44321_2026_387_MOESM15_ESM.zip › Appendix Fig. S1/Fig. S1C/H5_1-Image Export-39_c0-3x0-2752y0-2208.tif]

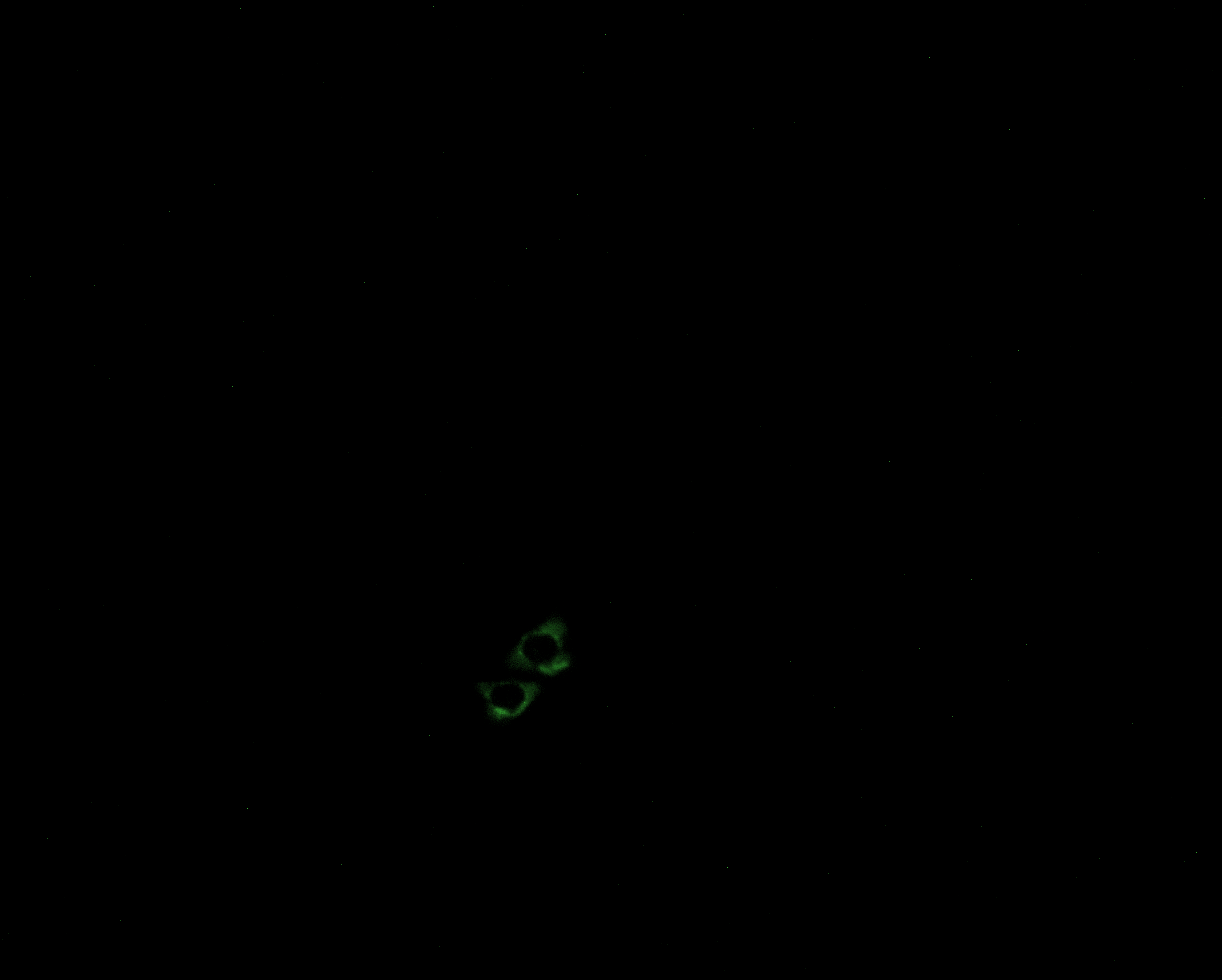

Supplement: Supplementary file 15 — Appendix Figure Source Data [file 44321_2026_387_MOESM15_ESM.zip › Appendix Fig. S1/Fig. S1C/H1_6-Image Export-14_c2x0-2752y0-2208_NP_5000.tif]

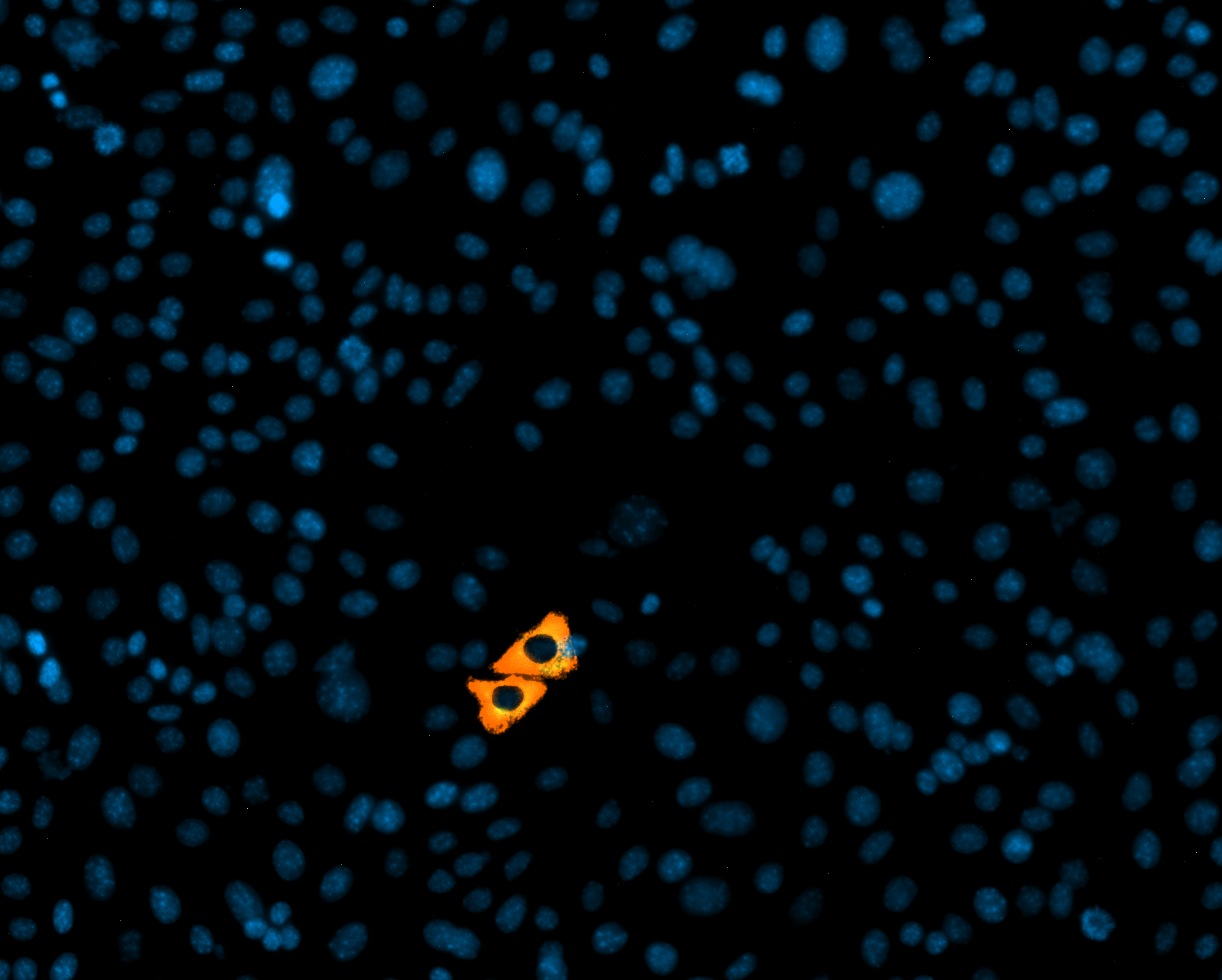

Supplement: Supplementary file 15 — Appendix Figure Source Data [file 44321_2026_387_MOESM15_ESM.zip › Appendix Fig. S1/Fig. S1C/H1_6-Image Export-14_c0-3x0-2752y0-2208.tif]

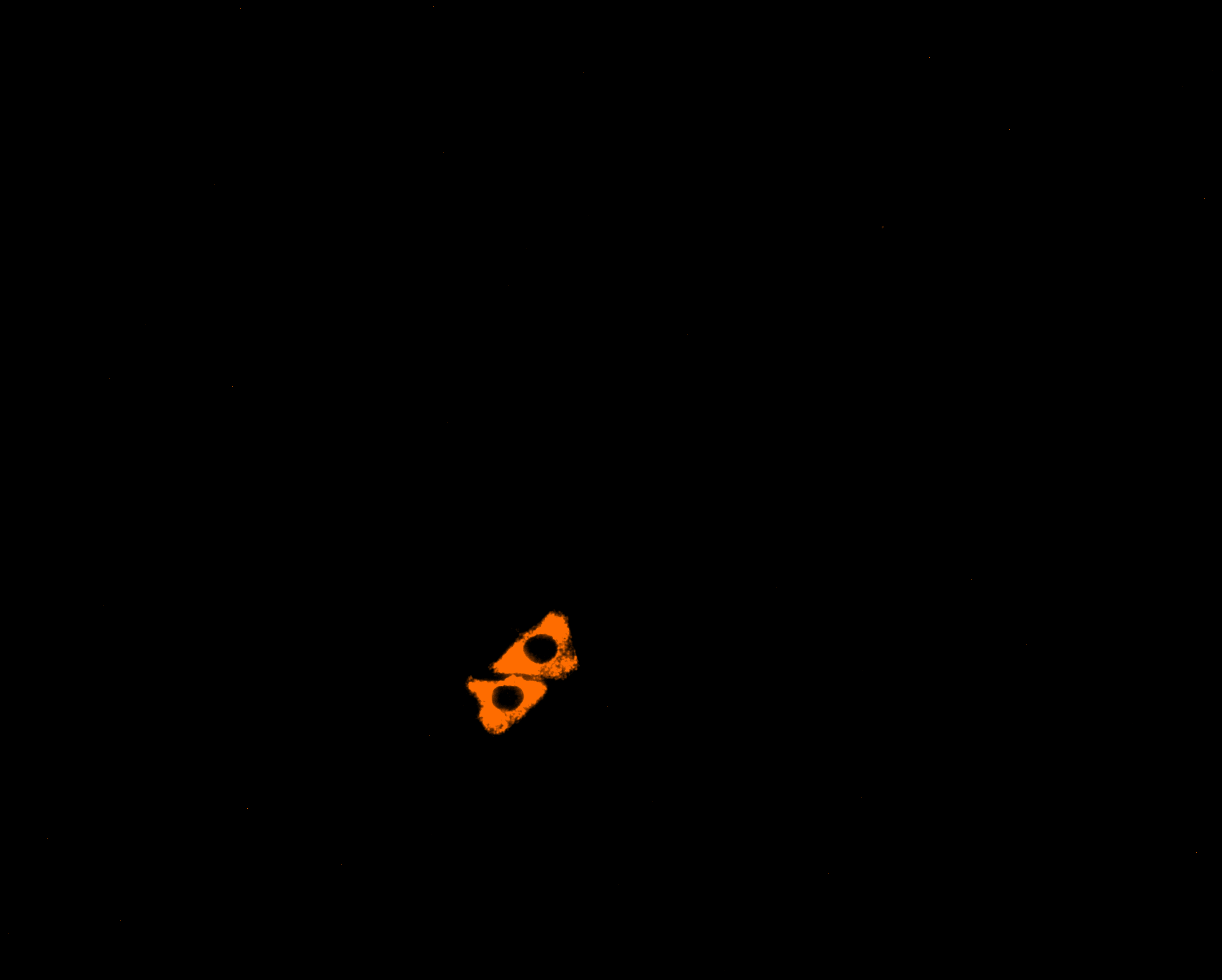

Supplement: Supplementary file 15 — Appendix Figure Source Data [file 44321_2026_387_MOESM15_ESM.zip › Appendix Fig. S1/Fig. S1C/H1_6-Image Export-14_c1x0-2752y0-2208_GP_5000.tif]

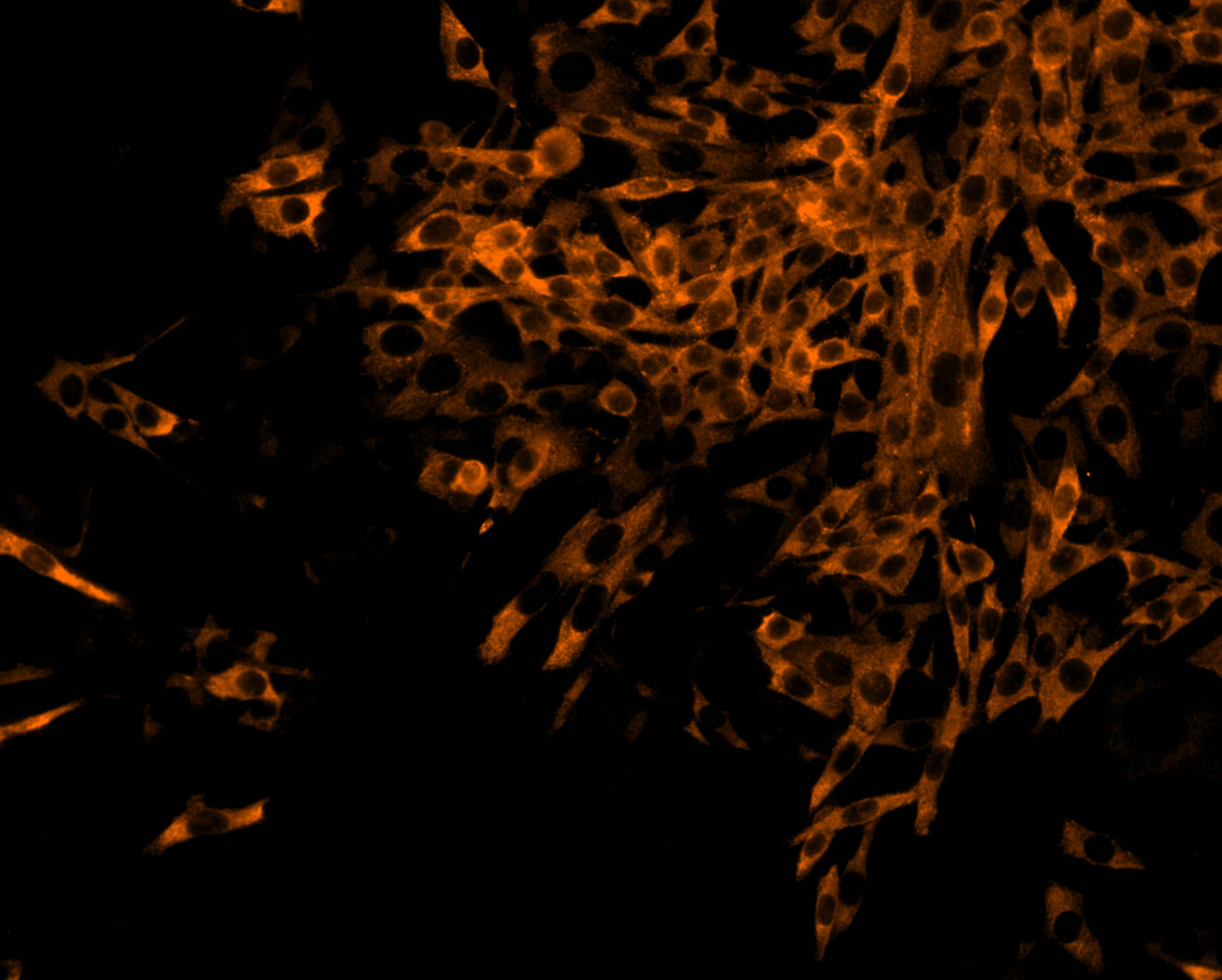

Supplement: Supplementary file 15 — Appendix Figure Source Data [file 44321_2026_387_MOESM15_ESM.zip › Appendix Fig. S1/Fig. S1C/H4_7-Image Export-38_c1x0-2752y0-2208_GP_5.tif]

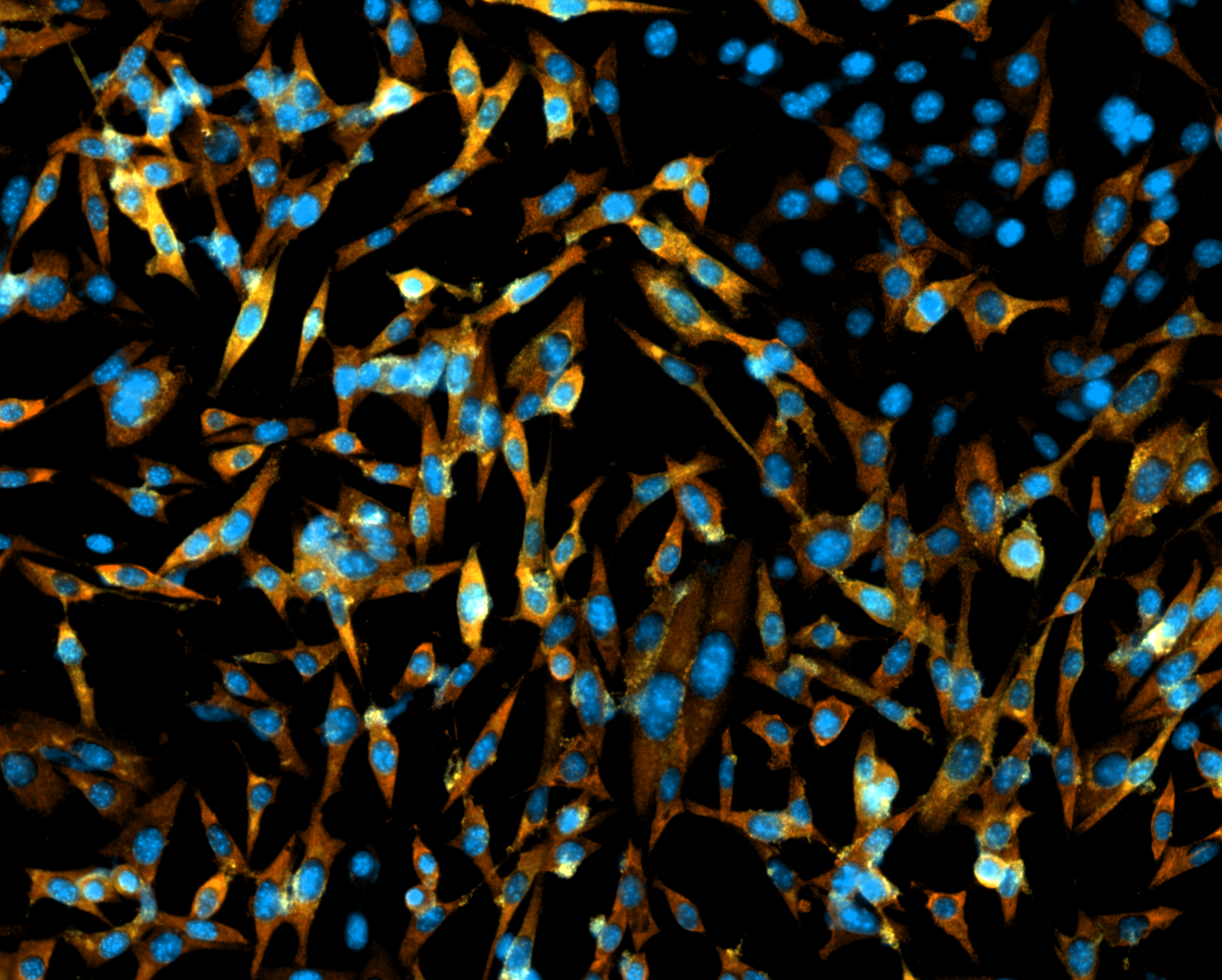

Supplement: Supplementary file 15 — Appendix Figure Source Data [file 44321_2026_387_MOESM15_ESM.zip › Appendix Fig. S1/Fig. S1C/H6_2-Image Export-44_c0-3x0-2752y0-2208.tif]

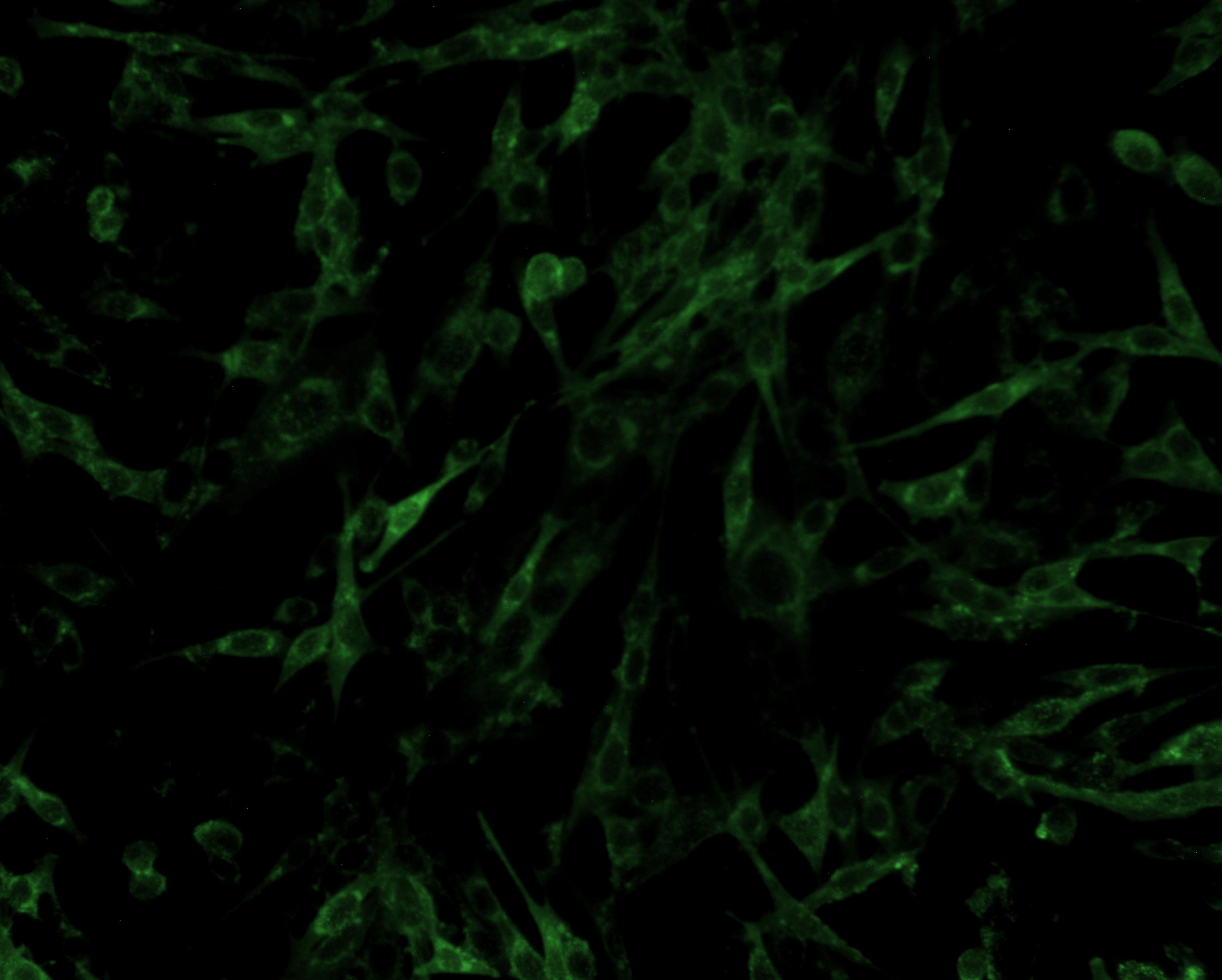

Supplement: Supplementary file 15 — Appendix Figure Source Data [file 44321_2026_387_MOESM15_ESM.zip › Appendix Fig. S1/Fig. S1C/H5_1-Image Export-39_c2x0-2752y0-2208_NP_0.5.tif]

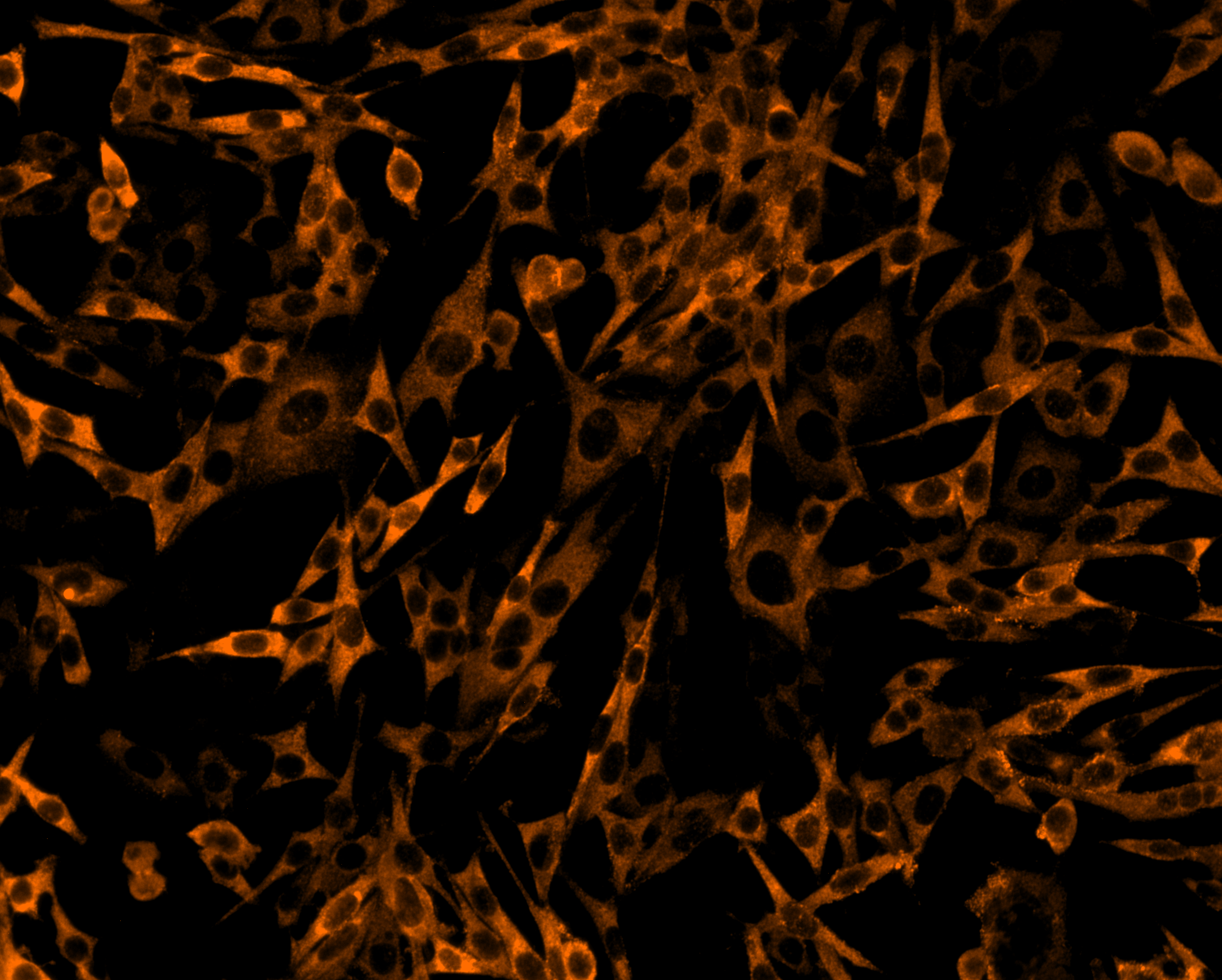

Supplement: Supplementary file 15 — Appendix Figure Source Data [file 44321_2026_387_MOESM15_ESM.zip › Appendix Fig. S1/Fig. S1C/H5_1-Image Export-39_c1x0-2752y0-2208_GP_0.5.tif]

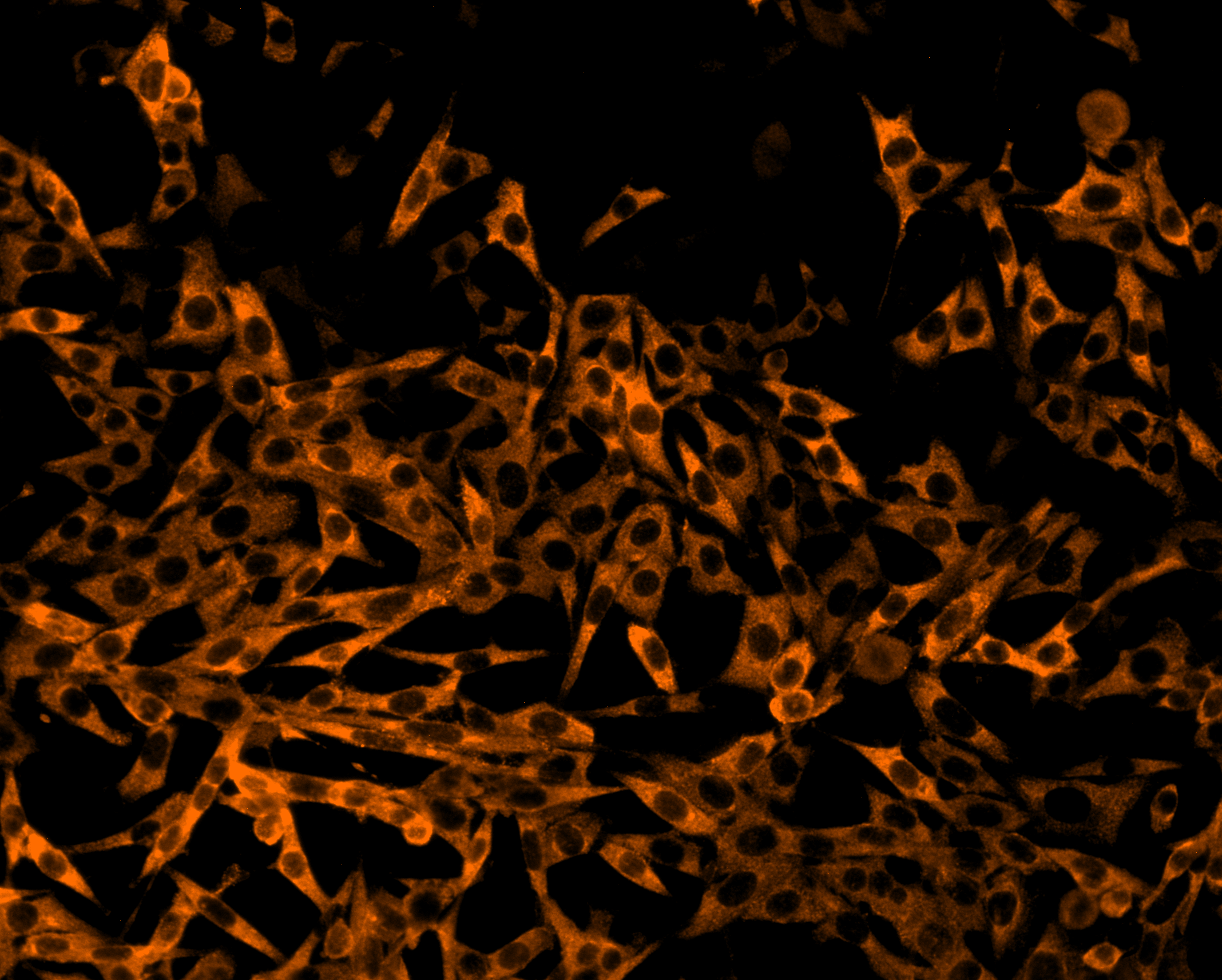

Supplement: Supplementary file 15 — Appendix Figure Source Data [file 44321_2026_387_MOESM15_ESM.zip › Appendix Fig. S1/Fig. S1C/H3_3-Image Export-27_c1x0-2752y0-2208_GP_50.tif]

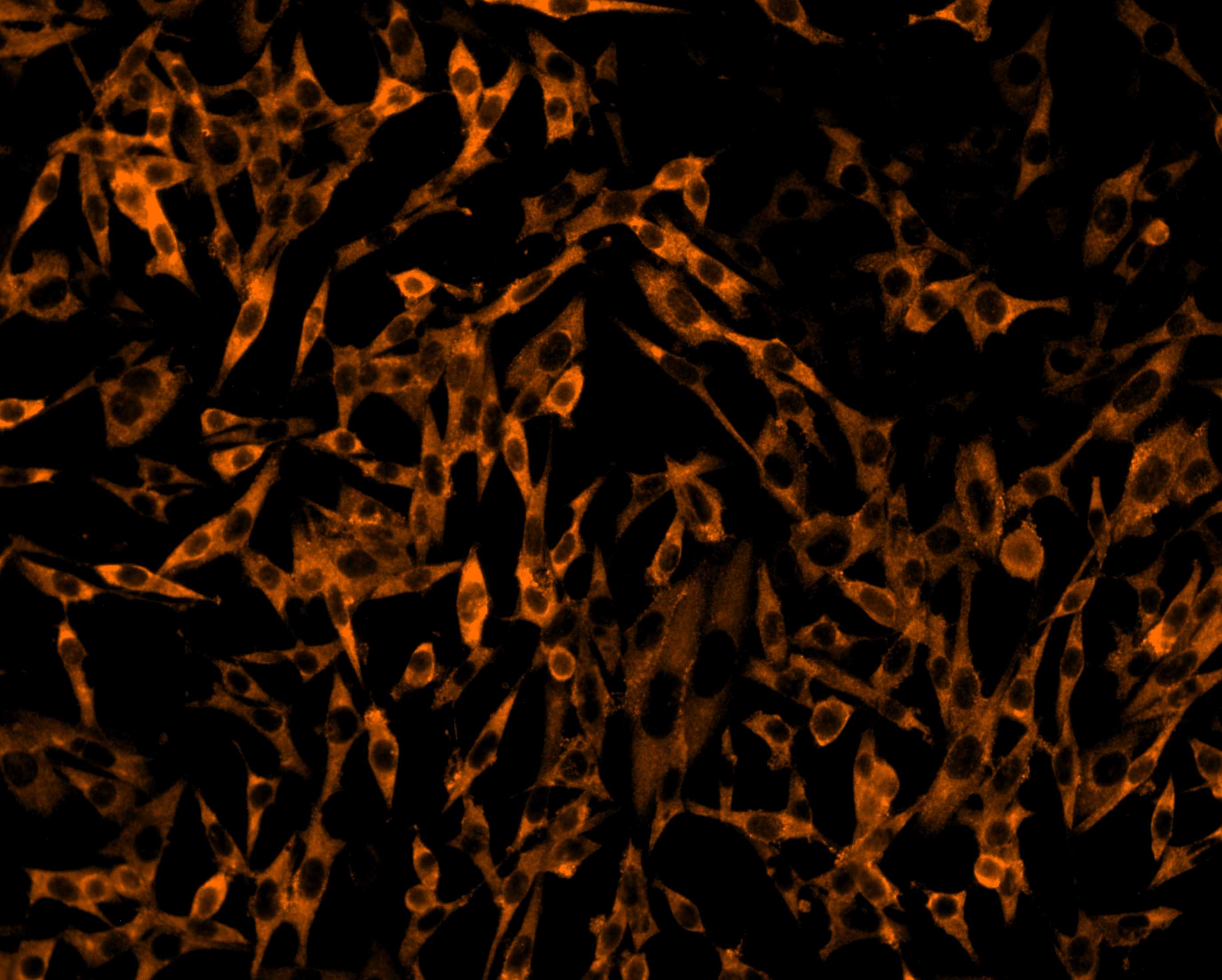

Supplement: Supplementary file 15 — Appendix Figure Source Data [file 44321_2026_387_MOESM15_ESM.zip › Appendix Fig. S1/Fig. S1C/H6_2-Image Export-44_c1x0-2752y0-2208_GP_0.05.tif]

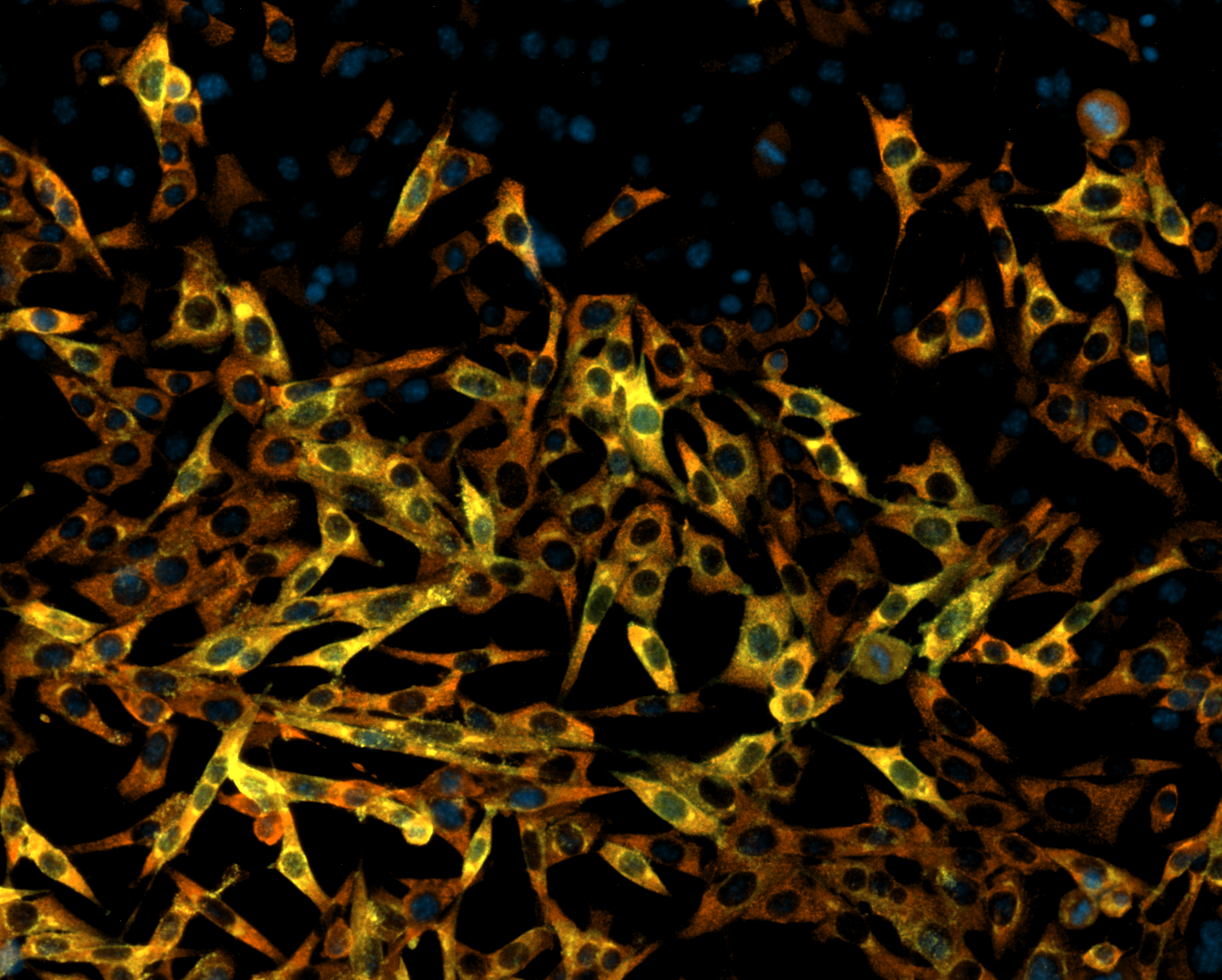

Supplement: Supplementary file 15 — Appendix Figure Source Data [file 44321_2026_387_MOESM15_ESM.zip › Appendix Fig. S1/Fig. S1C/H3_3-Image Export-27_c0-3x0-2752y0-2208.tif]

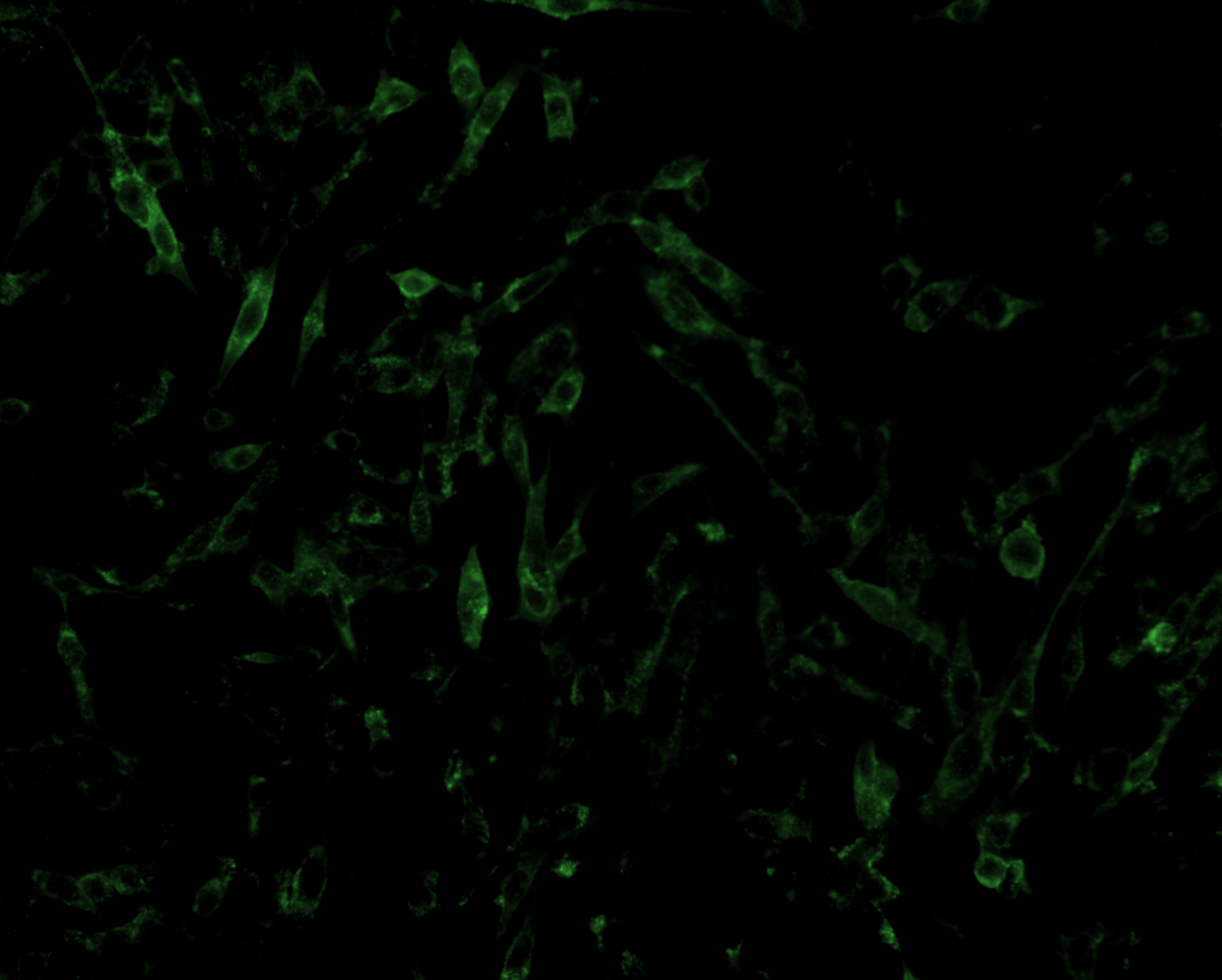

Supplement: Supplementary file 15 — Appendix Figure Source Data [file 44321_2026_387_MOESM15_ESM.zip › Appendix Fig. S1/Fig. S1C/H6_2-Image Export-44_c2x0-2752y0-2208_NP_0.05.tif]

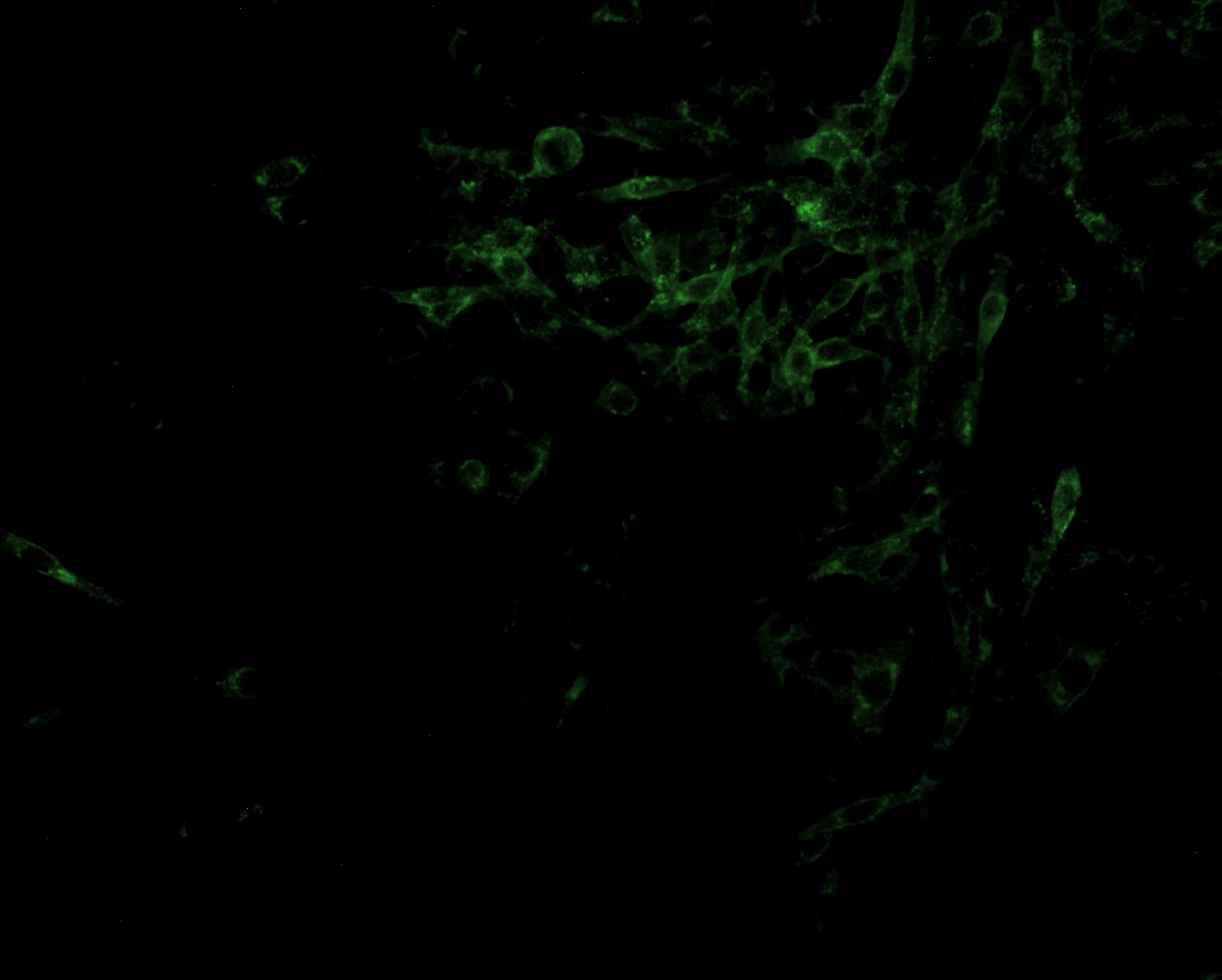

Supplement: Supplementary file 15 — Appendix Figure Source Data [file 44321_2026_387_MOESM15_ESM.zip › Appendix Fig. S1/Fig. S1C/H4_7-Image Export-38_c2x0-2752y0-2208_NP_5.tif]

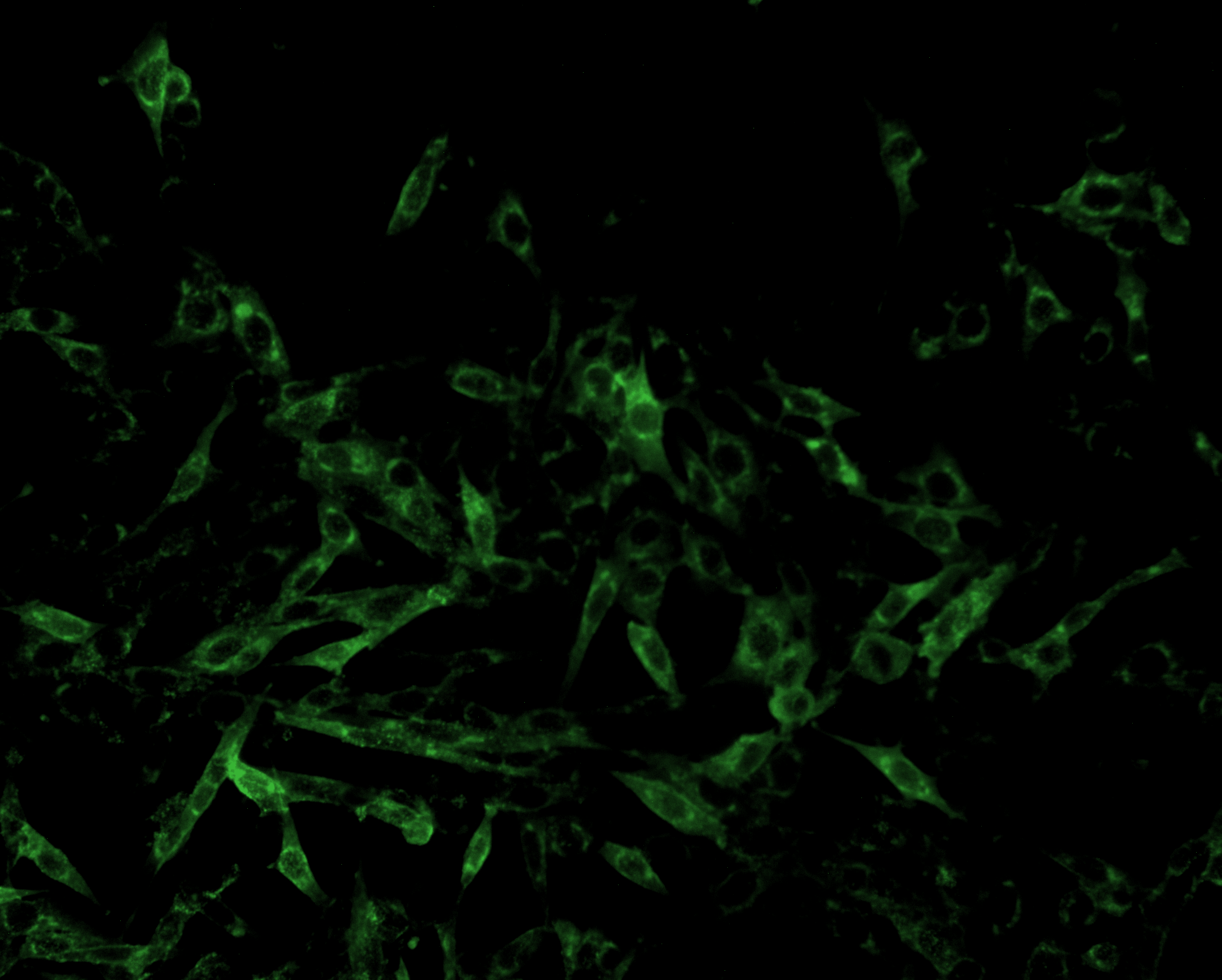

Supplement: Supplementary file 15 — Appendix Figure Source Data [file 44321_2026_387_MOESM15_ESM.zip › Appendix Fig. S1/Fig. S1C/H3_3-Image Export-27_c2x0-2752y0-2208_NP_50.tif]

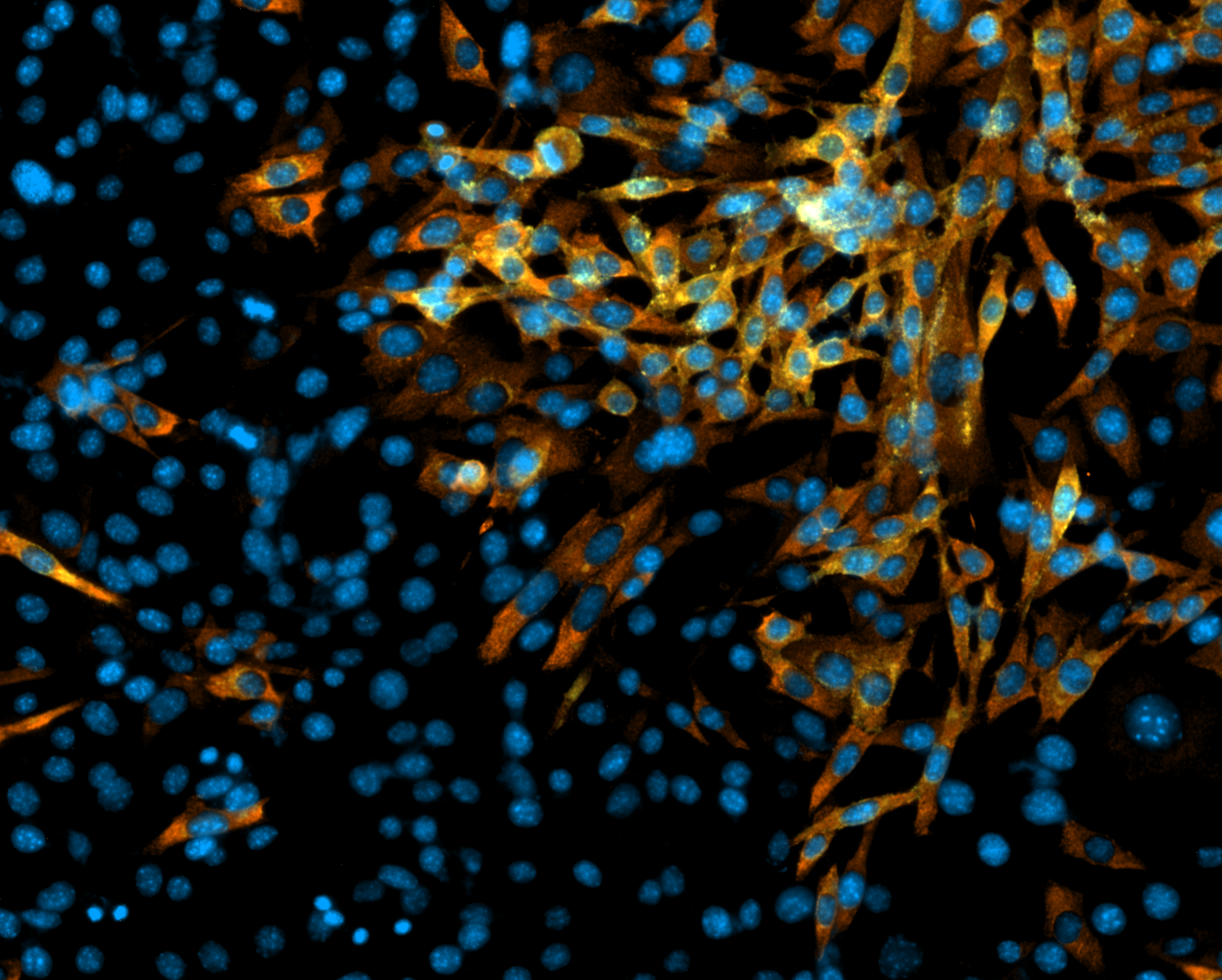

Supplement: Supplementary file 15 — Appendix Figure Source Data [file 44321_2026_387_MOESM15_ESM.zip › Appendix Fig. S1/Fig. S1C/H4_7-Image Export-38_c0-3x0-2752y0-2208.tif]

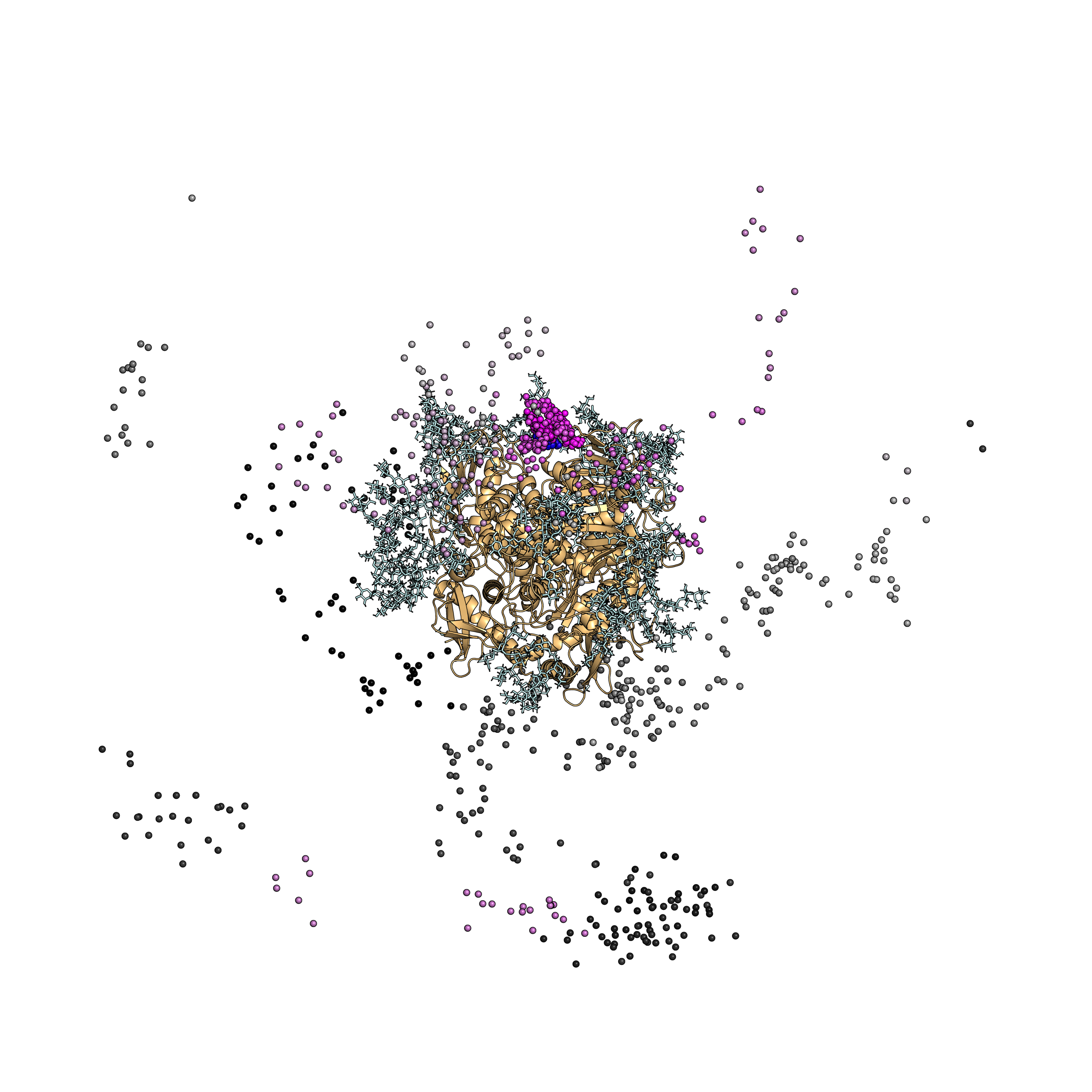

Supplement: Supplementary file 15 — Appendix Figure Source Data [file 44321_2026_387_MOESM15_ESM.zip › Appendix Fig. S5/Fig. S5B/Replica_6.png]

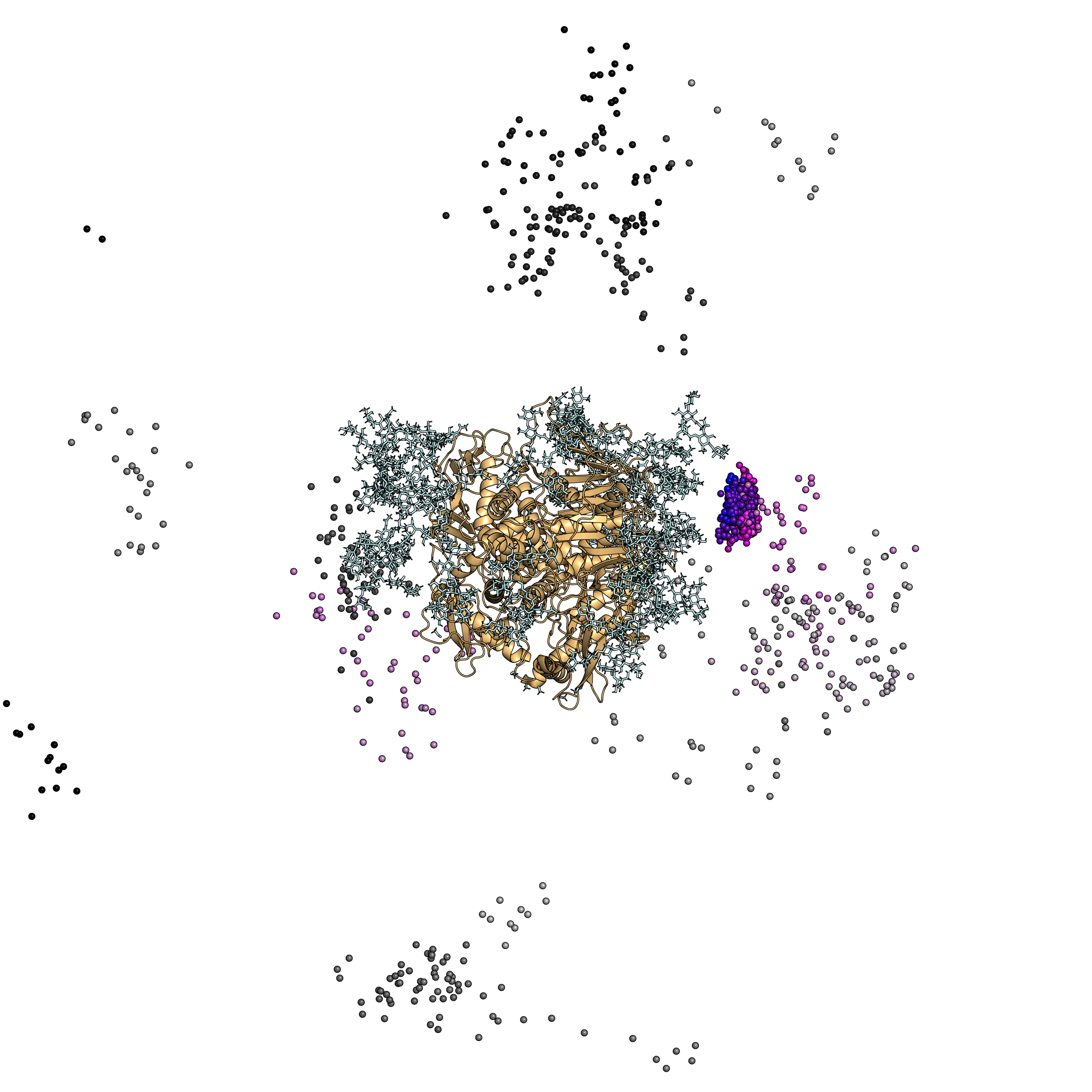

Supplement: Supplementary file 15 — Appendix Figure Source Data [file 44321_2026_387_MOESM15_ESM.zip › Appendix Fig. S5/Fig. S5B/Replica_7.png]

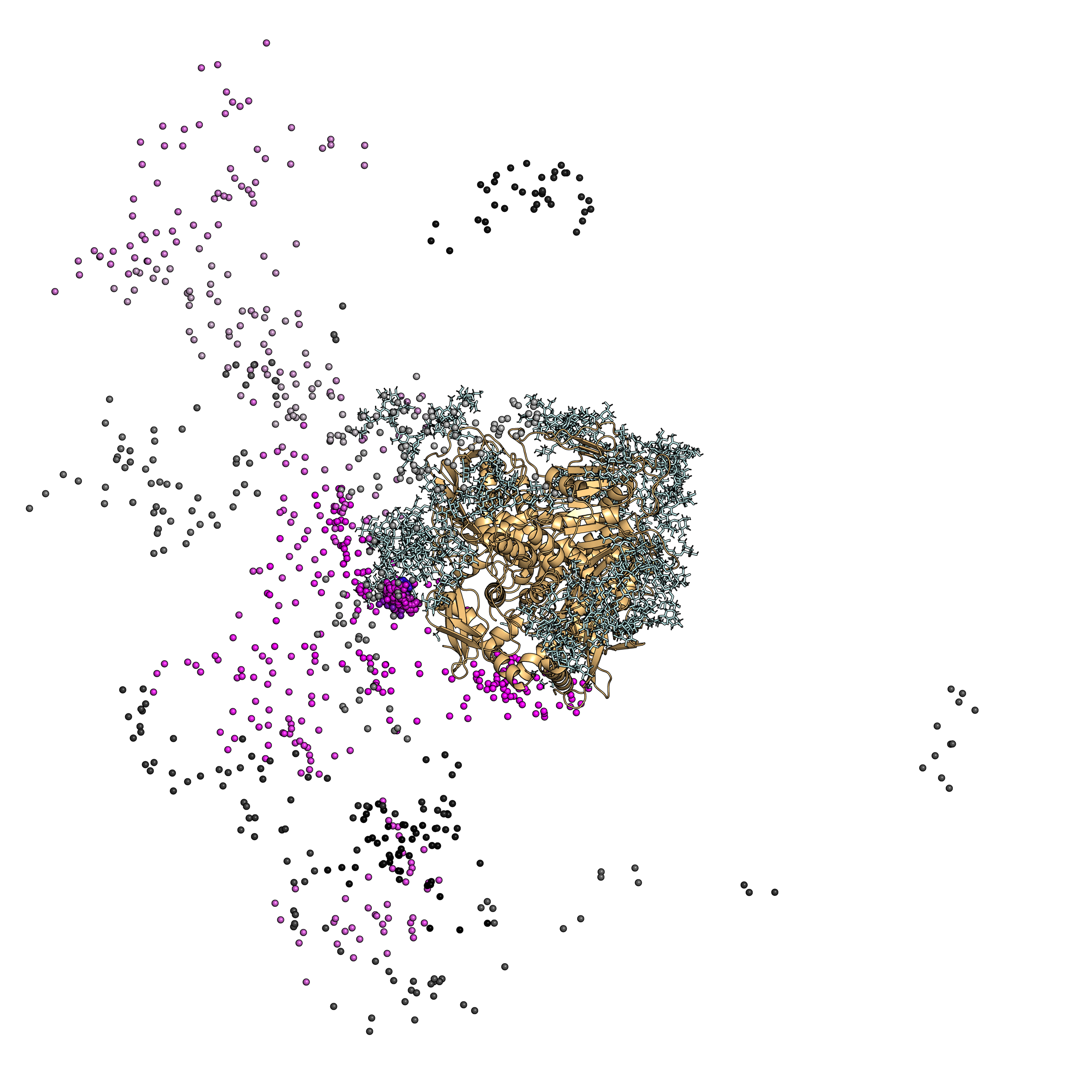

Supplement: Supplementary file 15 — Appendix Figure Source Data [file 44321_2026_387_MOESM15_ESM.zip › Appendix Fig. S5/Fig. S5B/Replica_5.png]

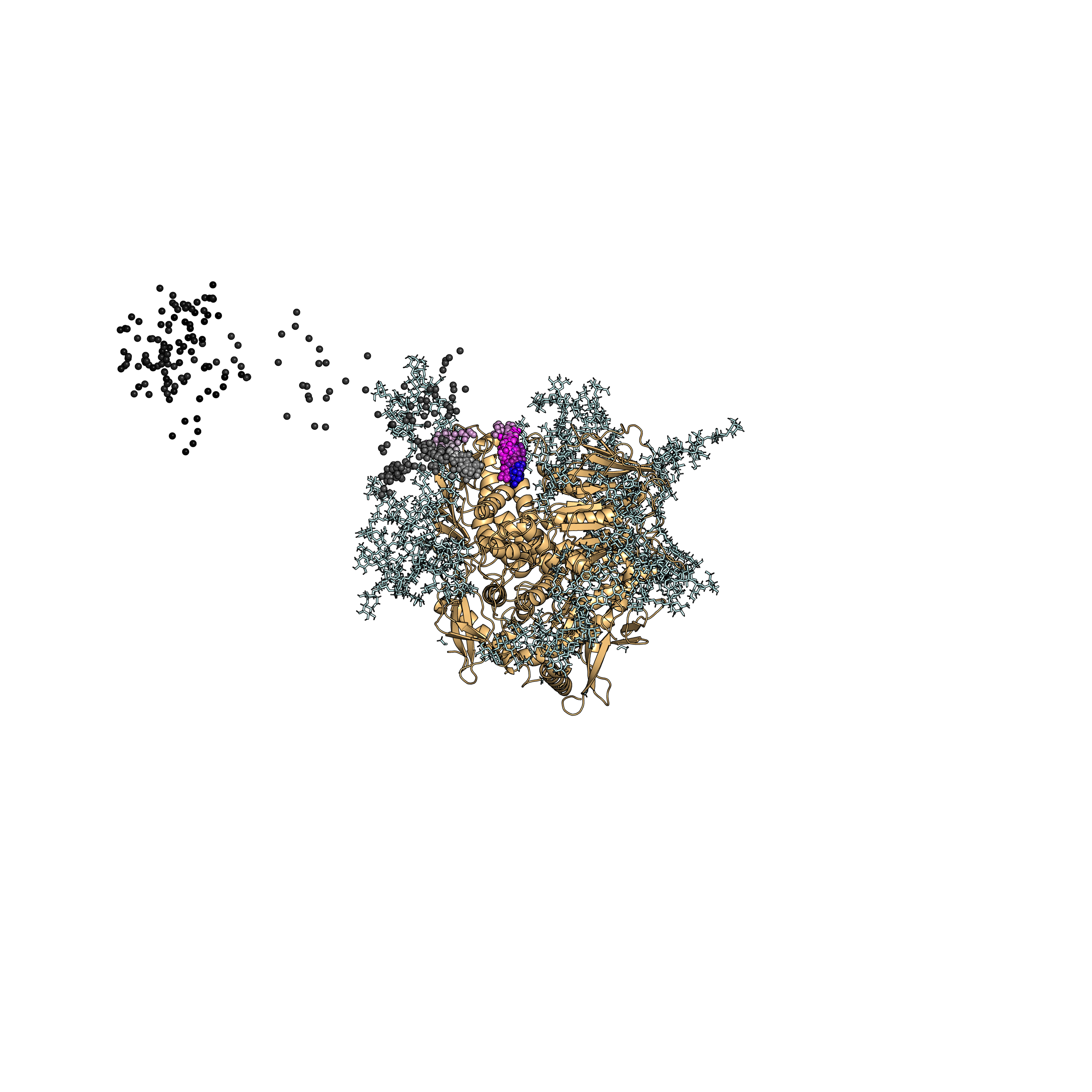

Supplement: Supplementary file 15 — Appendix Figure Source Data [file 44321_2026_387_MOESM15_ESM.zip › Appendix Fig. S5/Fig. S5B/Replica_4.png]

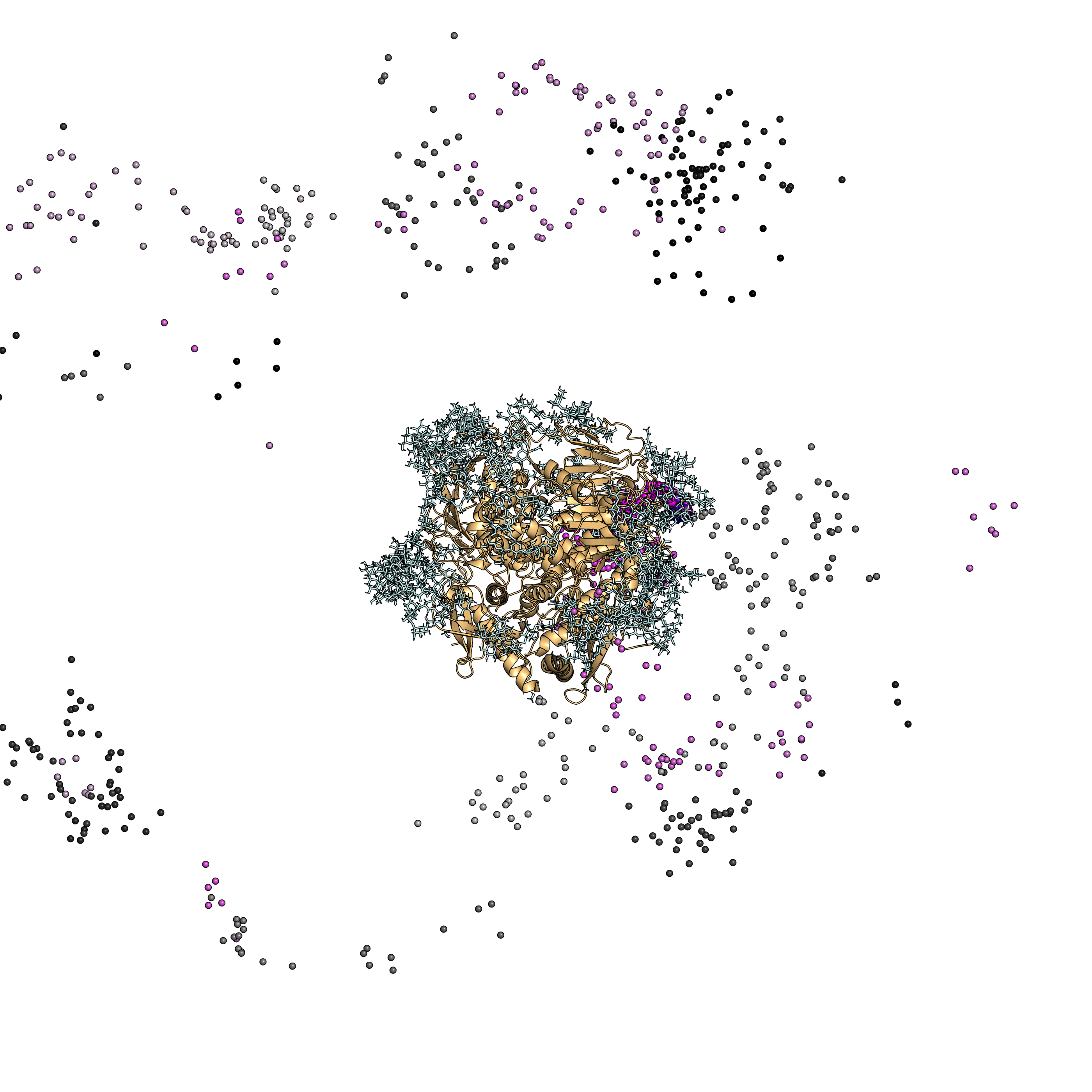

Supplement: Supplementary file 15 — Appendix Figure Source Data [file 44321_2026_387_MOESM15_ESM.zip › Appendix Fig. S5/Fig. S5B/Replica_1.png]

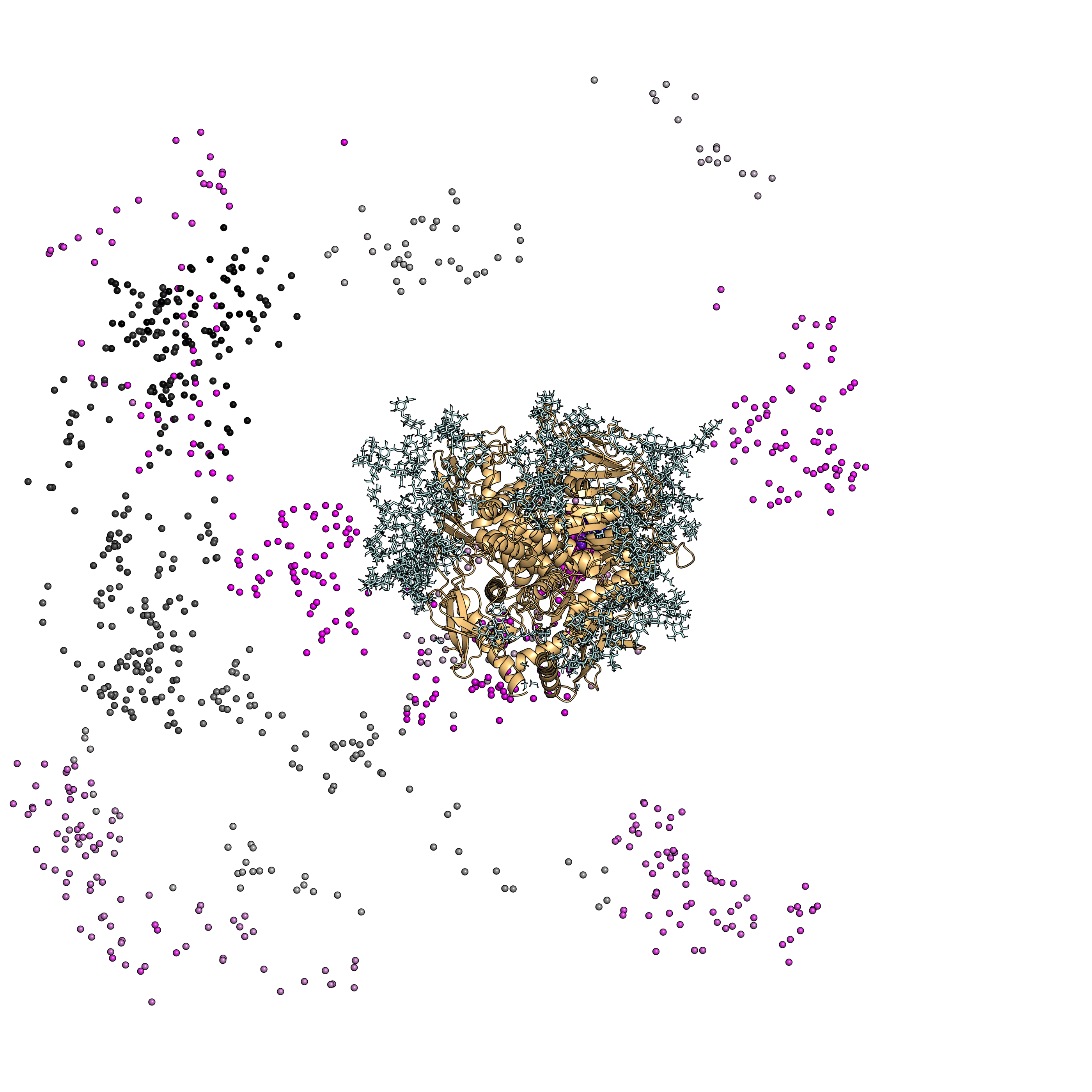

Supplement: Supplementary file 15 — Appendix Figure Source Data [file 44321_2026_387_MOESM15_ESM.zip › Appendix Fig. S5/Fig. S5B/Replica_3.png]

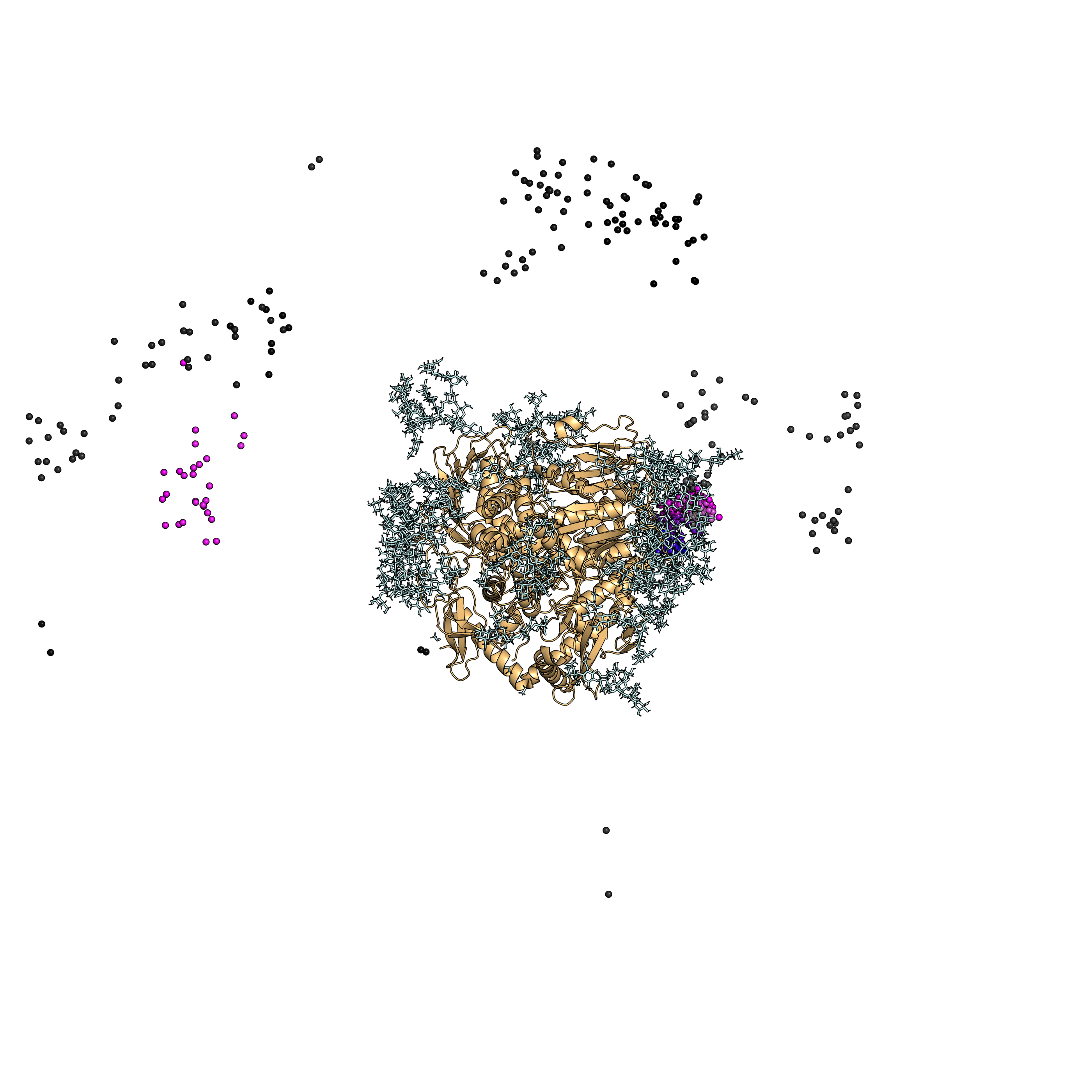

Supplement: Supplementary file 15 — Appendix Figure Source Data [file 44321_2026_387_MOESM15_ESM.zip › Appendix Fig. S5/Fig. S5B/Replica_2.png]

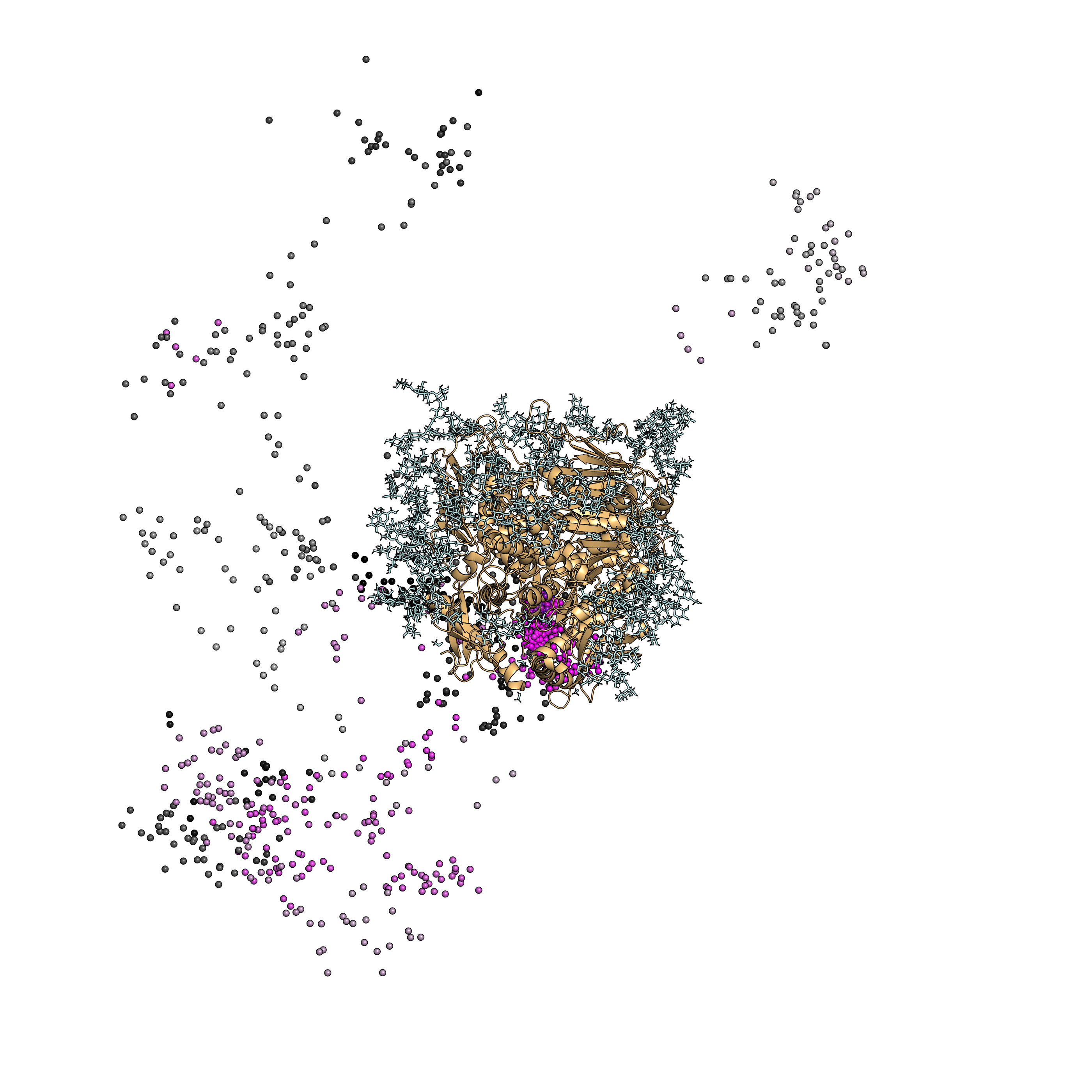

Supplement: Supplementary file 15 — Appendix Figure Source Data [file 44321_2026_387_MOESM15_ESM.zip › Appendix Fig. S5/Fig. S5B/Replica_10.png]

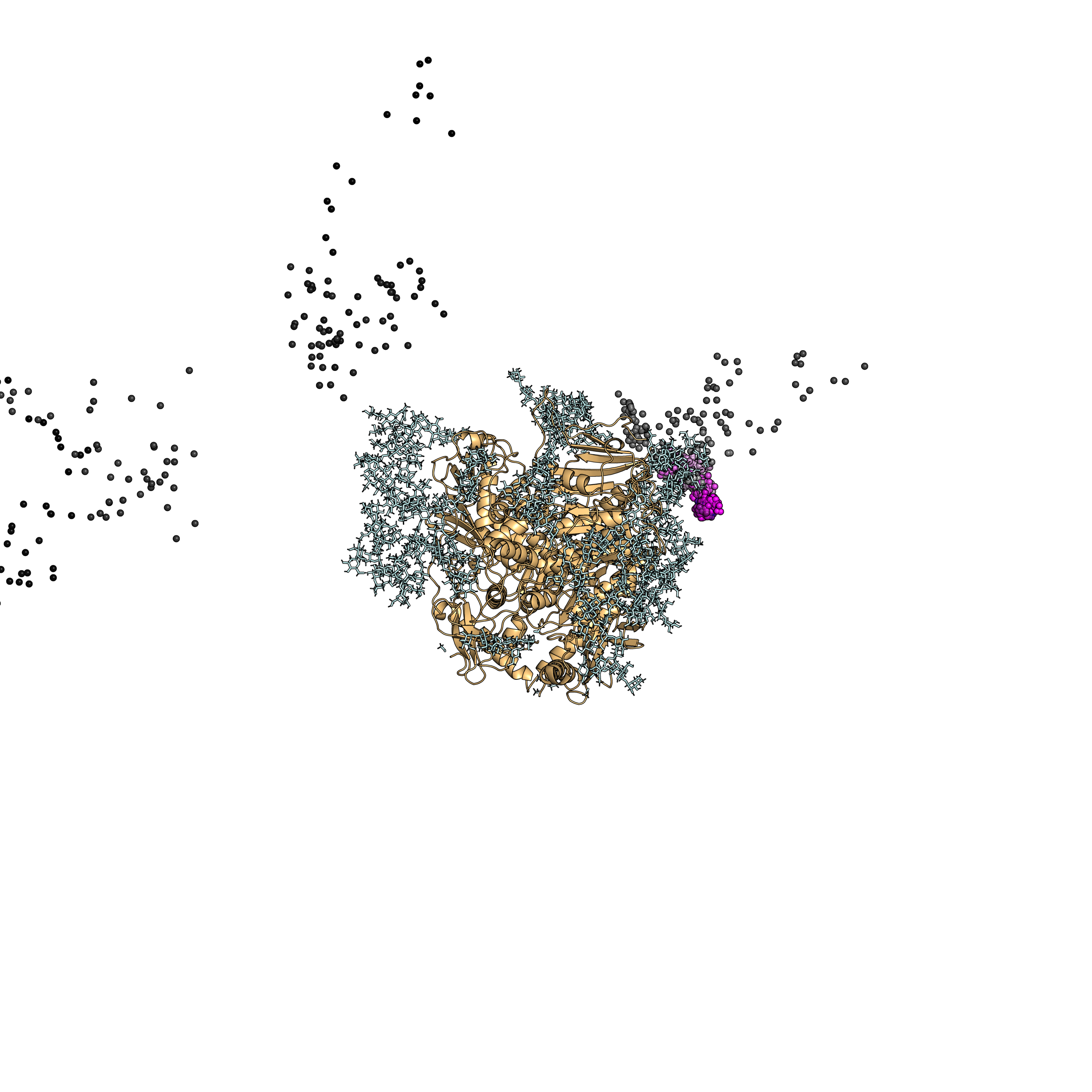

Supplement: Supplementary file 15 — Appendix Figure Source Data [file 44321_2026_387_MOESM15_ESM.zip › Appendix Fig. S5/Fig. S5B/Replica_9.png]

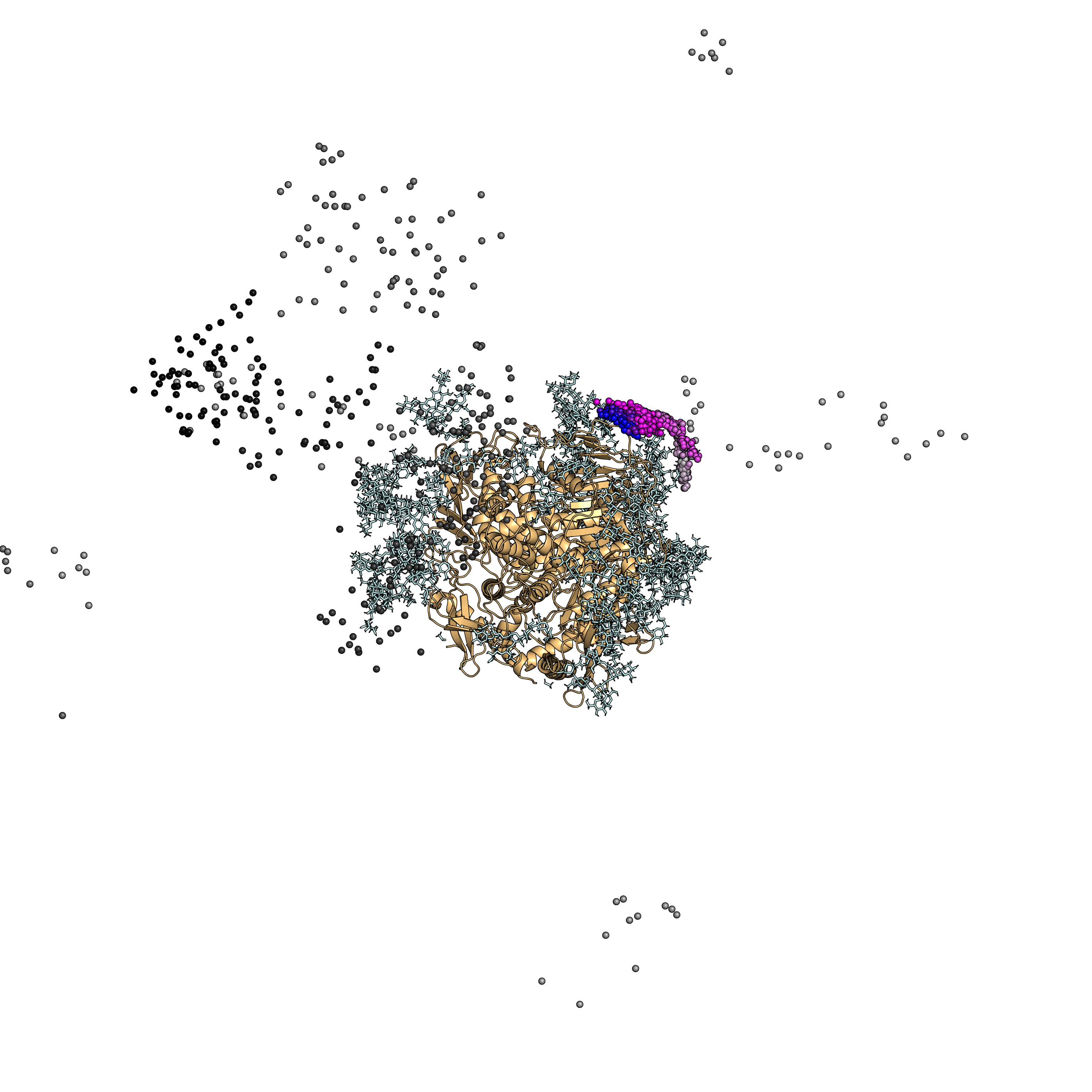

Supplement: Supplementary file 15 — Appendix Figure Source Data [file 44321_2026_387_MOESM15_ESM.zip › Appendix Fig. S5/Fig. S5B/Replica_8.png]

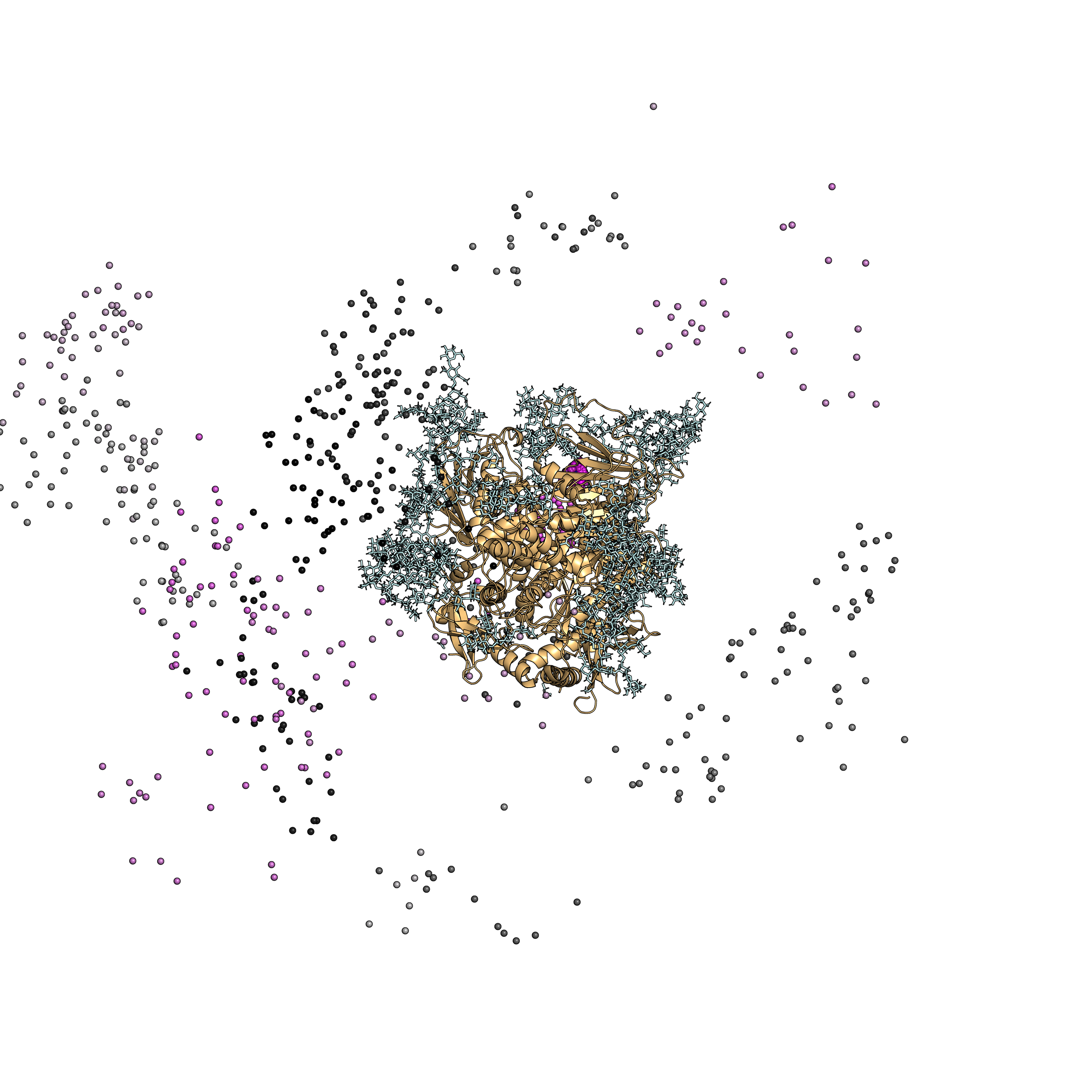

Supplement: Supplementary file 15 — Appendix Figure Source Data [file 44321_2026_387_MOESM15_ESM.zip › Appendix Fig. S5/Fig. S5A/Replica_6.png]

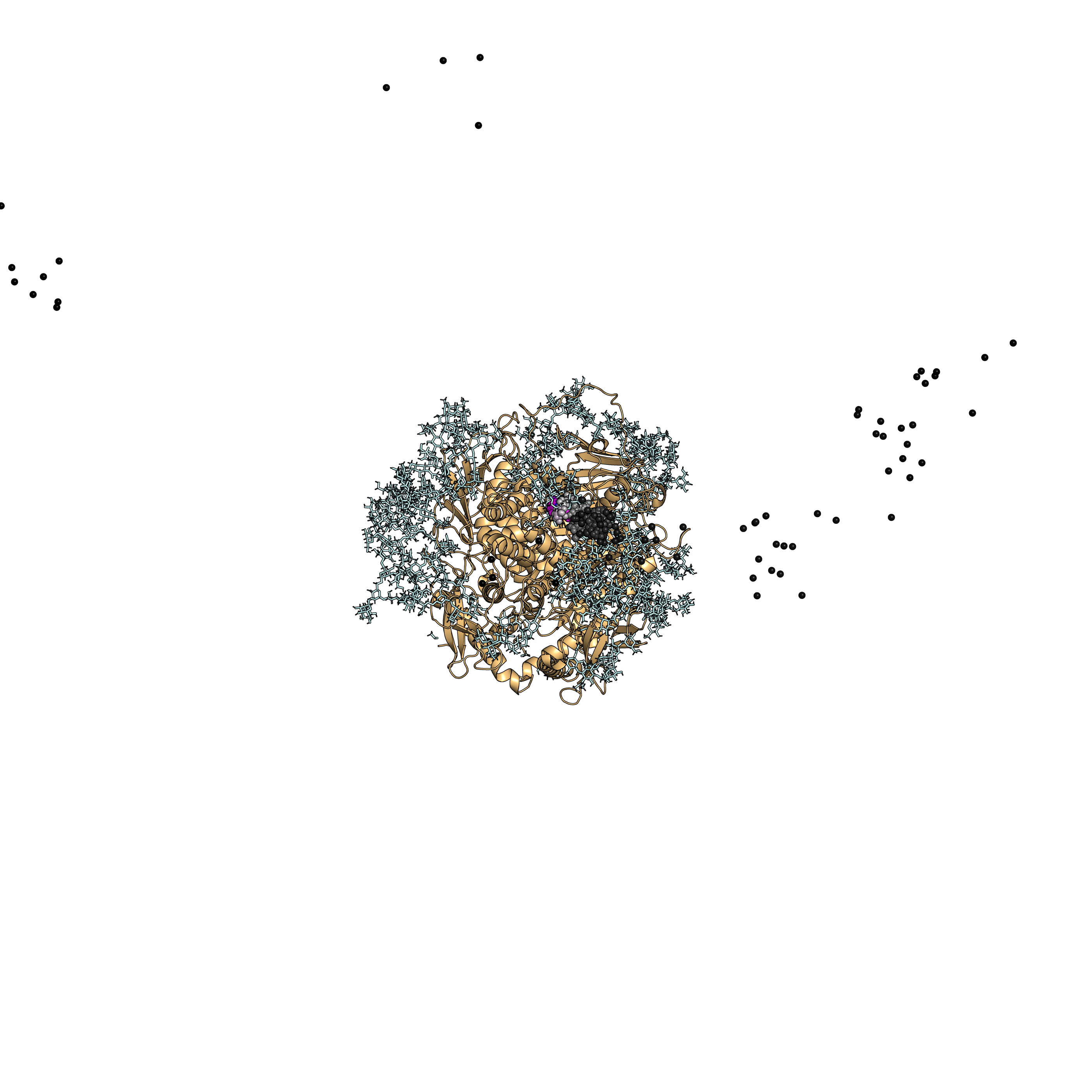

Supplement: Supplementary file 15 — Appendix Figure Source Data [file 44321_2026_387_MOESM15_ESM.zip › Appendix Fig. S5/Fig. S5A/Replica_7.png]

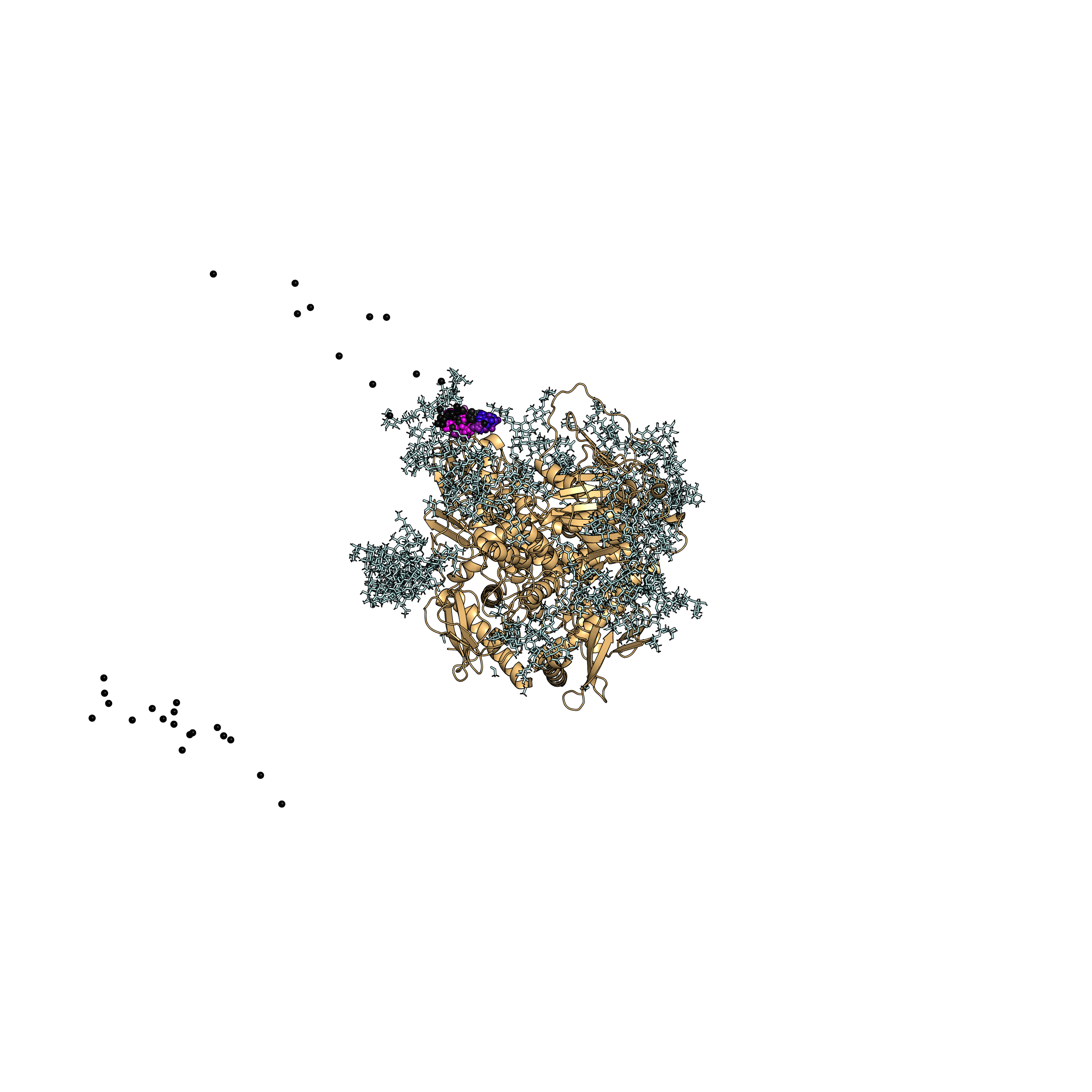

Supplement: Supplementary file 15 — Appendix Figure Source Data [file 44321_2026_387_MOESM15_ESM.zip › Appendix Fig. S5/Fig. S5A/Replica_5.png]

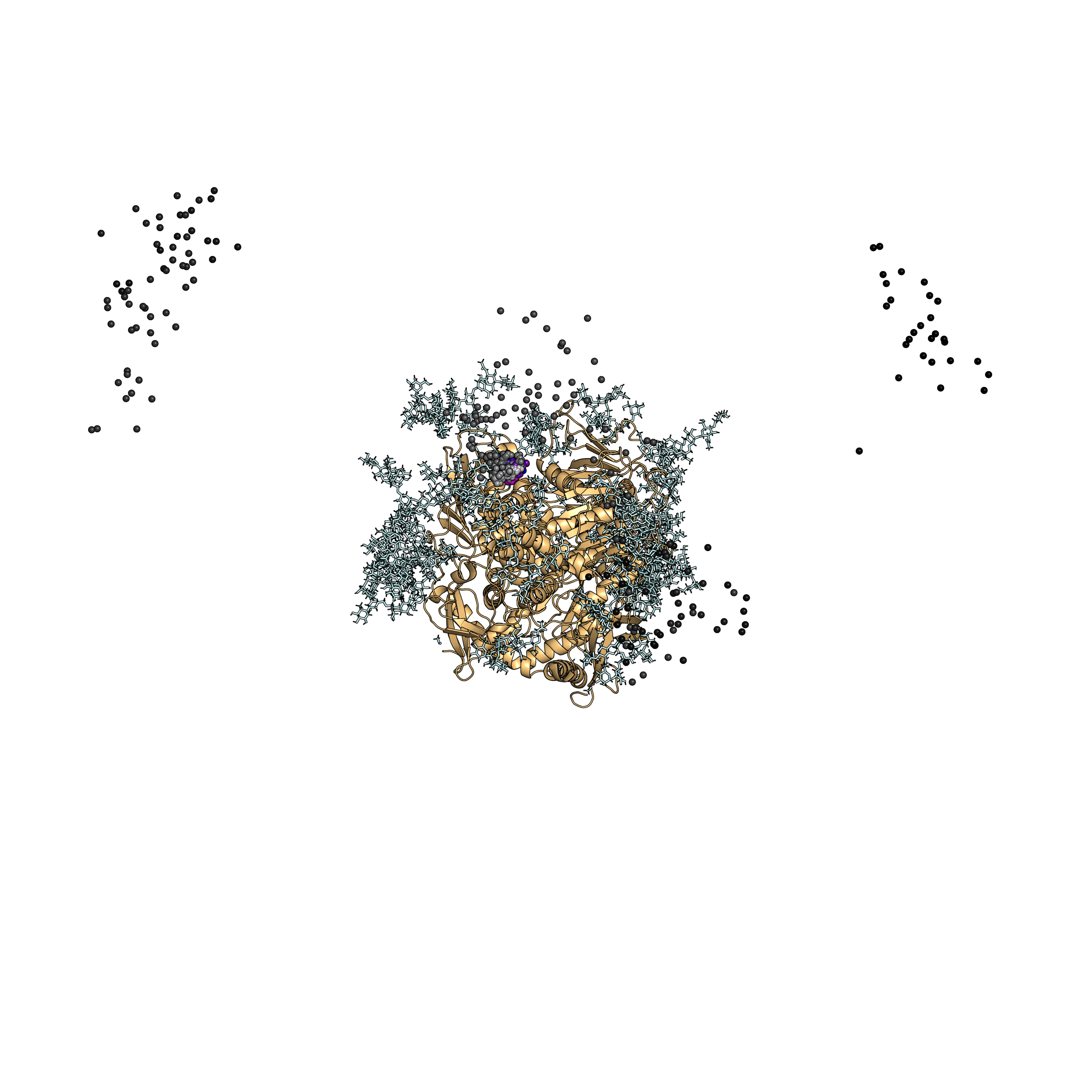

Supplement: Supplementary file 15 — Appendix Figure Source Data [file 44321_2026_387_MOESM15_ESM.zip › Appendix Fig. S5/Fig. S5A/Replica_4.png]

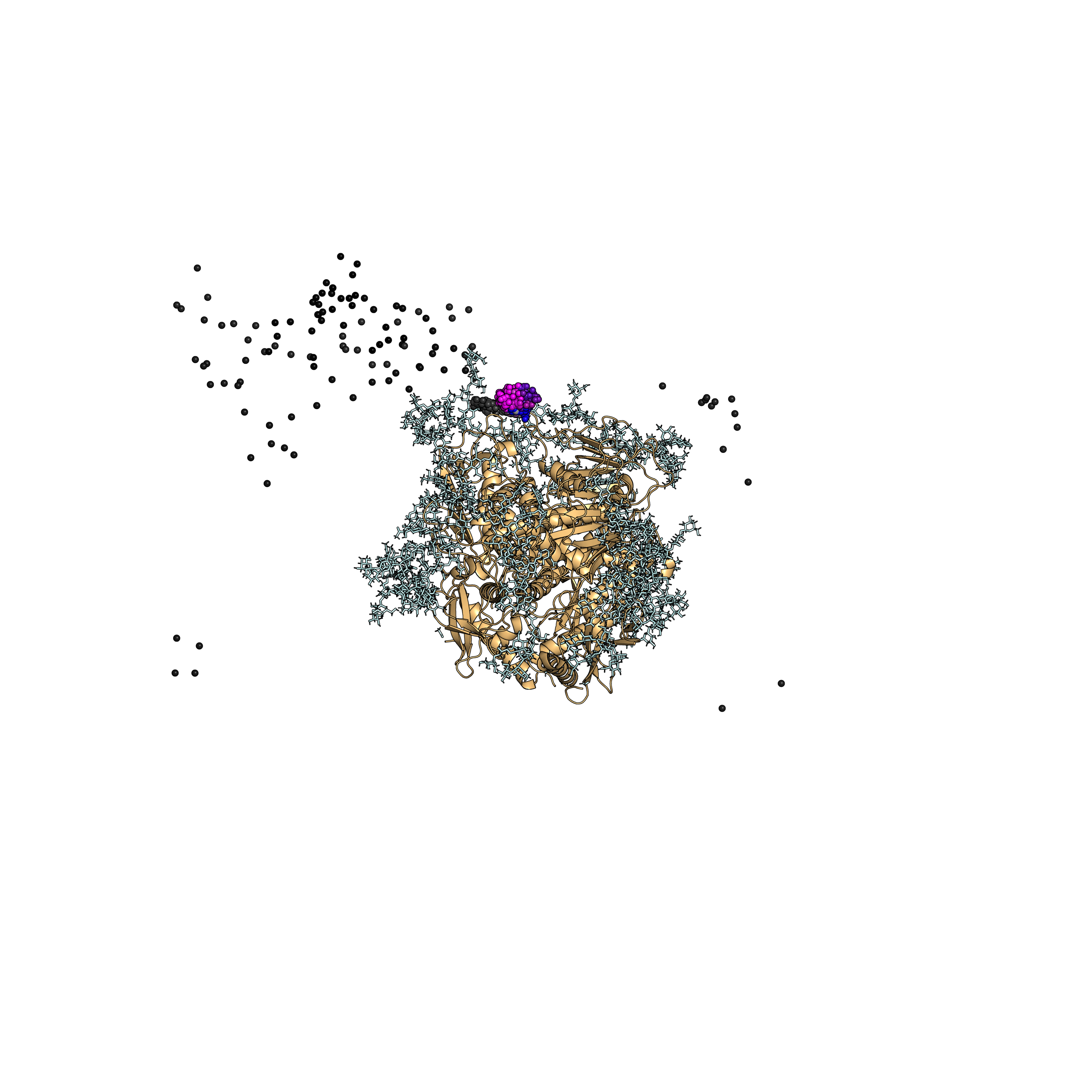

Supplement: Supplementary file 15 — Appendix Figure Source Data [file 44321_2026_387_MOESM15_ESM.zip › Appendix Fig. S5/Fig. S5A/Replica_1.png]

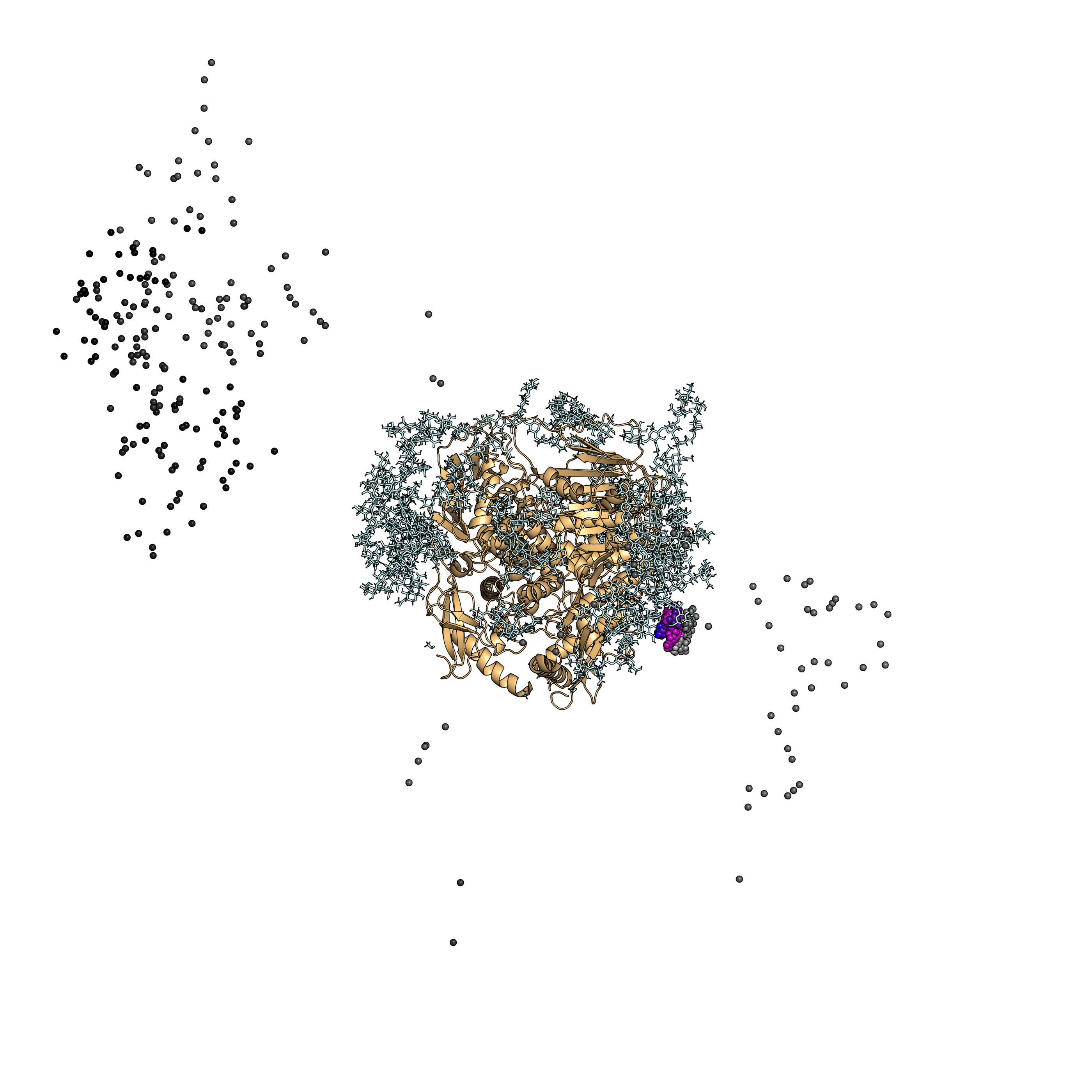

Supplement: Supplementary file 15 — Appendix Figure Source Data [file 44321_2026_387_MOESM15_ESM.zip › Appendix Fig. S5/Fig. S5A/Replica_3.png]

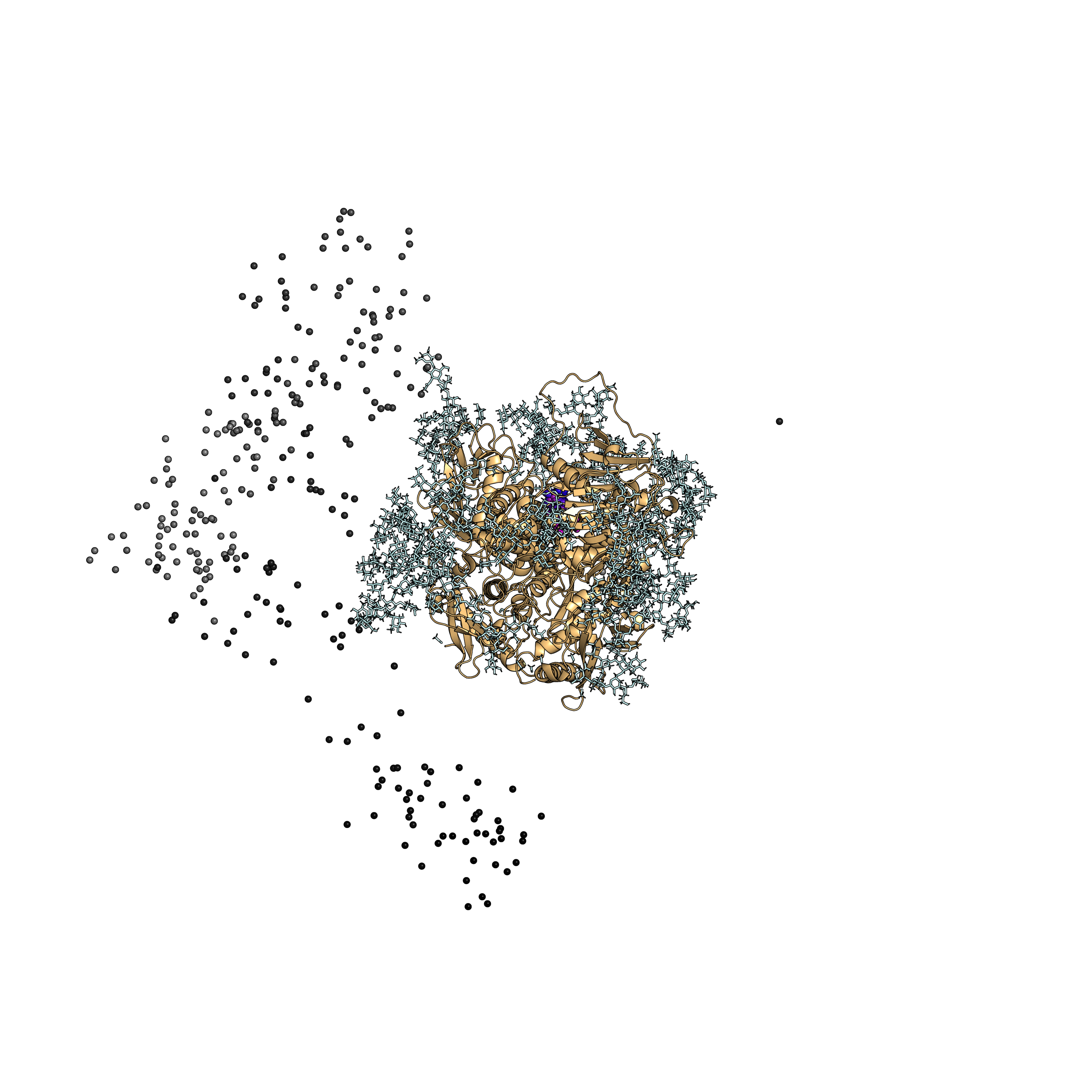

Supplement: Supplementary file 15 — Appendix Figure Source Data [file 44321_2026_387_MOESM15_ESM.zip › Appendix Fig. S5/Fig. S5A/Replica_2.png]

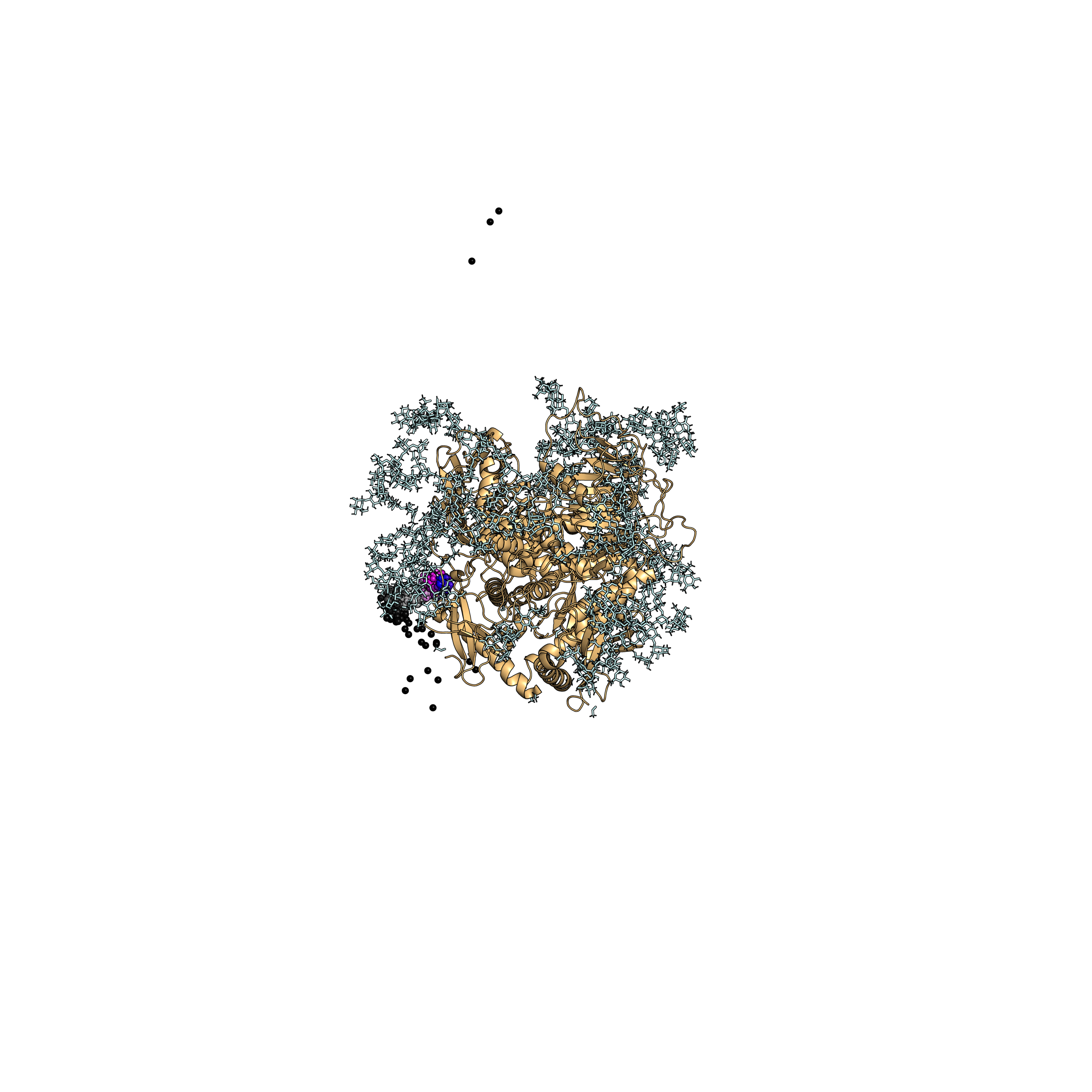

Supplement: Supplementary file 15 — Appendix Figure Source Data [file 44321_2026_387_MOESM15_ESM.zip › Appendix Fig. S5/Fig. S5A/Replica_10.png]

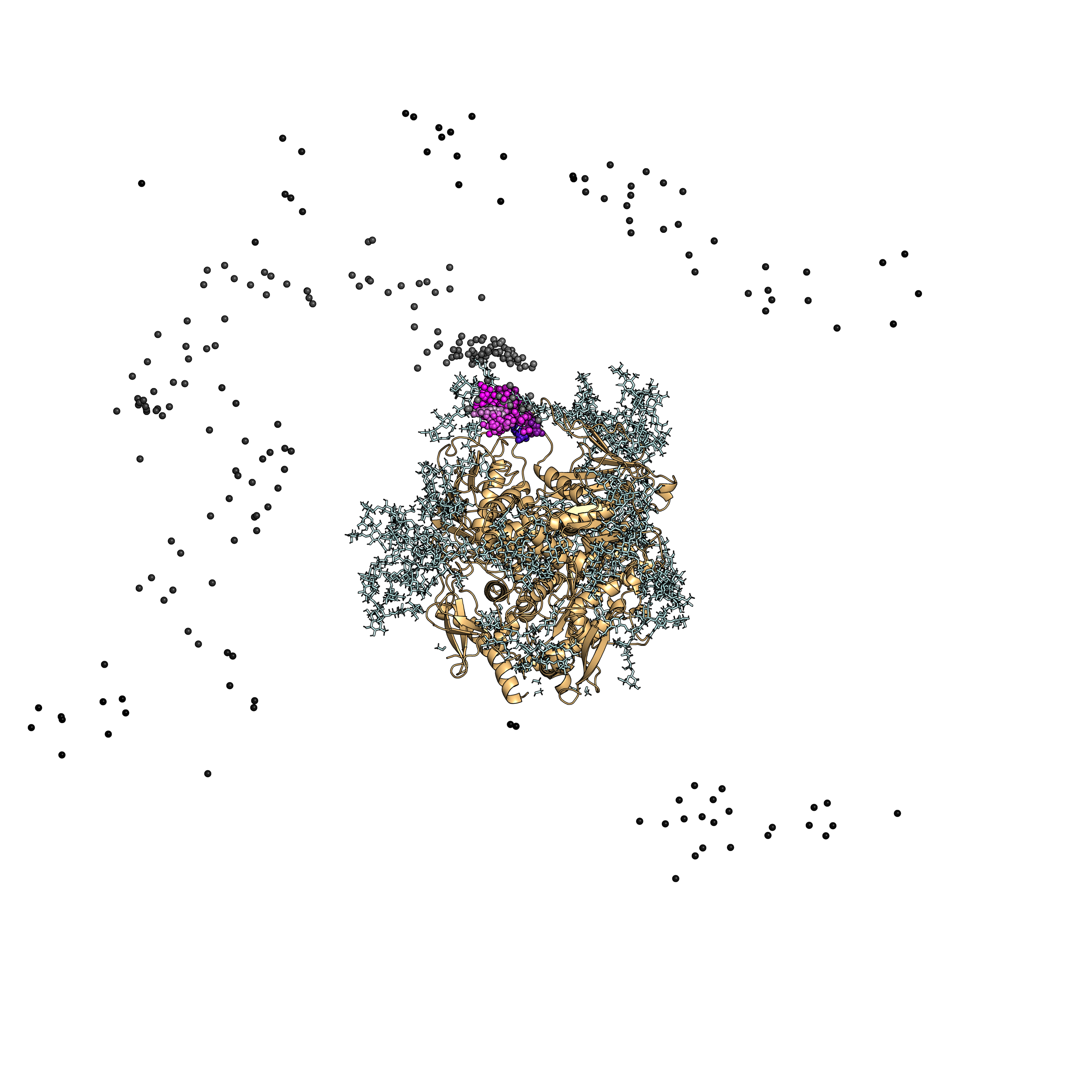

Supplement: Supplementary file 15 — Appendix Figure Source Data [file 44321_2026_387_MOESM15_ESM.zip › Appendix Fig. S5/Fig. S5A/Replica_9.png]

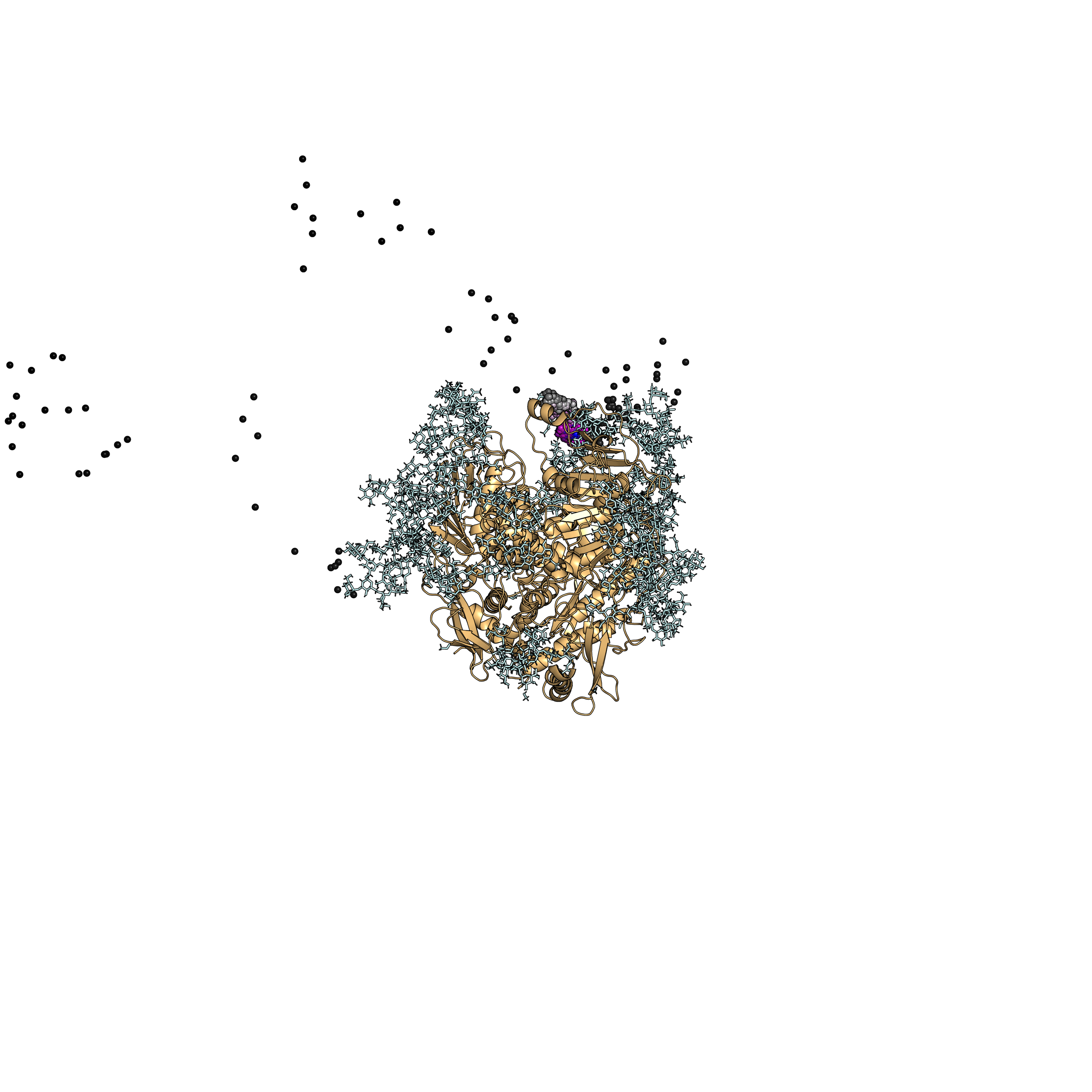

Supplement: Supplementary file 15 — Appendix Figure Source Data [file 44321_2026_387_MOESM15_ESM.zip › Appendix Fig. S5/Fig. S5A/Replica_8.png]

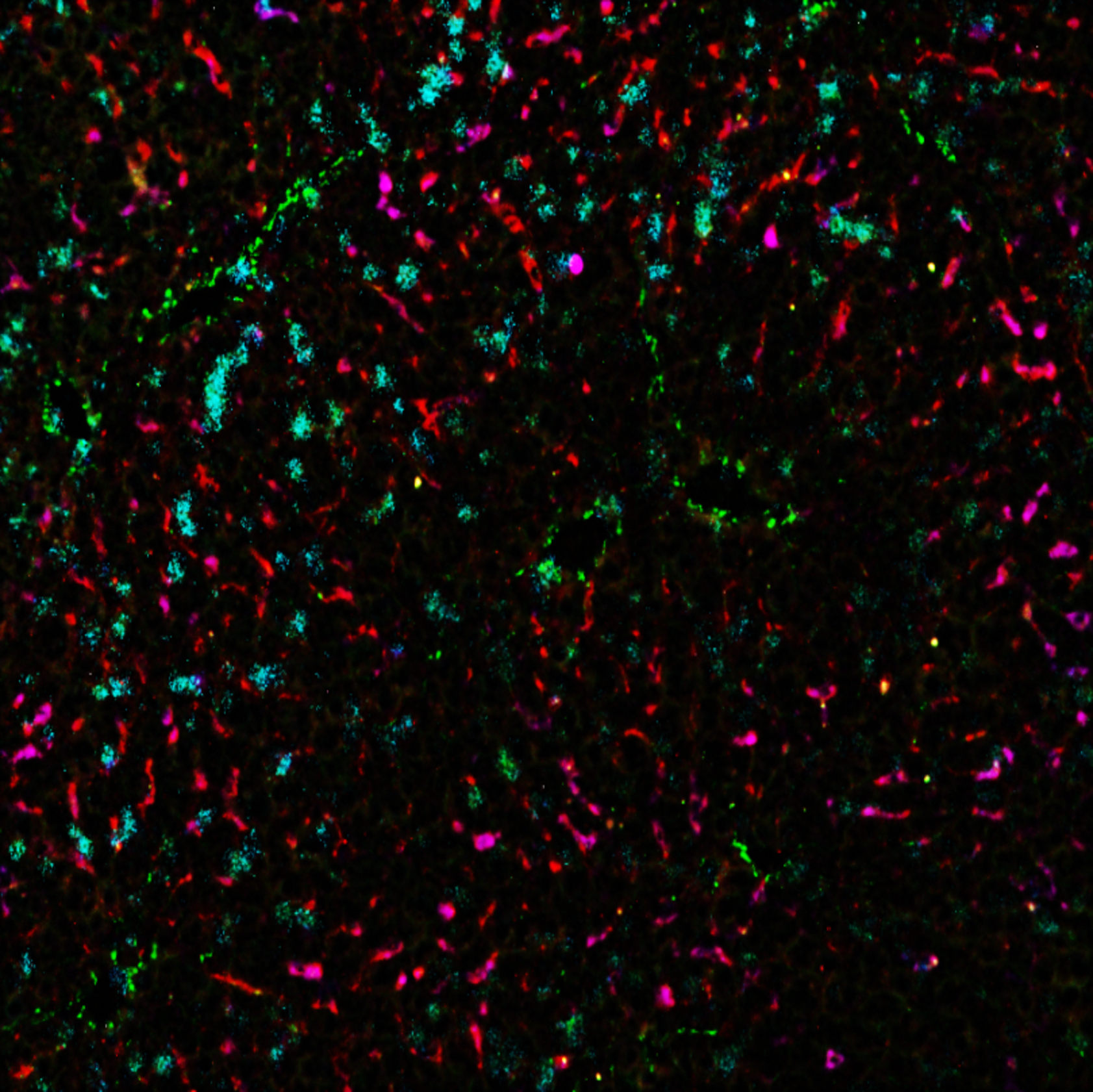

Supplement: Supplementary file 15 — Appendix Figure Source Data [file 44321_2026_387_MOESM15_ESM.zip › Appendix Fig. S6/Fig. S6A/Fig.S6A vehicle merged.tif]

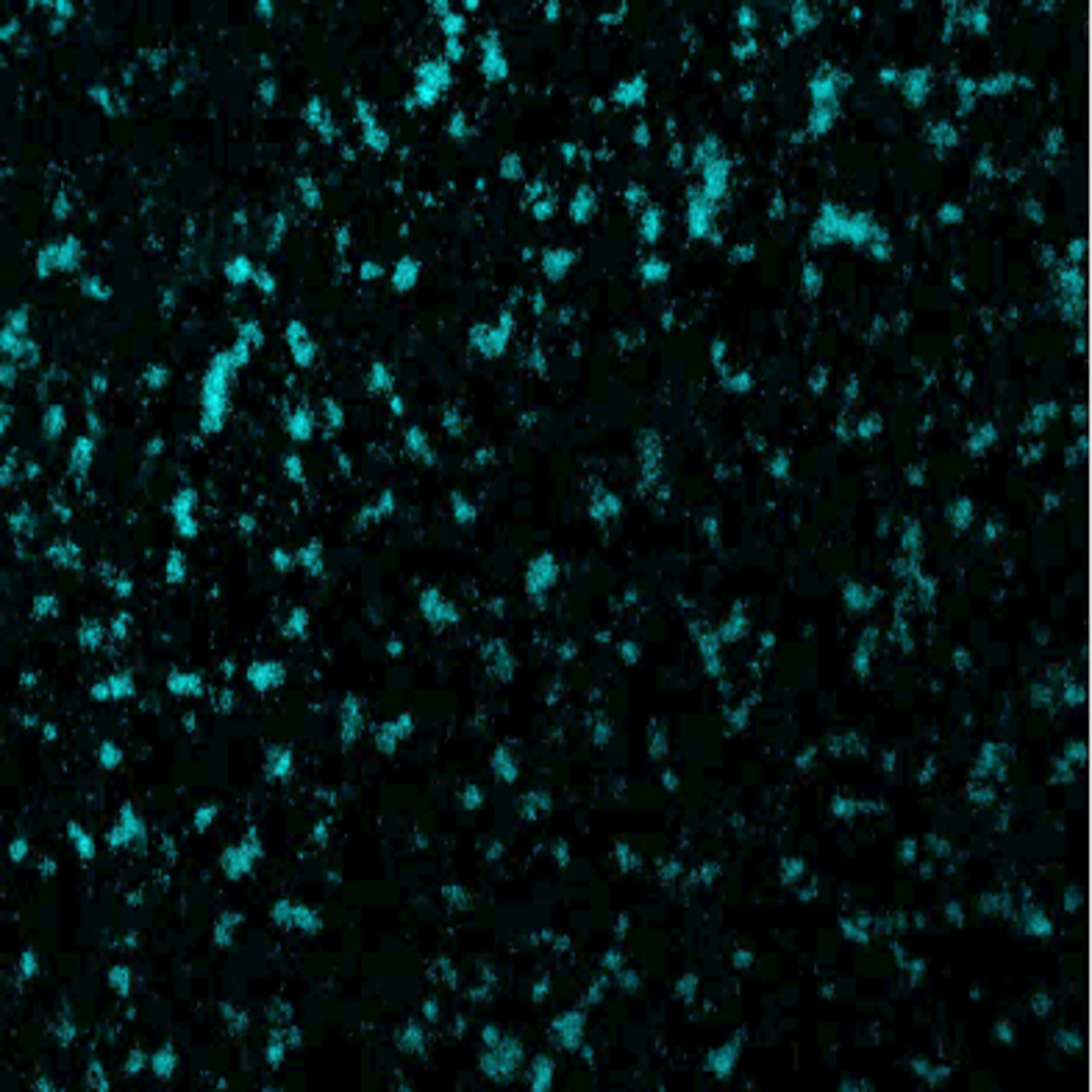

Supplement: Supplementary file 15 — Appendix Figure Source Data [file 44321_2026_387_MOESM15_ESM.zip › Appendix Fig. S6/Fig. S6A/Fig.S6A vehicle DAPI.tif]

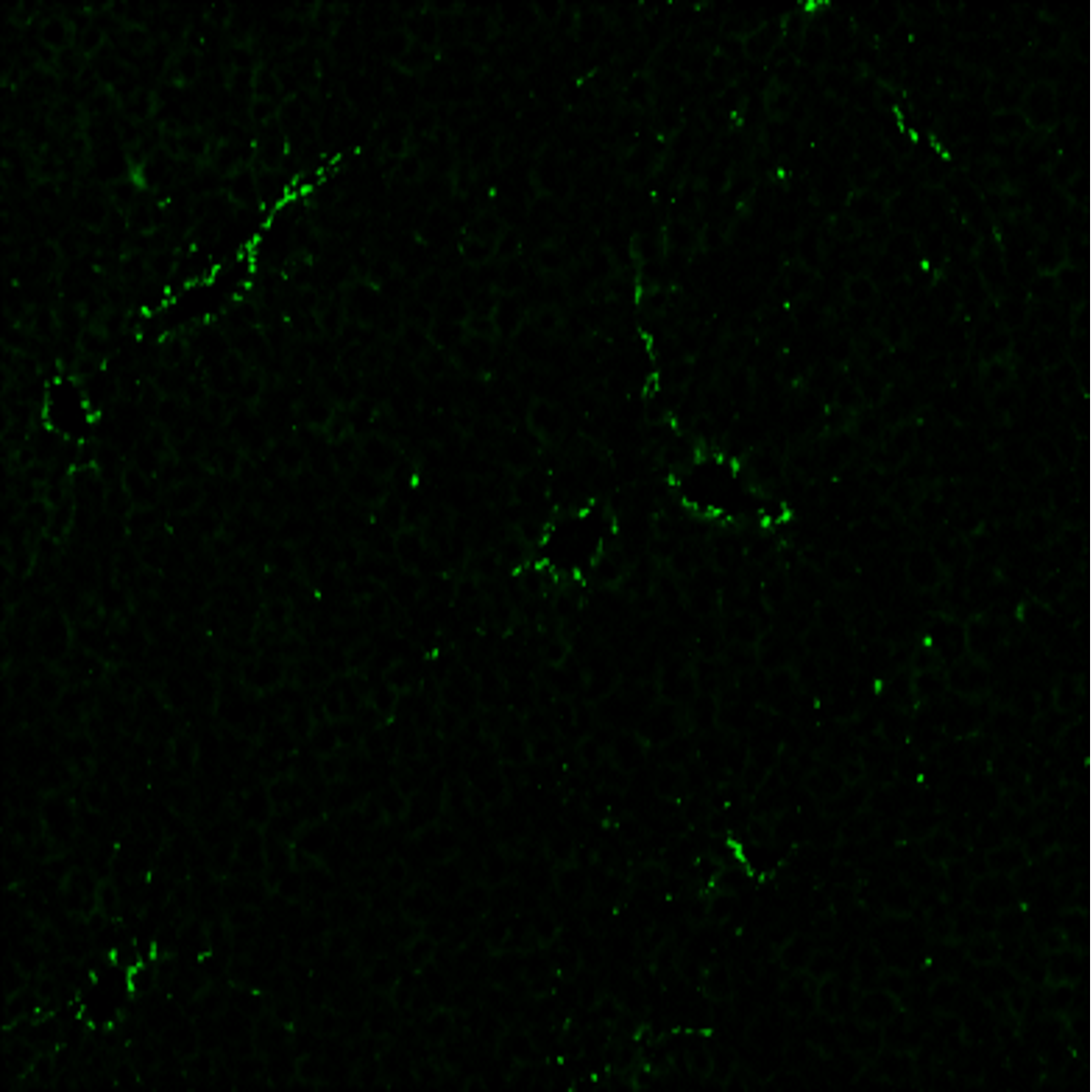

Supplement: Supplementary file 15 — Appendix Figure Source Data [file 44321_2026_387_MOESM15_ESM.zip › Appendix Fig. S6/Fig. S6A/Fig.S6A vehicle cd31.tif]

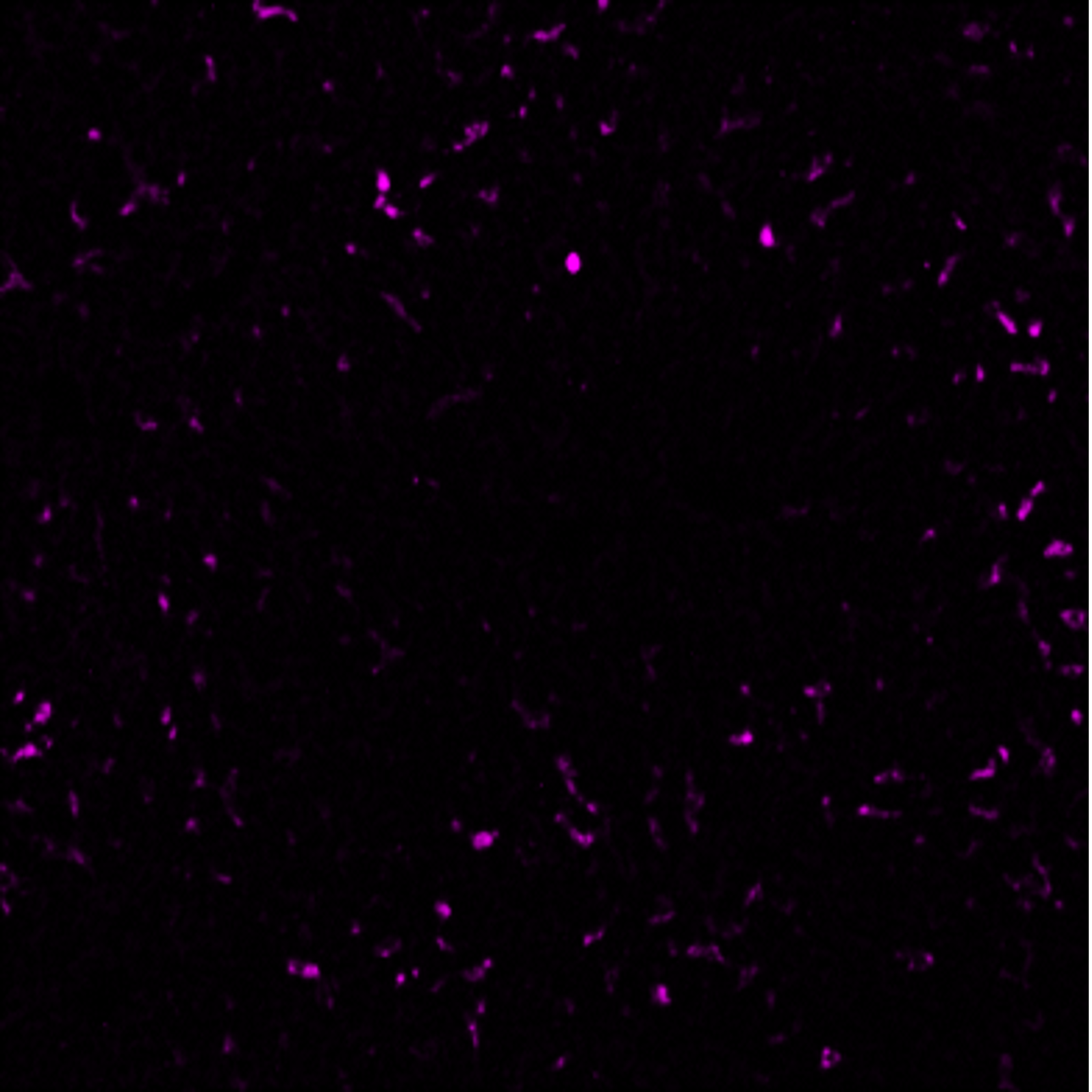

Supplement: Supplementary file 15 — Appendix Figure Source Data [file 44321_2026_387_MOESM15_ESM.zip › Appendix Fig. S6/Fig. S6A/Fig.S6A vehicle f480.tif]

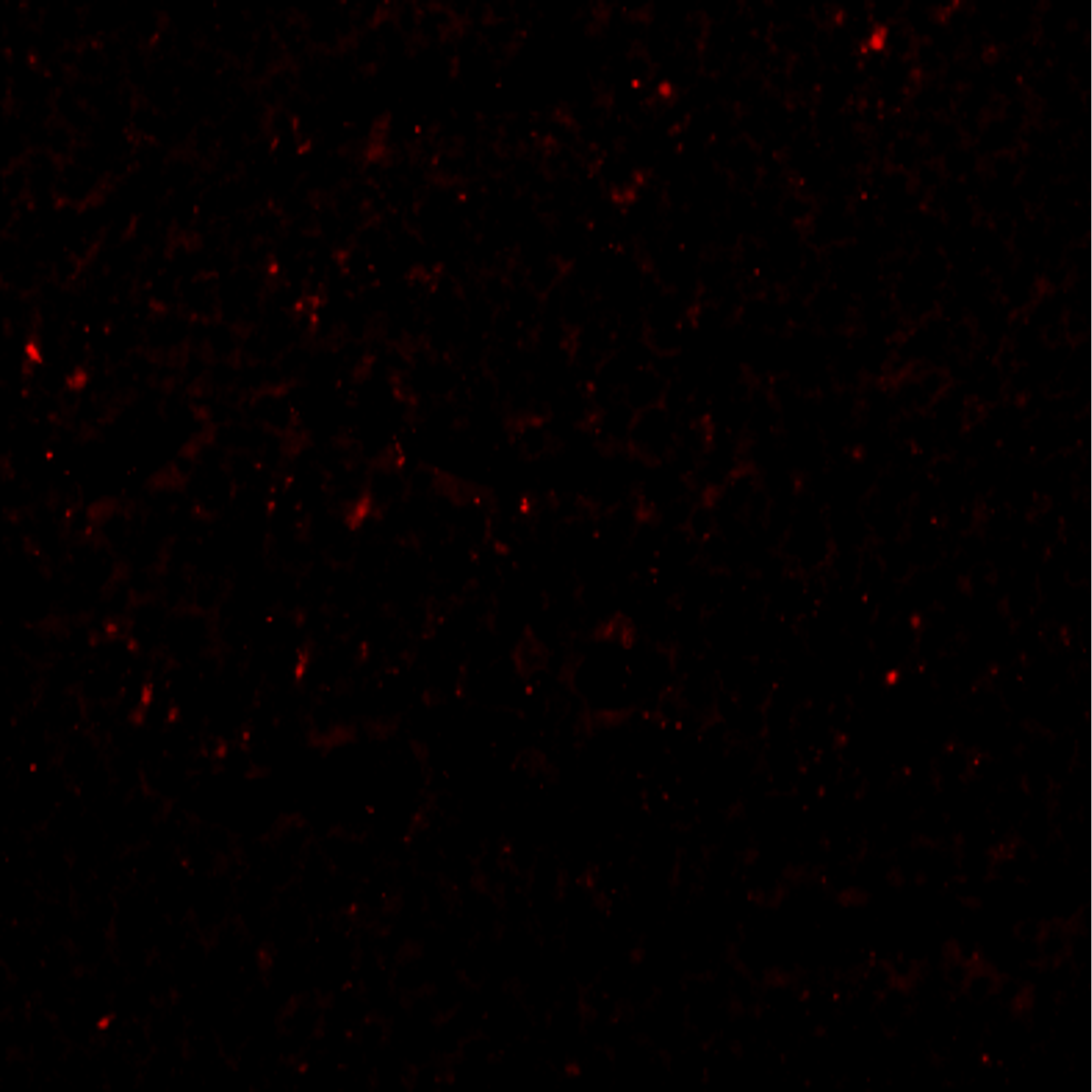

Supplement: Supplementary file 15 — Appendix Figure Source Data [file 44321_2026_387_MOESM15_ESM.zip › Appendix Fig. S6/Fig. S6A/Fig.S6A ds lcmv np.tif]

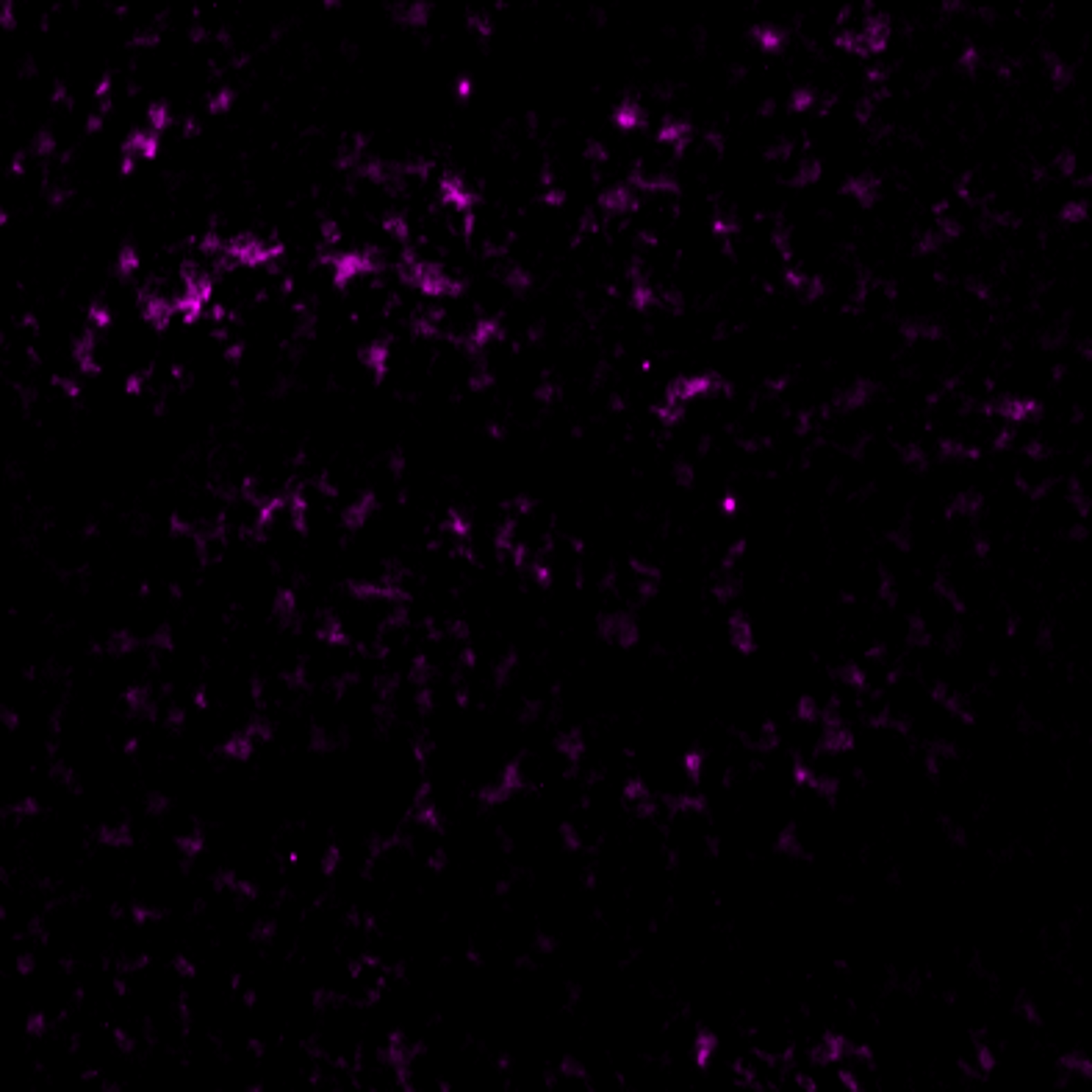

Supplement: Supplementary file 15 — Appendix Figure Source Data [file 44321_2026_387_MOESM15_ESM.zip › Appendix Fig. S6/Fig. S6A/Fig.S6A ds f480.tif]

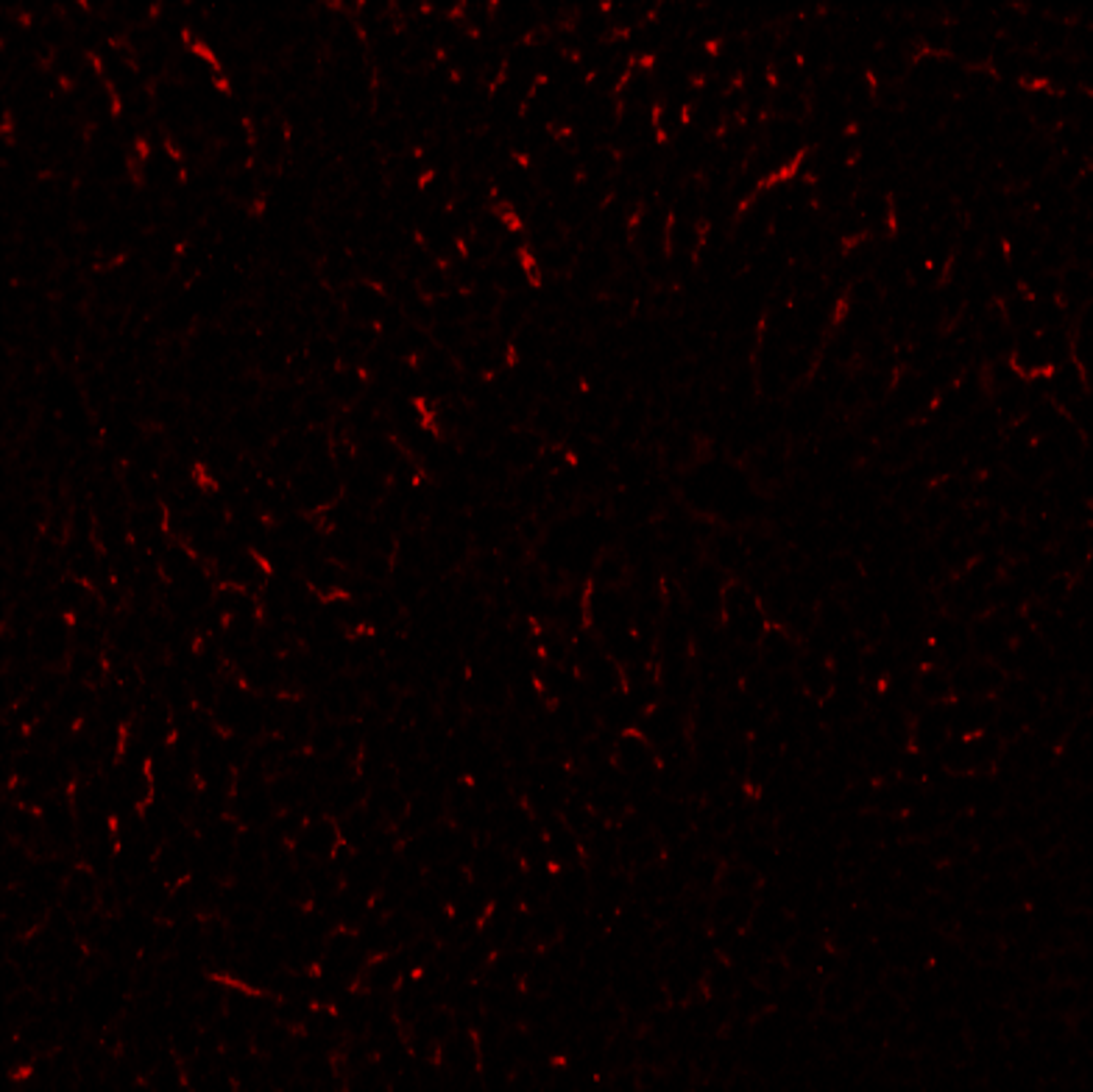

Supplement: Supplementary file 15 — Appendix Figure Source Data [file 44321_2026_387_MOESM15_ESM.zip › Appendix Fig. S6/Fig. S6A/Fig.S6A vehicle lcmv np.tif]

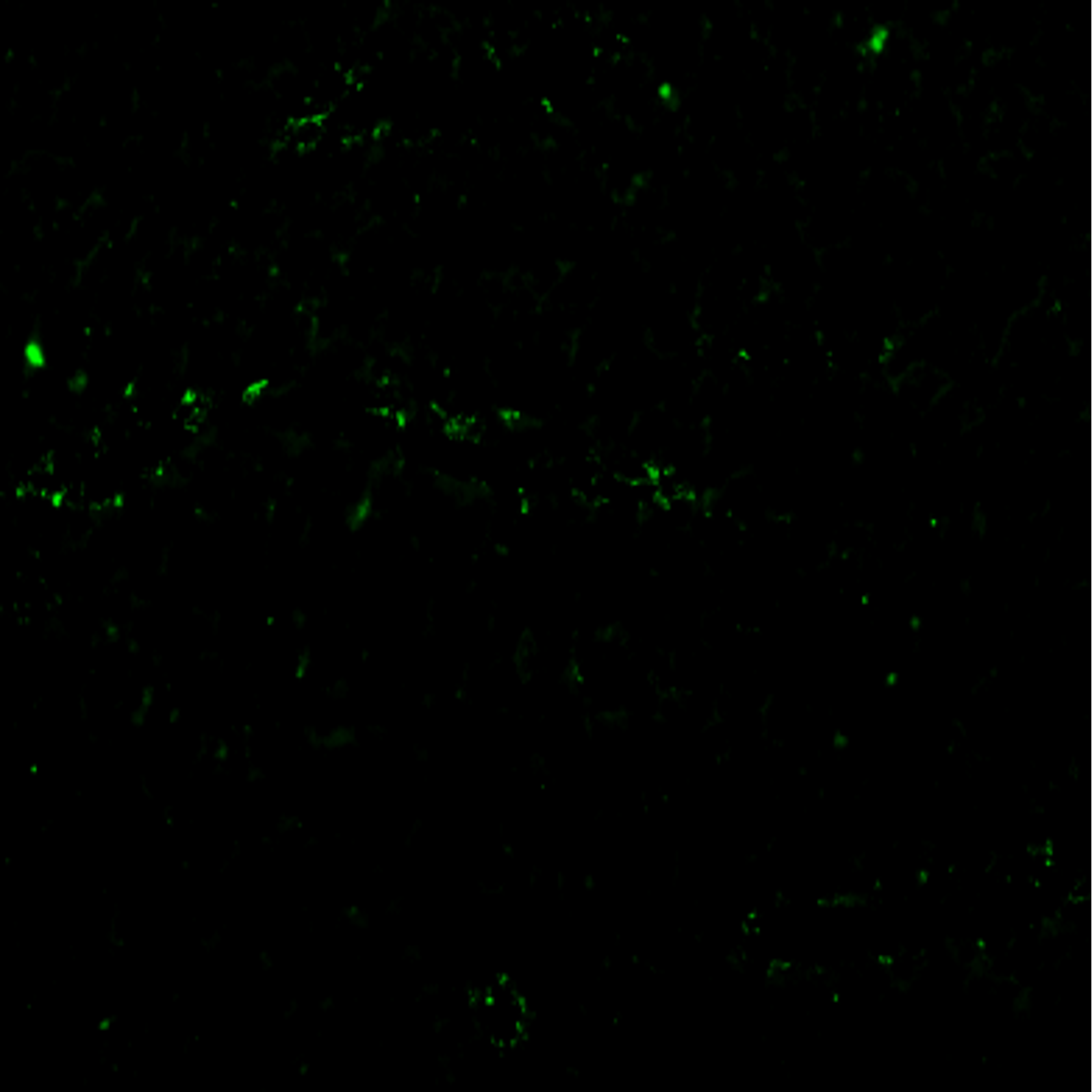

Supplement: Supplementary file 15 — Appendix Figure Source Data [file 44321_2026_387_MOESM15_ESM.zip › Appendix Fig. S6/Fig. S6A/Fig.S6A ds cd31.tif]

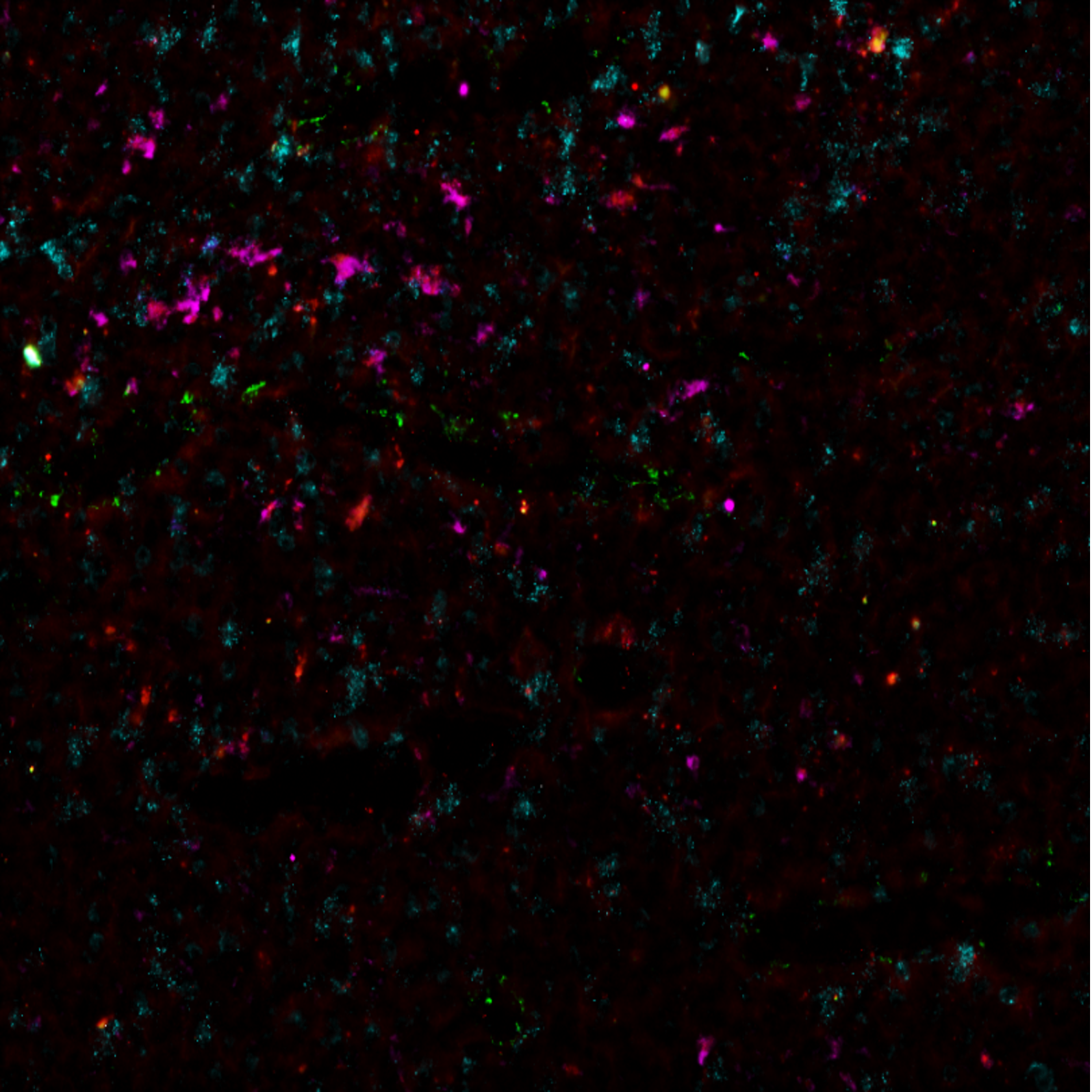

Supplement: Supplementary file 15 — Appendix Figure Source Data [file 44321_2026_387_MOESM15_ESM.zip › Appendix Fig. S6/Fig. S6A/Fig.S6A ds merged.tif]

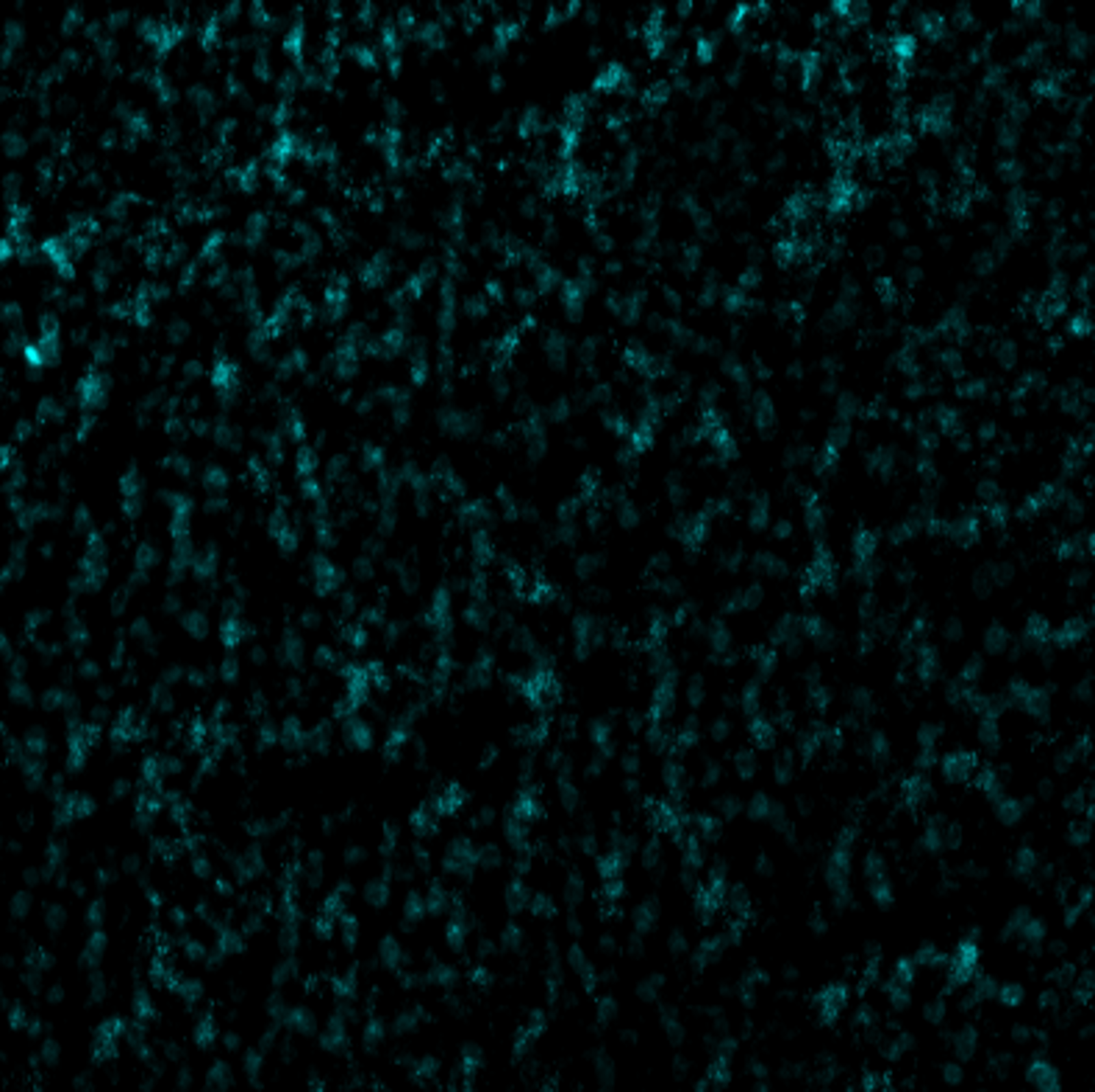

Supplement: Supplementary file 15 — Appendix Figure Source Data [file 44321_2026_387_MOESM15_ESM.zip › Appendix Fig. S6/Fig. S6A/Fig.S6A ds DAPI.tif]
