# Supplementary material for: Sulfated glycosaminoglycans inhibit LCMV entry and modulate antiviral immunity and pathology
Source: EMBO Mol Med. 2026 Feb 23;18(4):1235–64. doi: 10.1038/s44321-026-00387-8 (PMC13083911; doi:10.1038/s44321-026-00387-8)
Supplement: Supplementary file 5 — Source data Fig. 3 [file 44321_2026_387_MOESM5_ESM.zip › Fig. 3/Fig. 3C/Fig. 3C all images.pdf]

Analysis 2022

Dextran, 1 min.

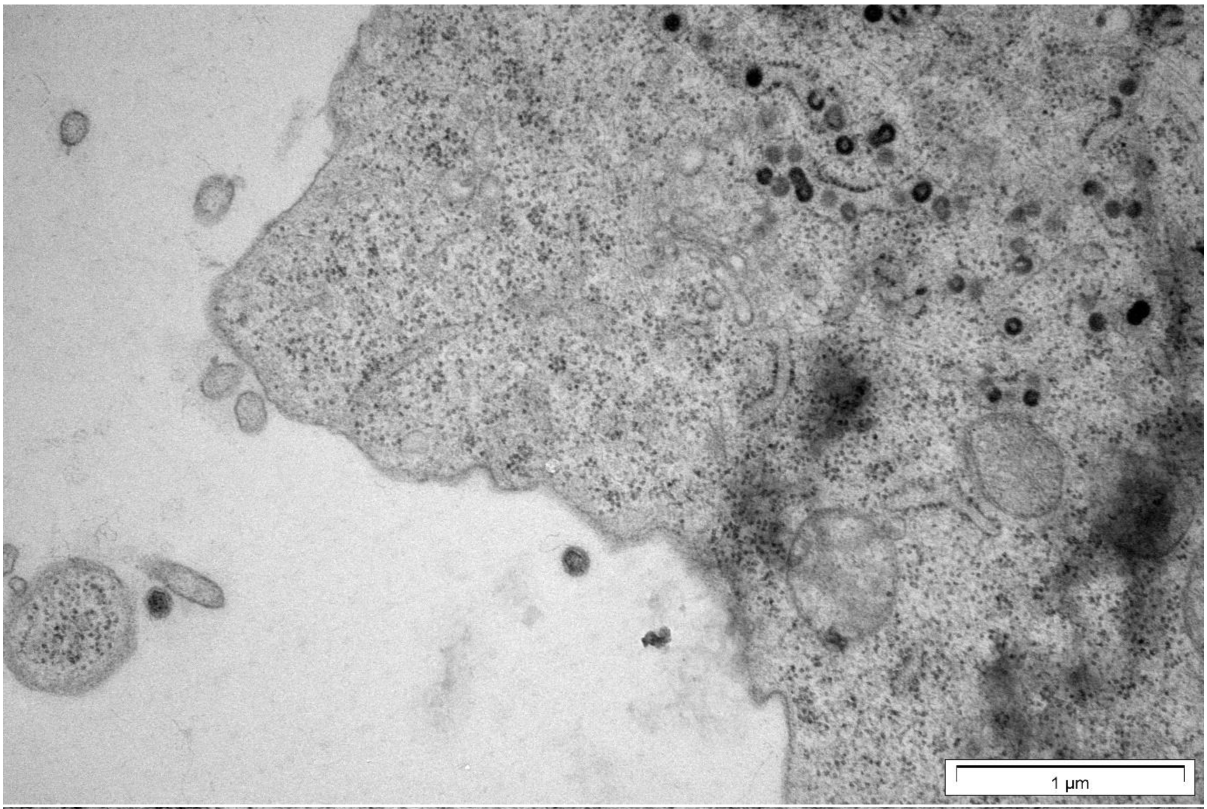

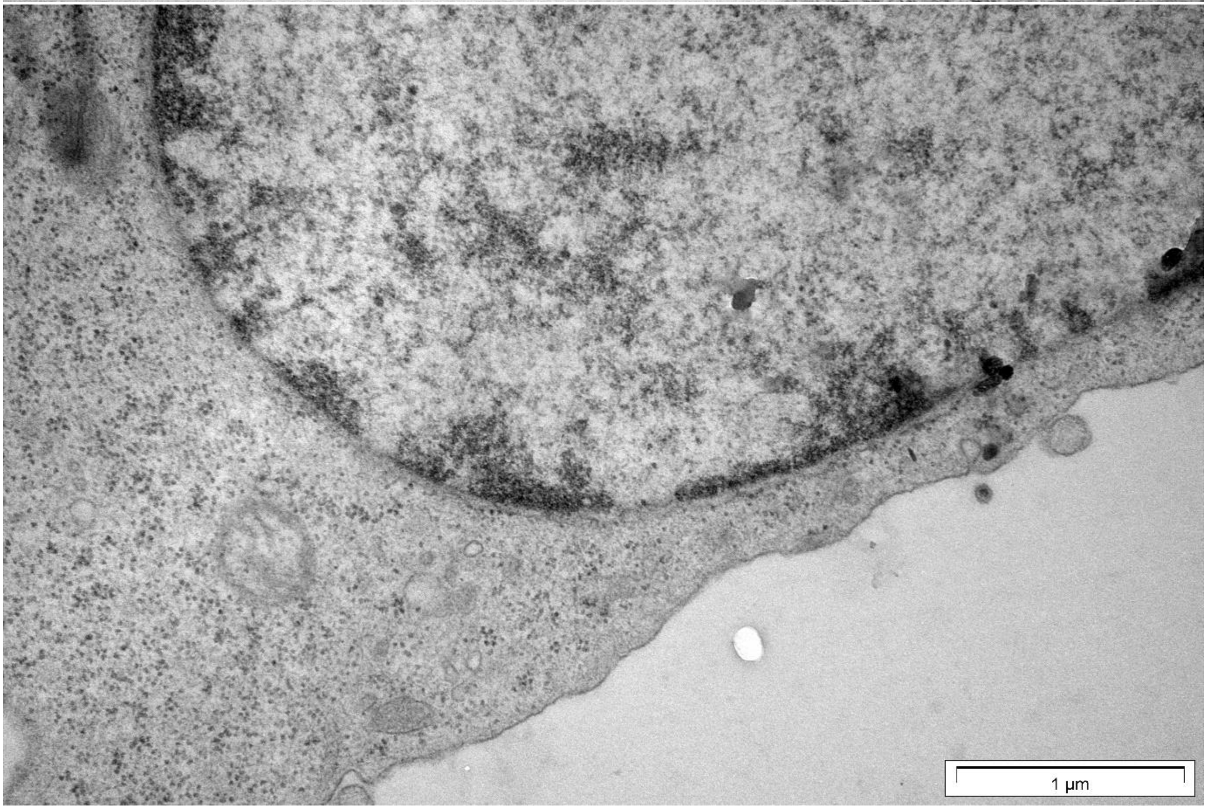

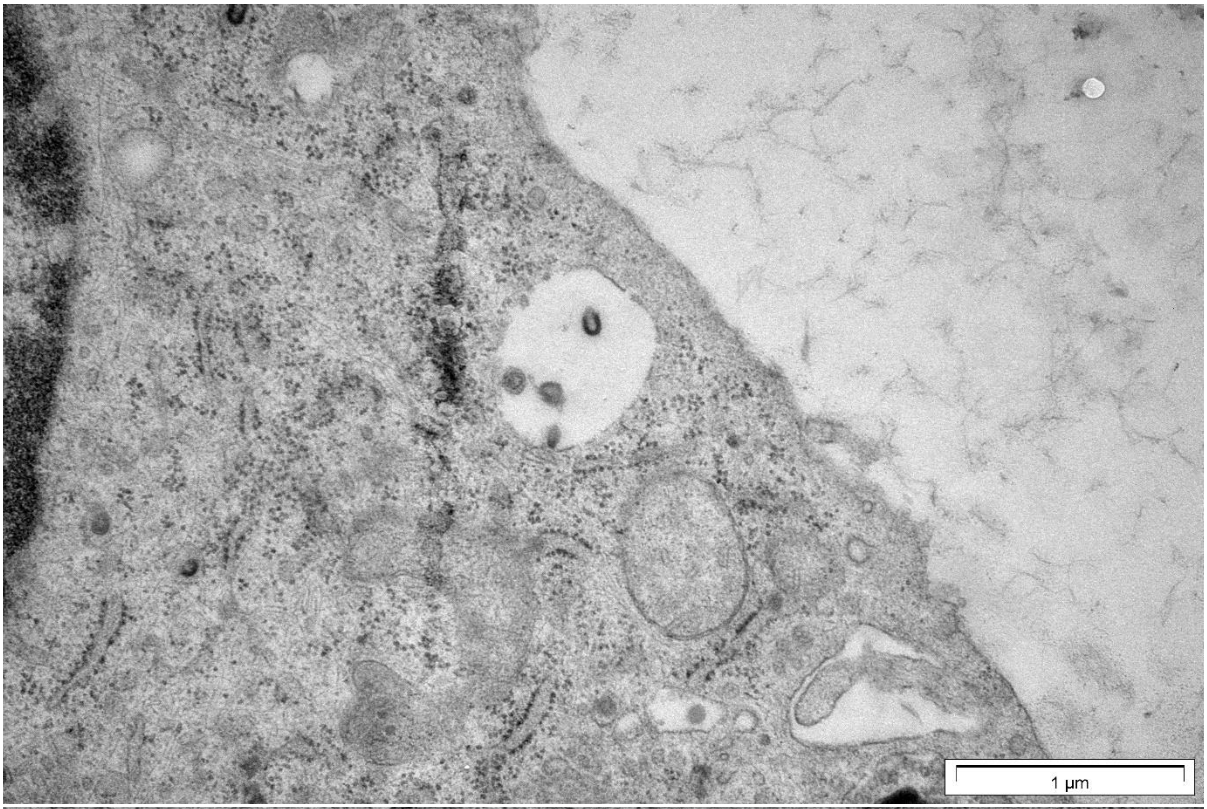
1 µm

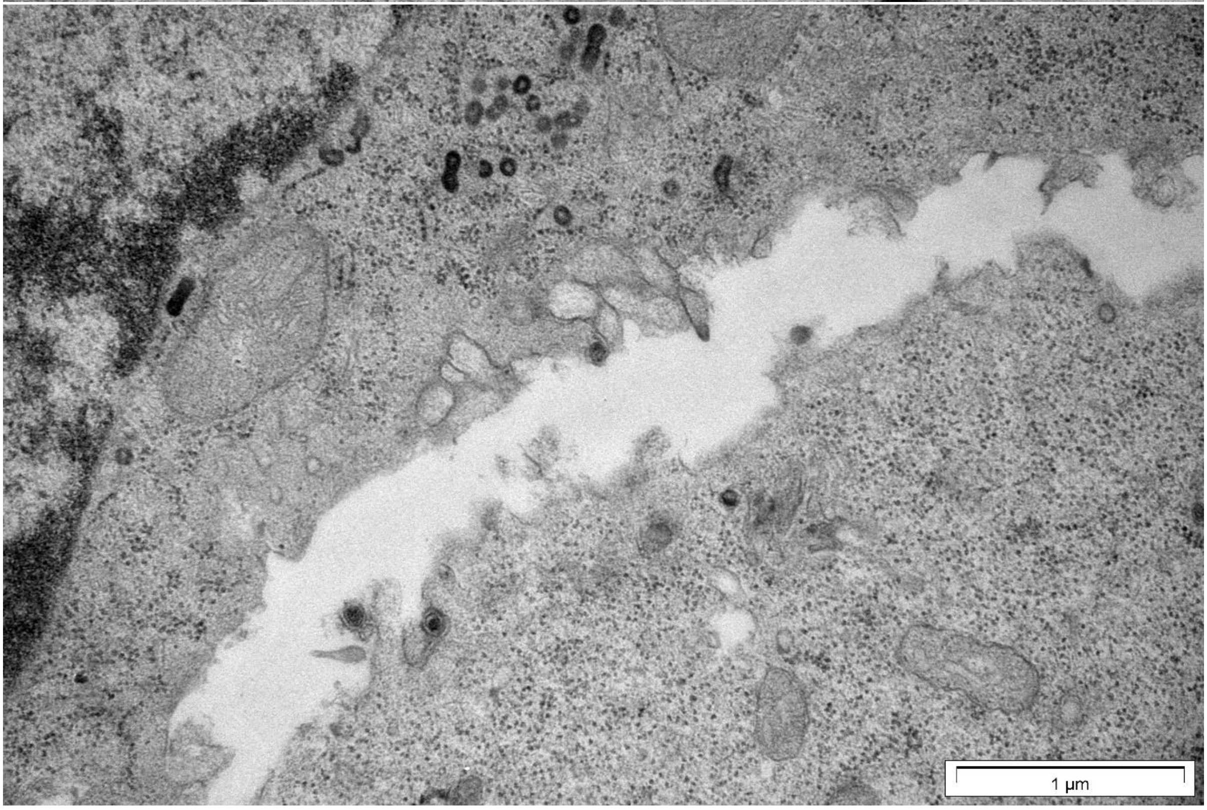
1 µm

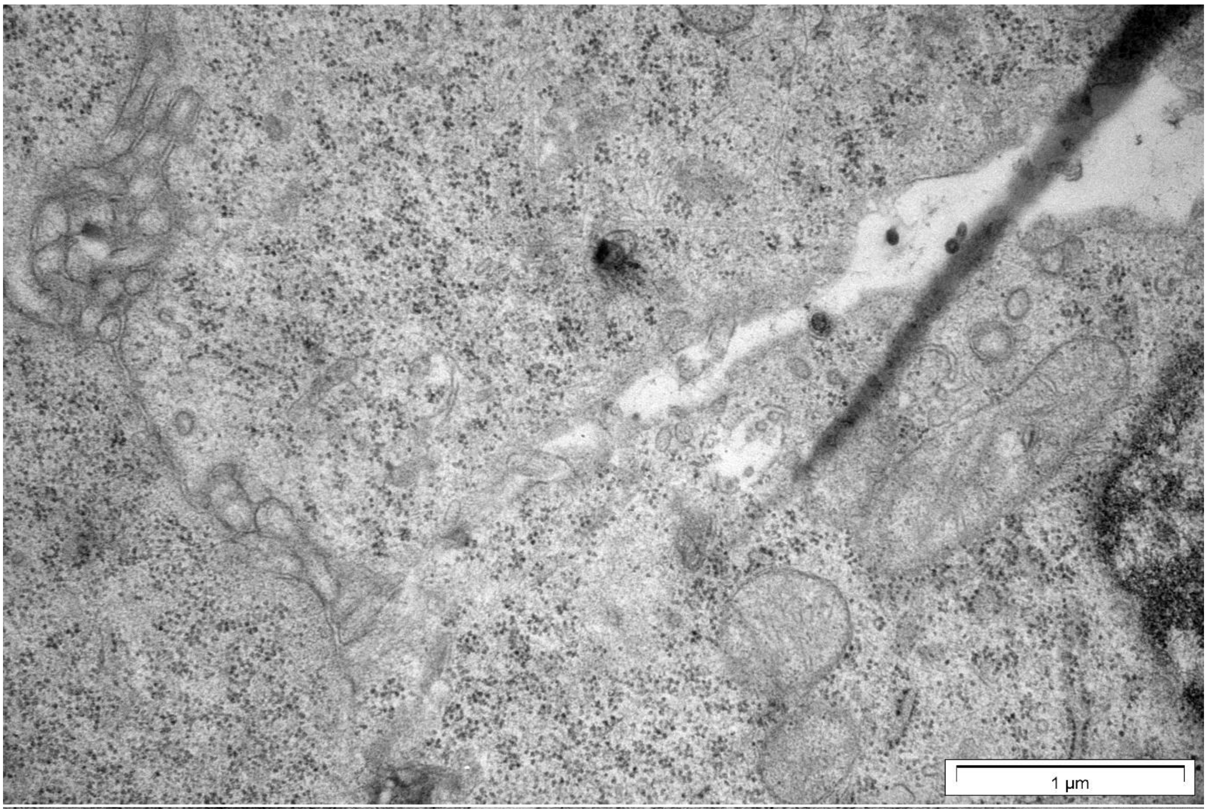
1 µm

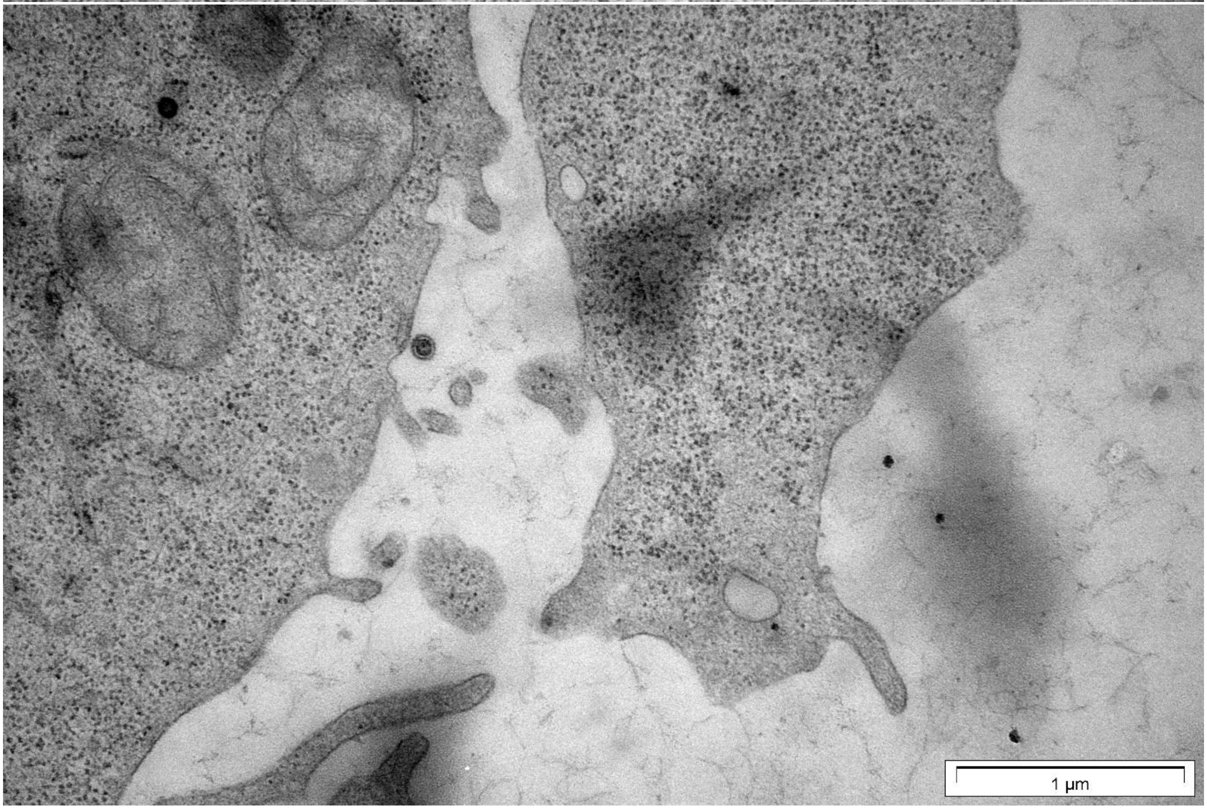
1 µm

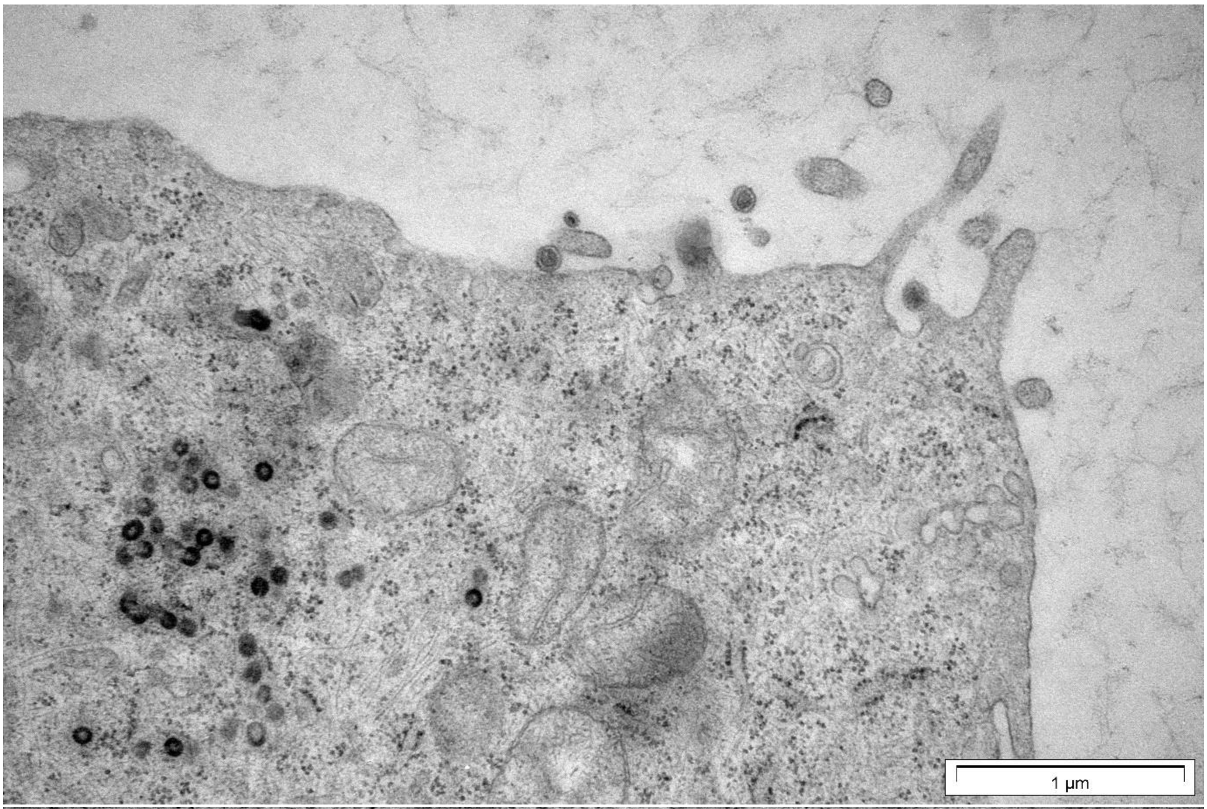
1 µm

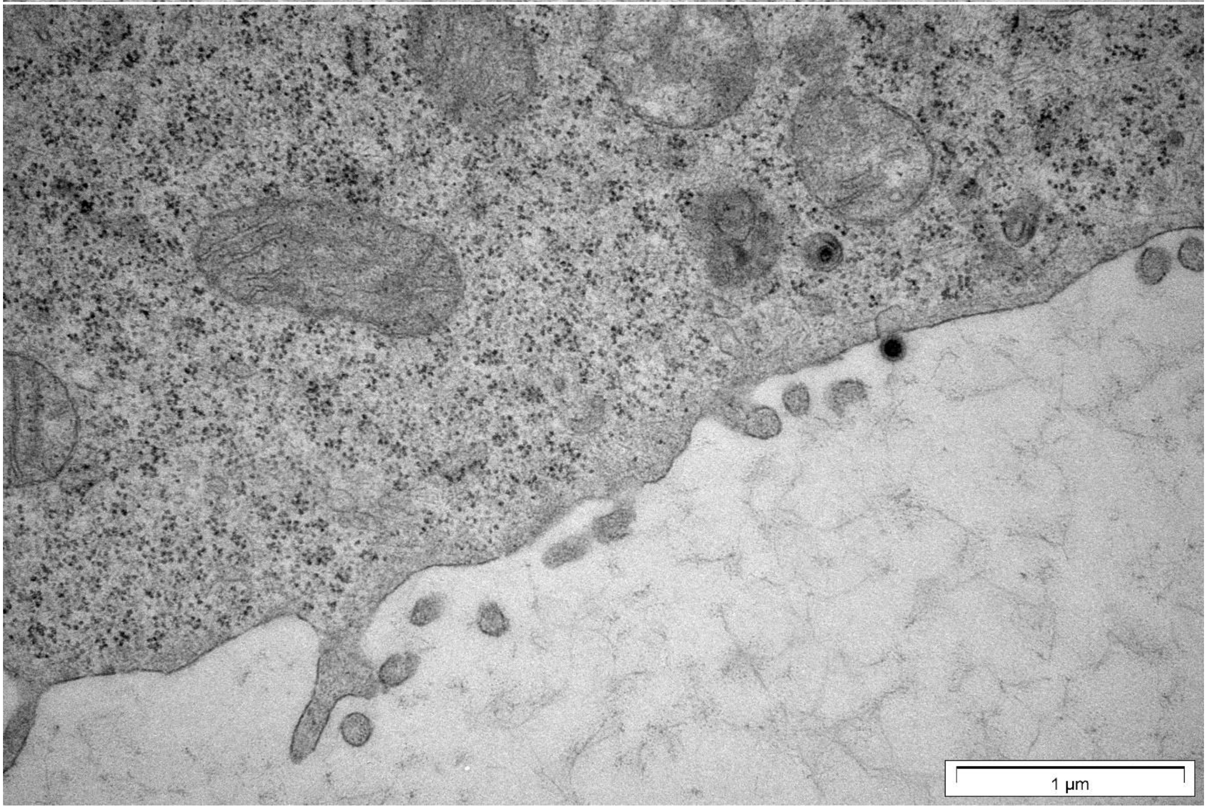
1 µm

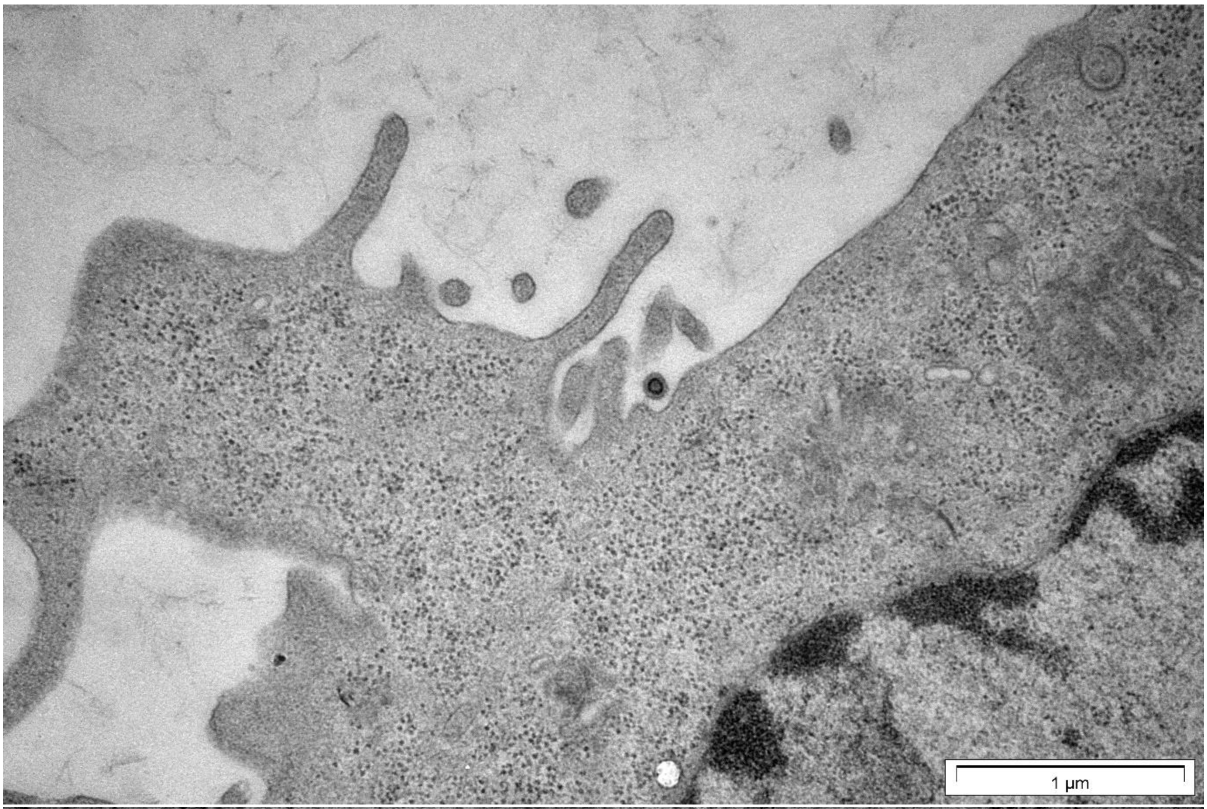
1 µm

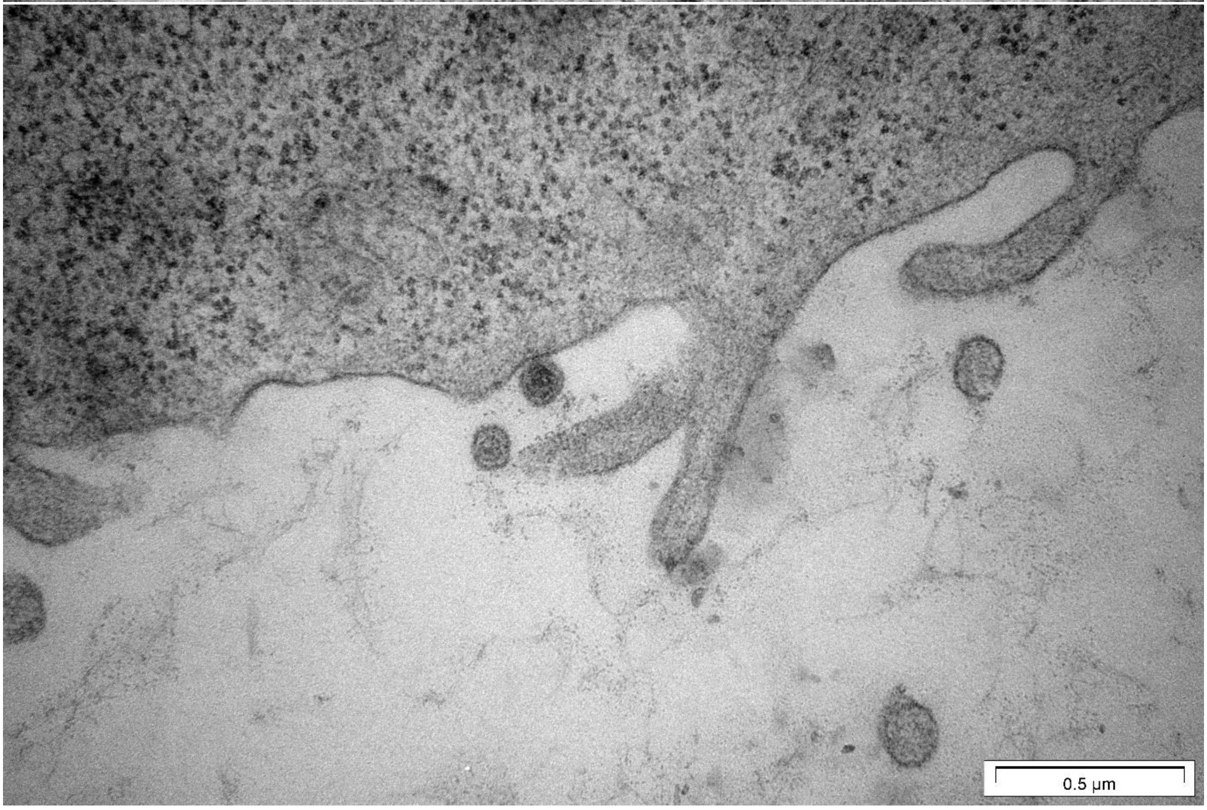
0.5 µm

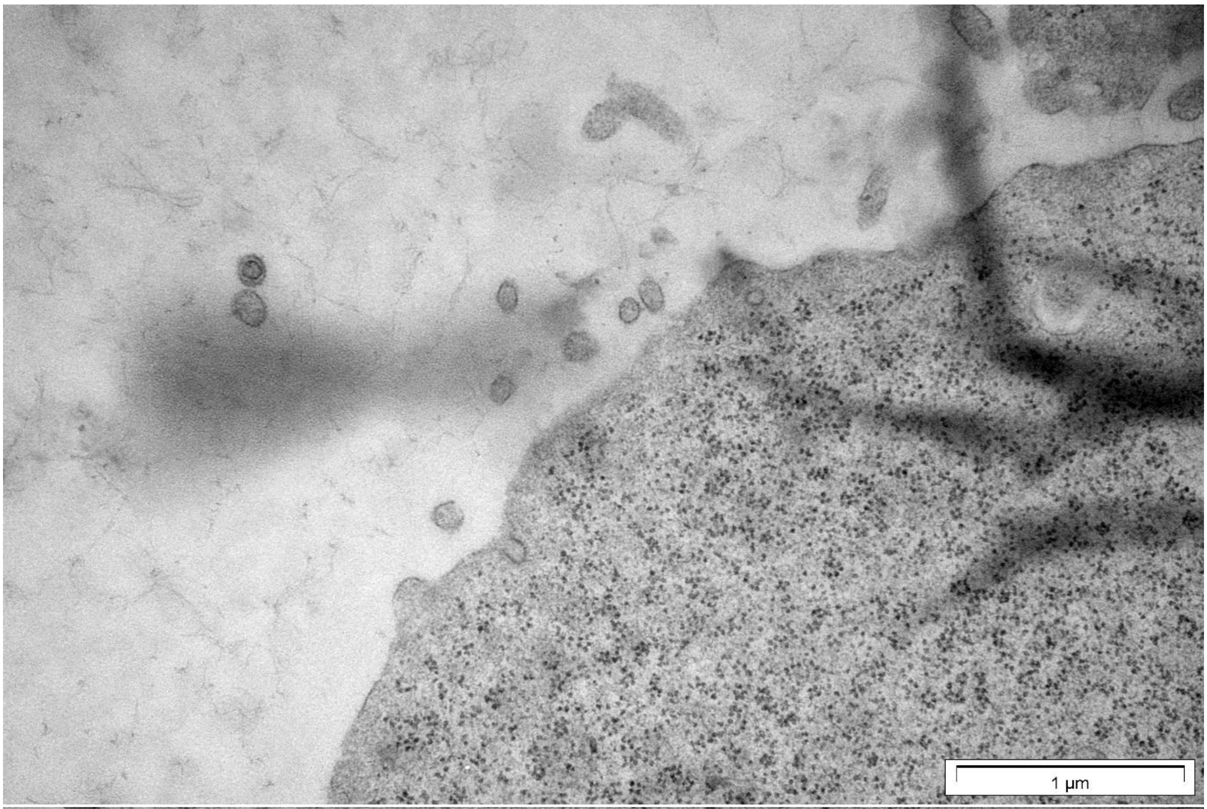
1 µm

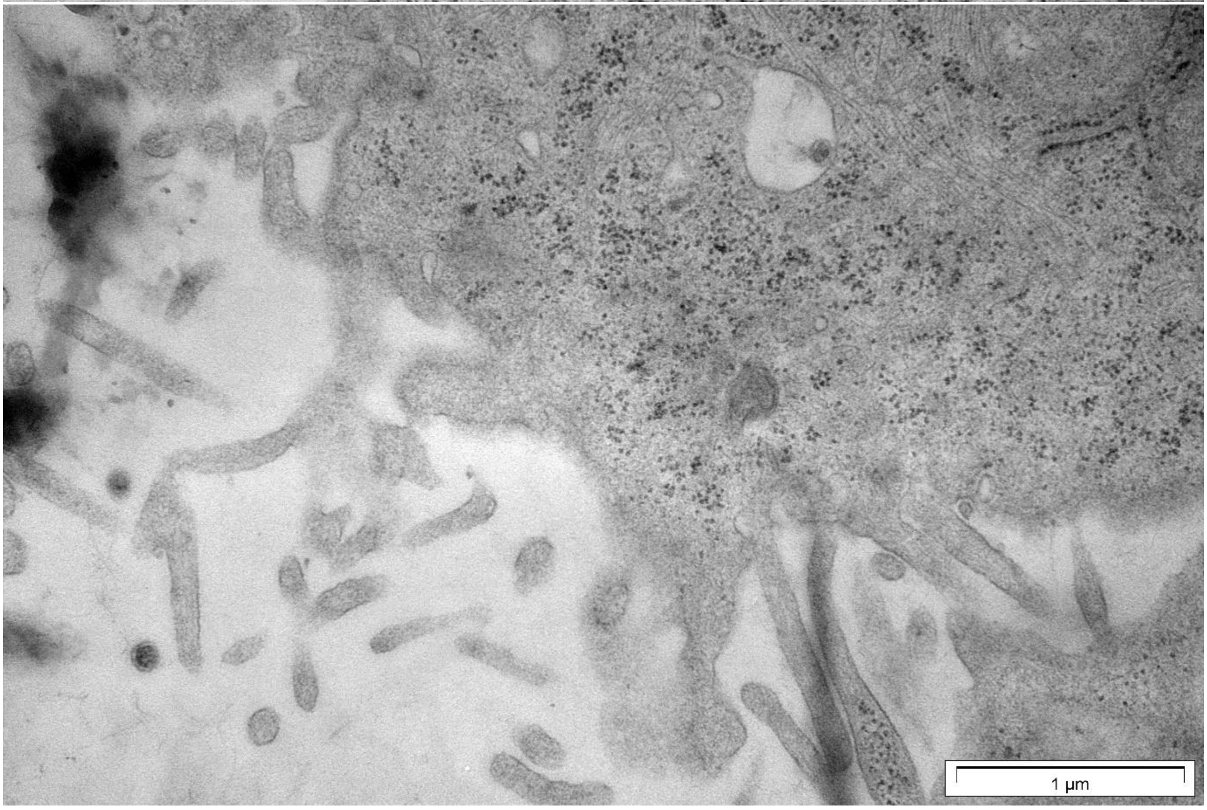
1 µm

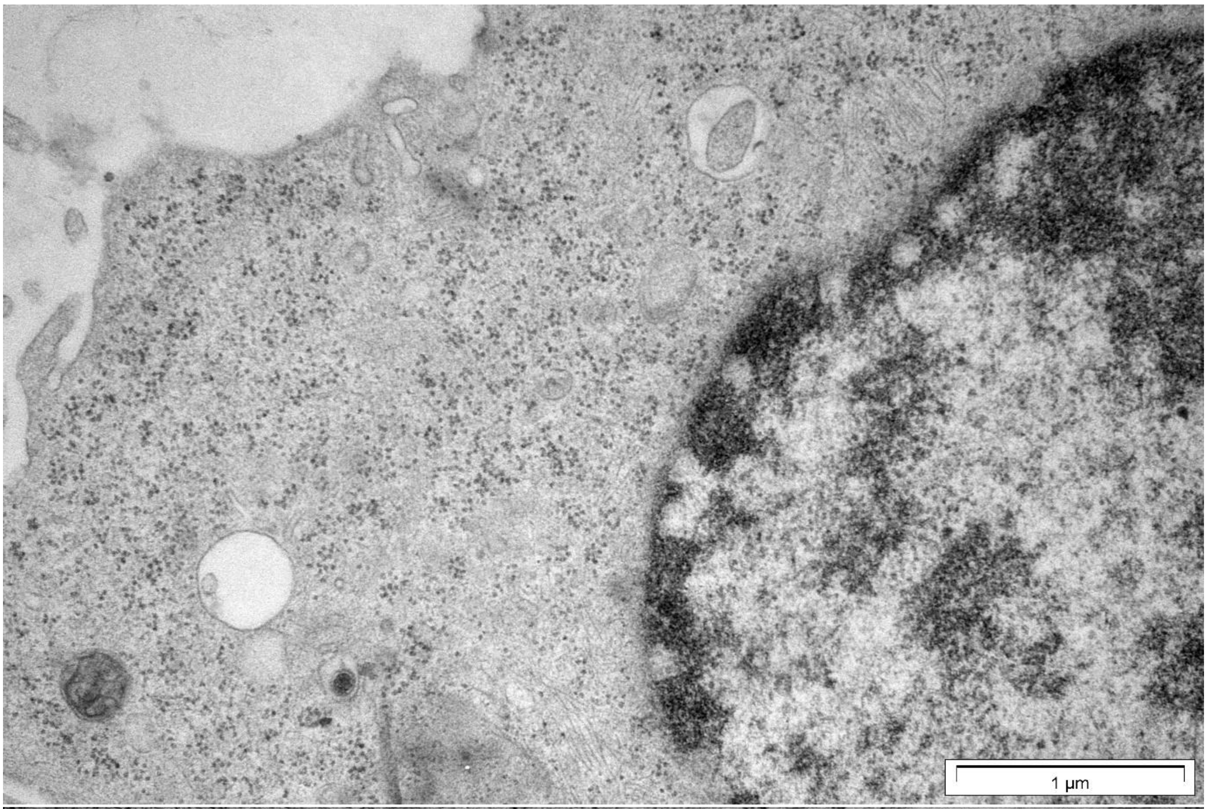
1 µm

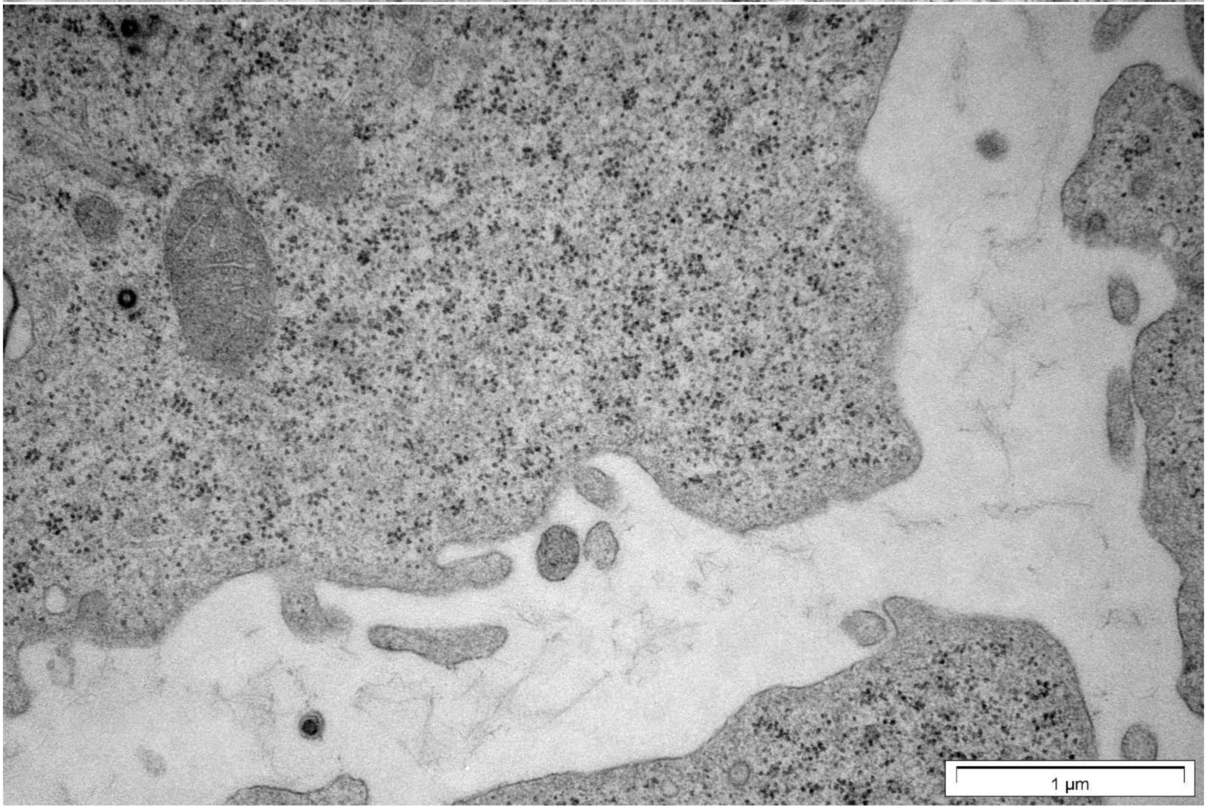
1 µm

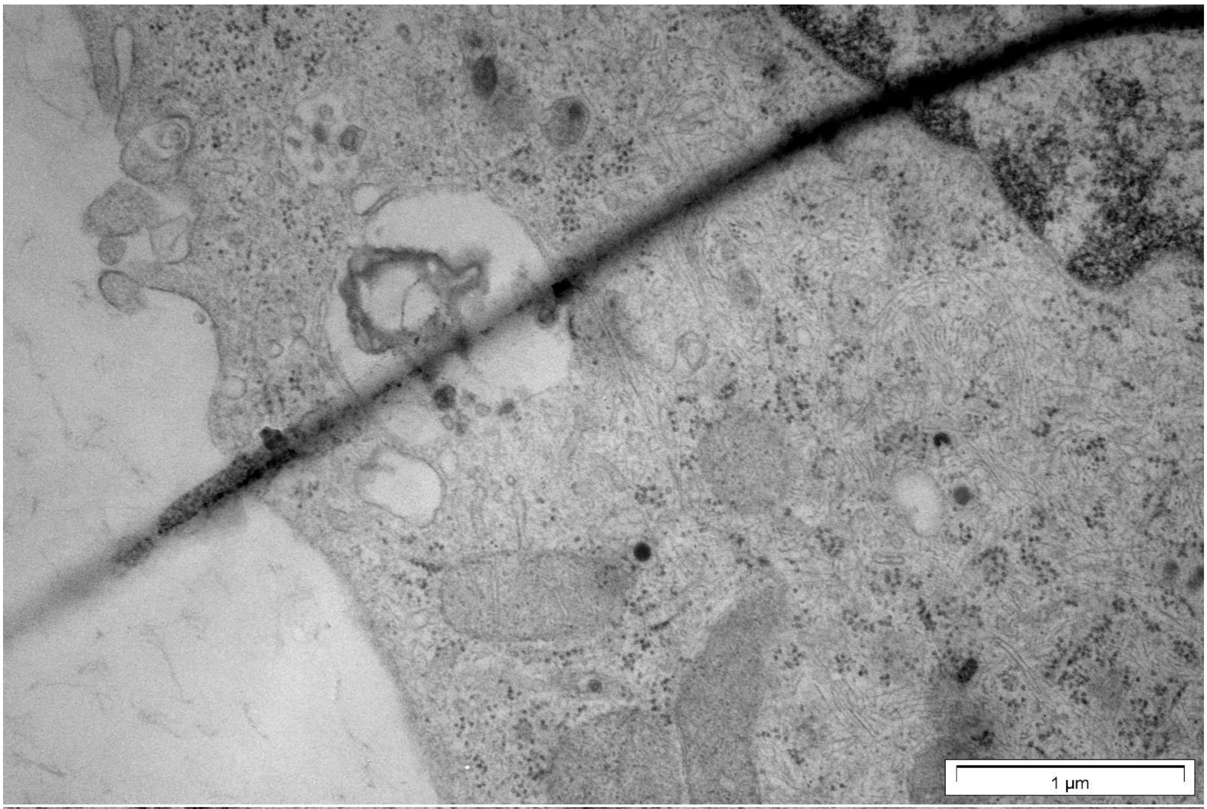
1 µm

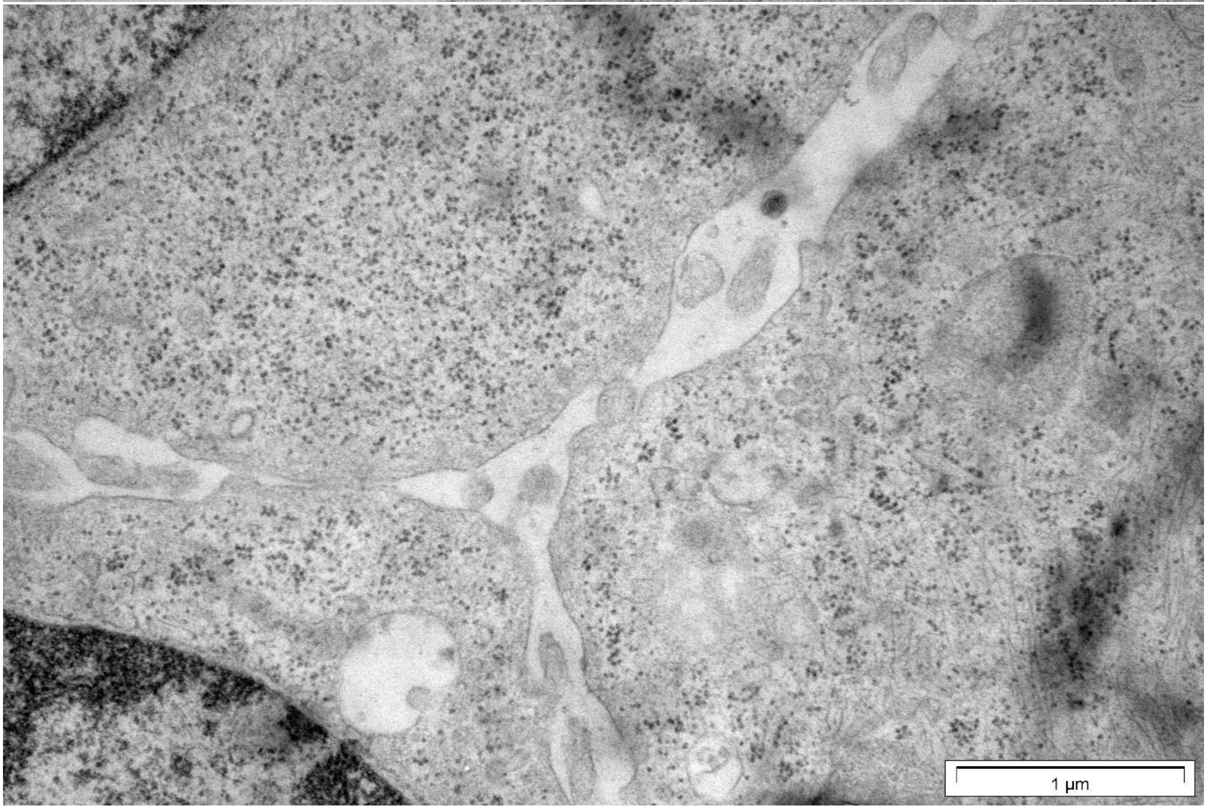
1 µm

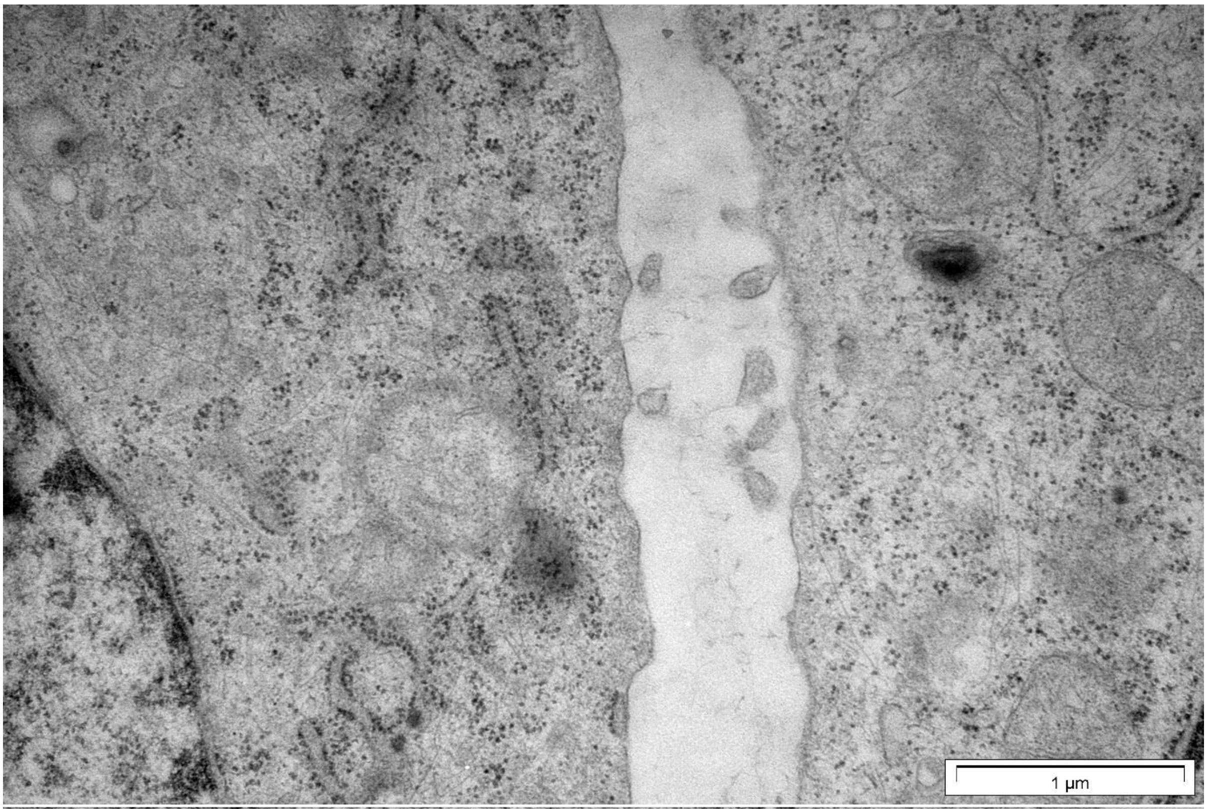
1 µm

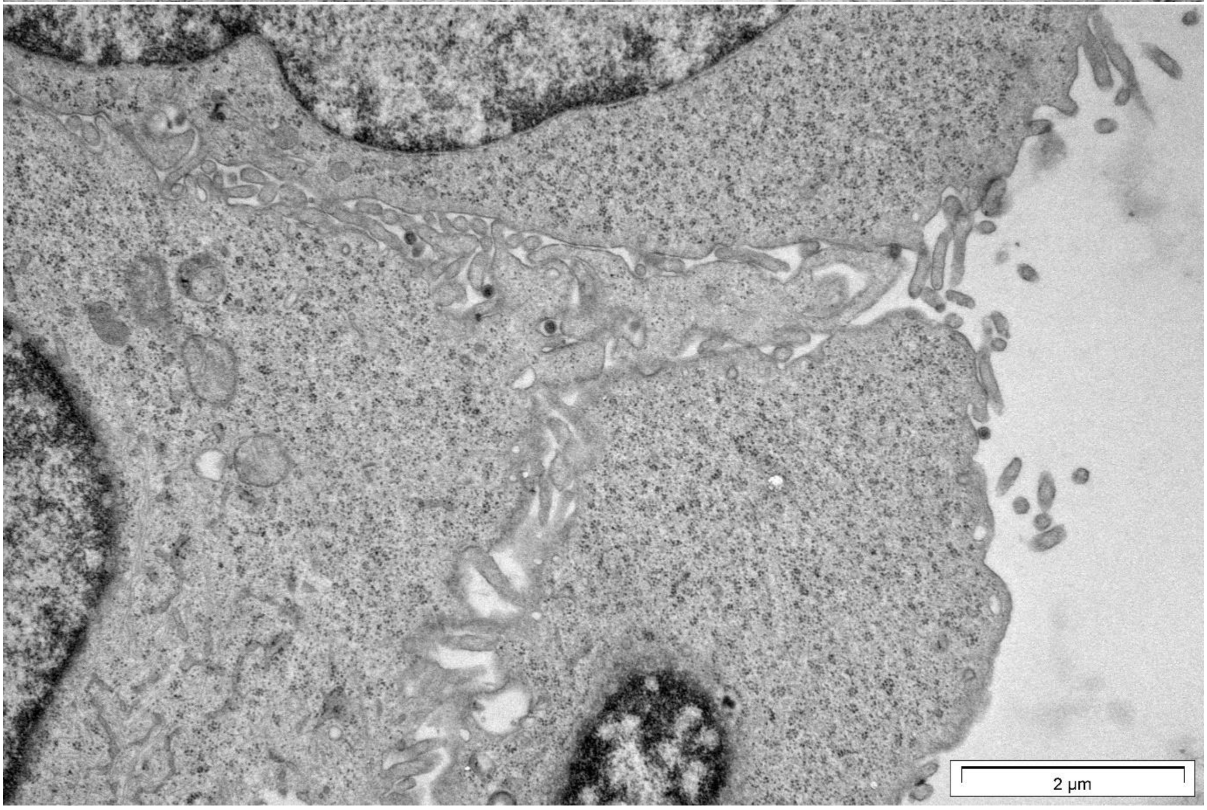
2 µm

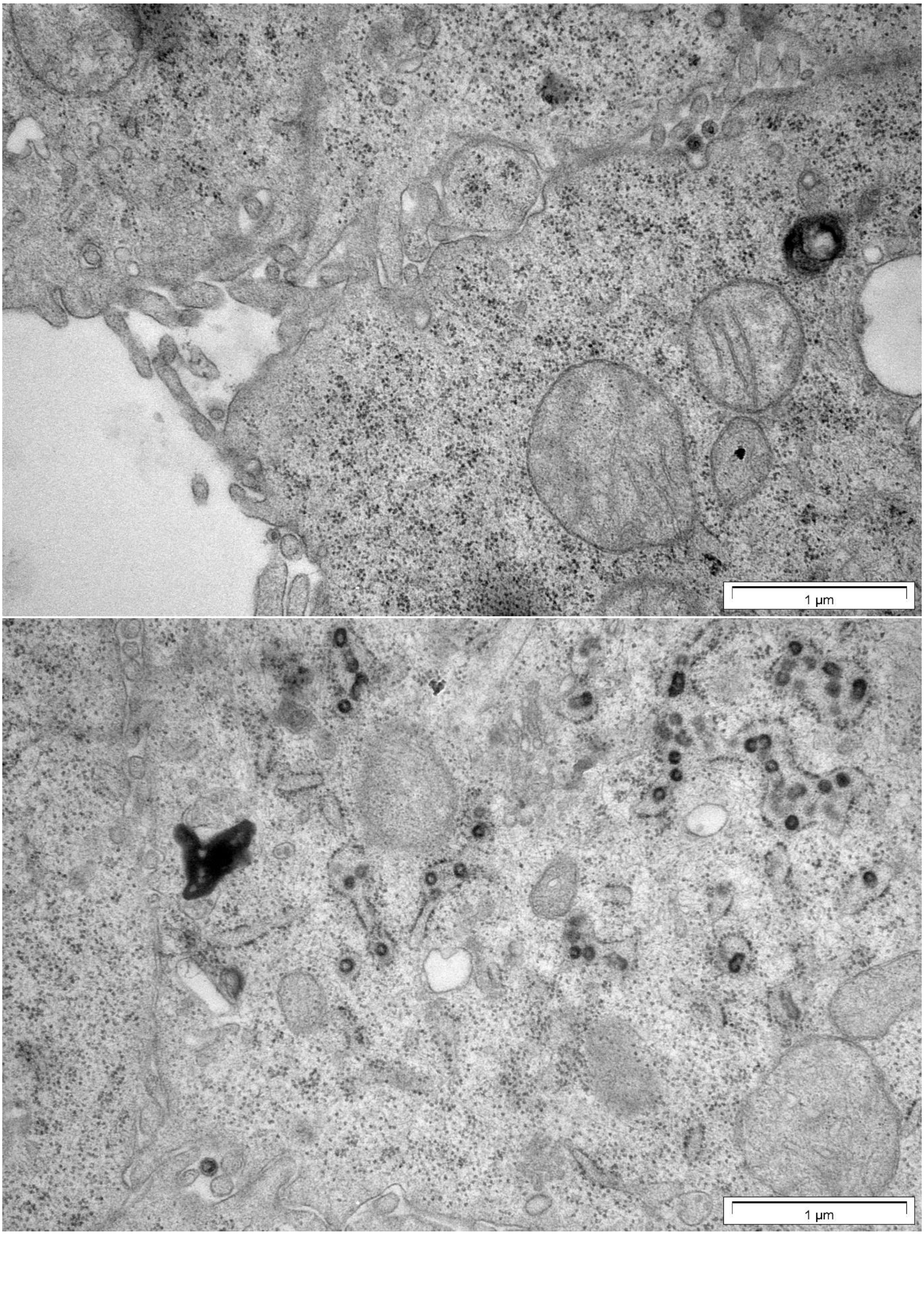
1 µm
1 µm

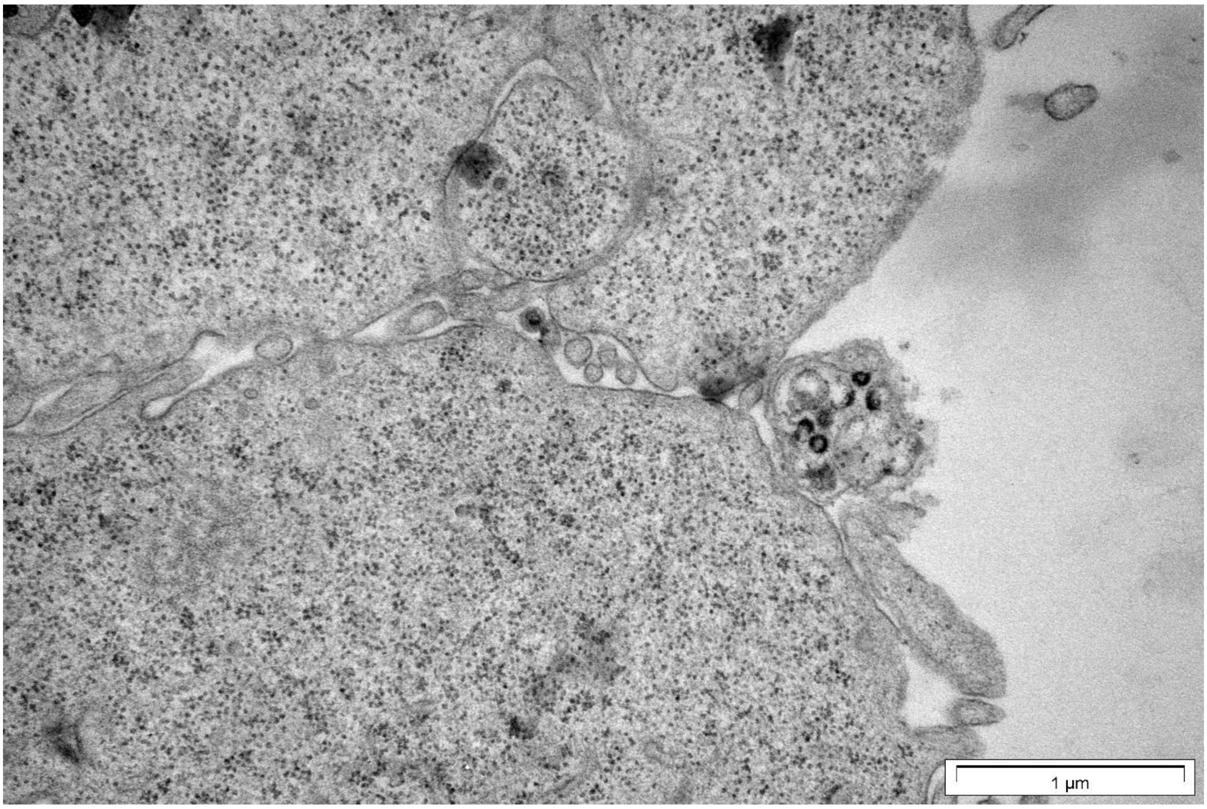
1 µm

Dextran, 5 min.

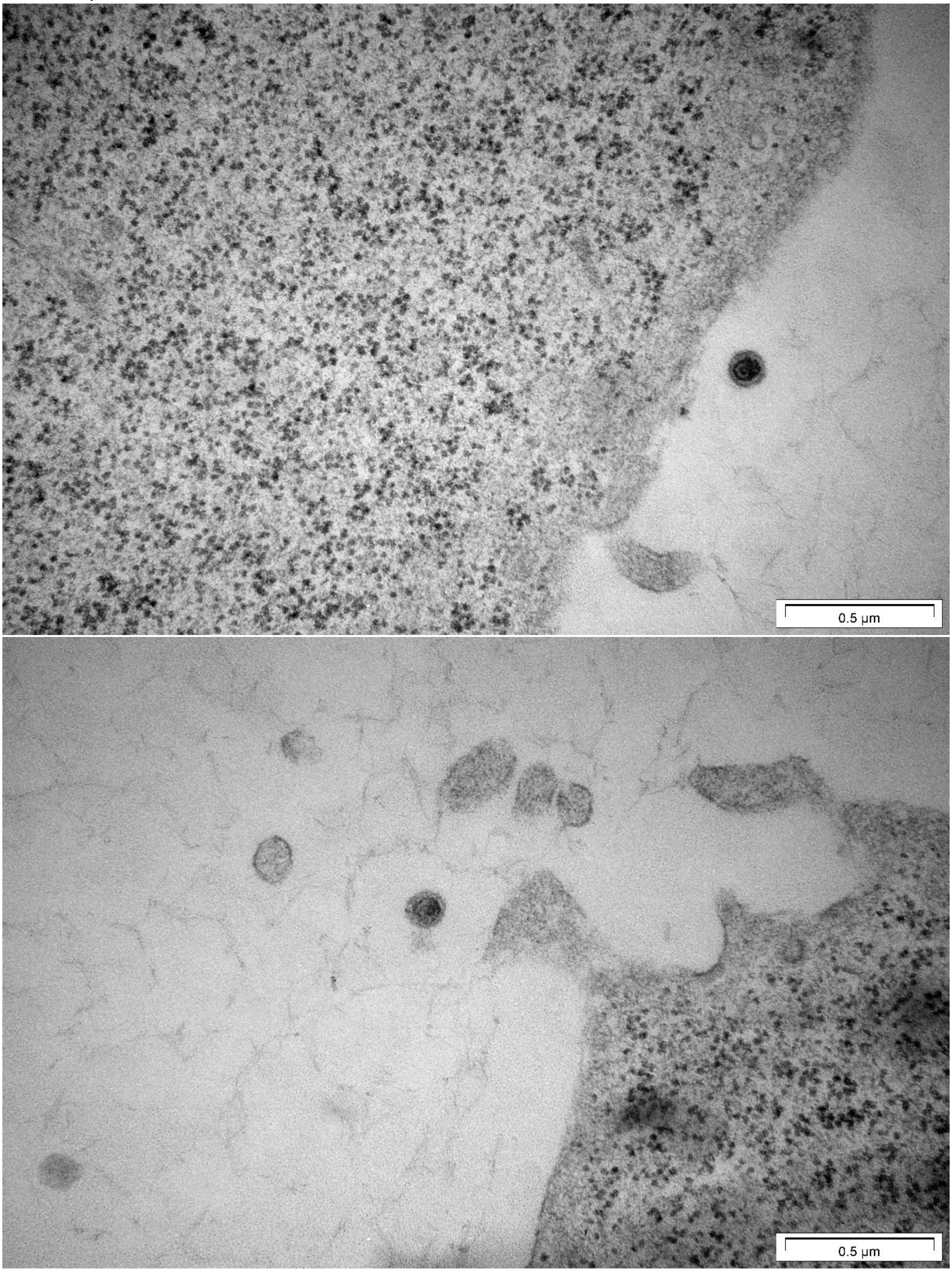

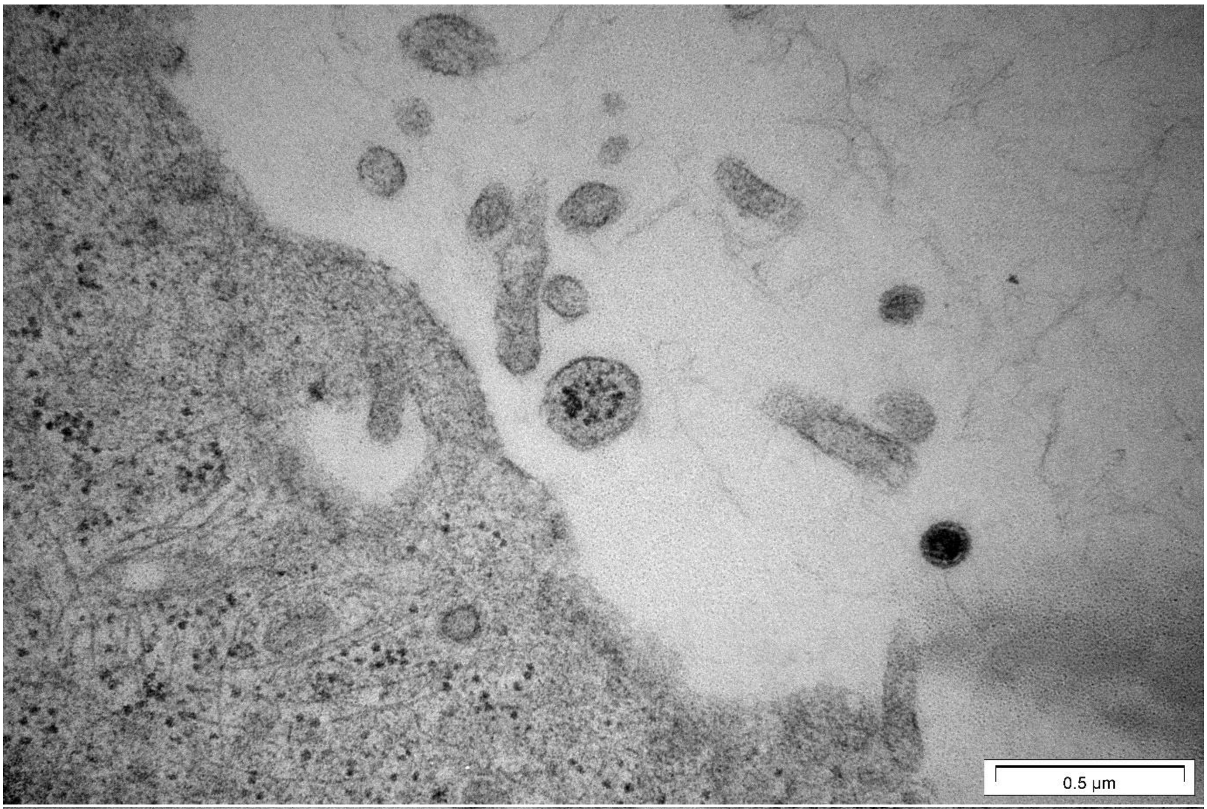
0.5 µm

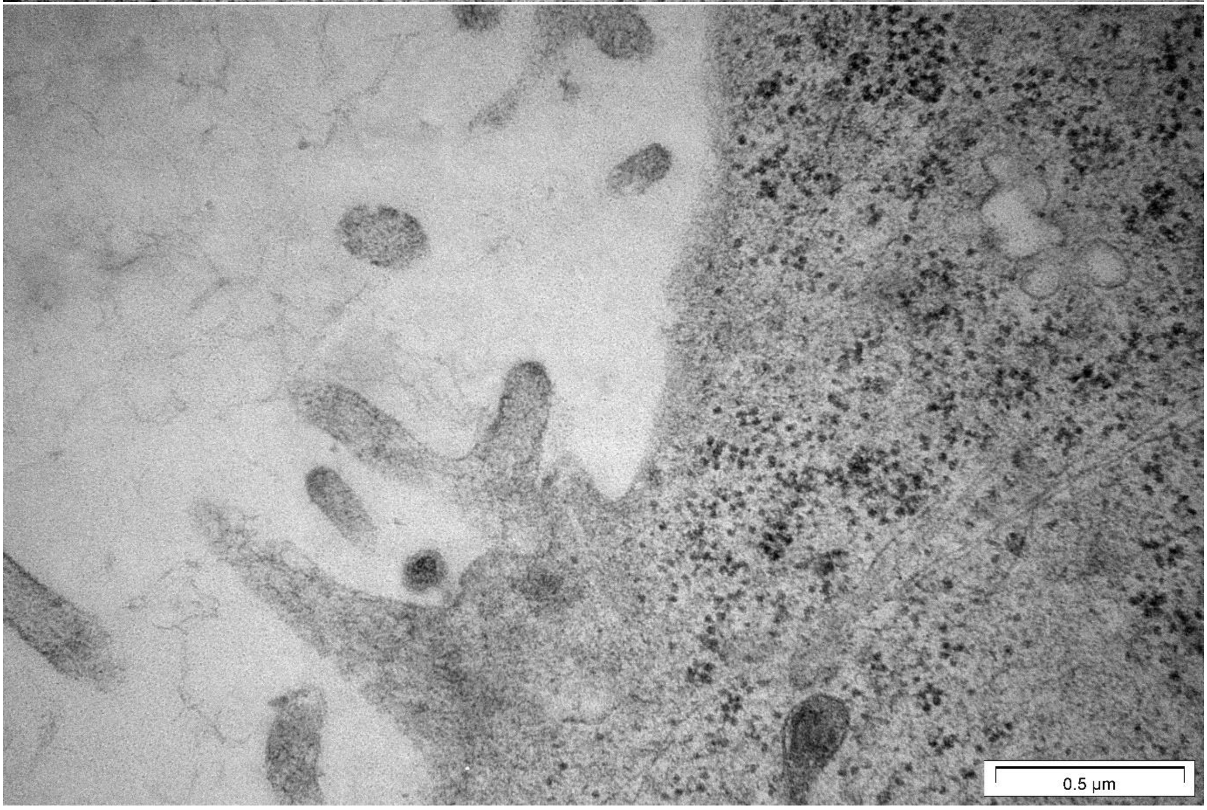
0.5 µm

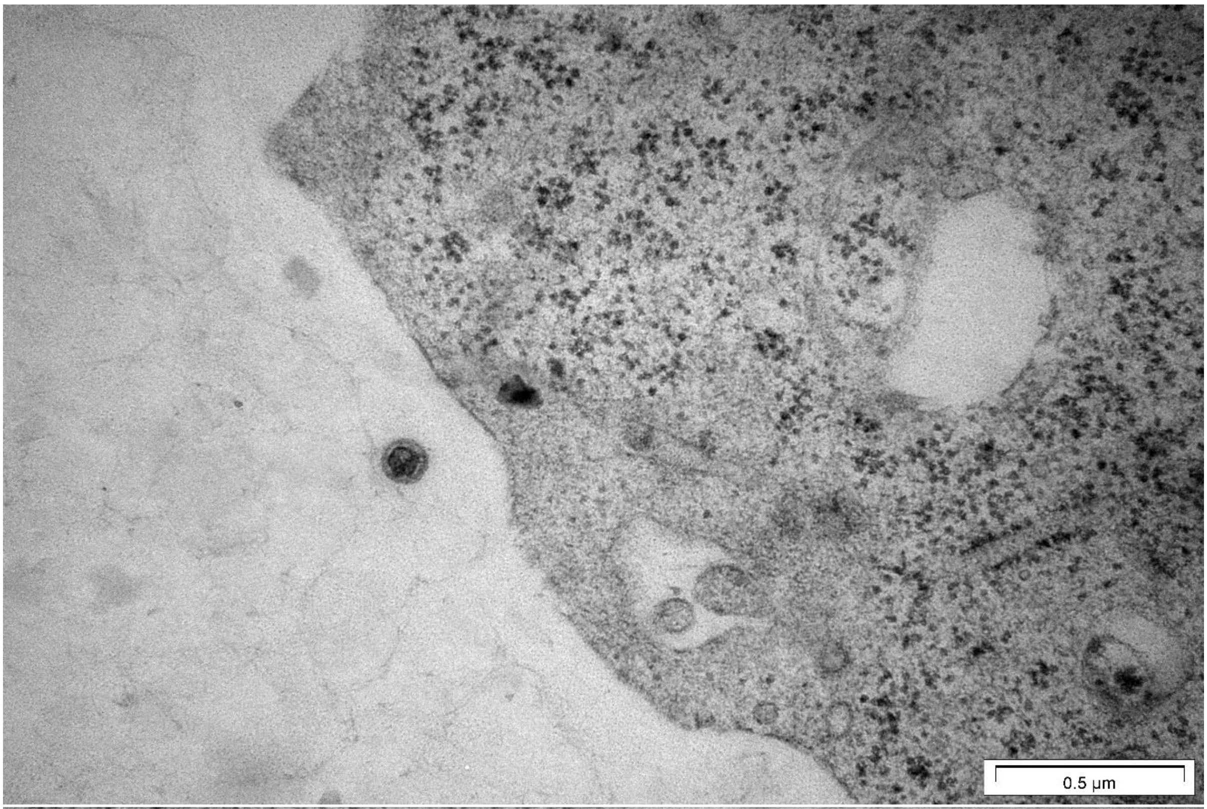
0.5 μm

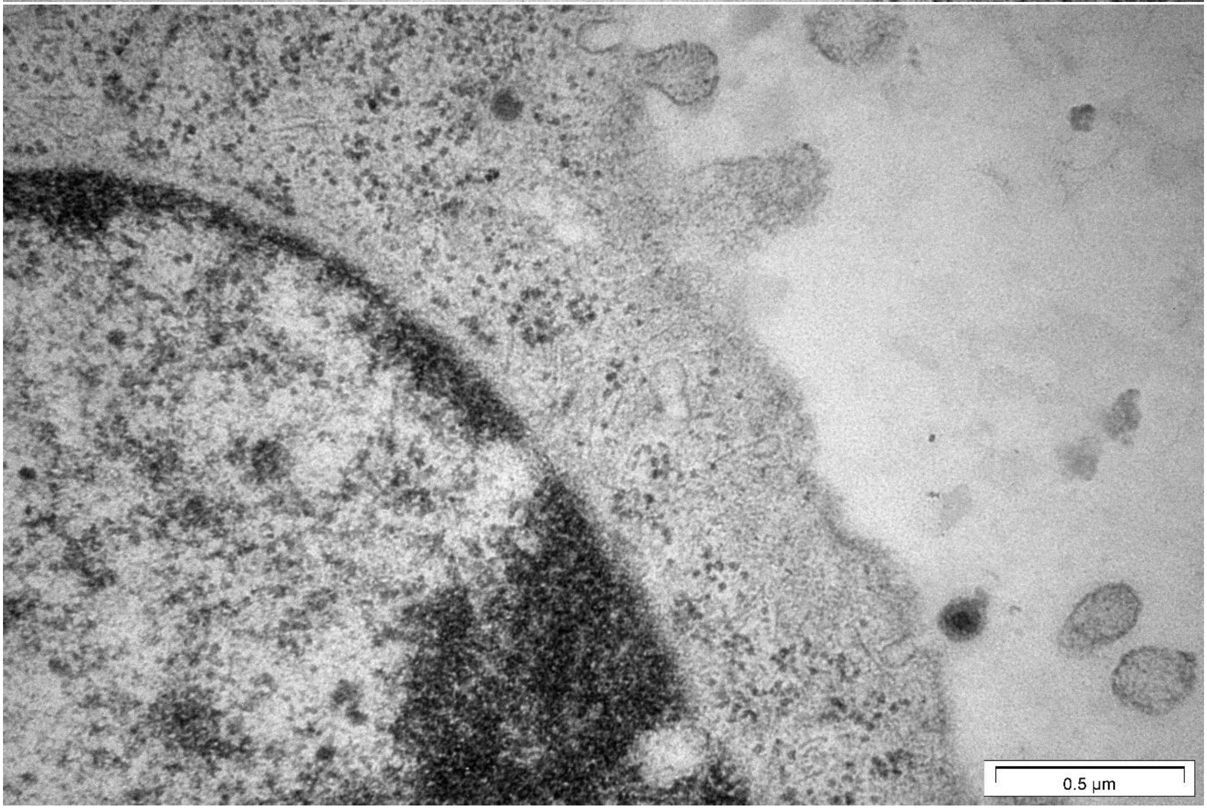
0.5 μm

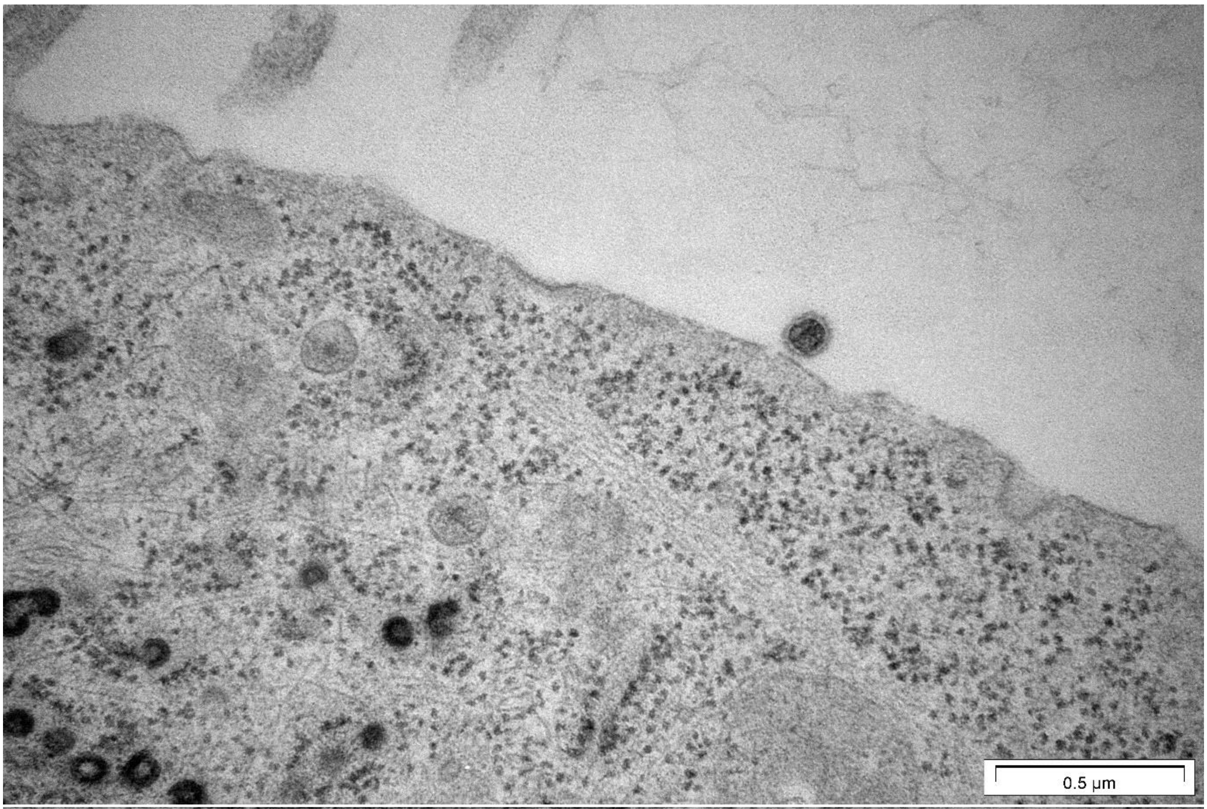
0.5 µm

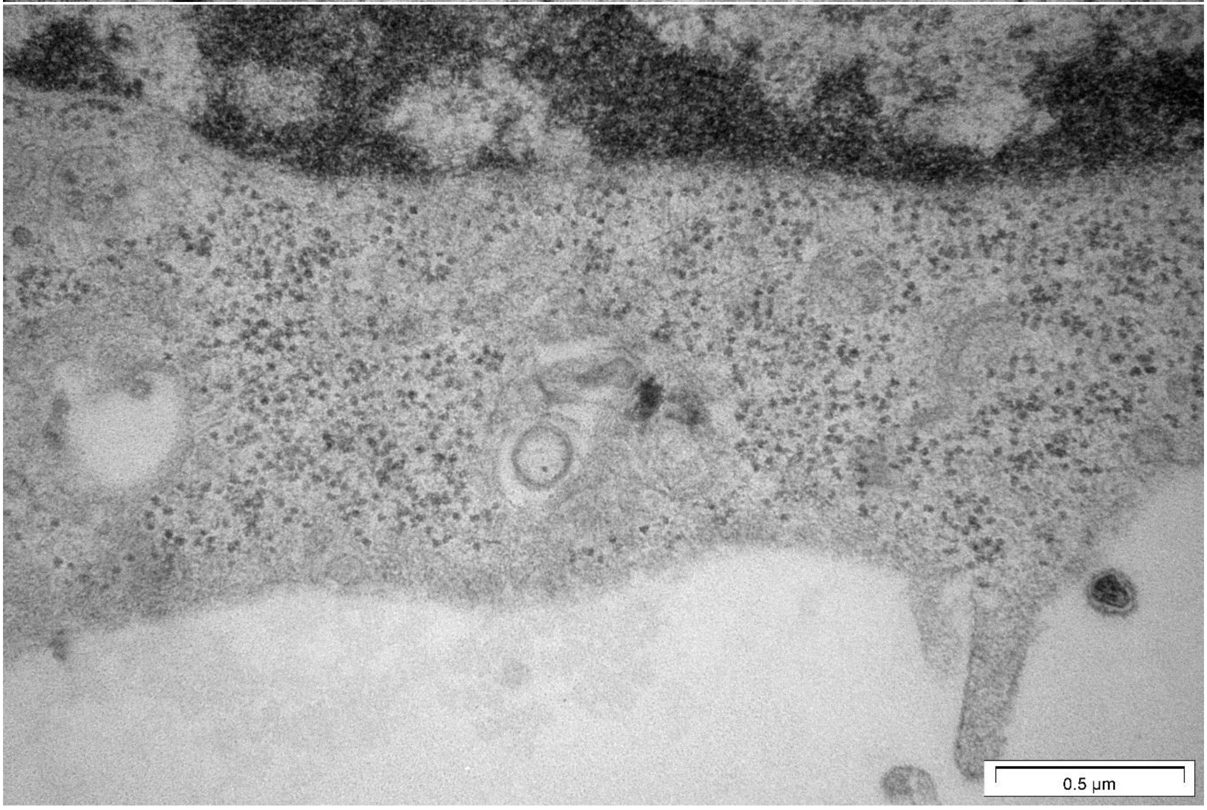
0.5 µm

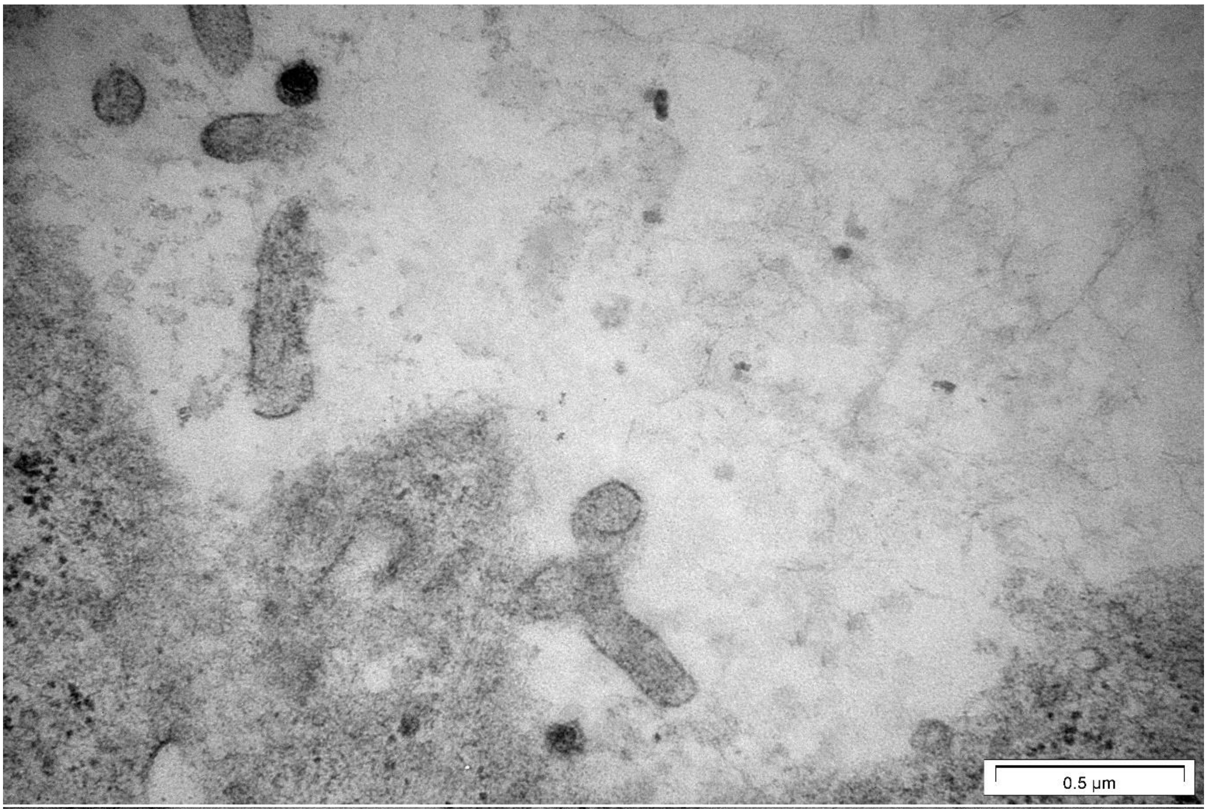
0.5 µm

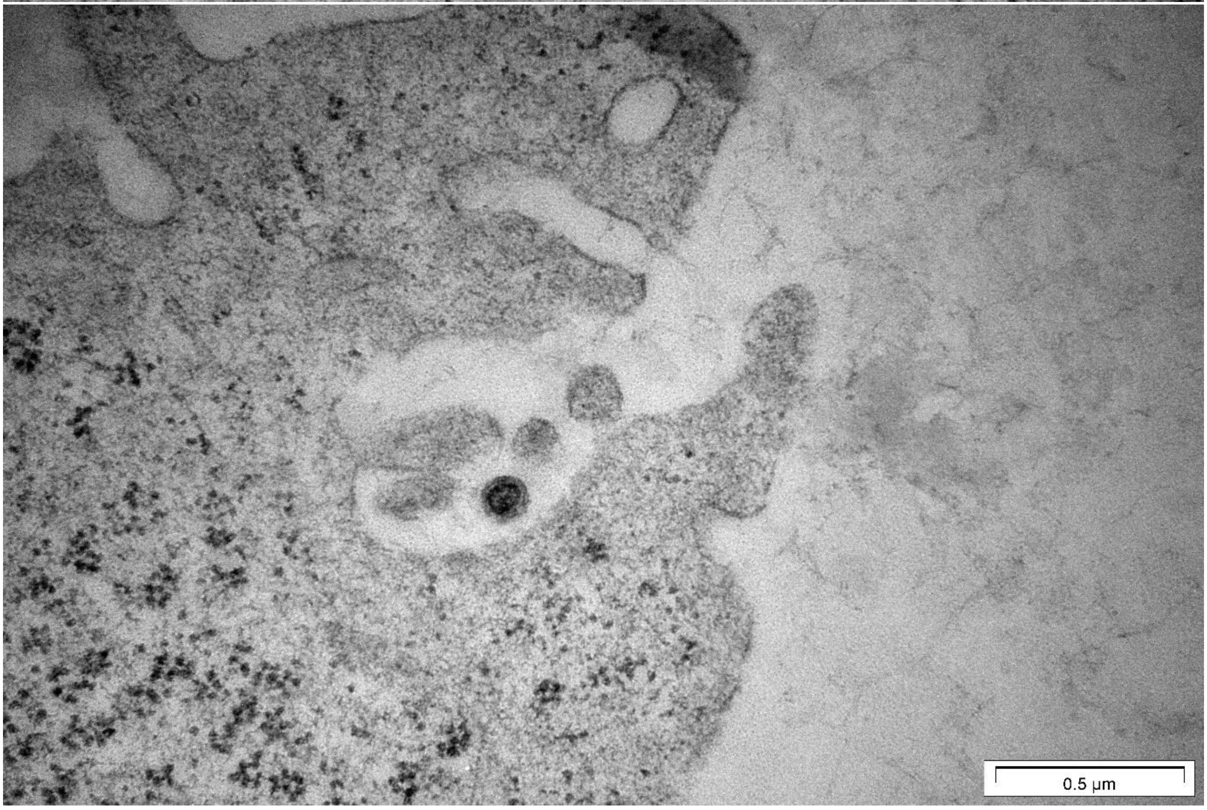
0.5 µm

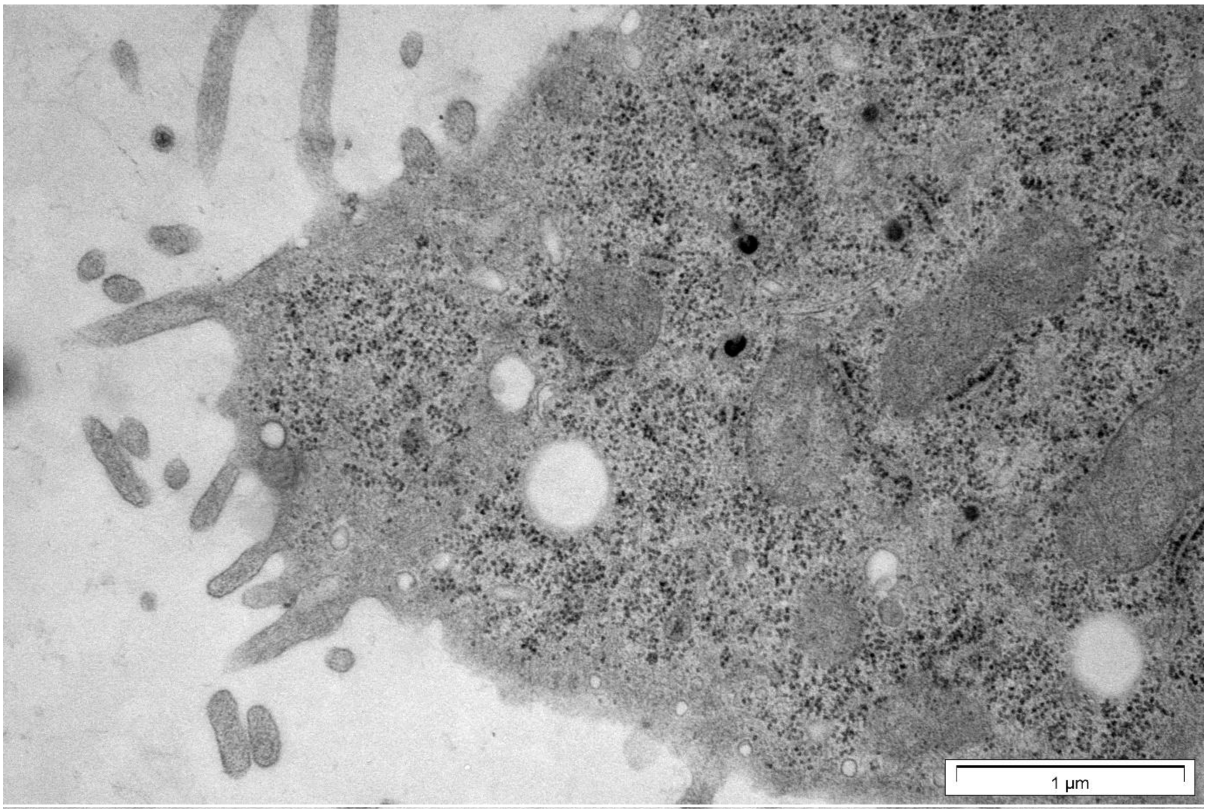
1 µm

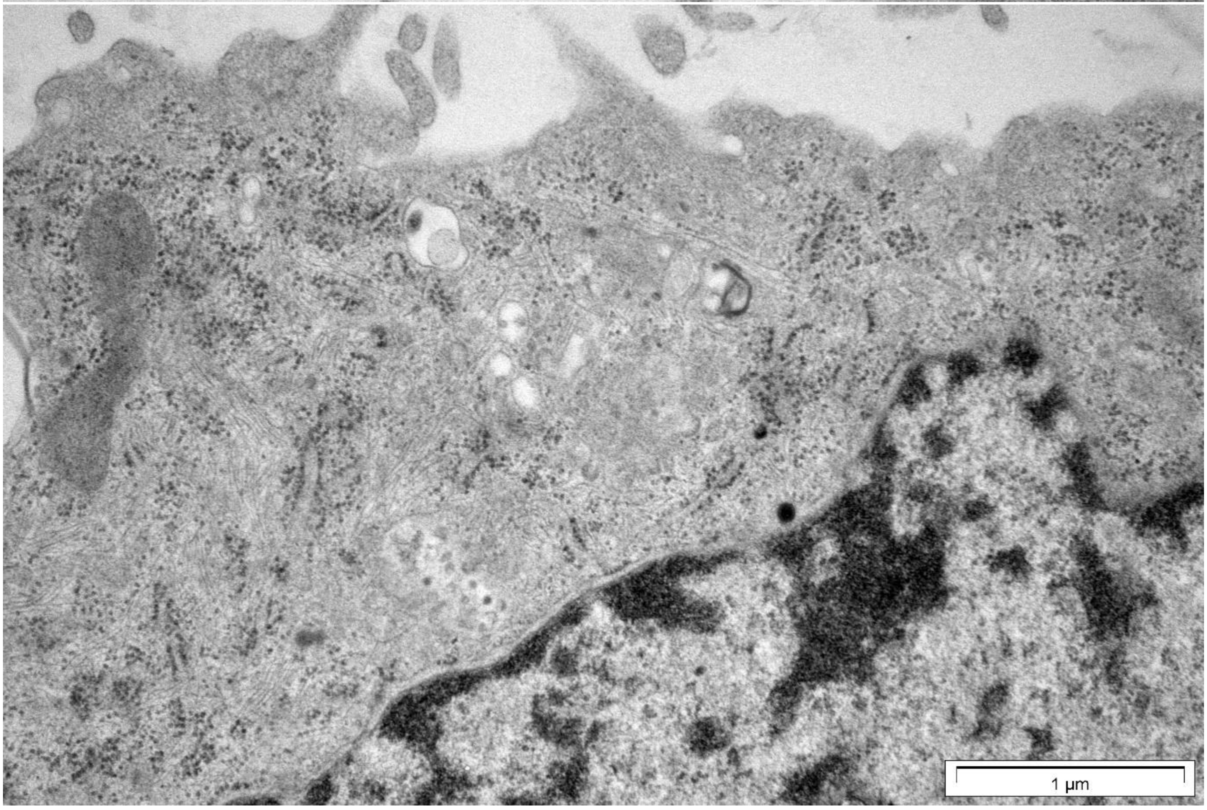
1 µm

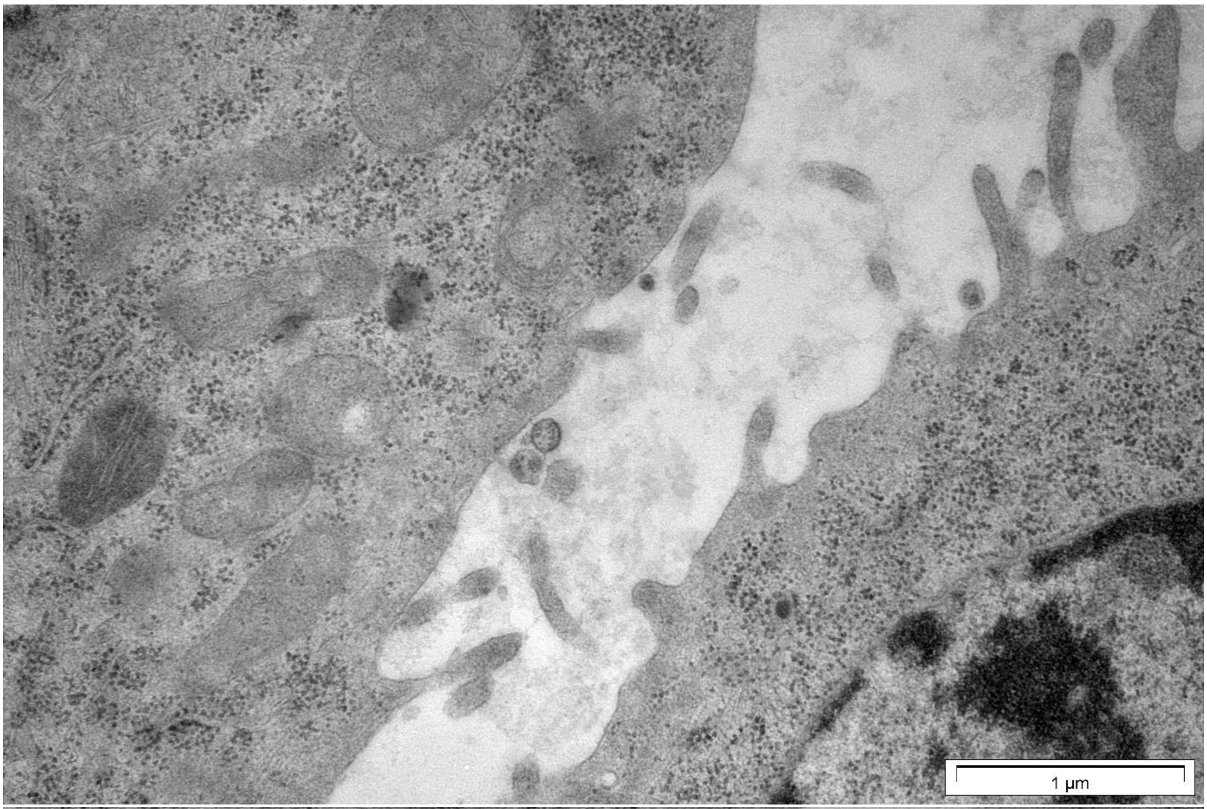
1 µm

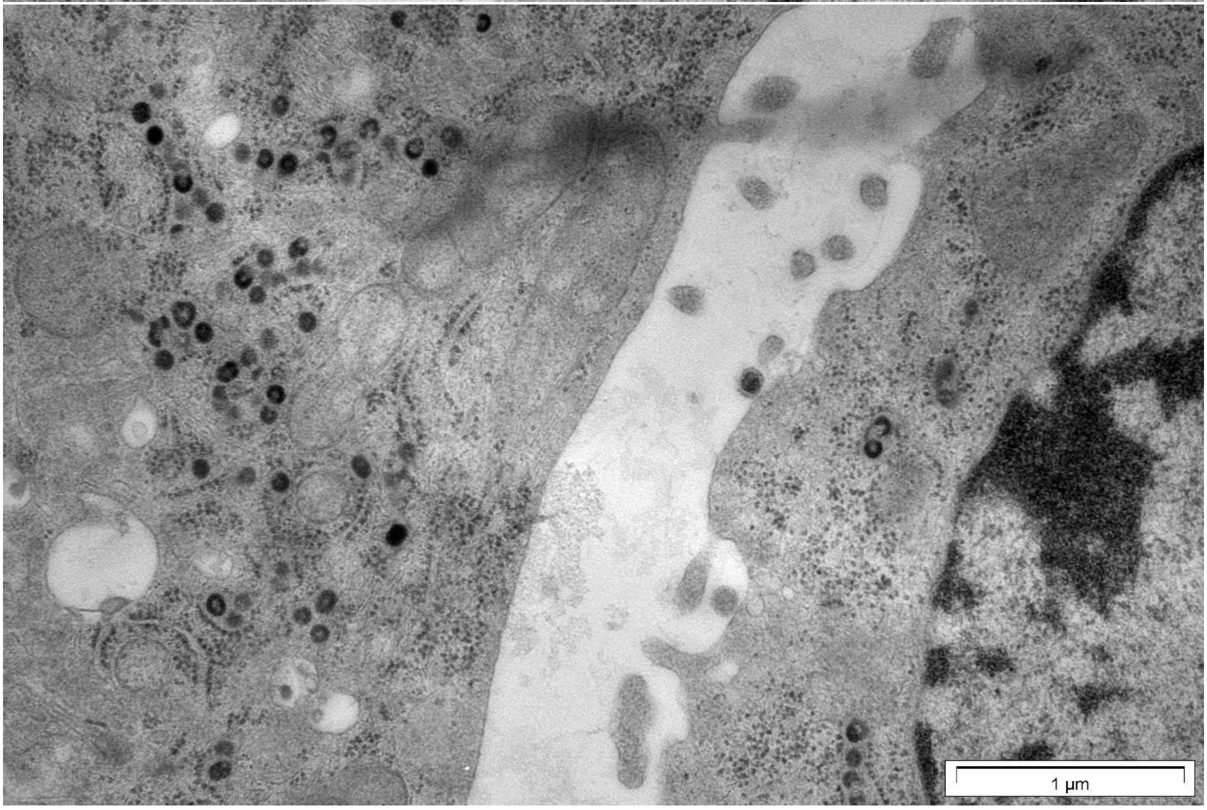
1 µm

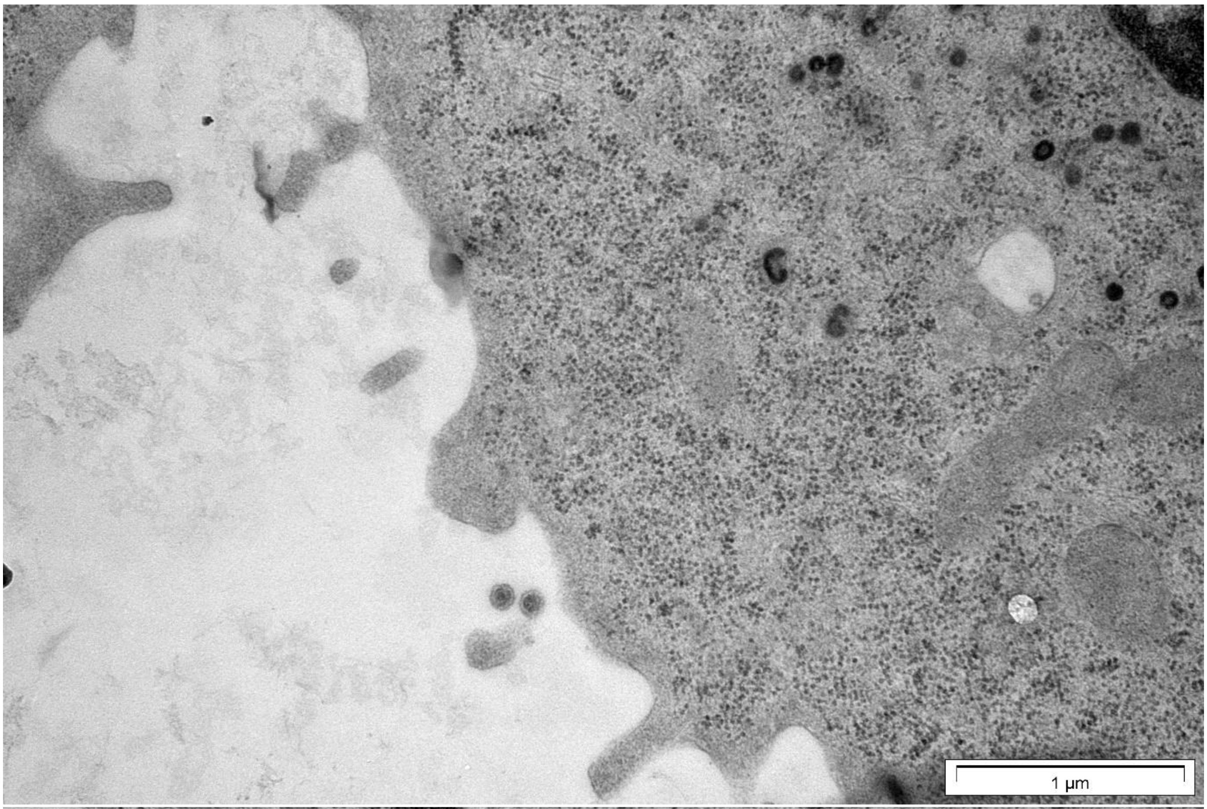
1 µm

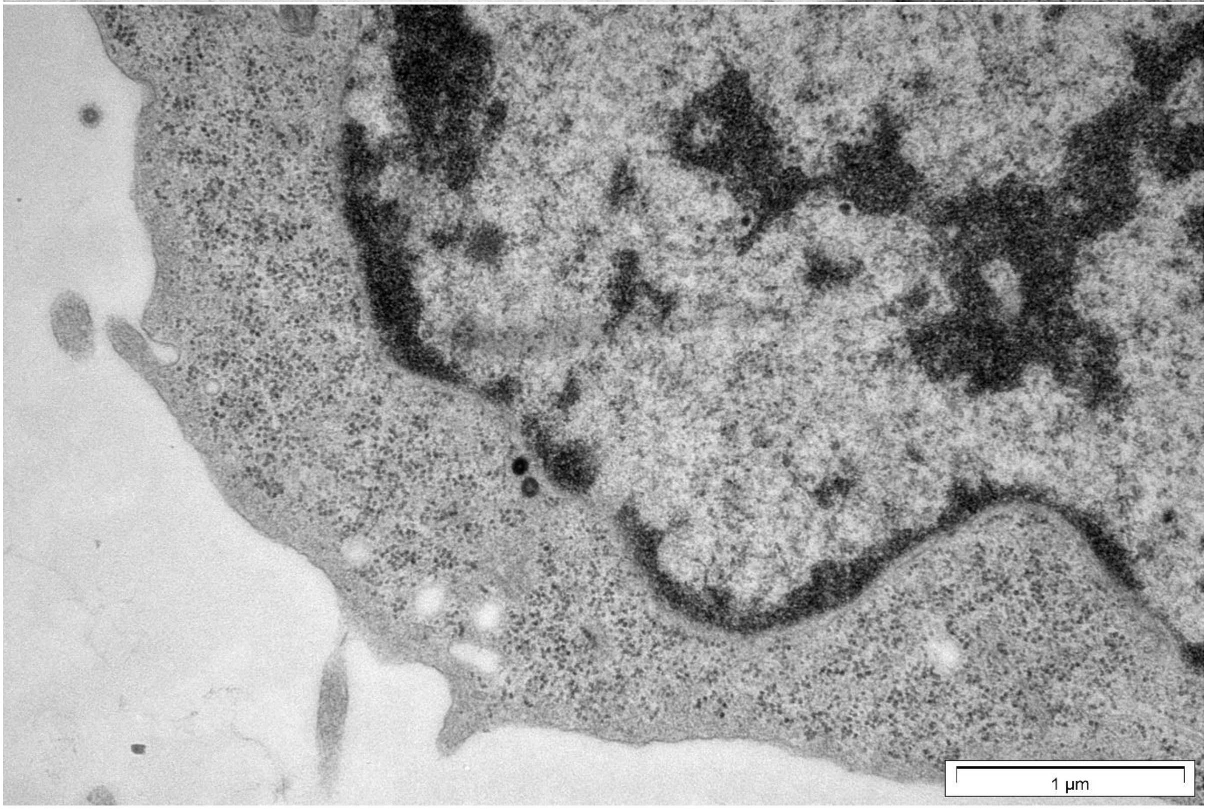
1 µm

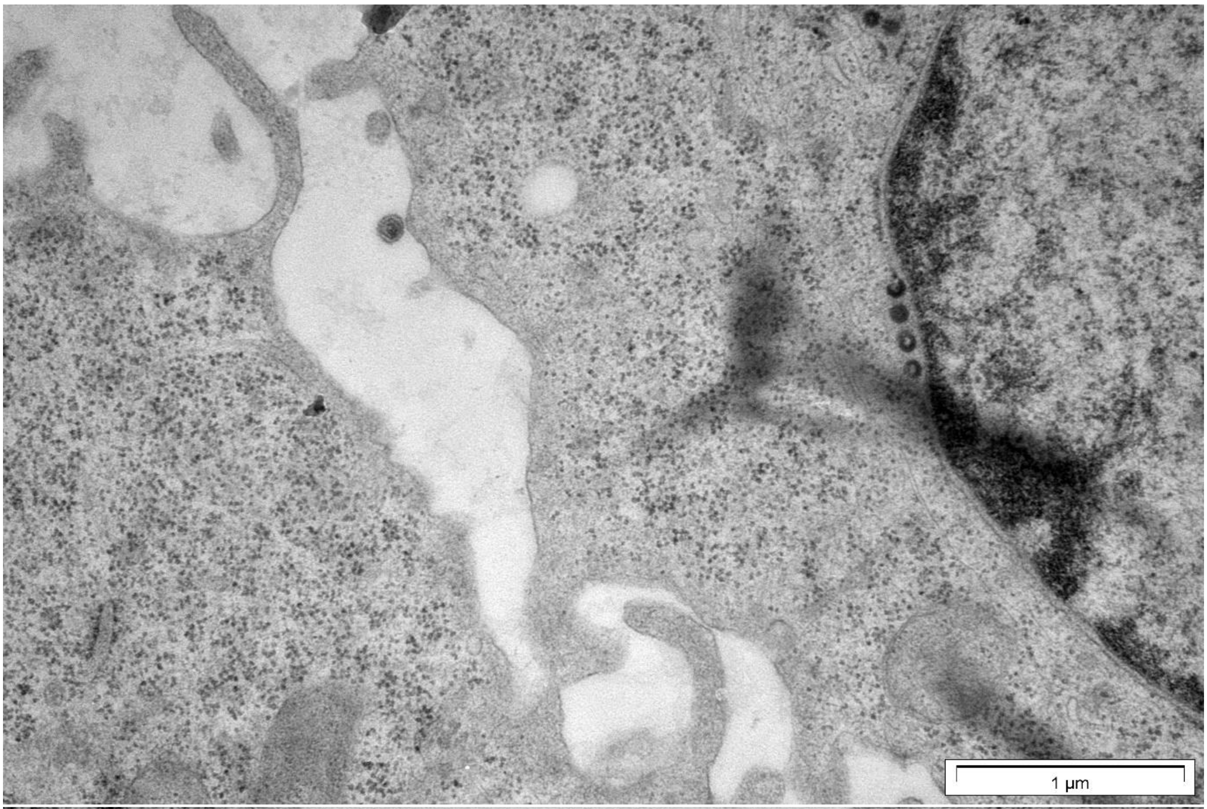
1 µm

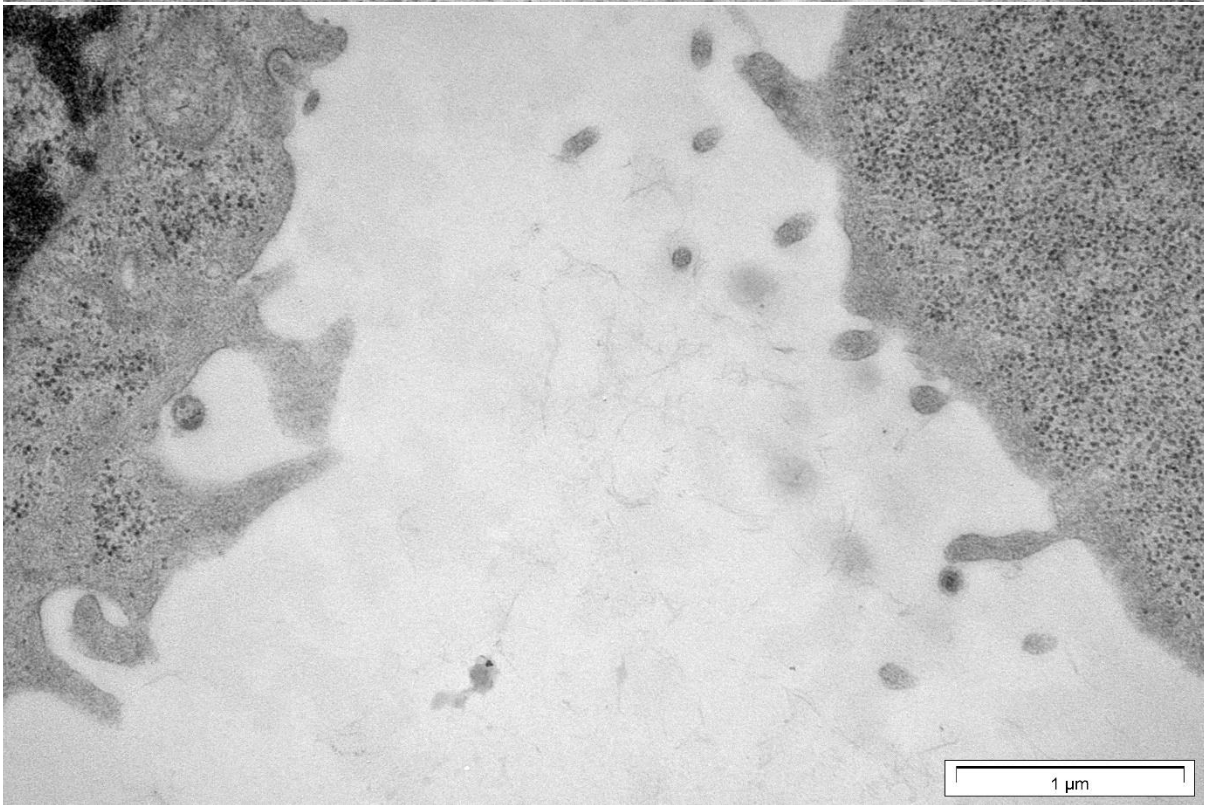
1 µm

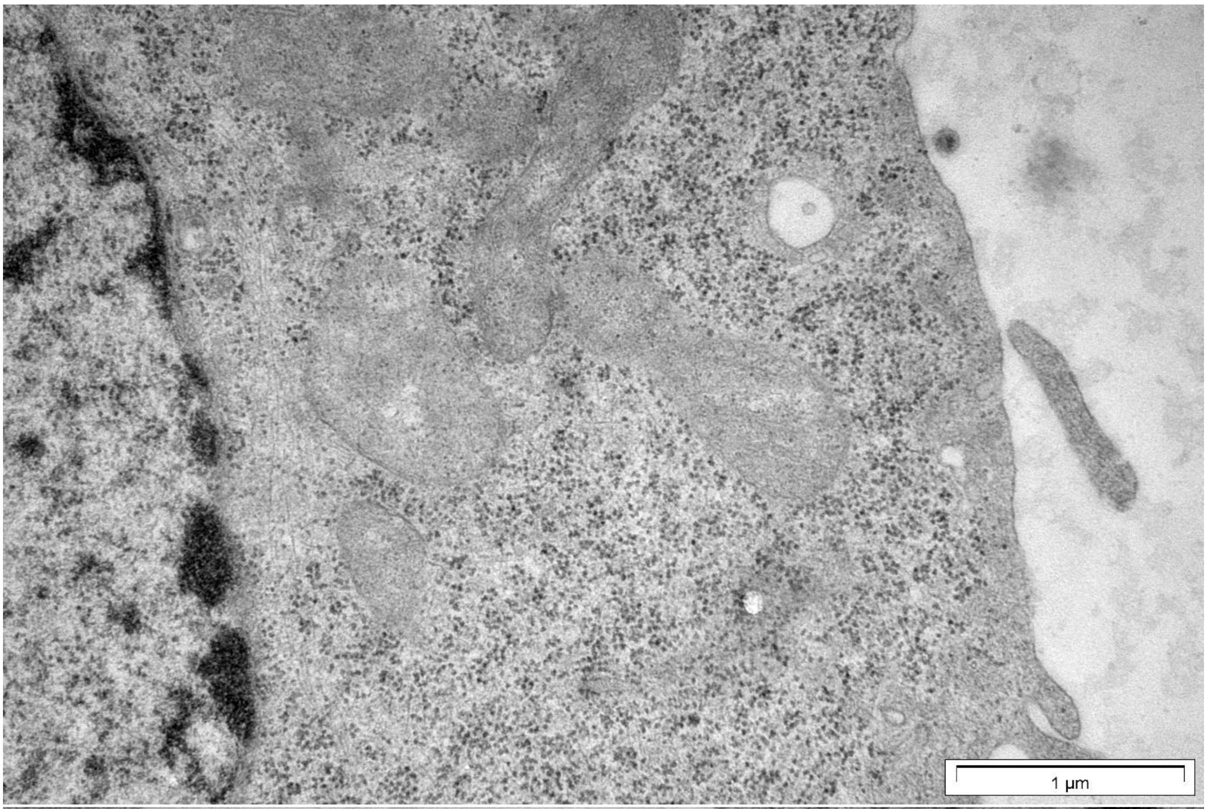
1 µm

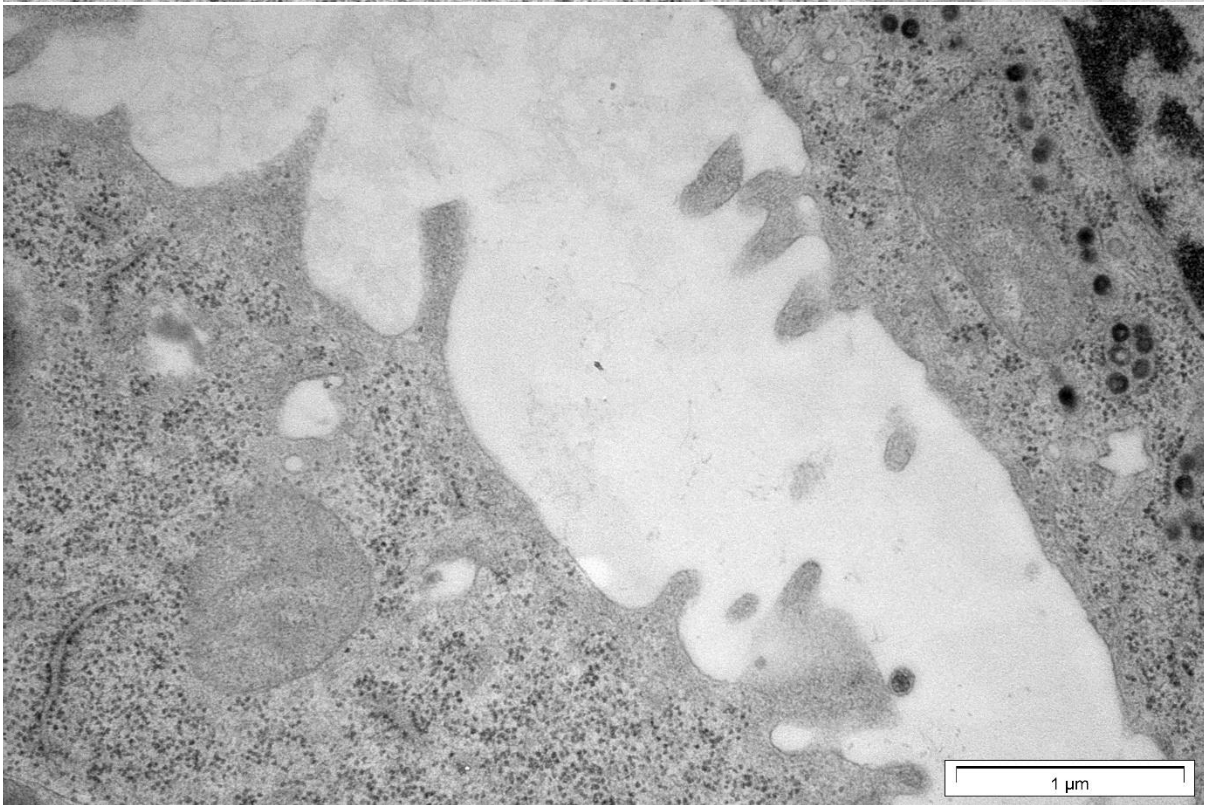
1 µm

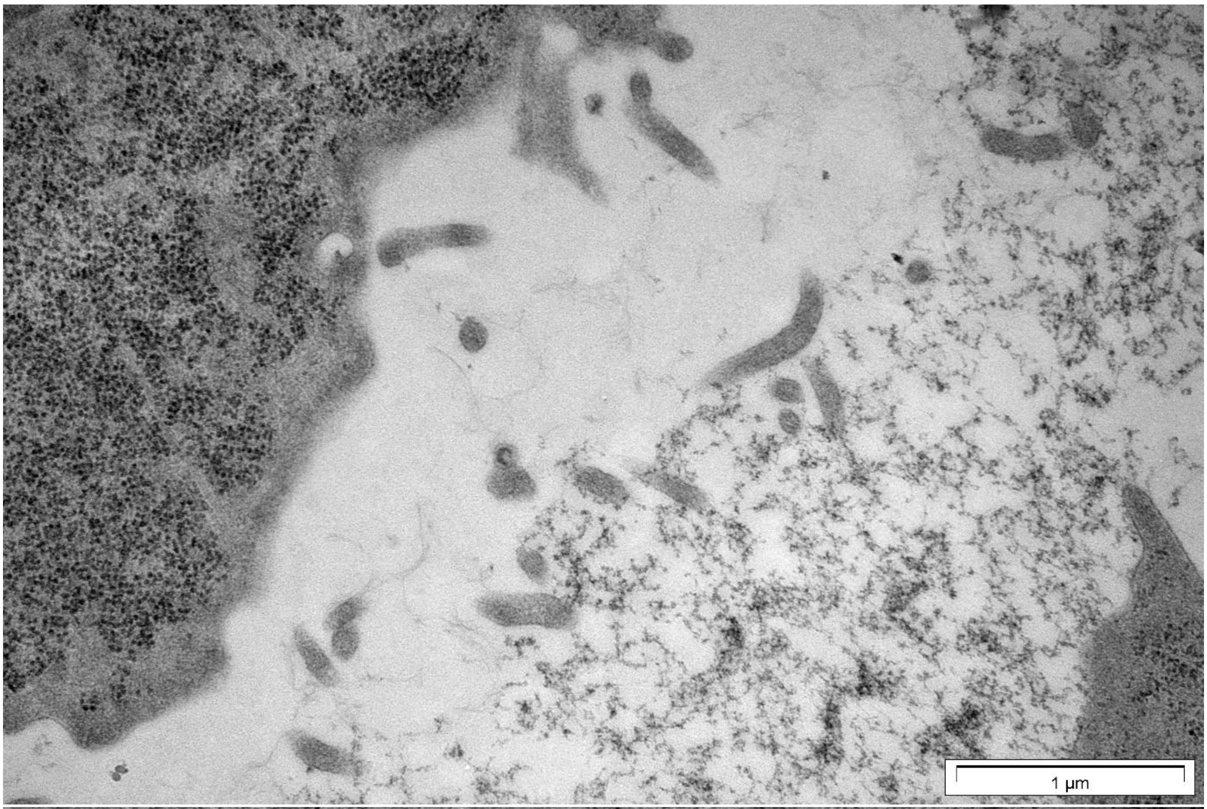
1 µm

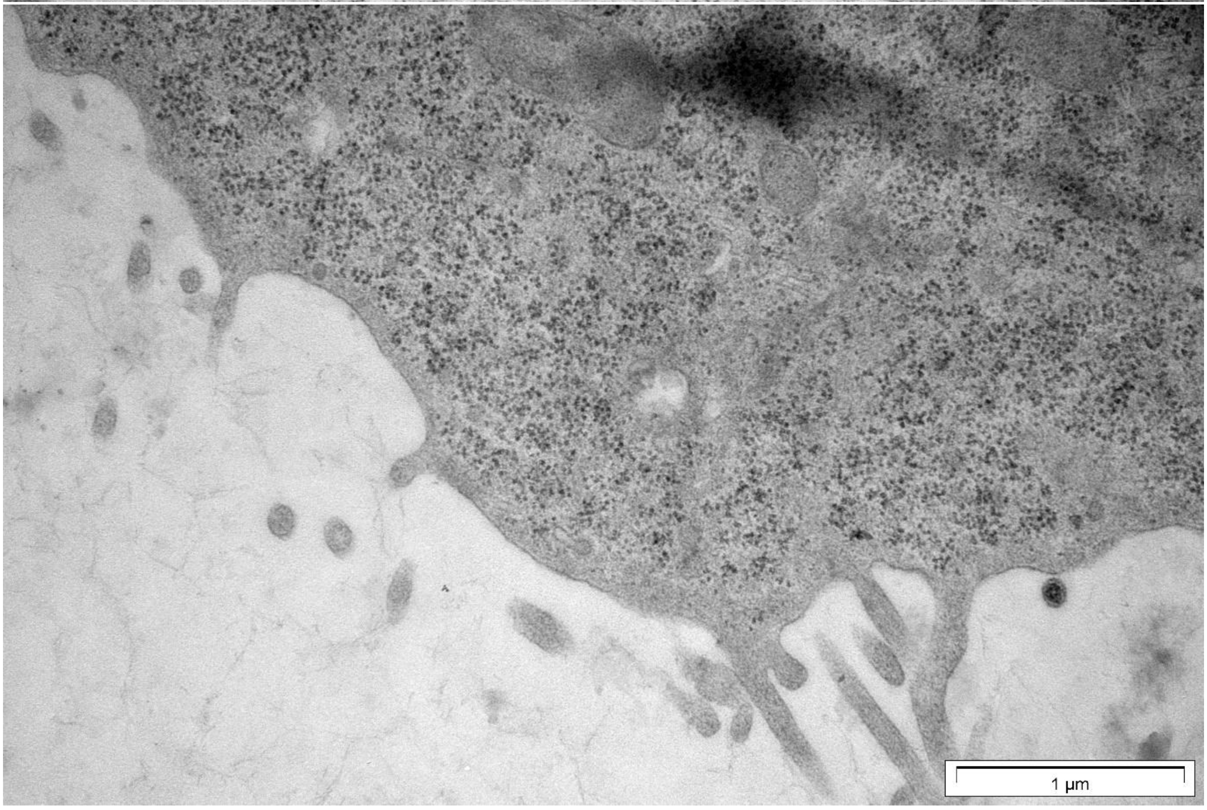
1 µm

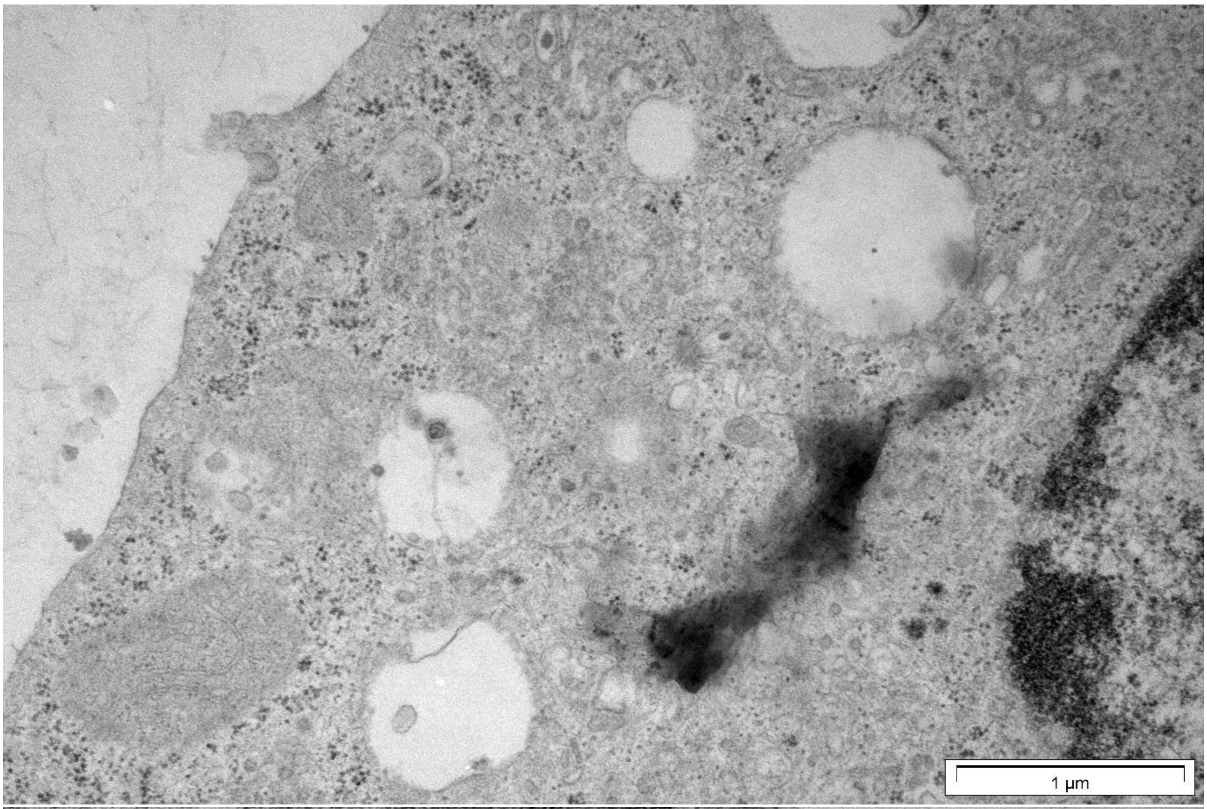
1 µm

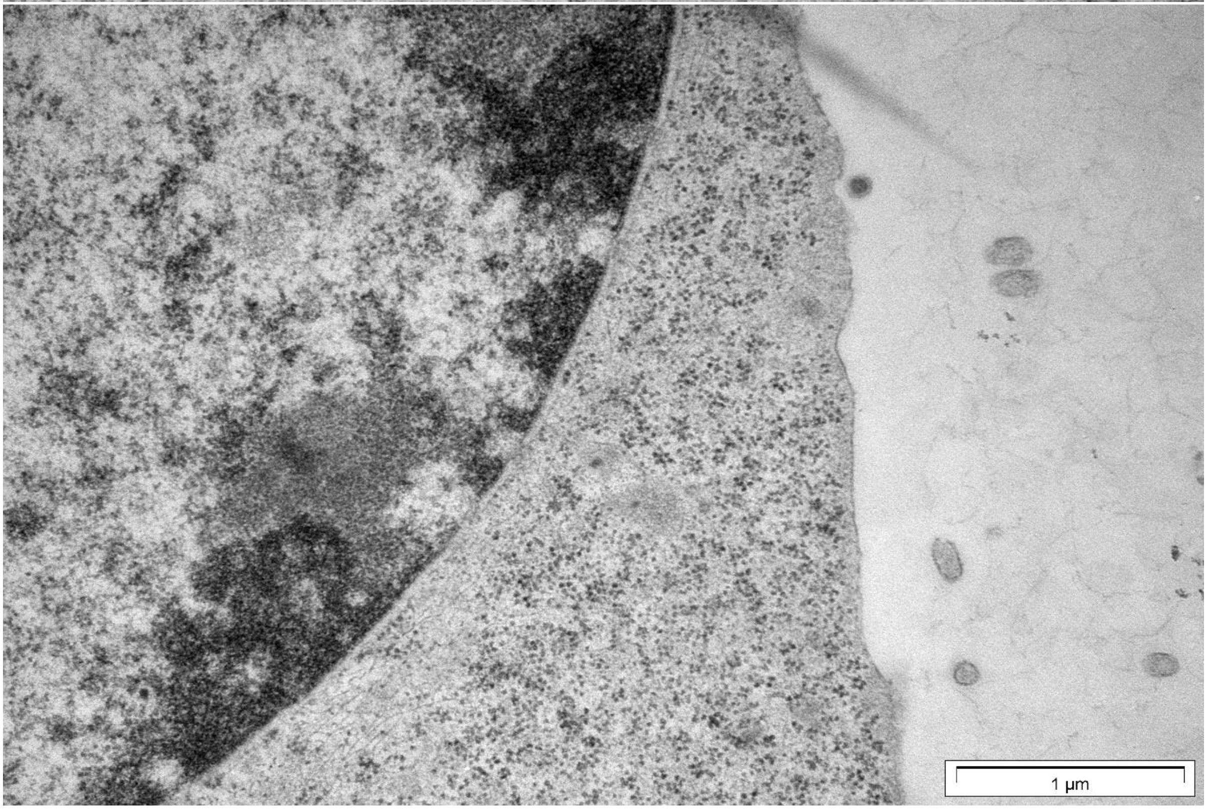
1 µm

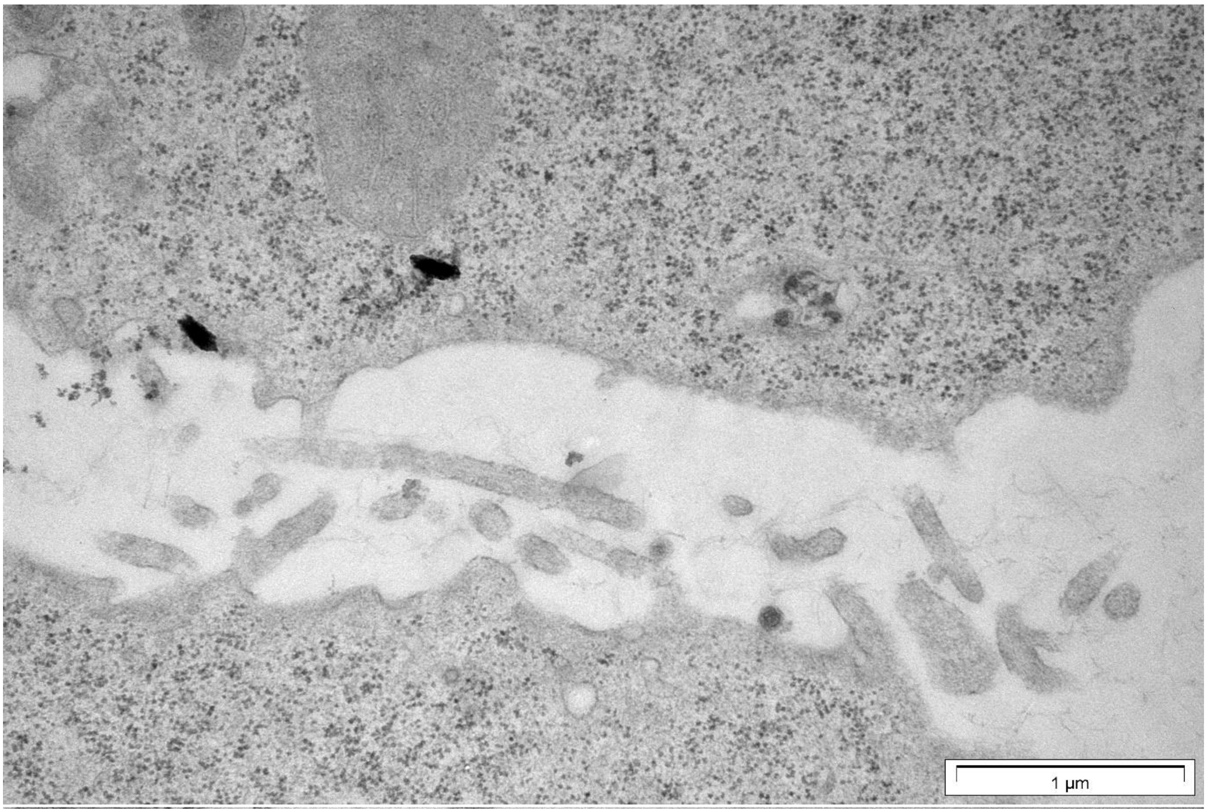
1 µm
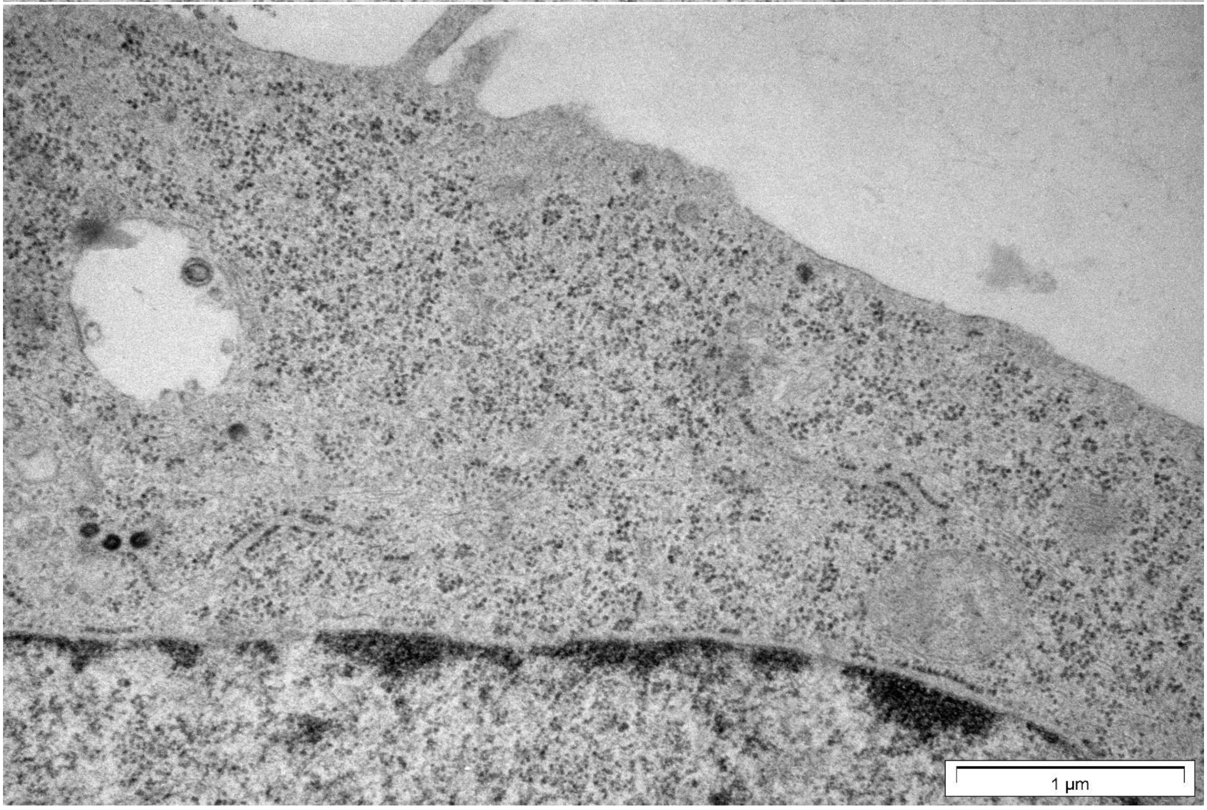
1 µm

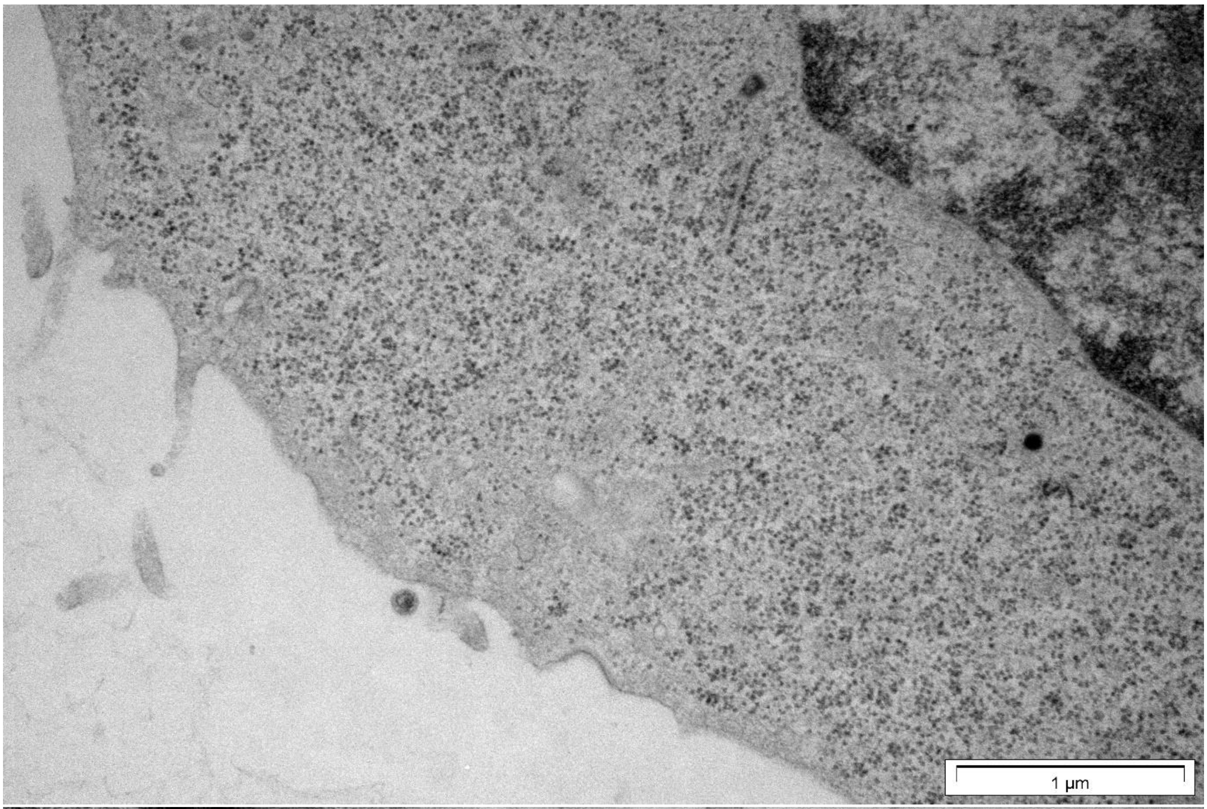
1 µm
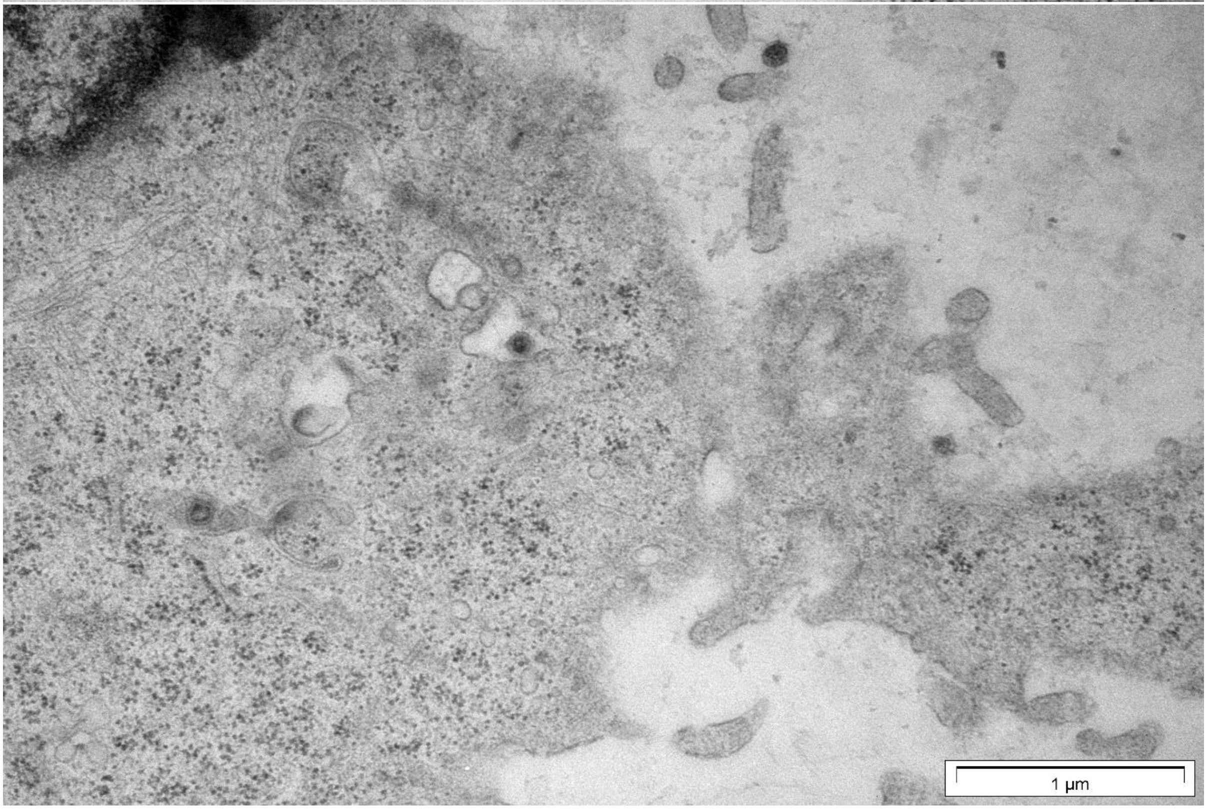
1 µm

Dextran, 15 min.

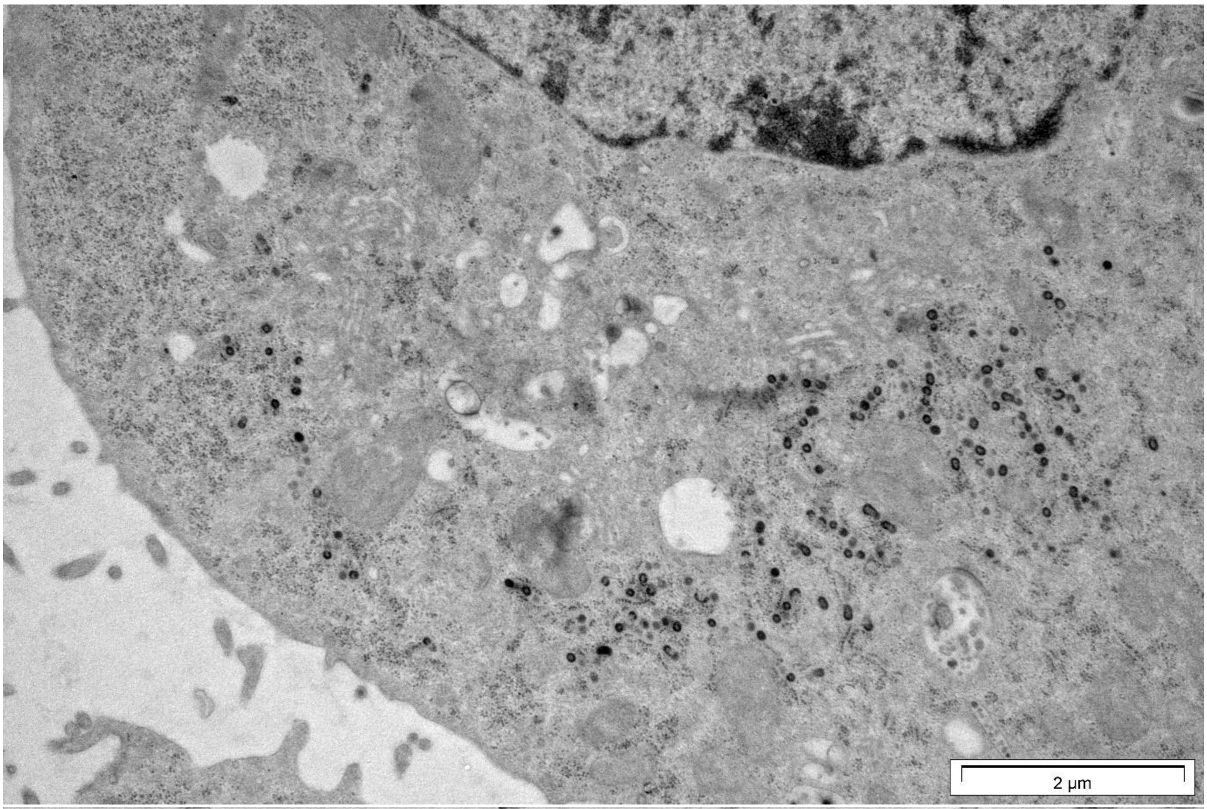

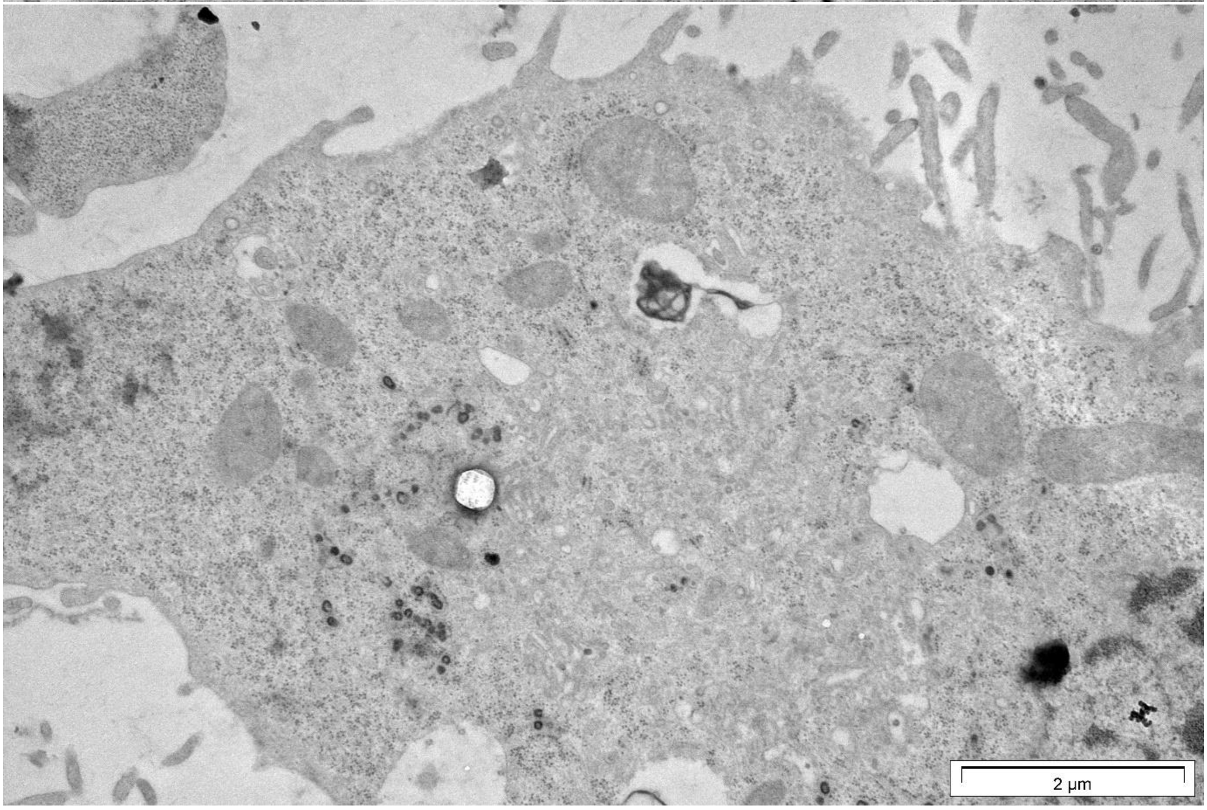

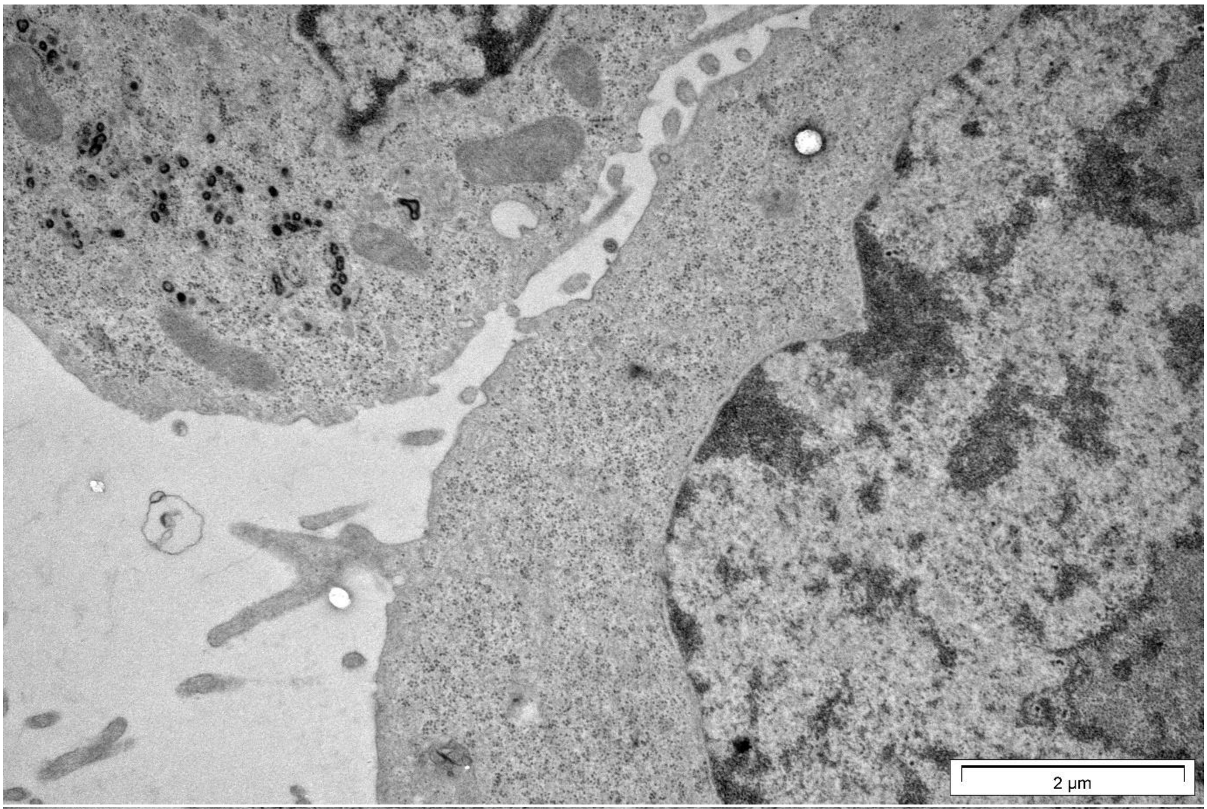
2 µm
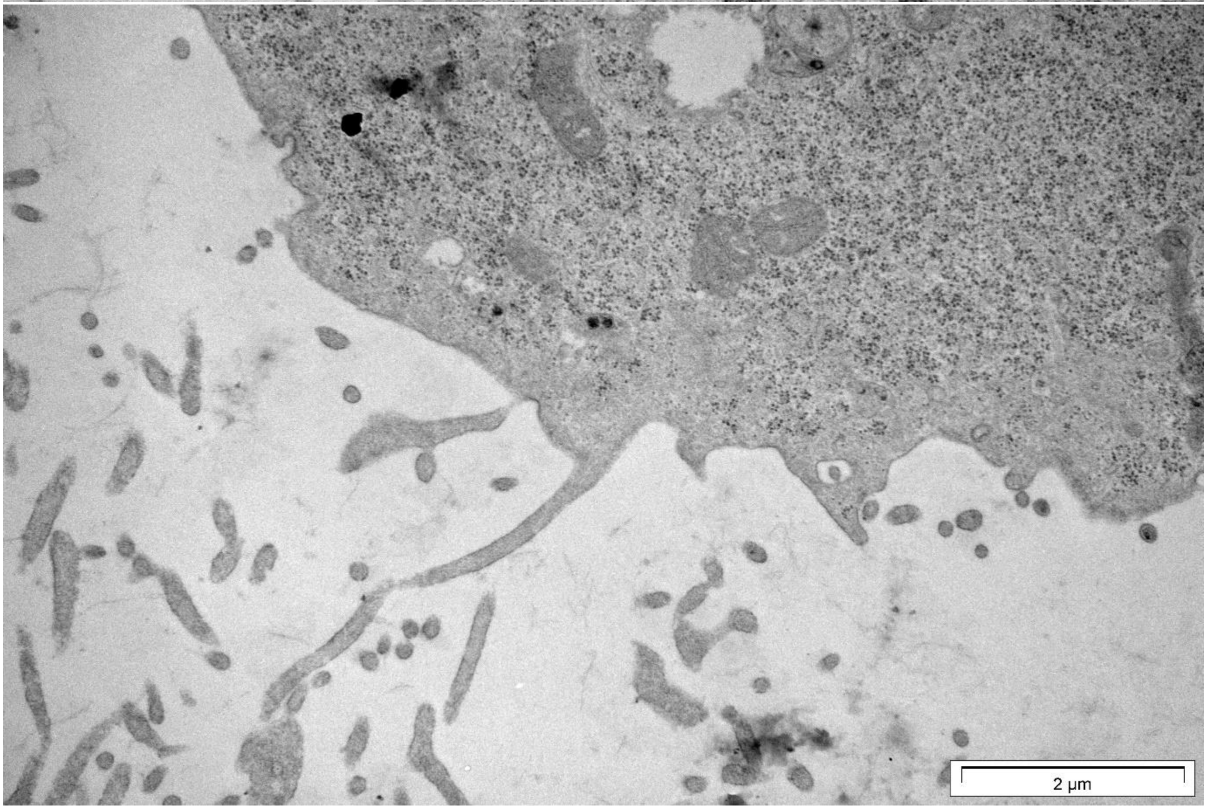
2 µm

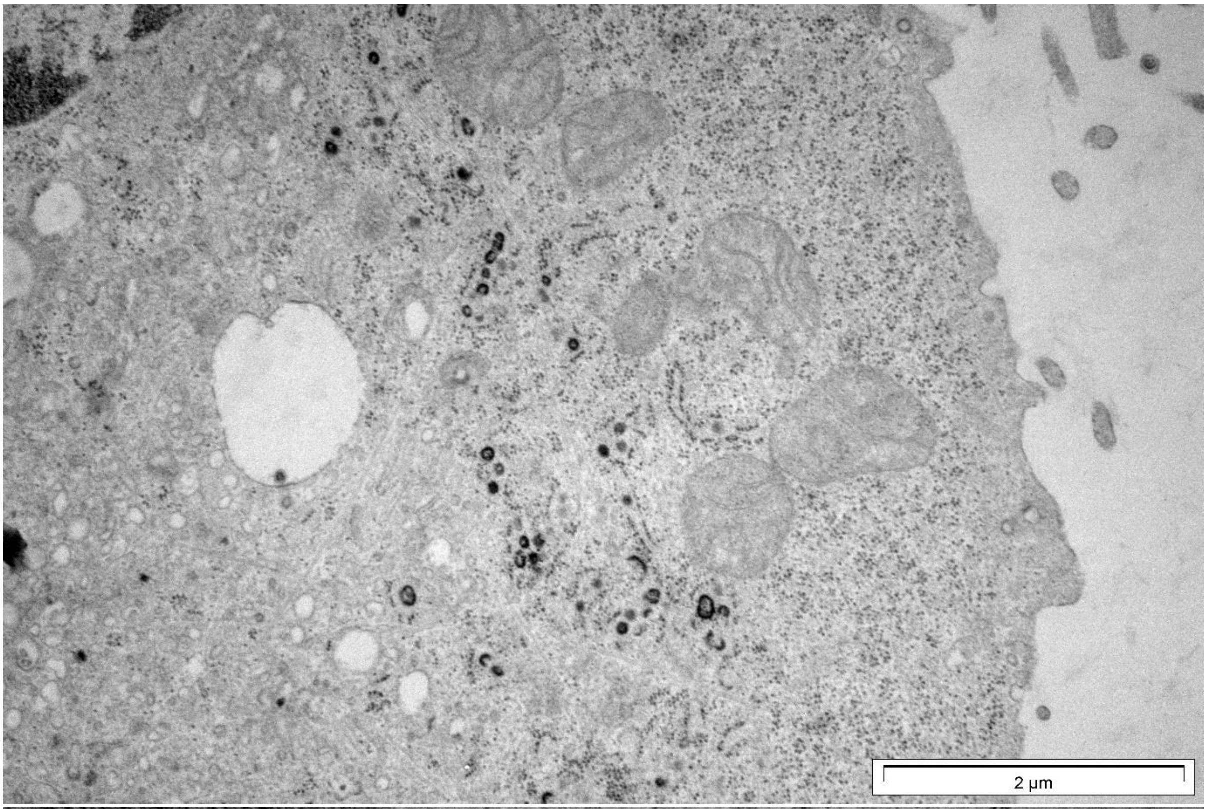
2 µm

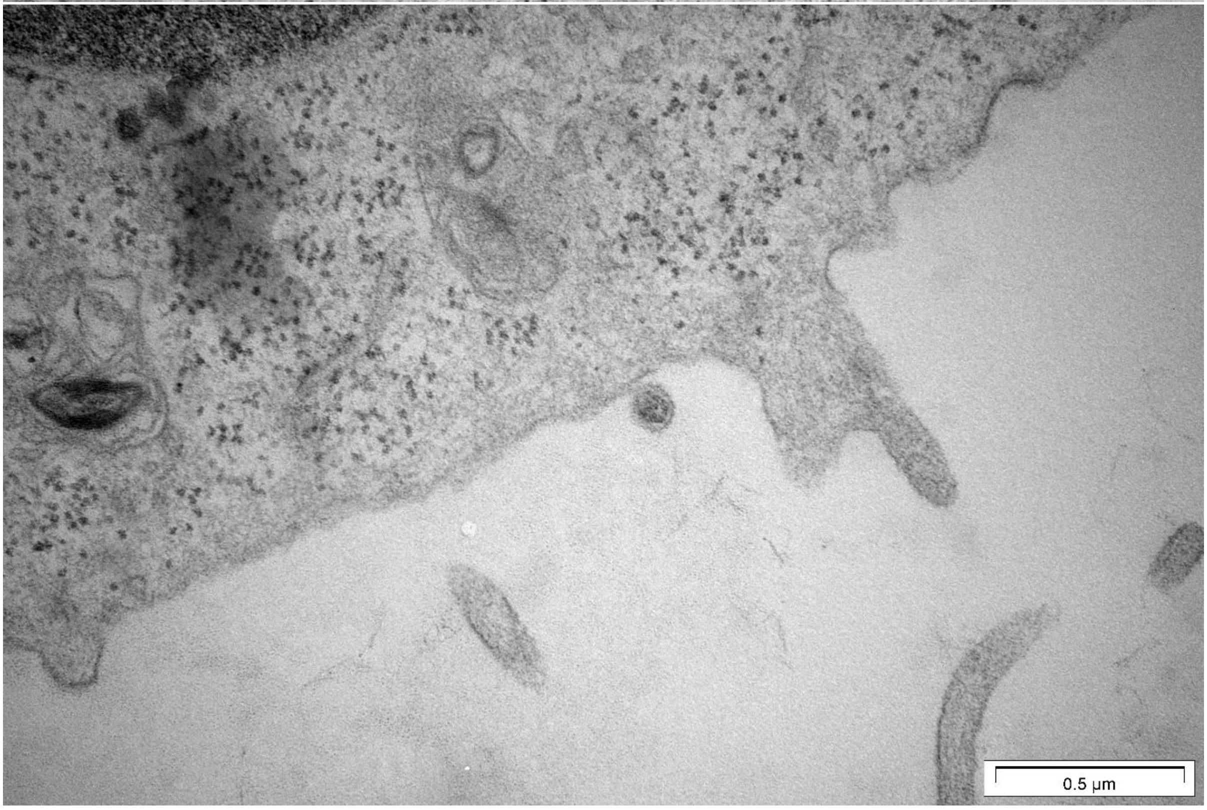
0.5 µm

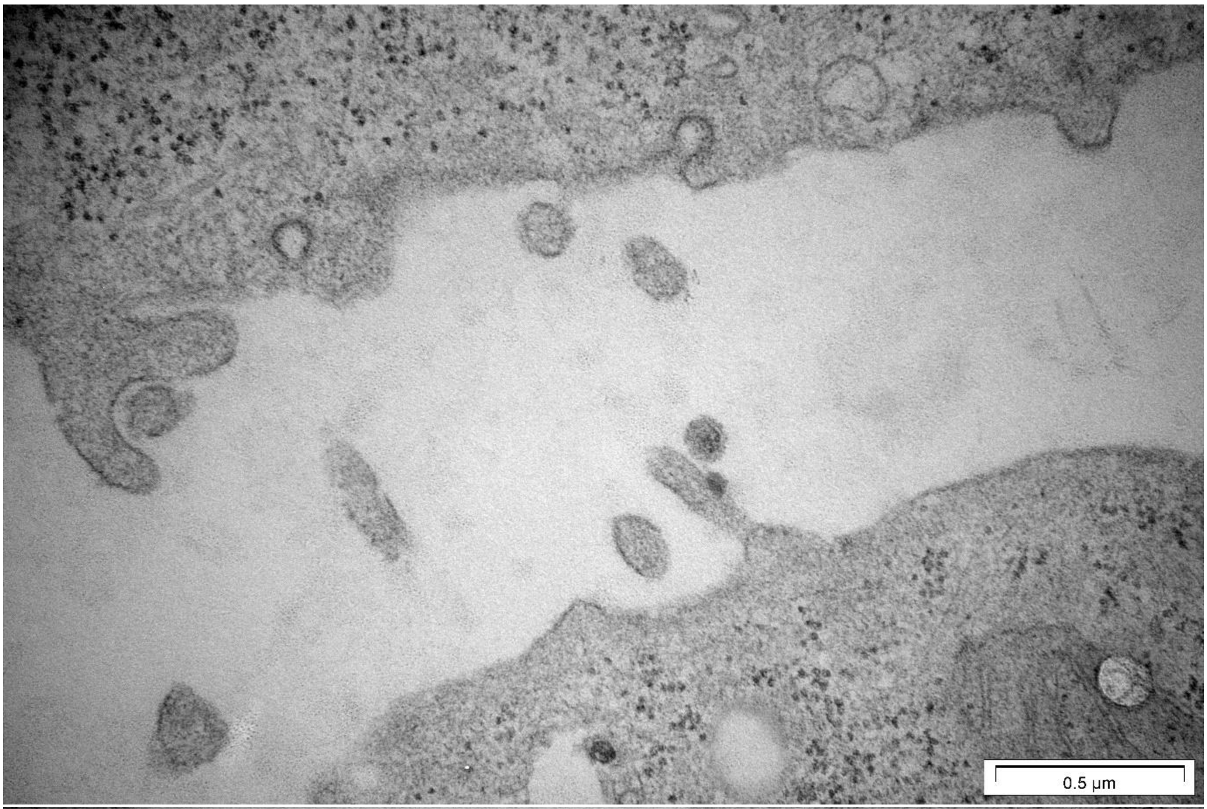
0.5 µm

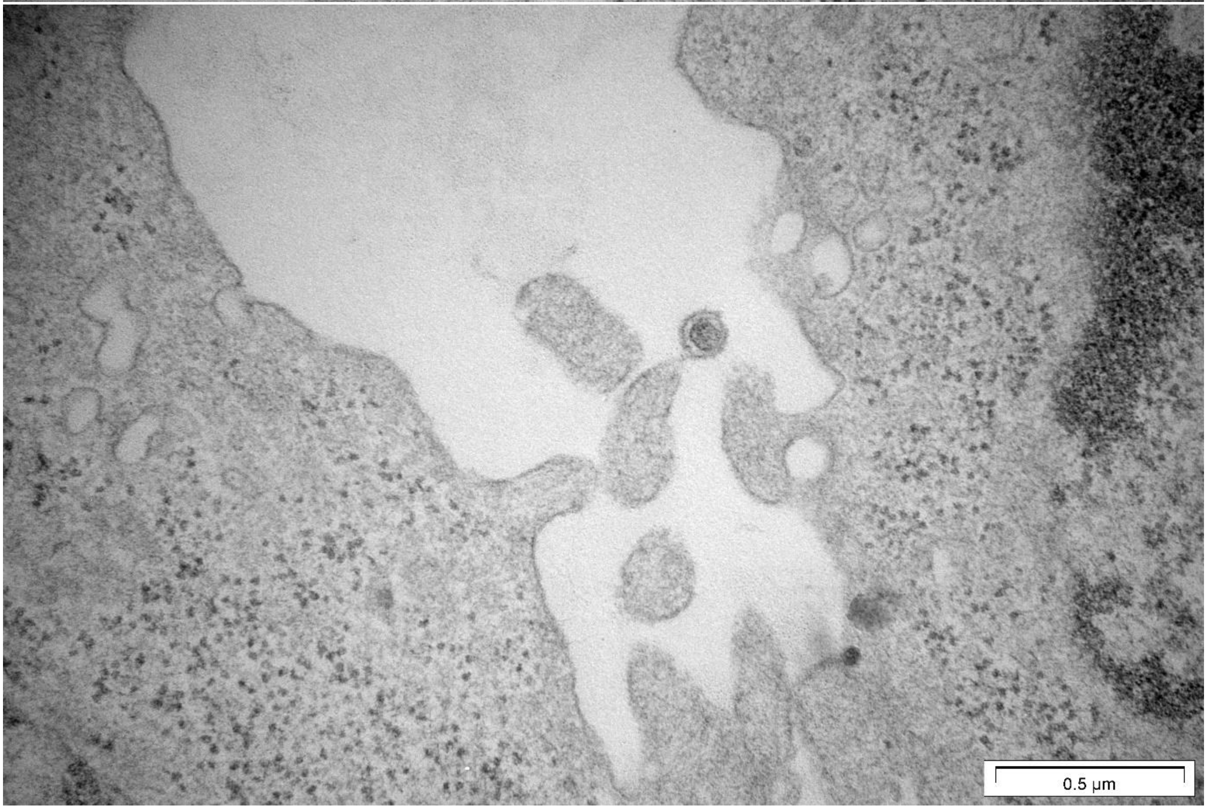
0.5 µm

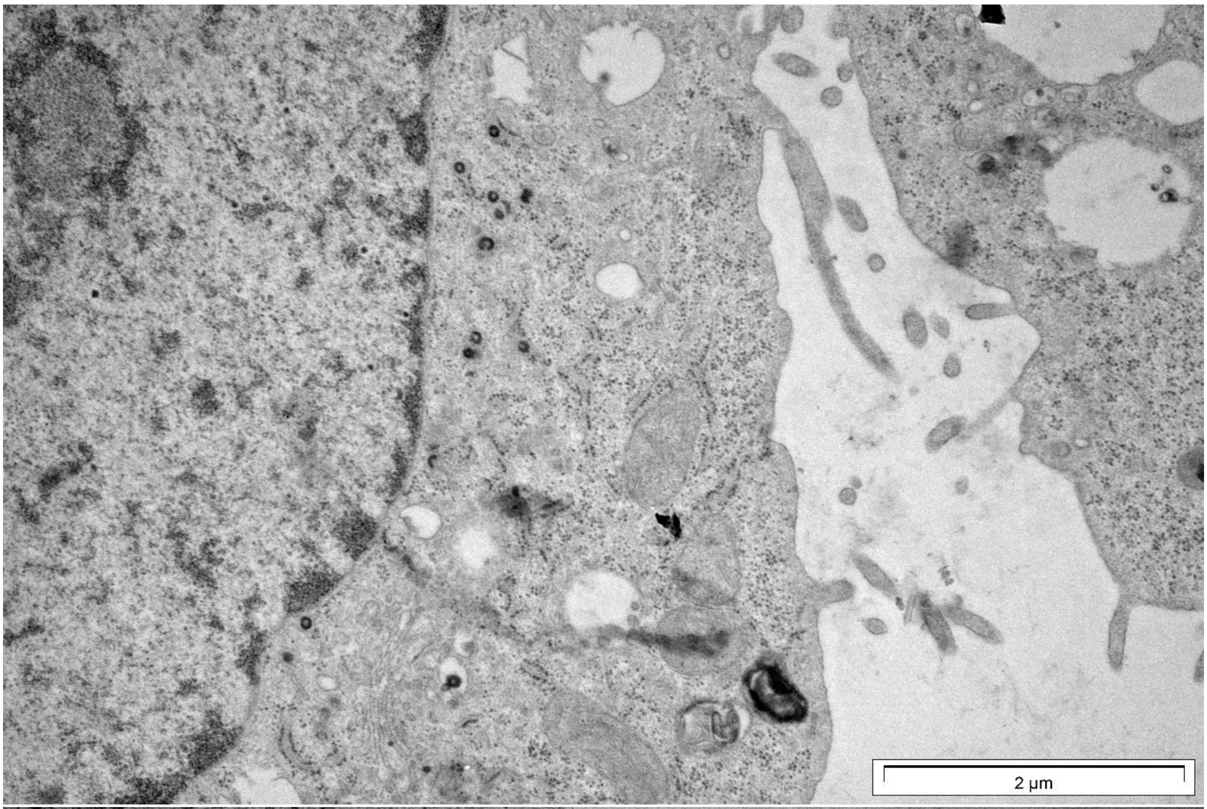
2 µm

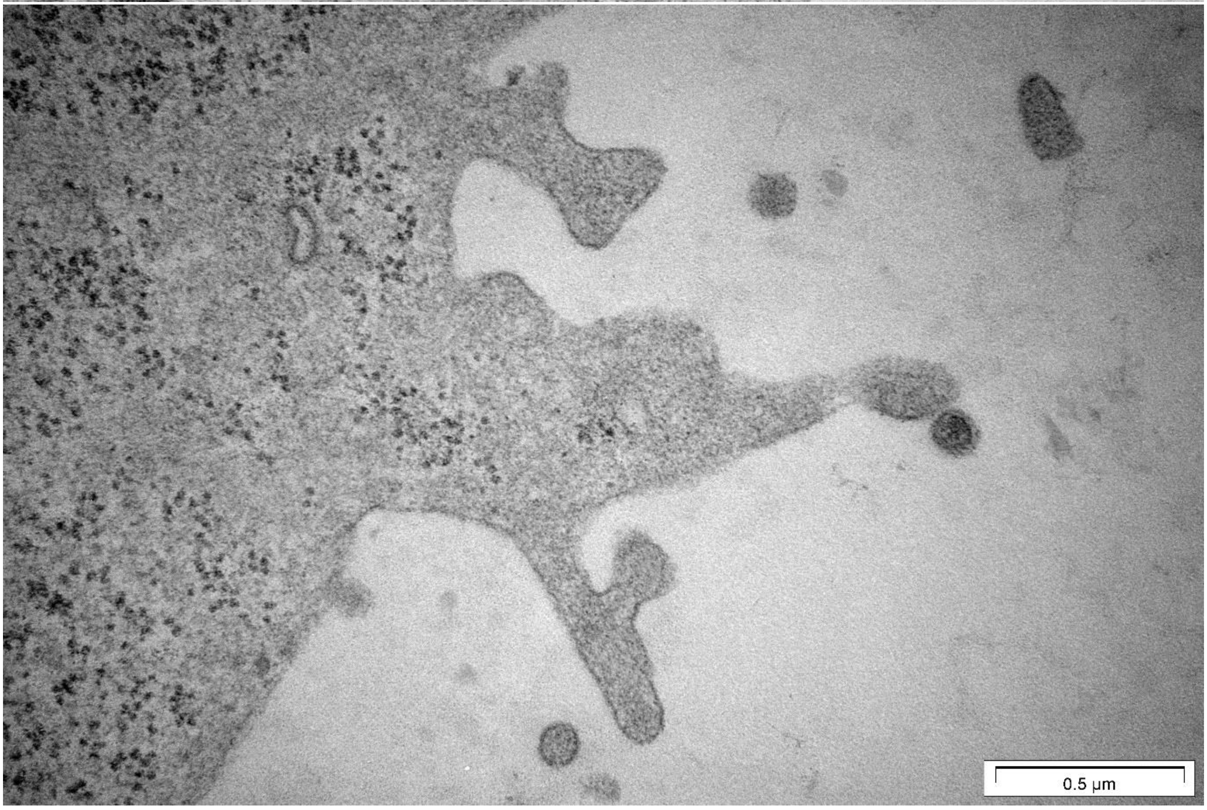
0.5 µm

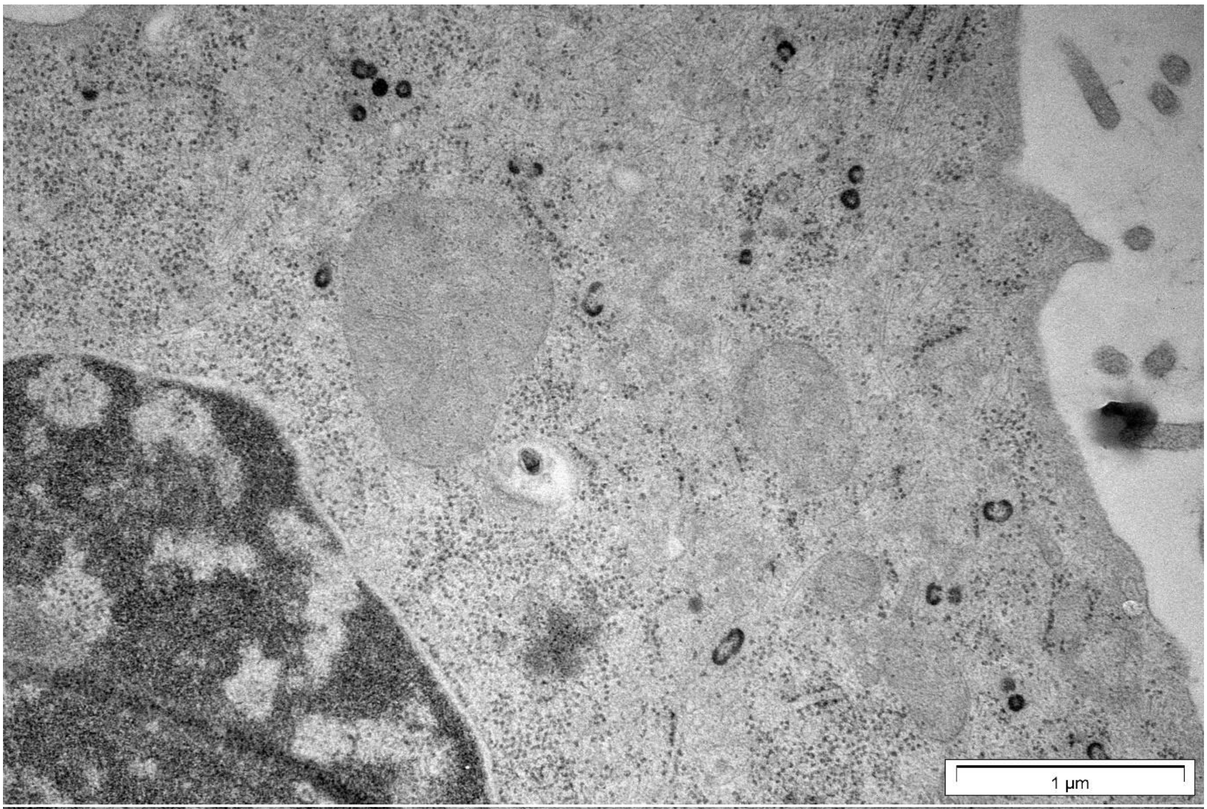
1 µm
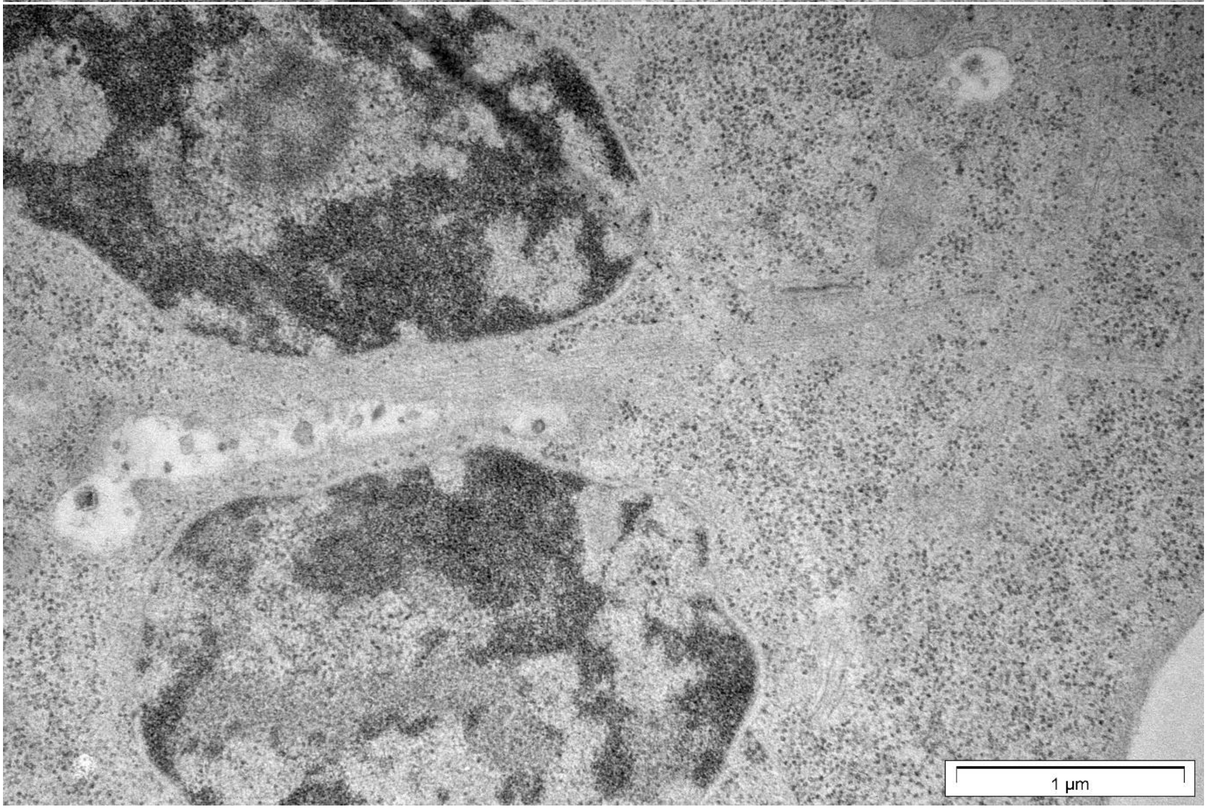
1 µm

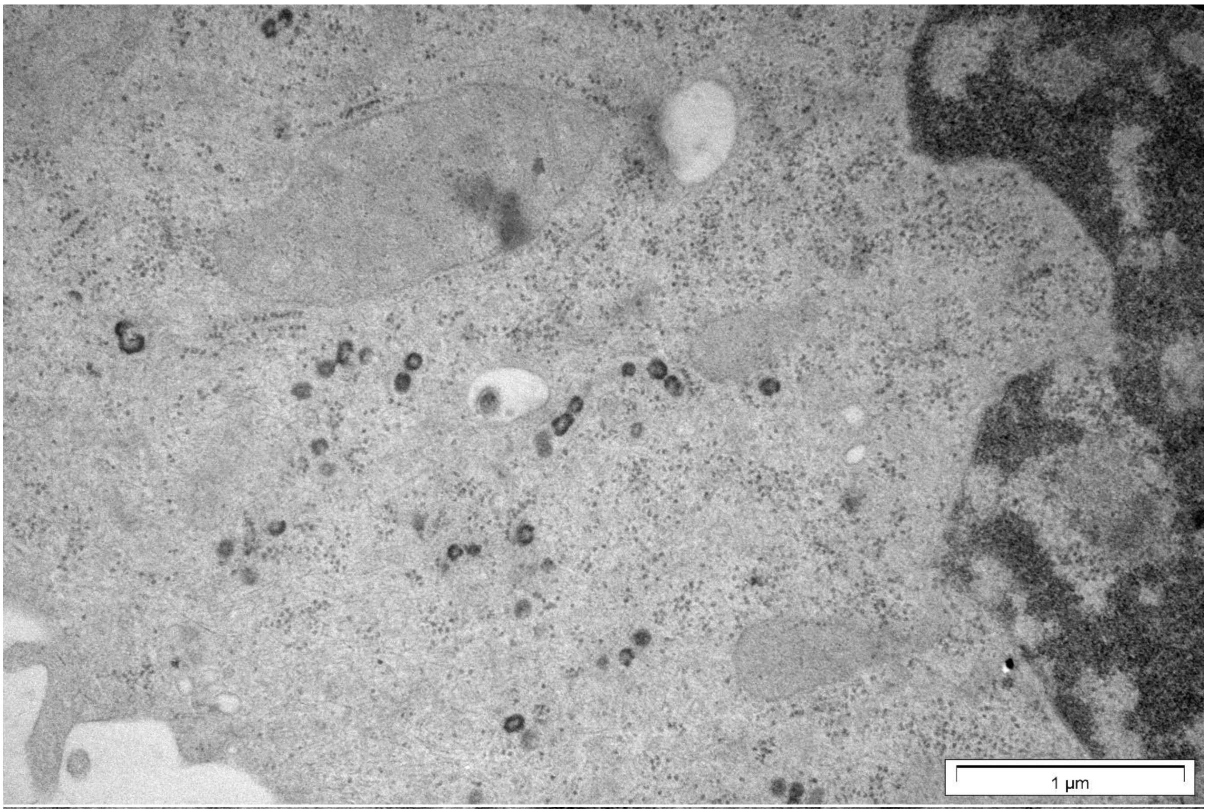
1 µm

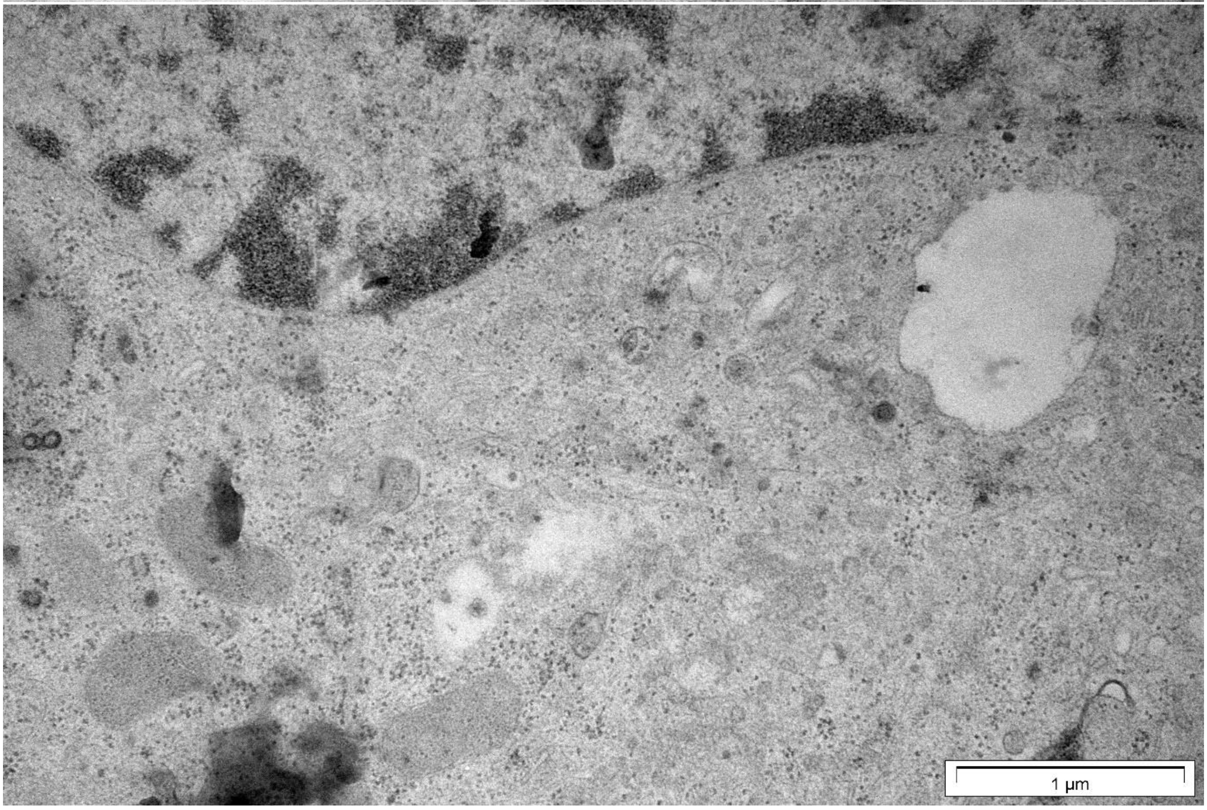
1 µm

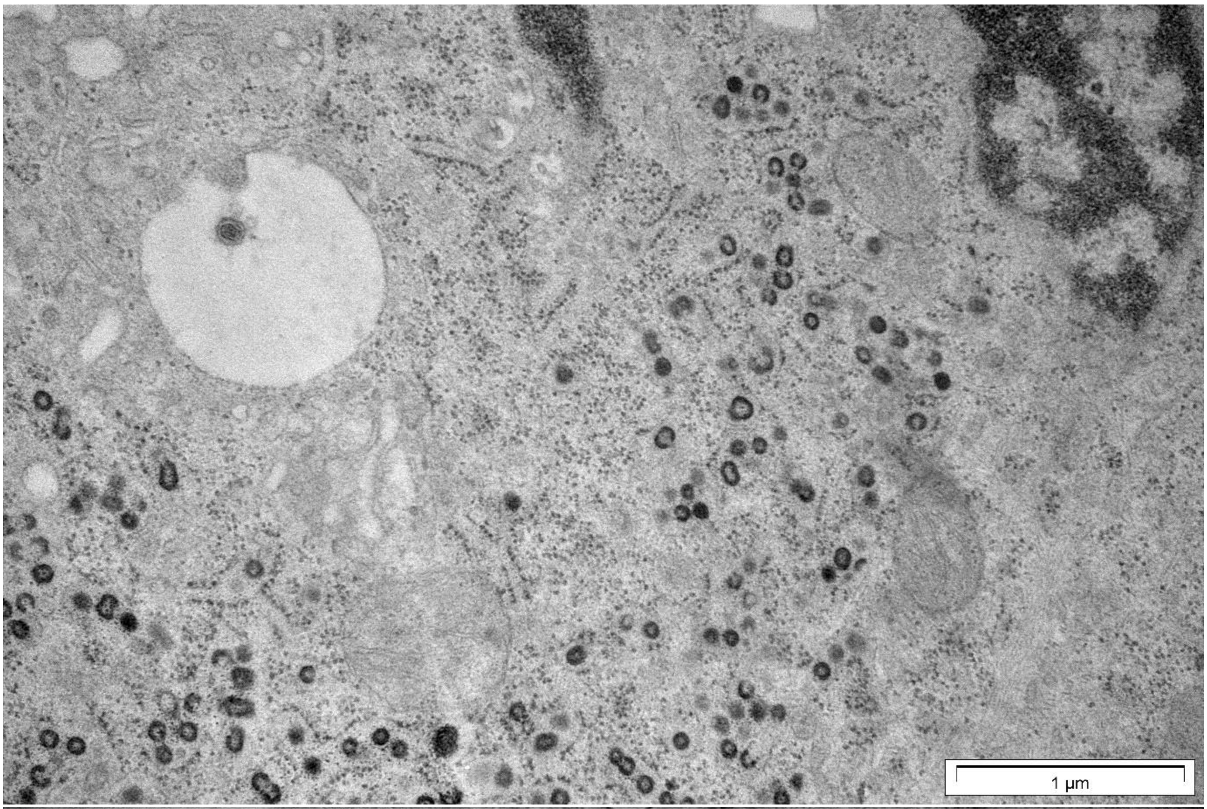
1 µm

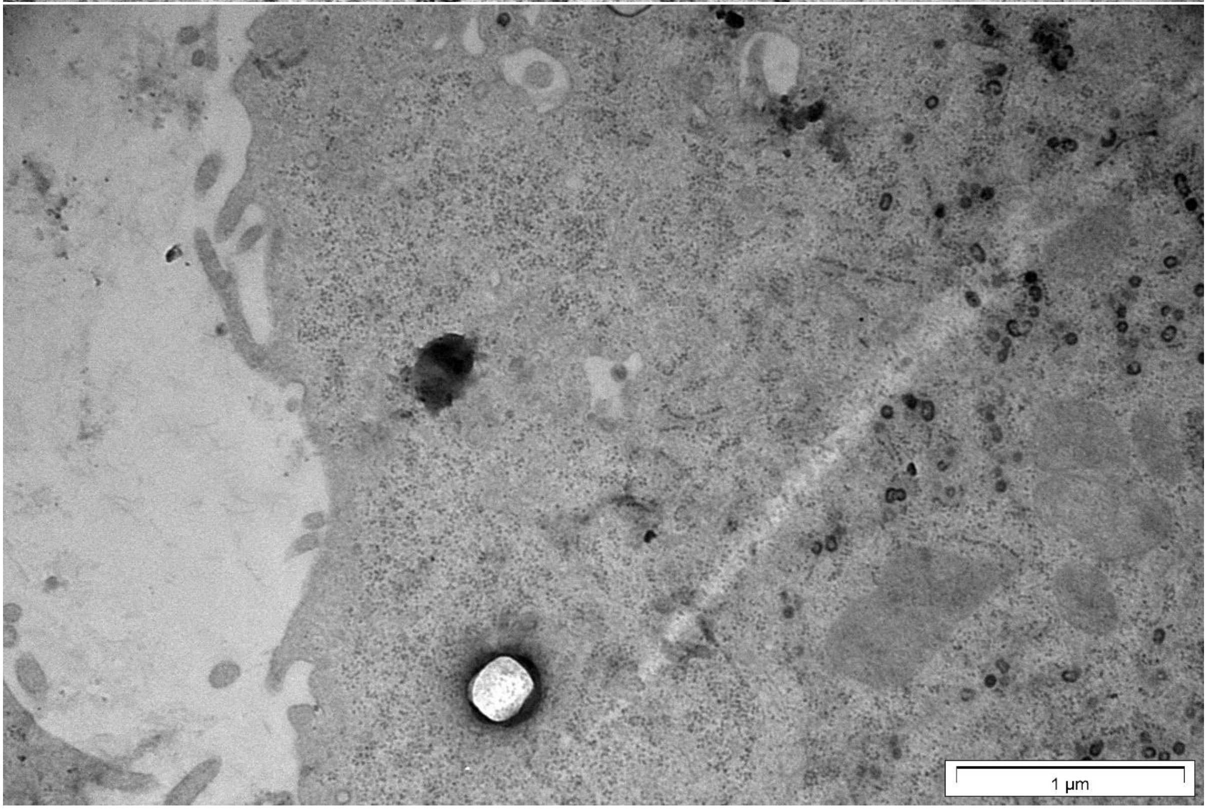
1 µm

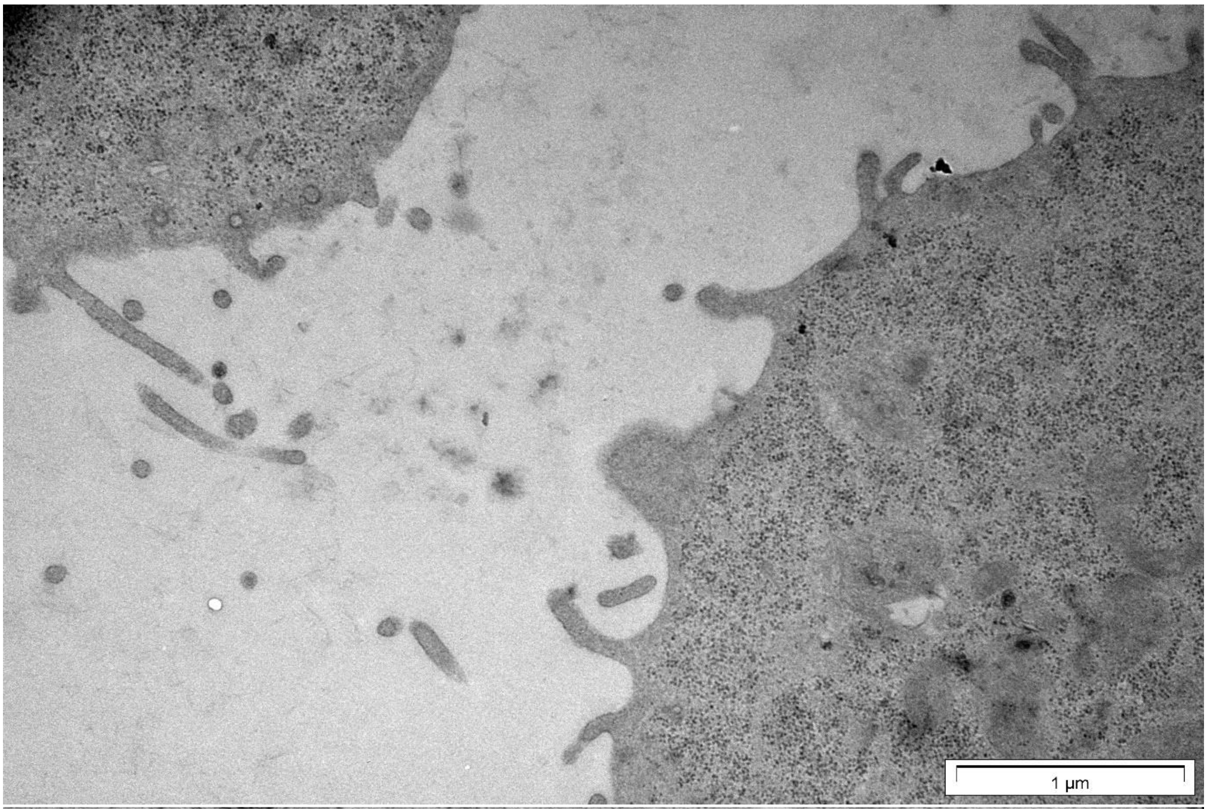
1 µm

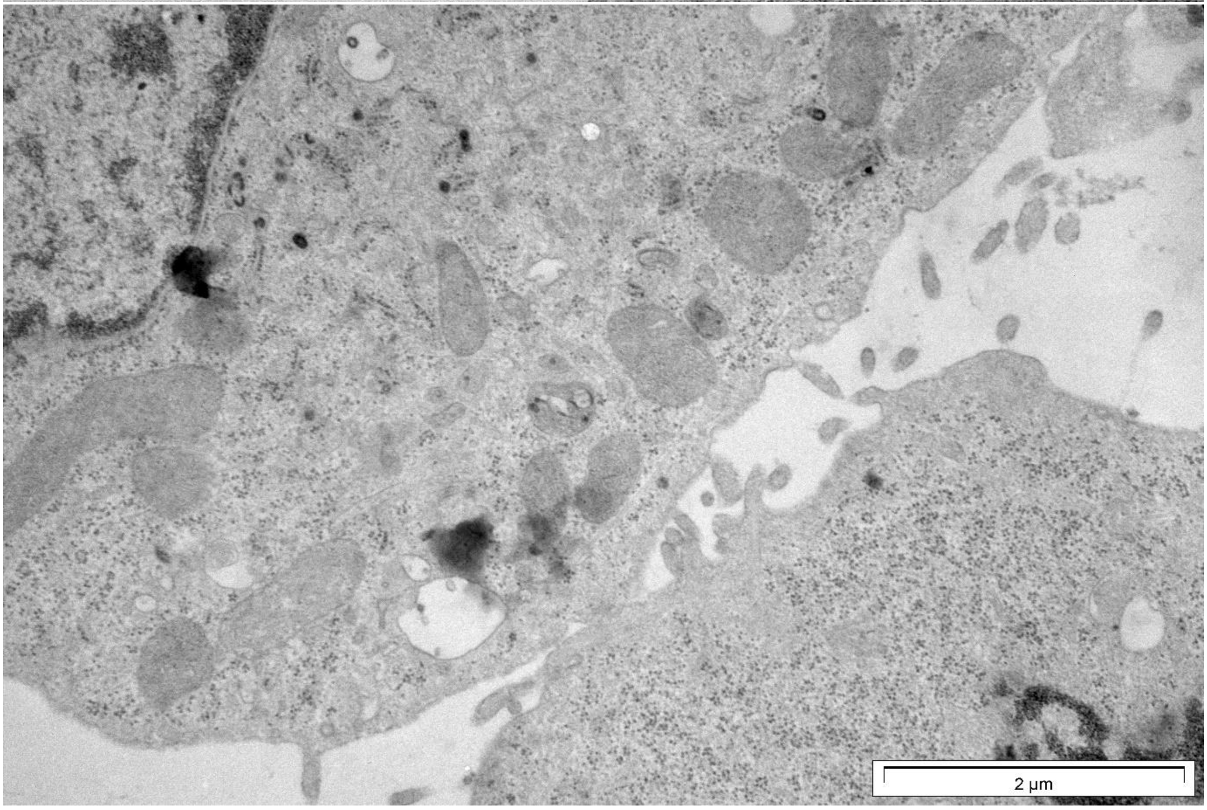
2 µm

Dextran sulfate, 1 min.

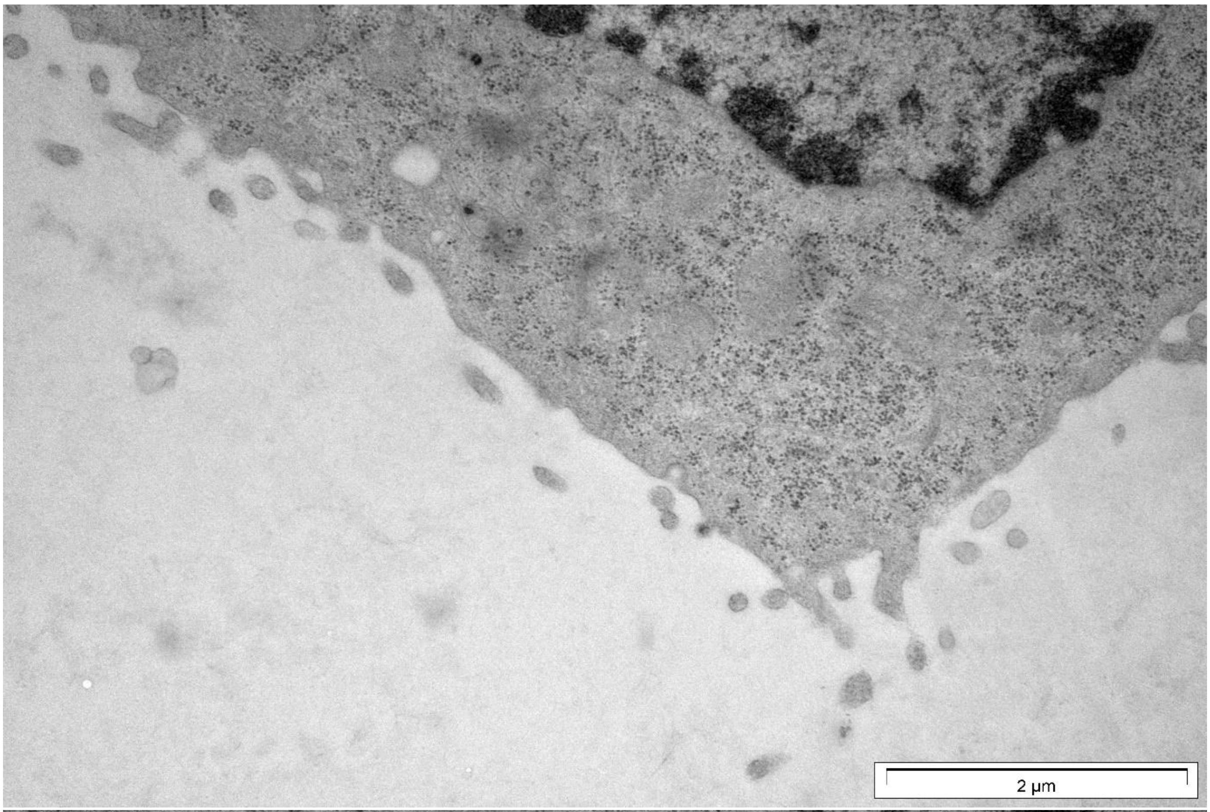

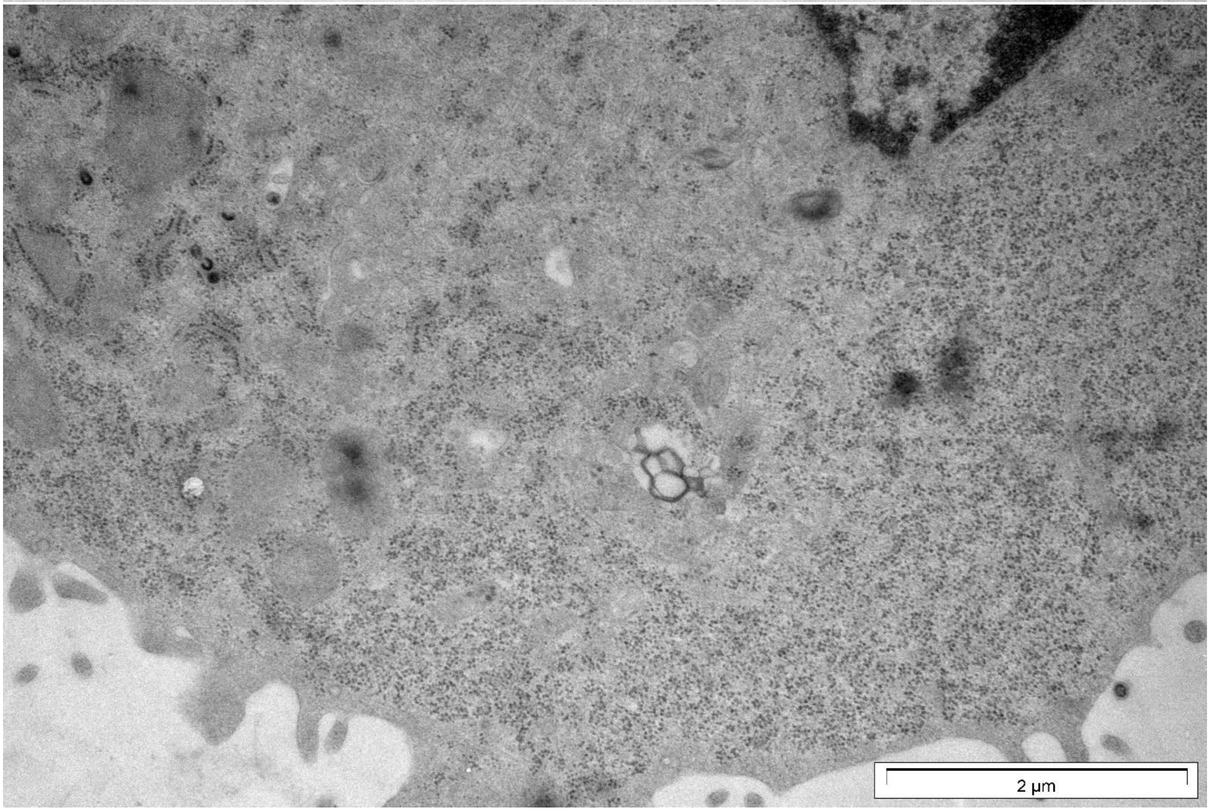

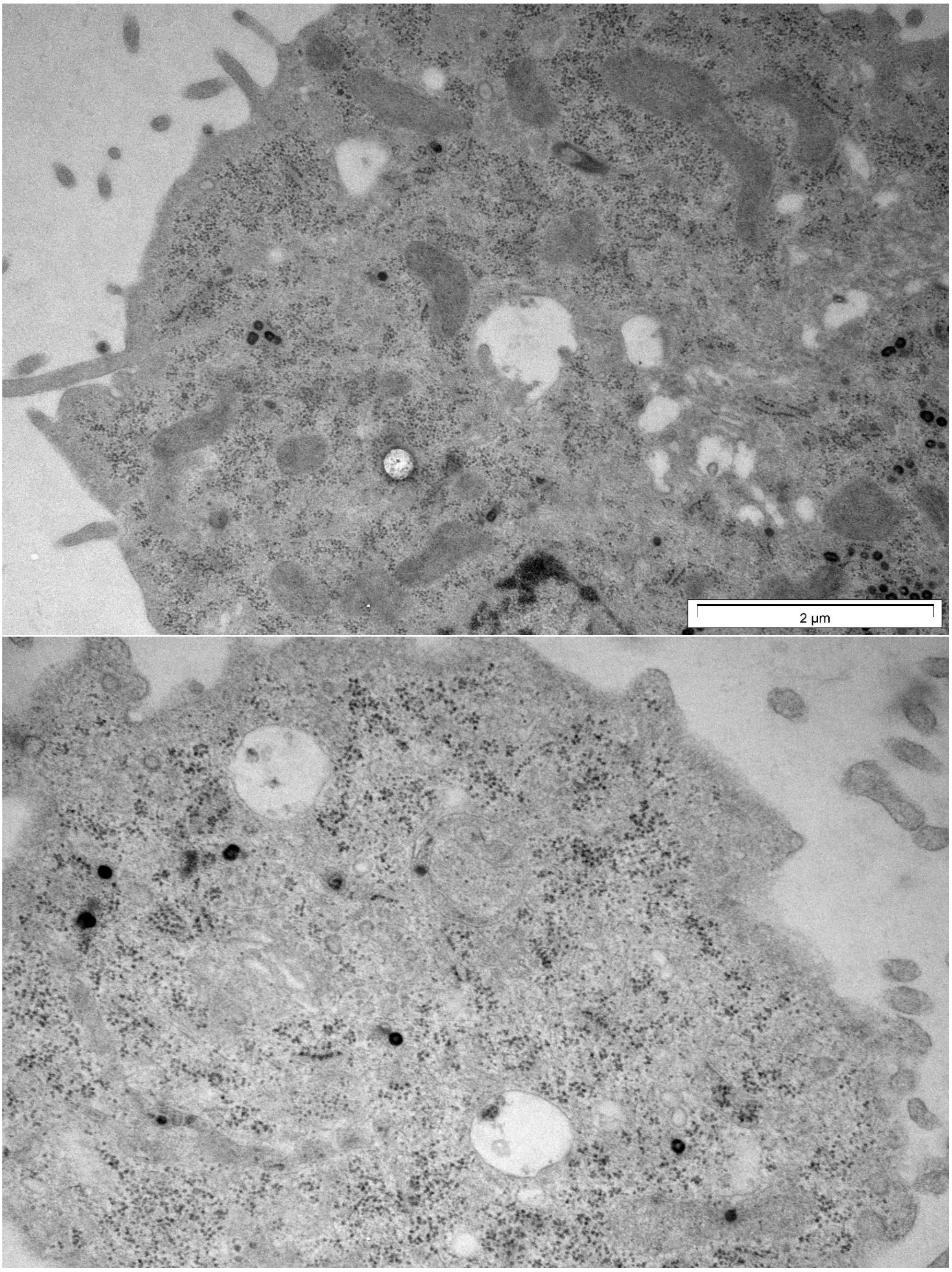
2 µm

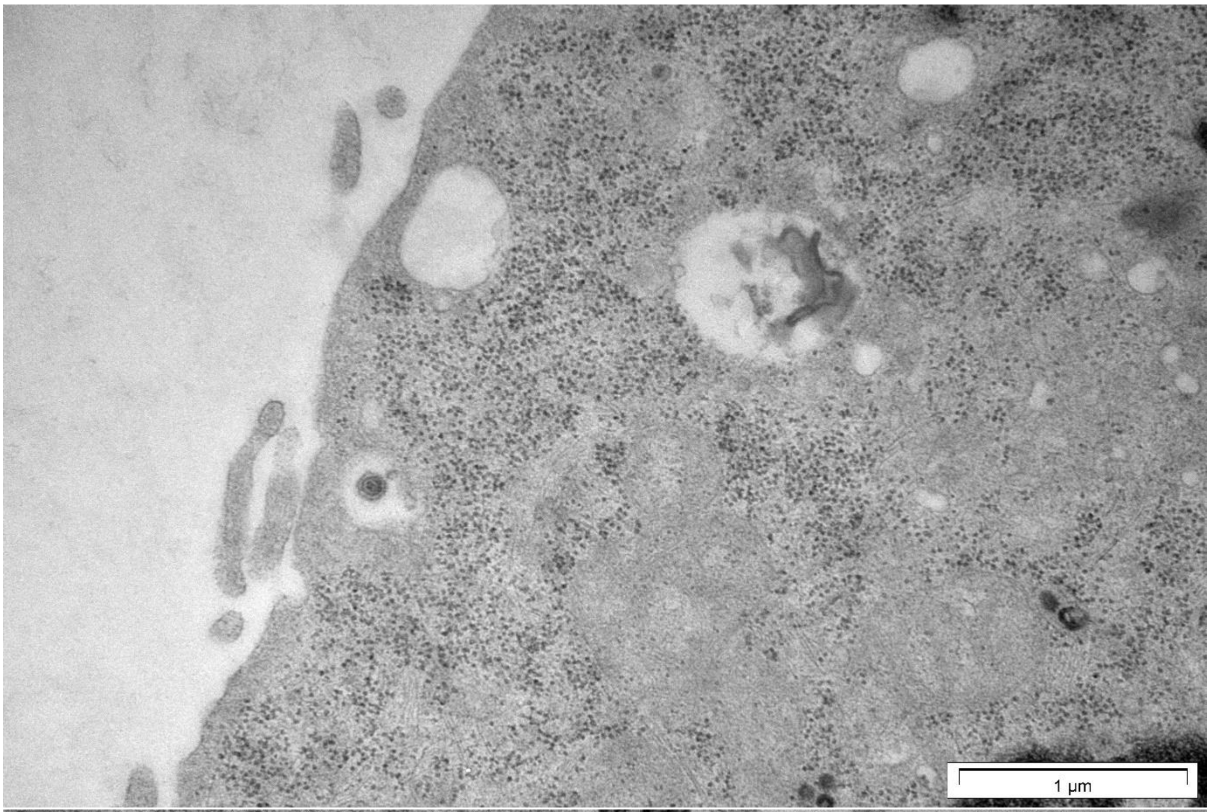
1 µm

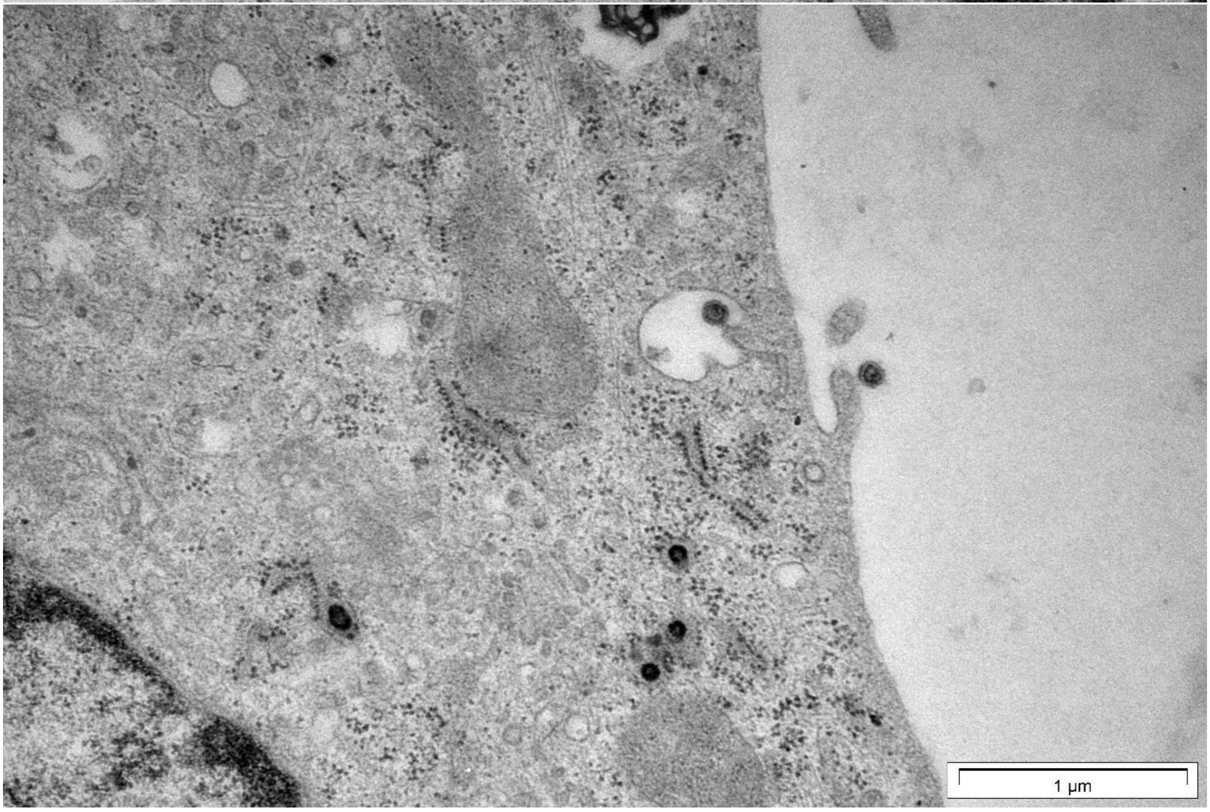
1 µm

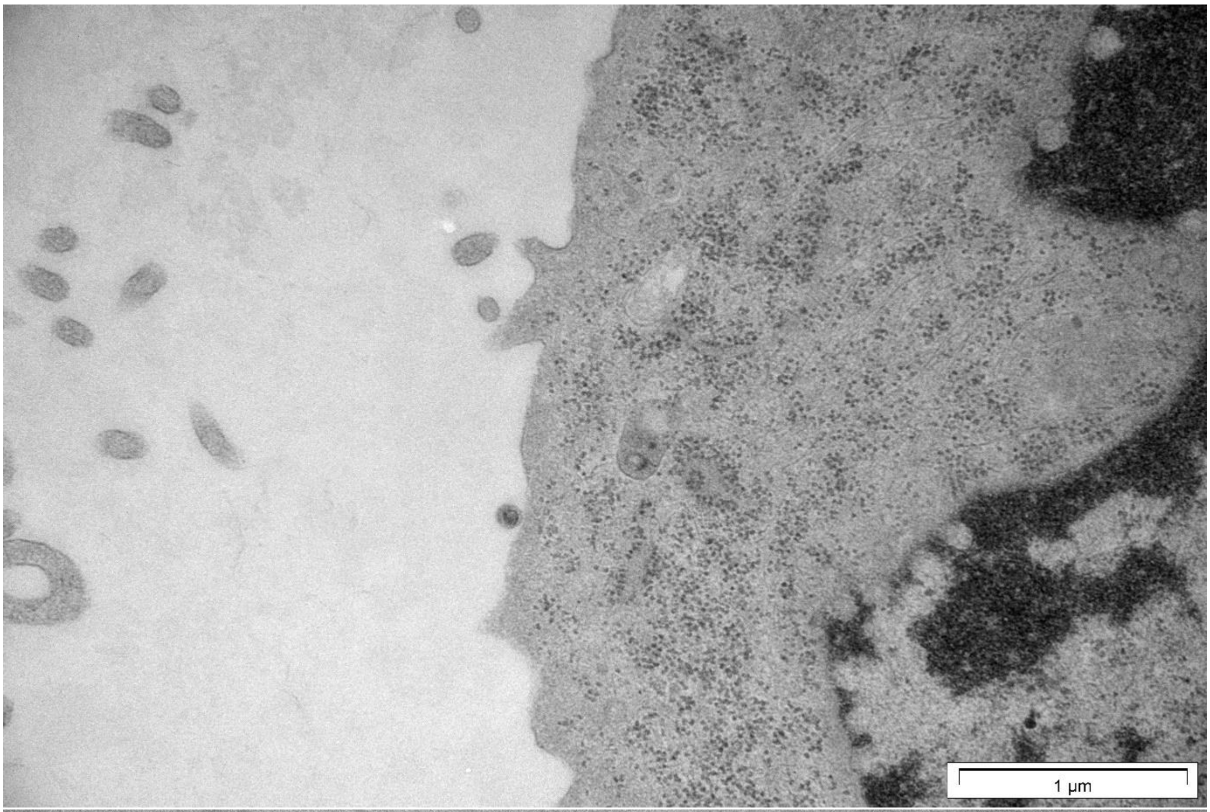
1 µm

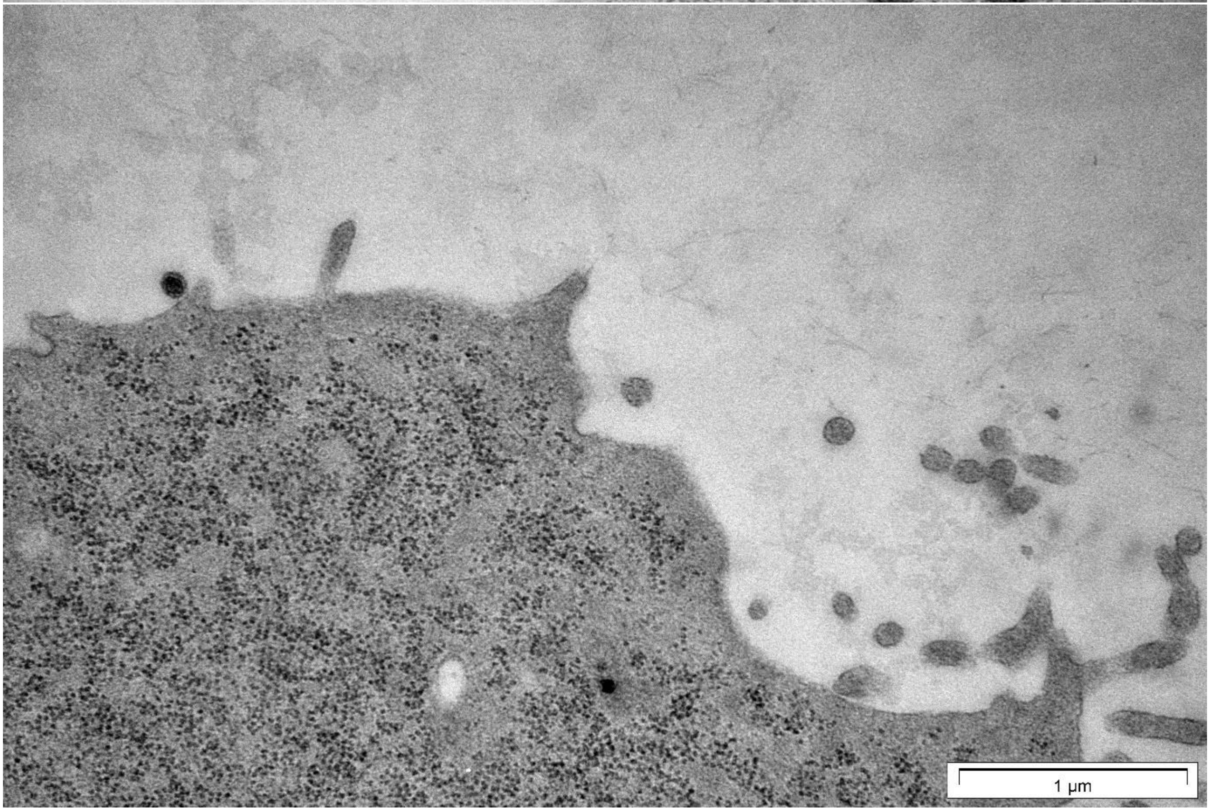
1 µm

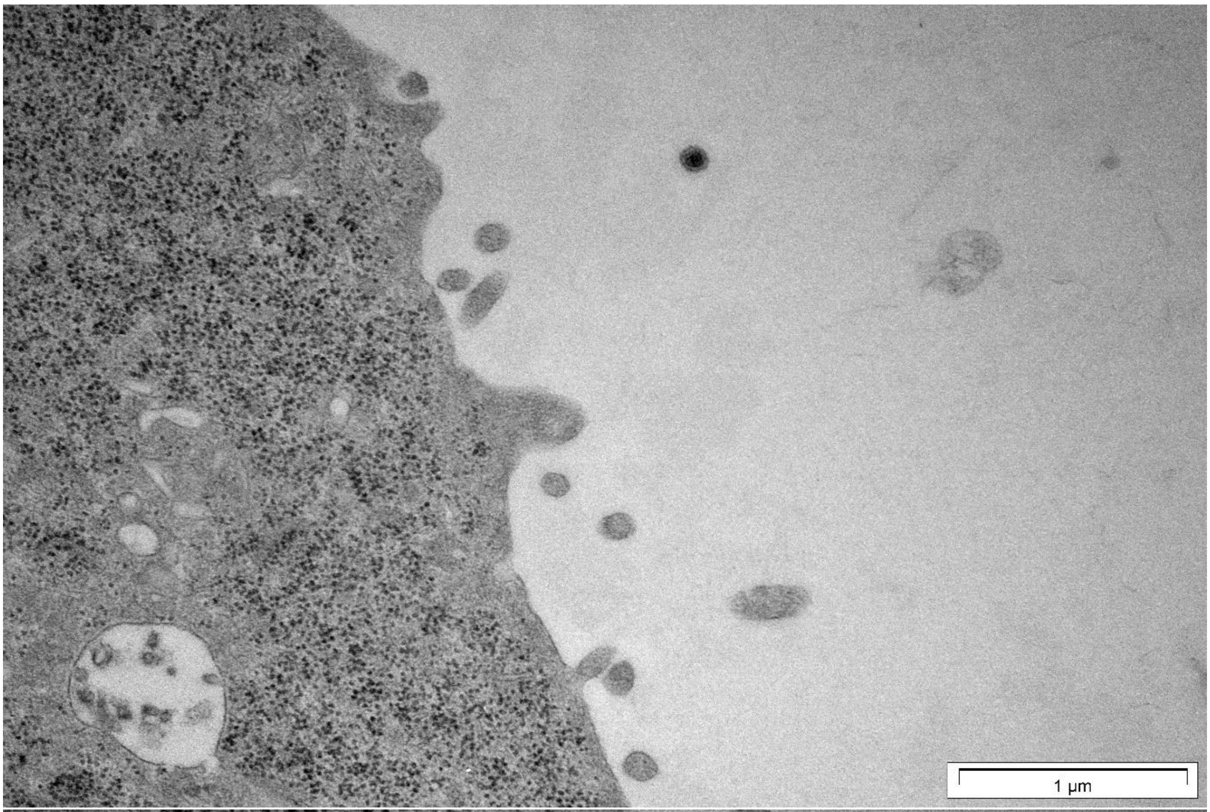
1 µm

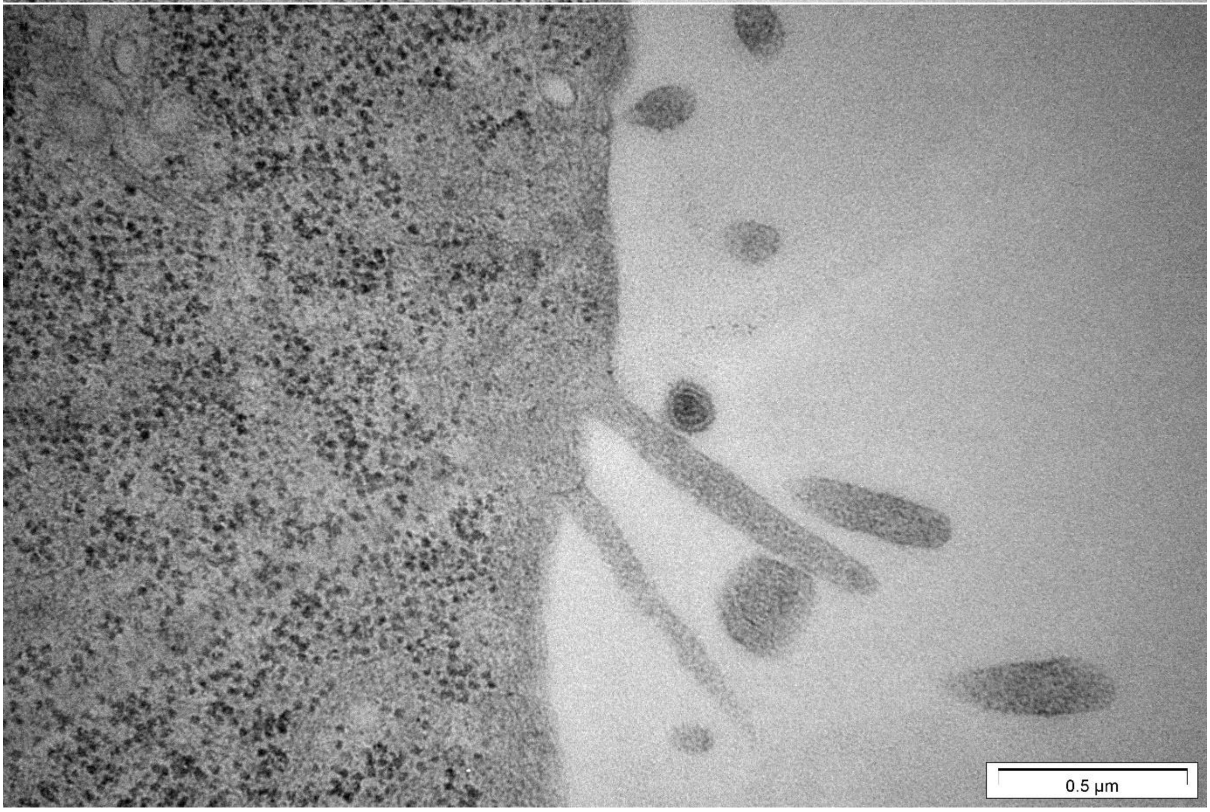
0.5 µm

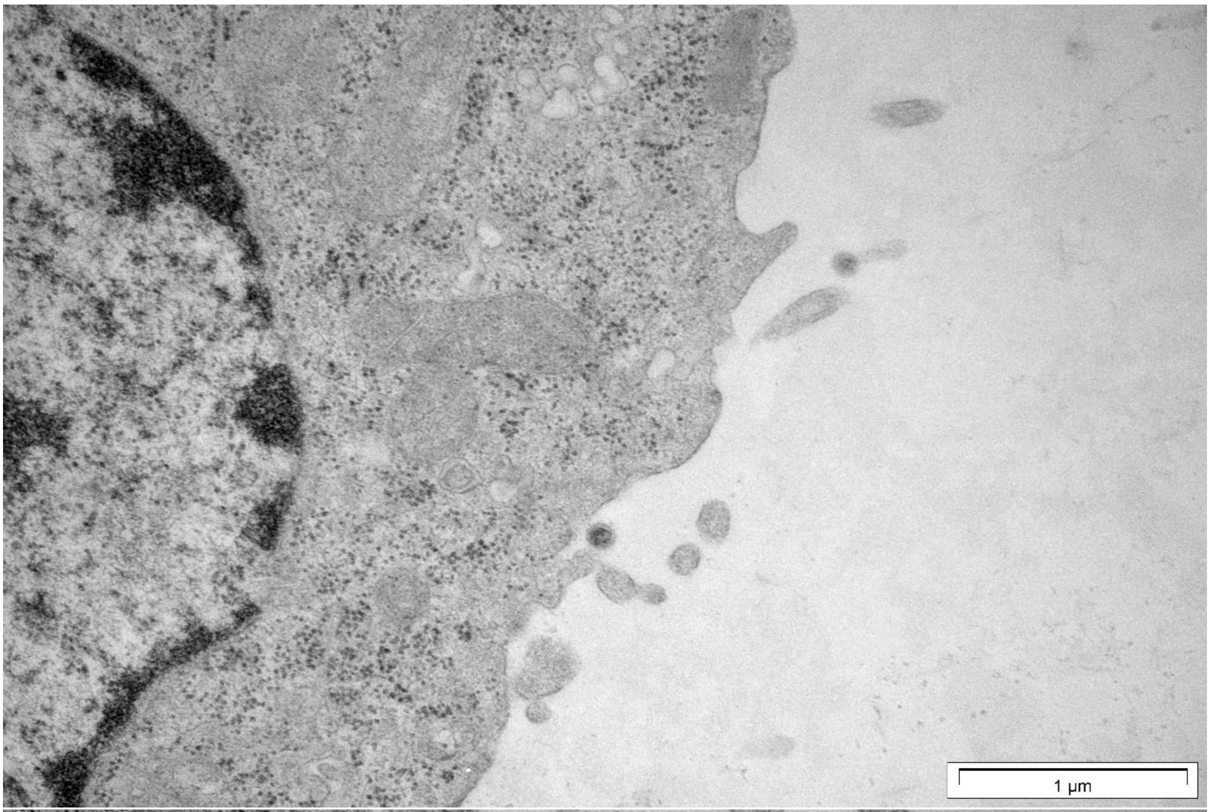
1 µm

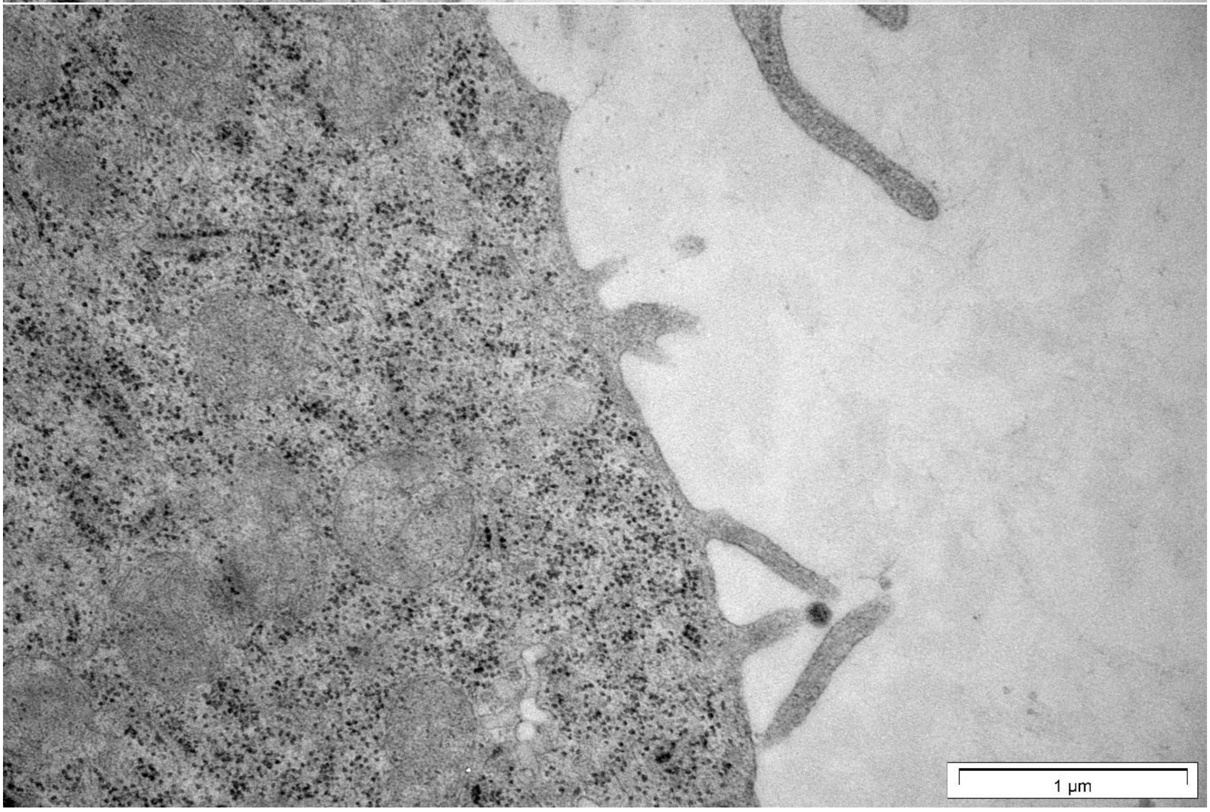
1 µm

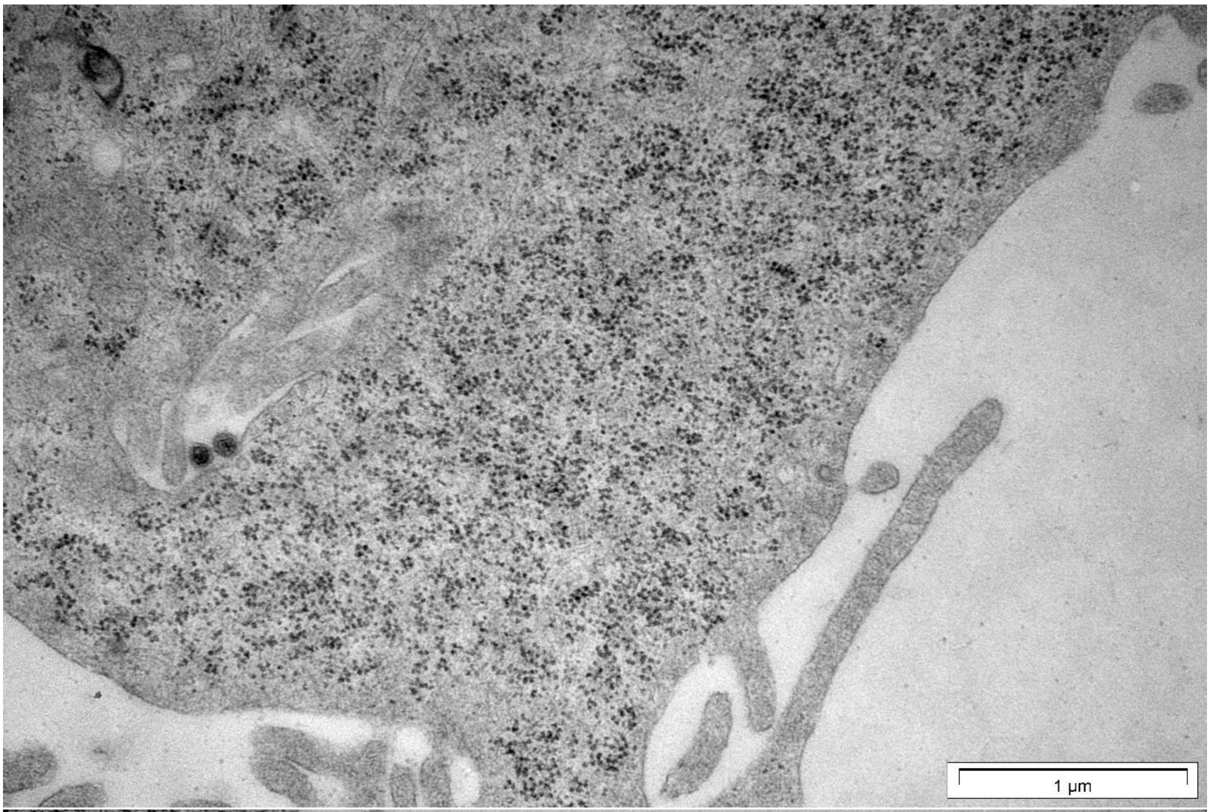
1 µm

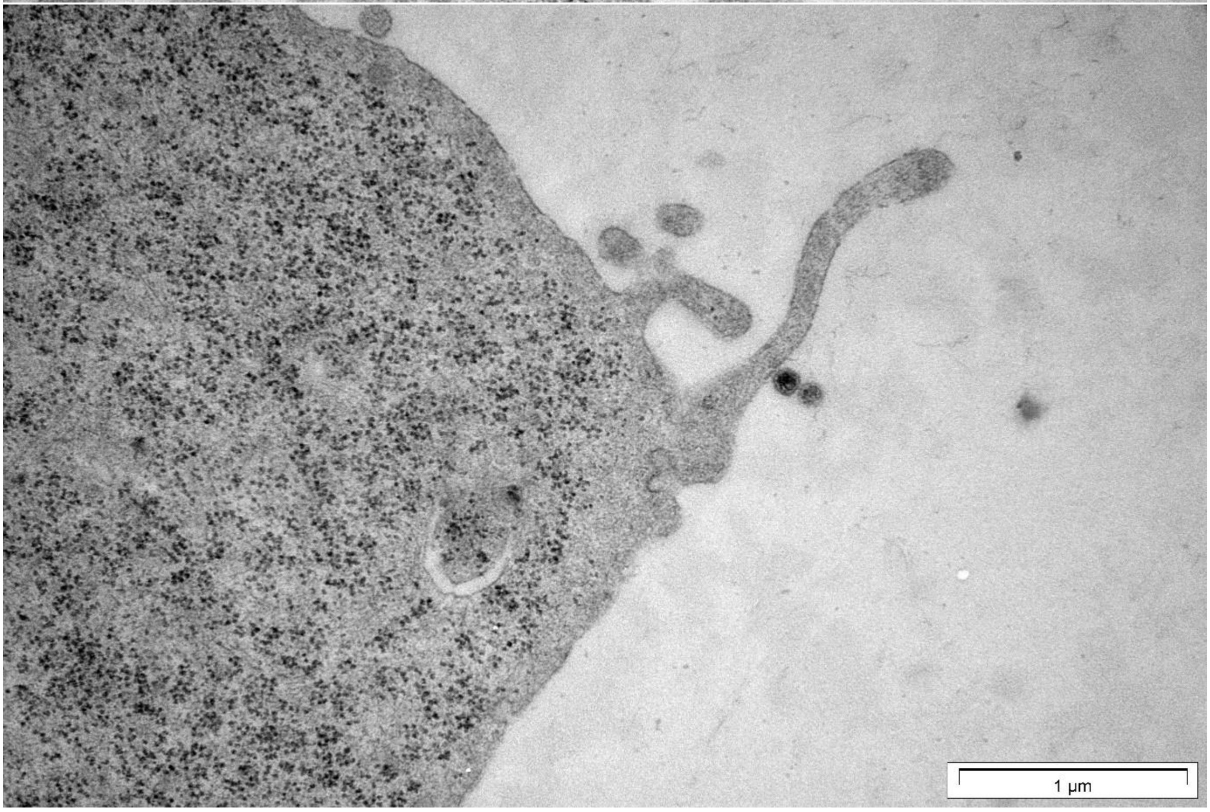
1 µm

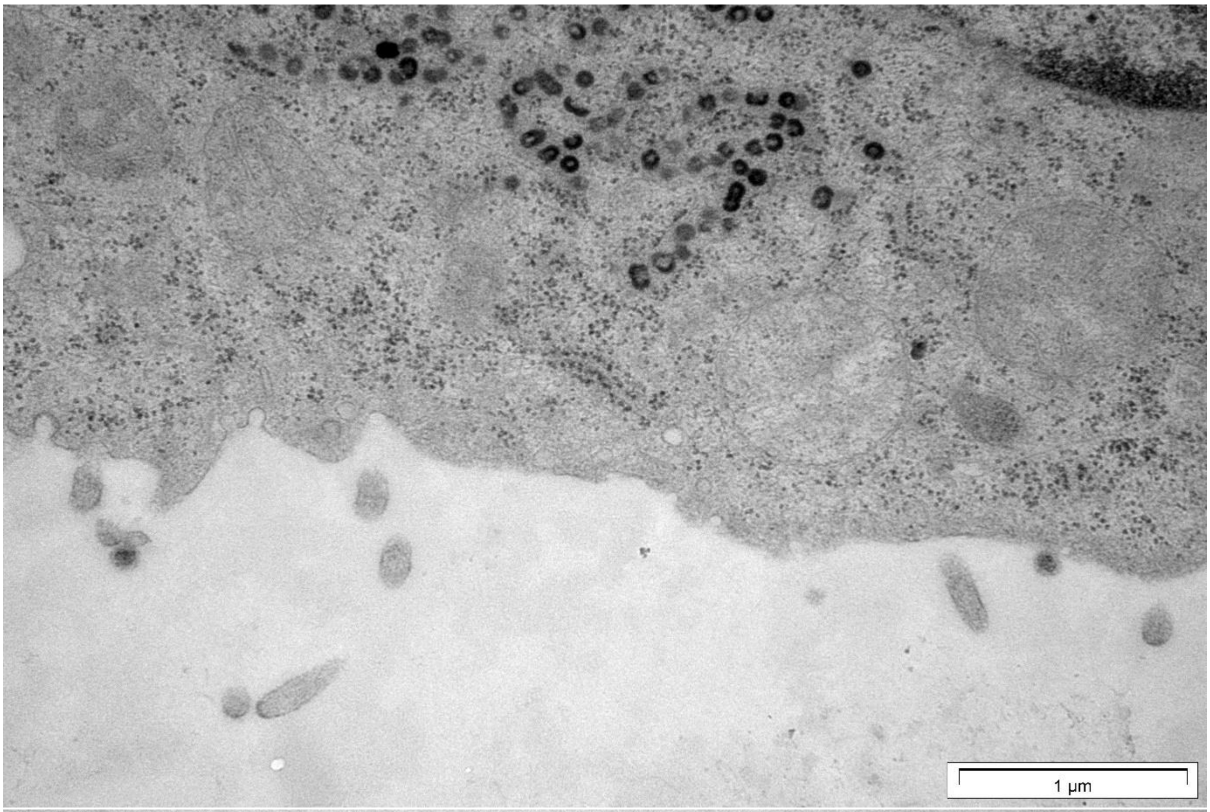
1 µm
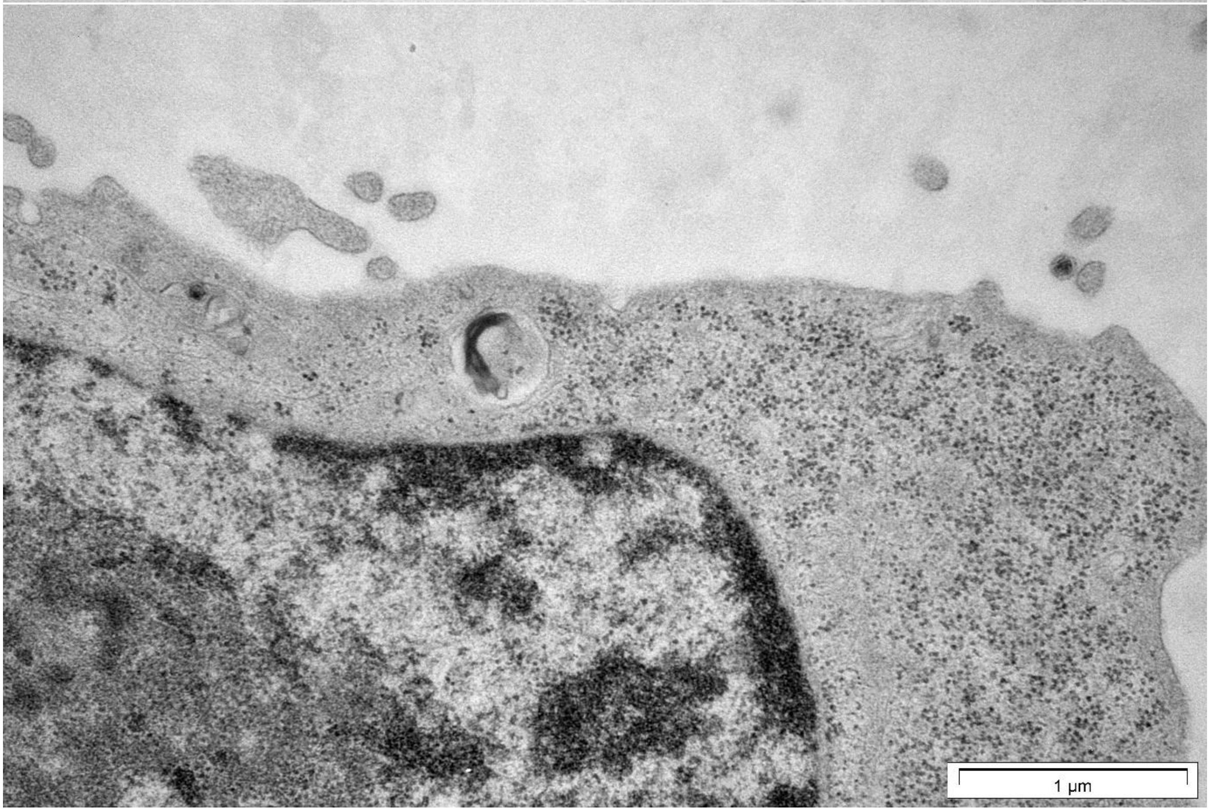
1 µm

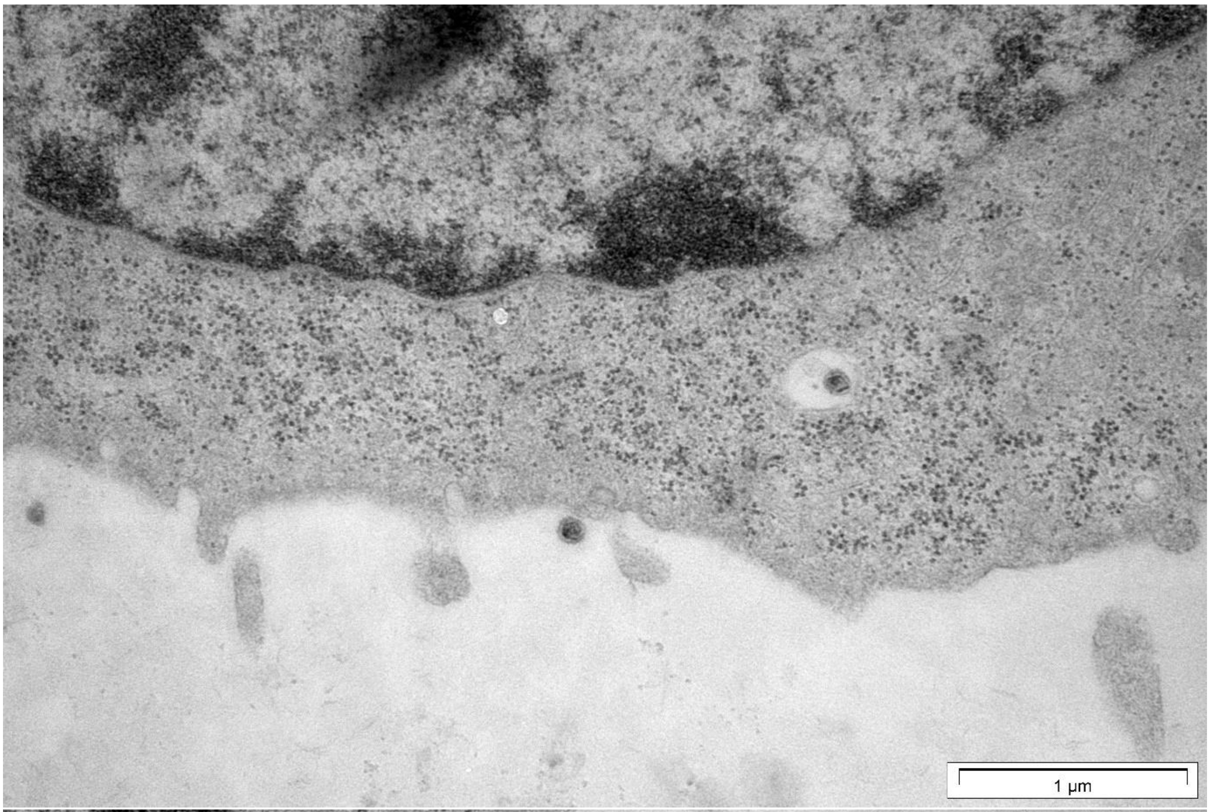
1 µm

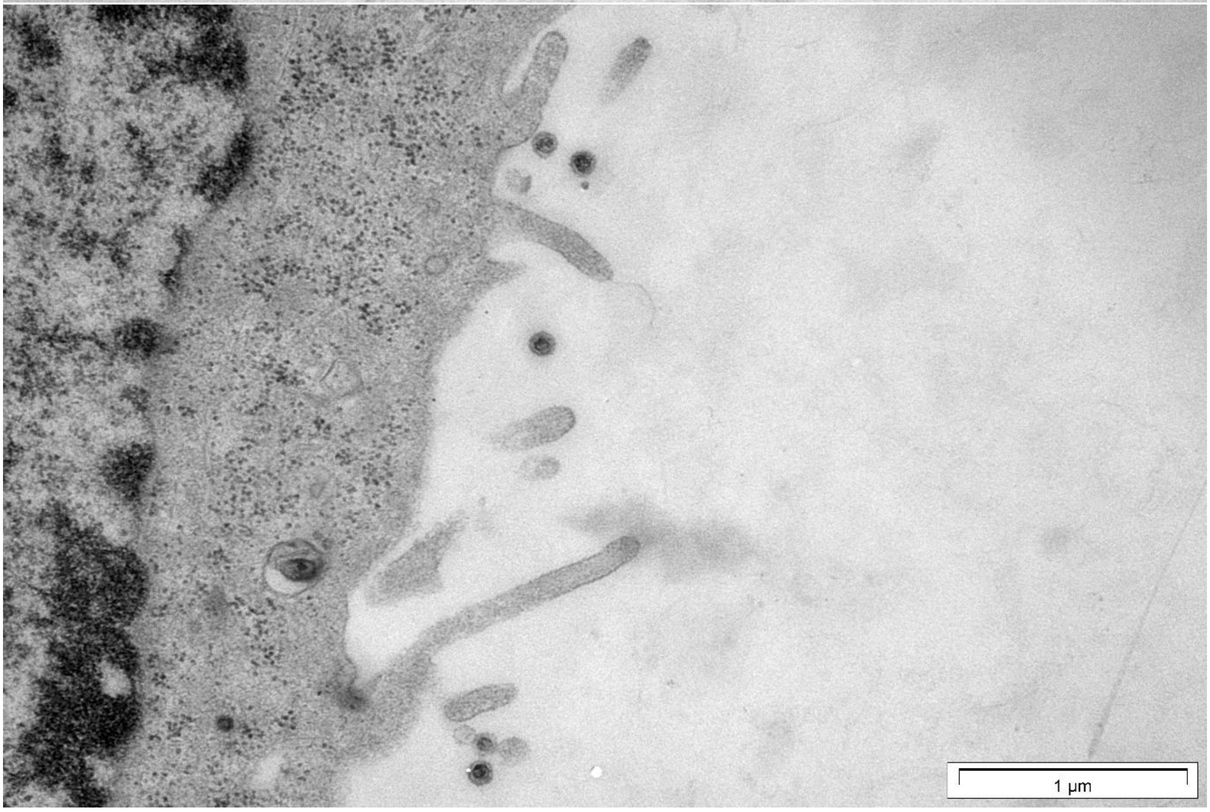
1 µm

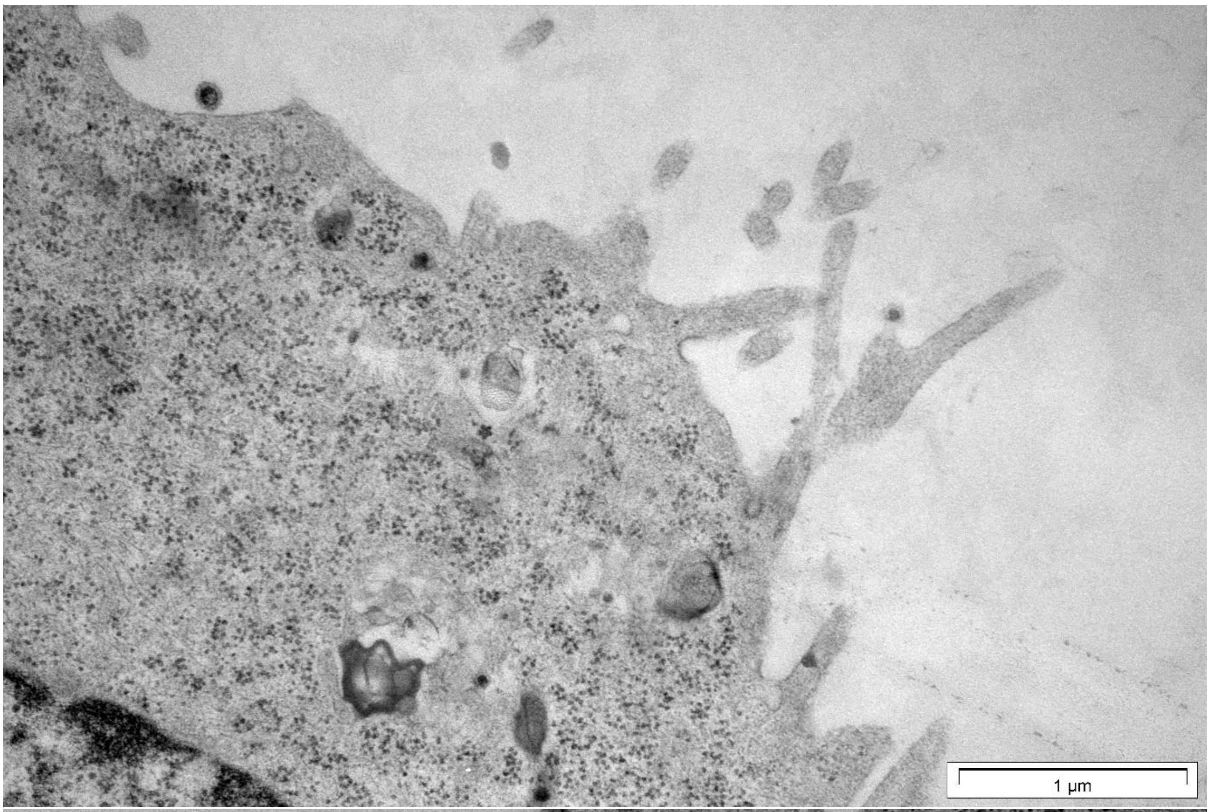
1 µm

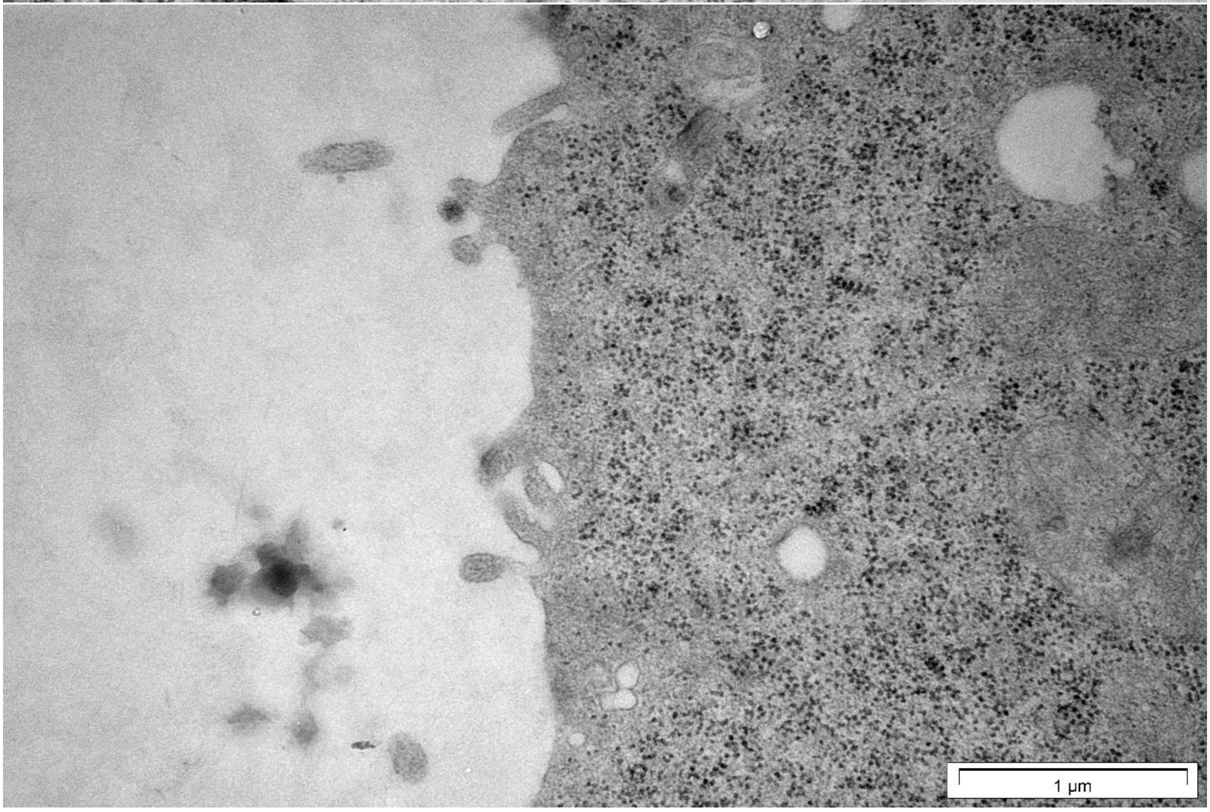
1 µm

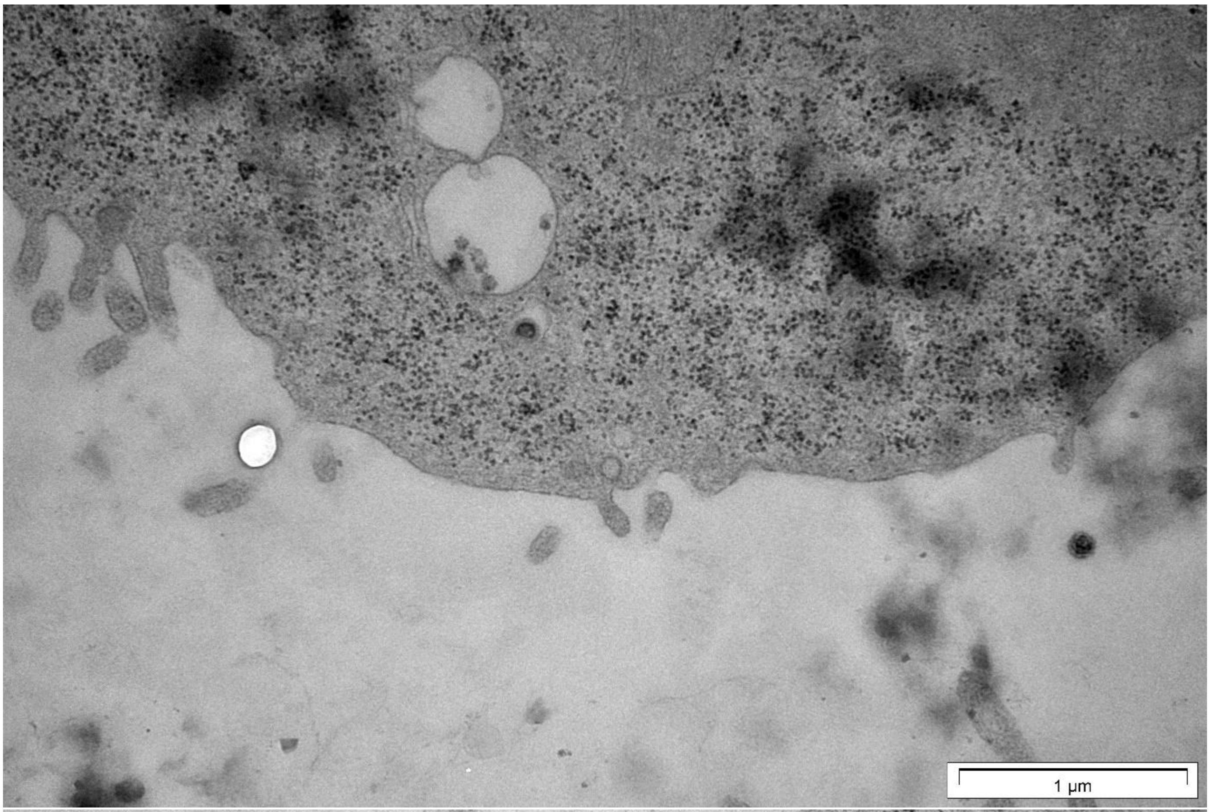
1 µm

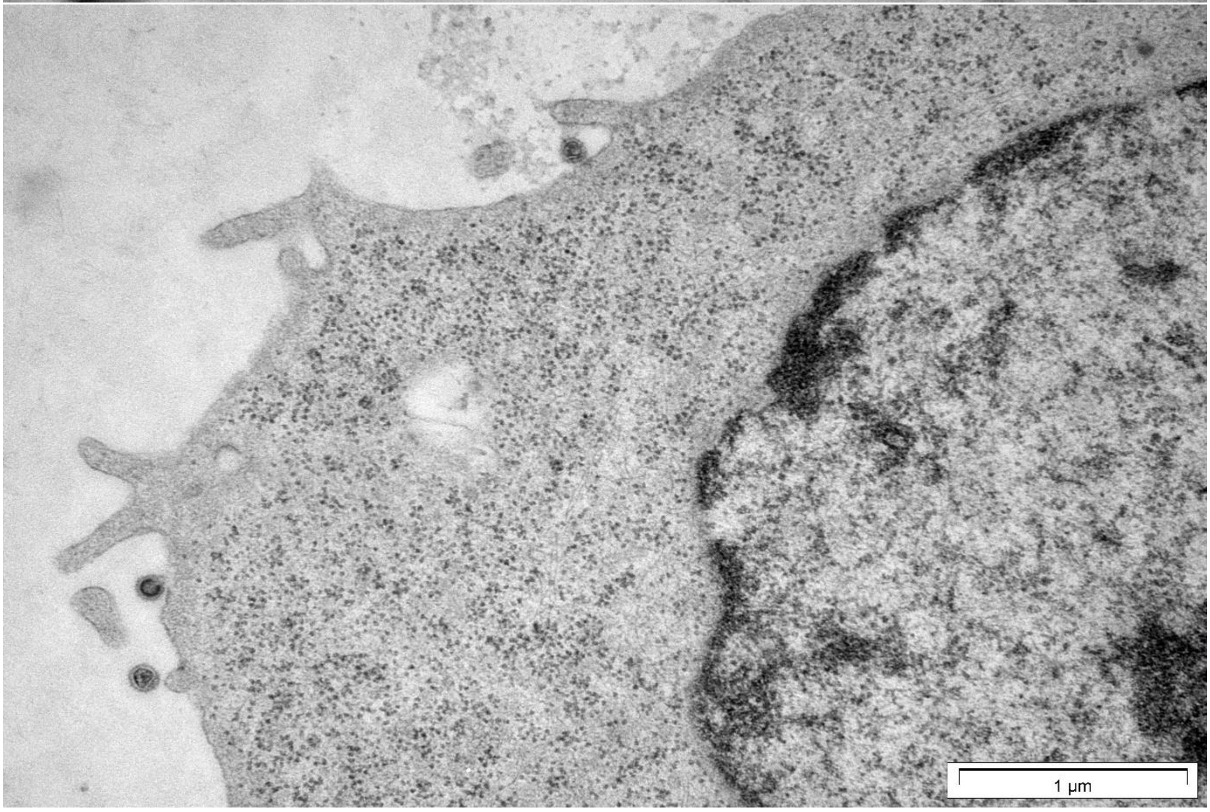
1 µm

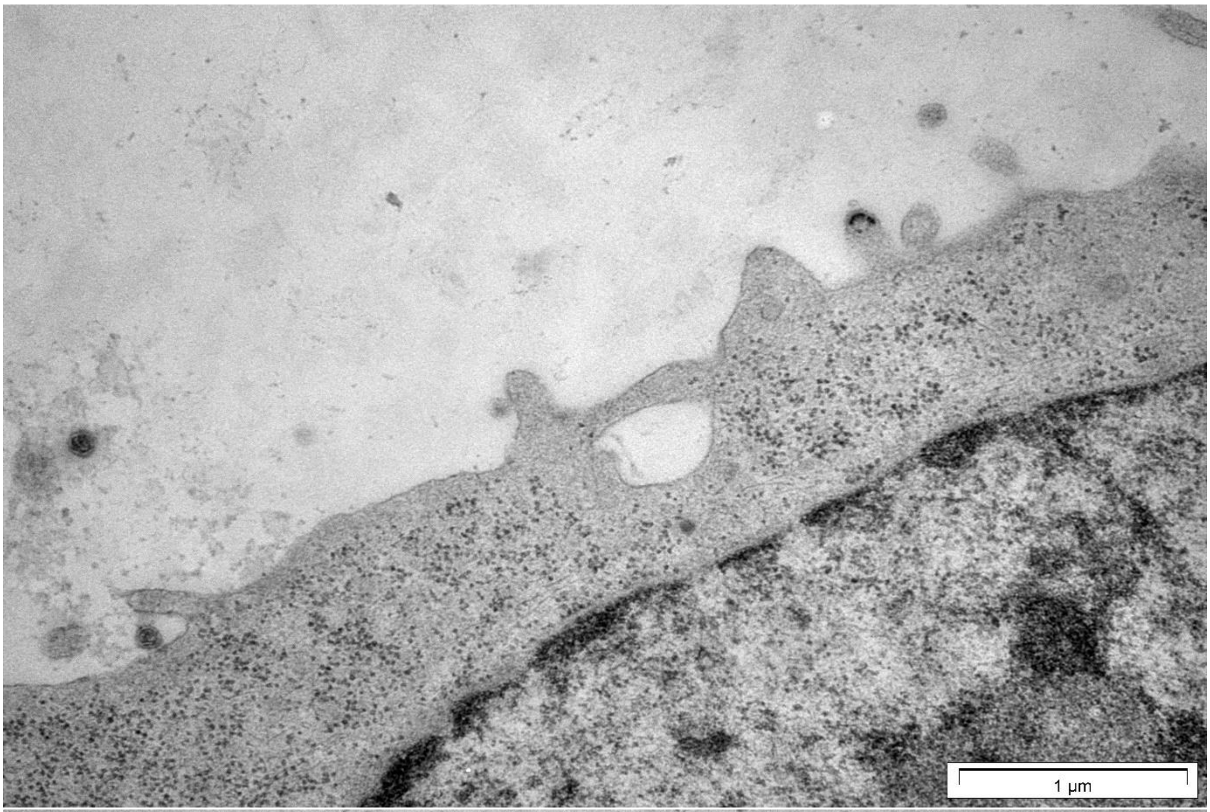
1 µm

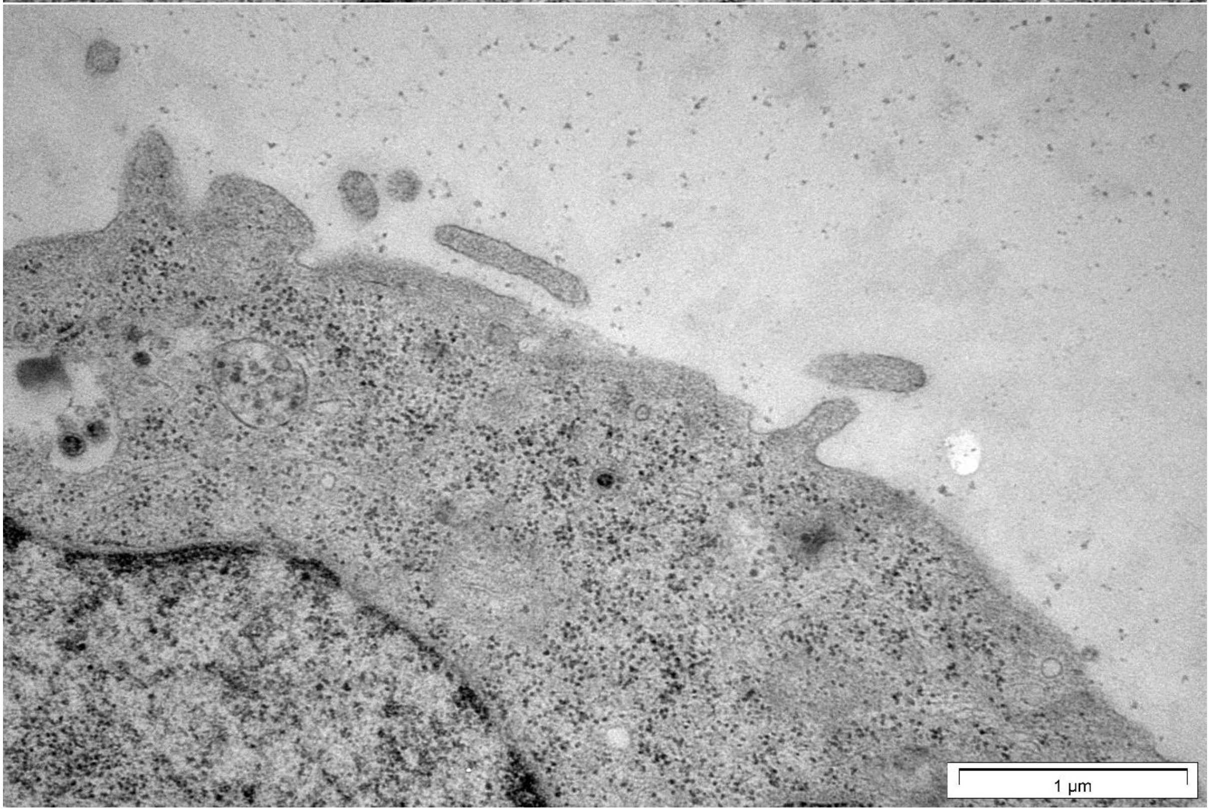
1 µm

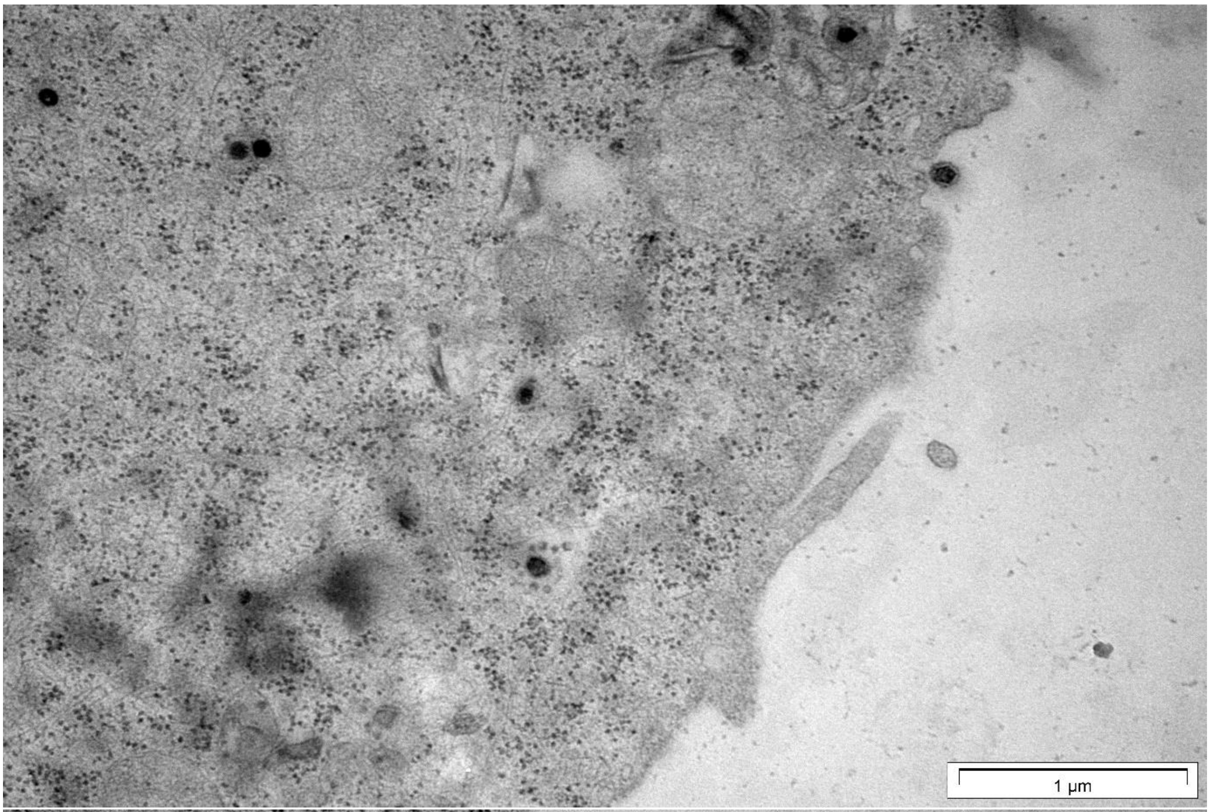
1 µm

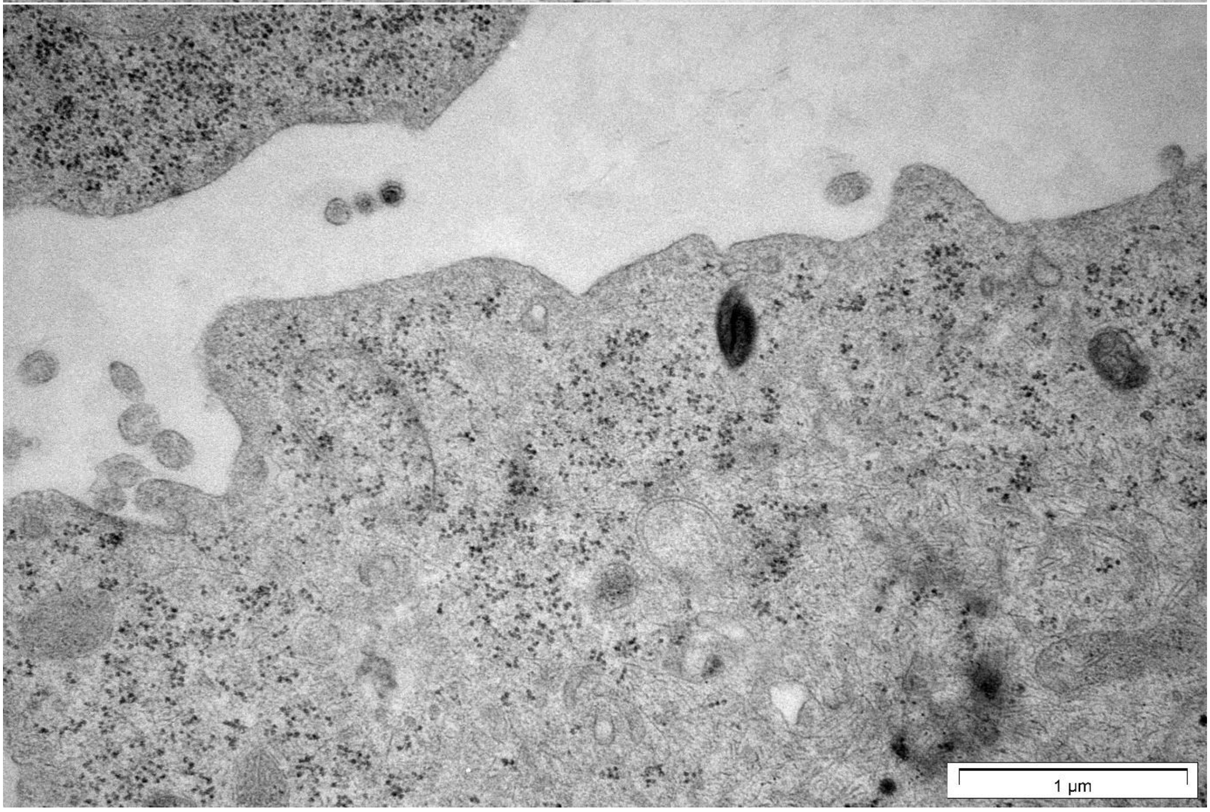
1 µm

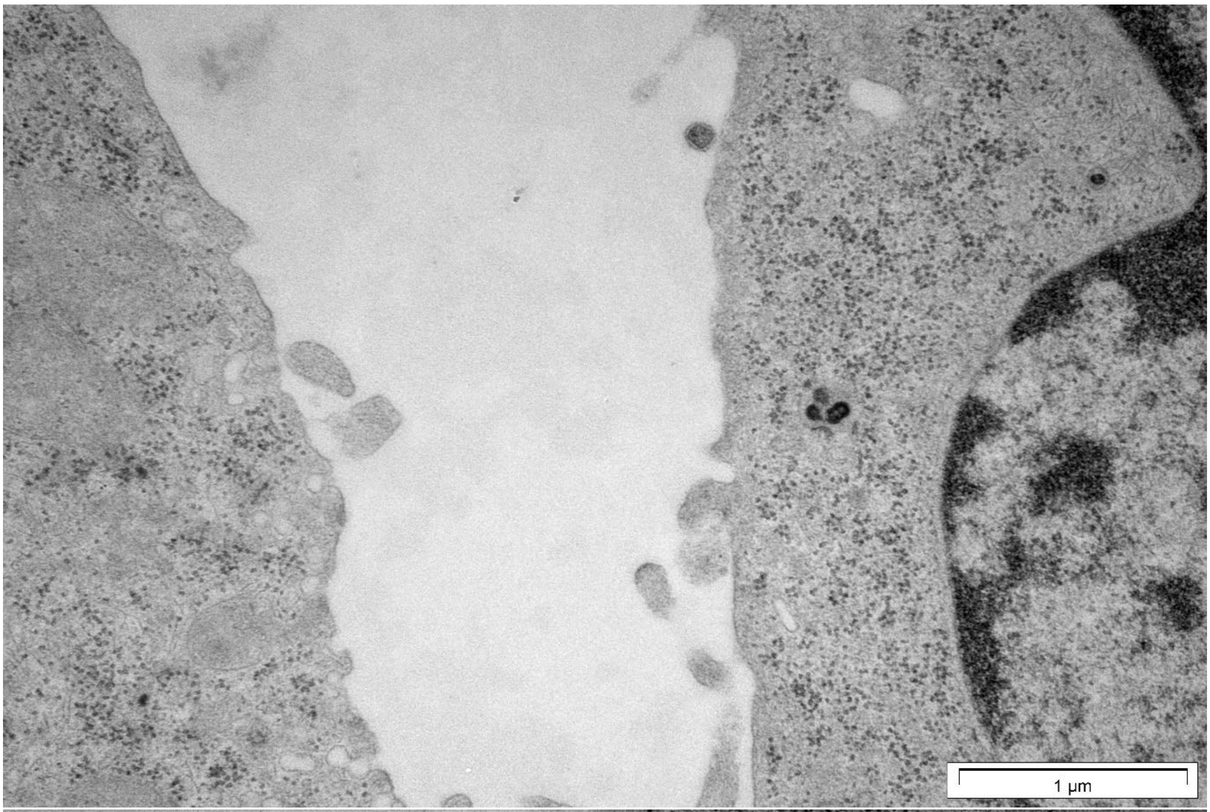
1 µm

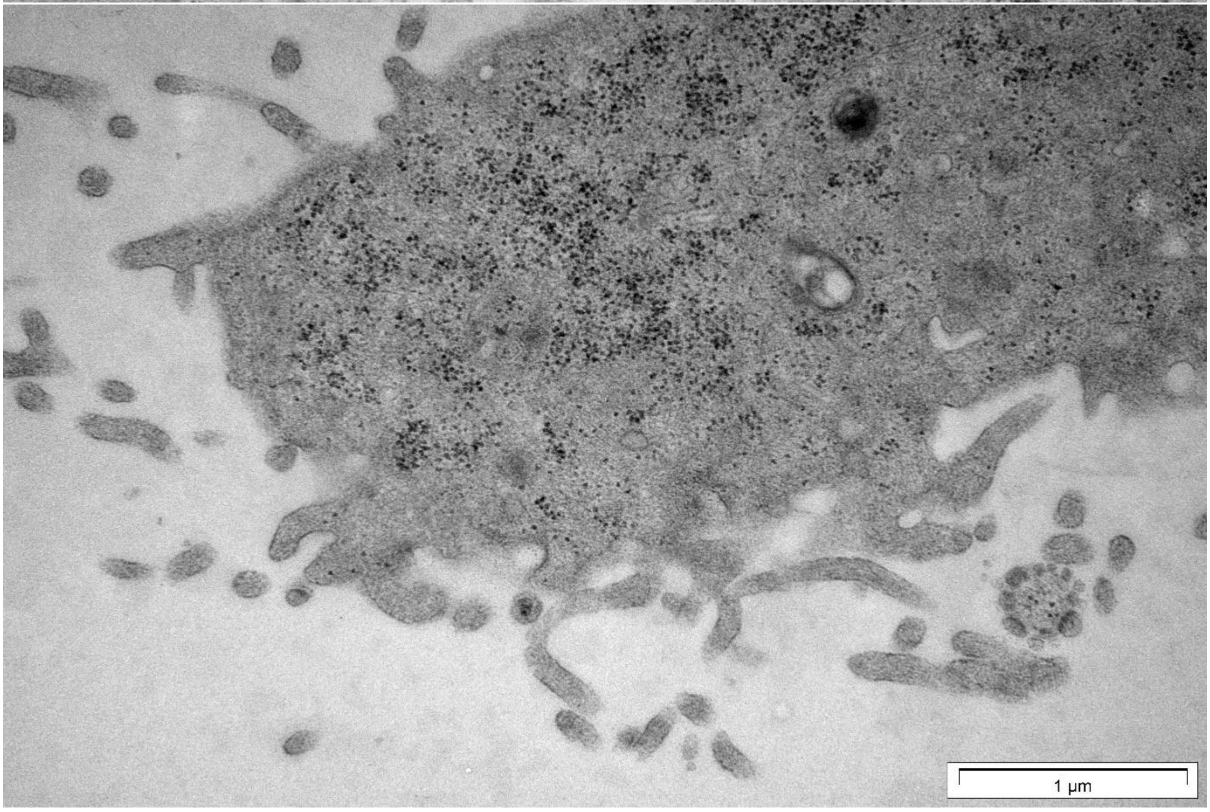
1 µm

Dextran sulfate, 5 min.

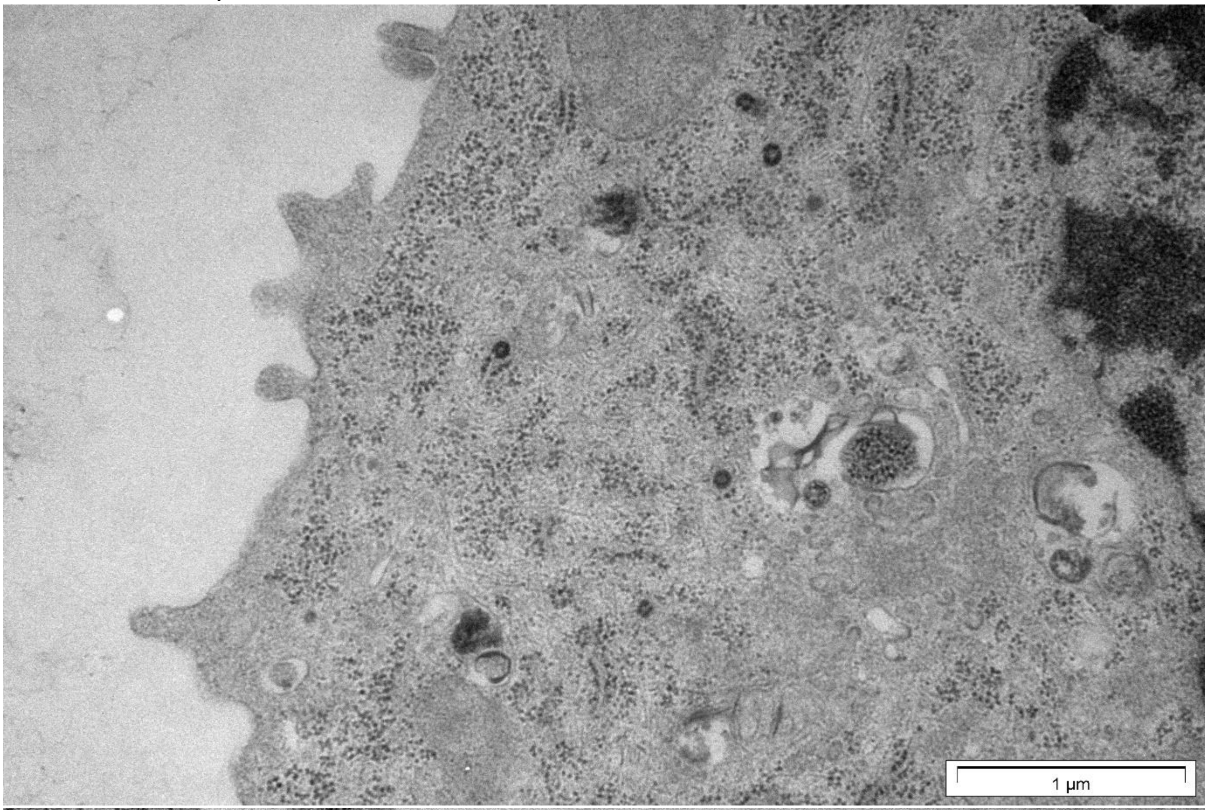

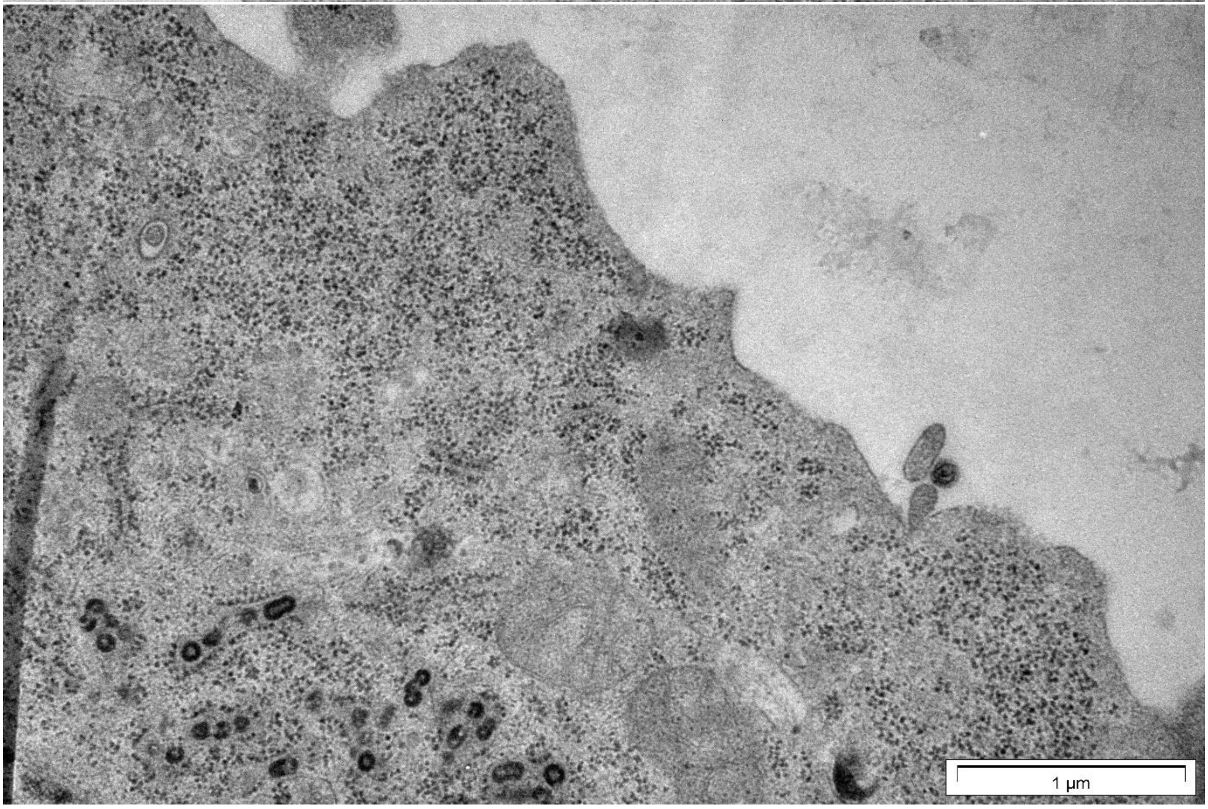

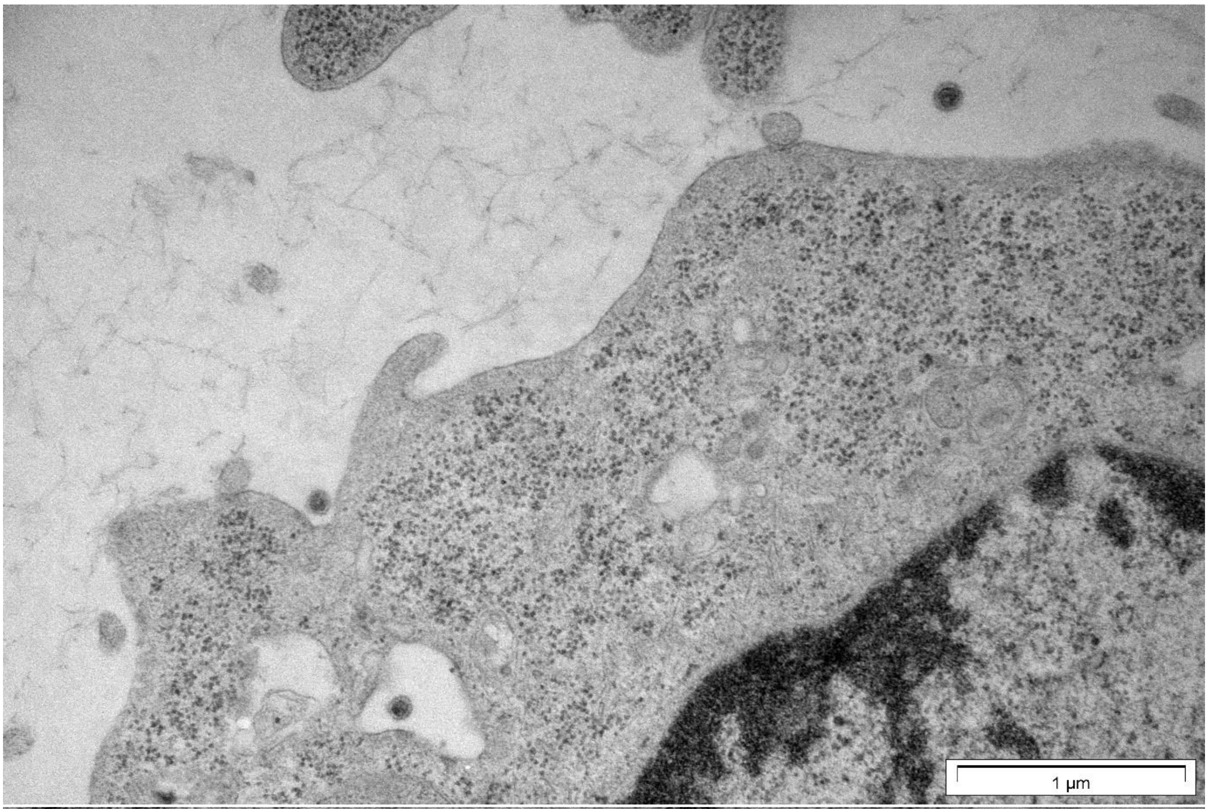
1 µm

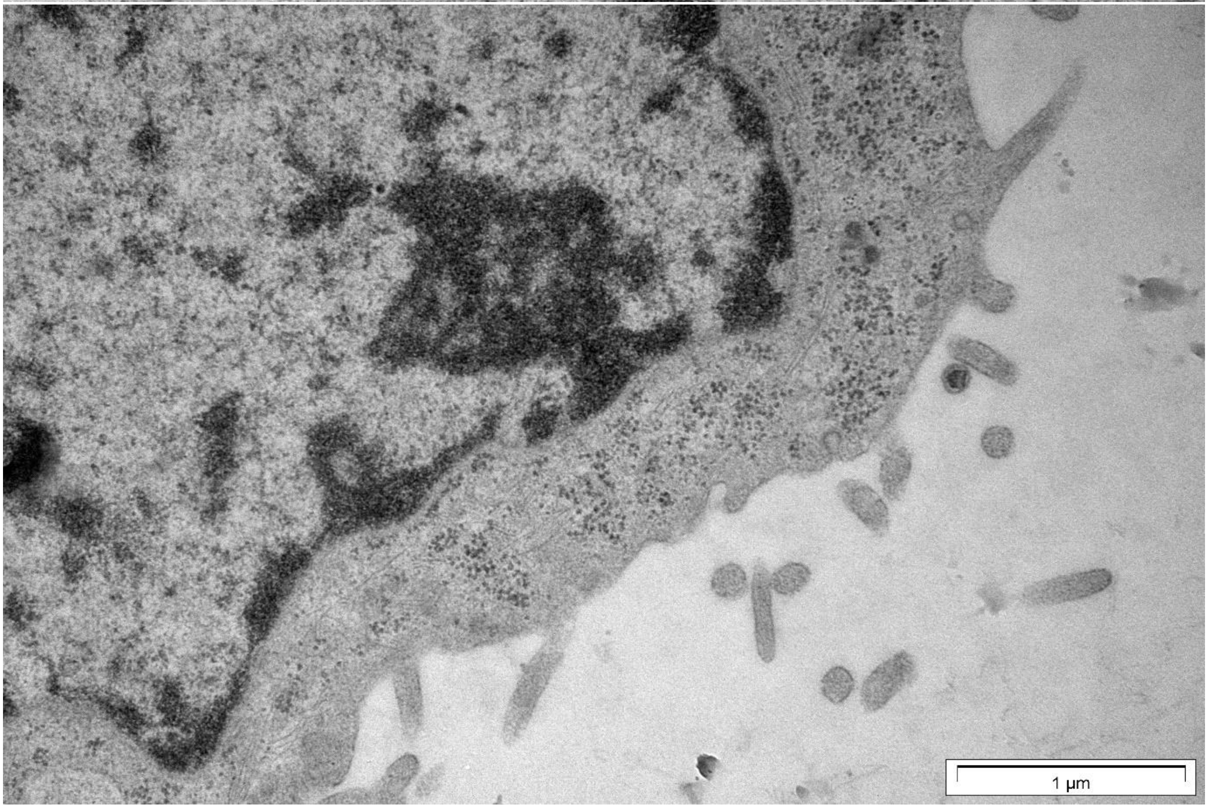
1 µm

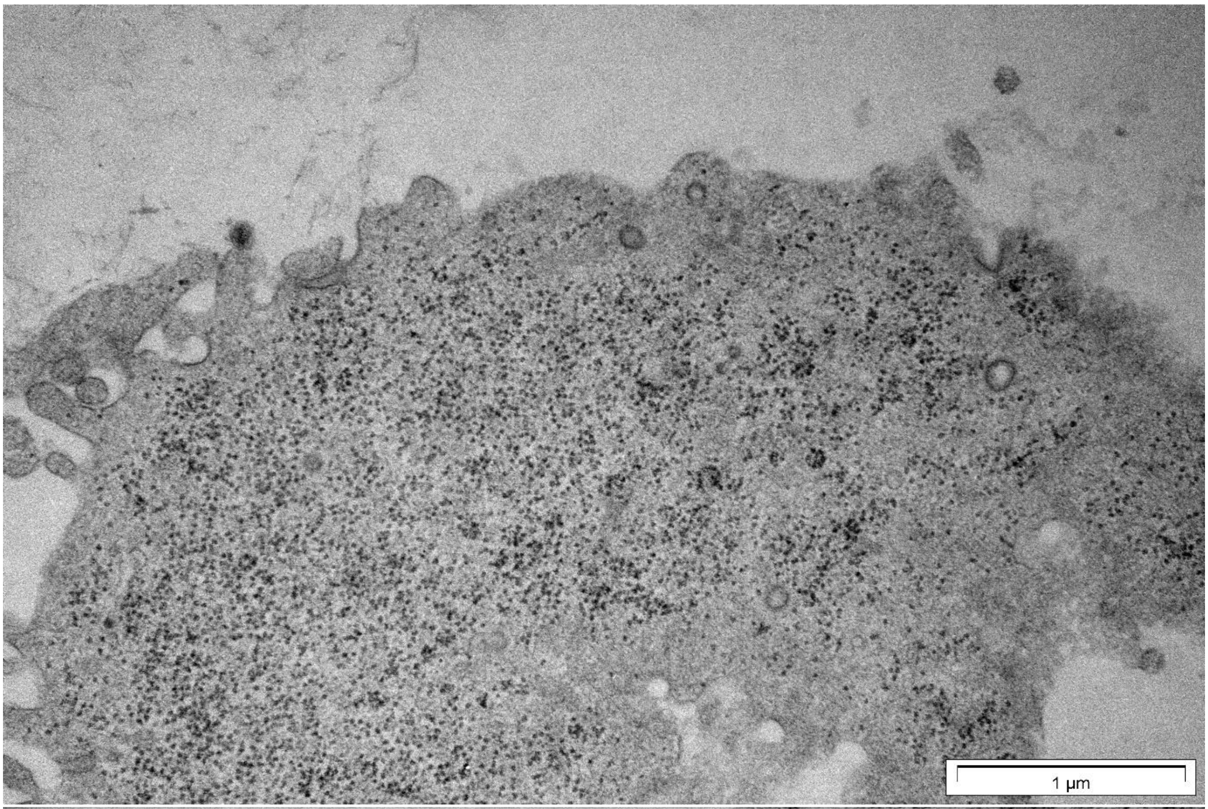
1 µm

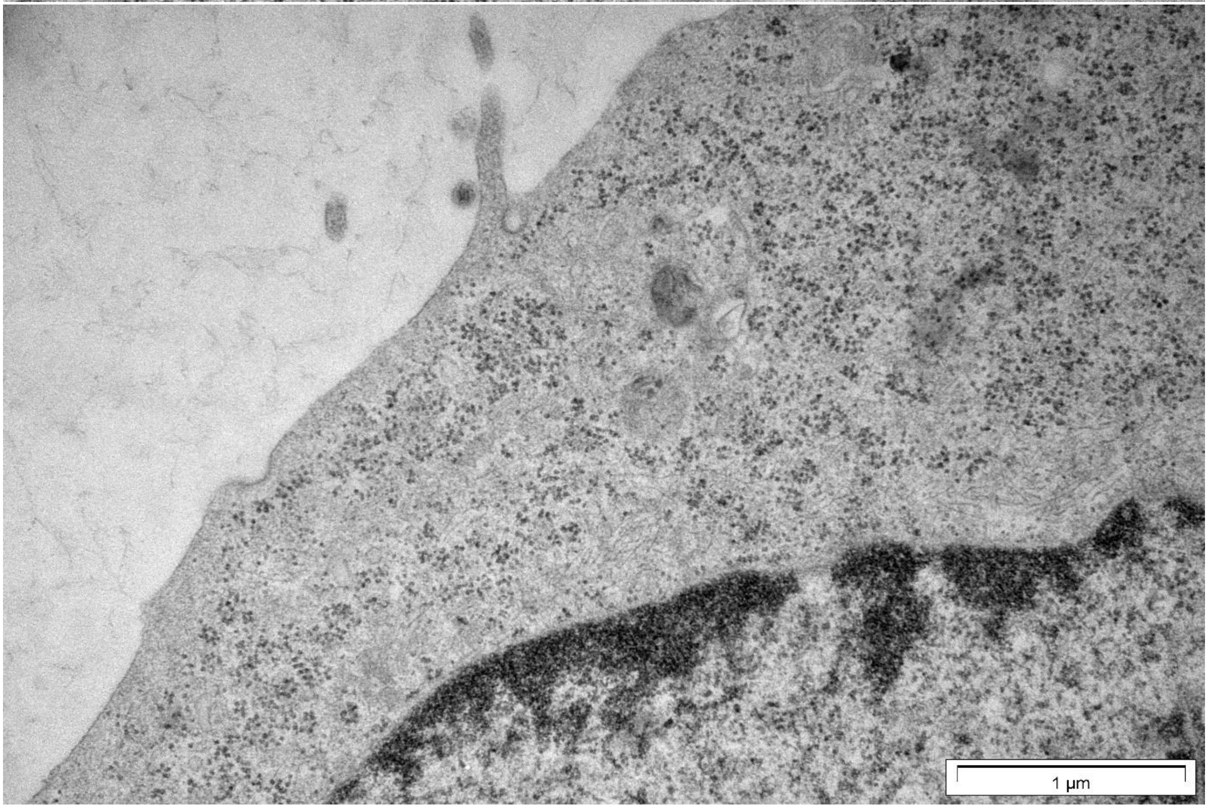
1 µm

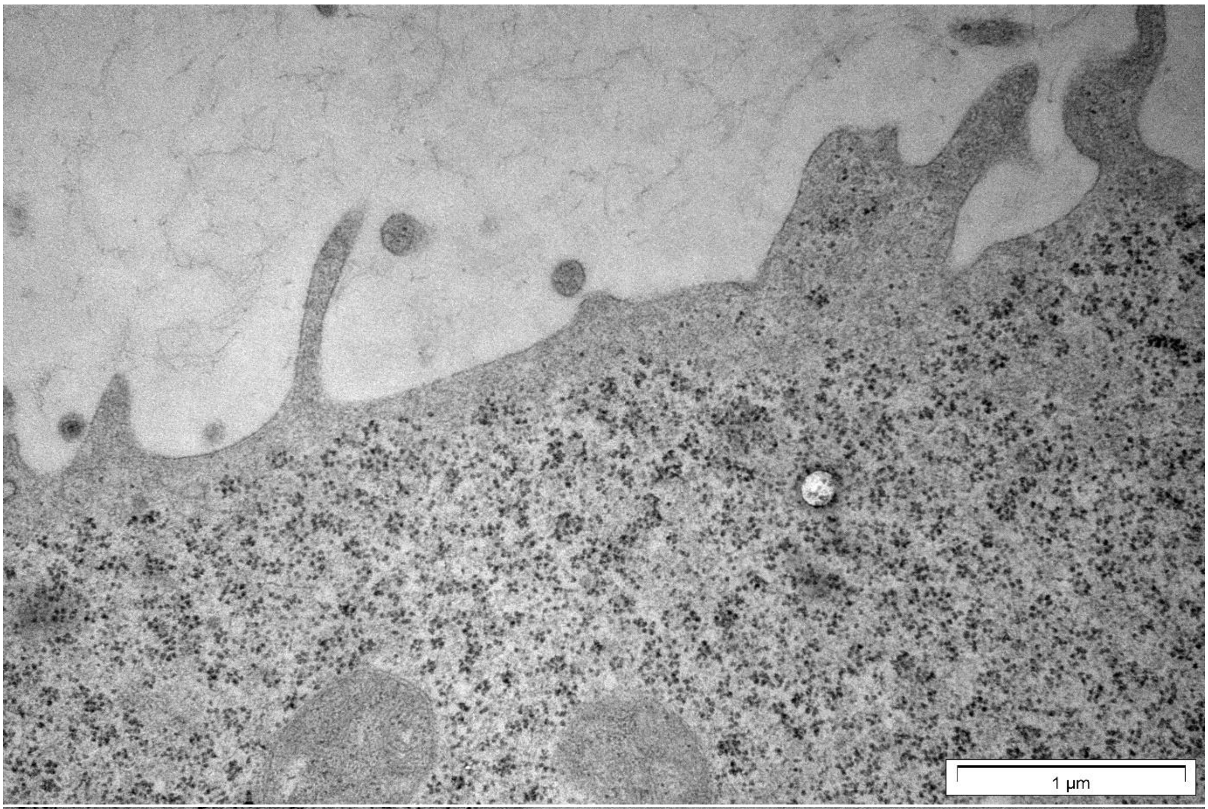
1 µm

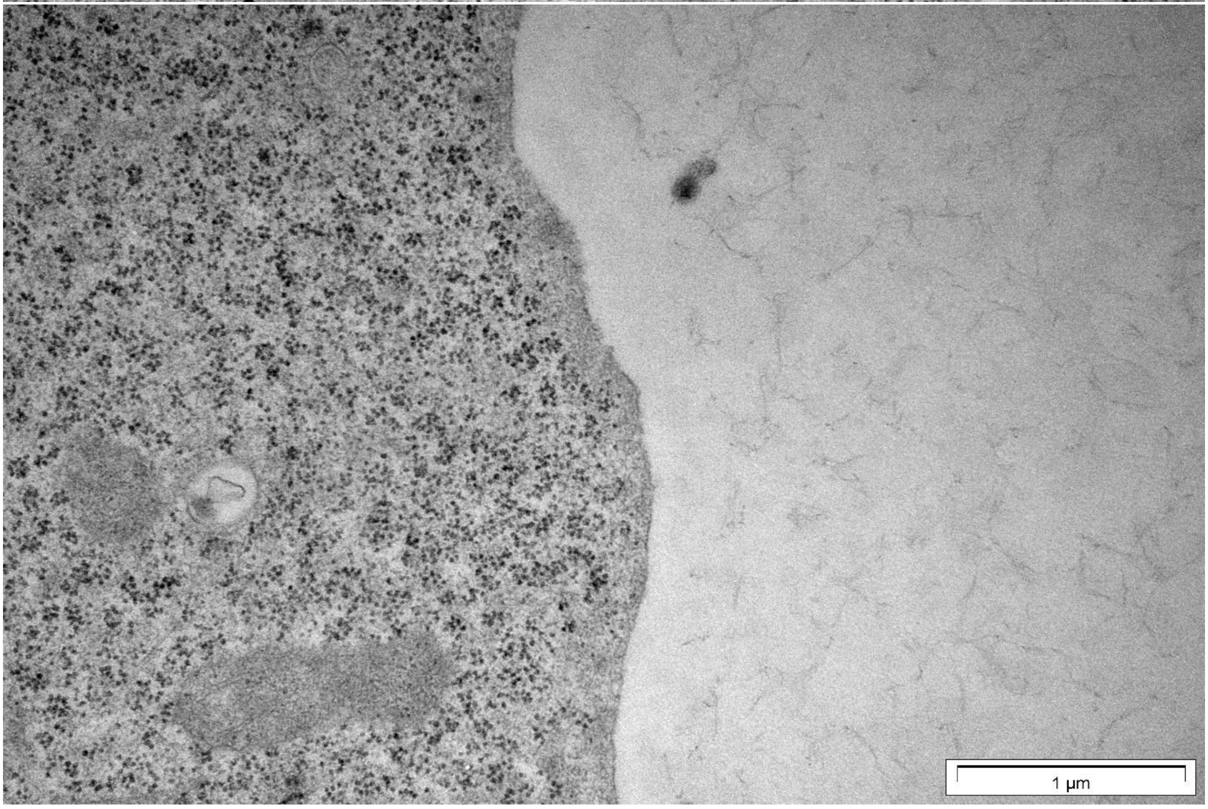
1 µm

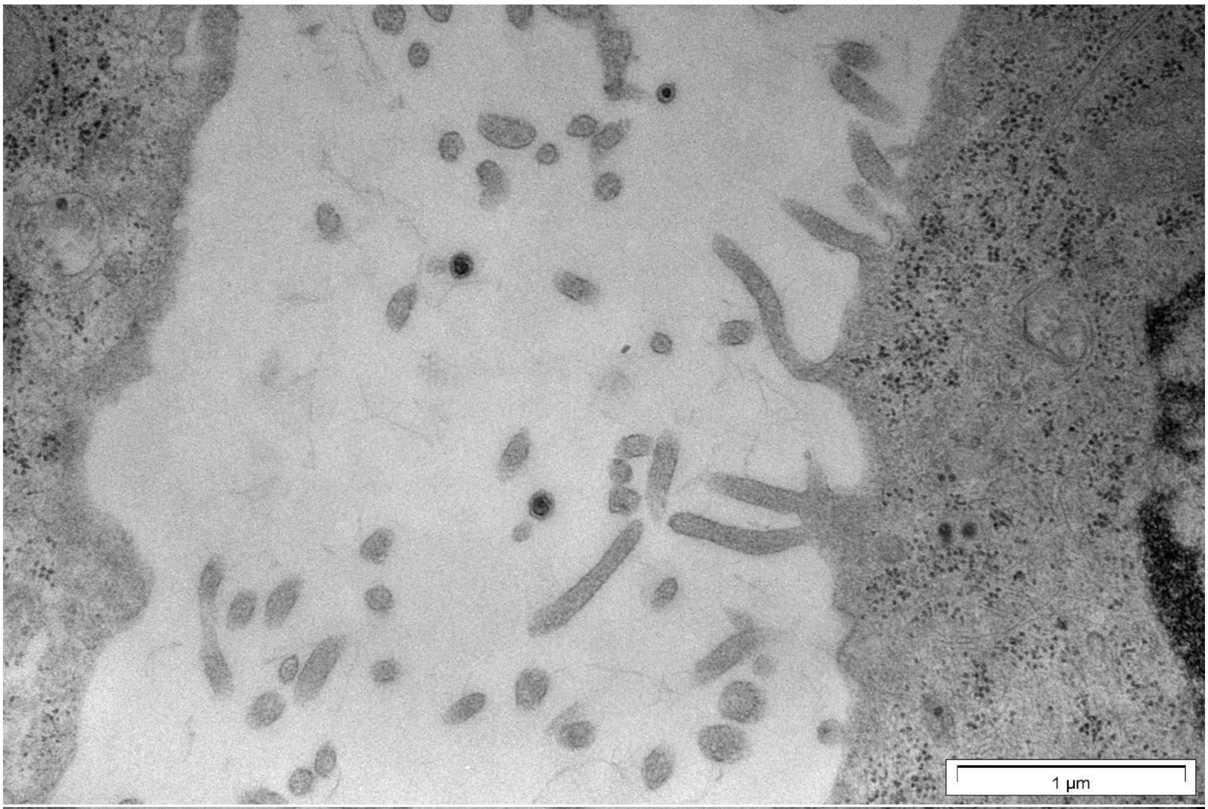
1 µm

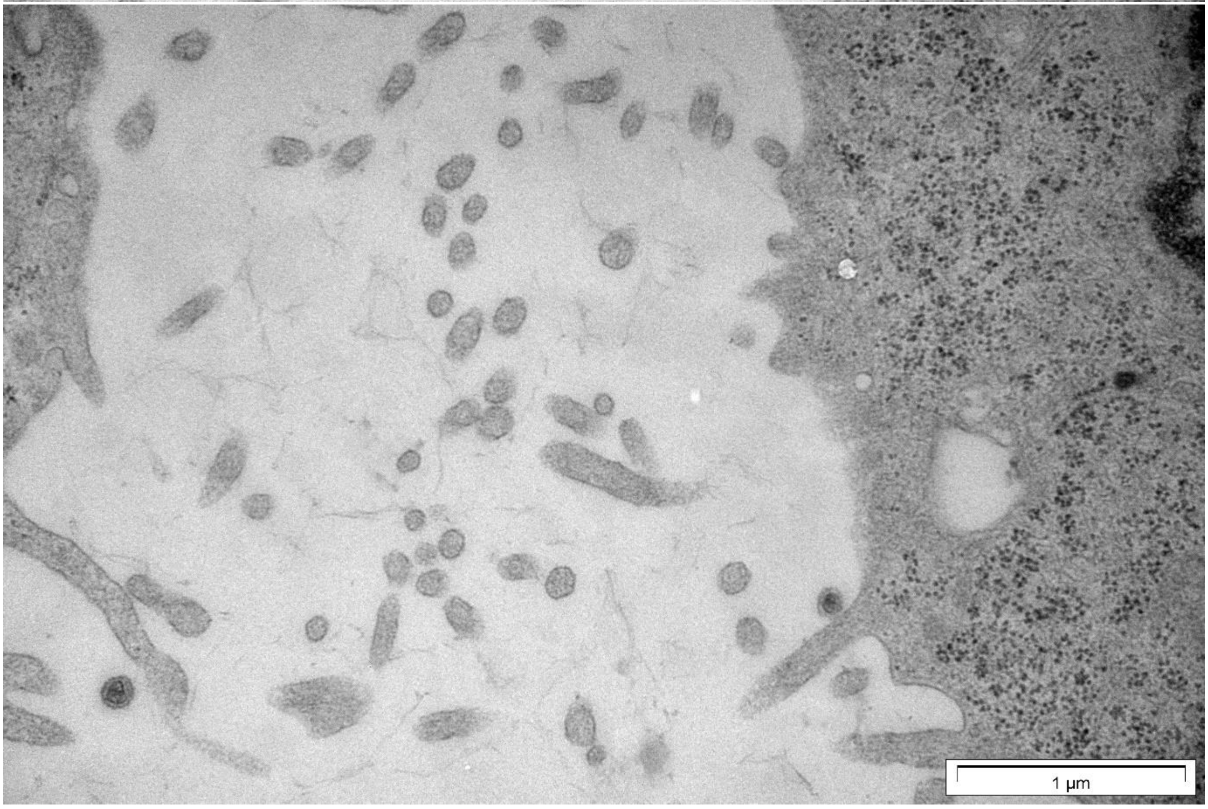
1 µm

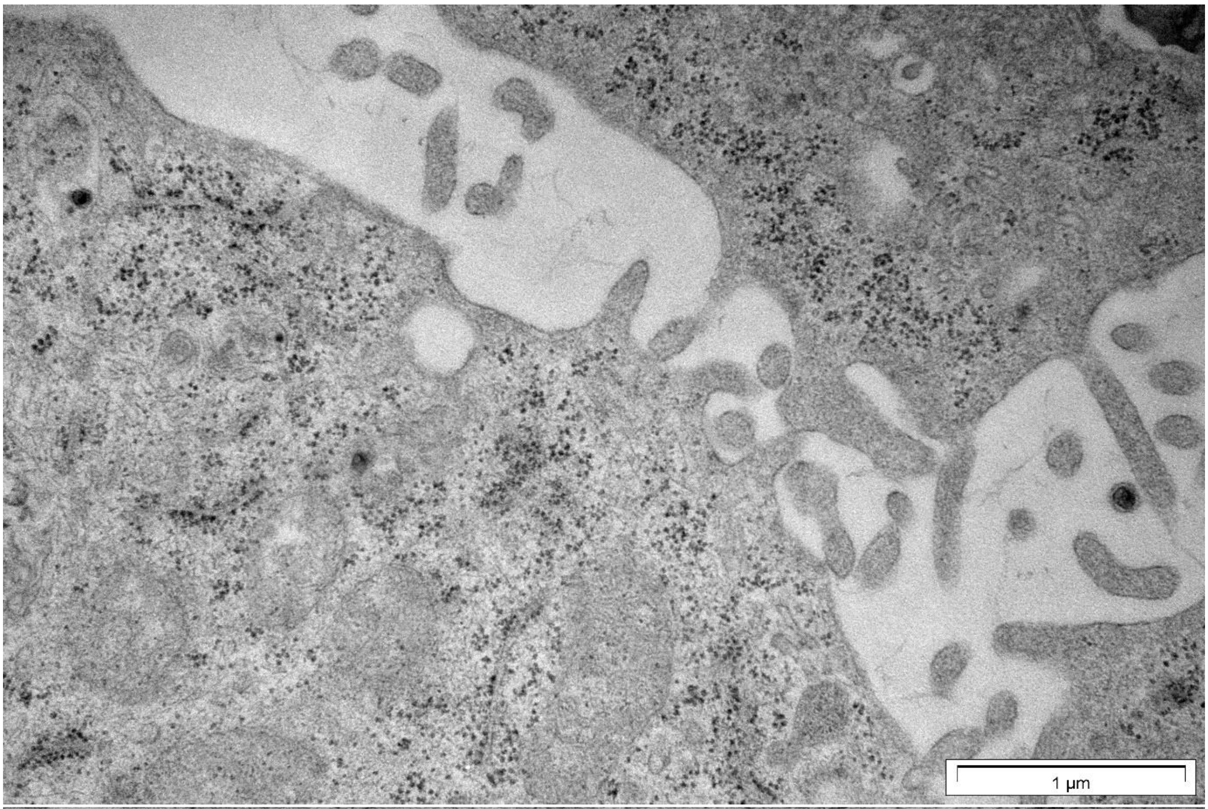
1 µm

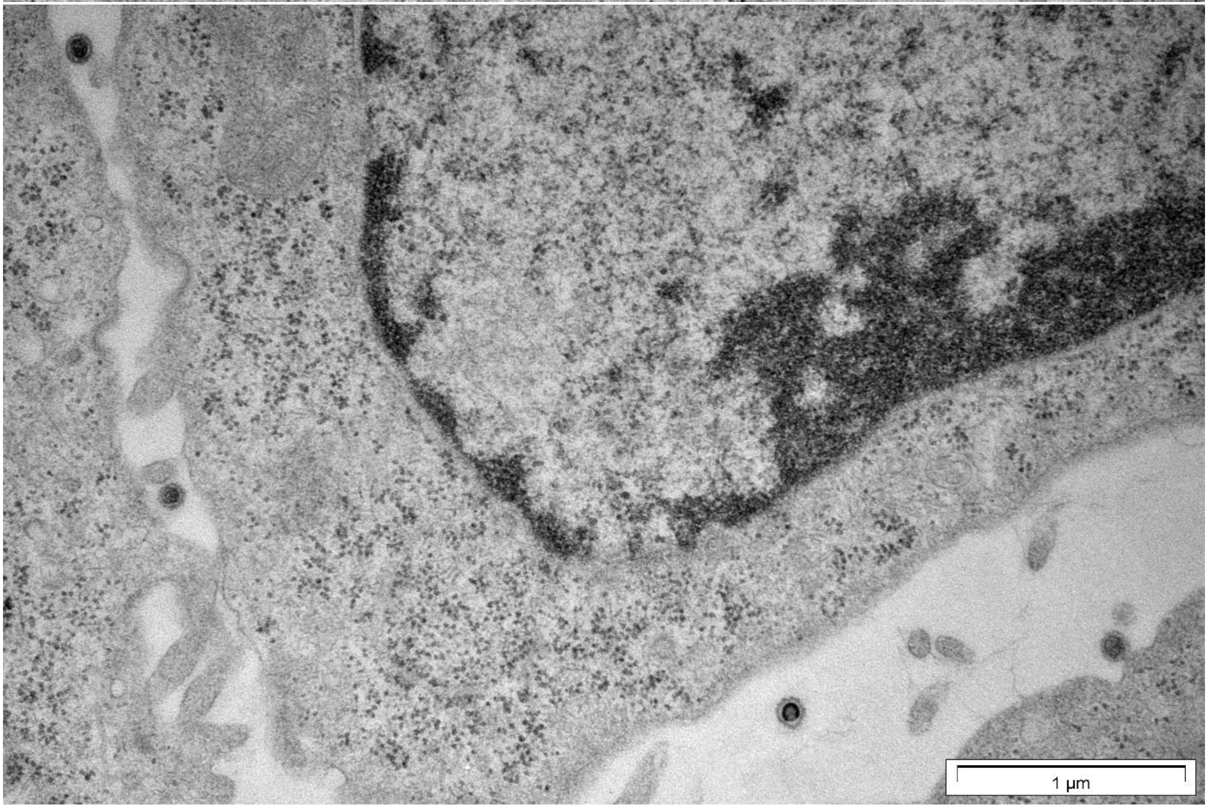
1 µm

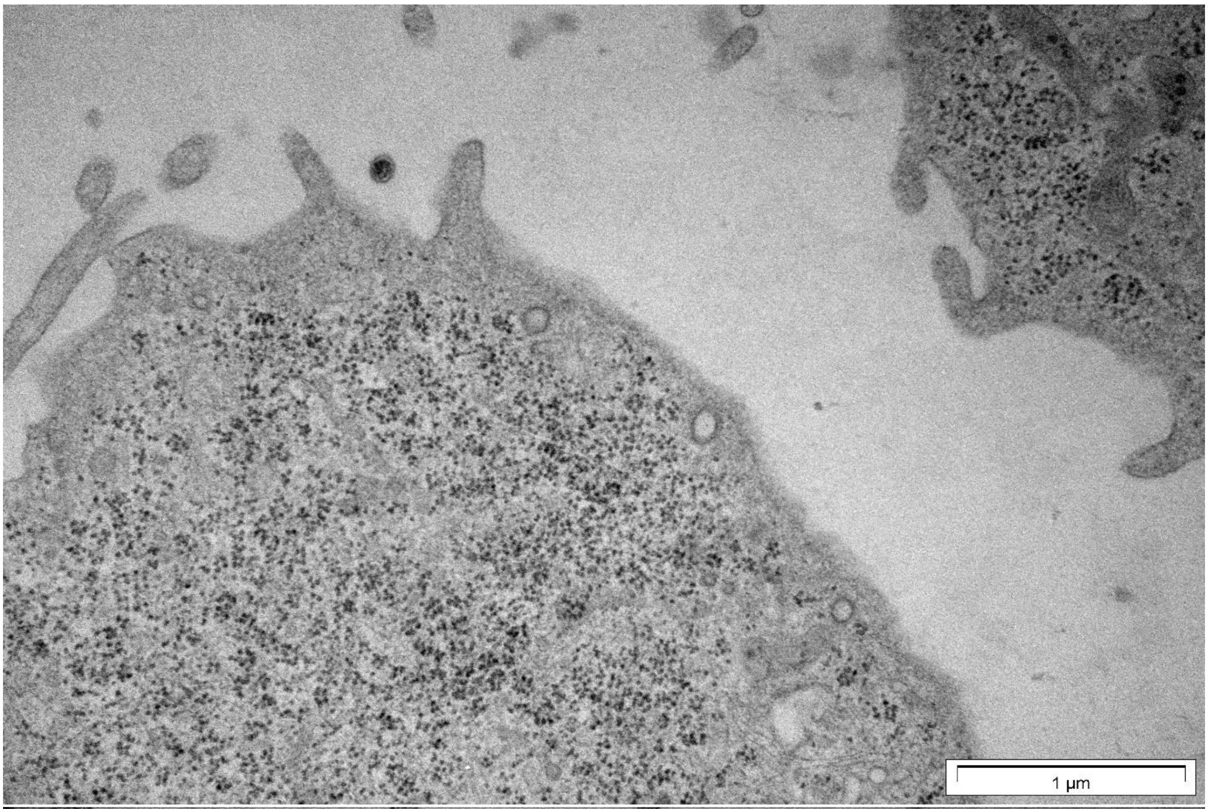
1 µm

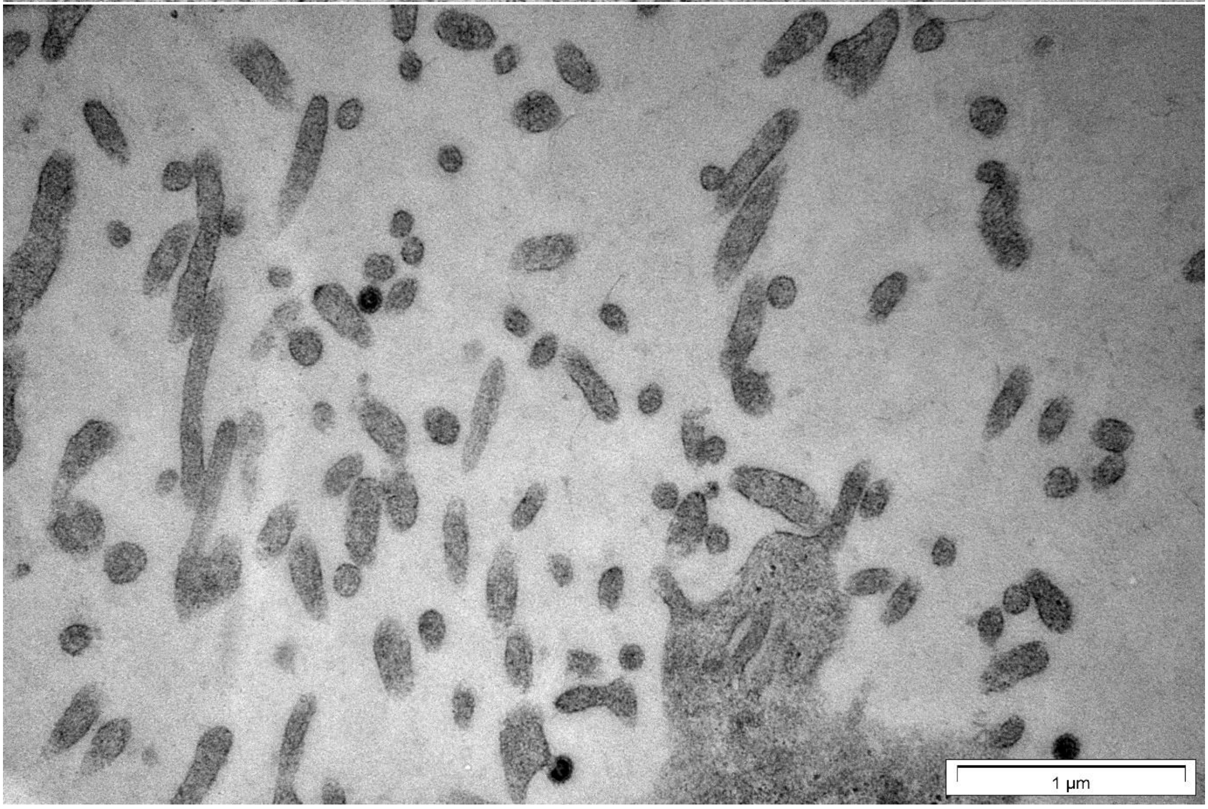
1 µm

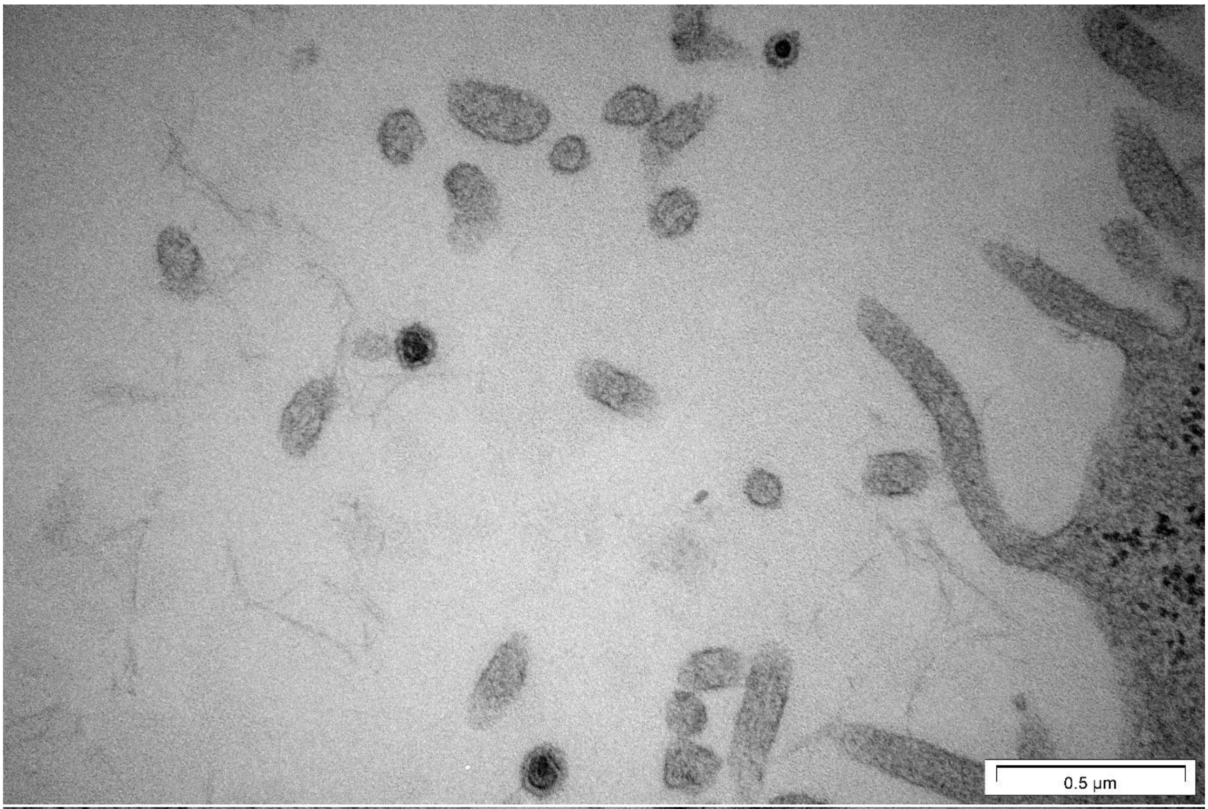
0.5 µm

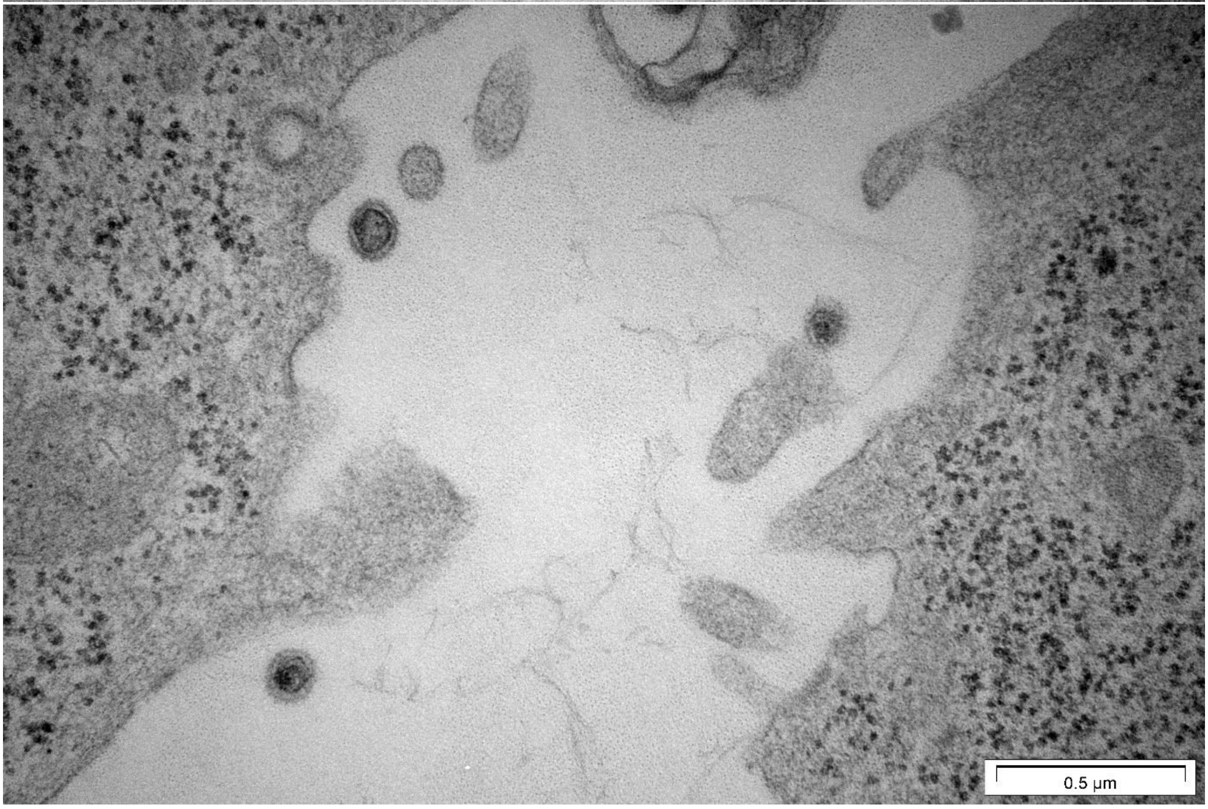
0.5 µm

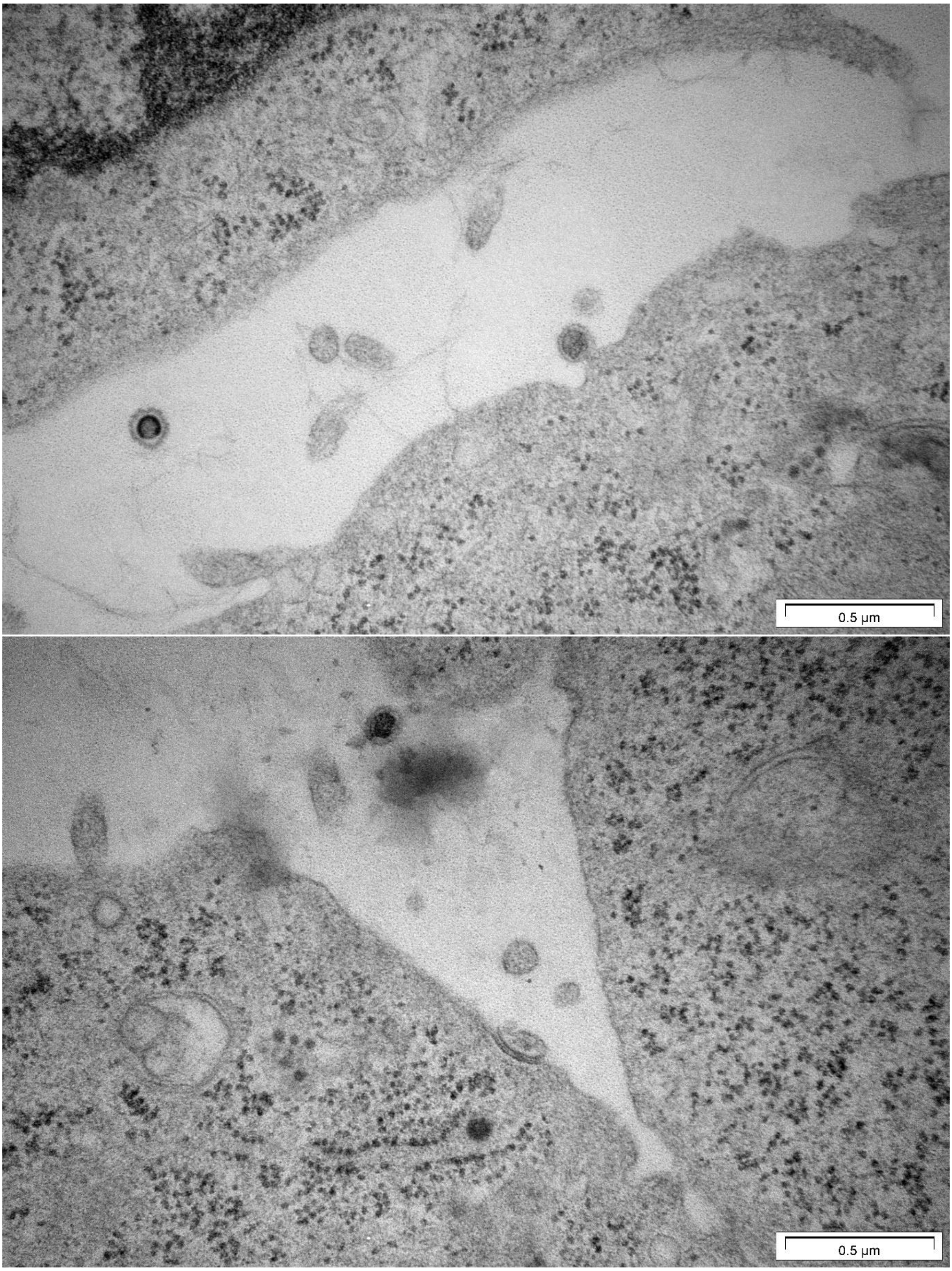

0.5 µm
0.5 µm

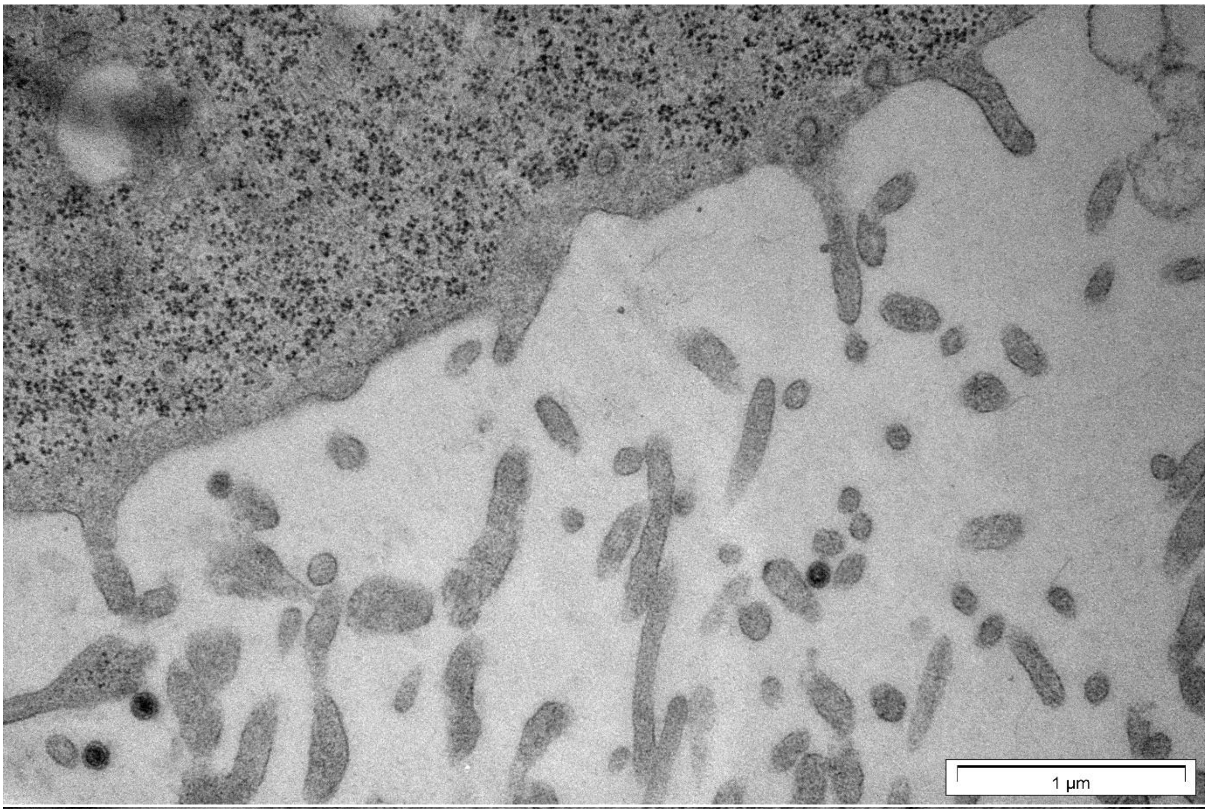
1 µm
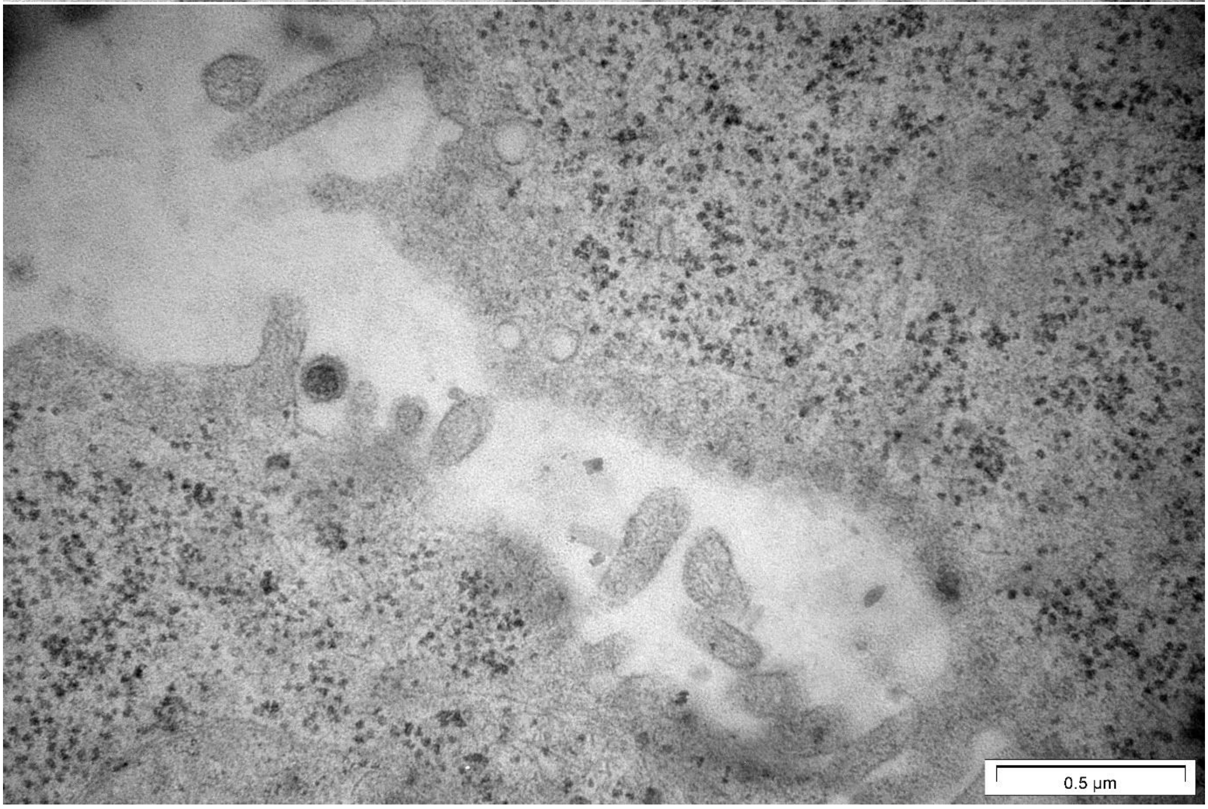
0.5 µm

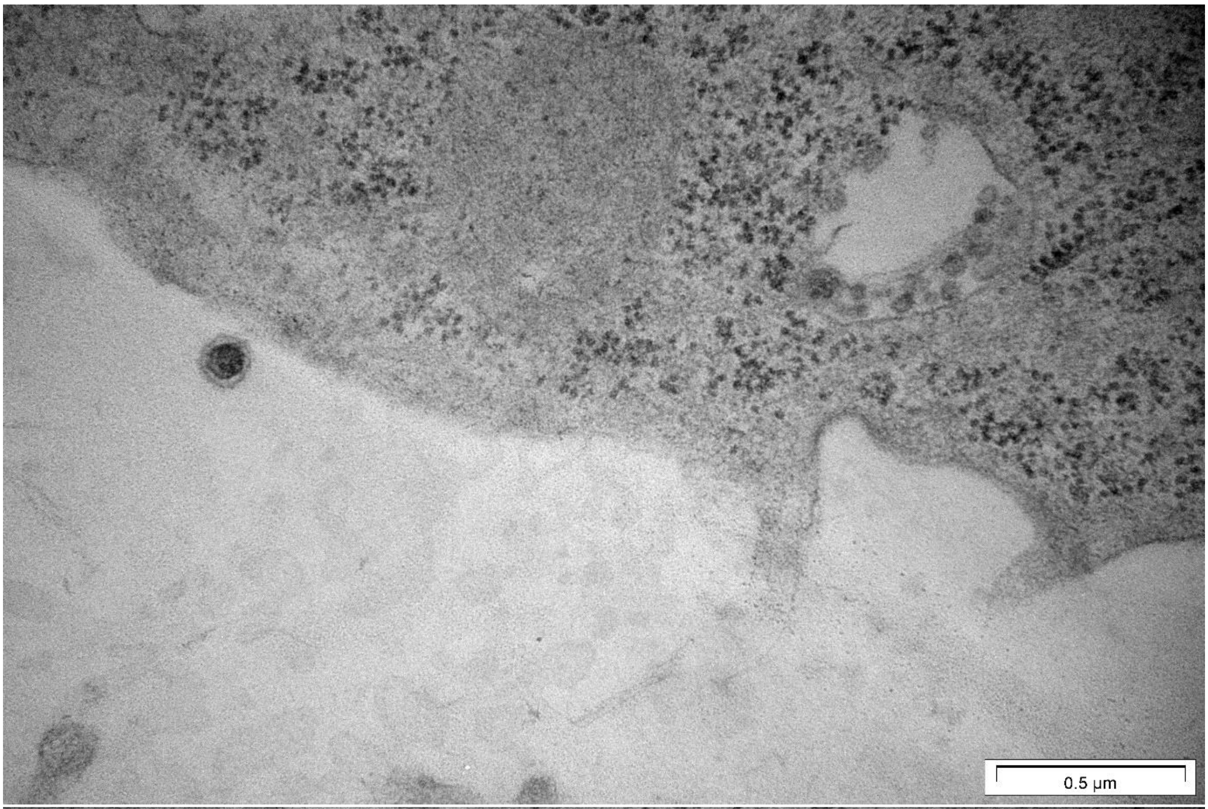
0.5 µm

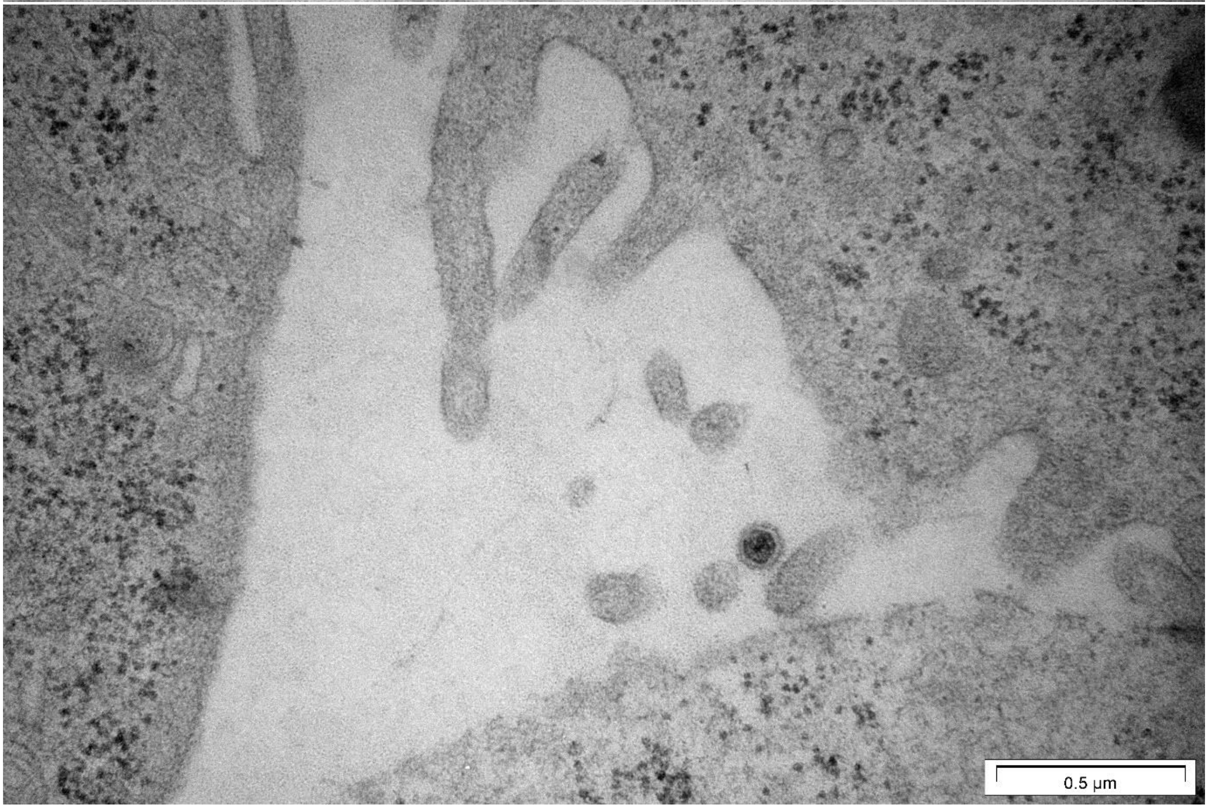
0.5 µm

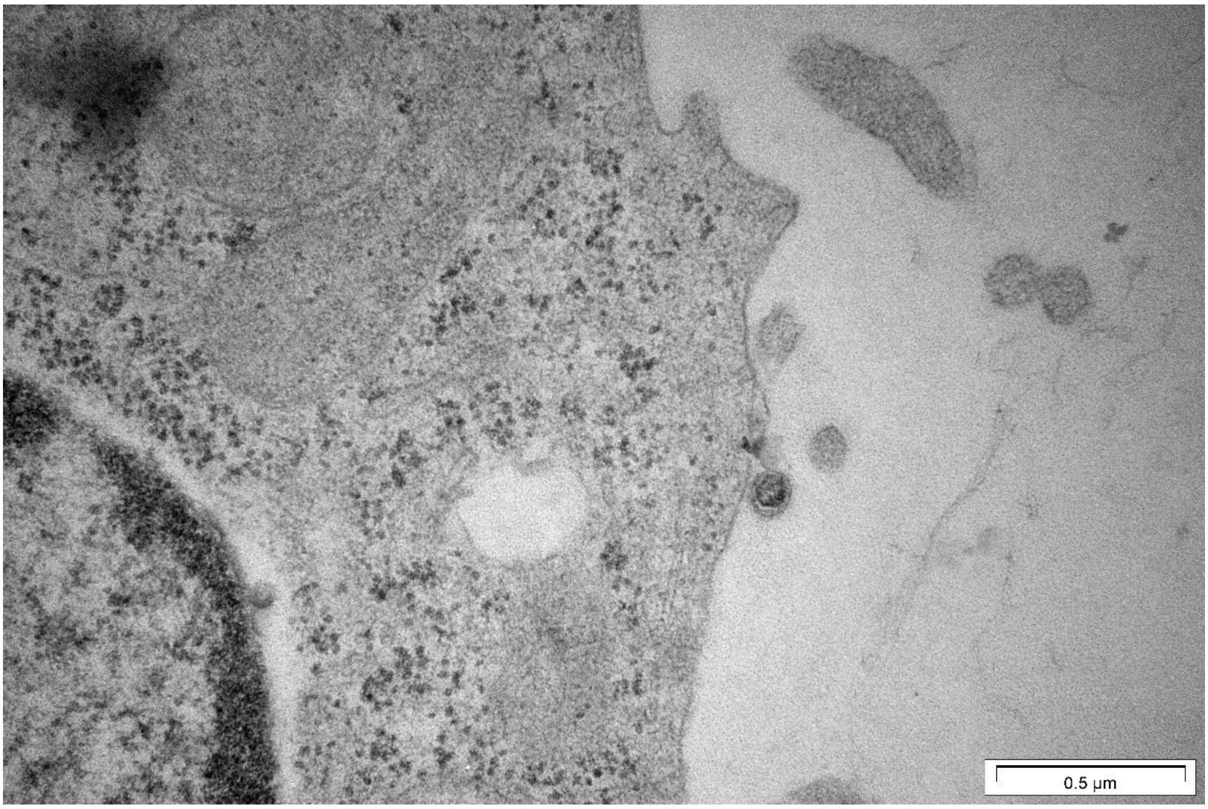
0.5 µm

Dextran sulfate, 15 min.

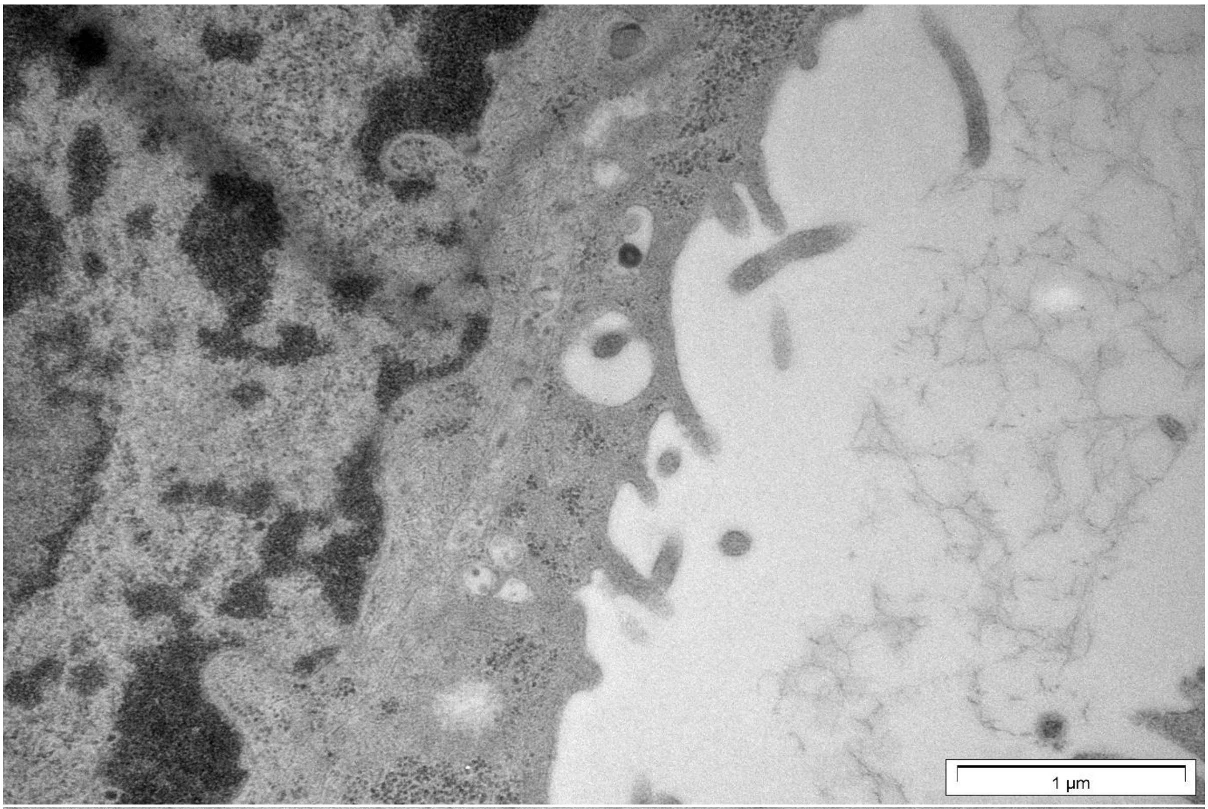

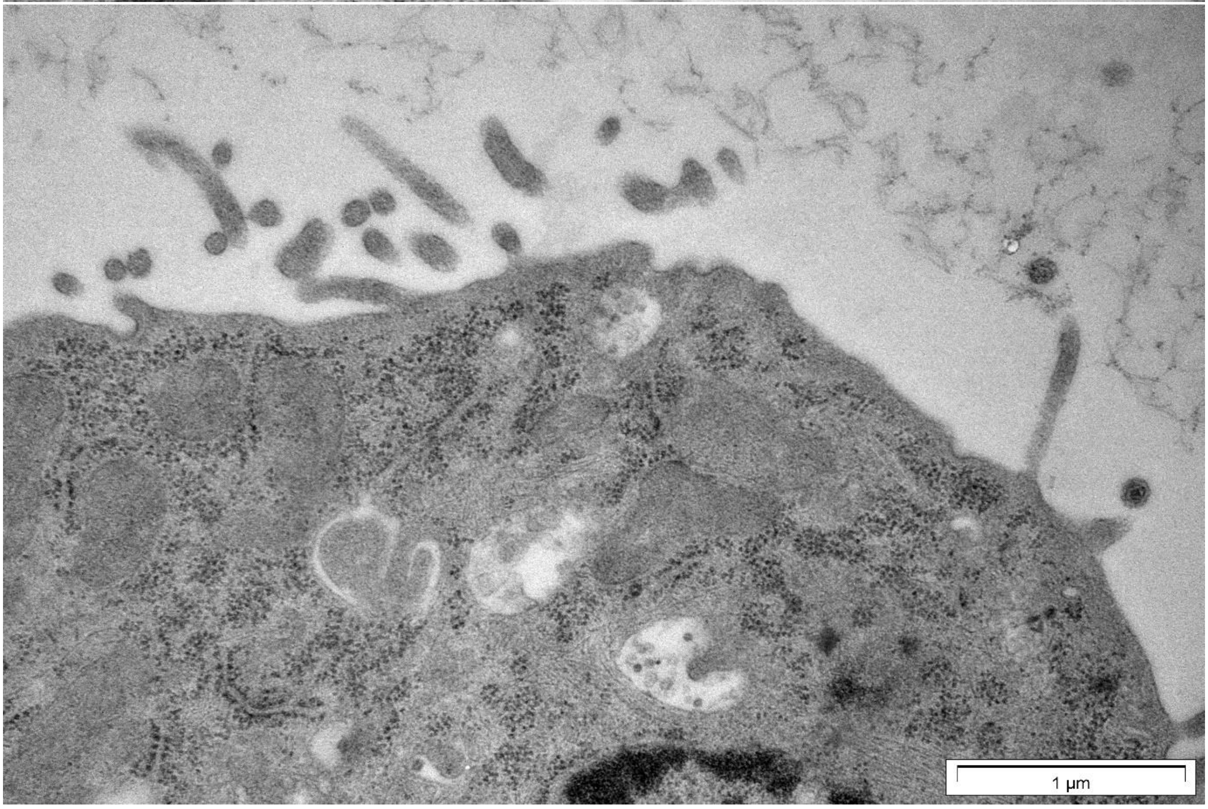

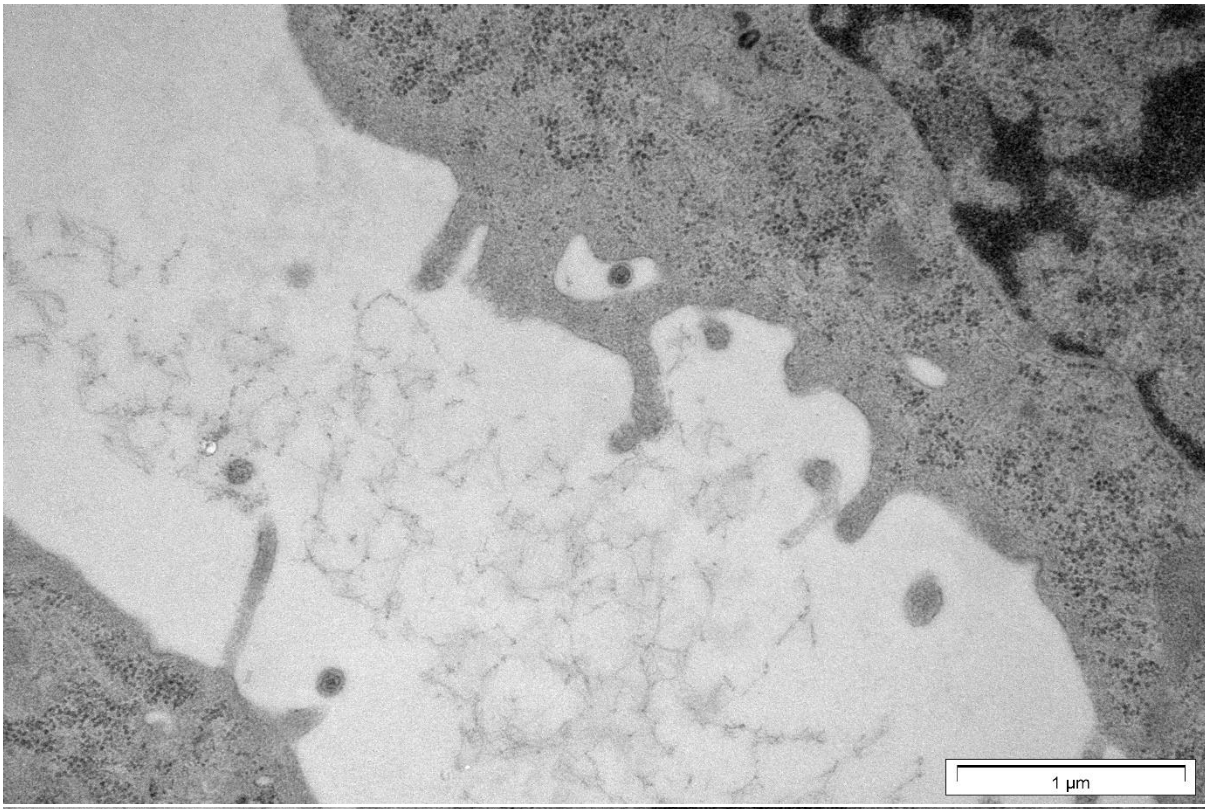
1 µm

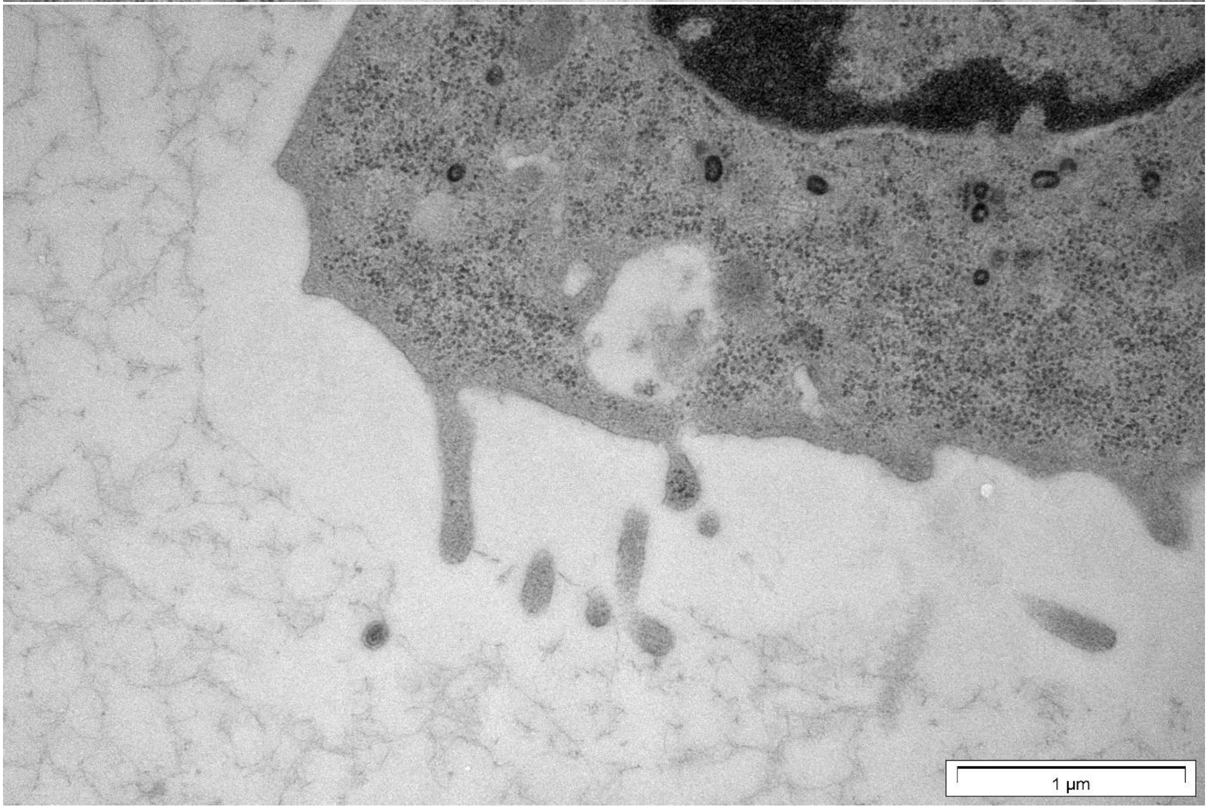
1 µm

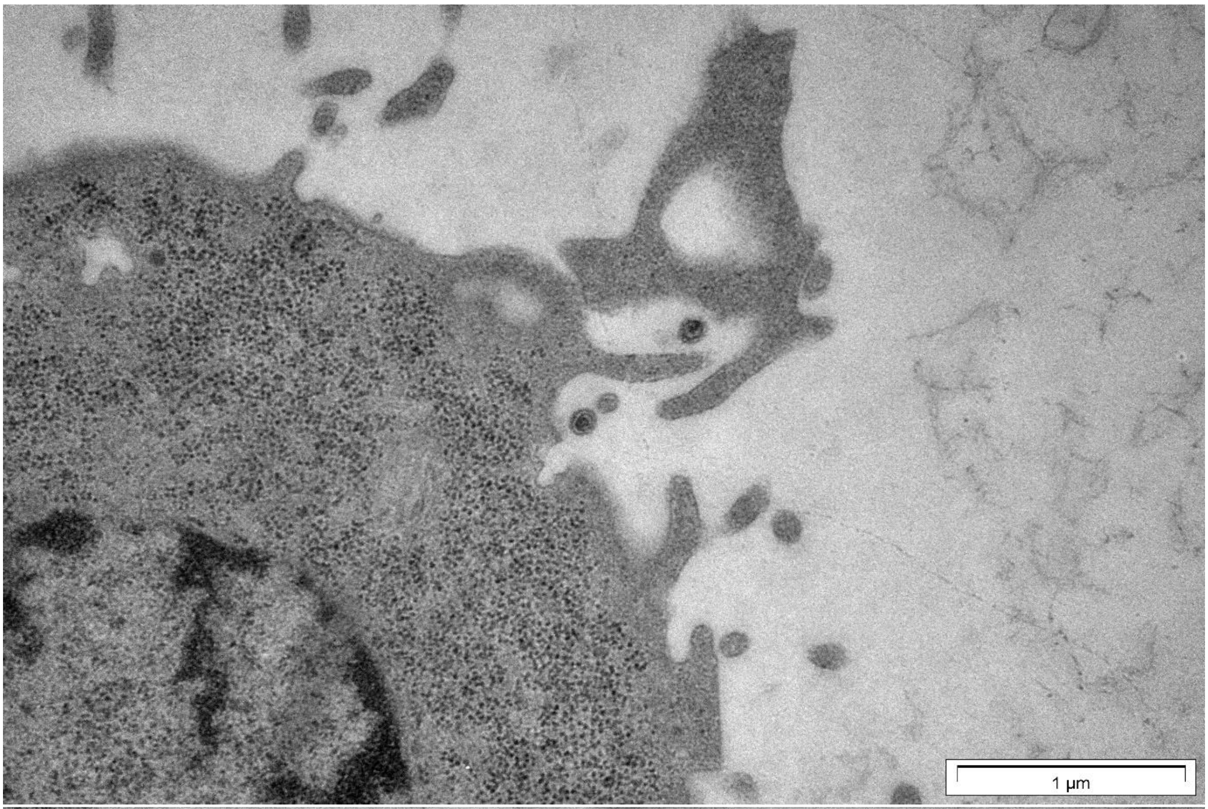
1 µm

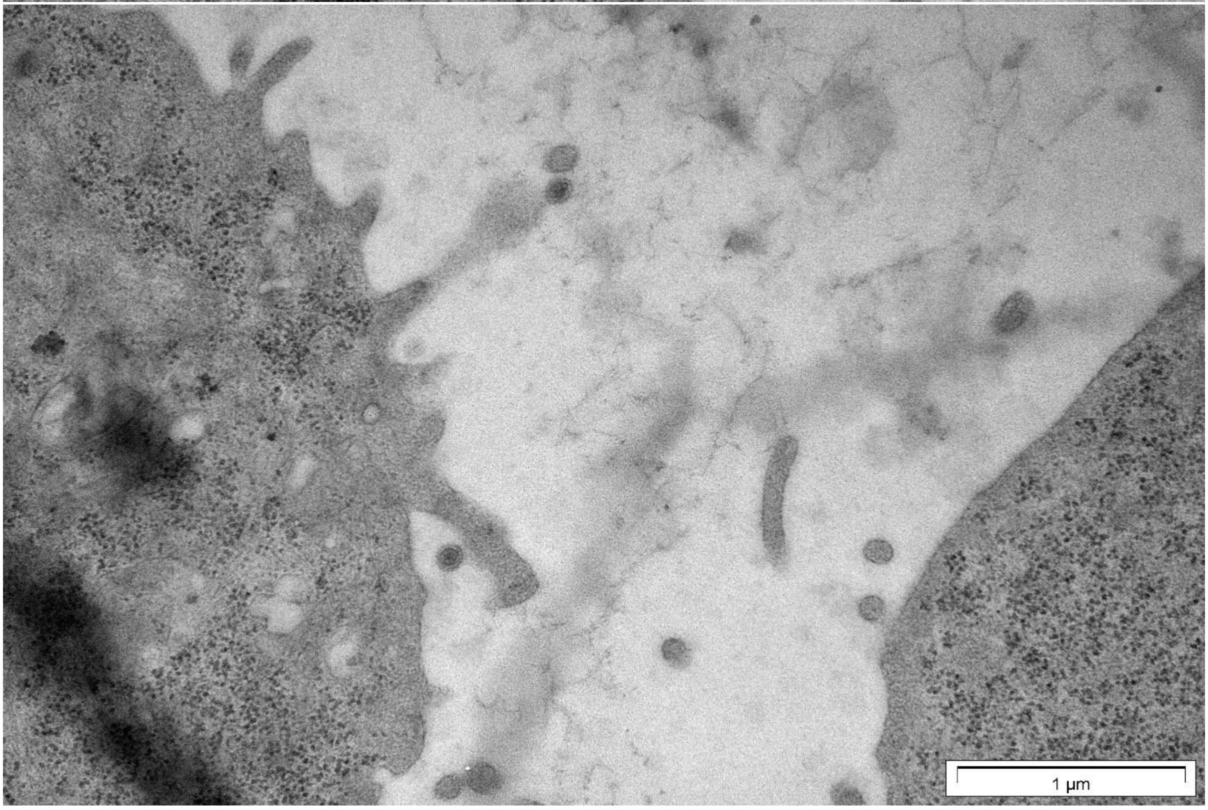
1 µm

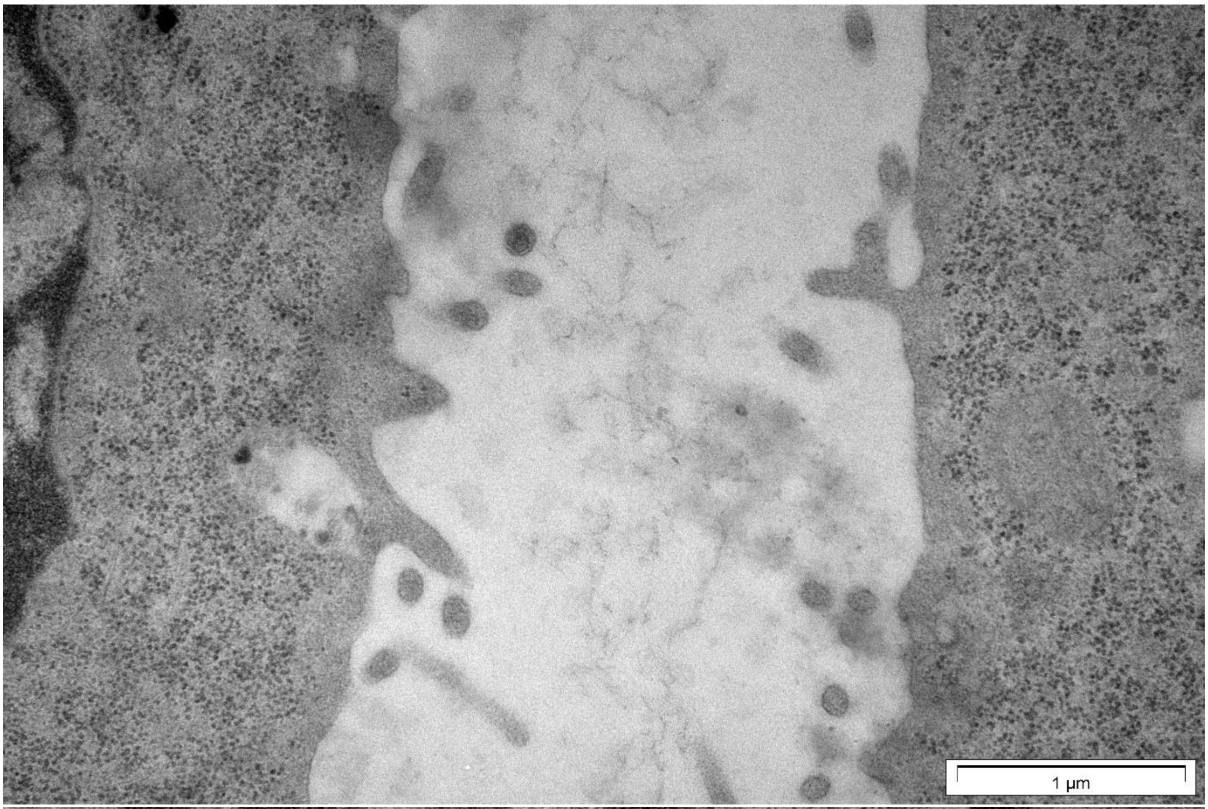
1 µm

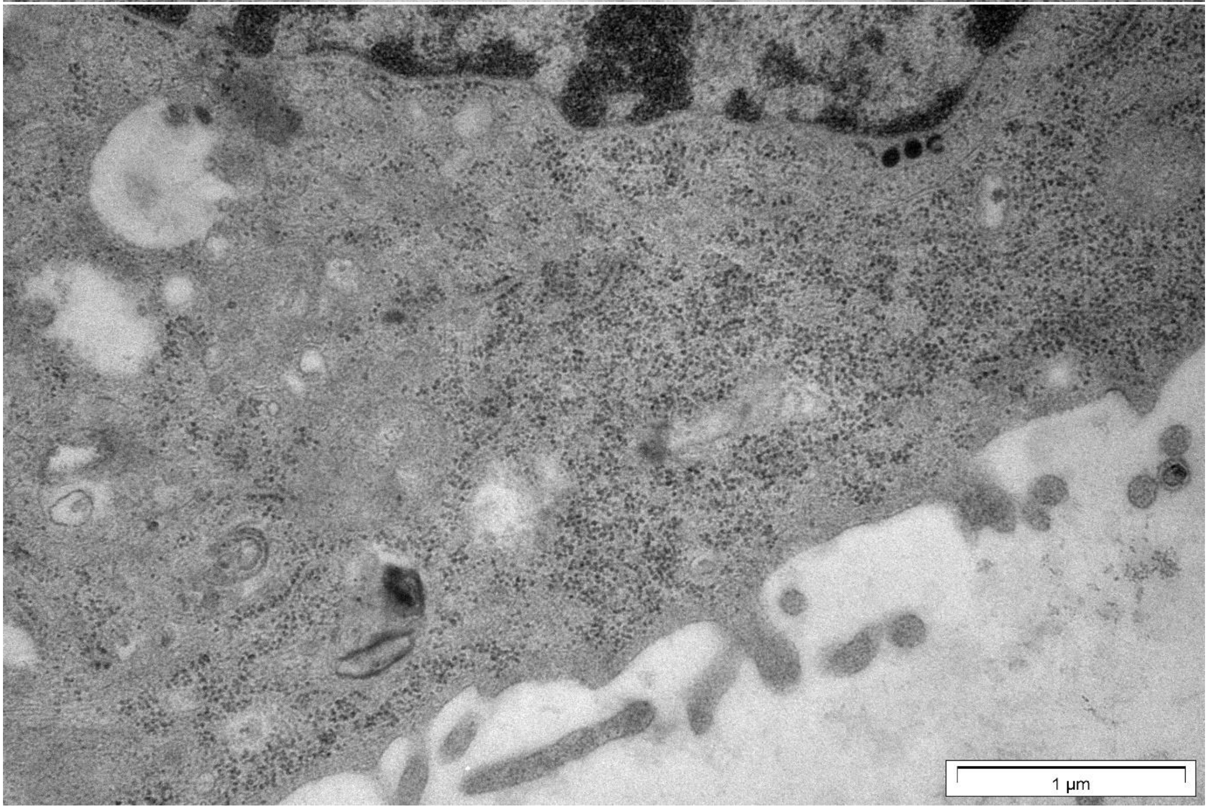
1 µm

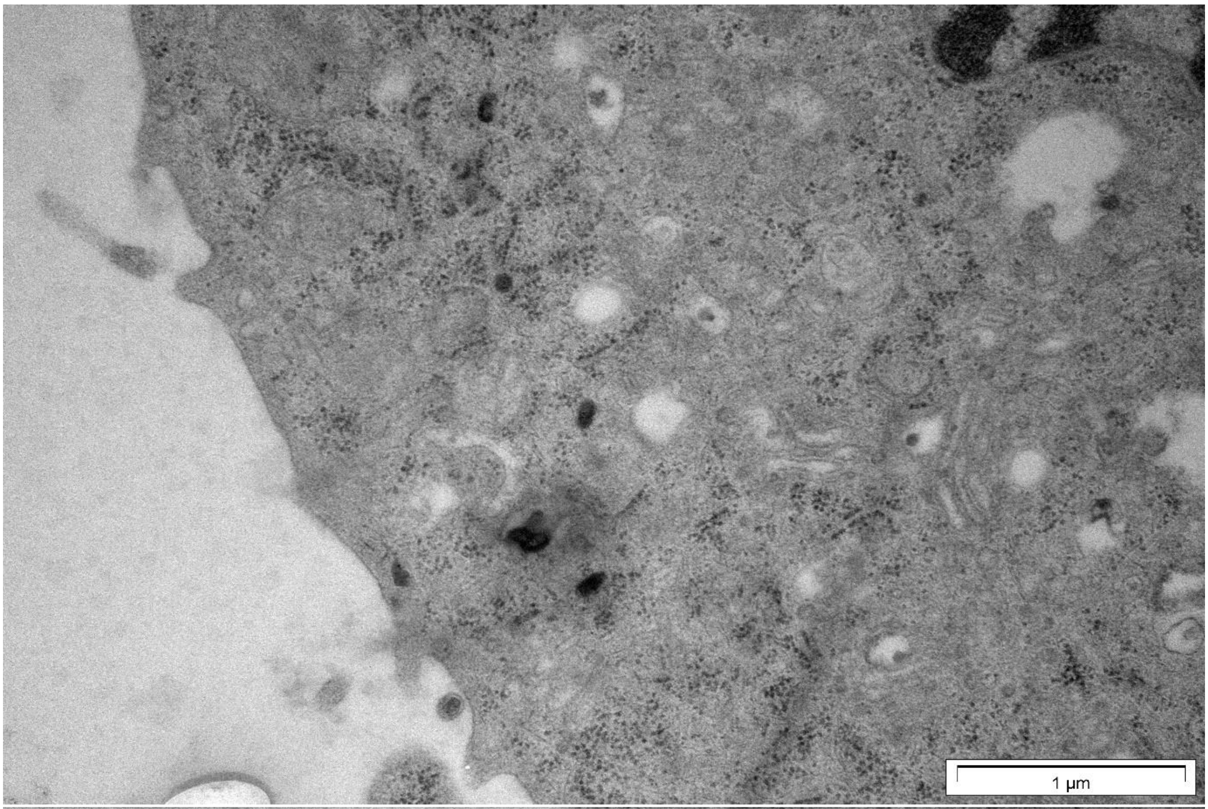
1 µm

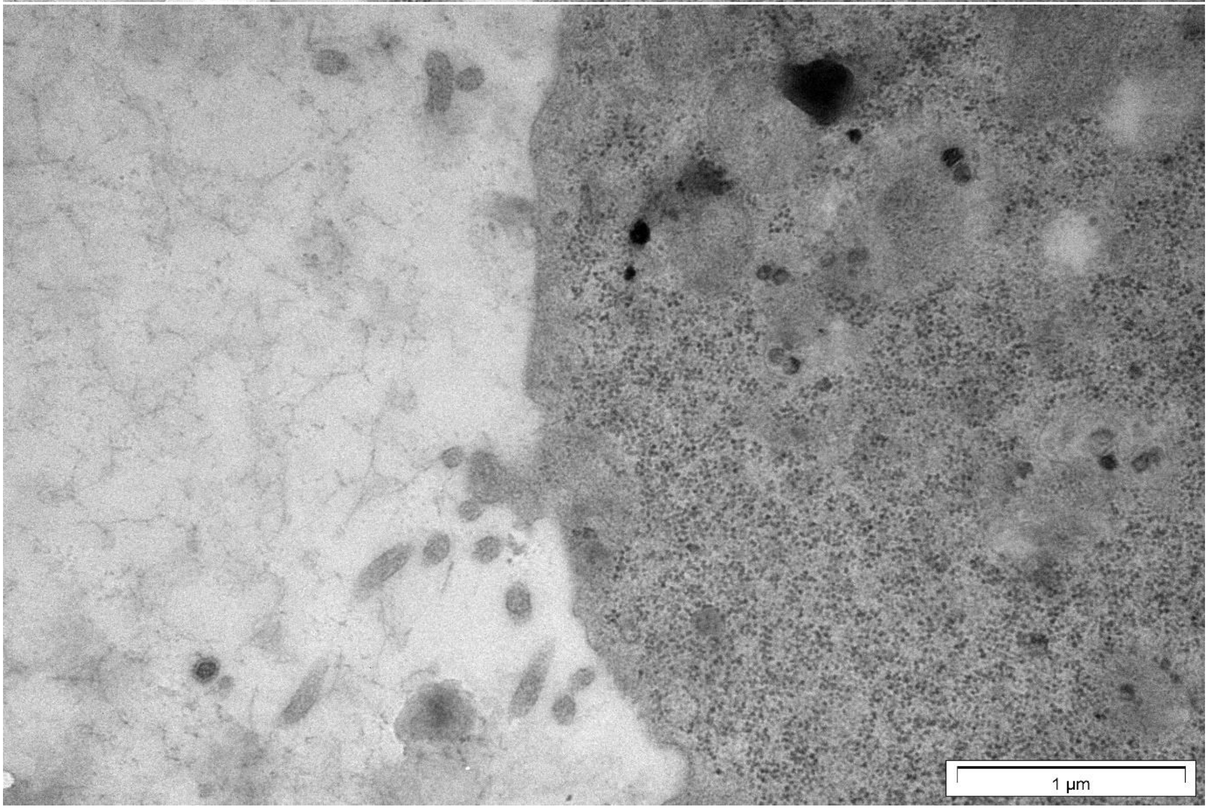
1 µm

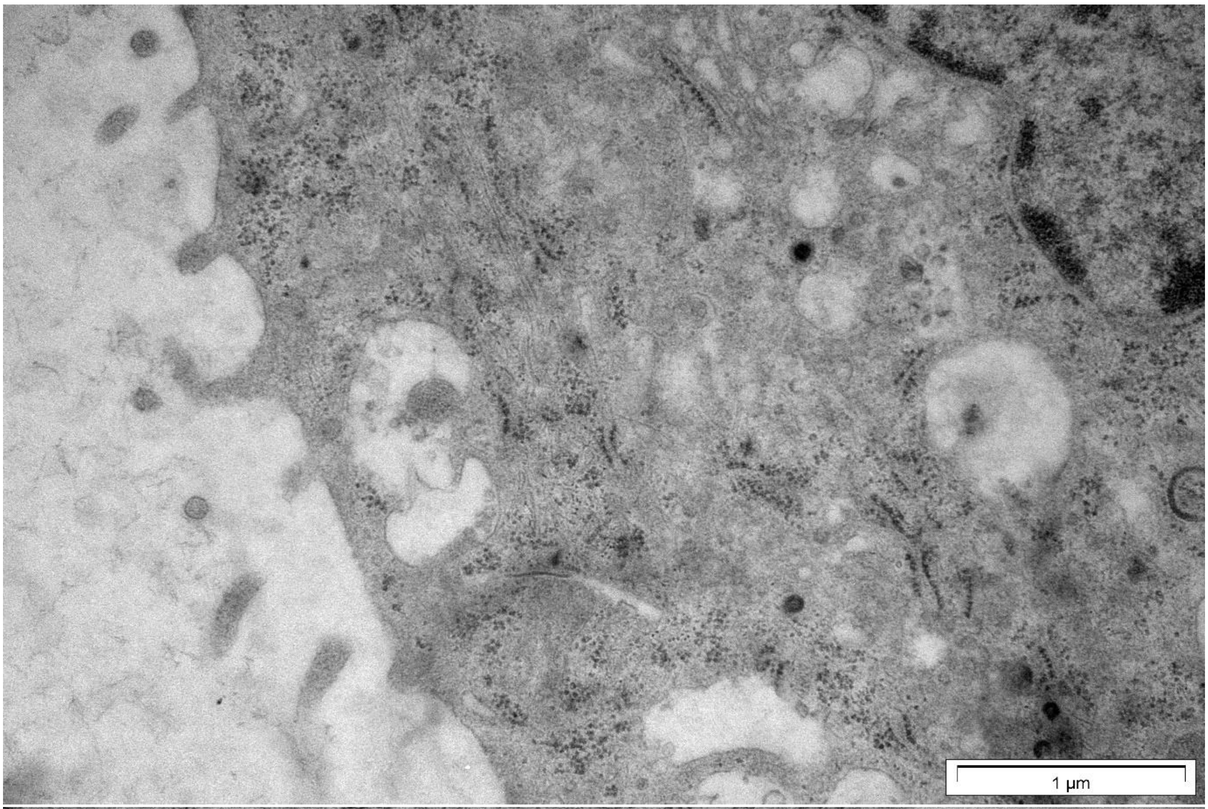
1 µm

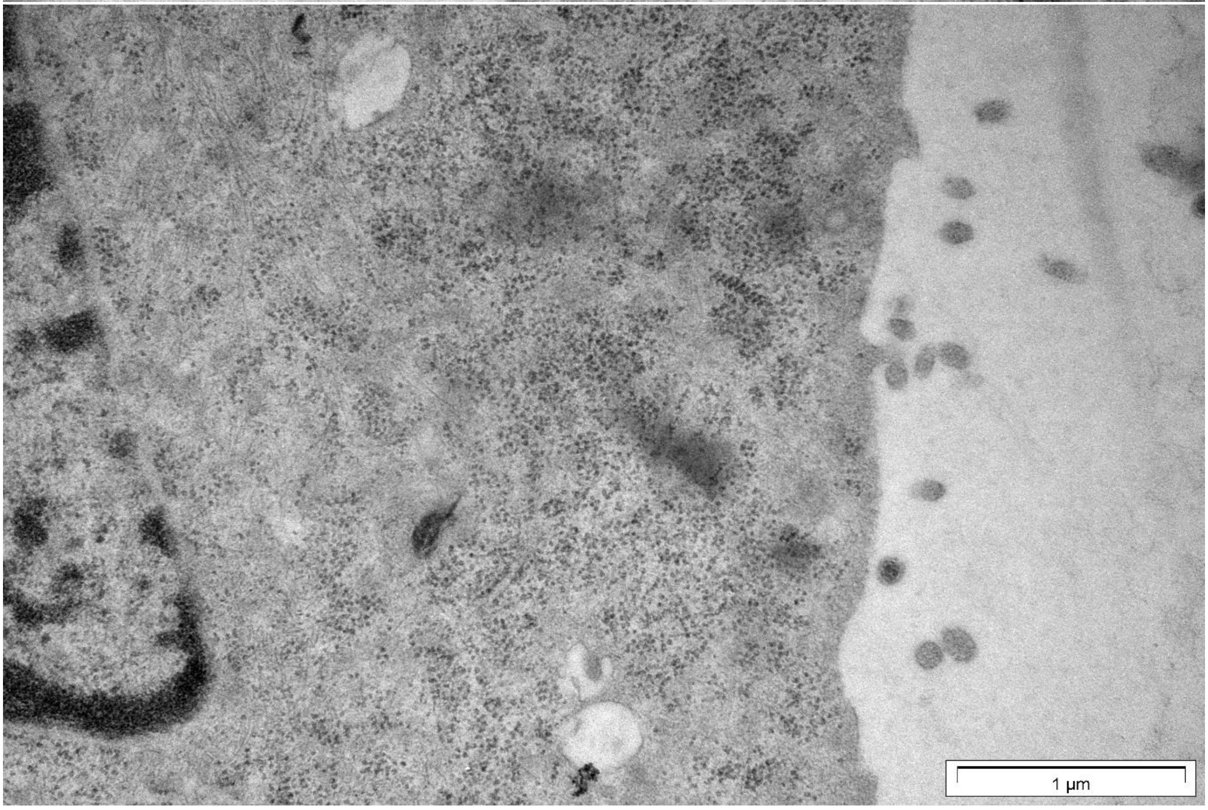
1 µm

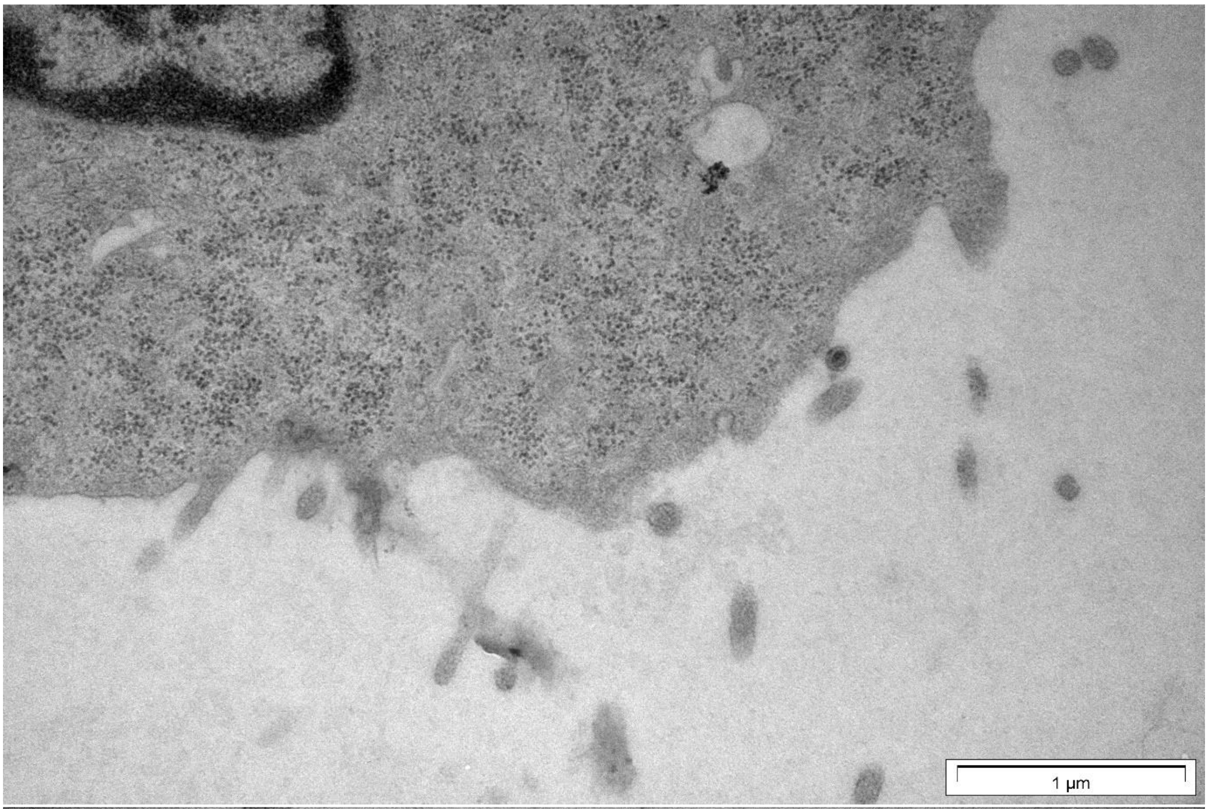
1 µm

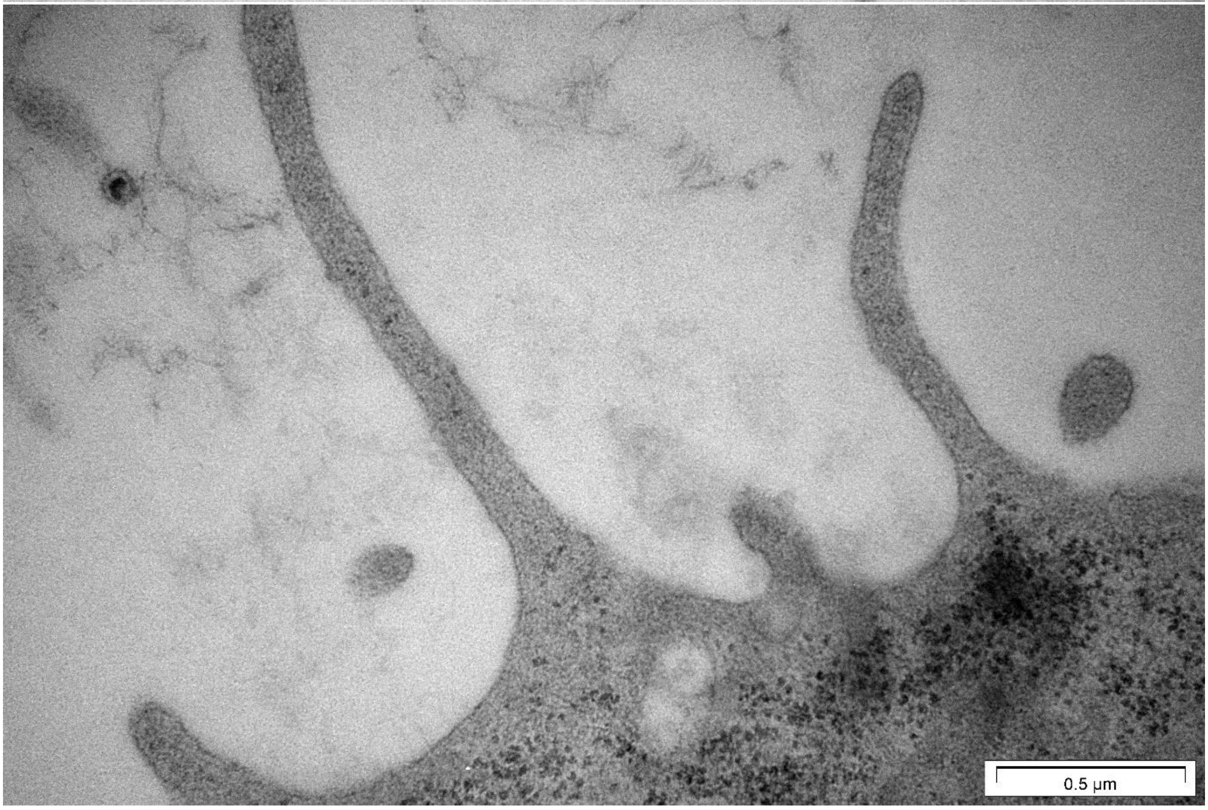
0.5 µm

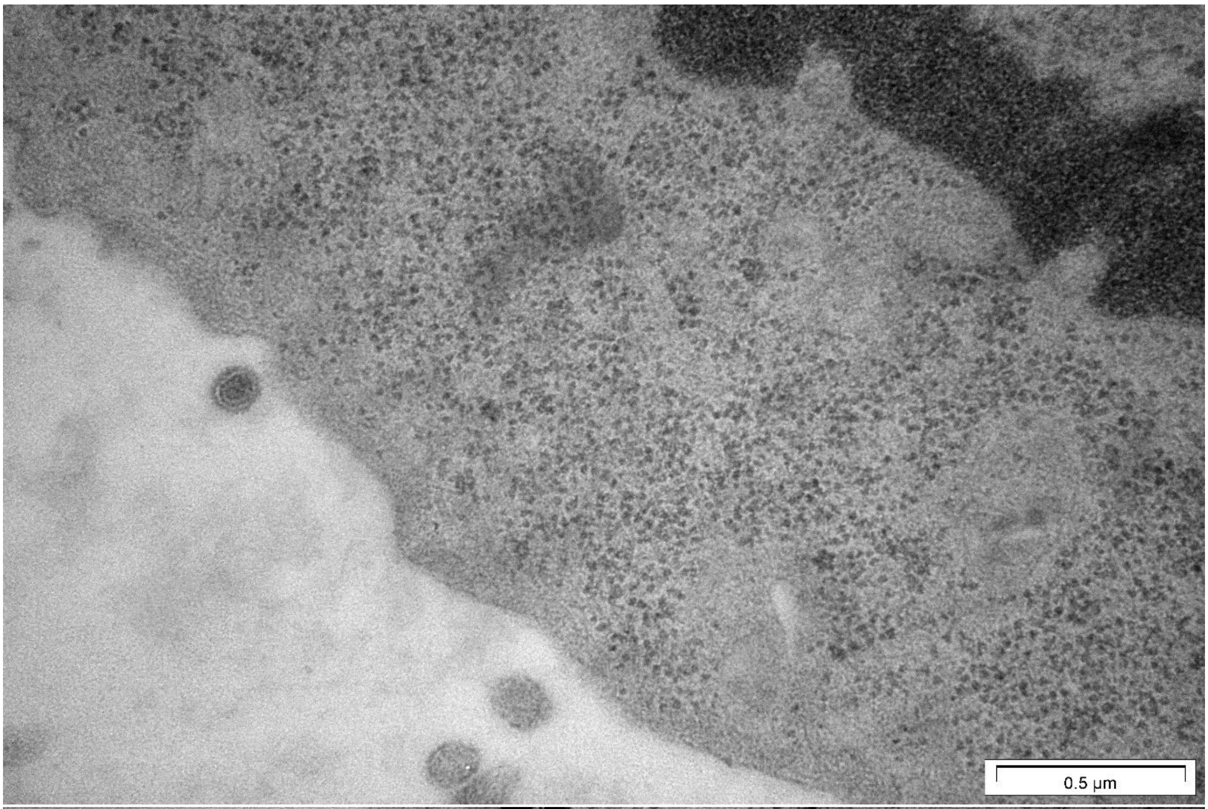
0.5 µm

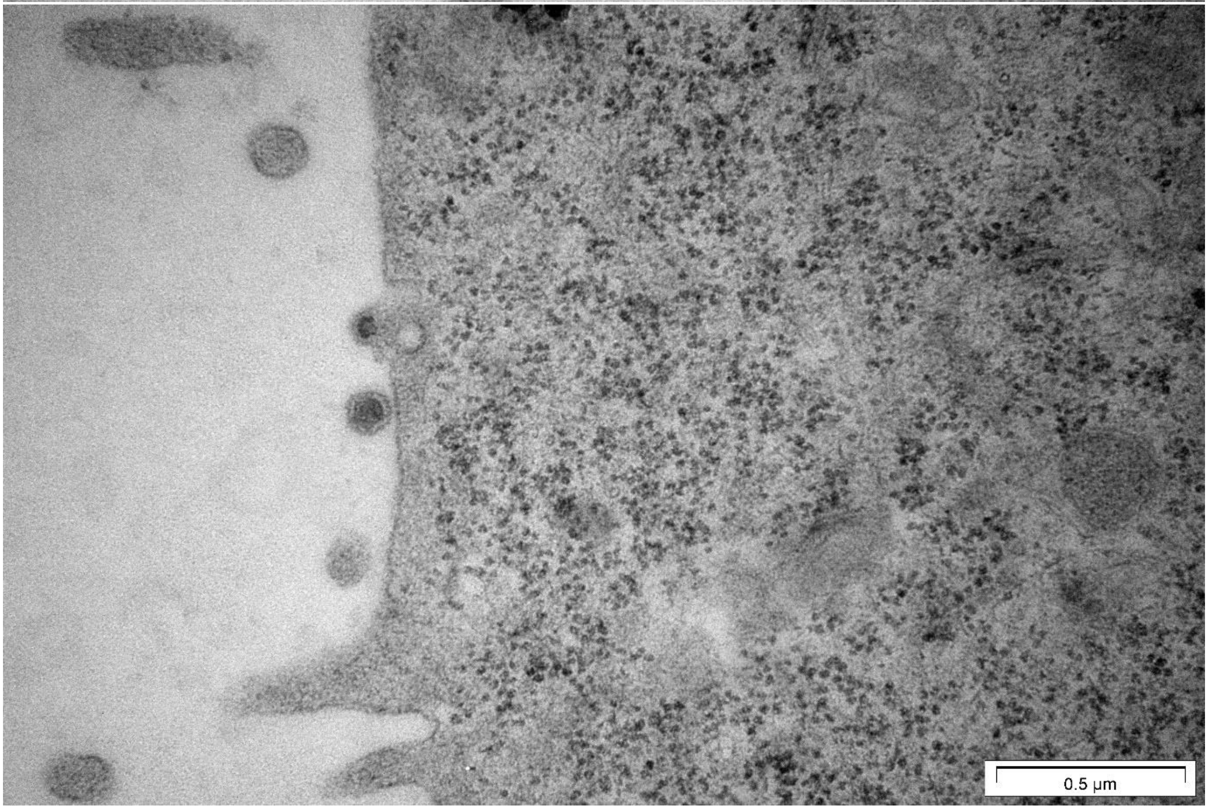
0.5 µm

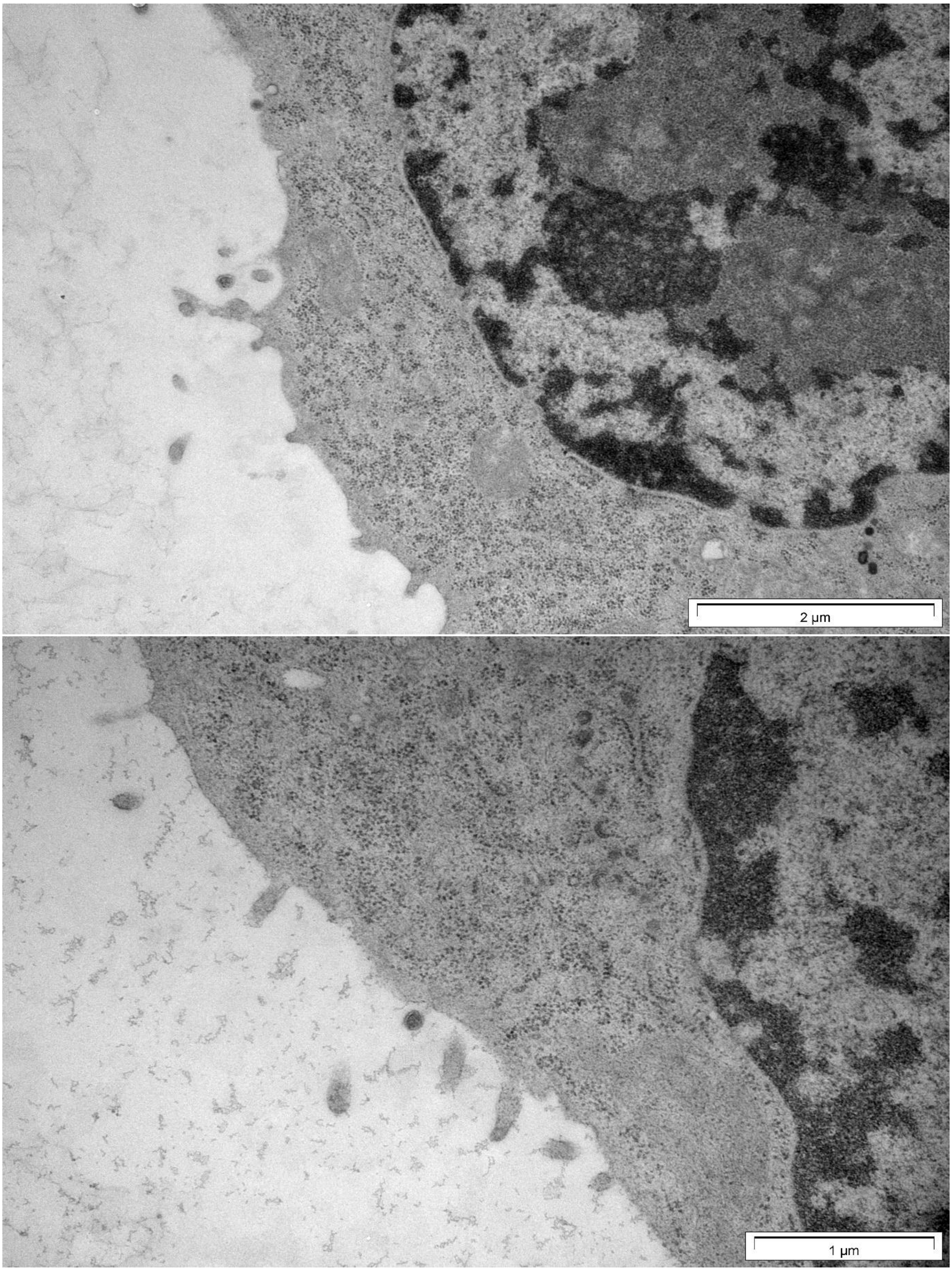
2 µm
1 µm

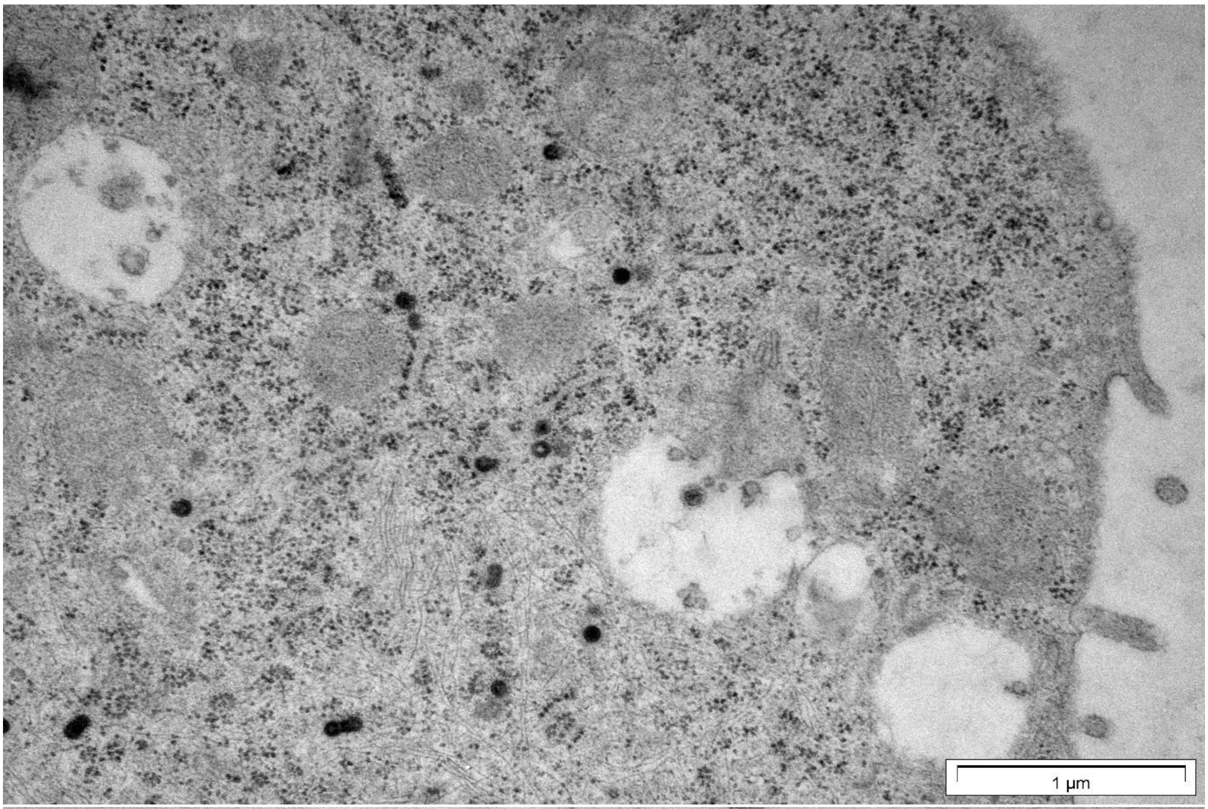
1 µm

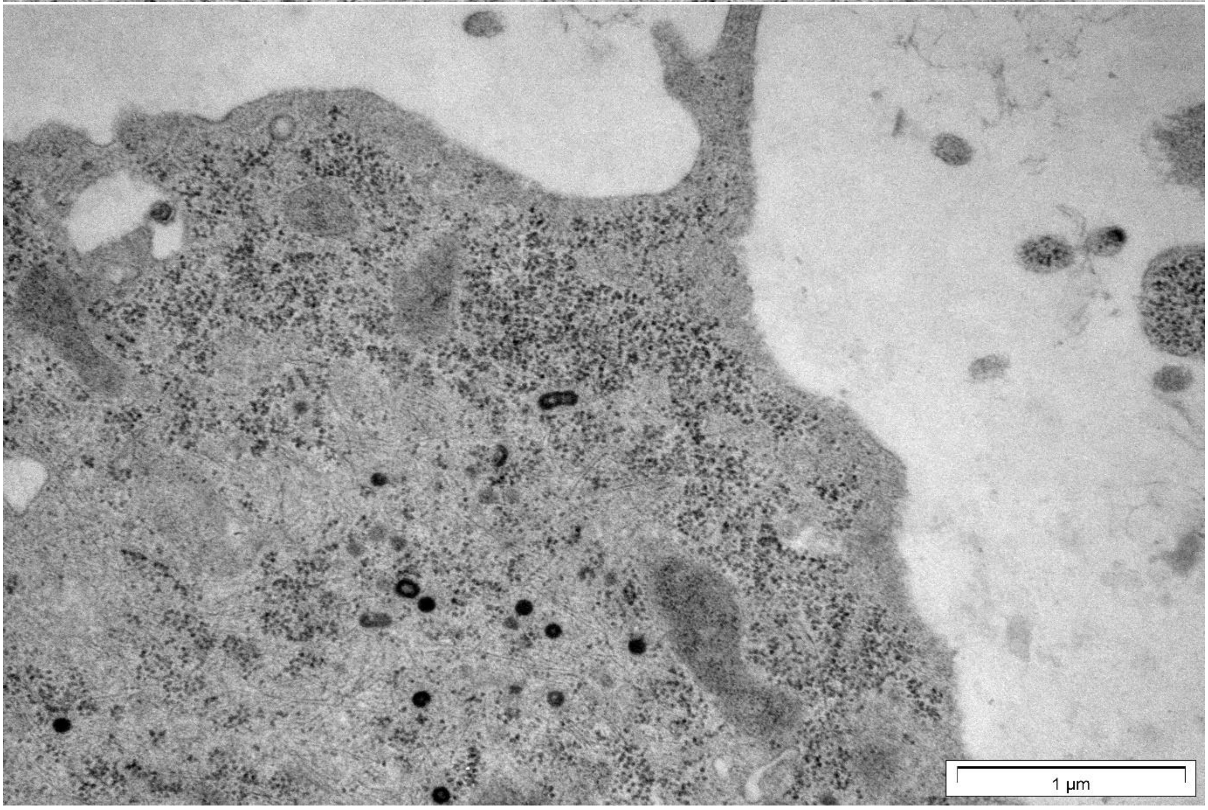
1 µm

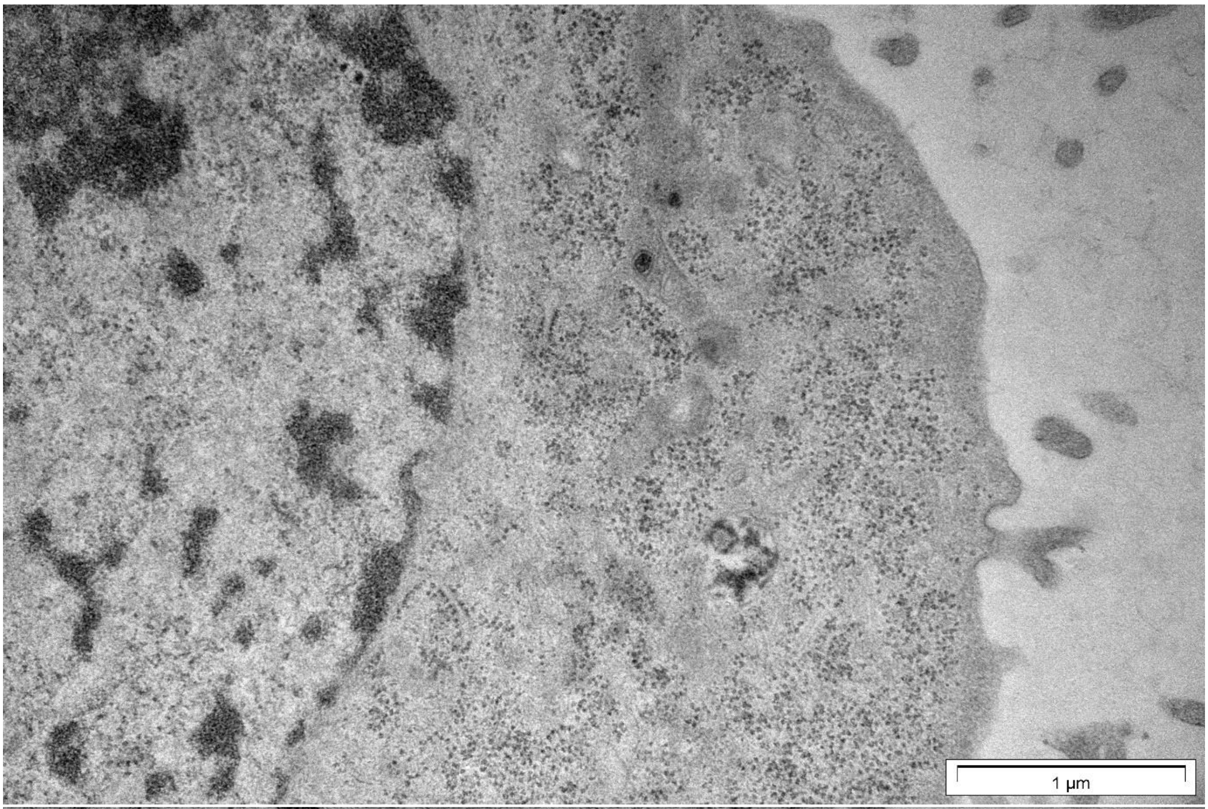
1 µm

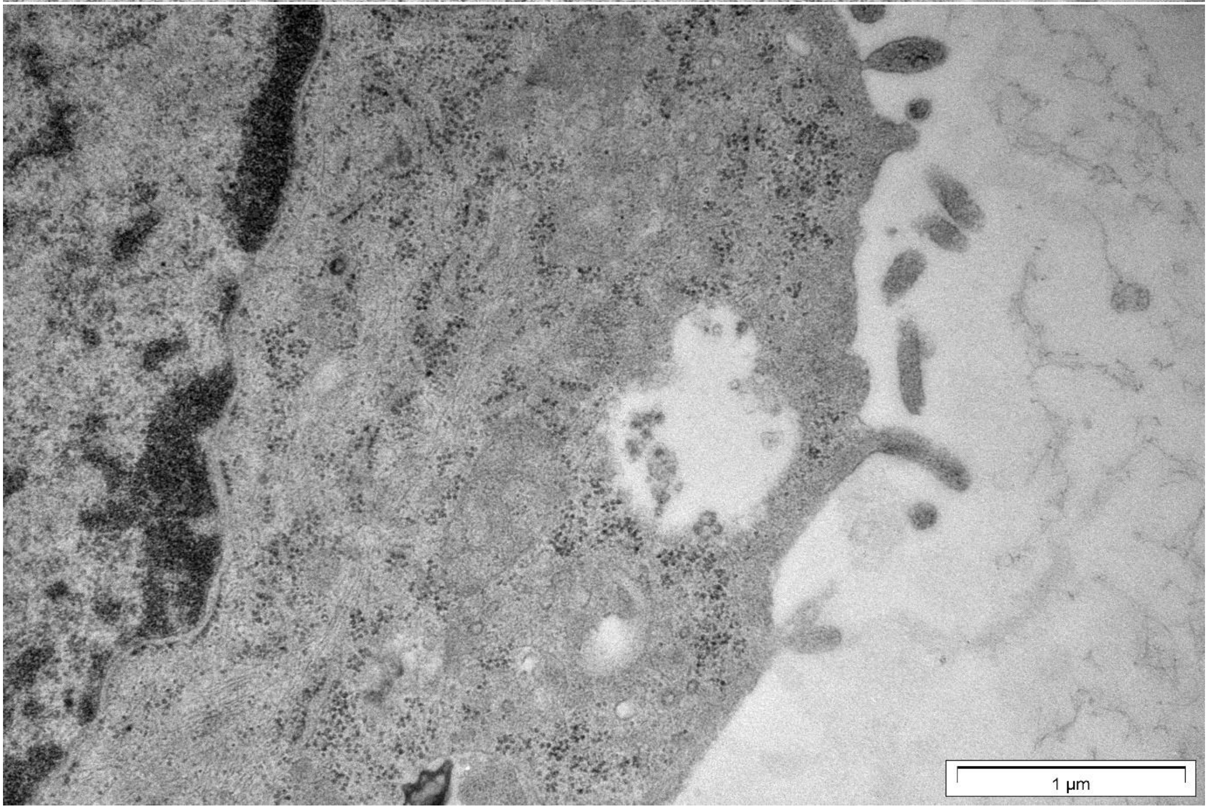
1 µm

<u>**Analysis 2023**</u>

Dextran, 1 min.

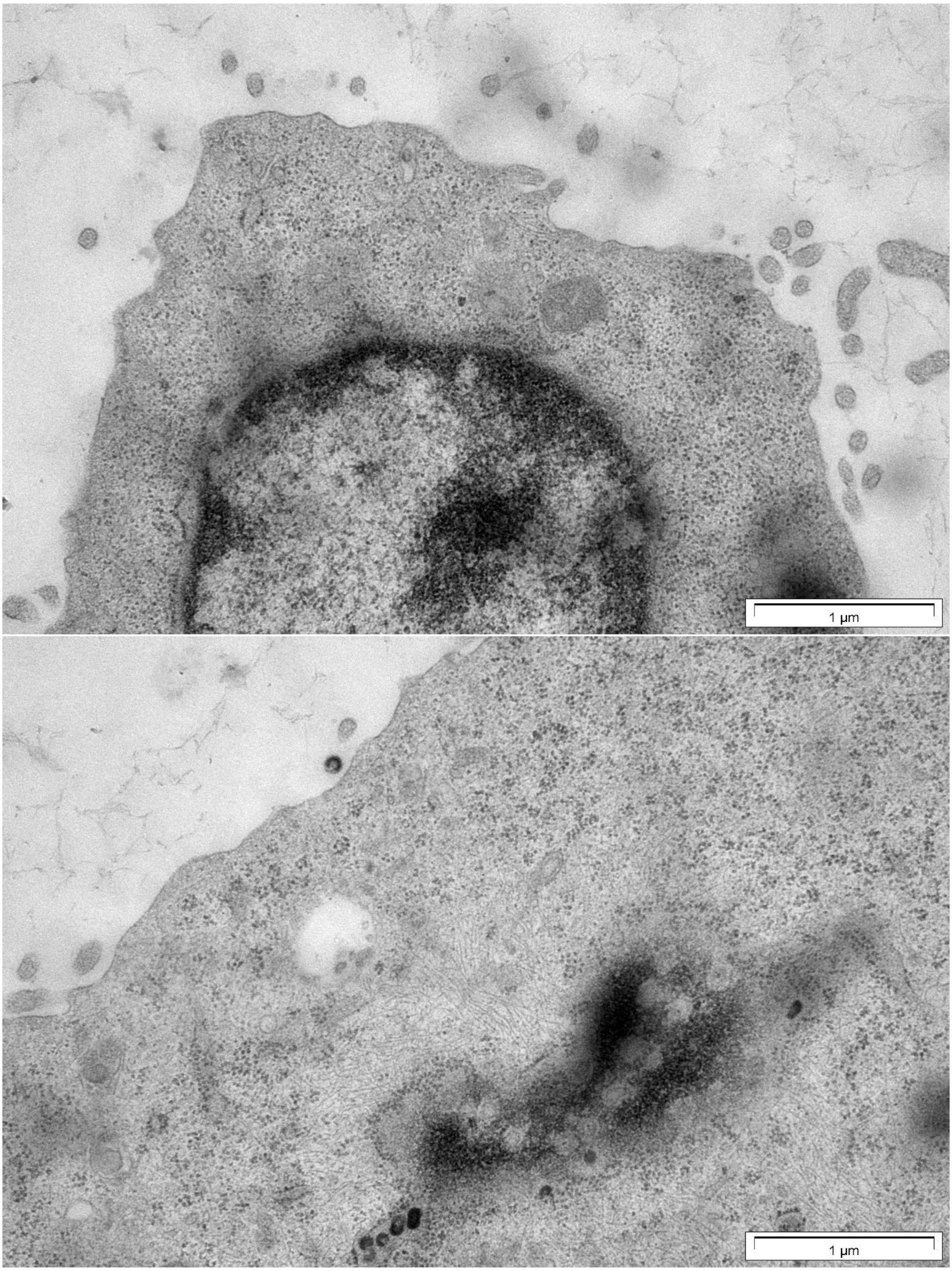

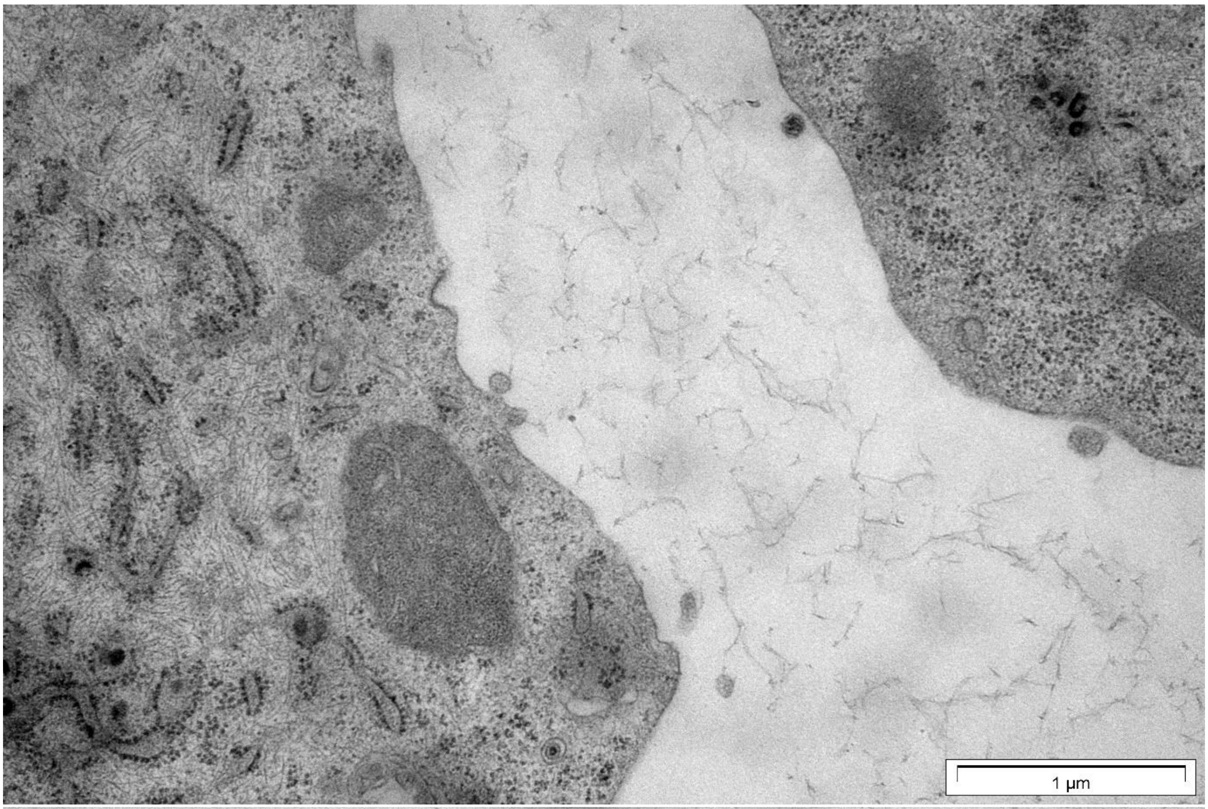
1 µm

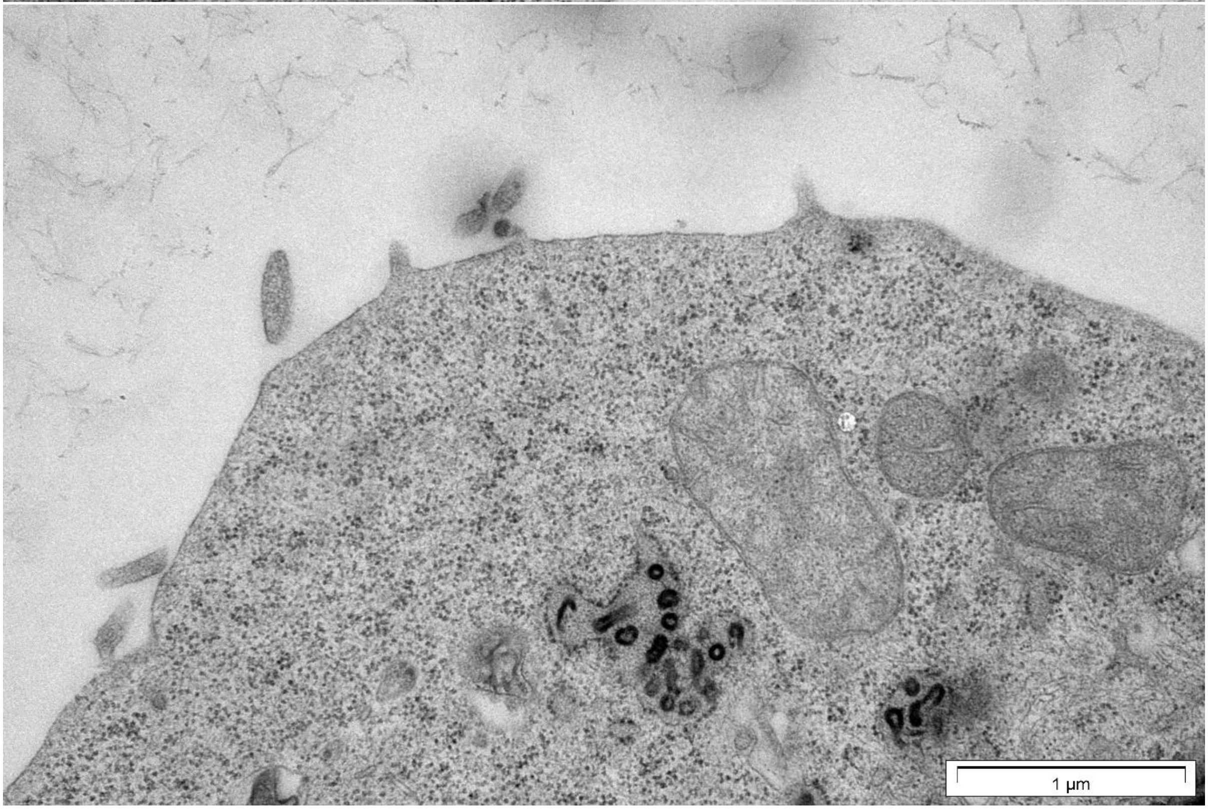
1 µm

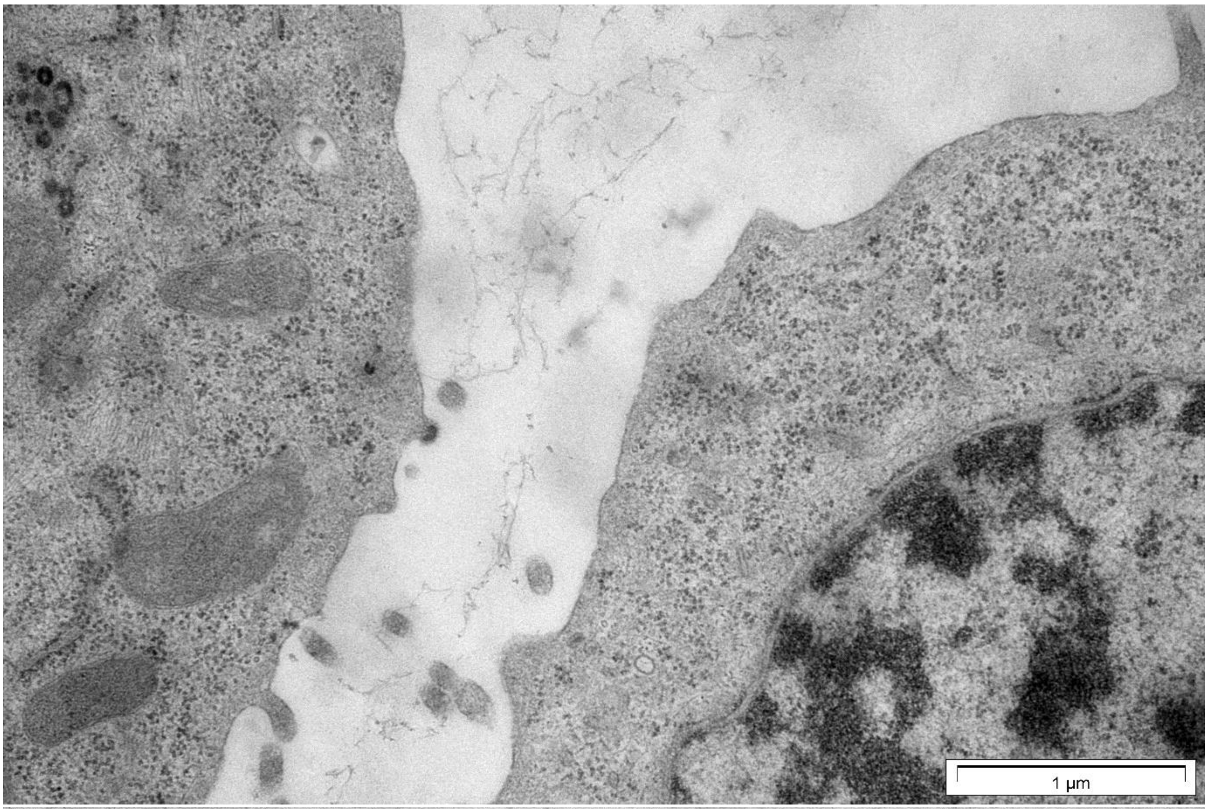
1 µm

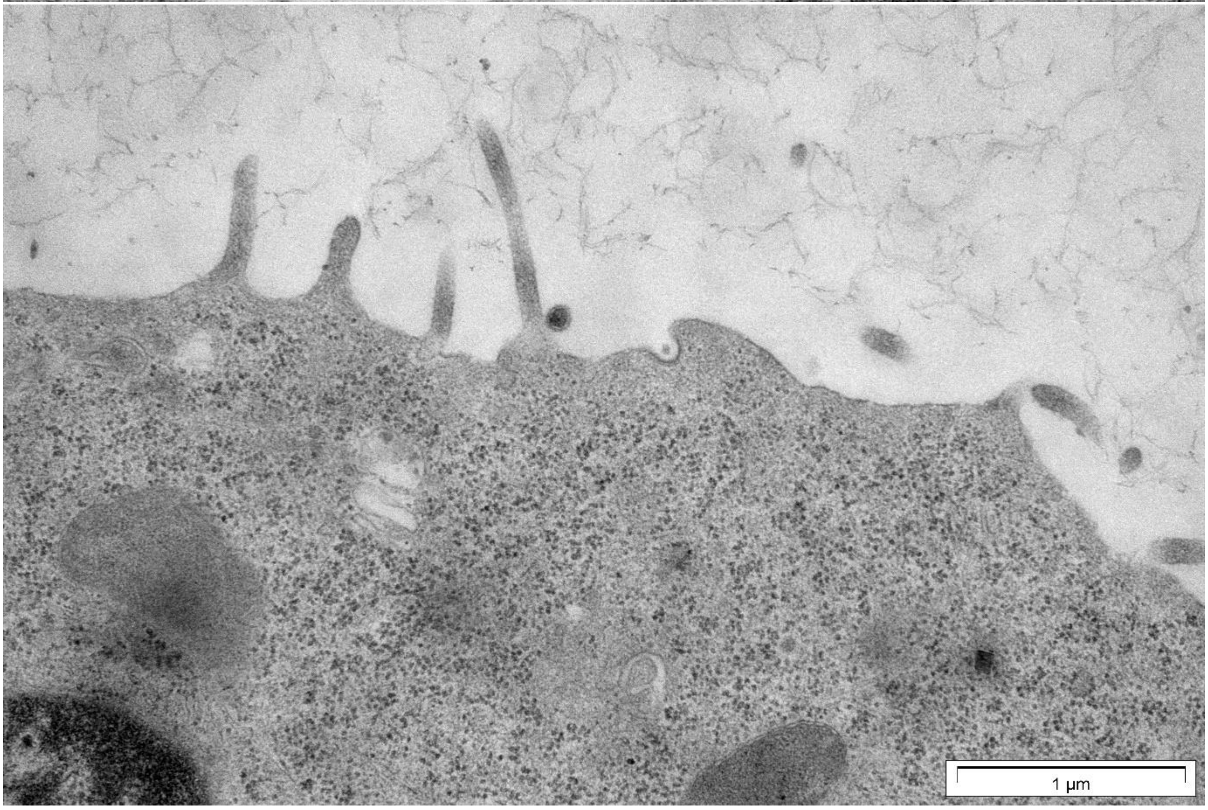
1 µm

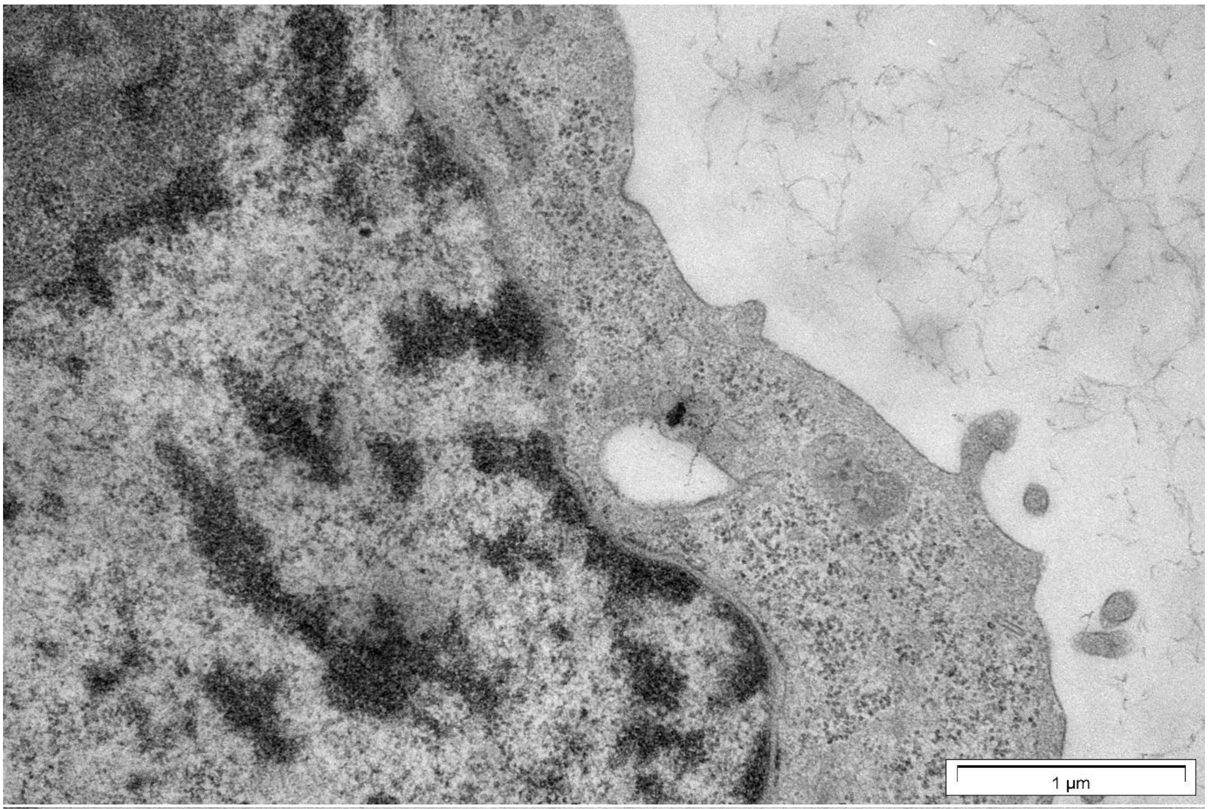
1 µm

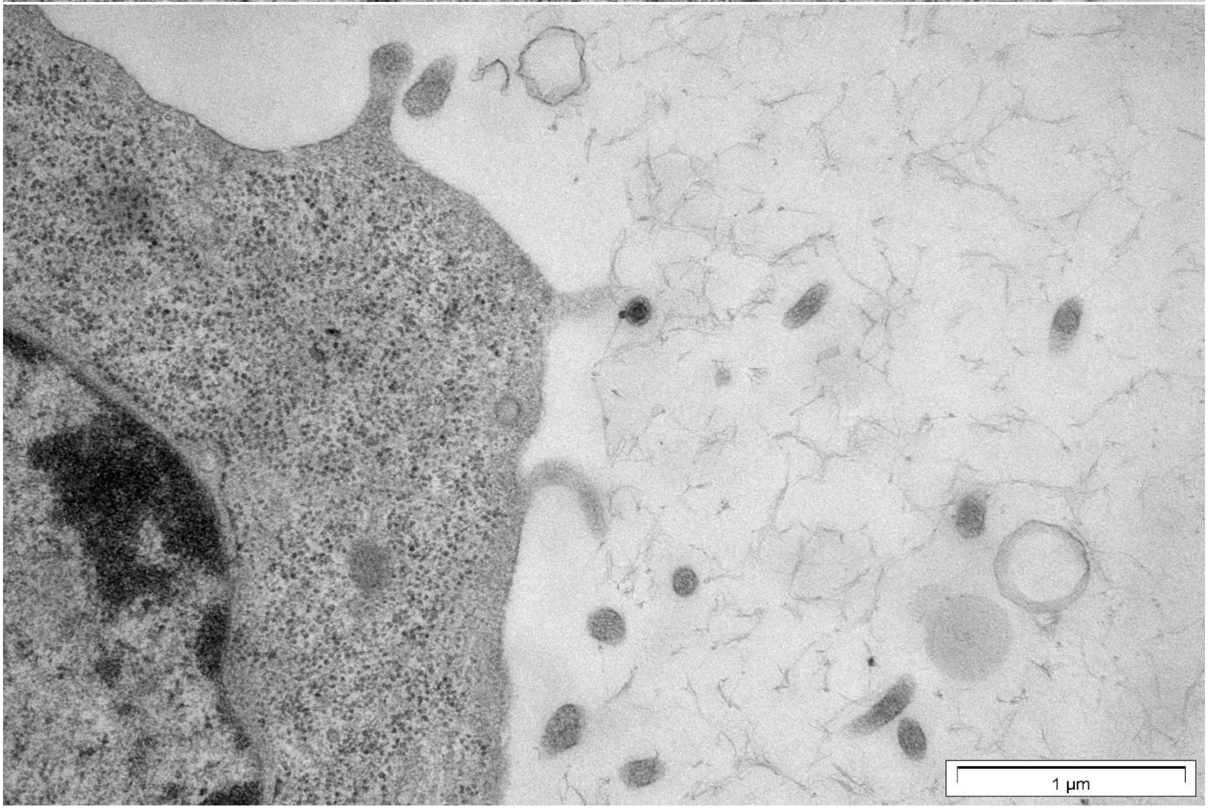
1 µm

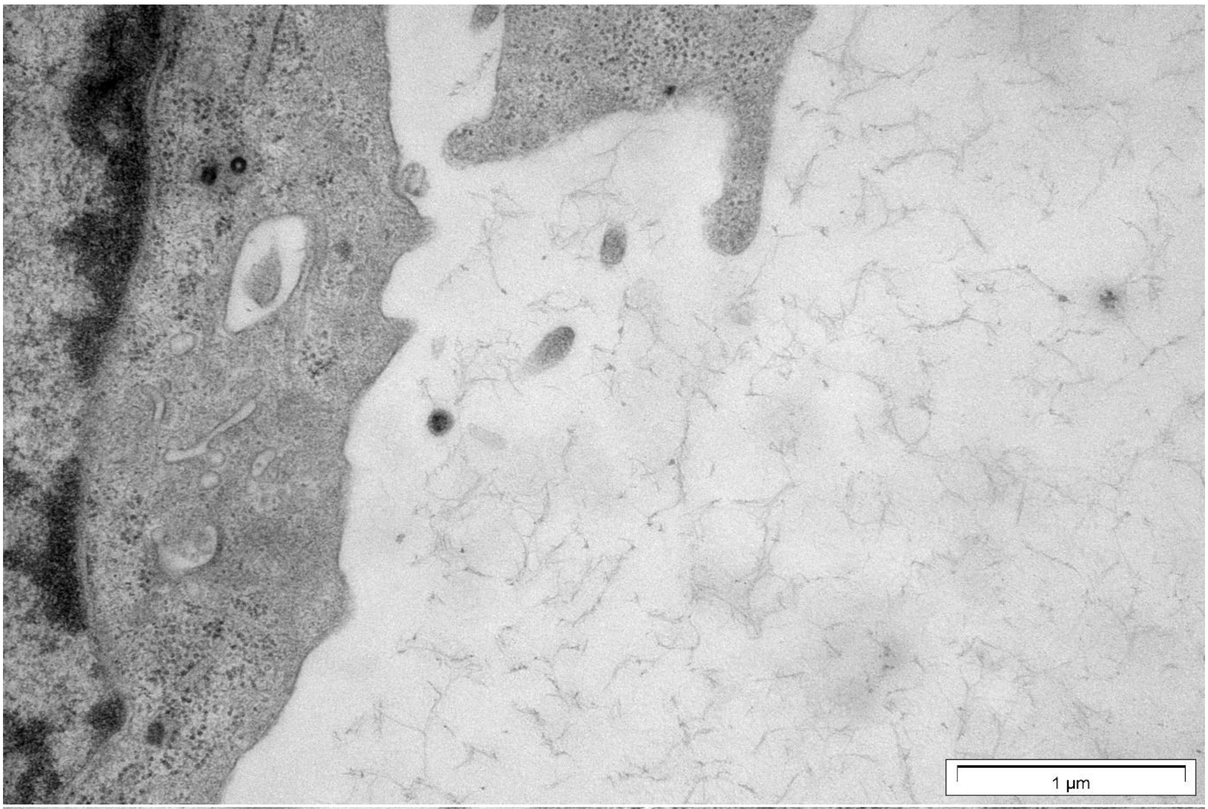
1 µm

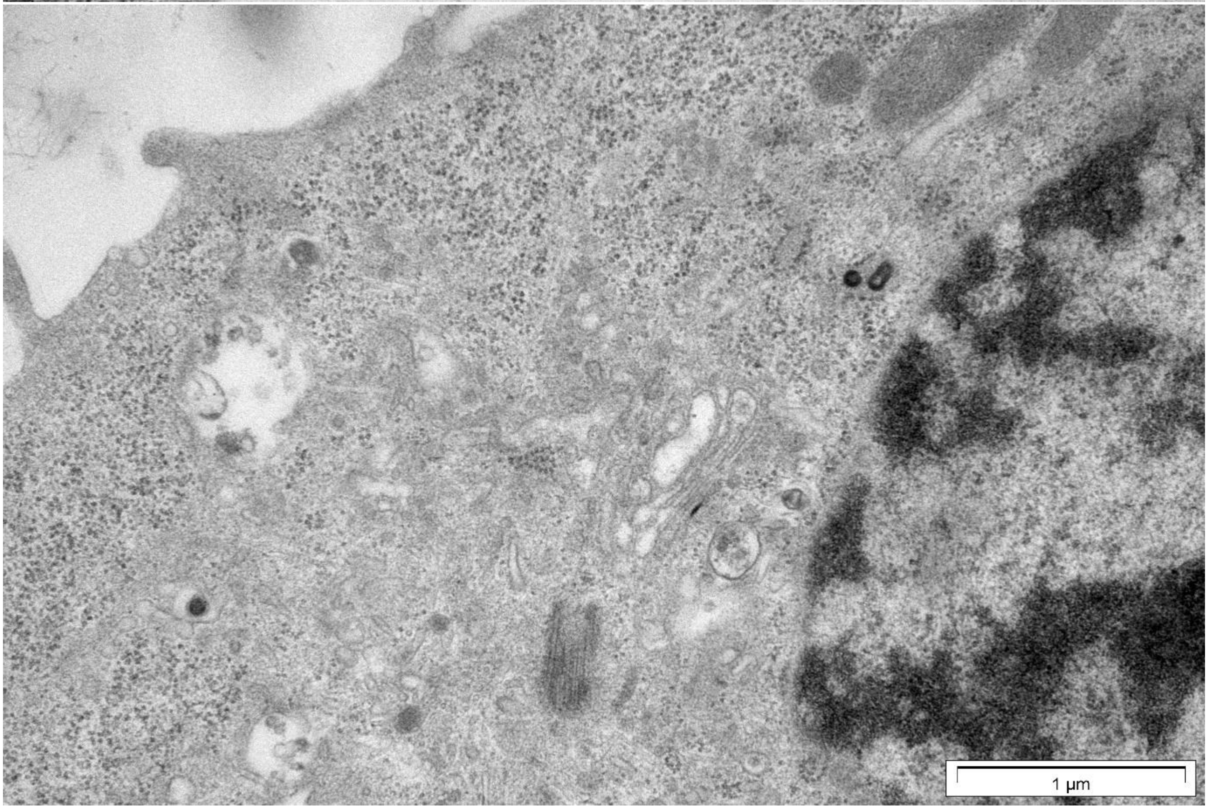
1 µm

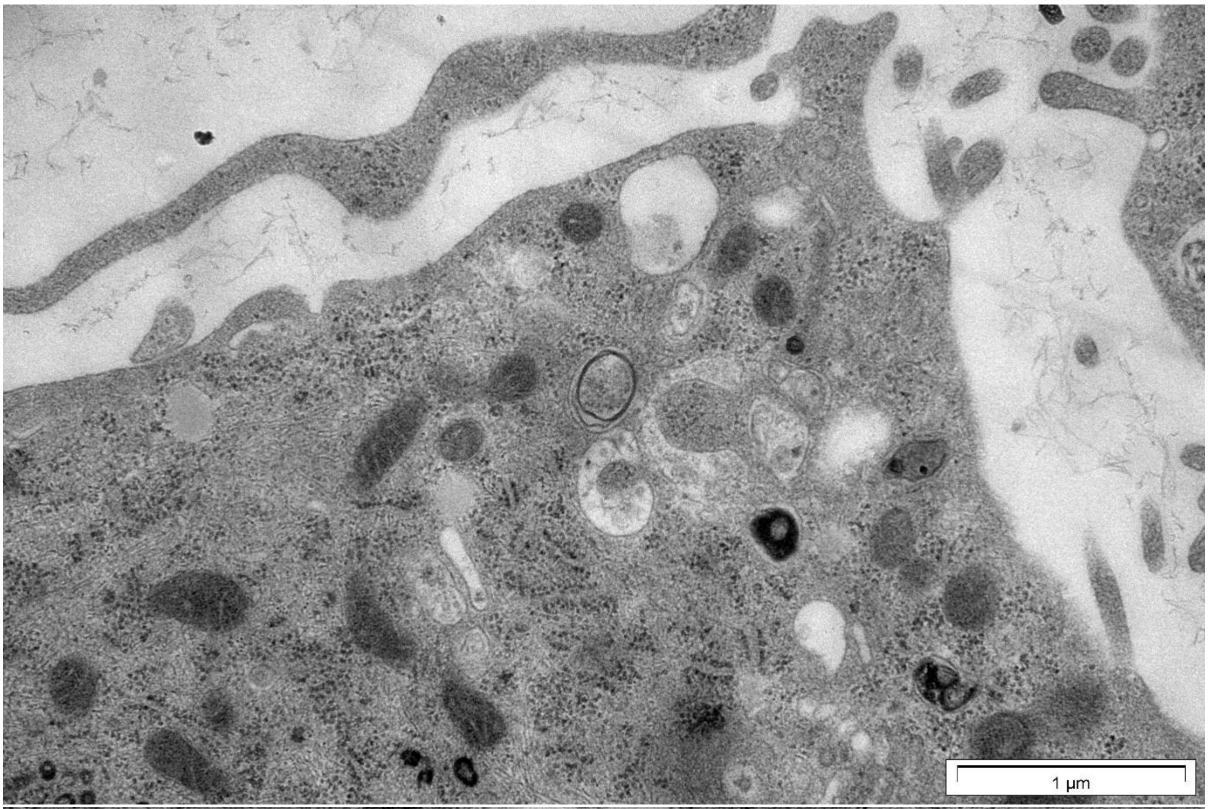
1 µm

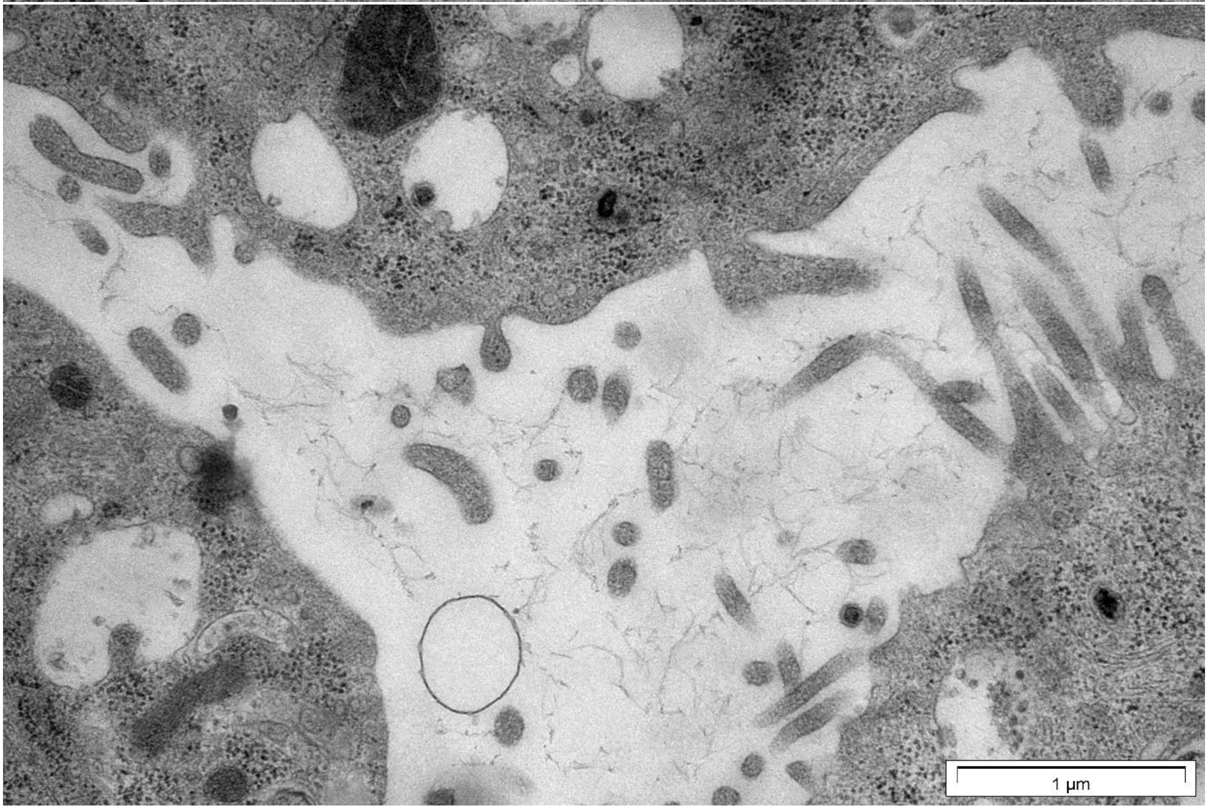
1 µm

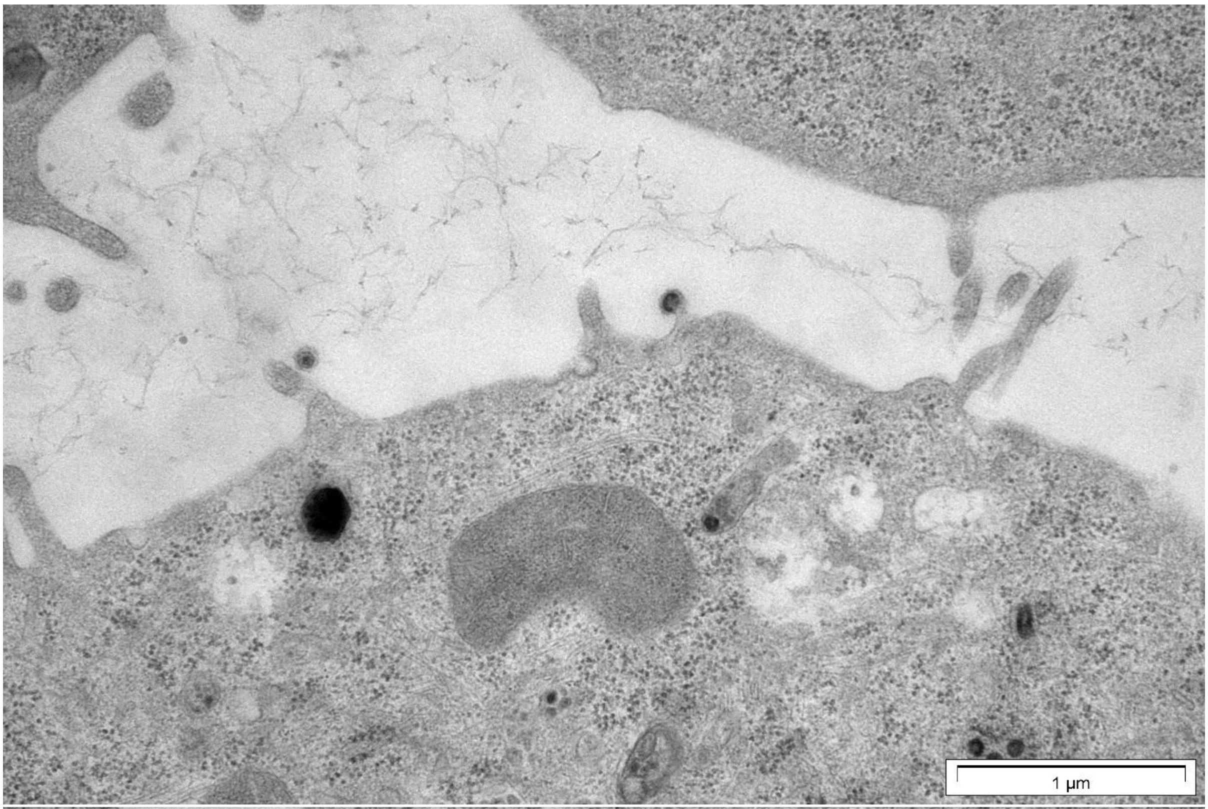
1 µm

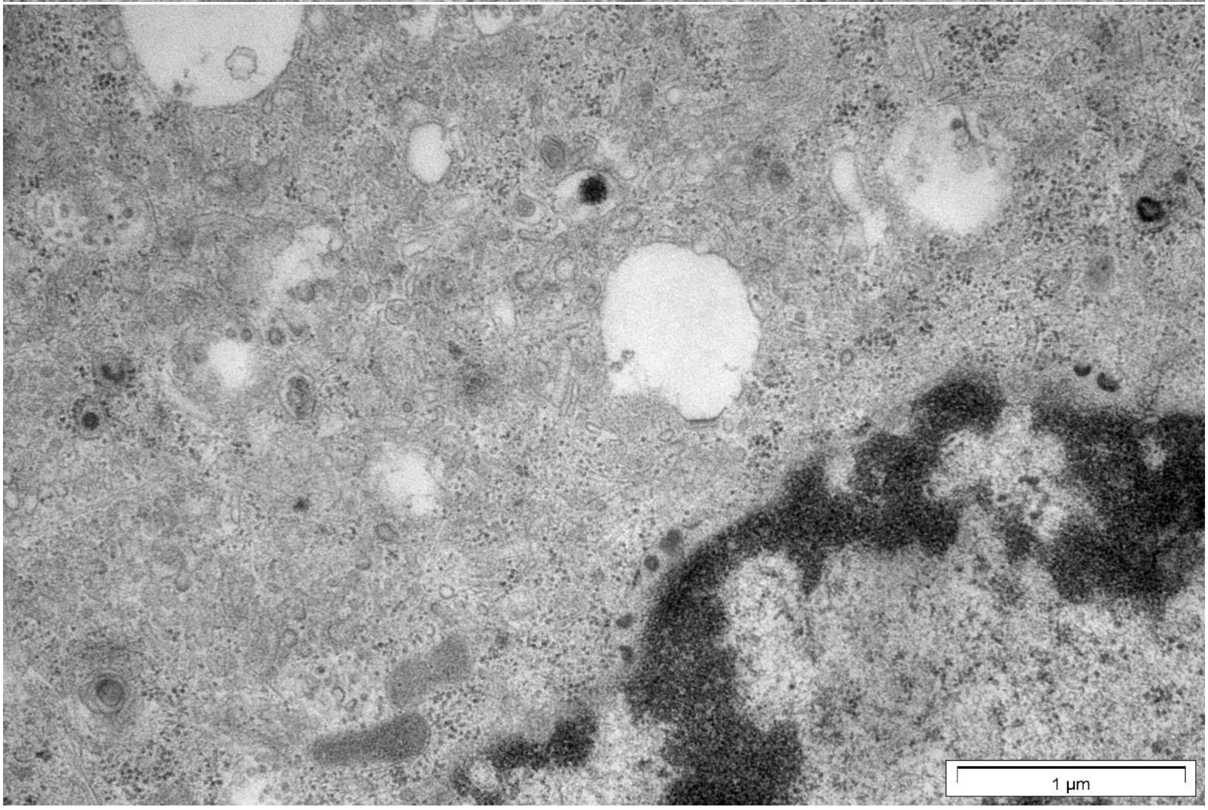
1 µm

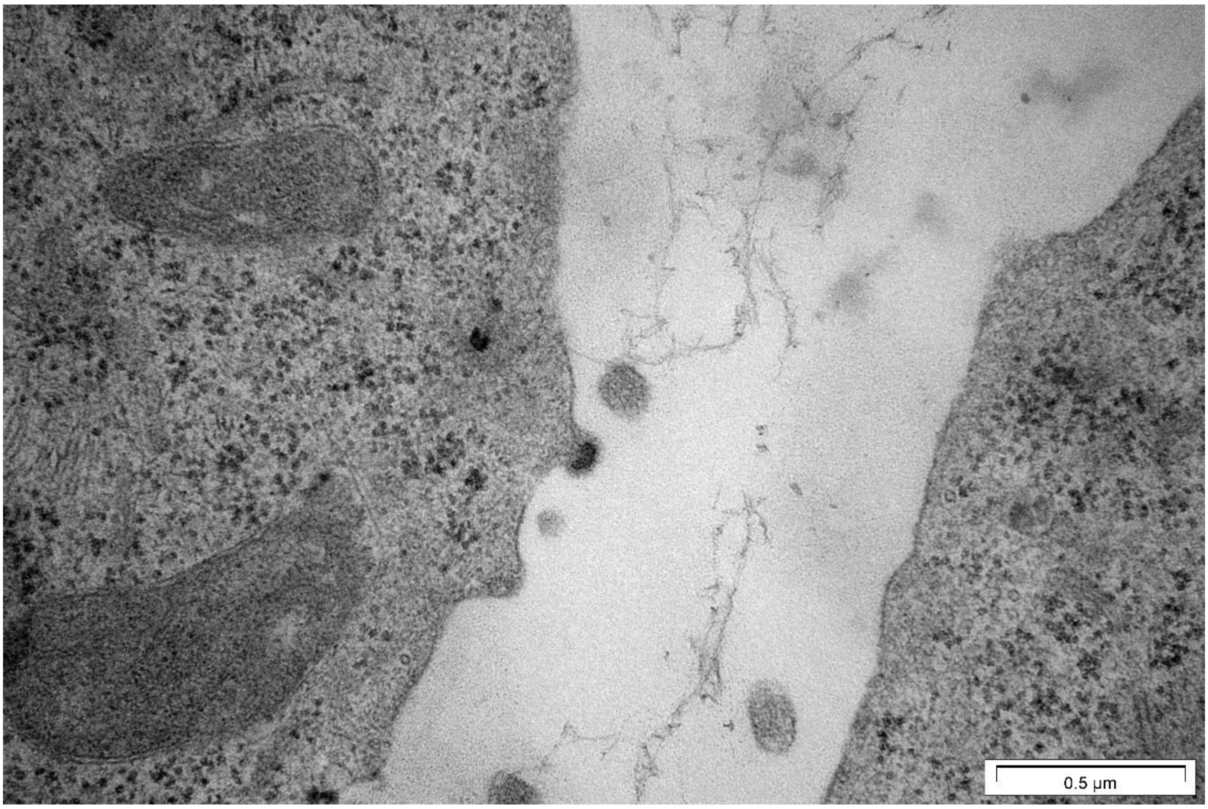
0.5 µm

Dextran, 5 min.

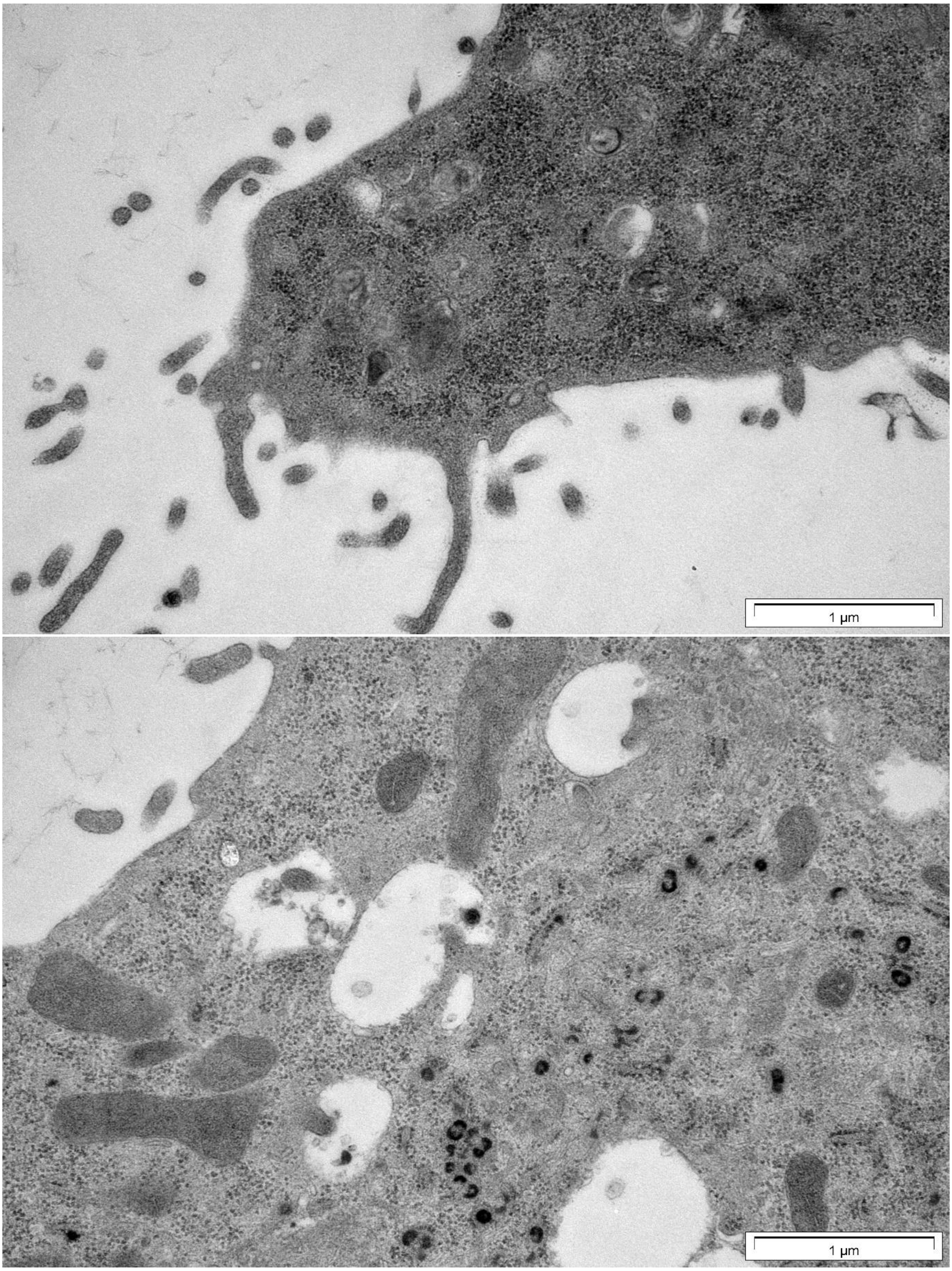

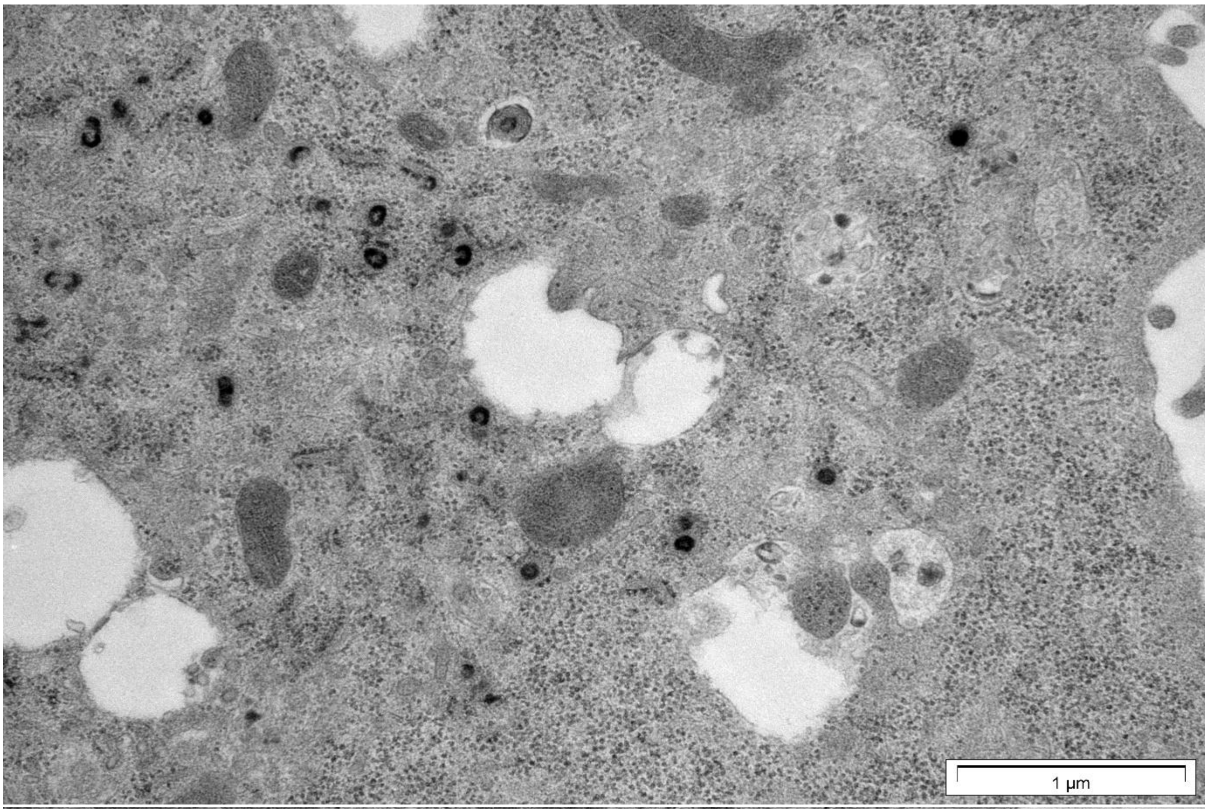
1 µm

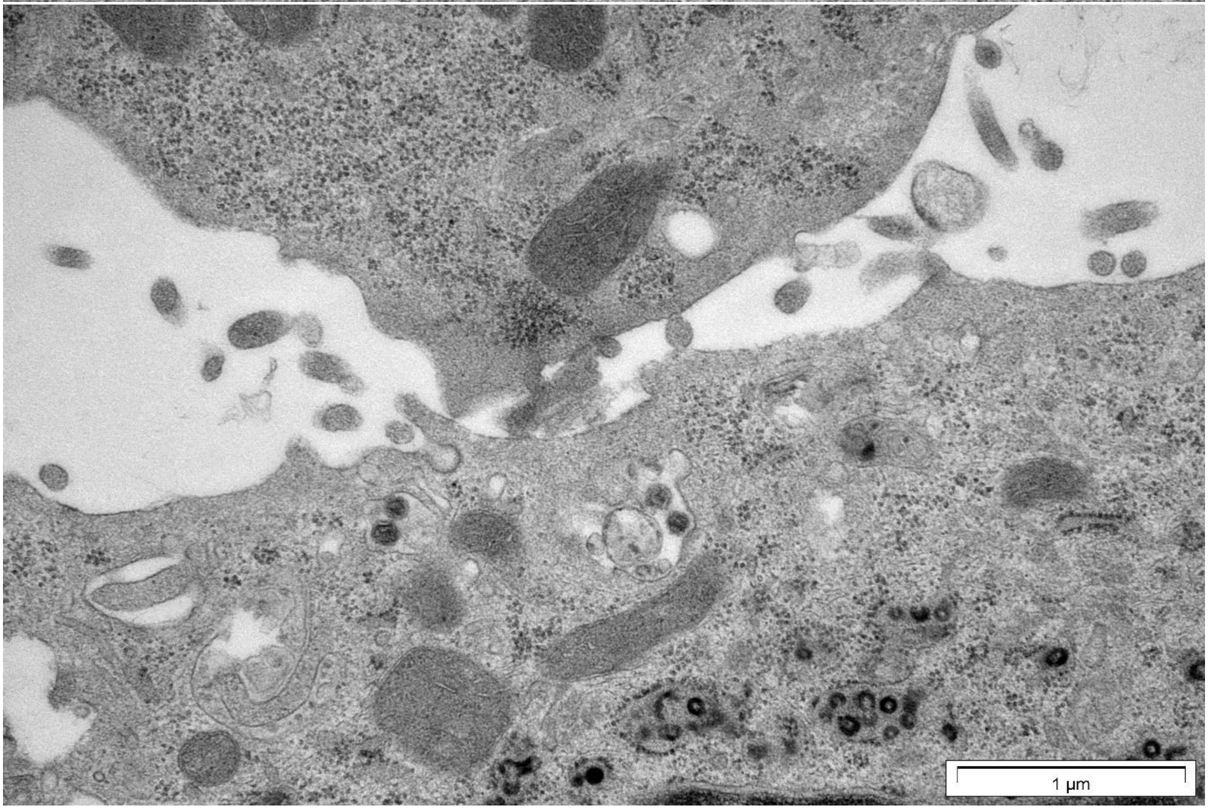
1 µm

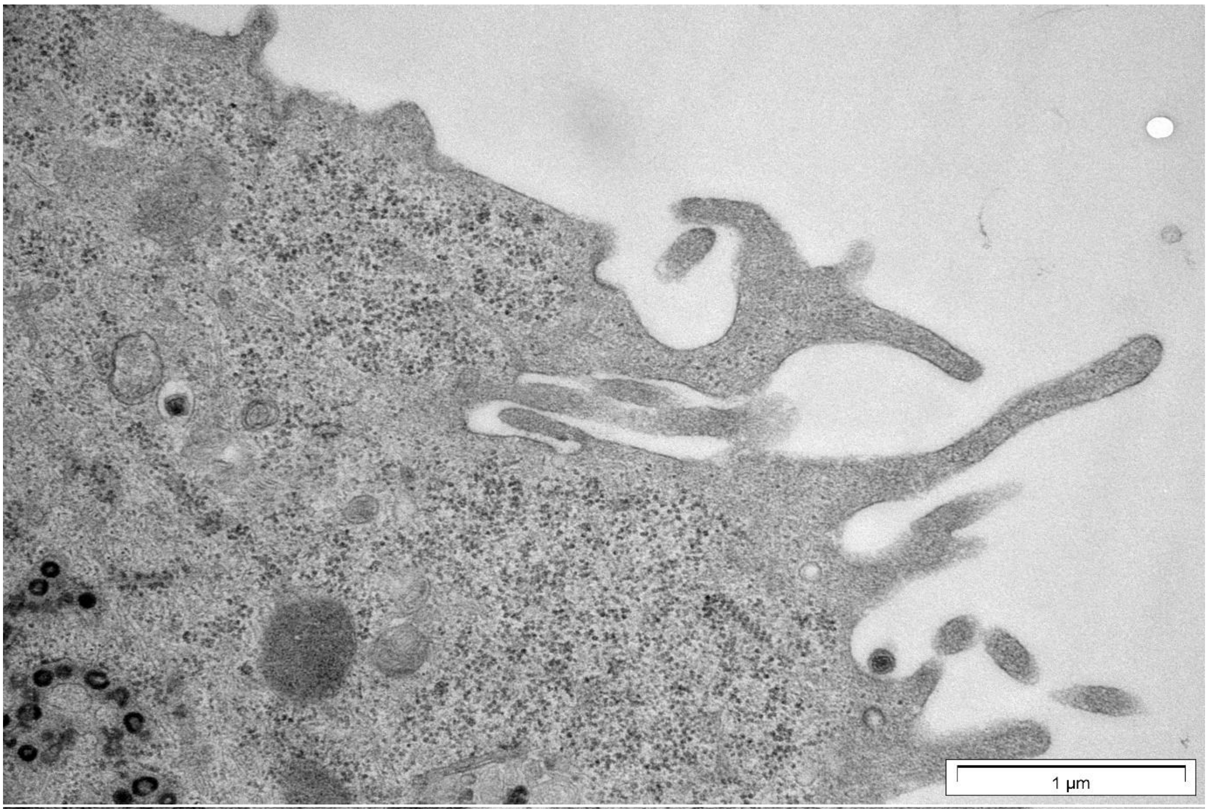

1 µm

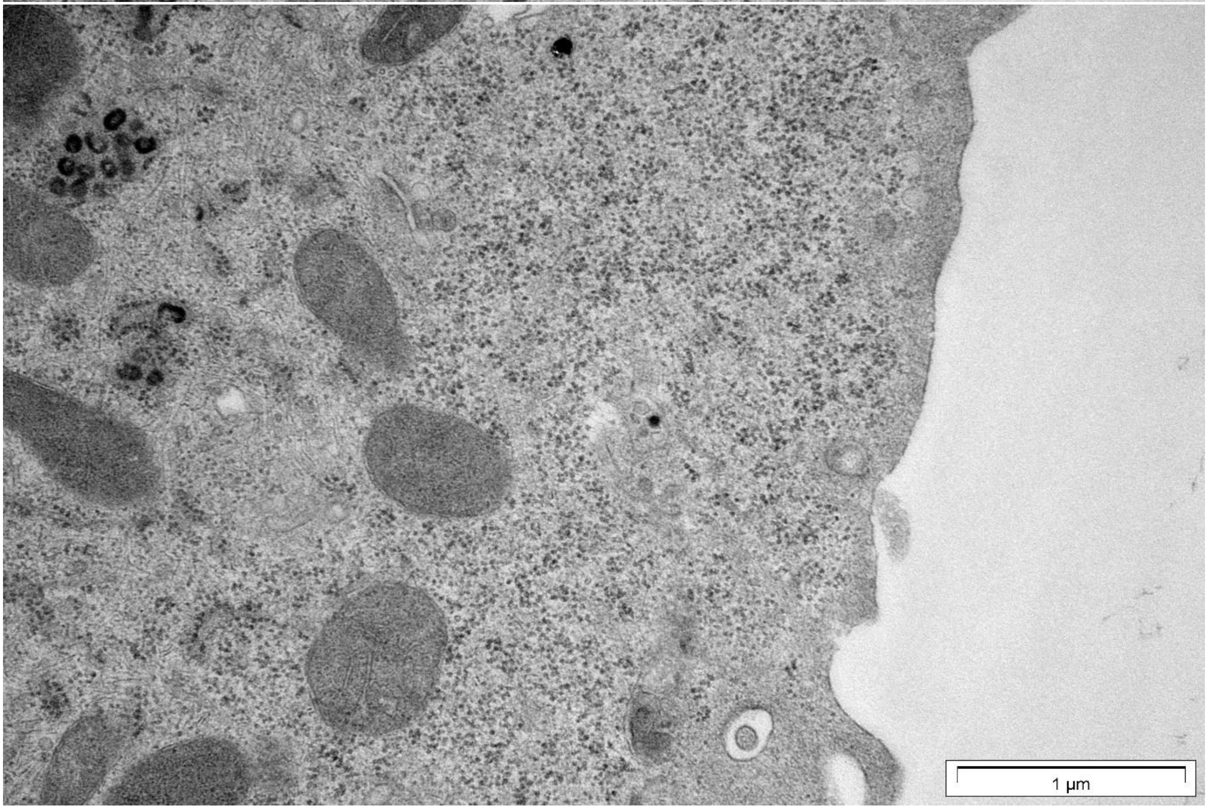

1 µm

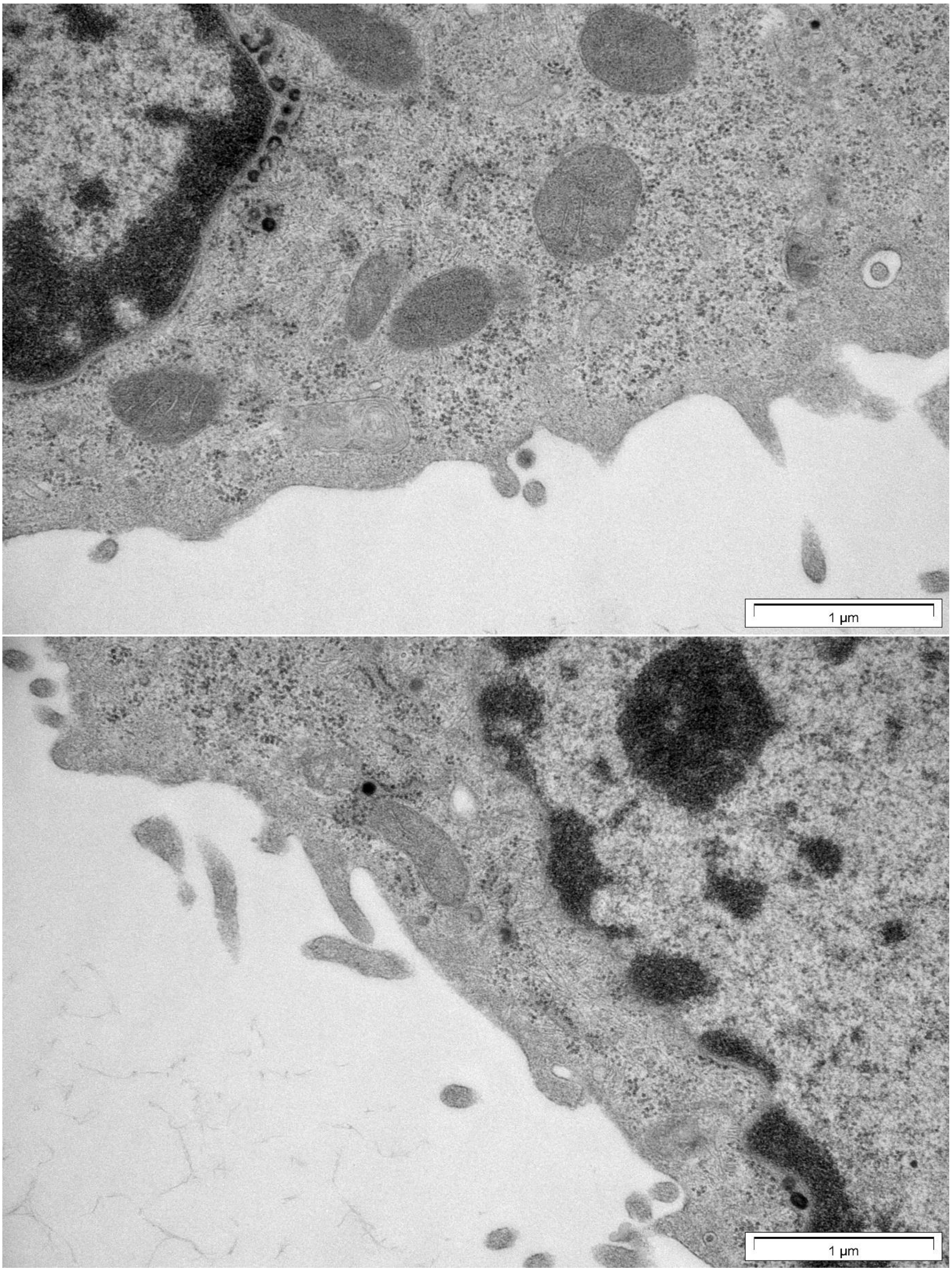

1 µm
1 µm

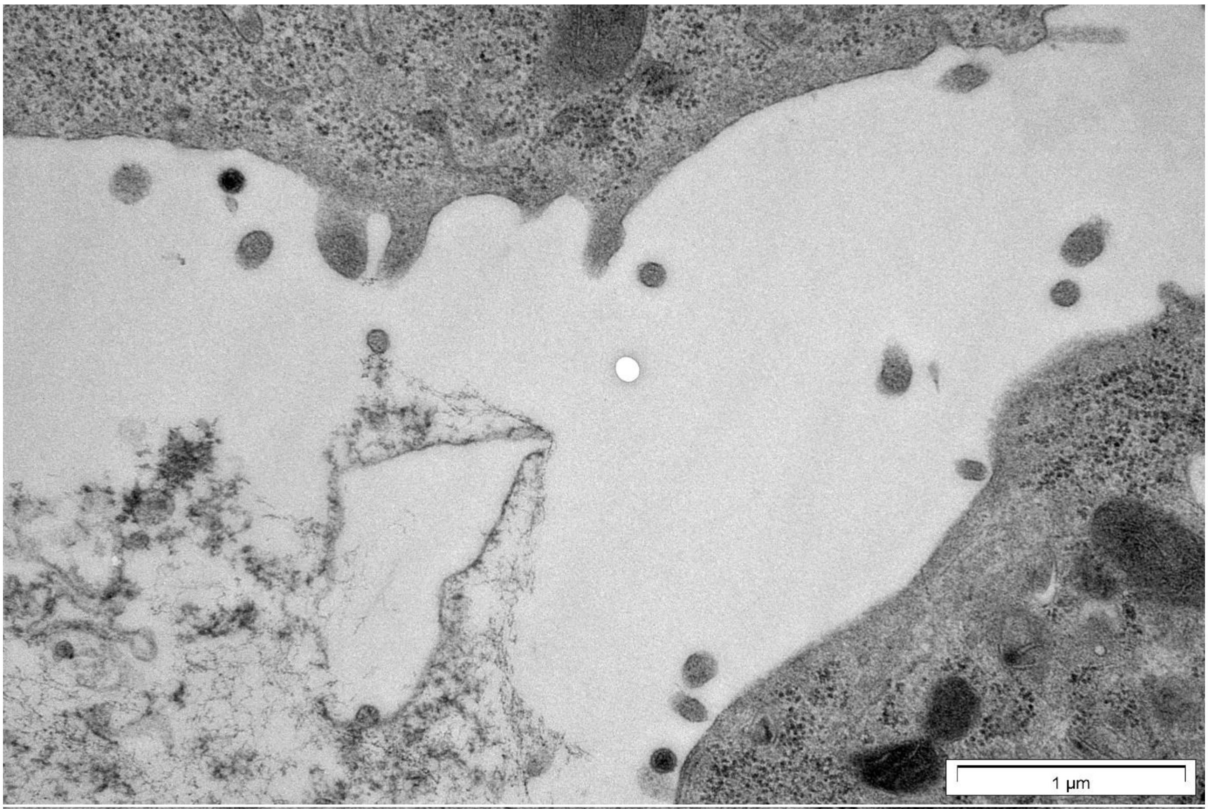
1 µm

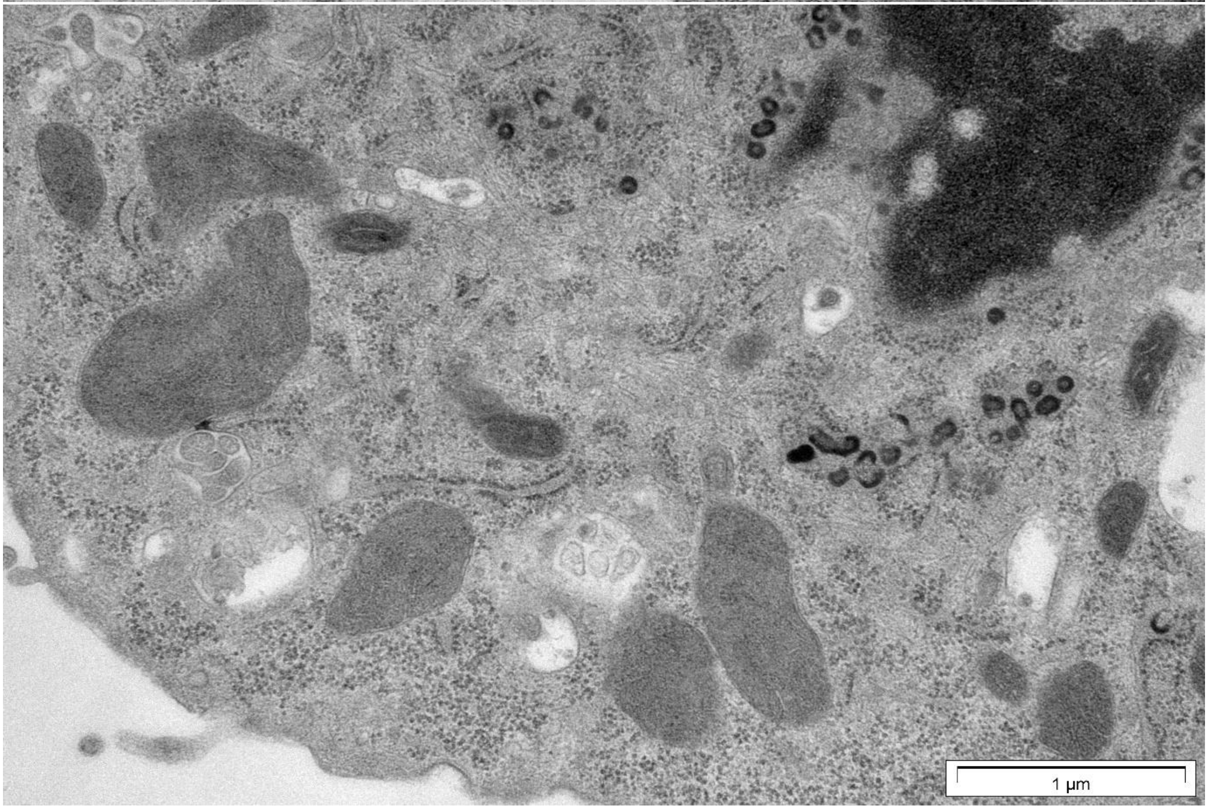
1 µm

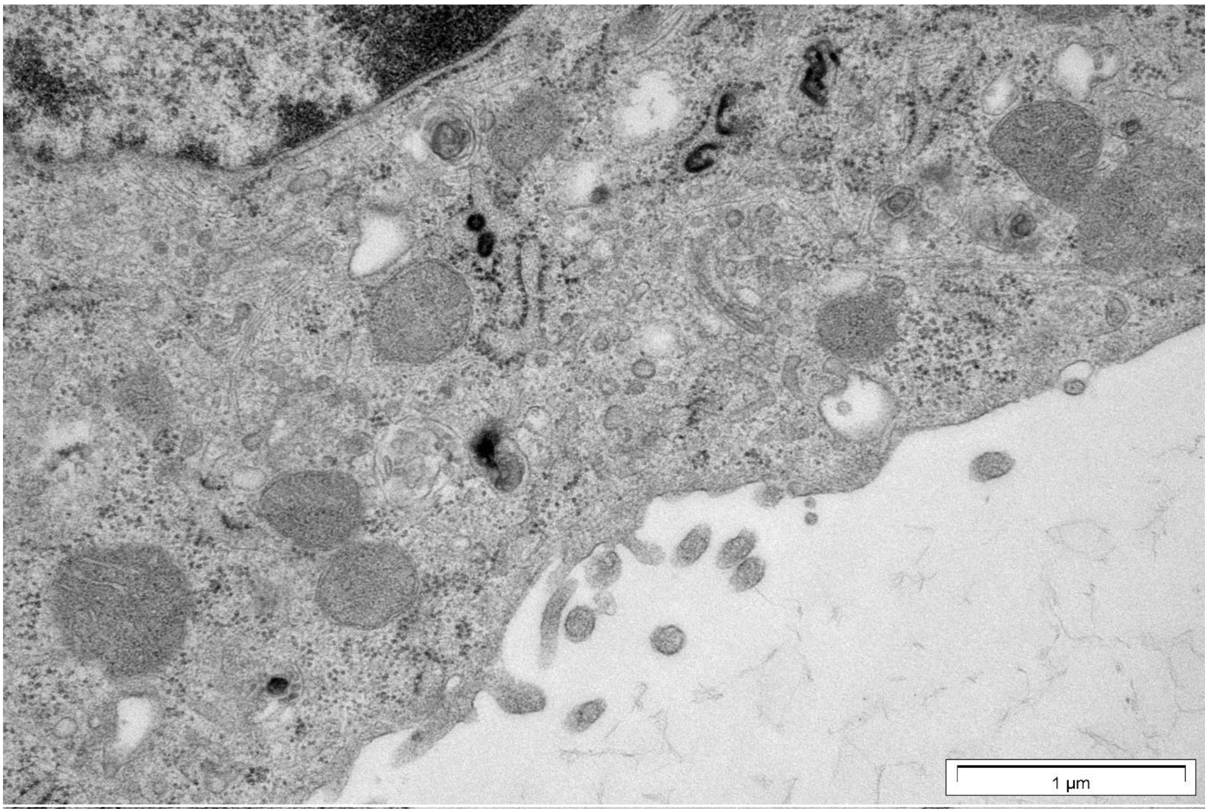
1 µm

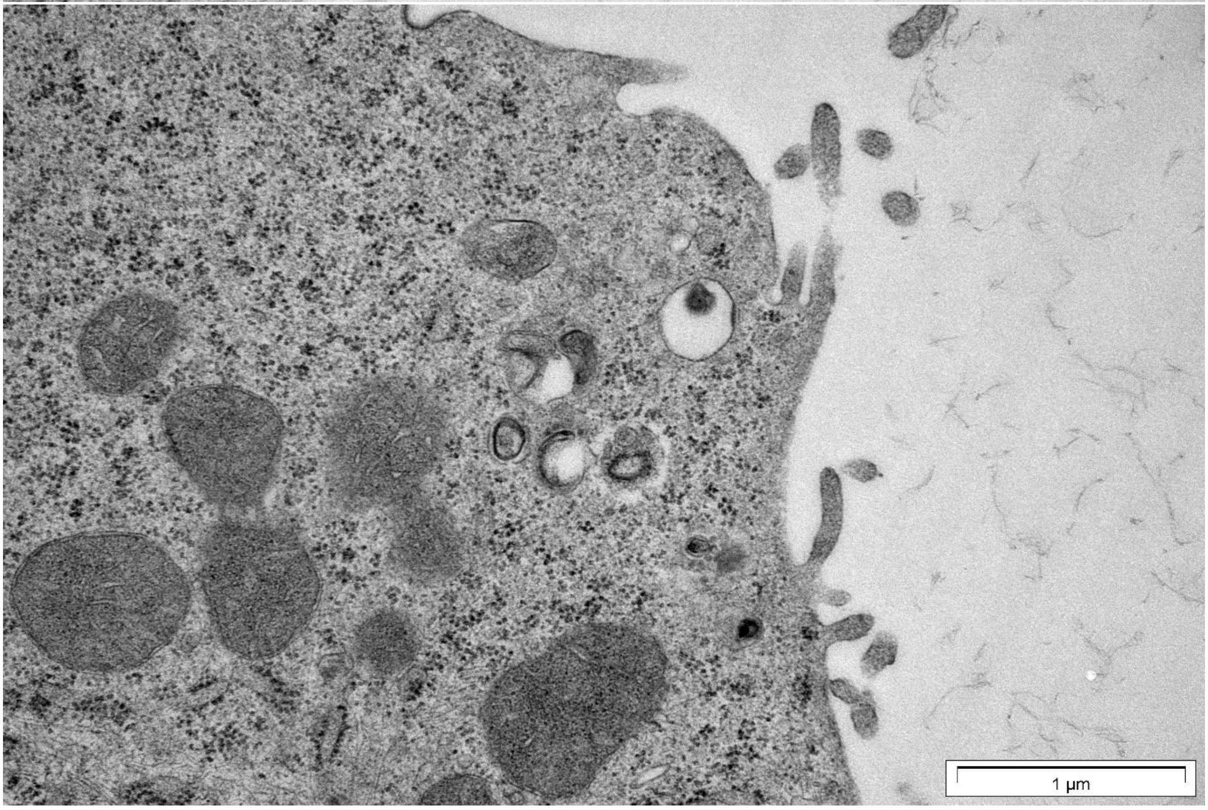
1 µm

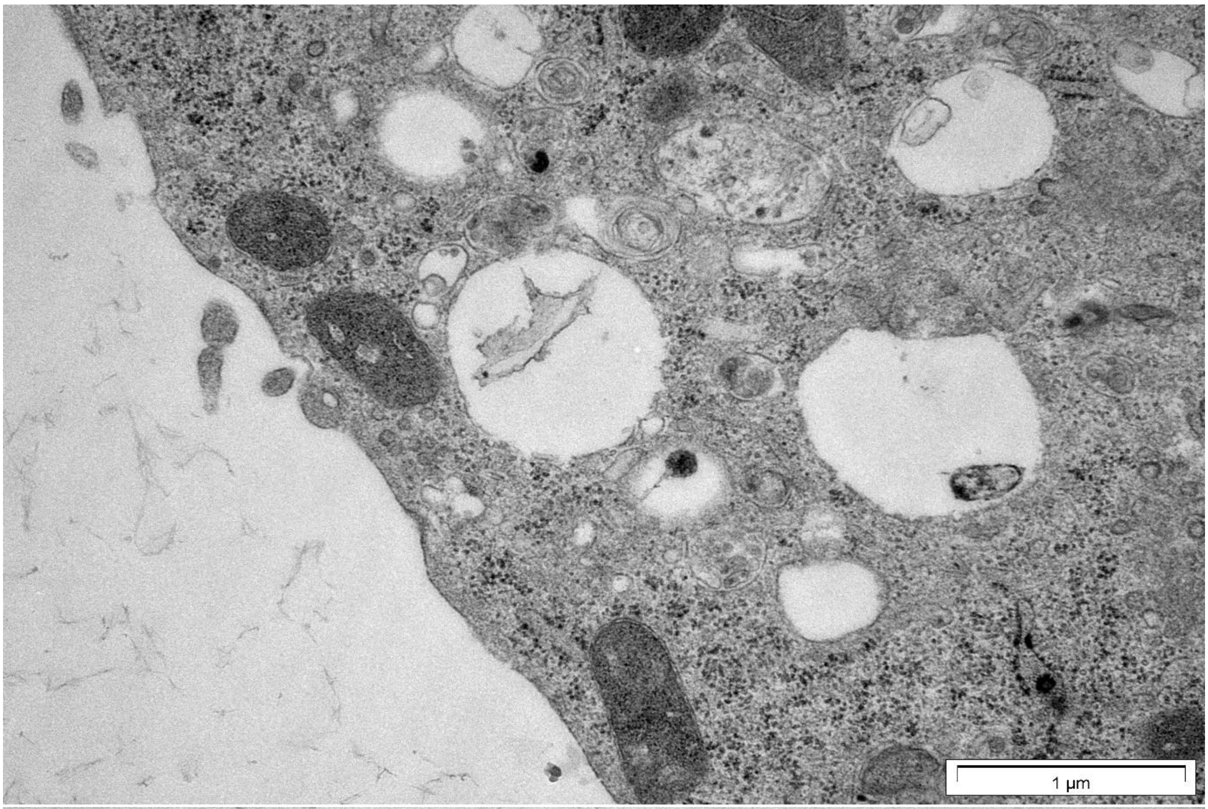
1 µm

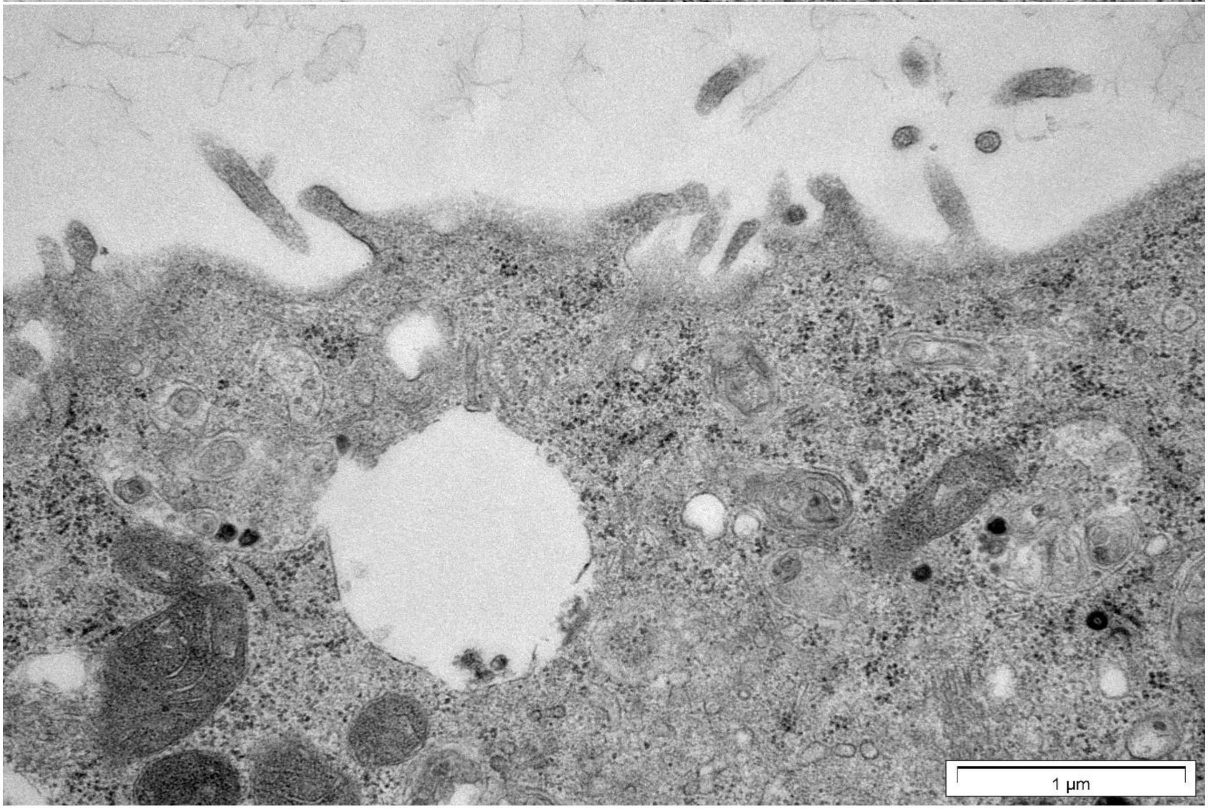
1 µm

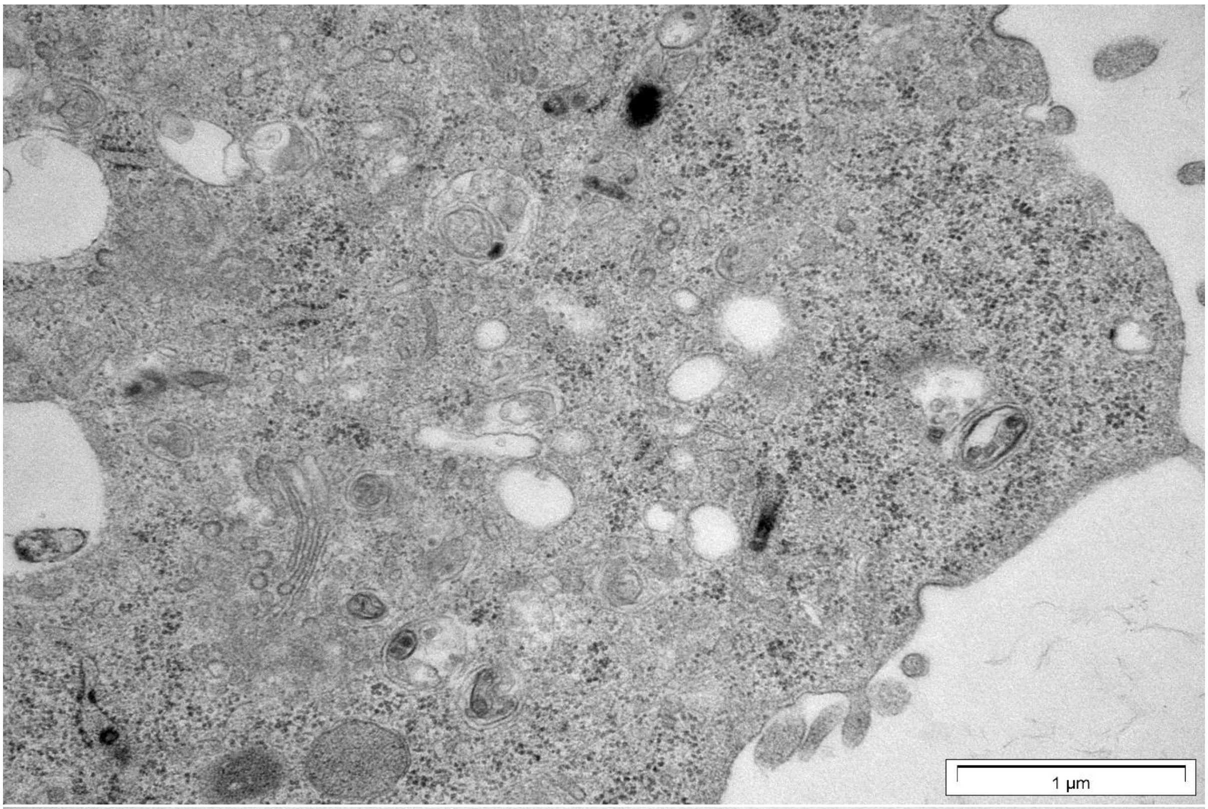
1 µm

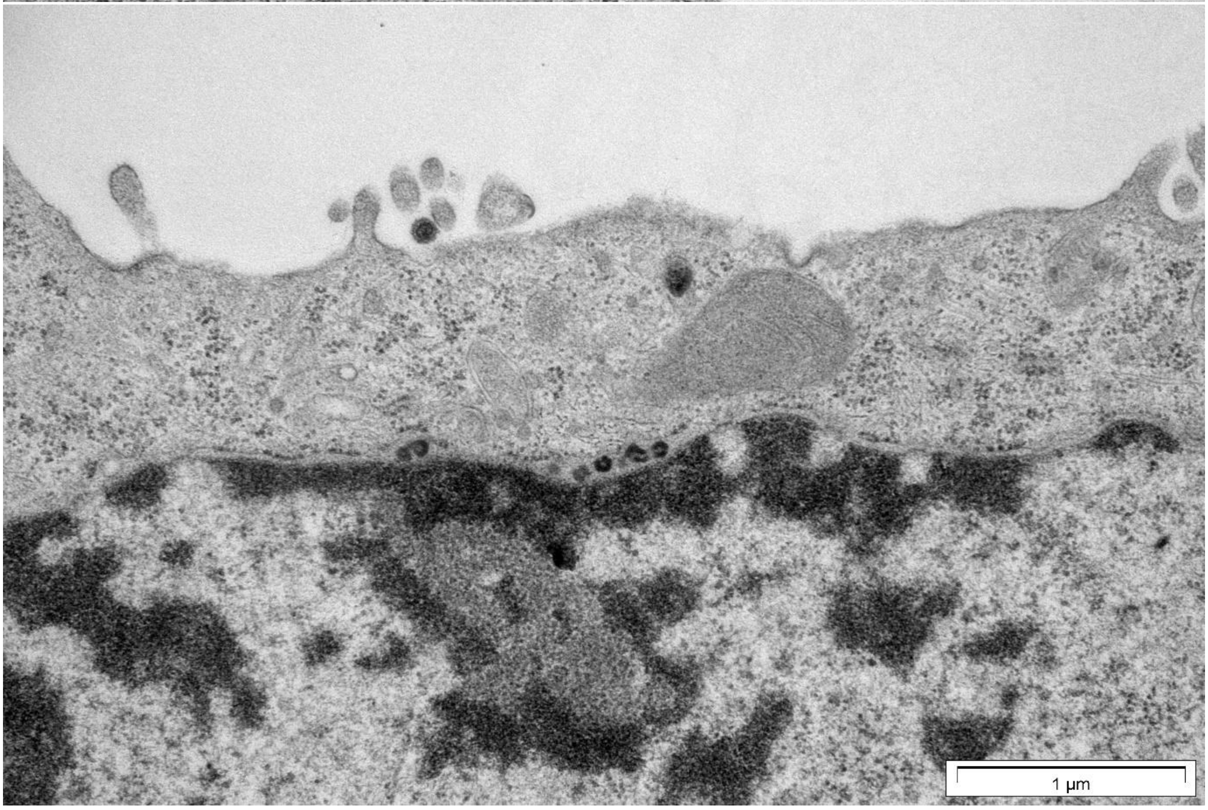
1 µm

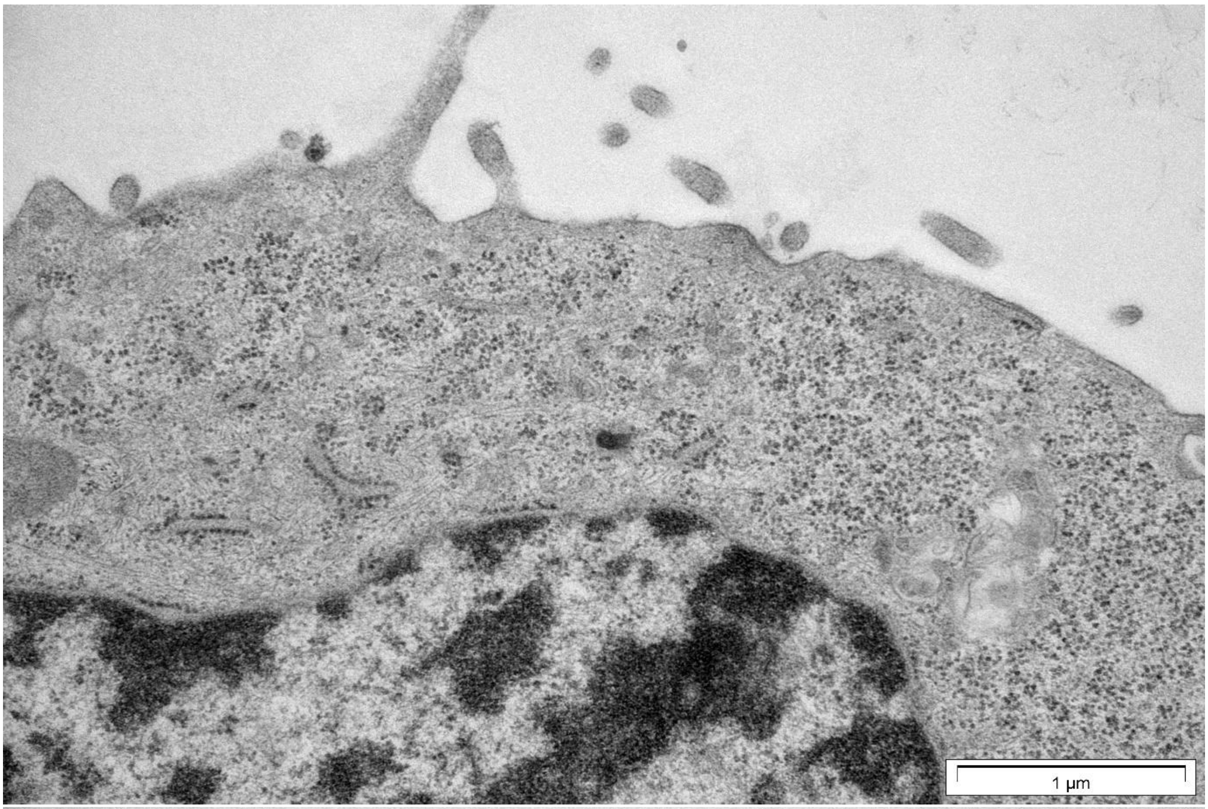
1 µm

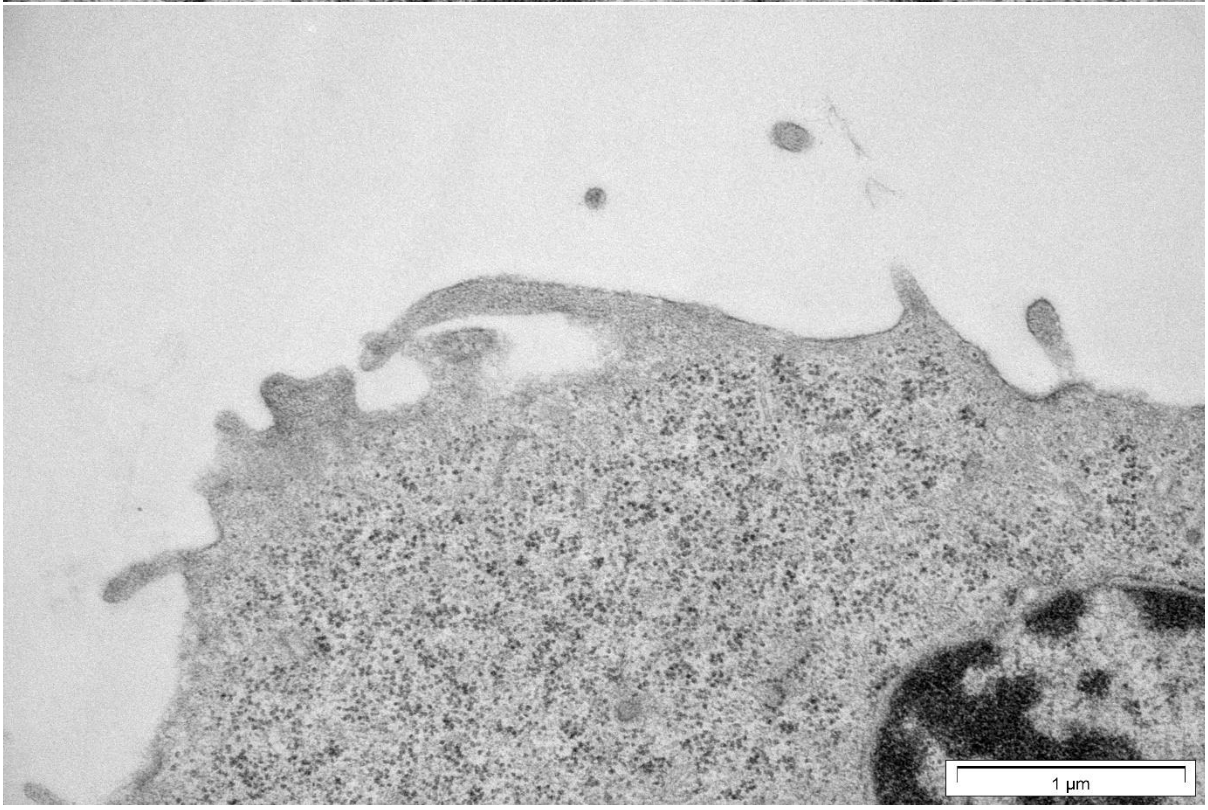
1 µm

Dextran, 15 min.

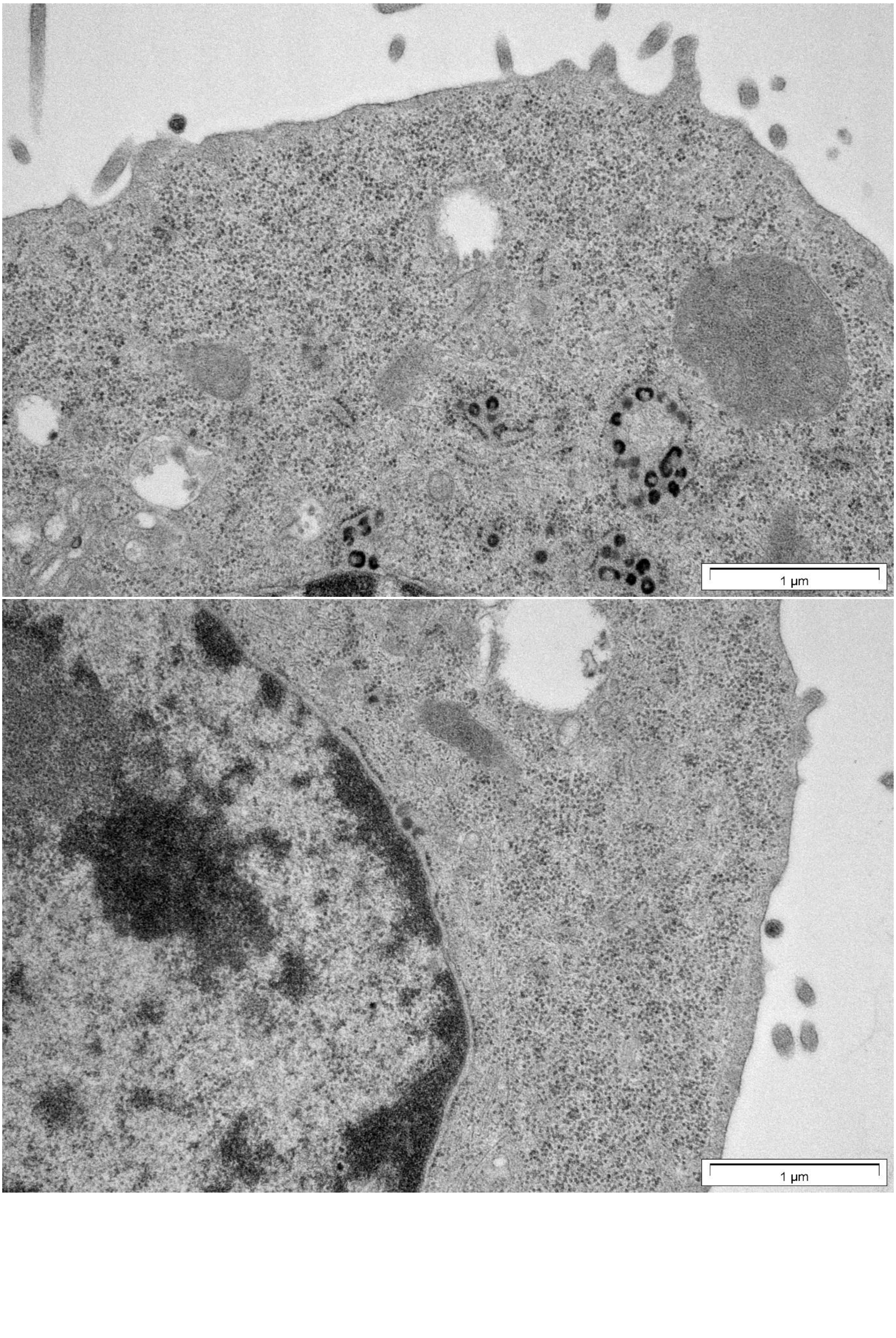

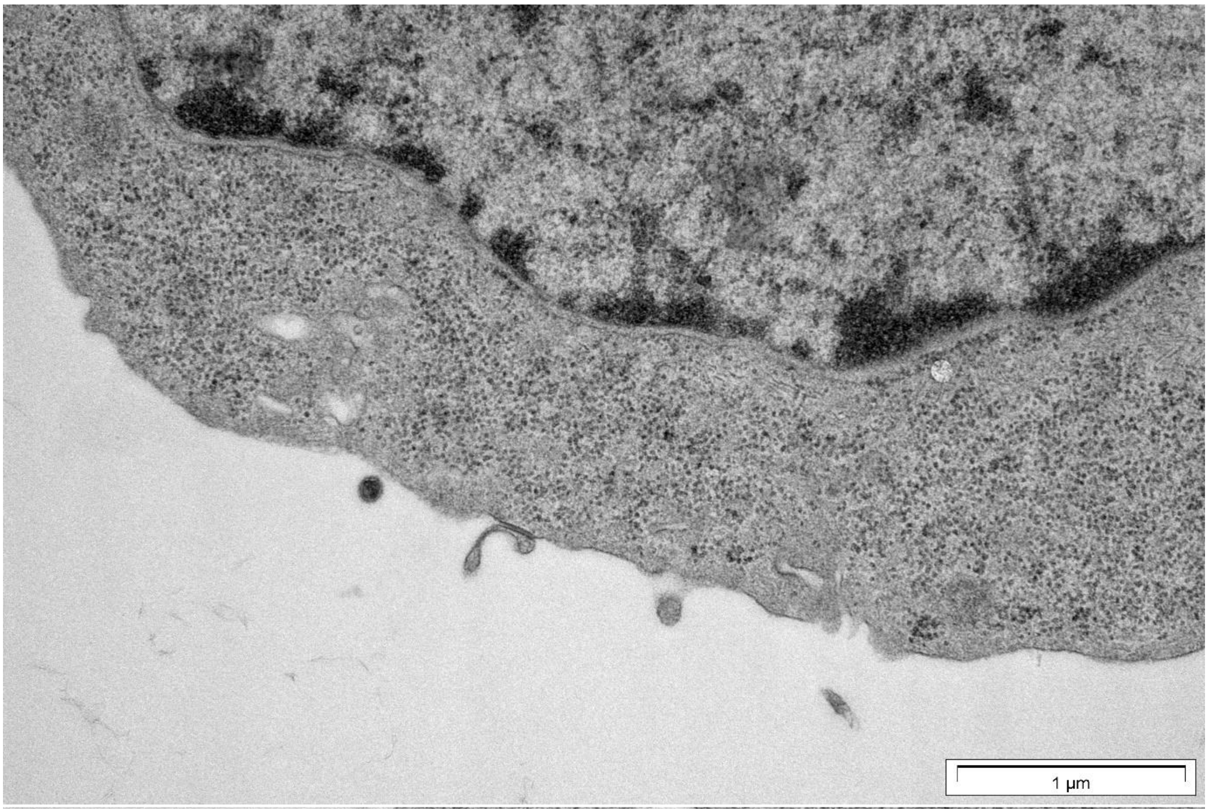
1 µm

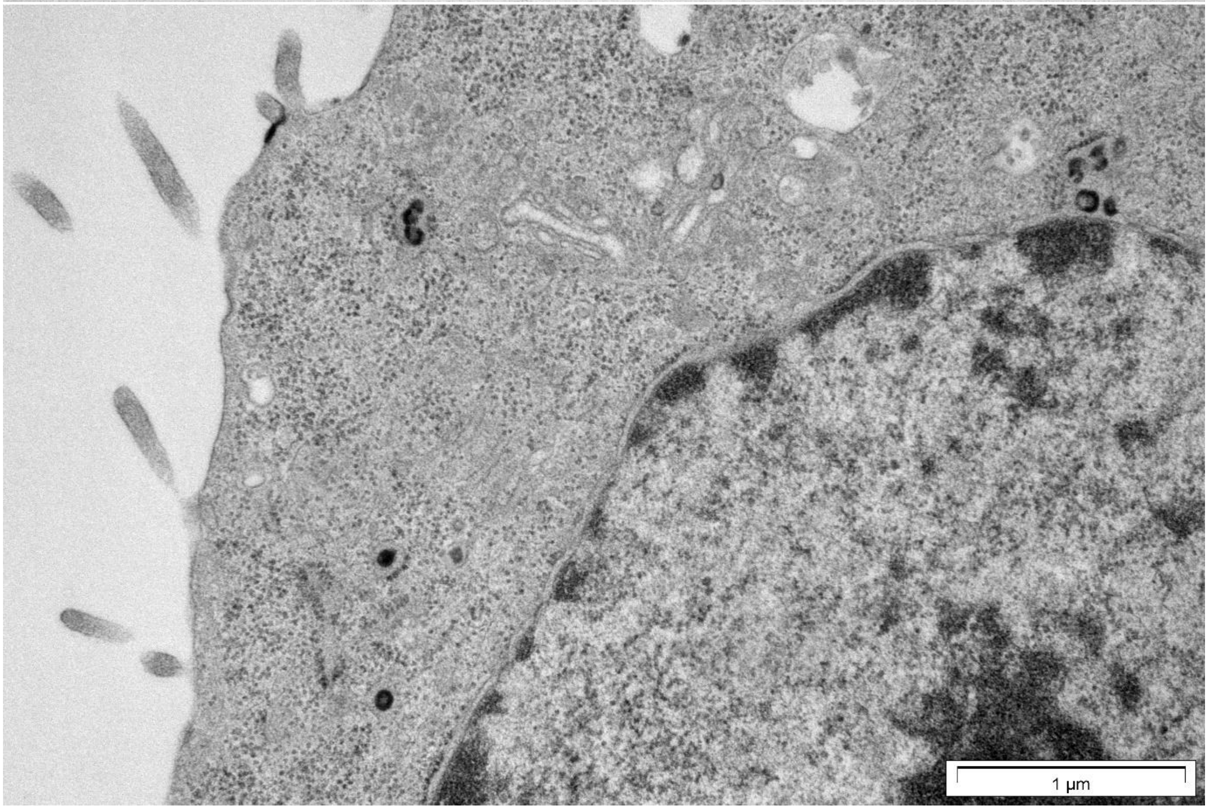
1 µm

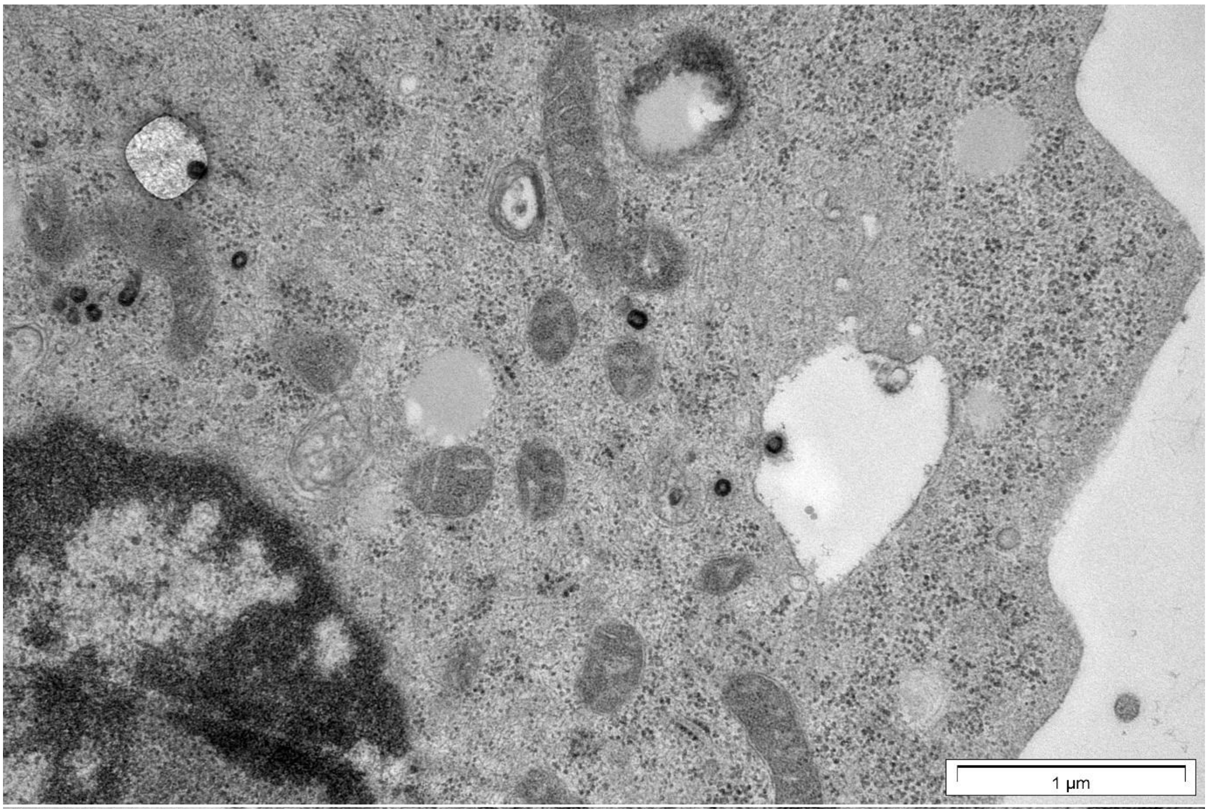
1 µm

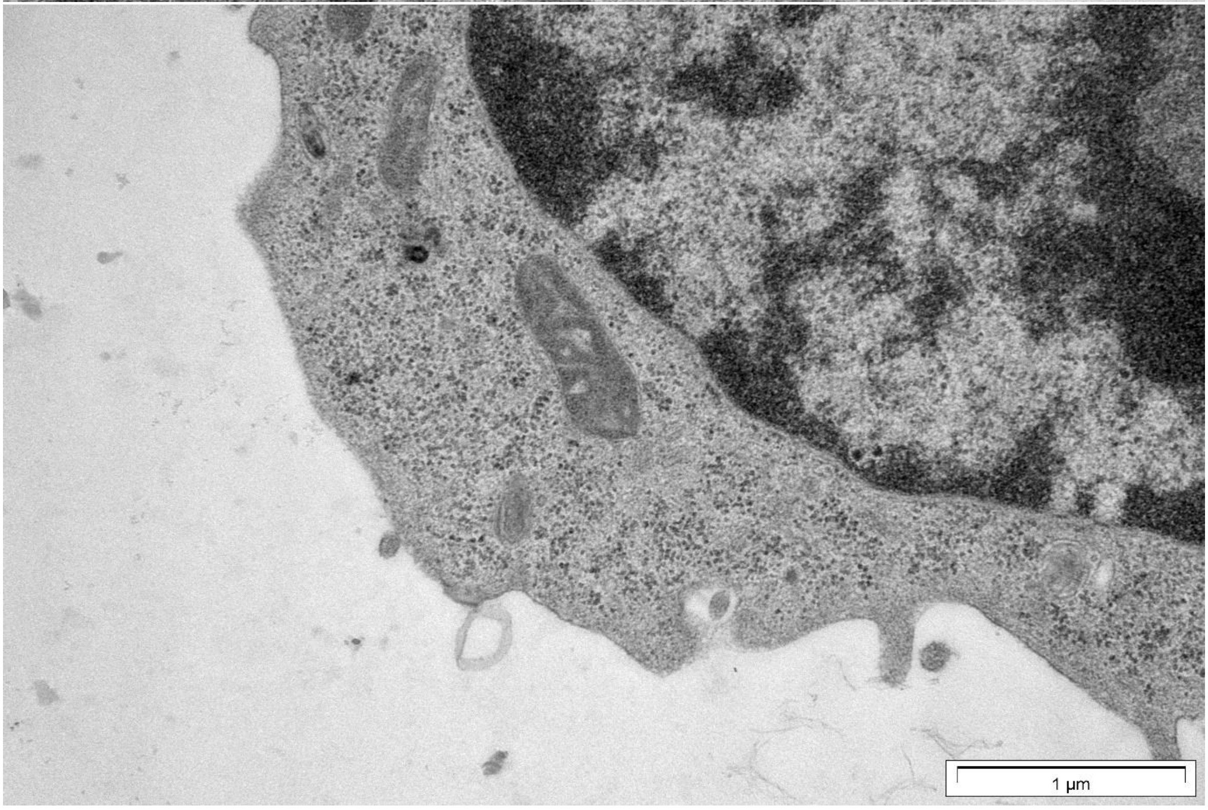
1 µm

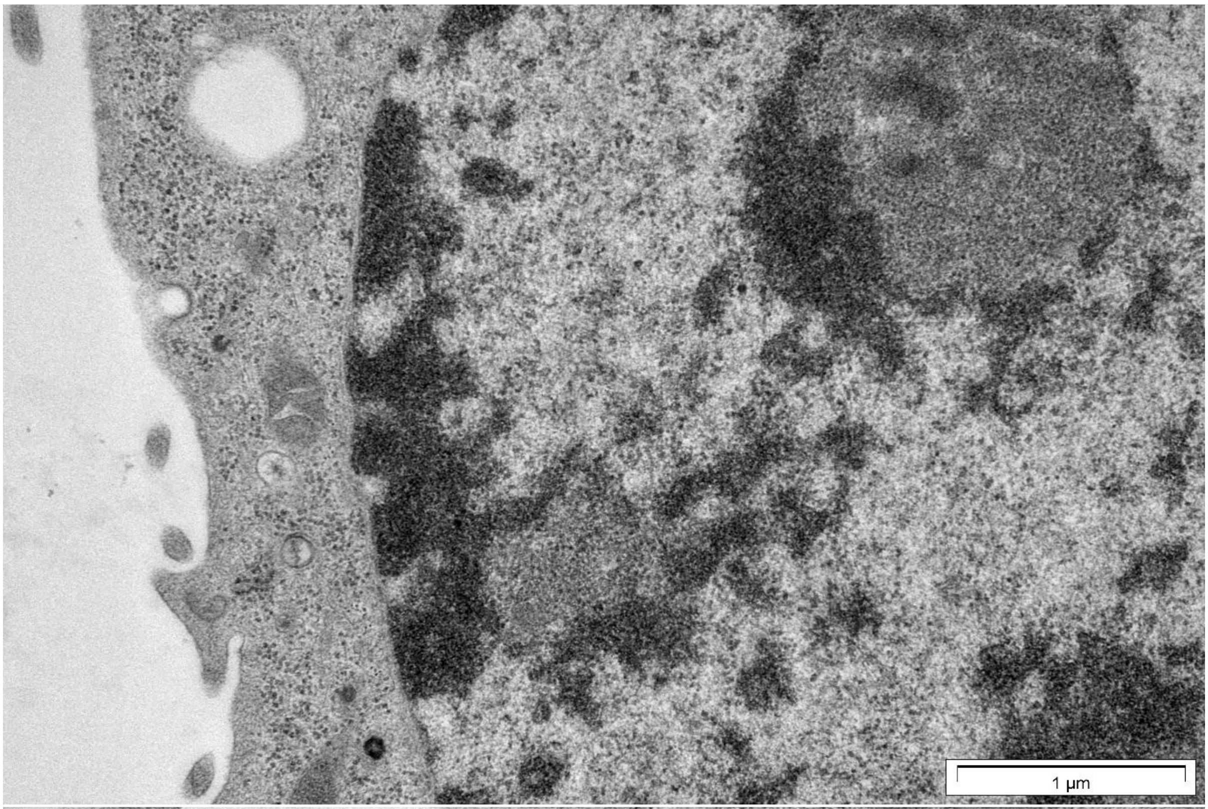
1 µm

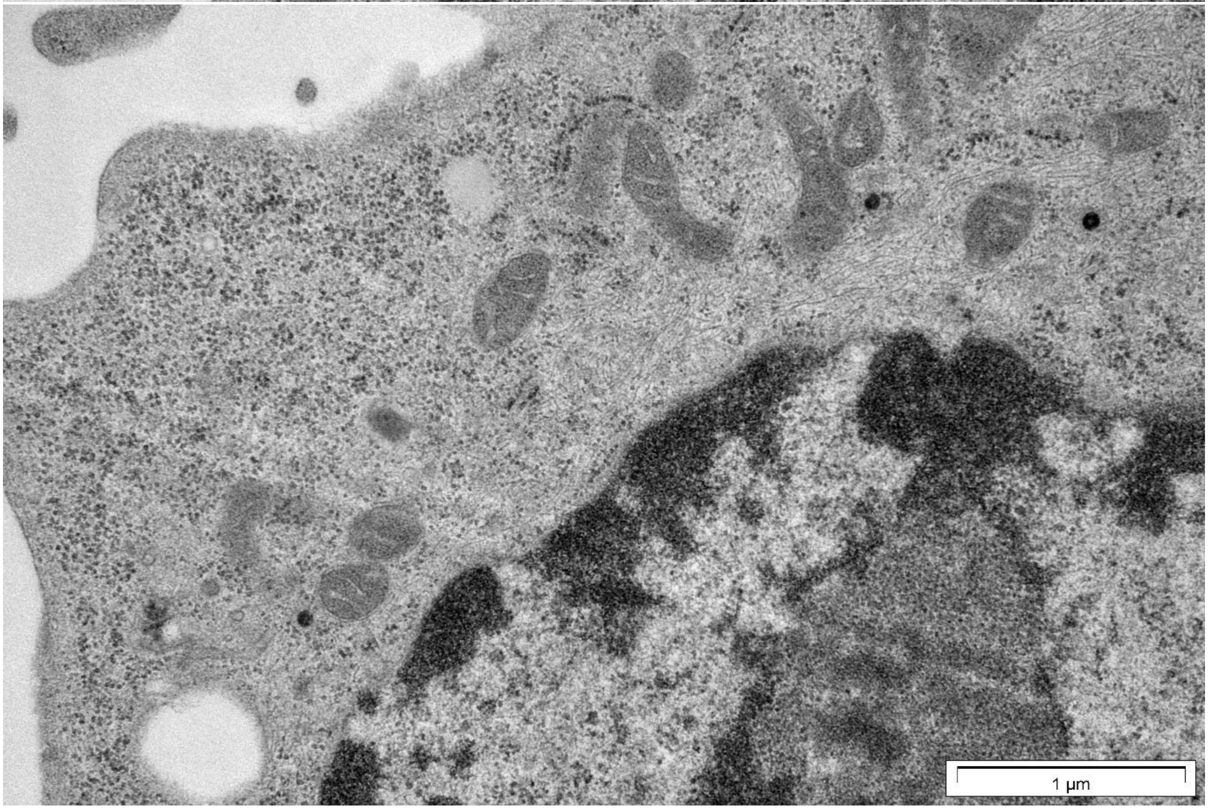
1 µm

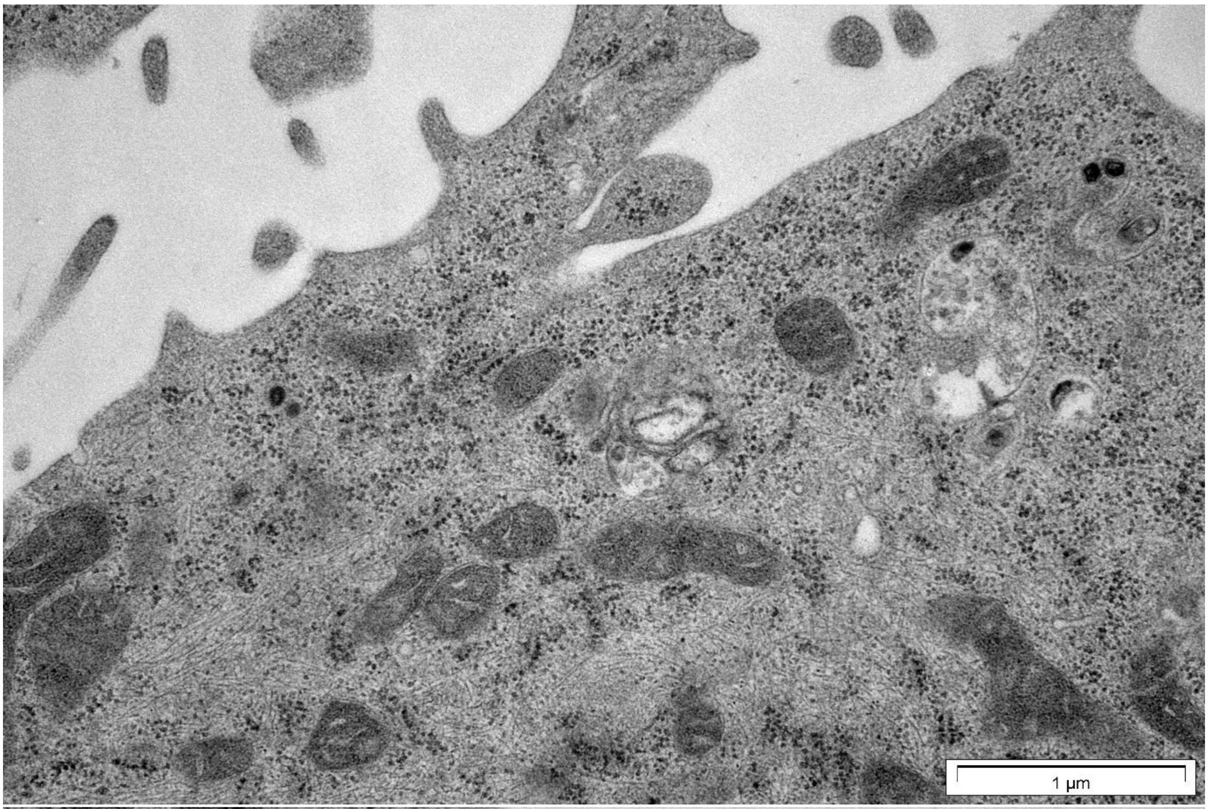
1 µm

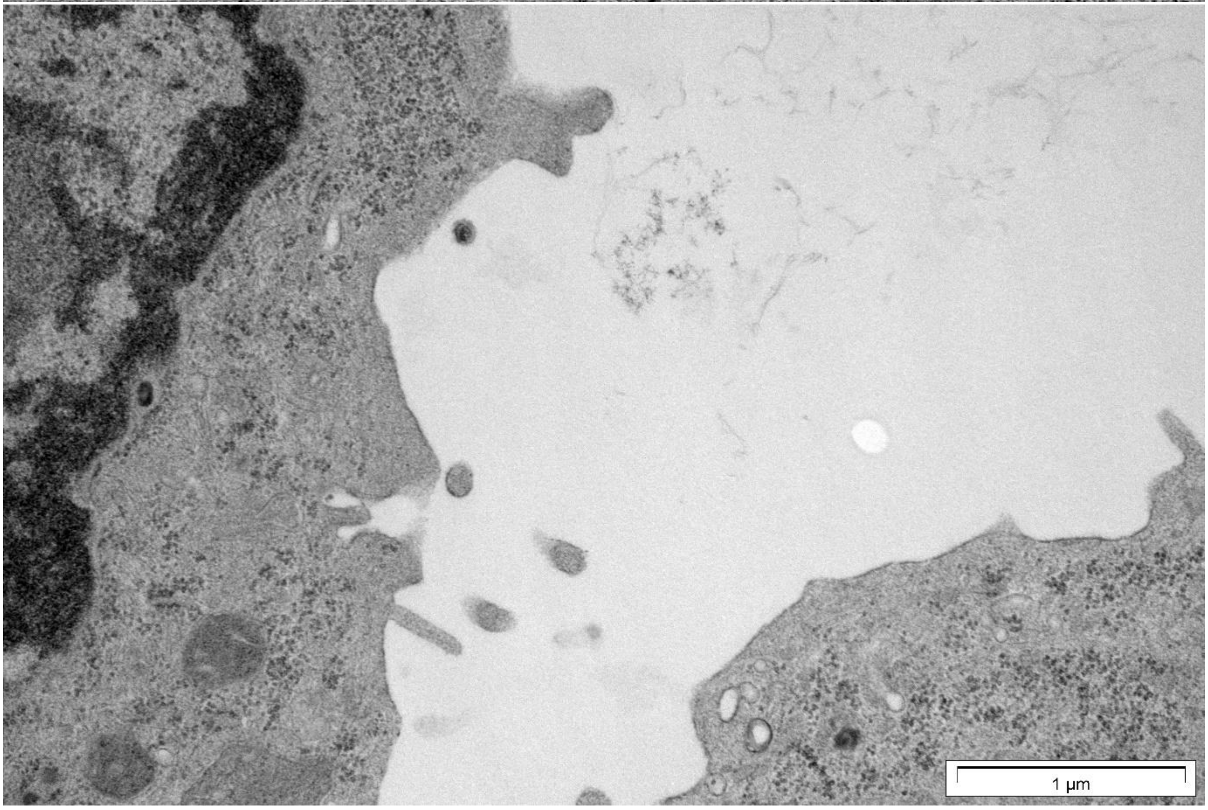
1 µm

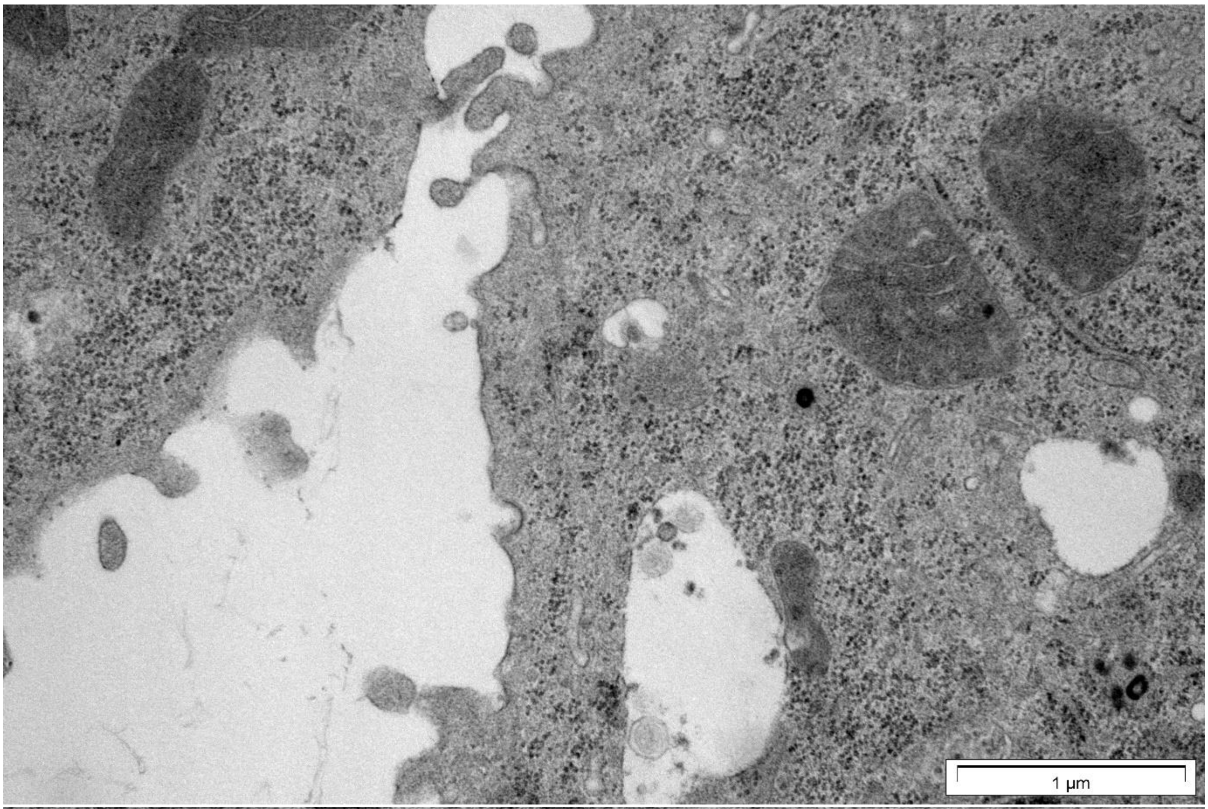
1 µm

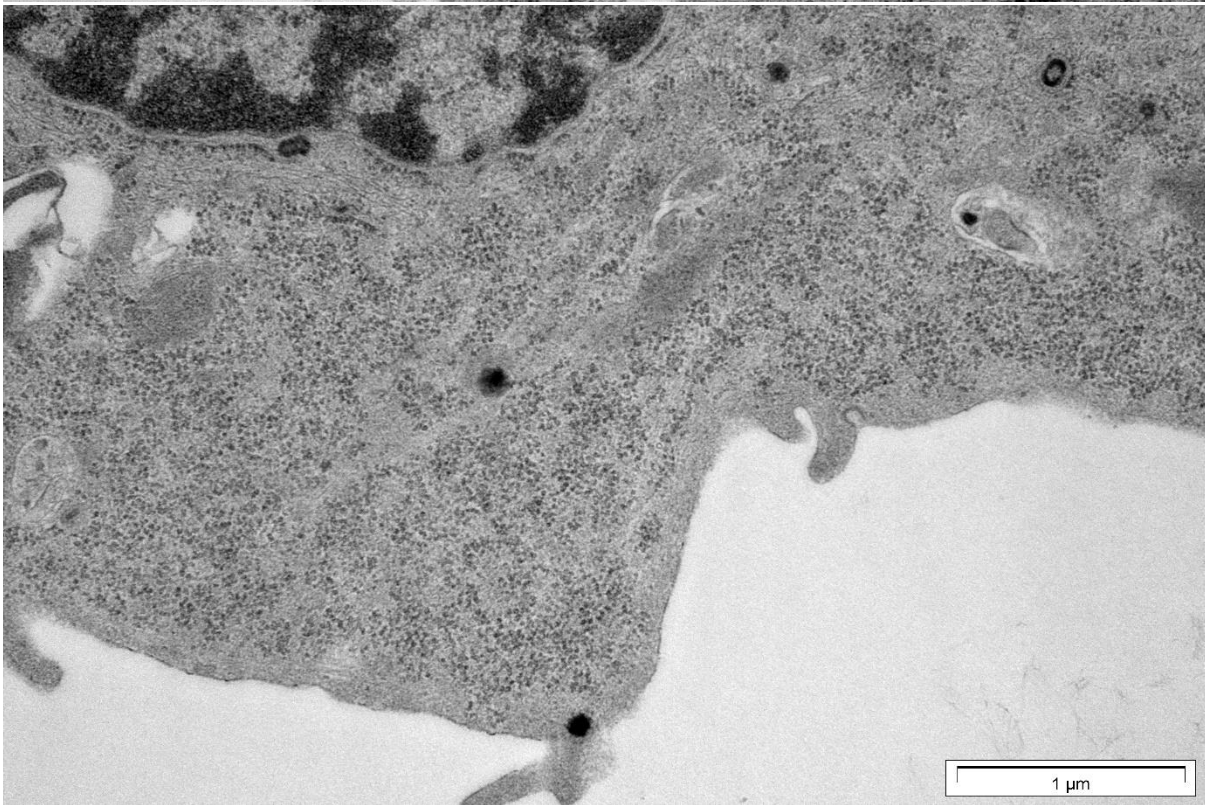
1 µm

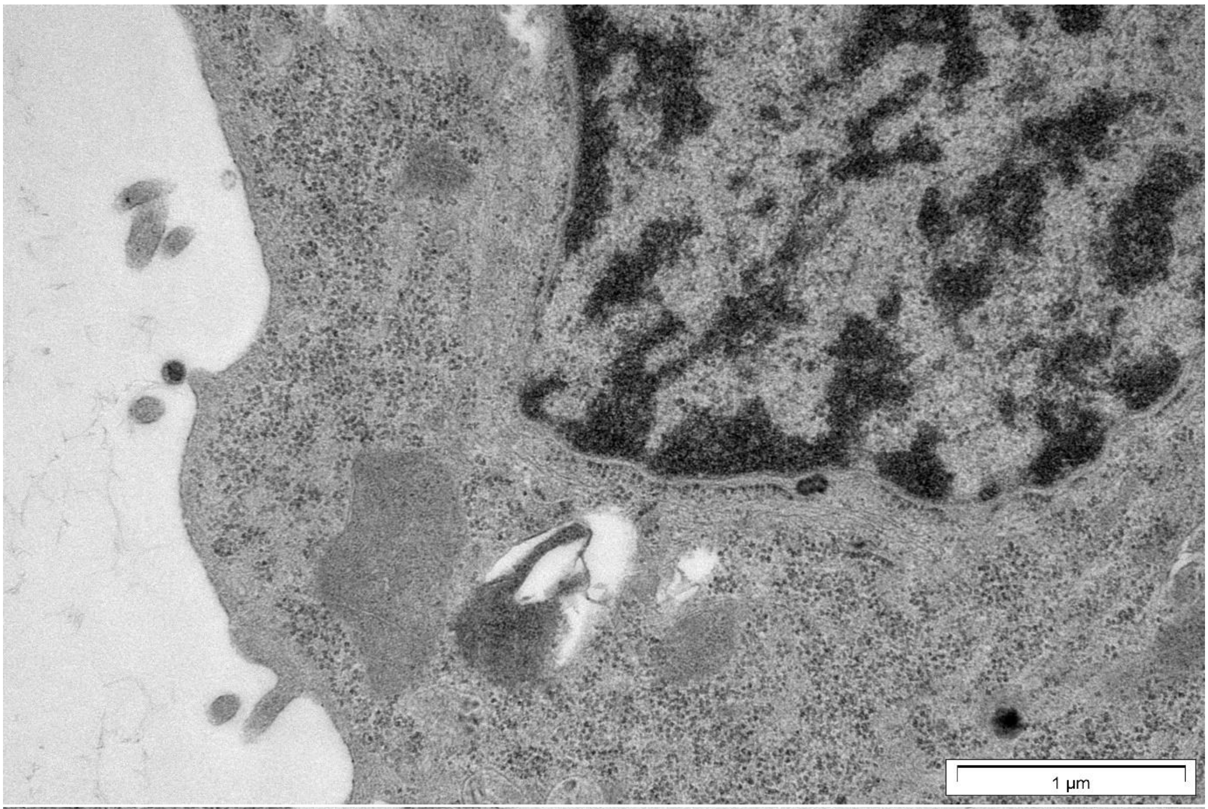
1 µm

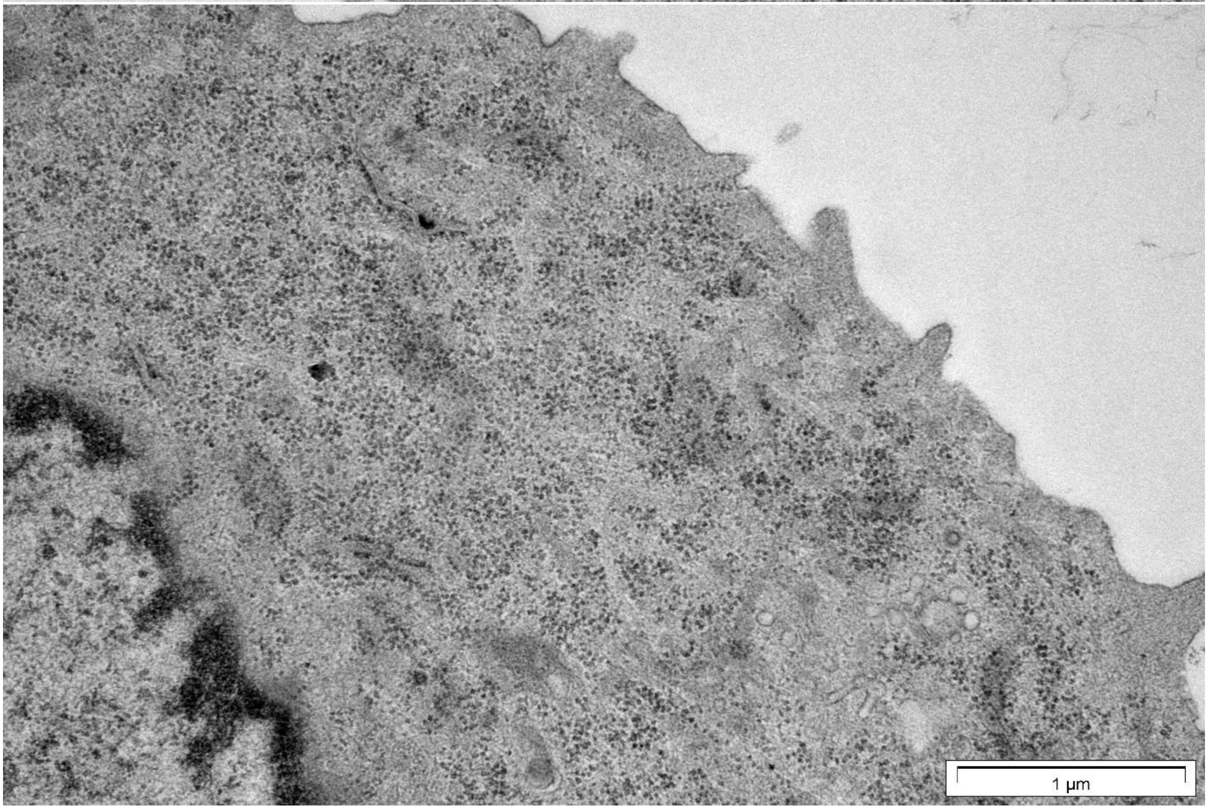
1 µm

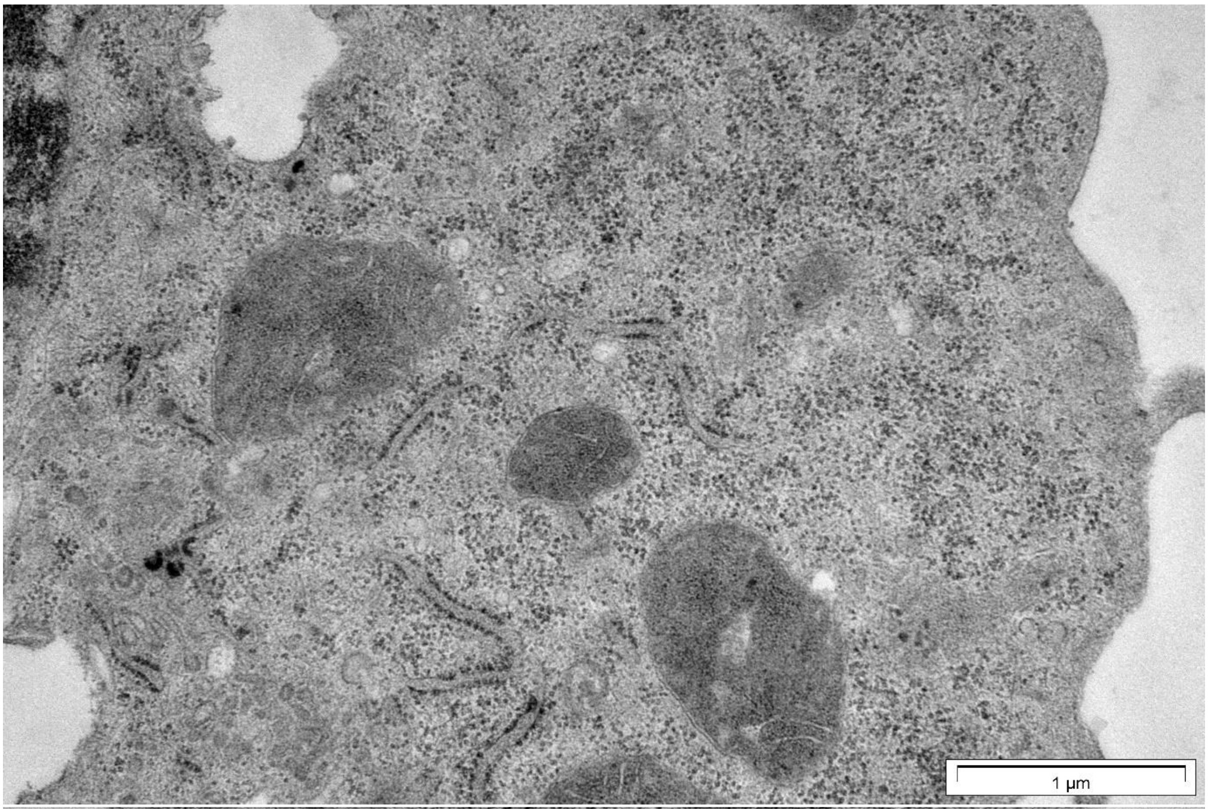
1 µm

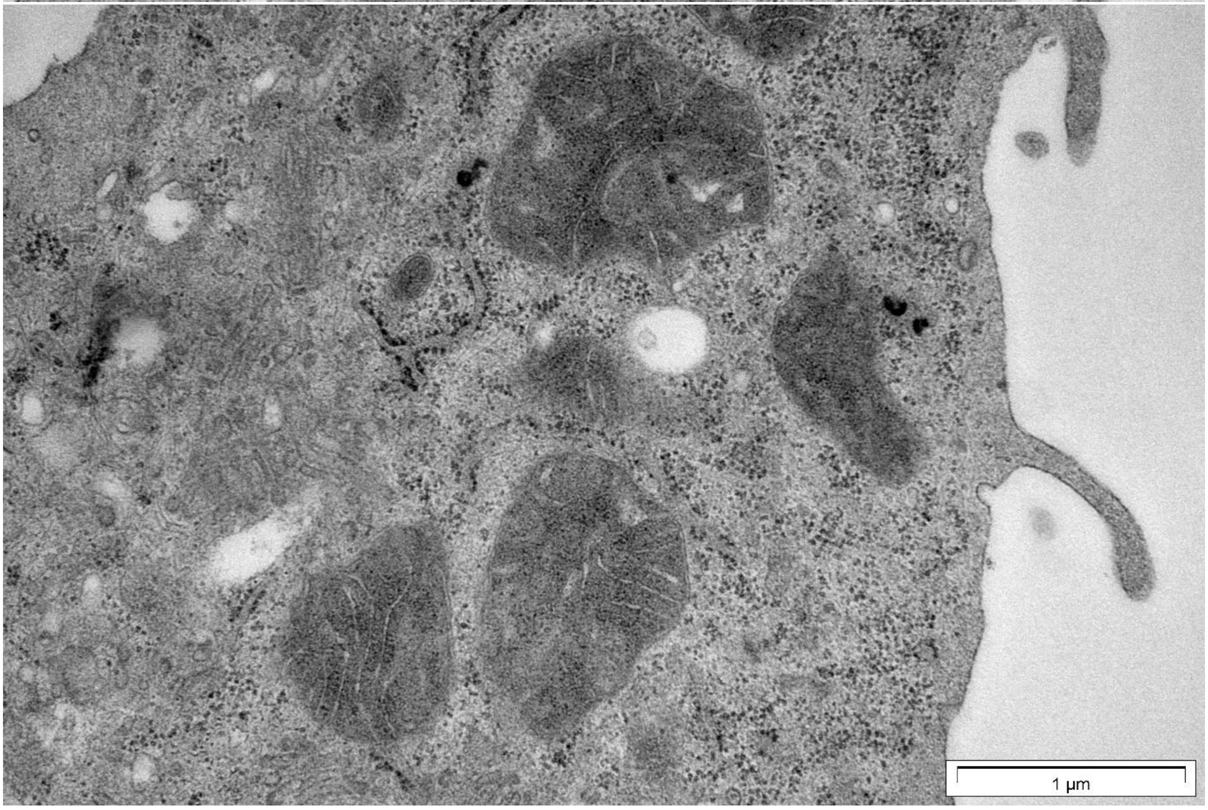
1 µm

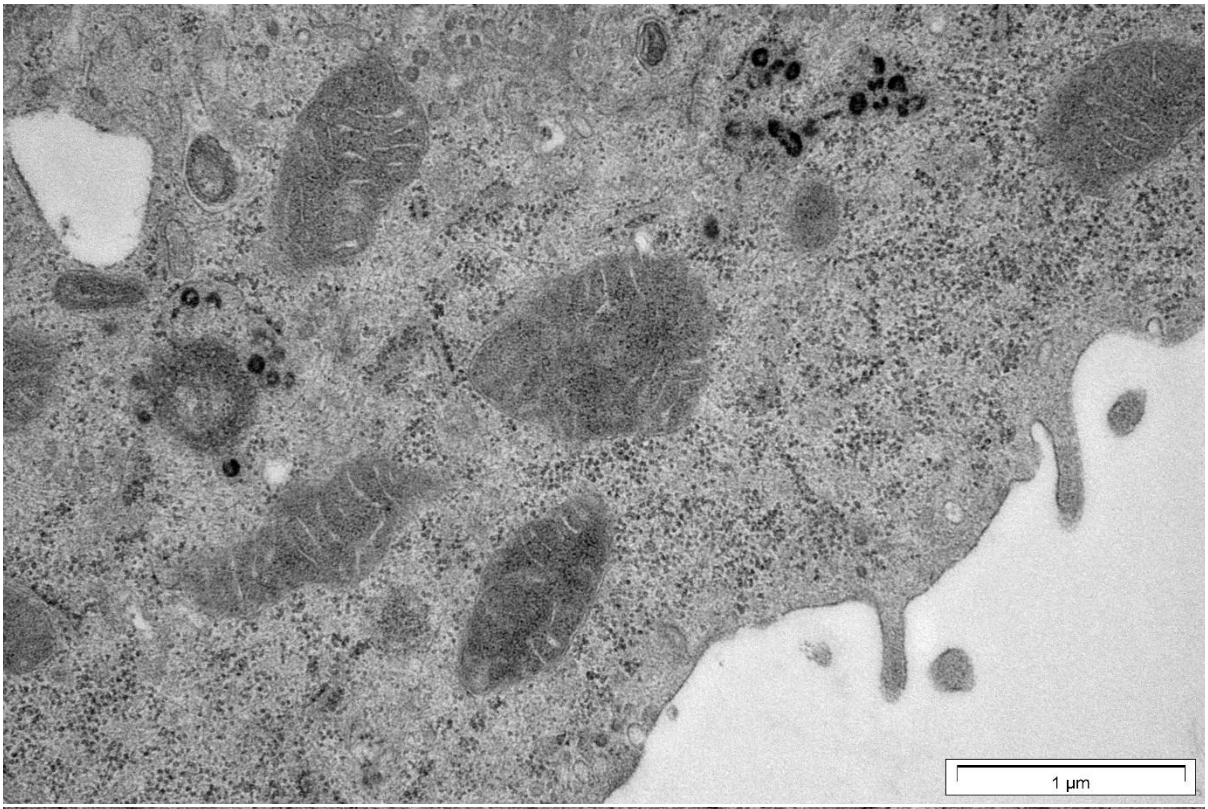
1 µm

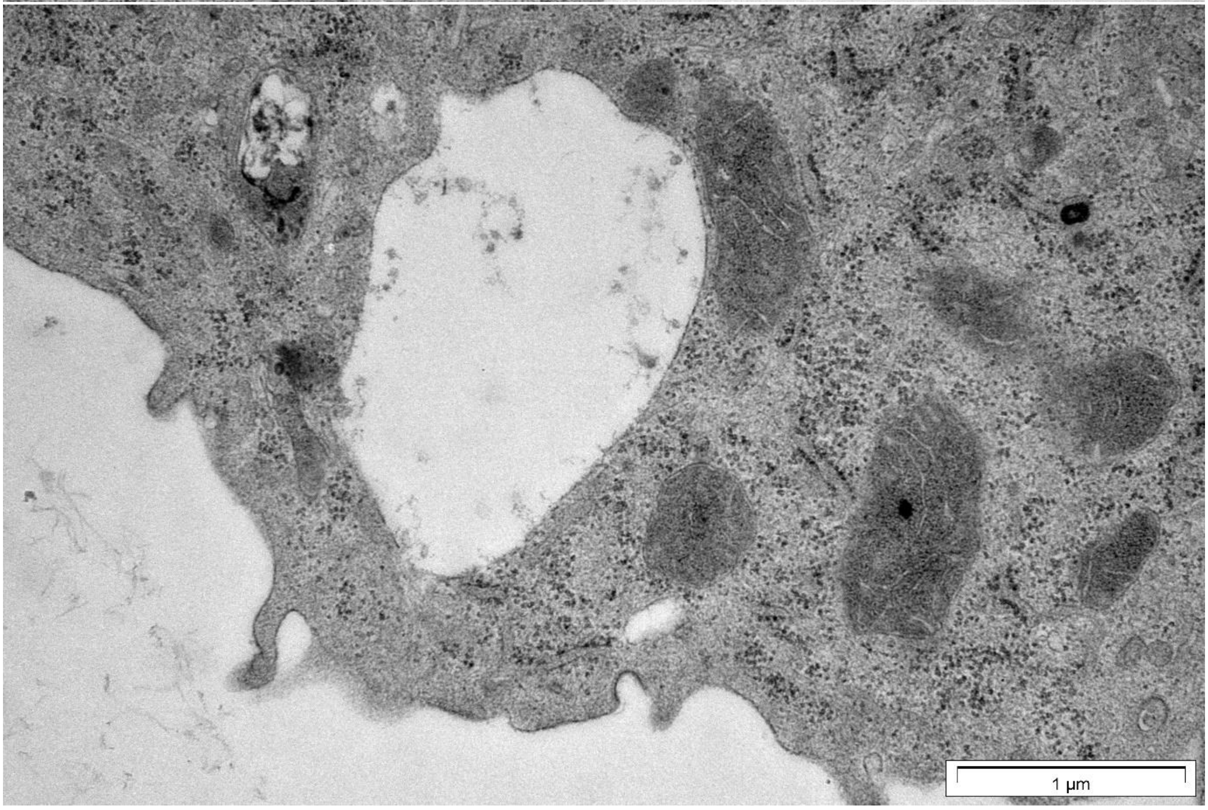
1 µm

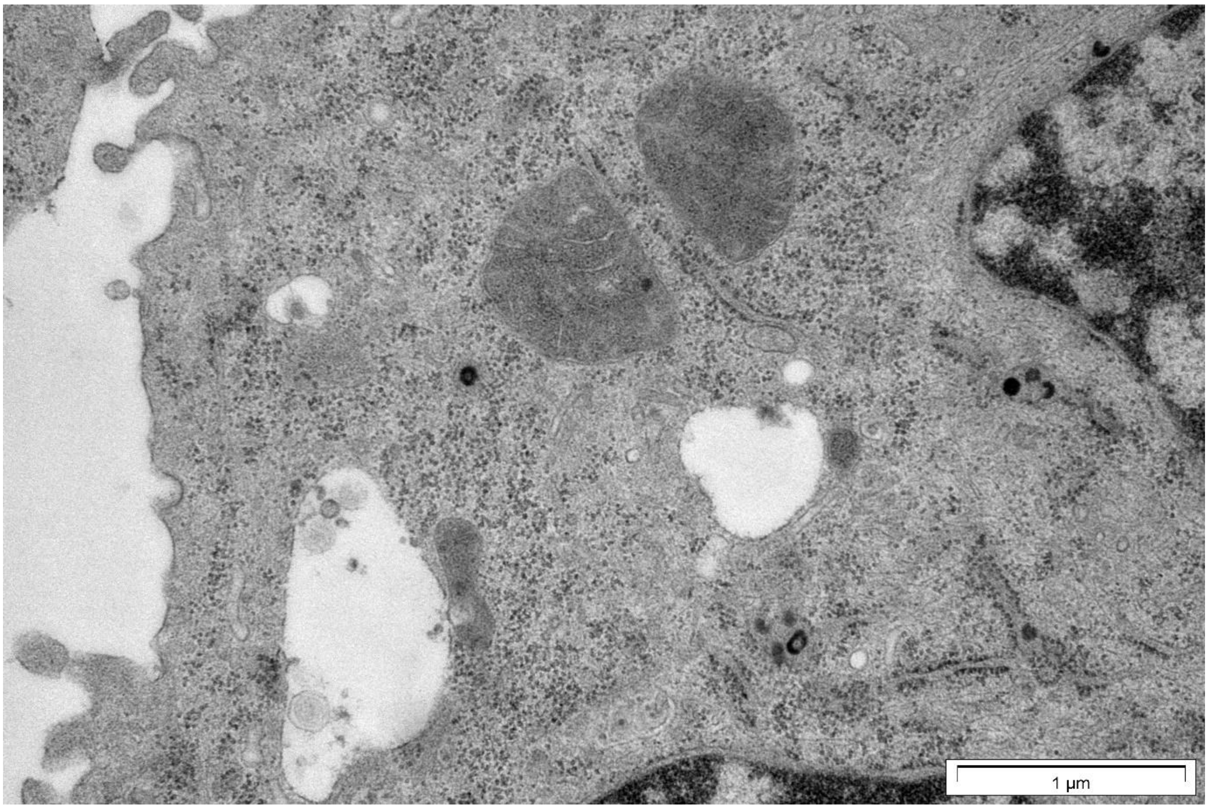
1 µm

Dextran sulfate, 1 min.

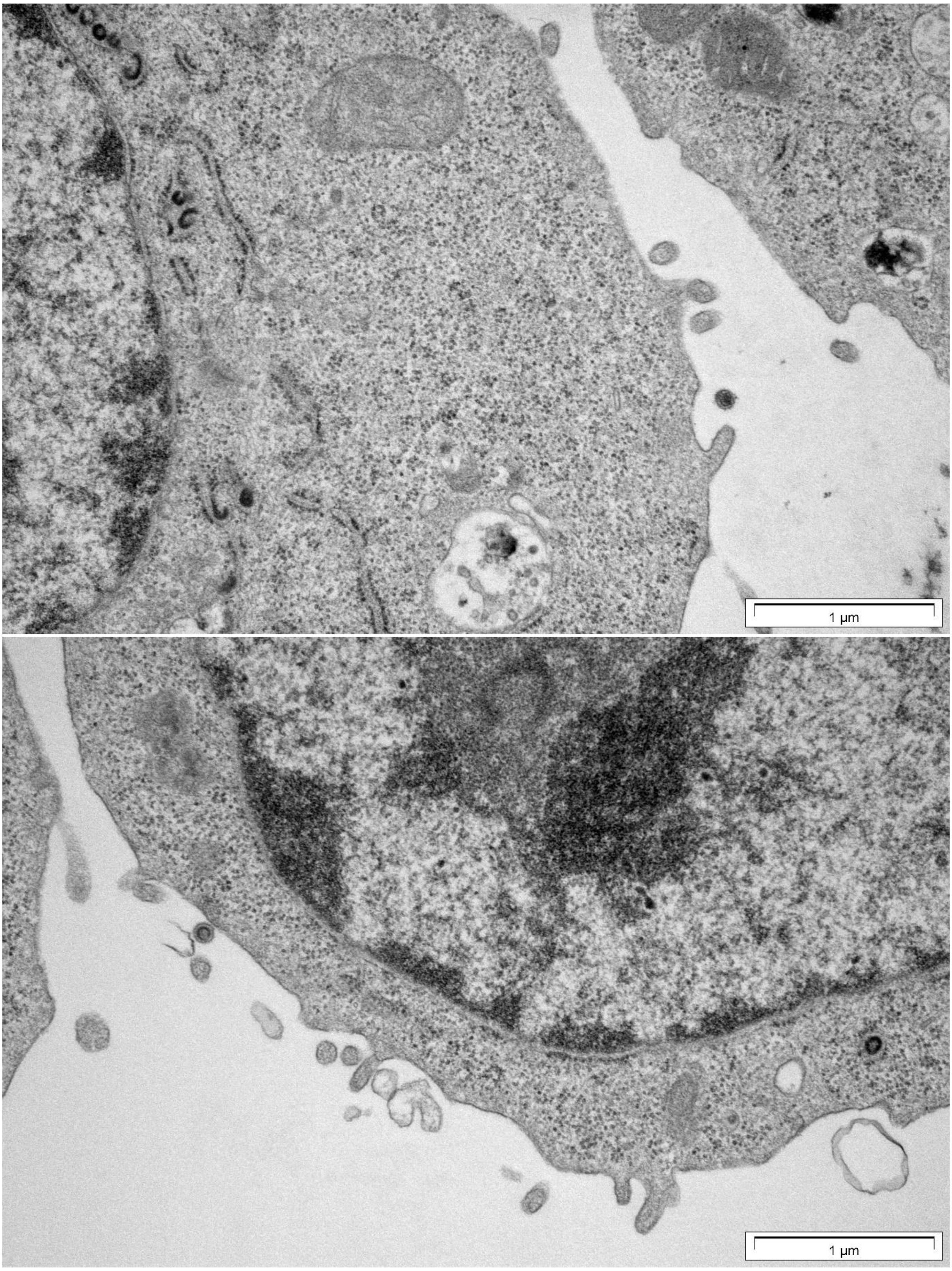

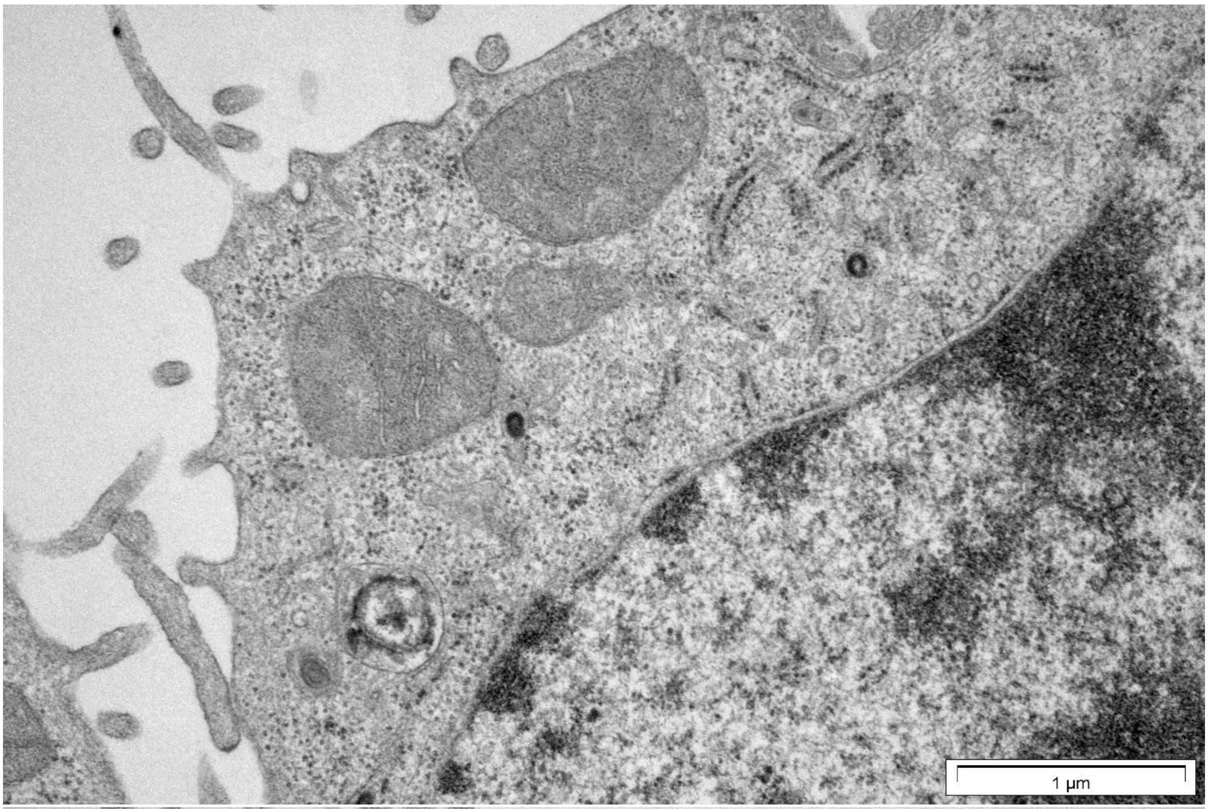
1 µm

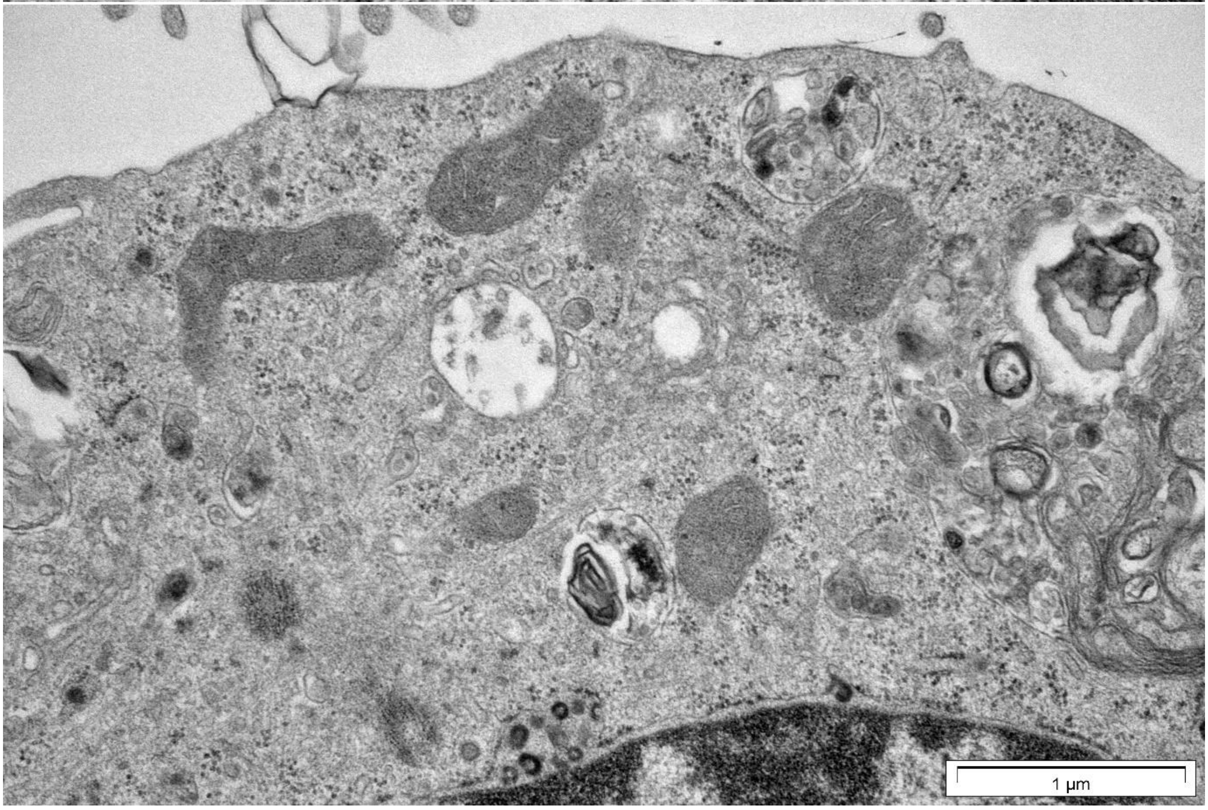
1 µm

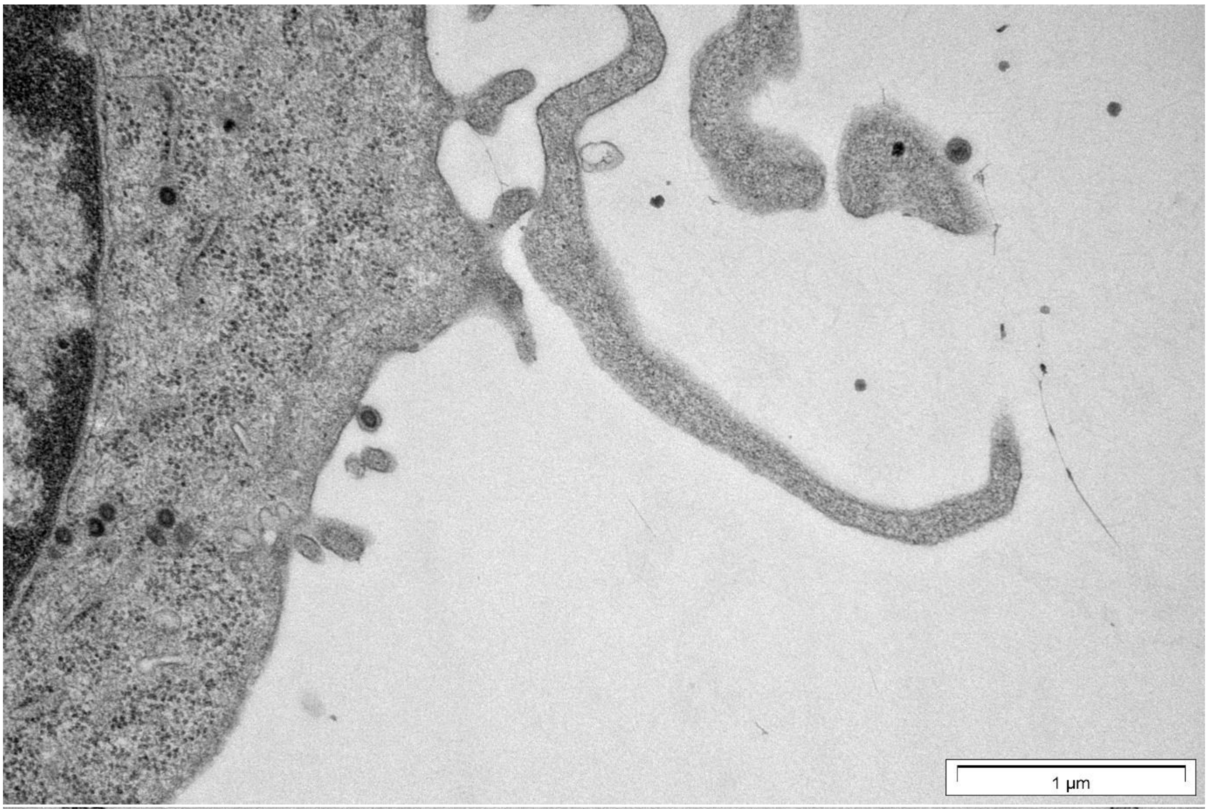
1 µm

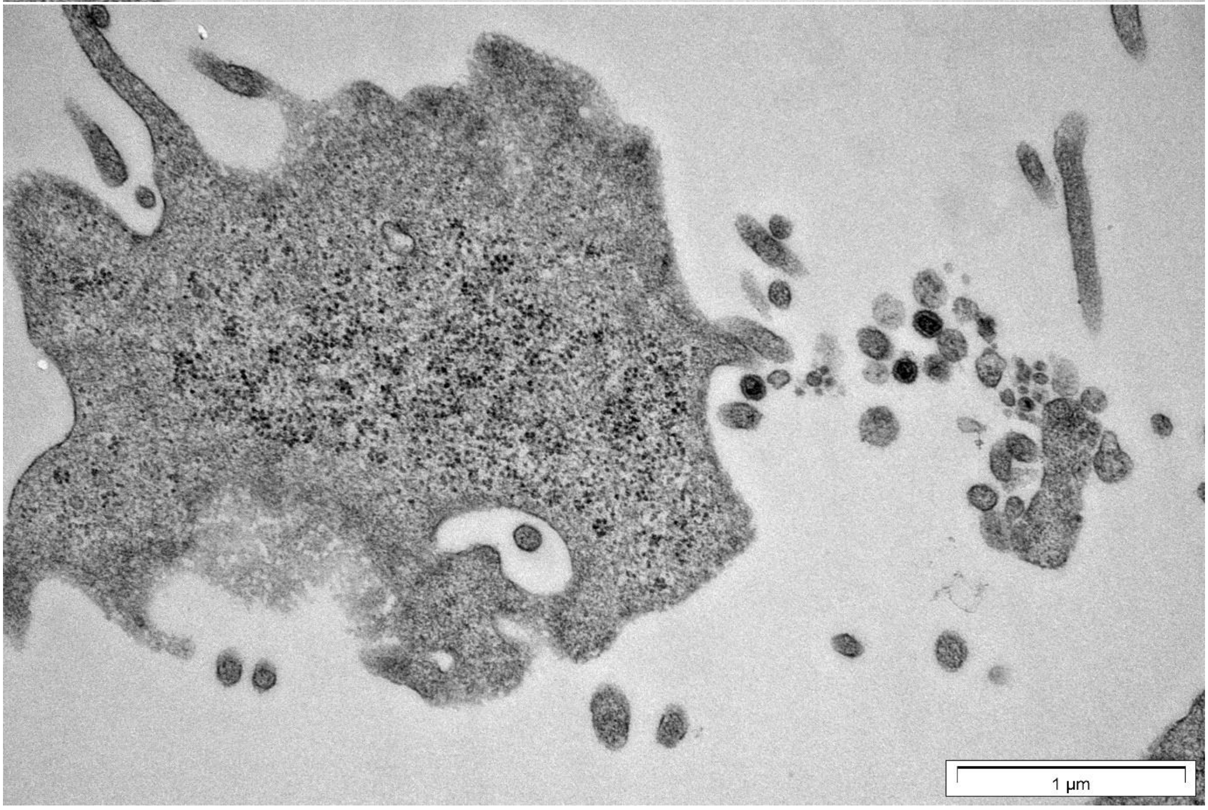
1 µm

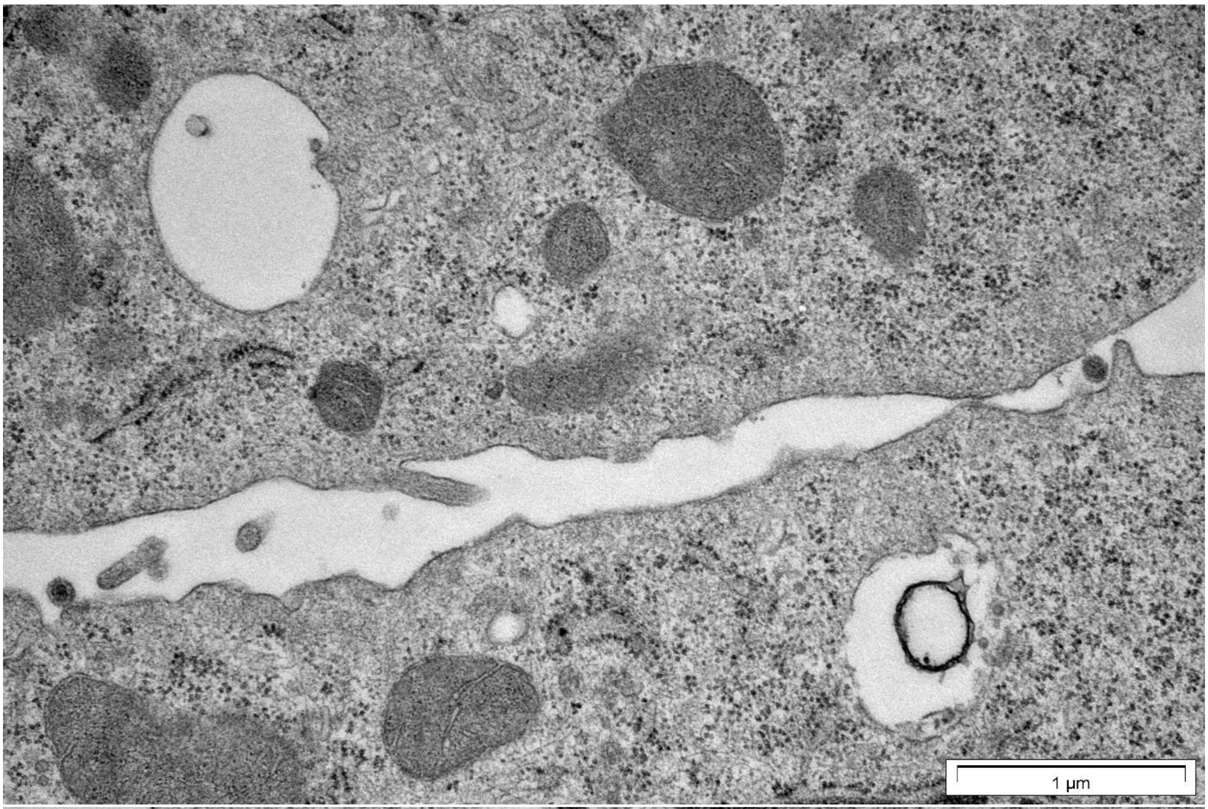
1 µm

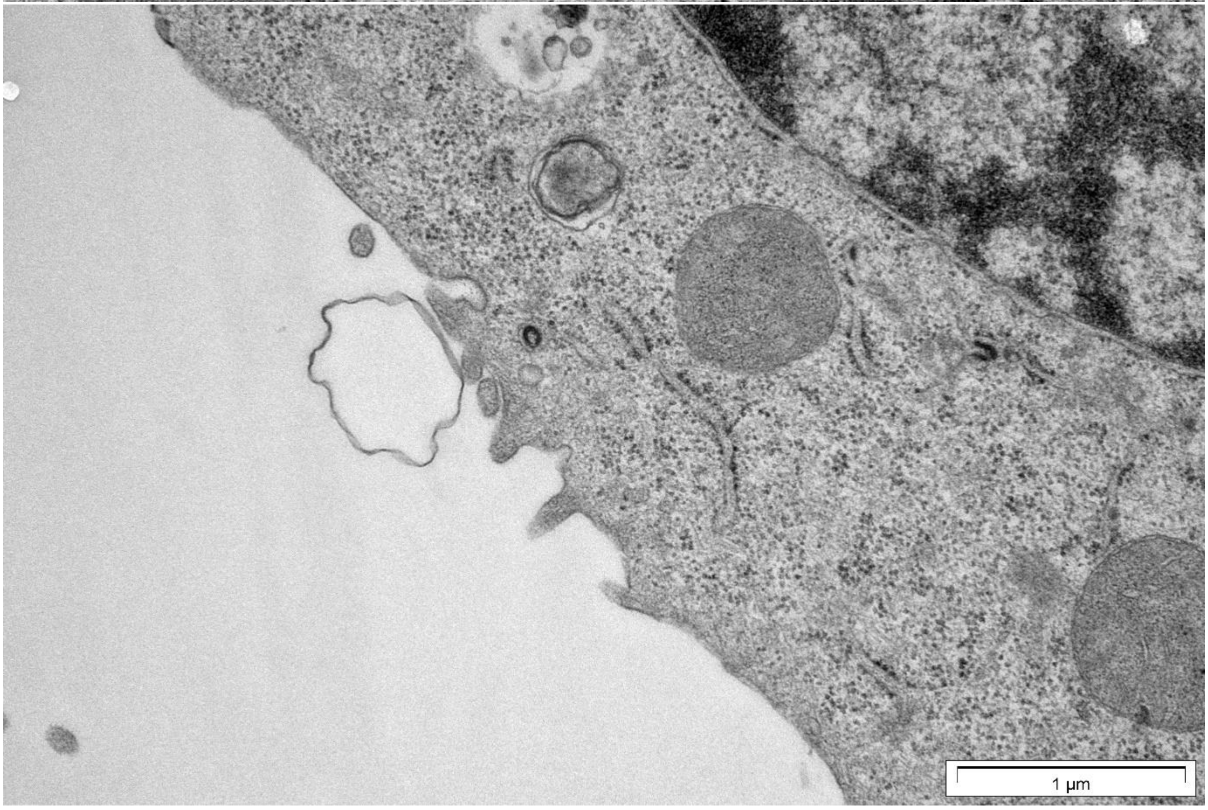
1 µm

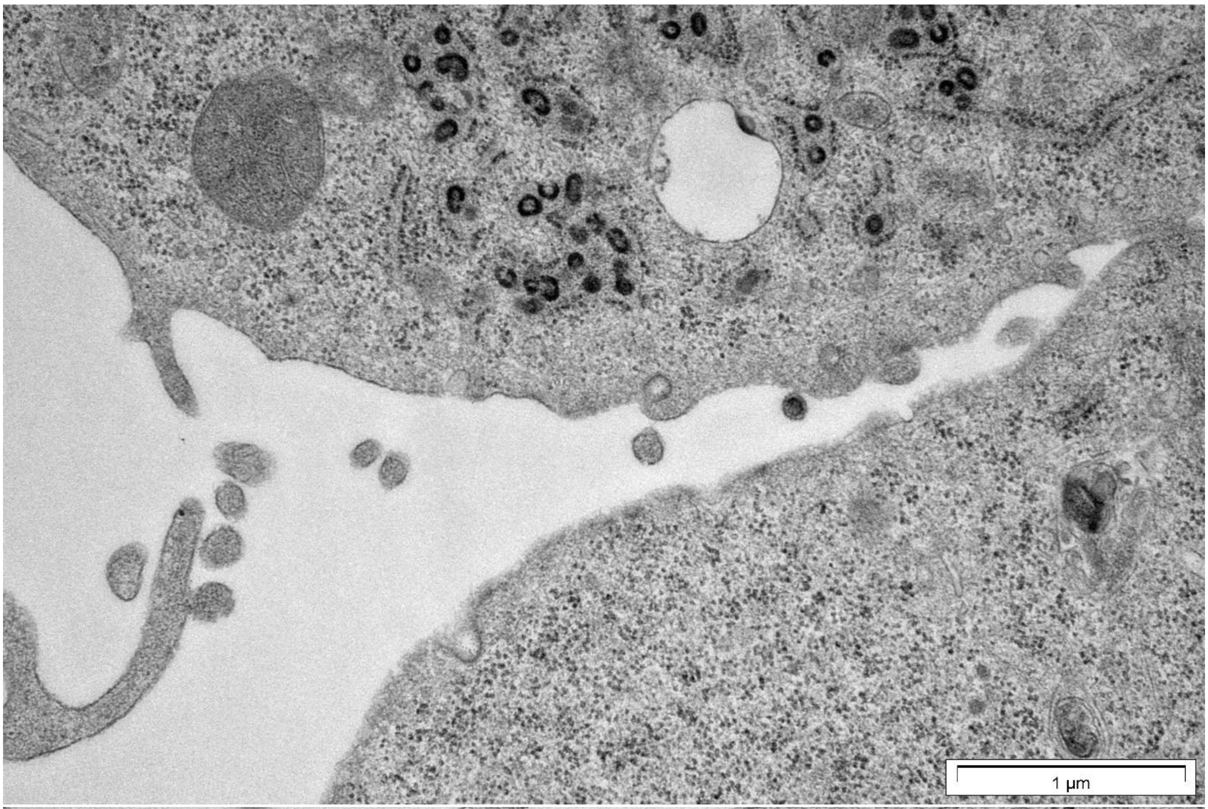
1 µm
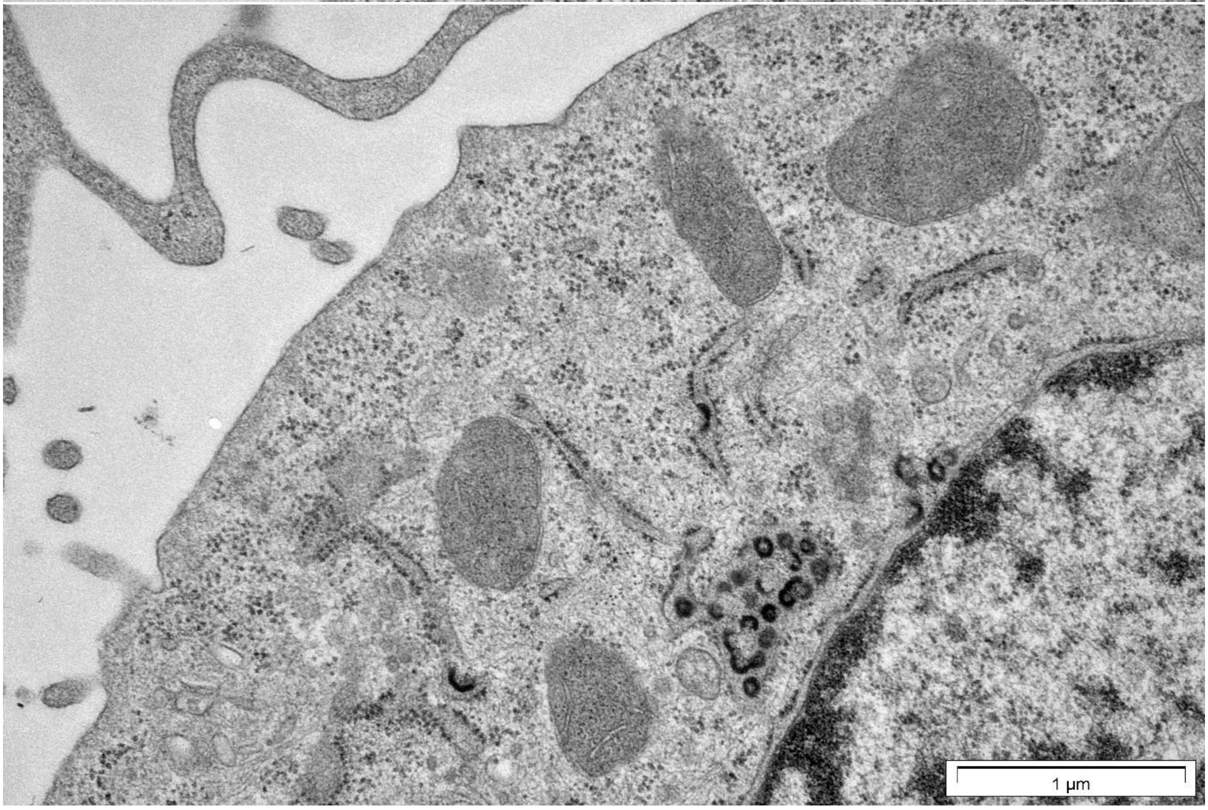
1 µm

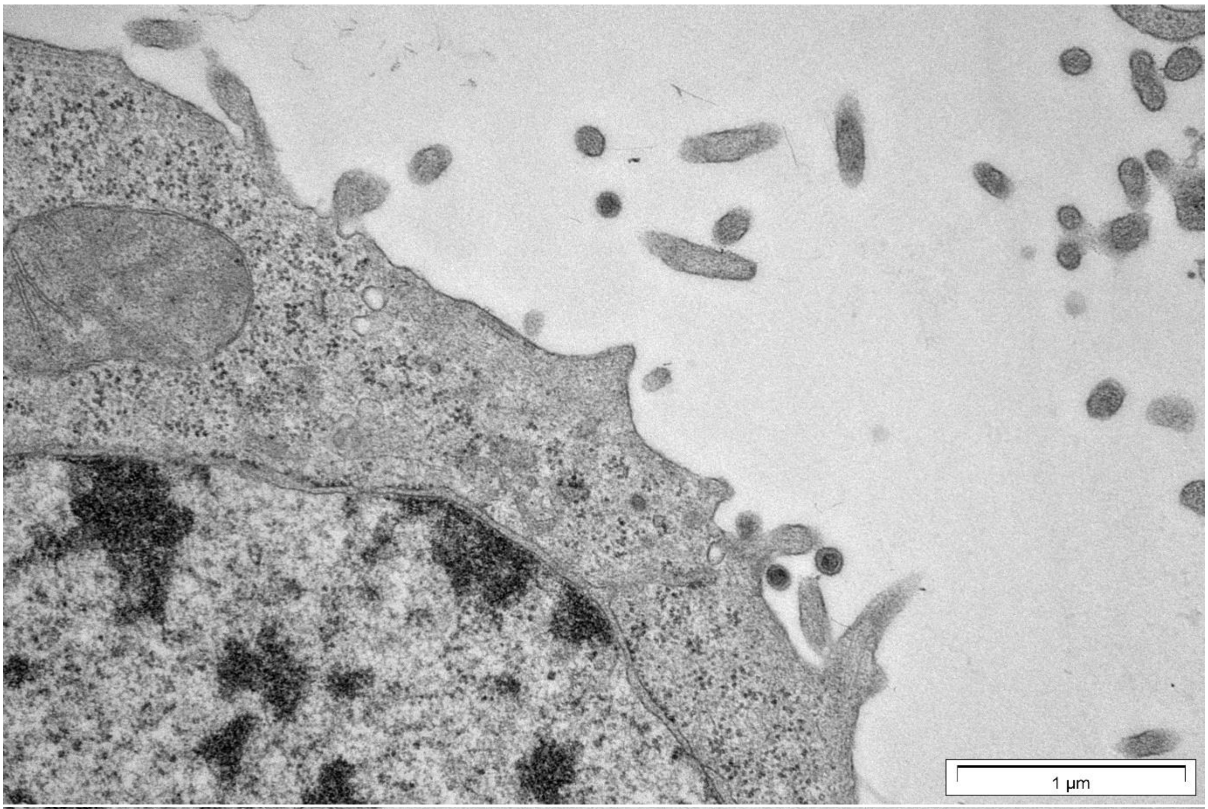
1 µm
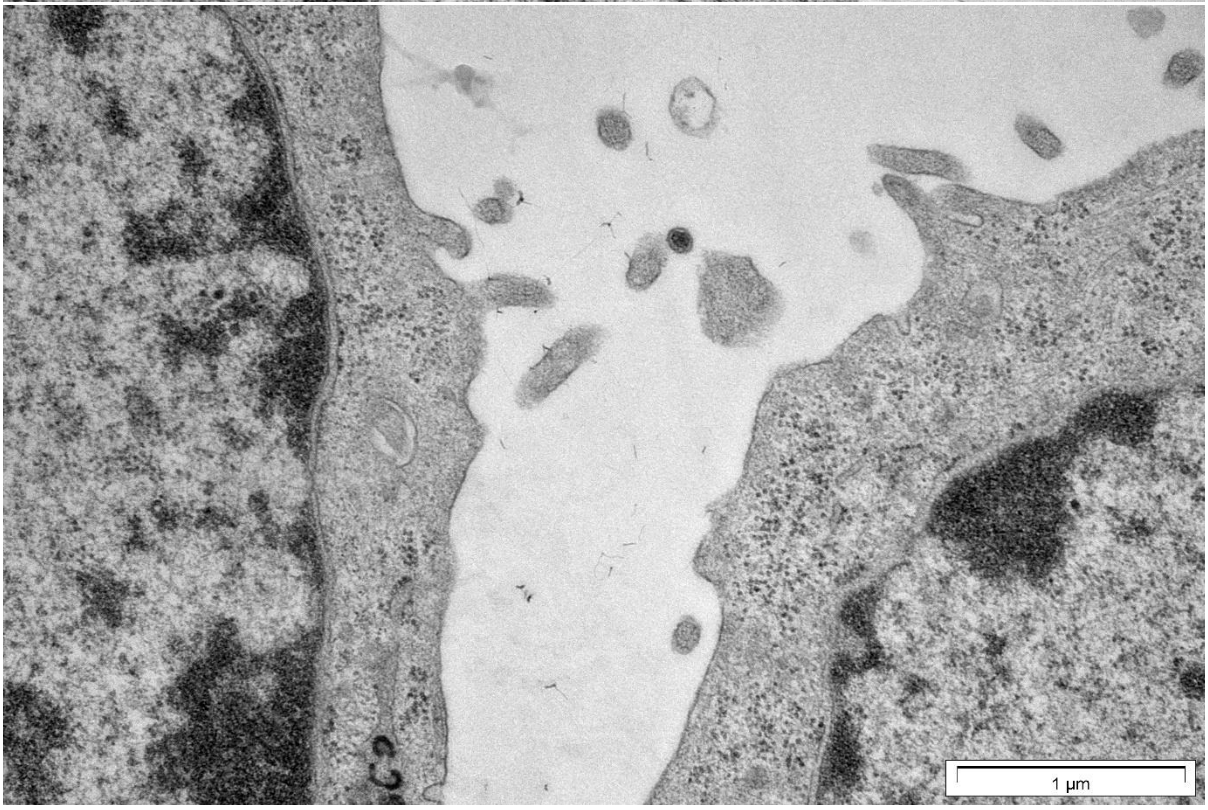
1 µm

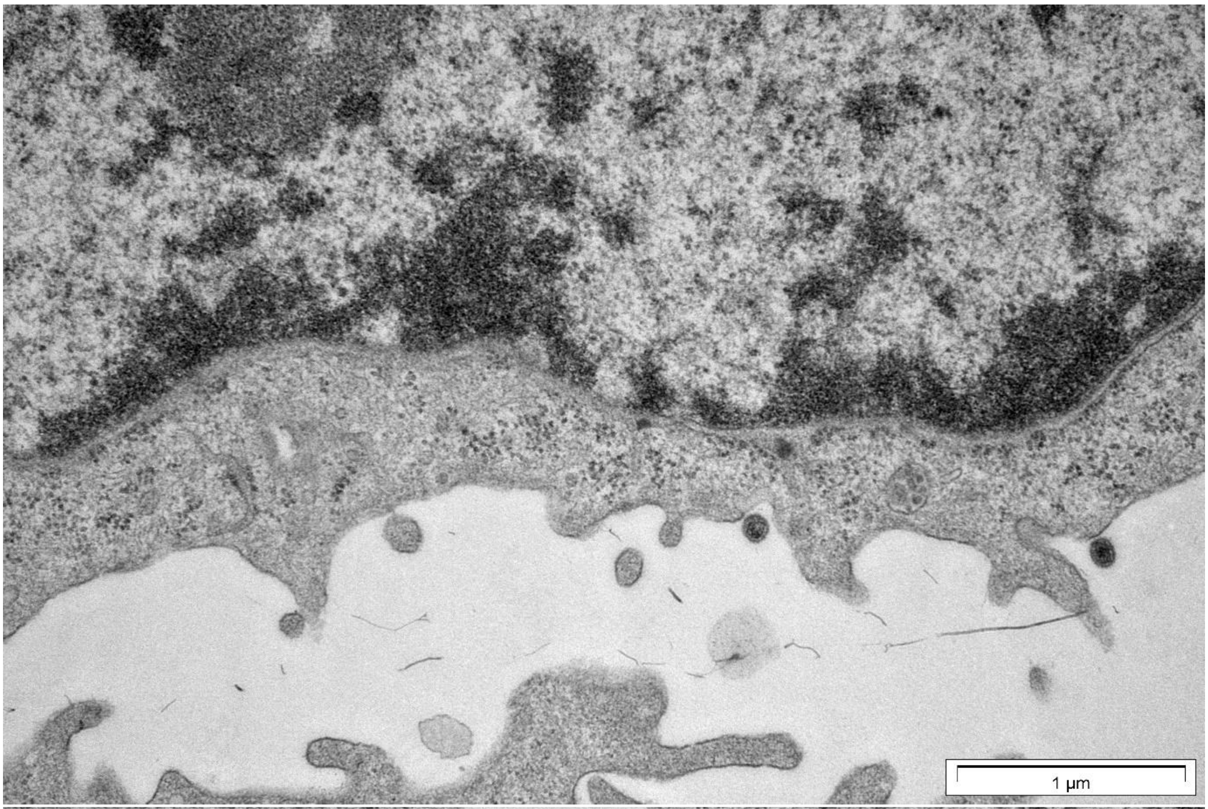
1 µm

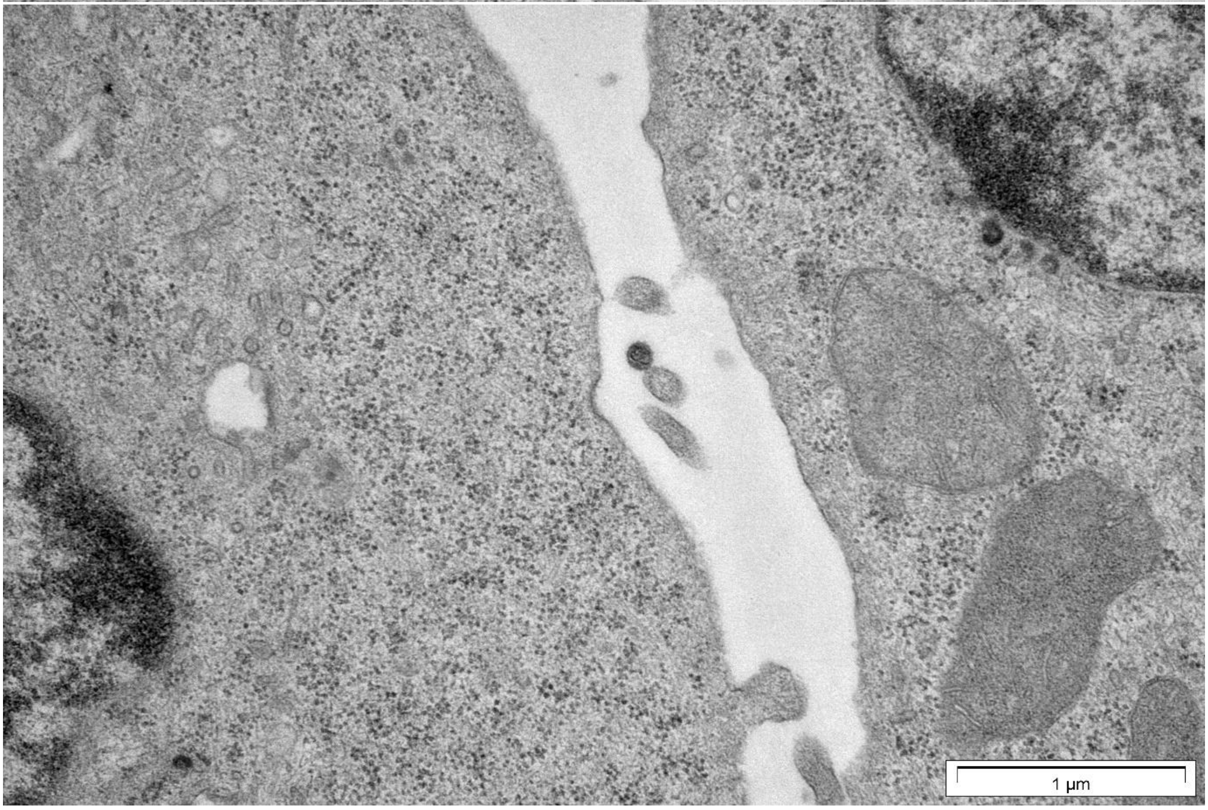
1 µm

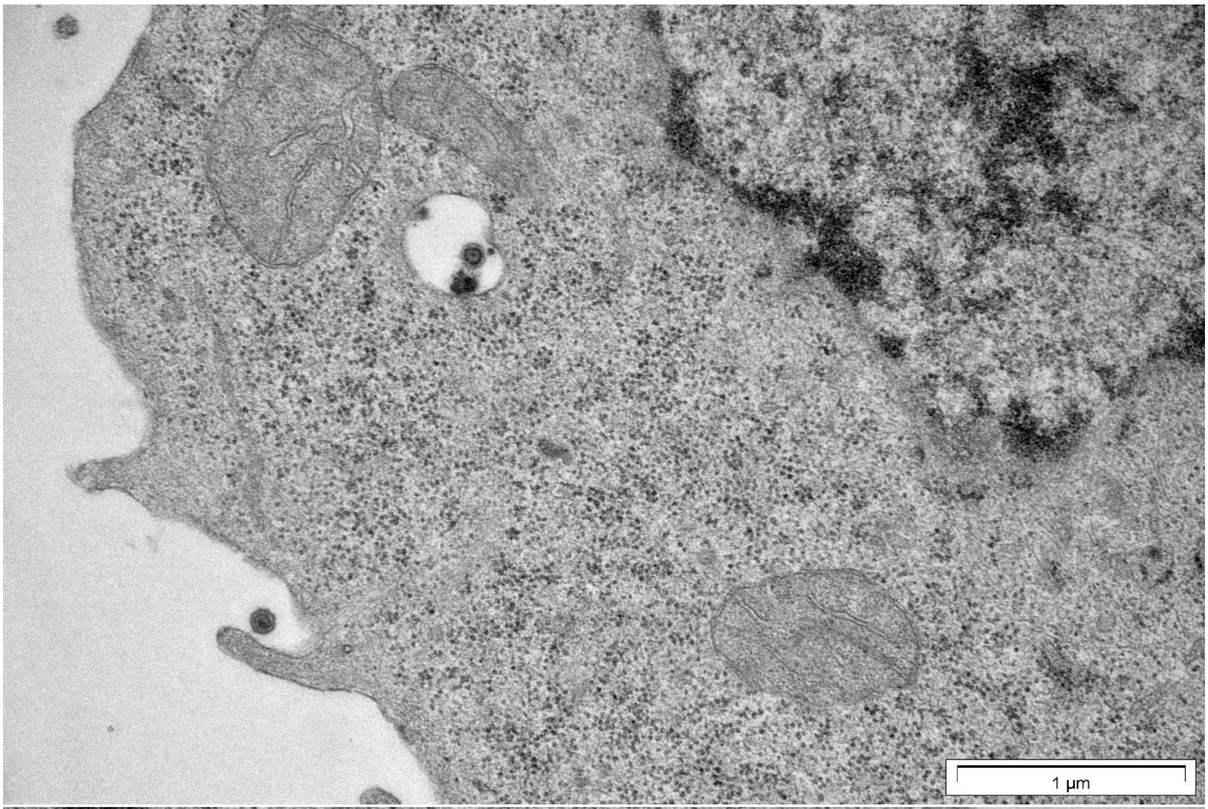
1 µm

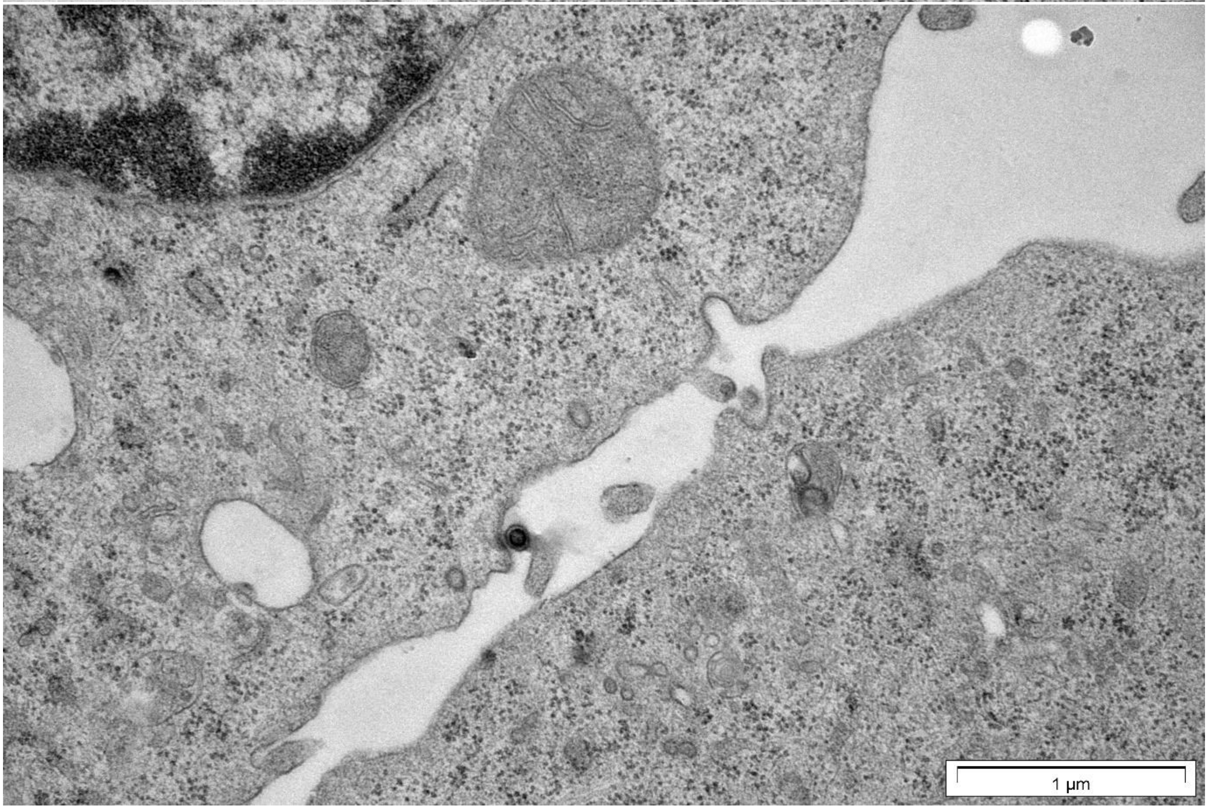
1 µm

Dextran sulfate, 5 min.

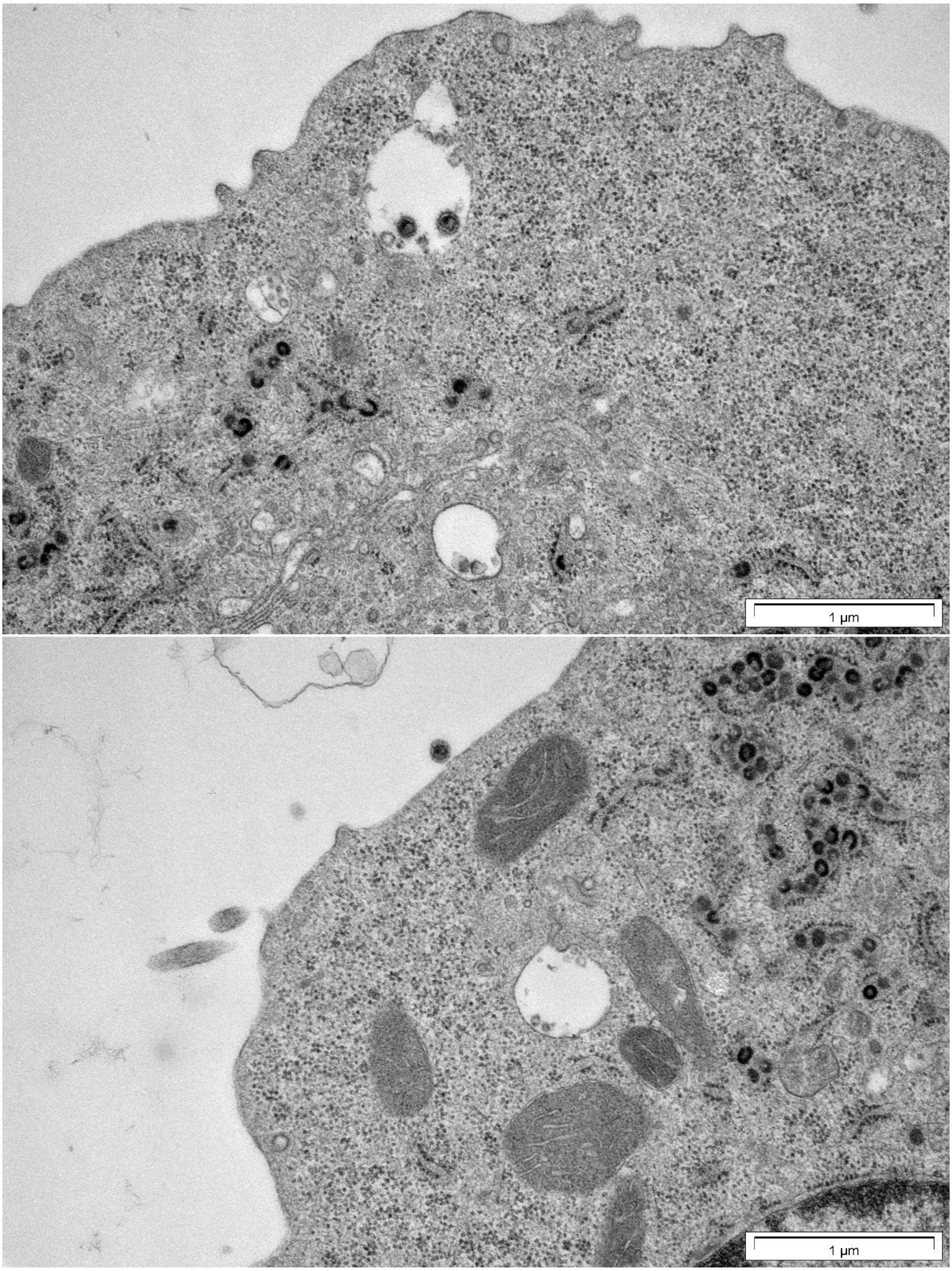

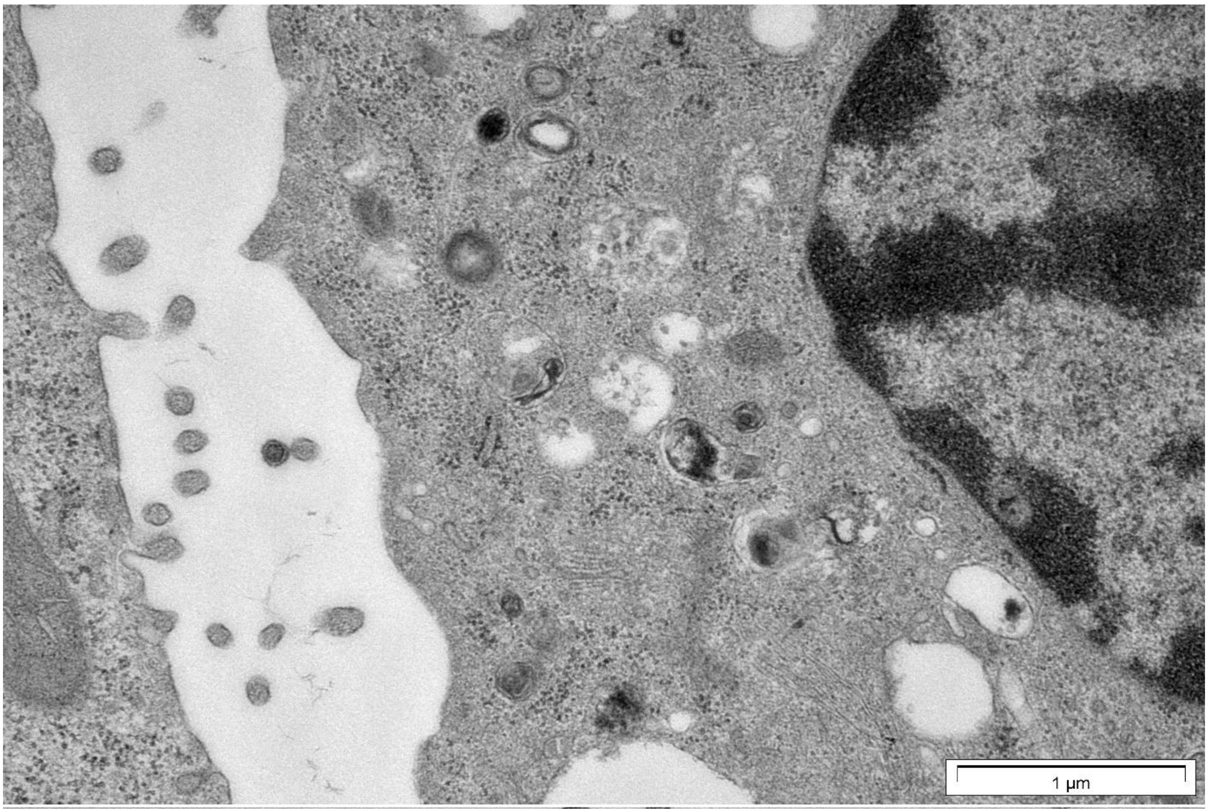
1 µm

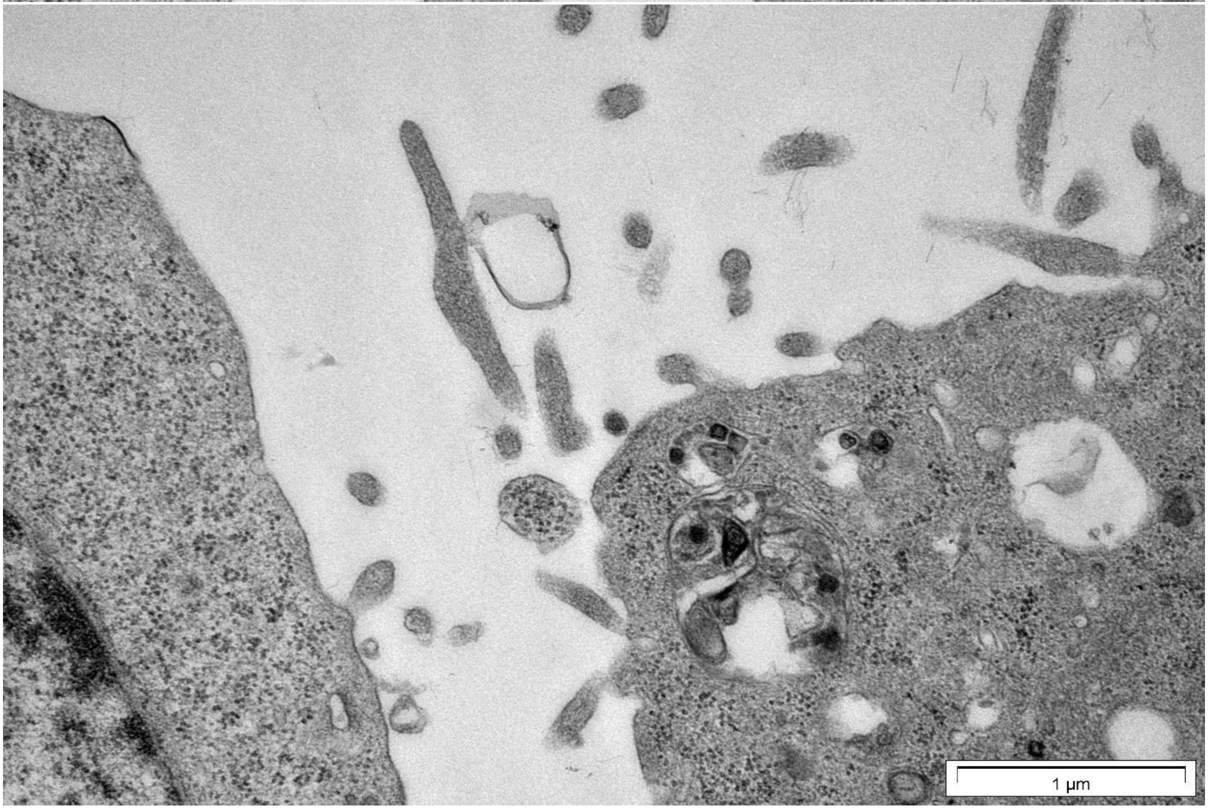
1 µm

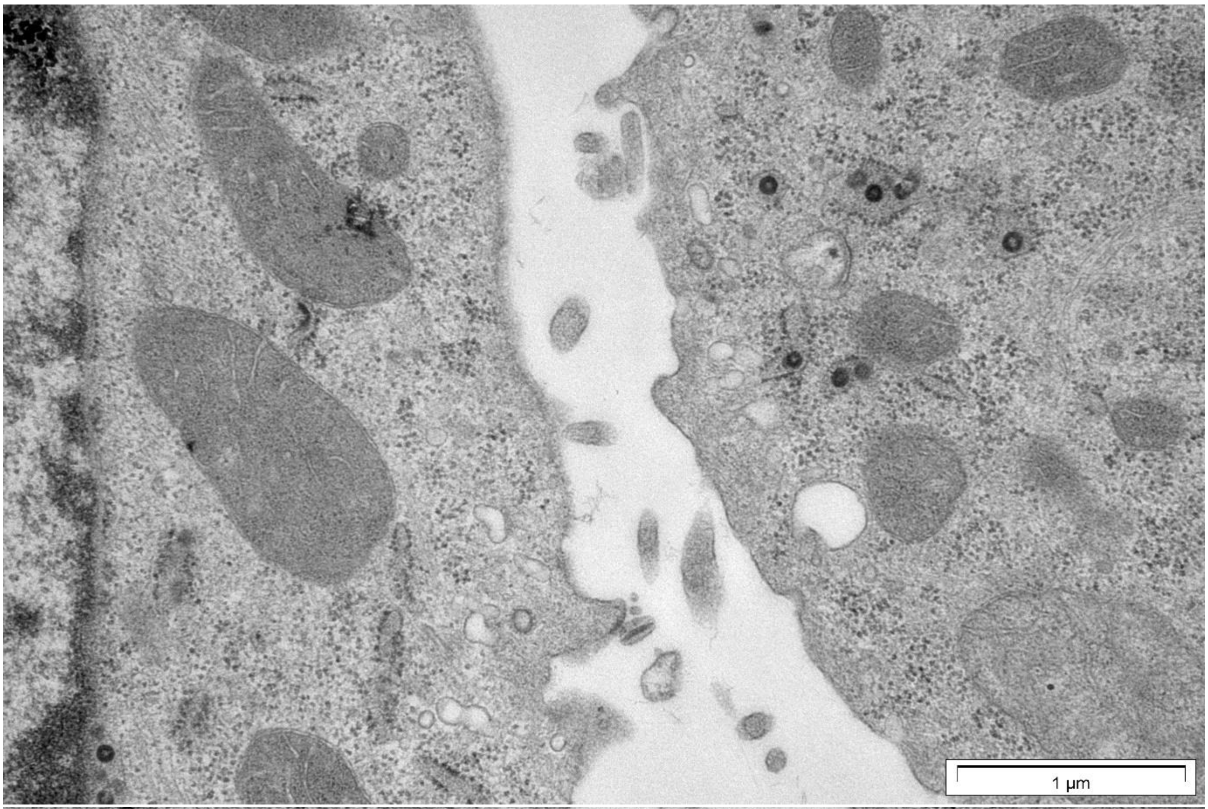
1 µm

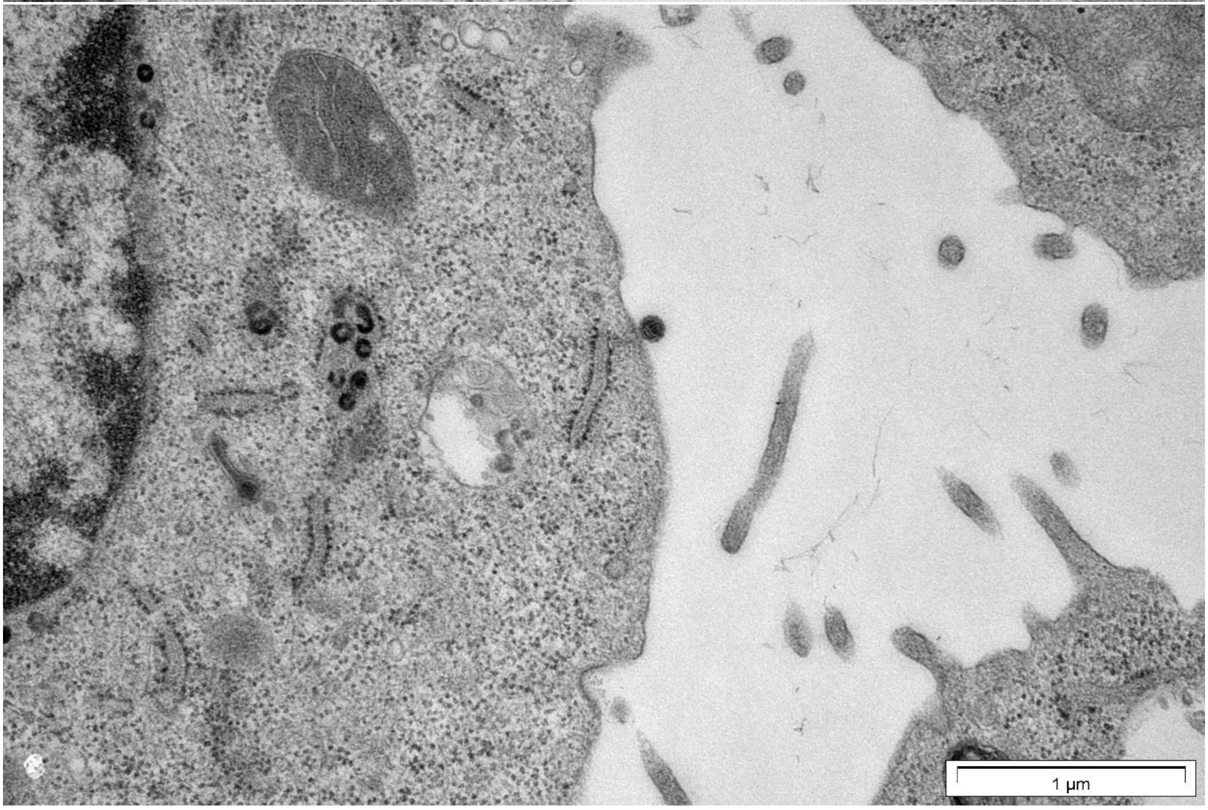
1 µm

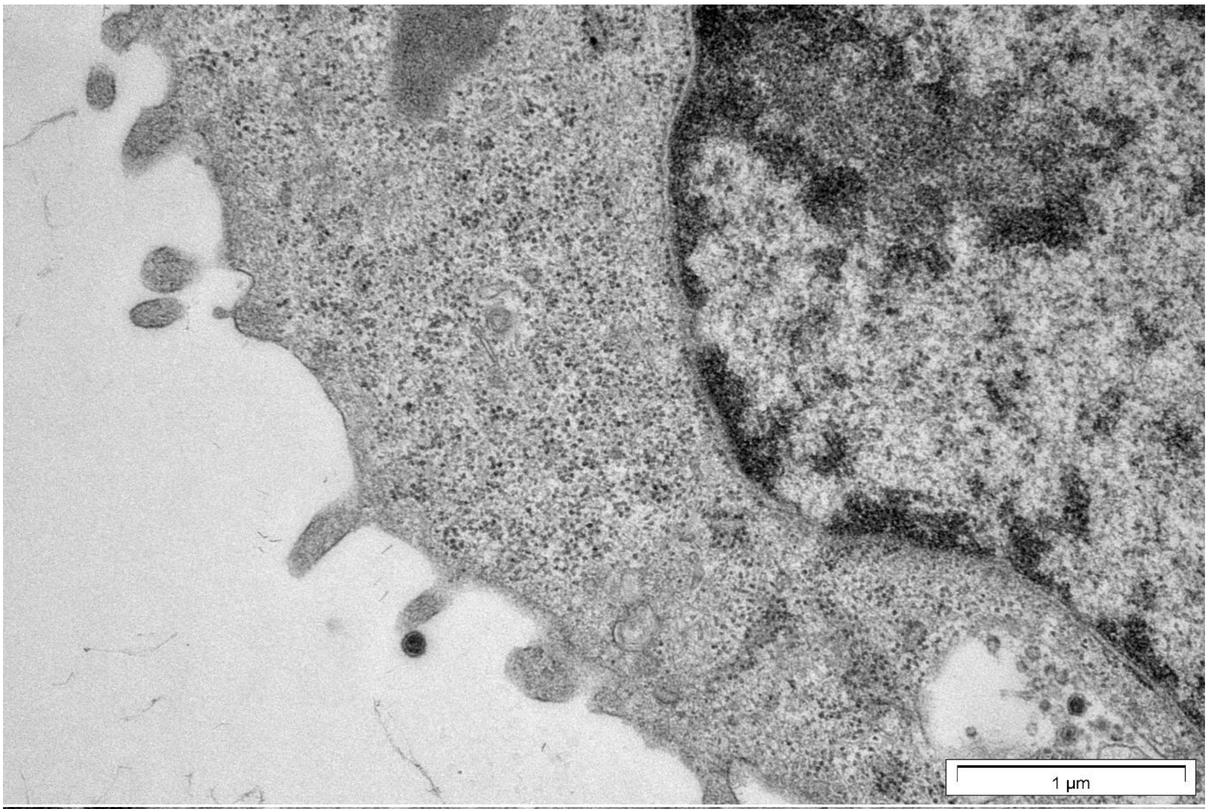
1 µm

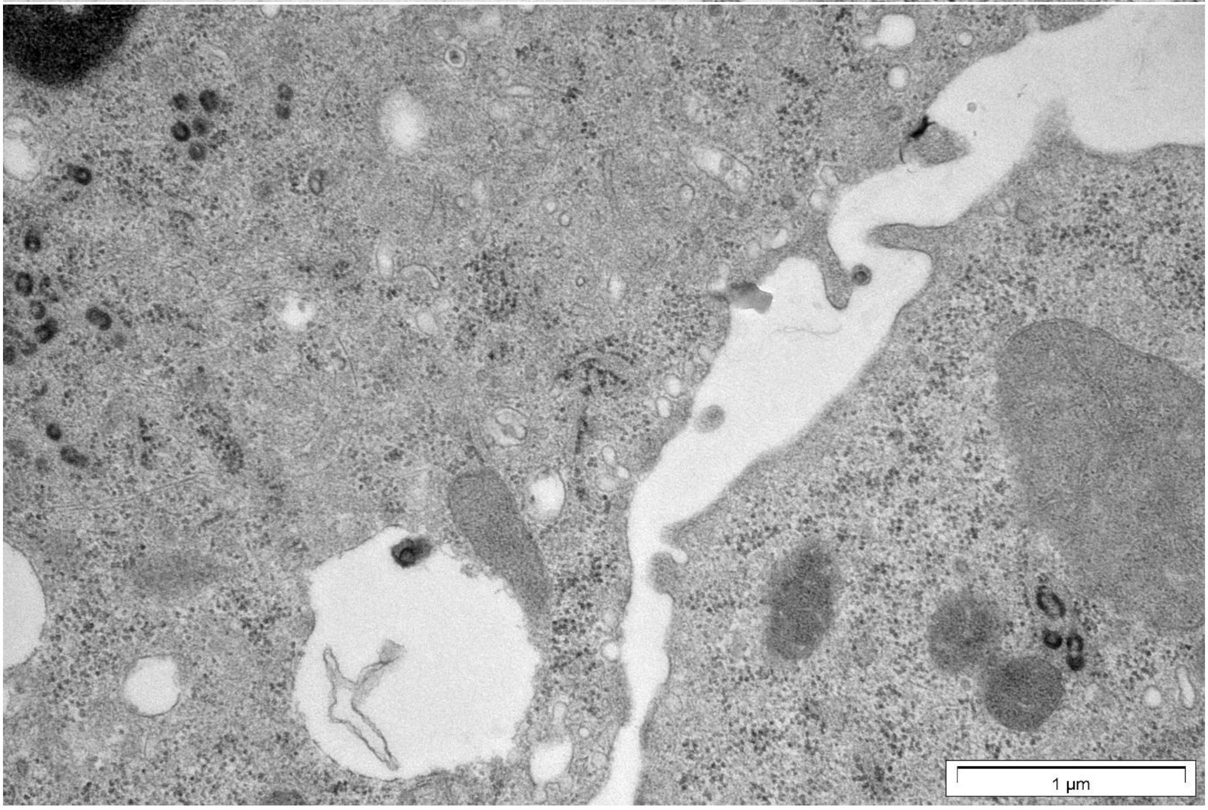
1 µm

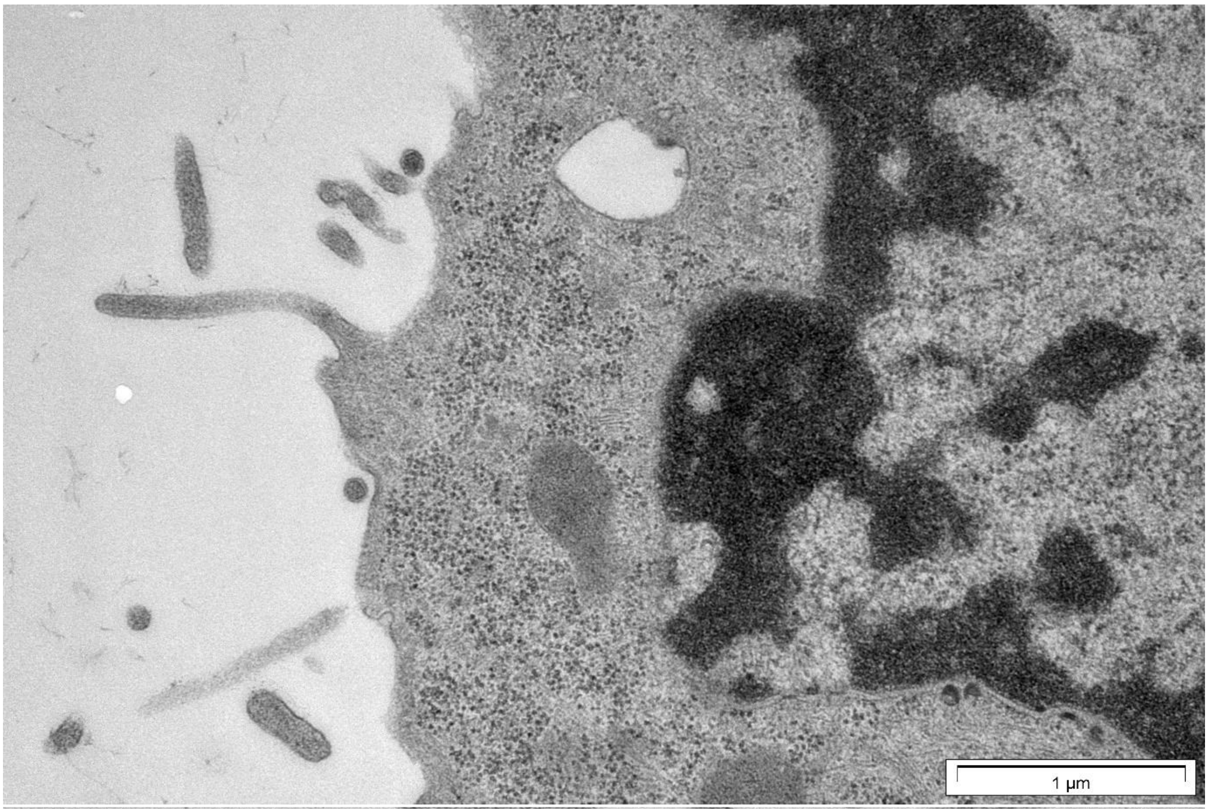
1 µm

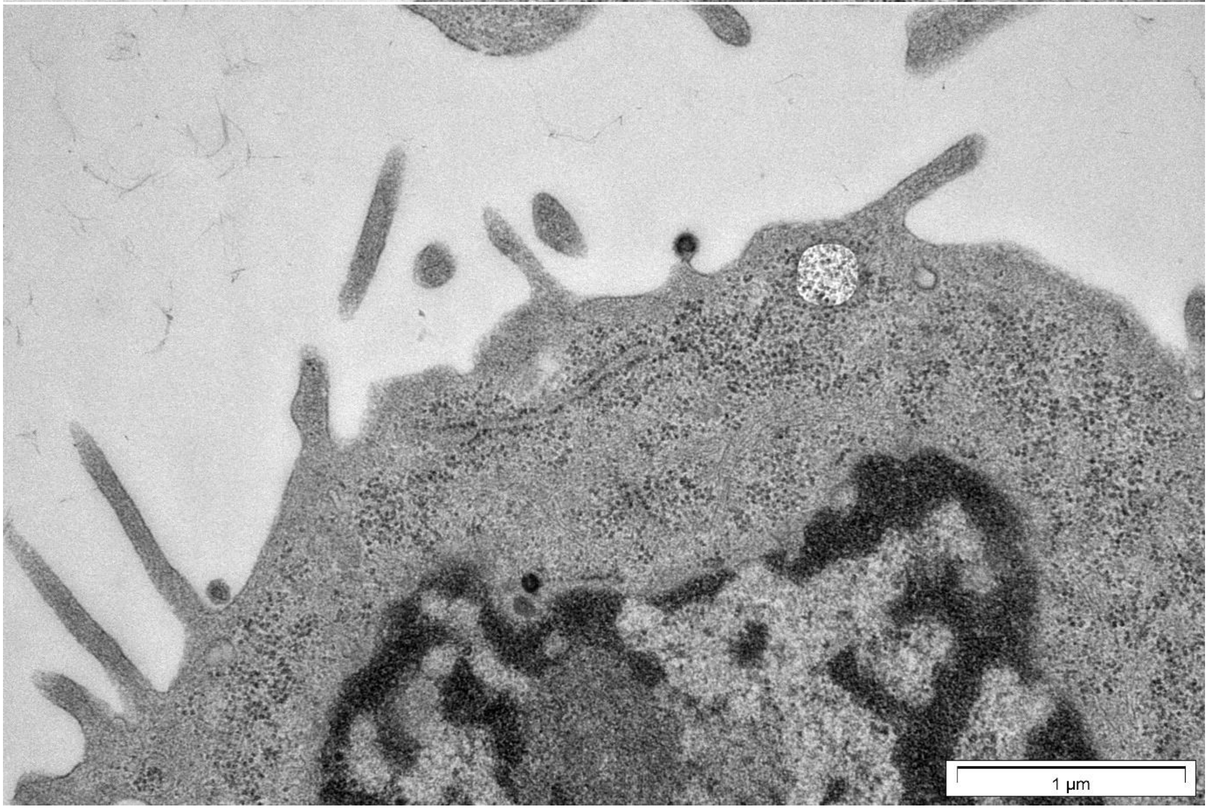
1 µm

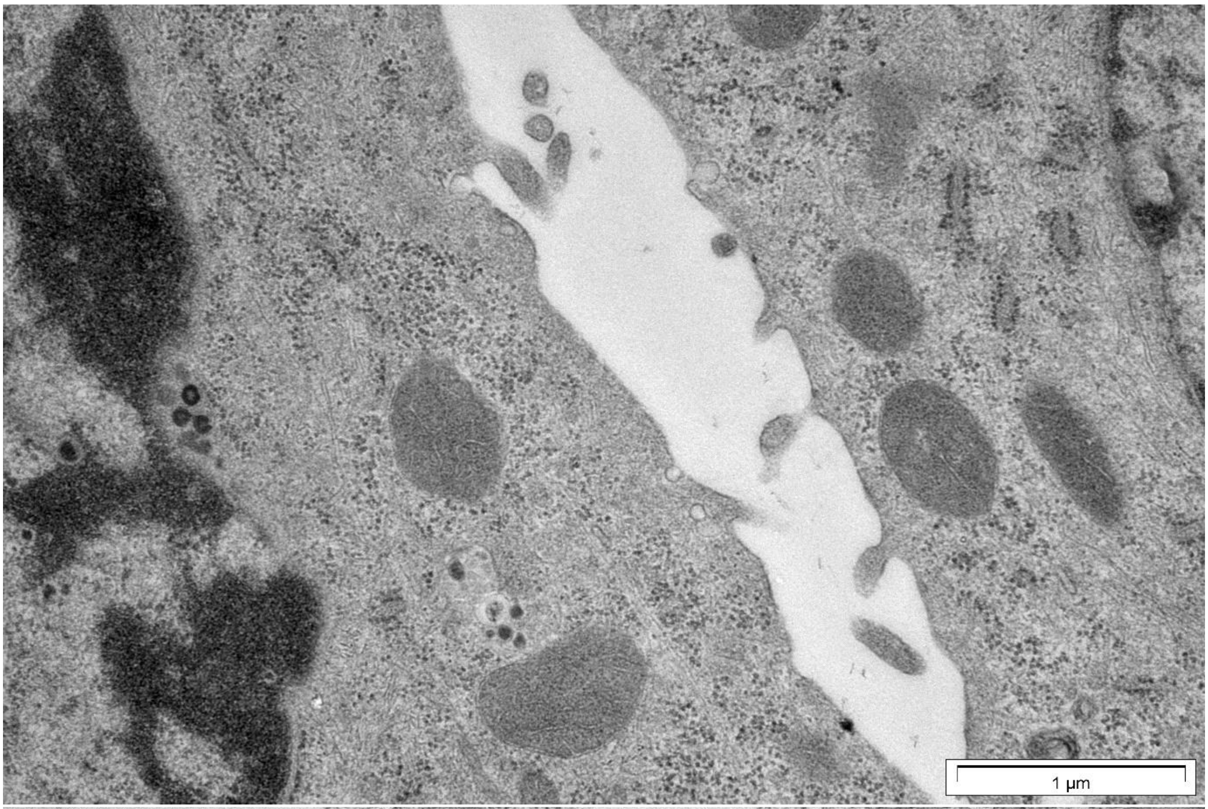
1 µm

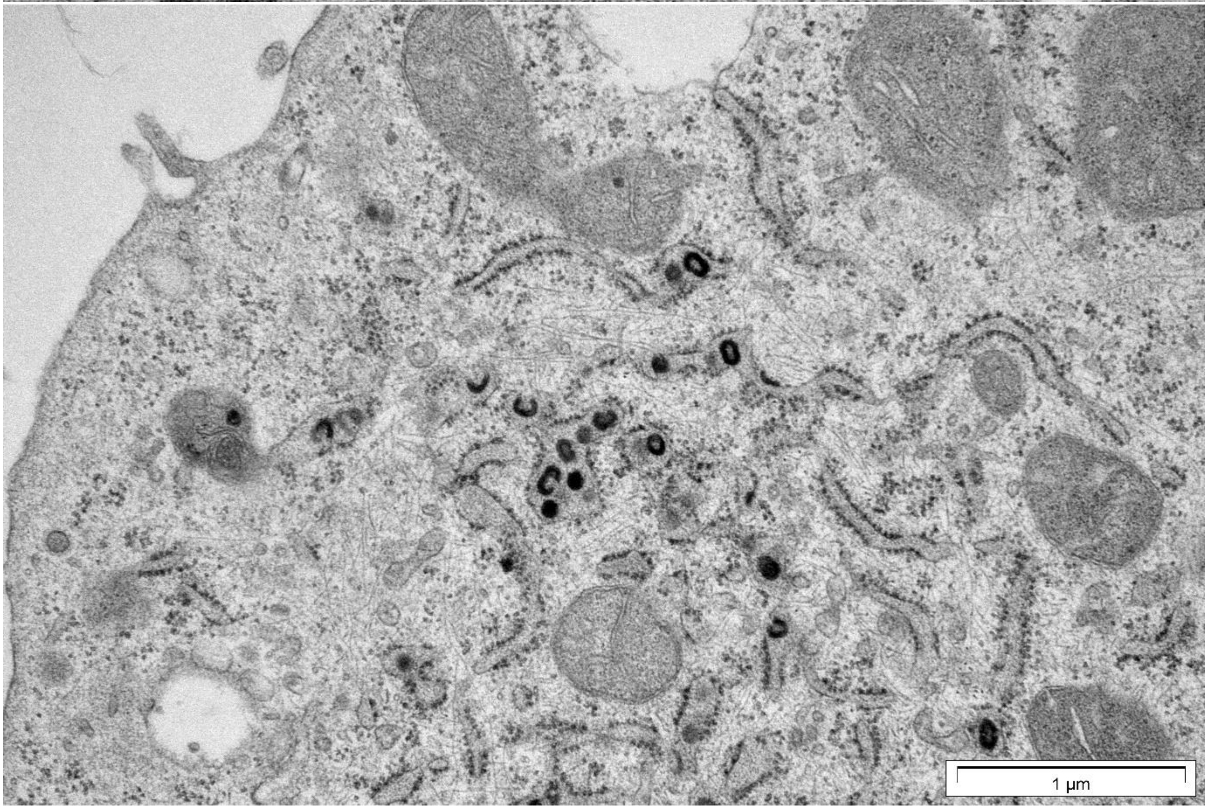
1 µm

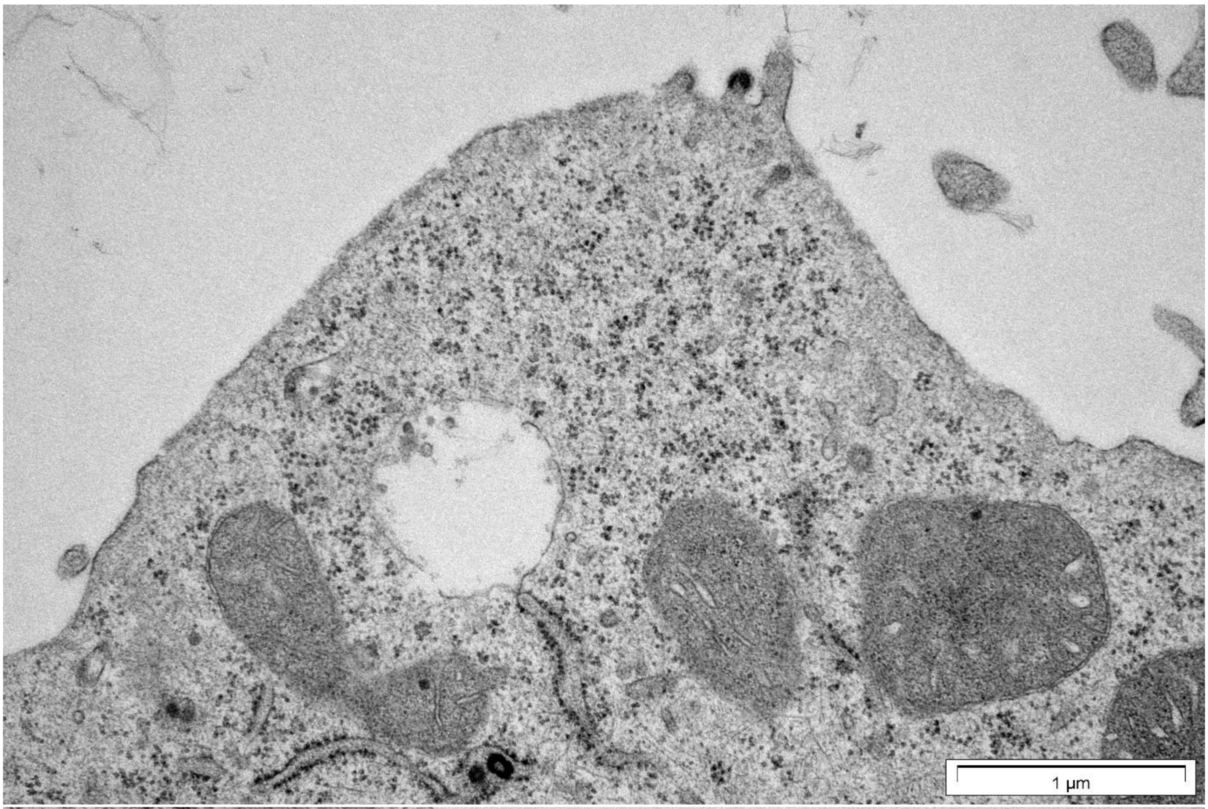
1 µm

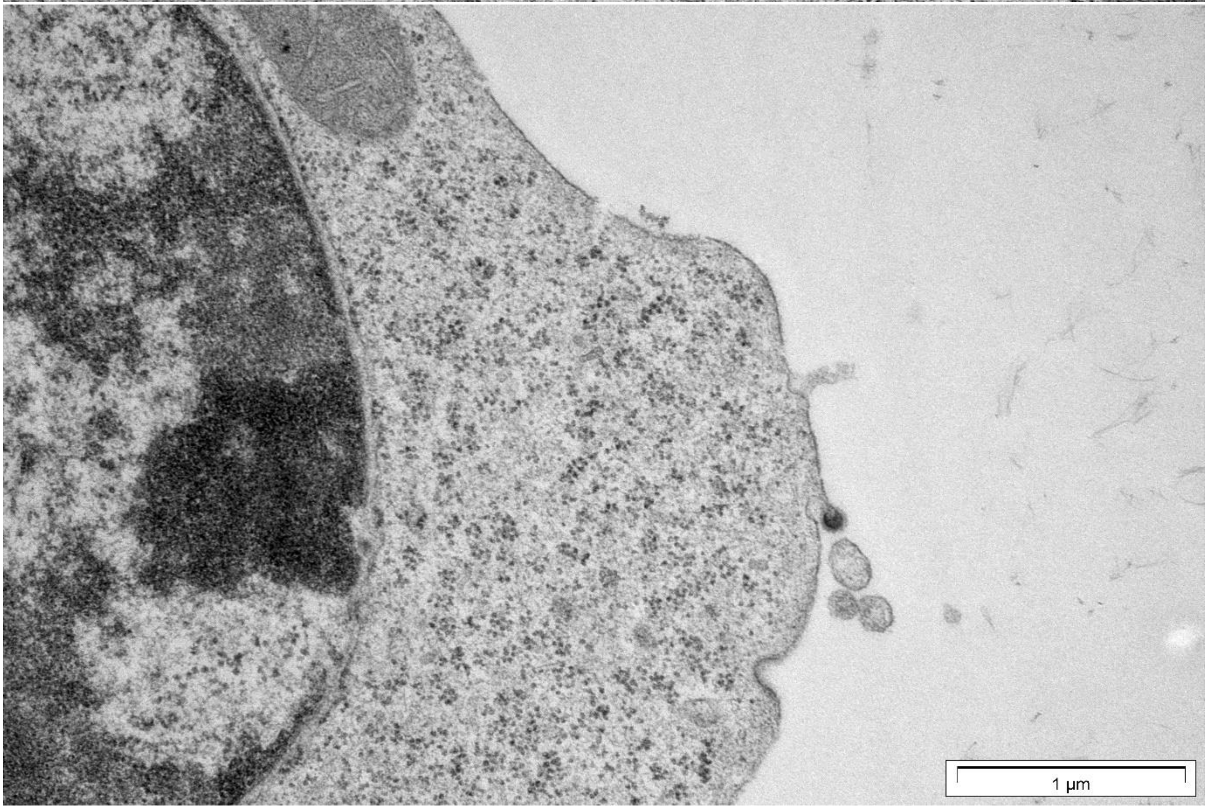
1 µm

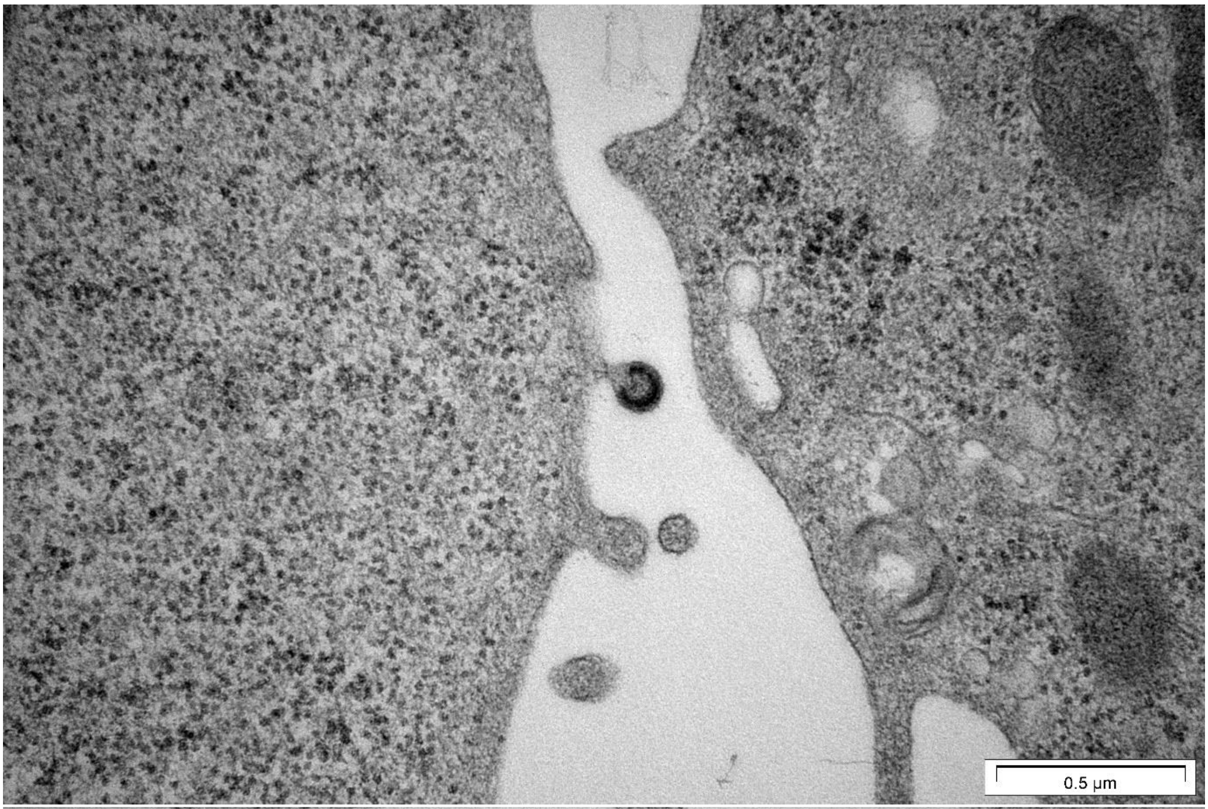
0.5 µm

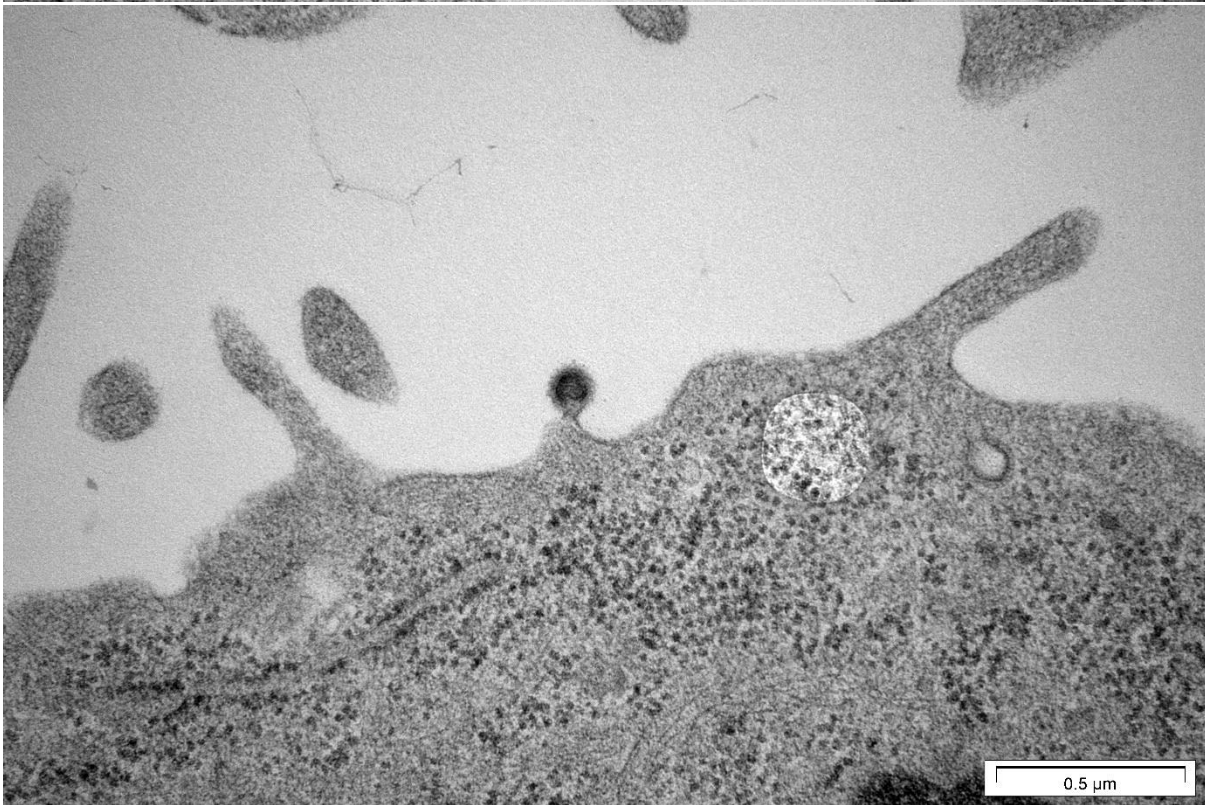
0.5 µm

Dextran sulfate, 15 min.

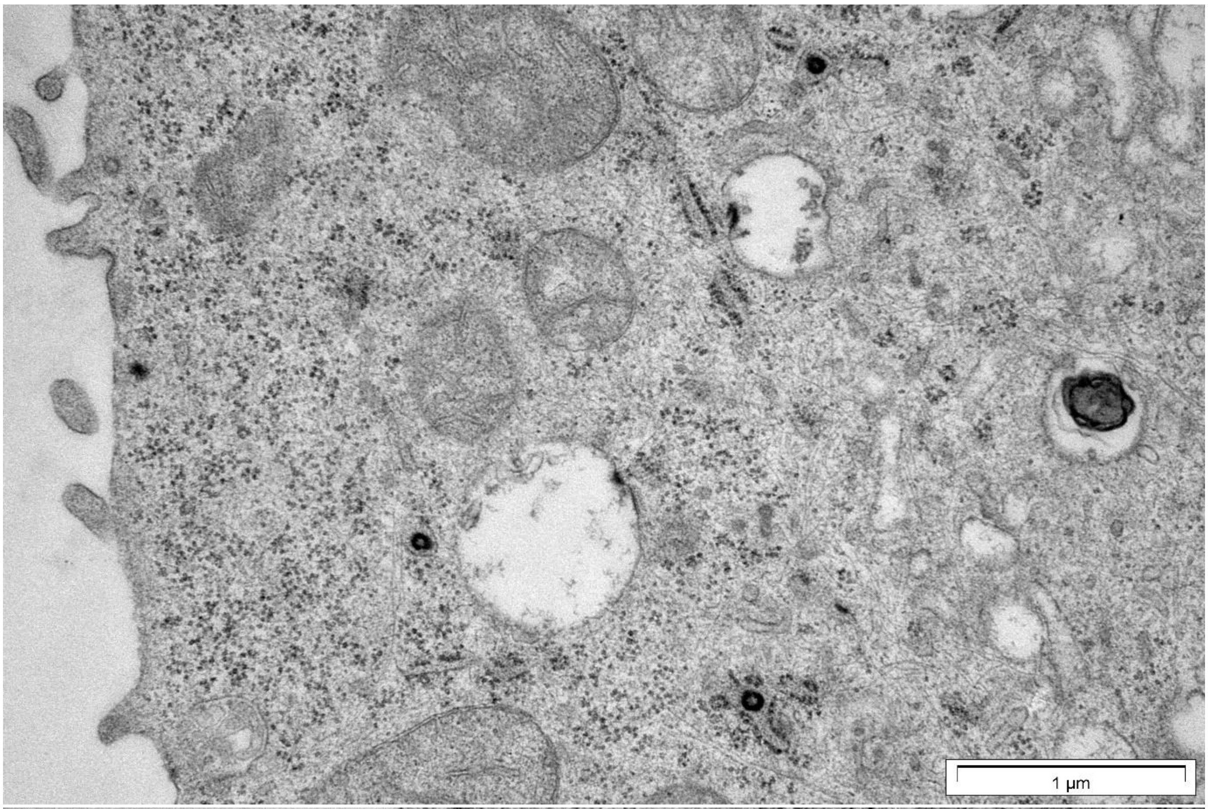

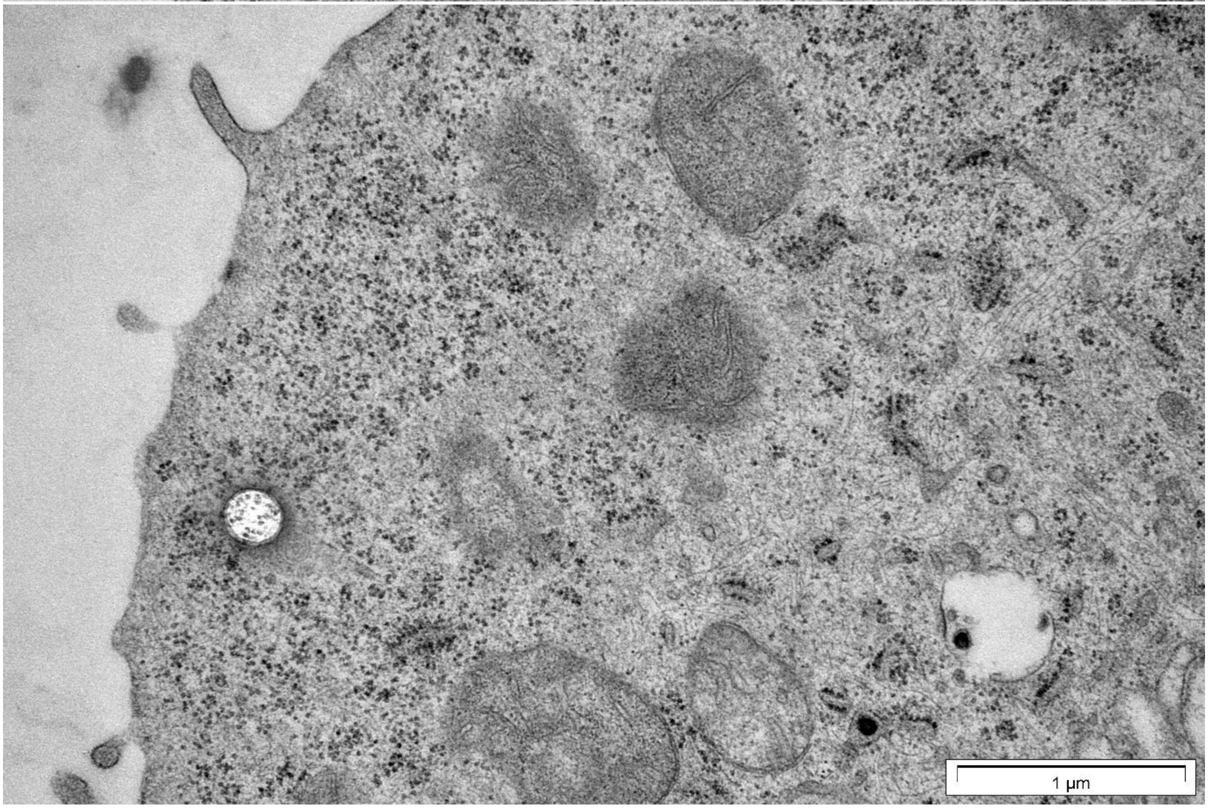

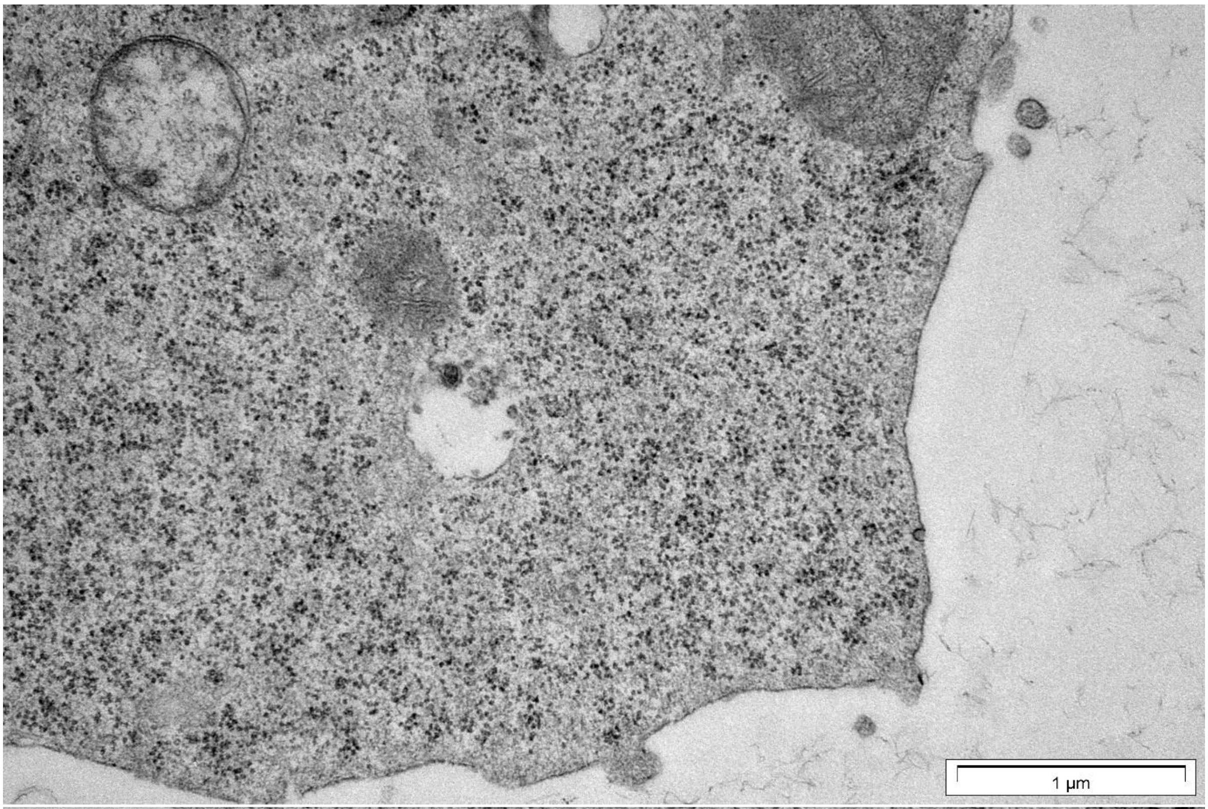
1 µm

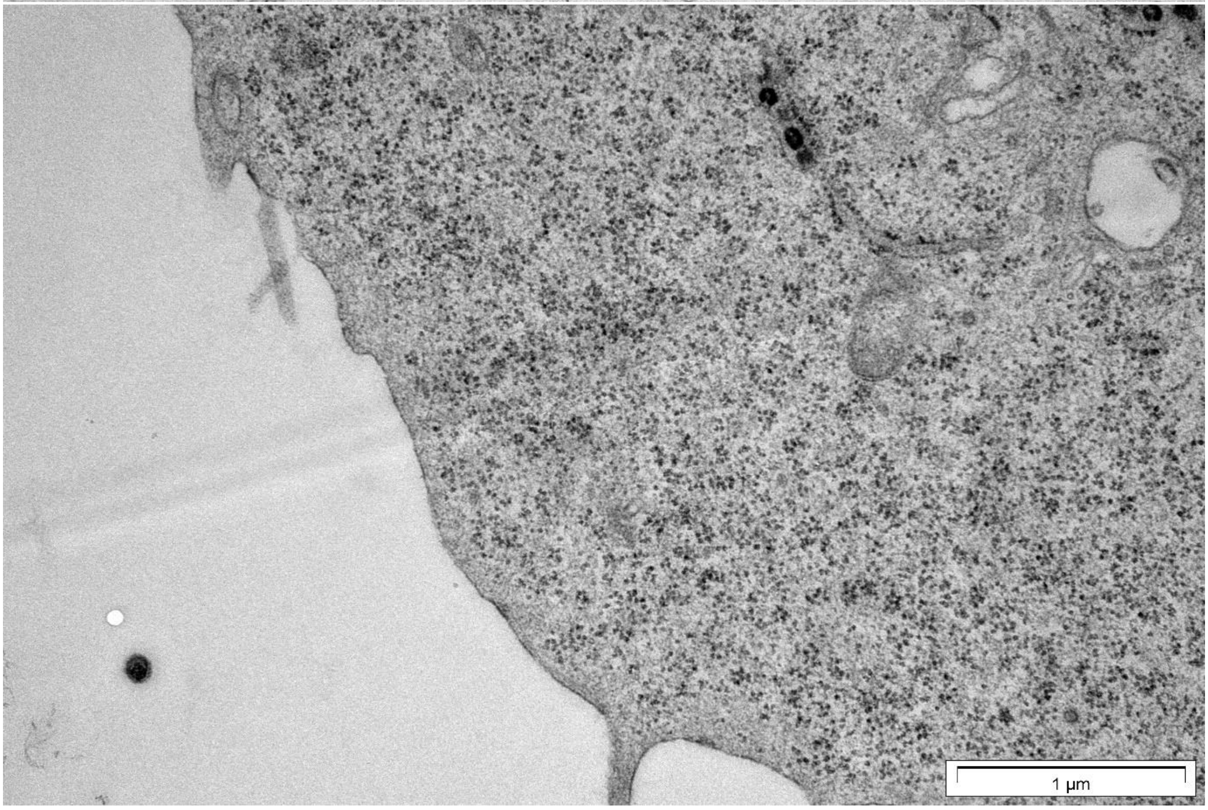
1 µm

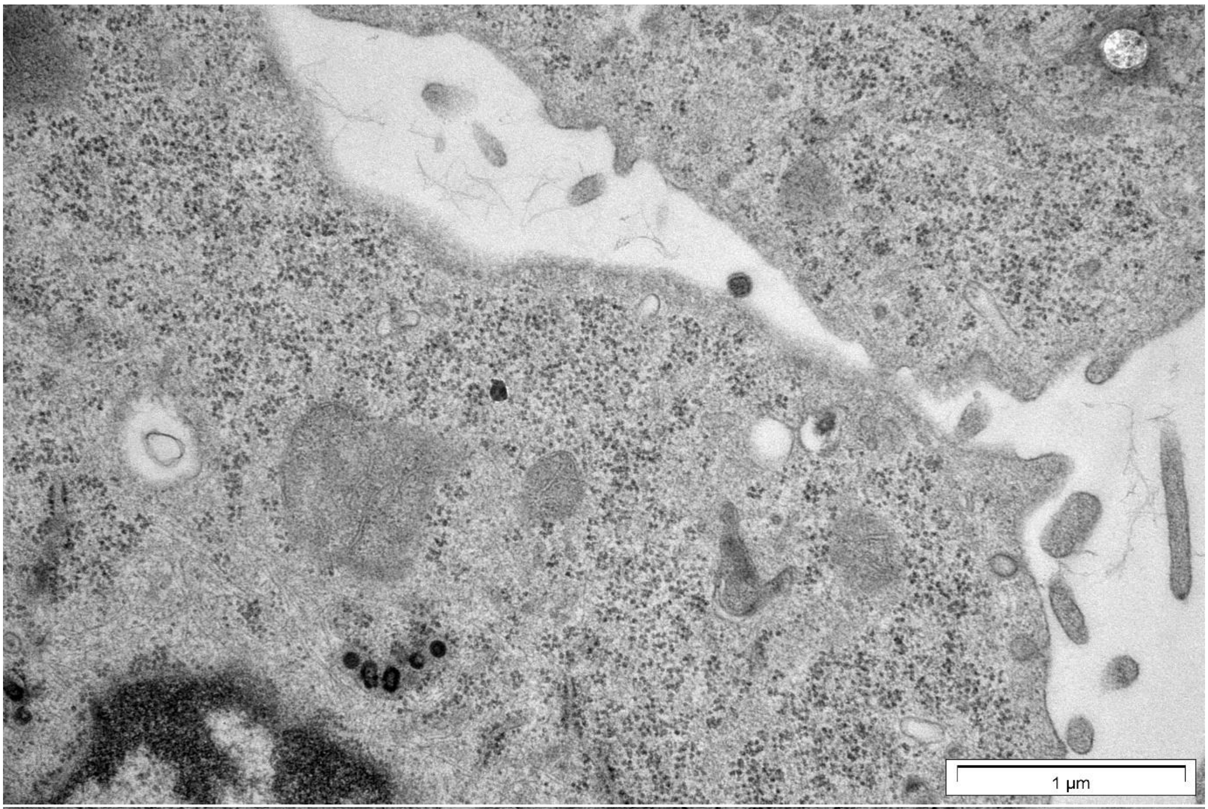
1 µm

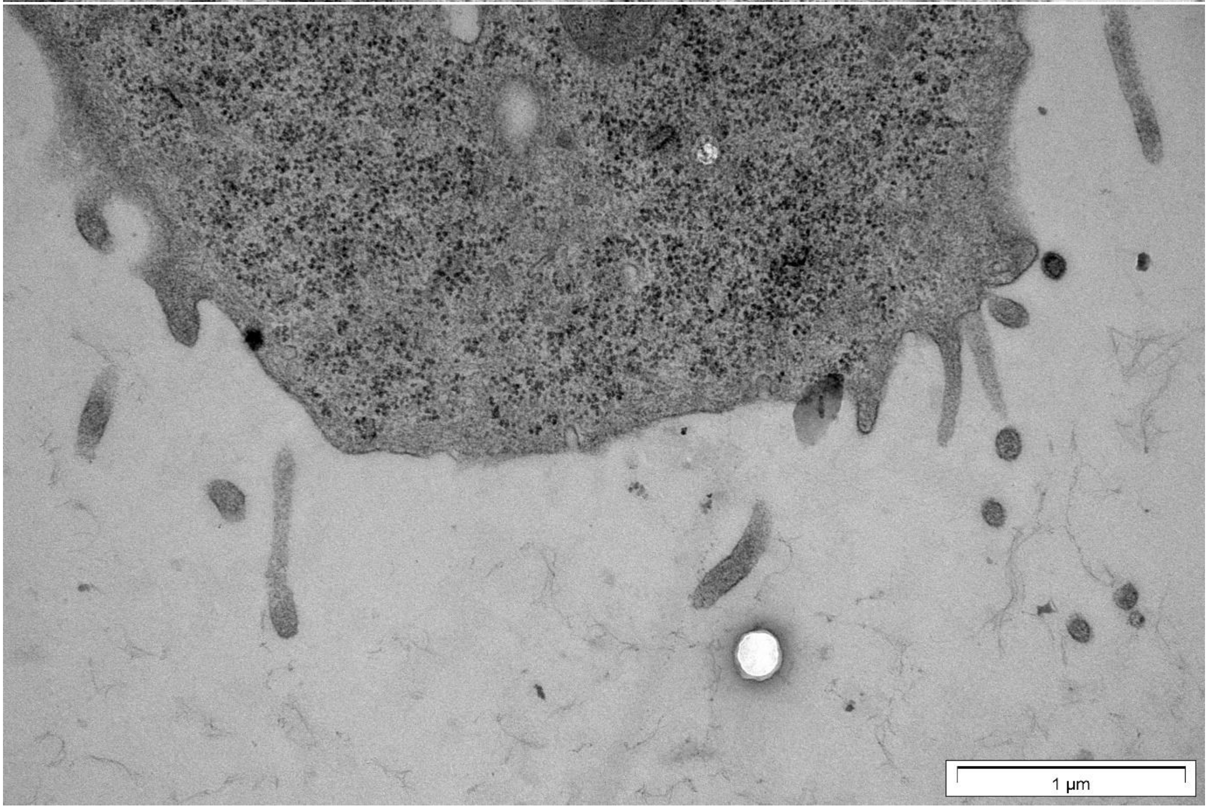
1 µm

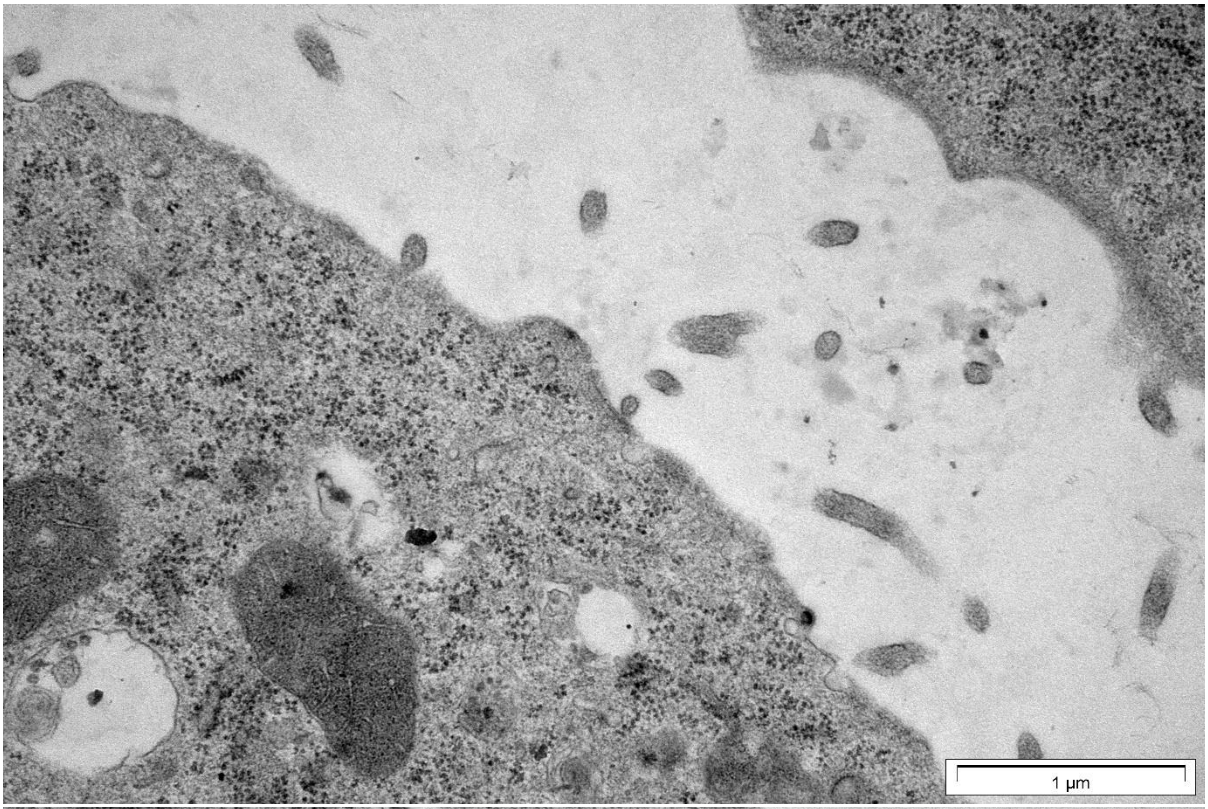
1 µm

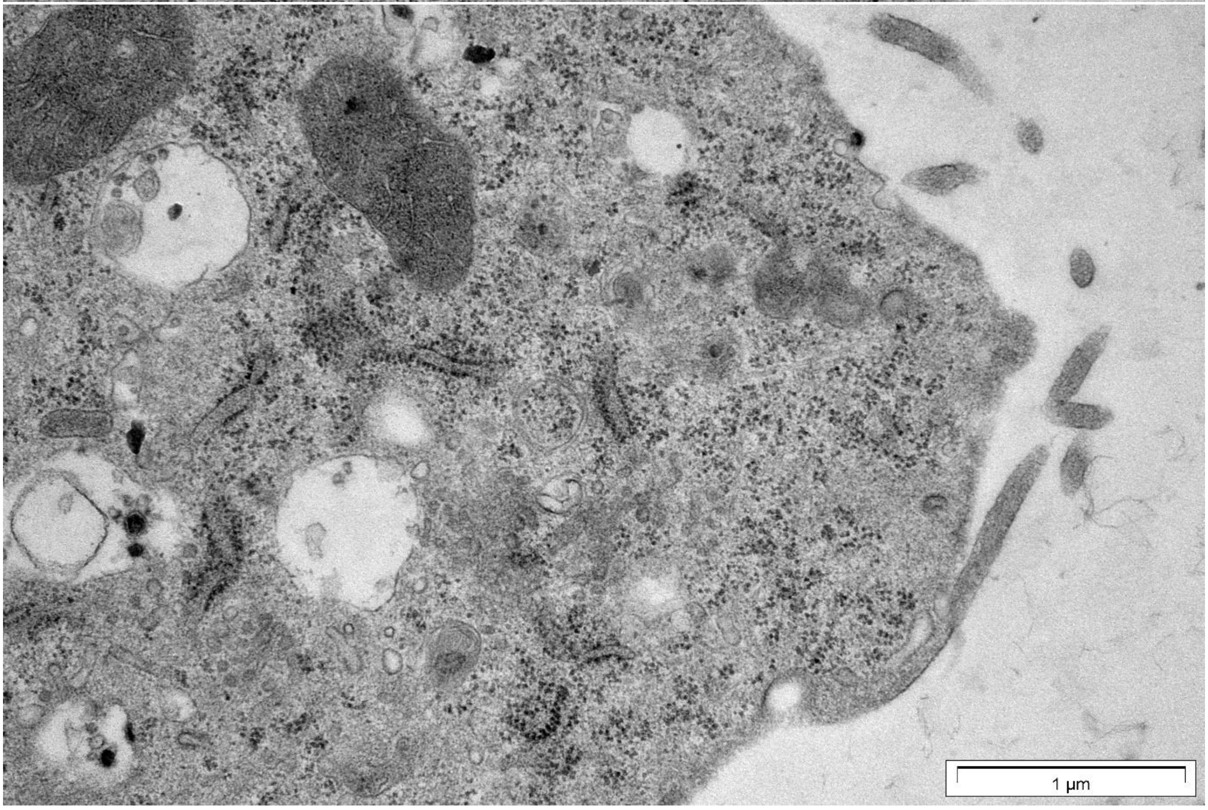
1 µm

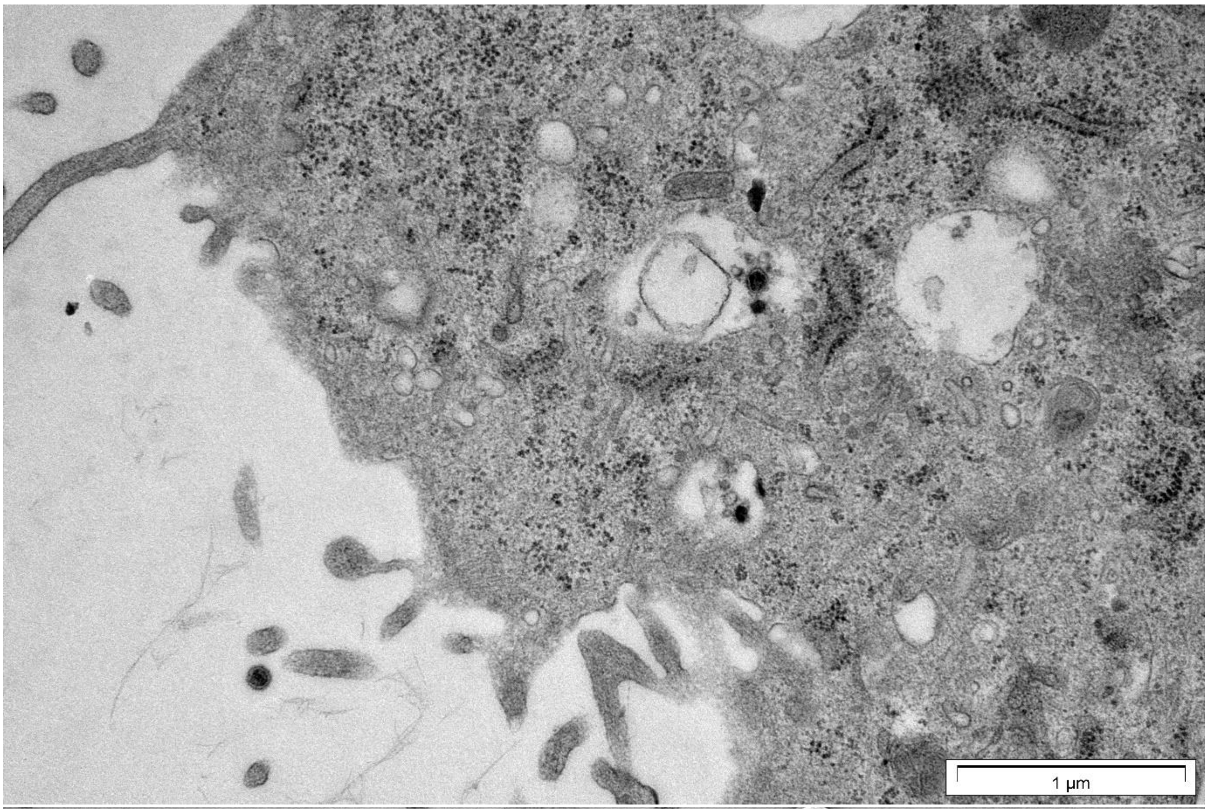
1 µm

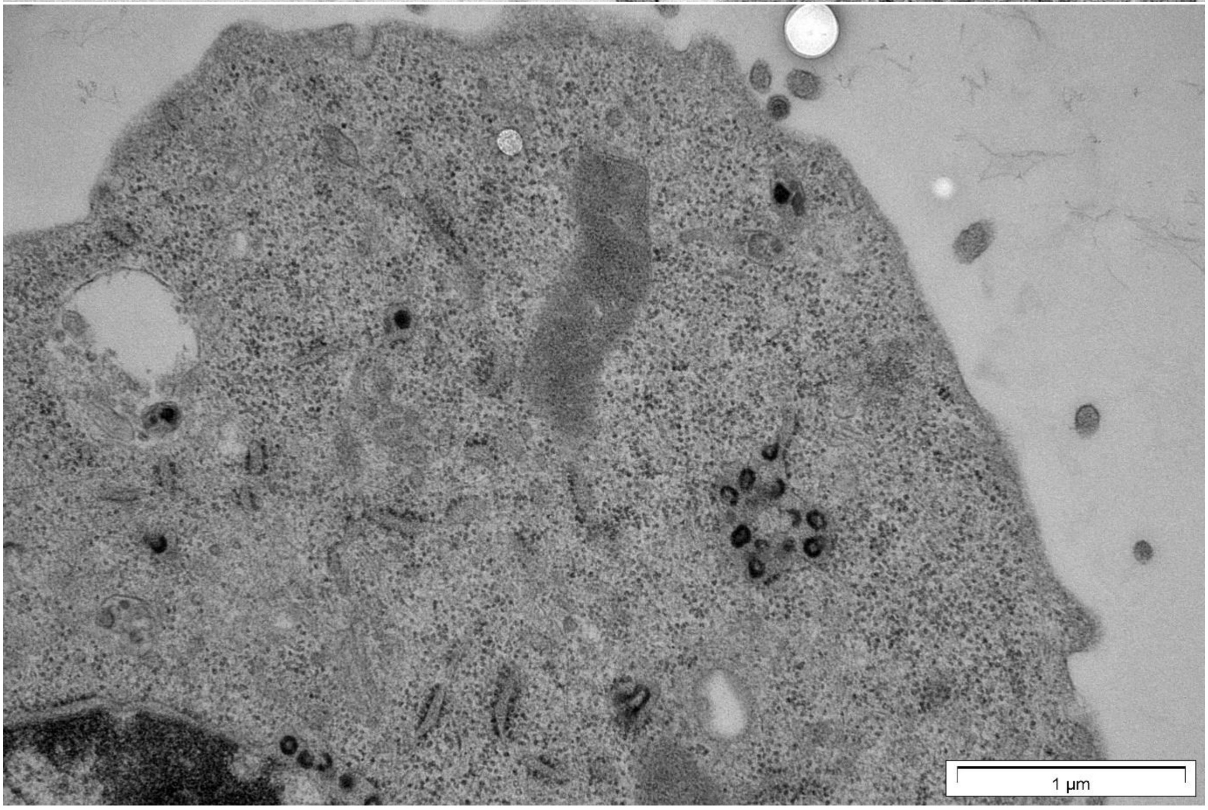
1 µm

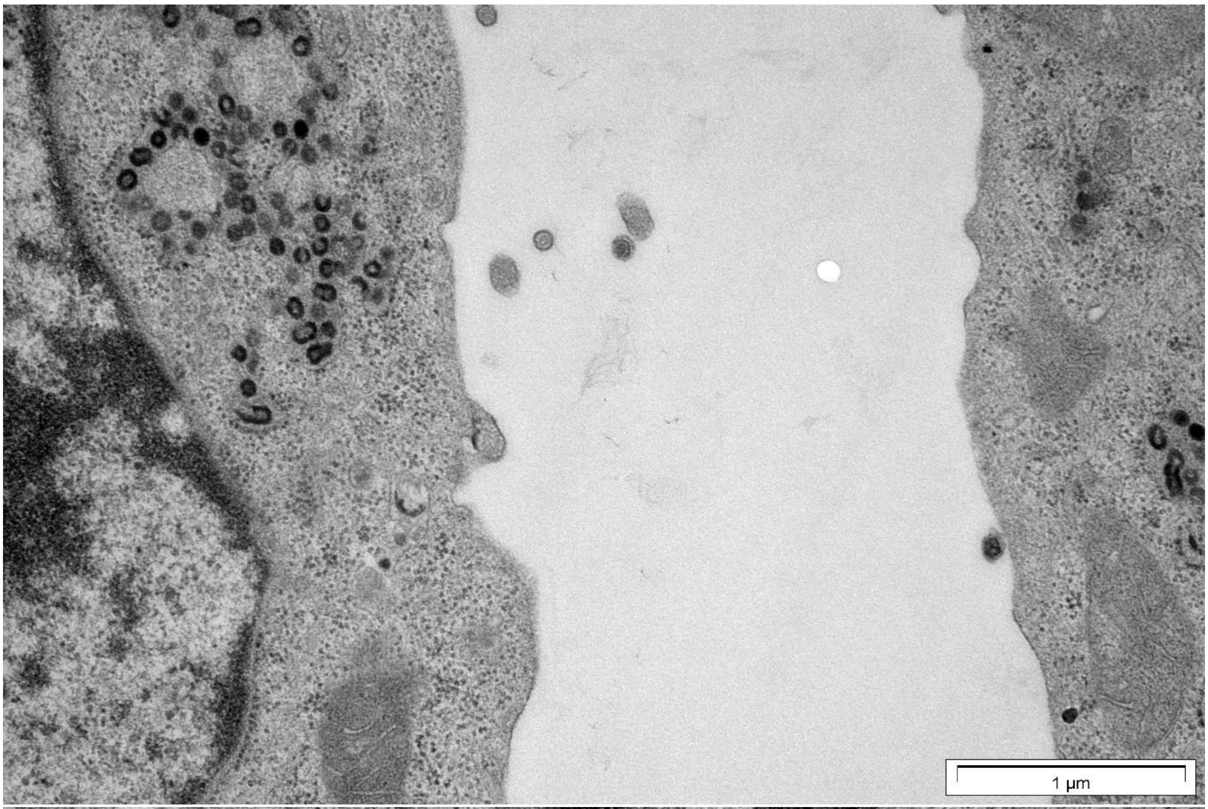
1 µm

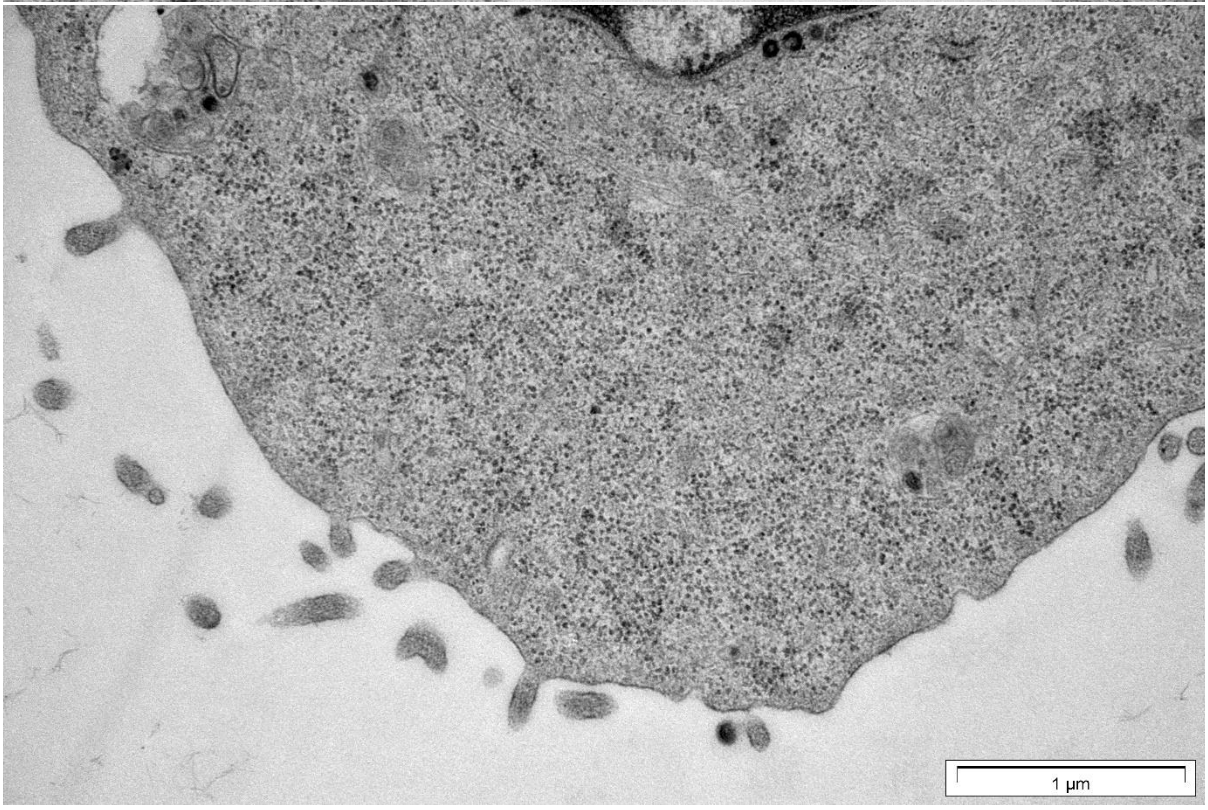
1 µm

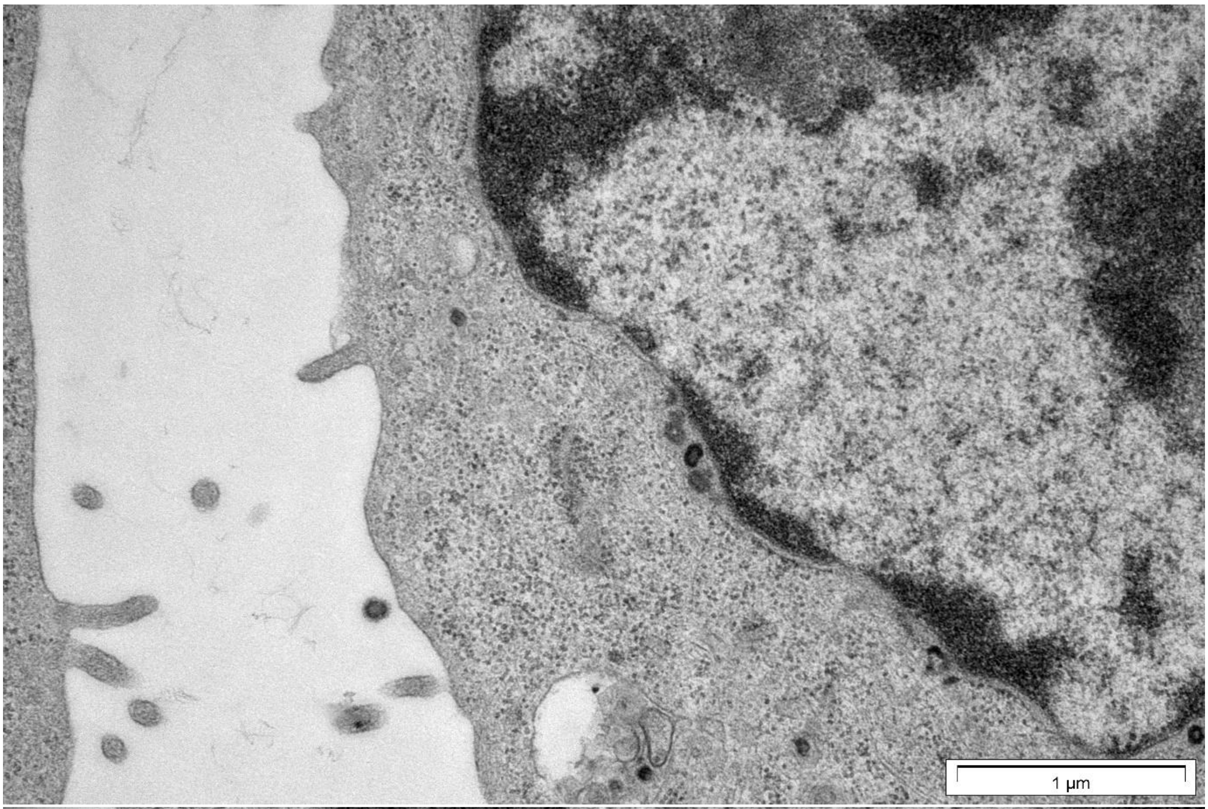
1 µm

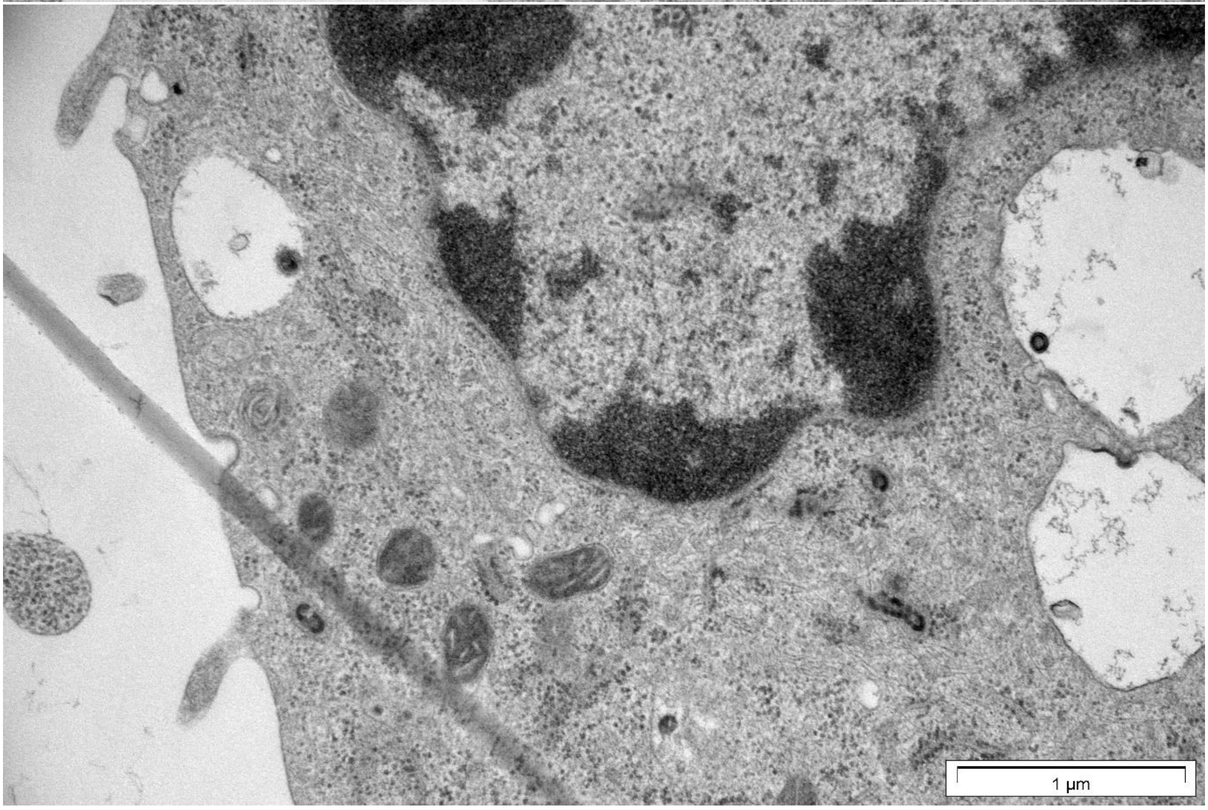
1 µm

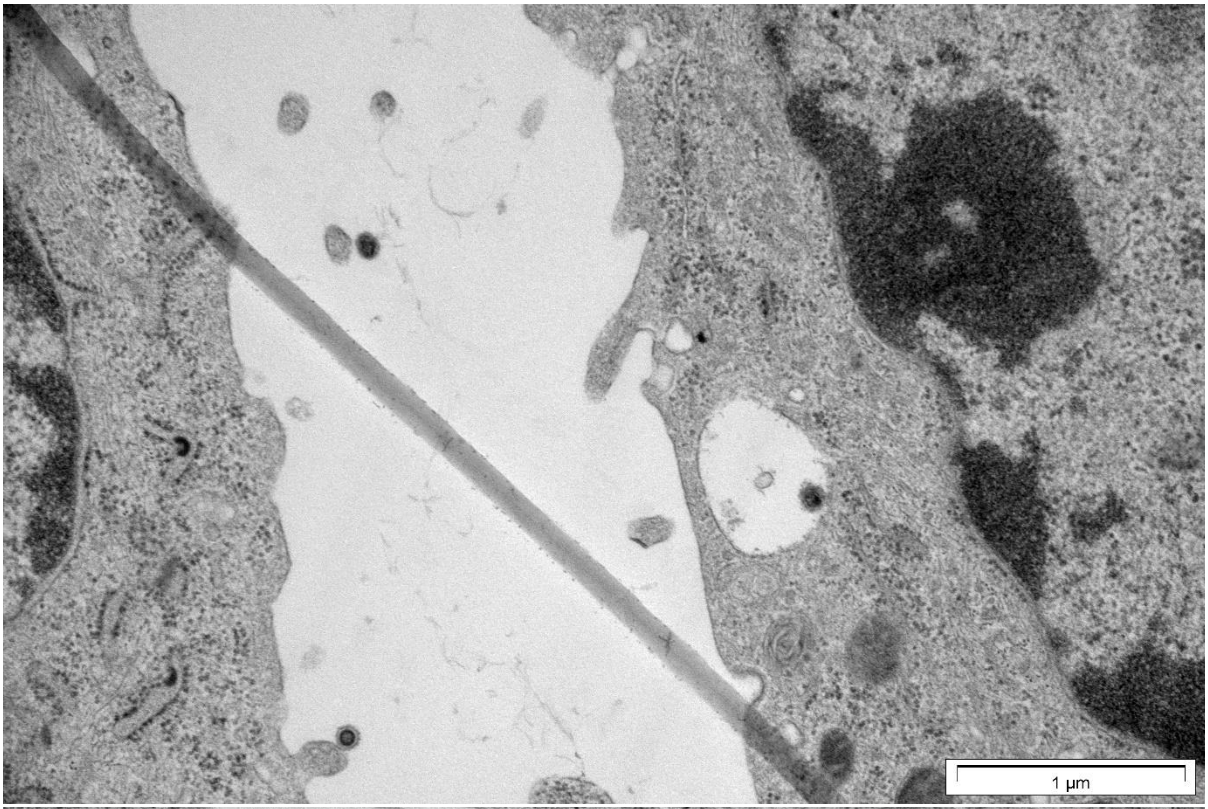
1 µm

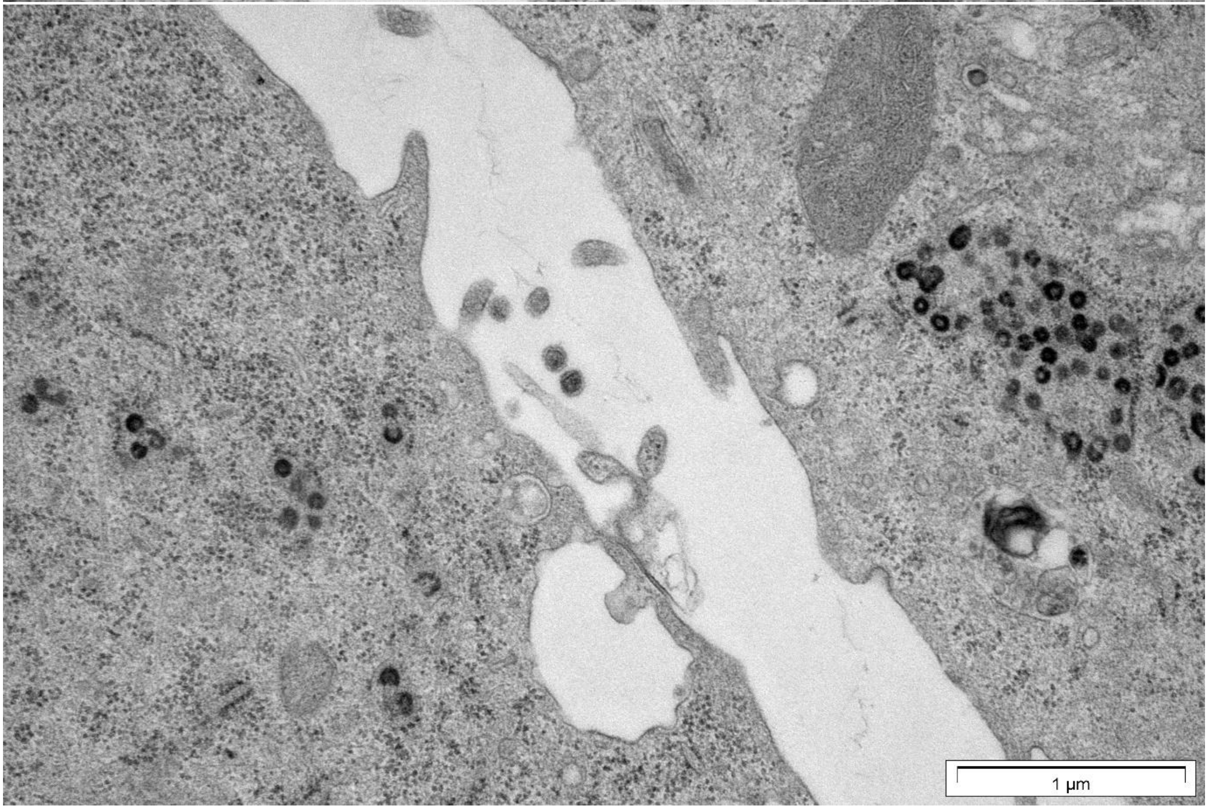
1 µm
