# Supplementary material for: Sulfated glycosaminoglycans inhibit LCMV entry and modulate antiviral immunity and pathology
Source: EMBO Mol Med. 2026 Feb 23;18(4):1235–64. doi: 10.1038/s44321-026-00387-8 (PMC13083911; doi:10.1038/s44321-026-00387-8)

Dextran sulfate experiment -228072025: MC57: Dextran Dextran

150K_D_5_GP_AB_1.czi

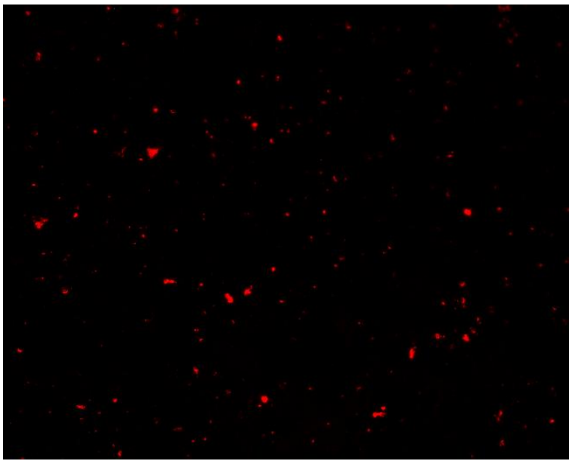

150K_D_5_GP_AB_2.czi

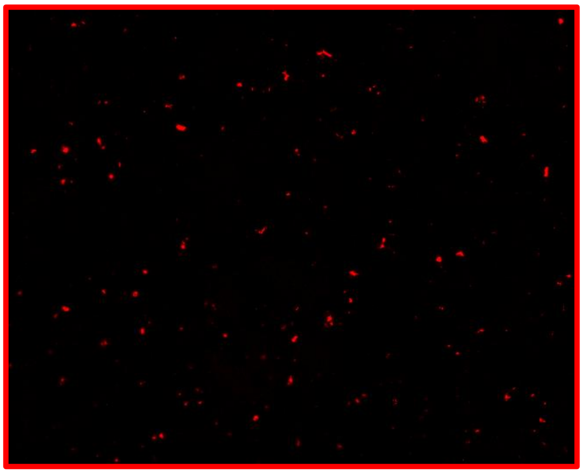

150K_D_5_GP_AB_3.czi

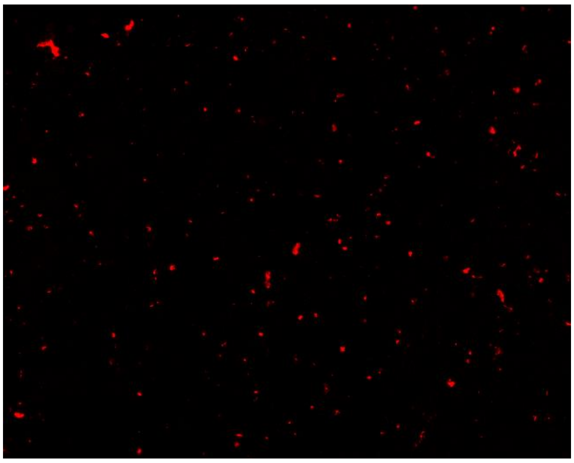

150K_D_5_GP_AB_4.czi

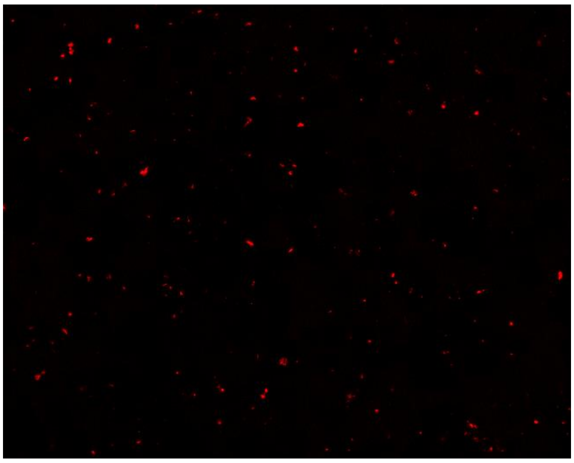

150K_D_50_GP_AB_1.czi

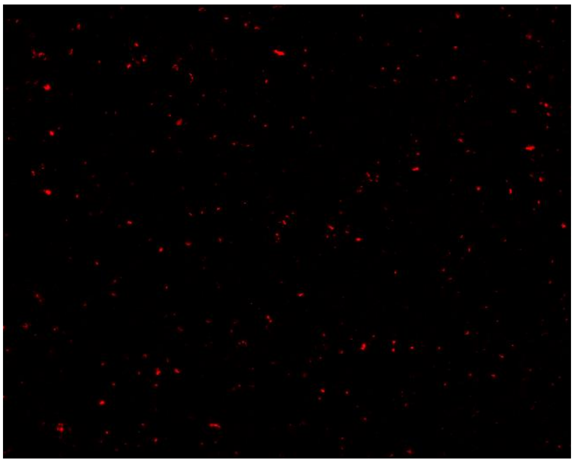

150K_D_50_GP_AB_2.czi

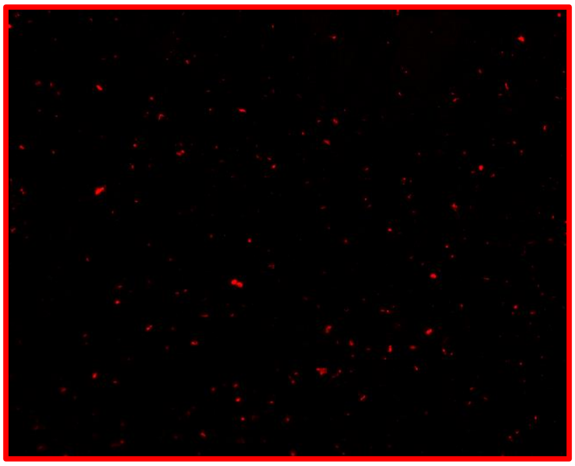

150K_D_50_GP_AB_3.czi

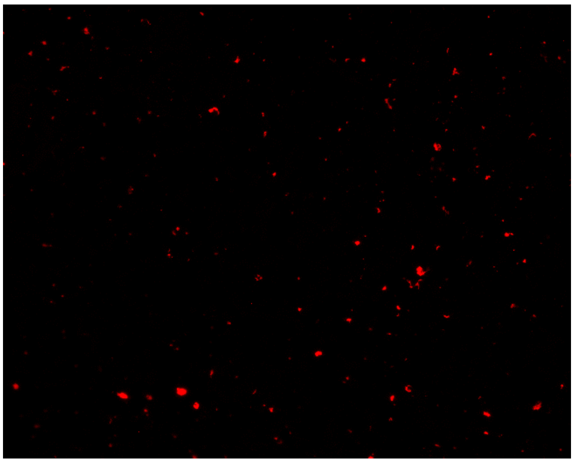

150K_D_50_GP_AB_4.czi

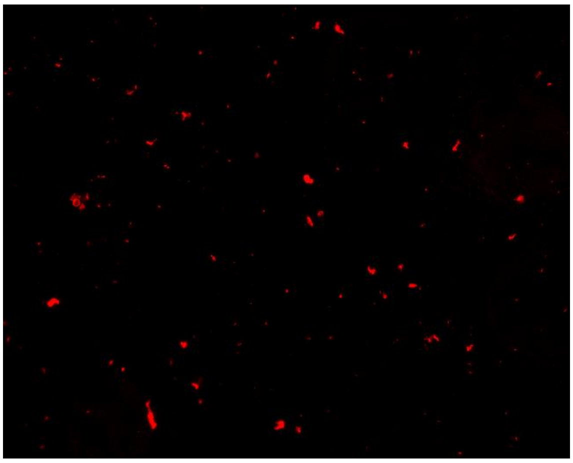

150K_D_500_GP_AB_1.czi

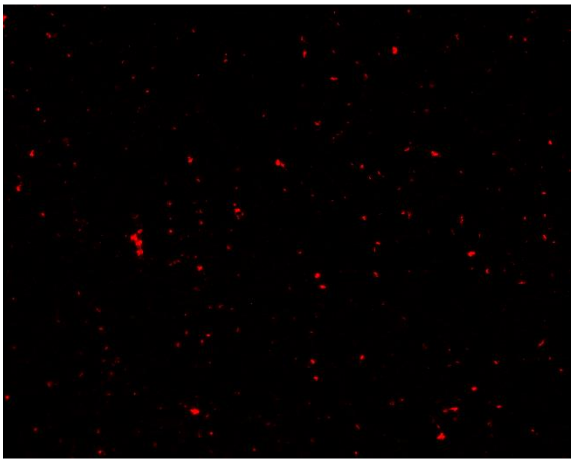

150K_D_500_GP_AB_2.czi

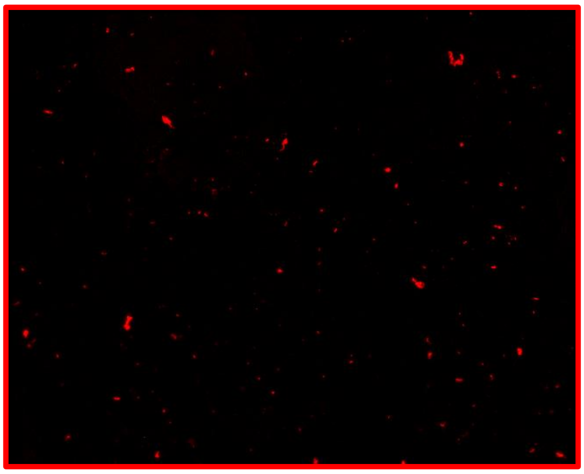

150K_D_500_GP_AB_3.czi

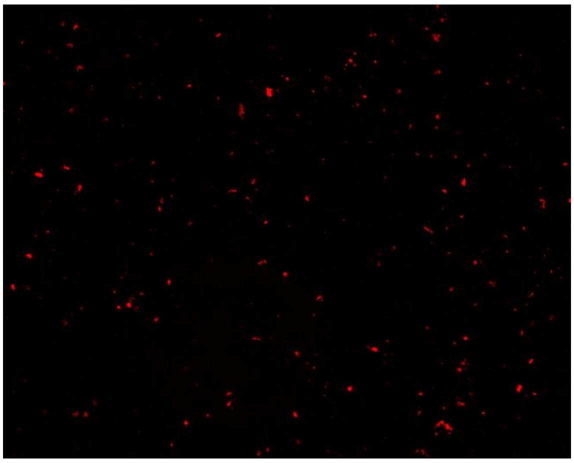

150K_D_500_GP_AB_4.czi

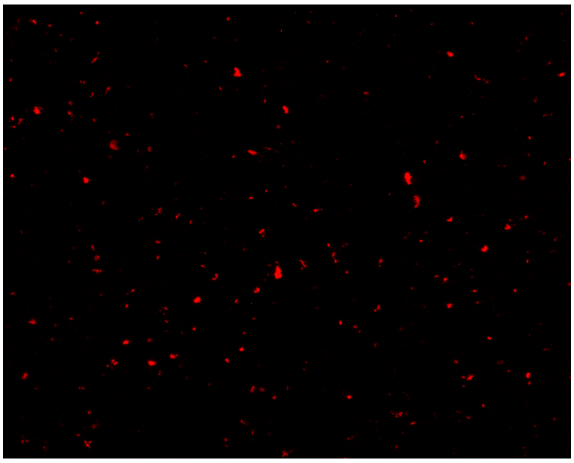

Dextran sulfate experiment -228072025: MC57: Dextran Dextran sulfate

150K_DS_5_GP_AB_1.czi

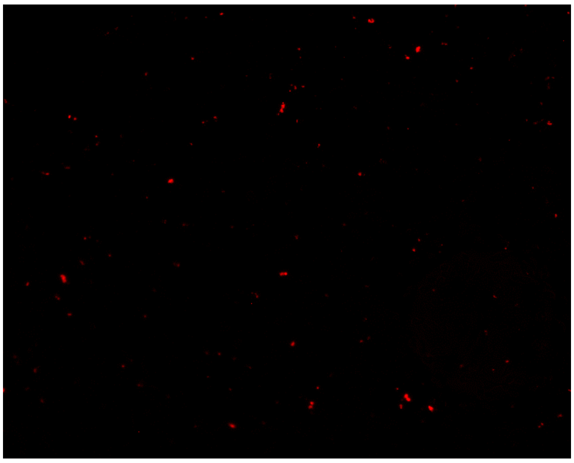

150K_DS_5_GP_AB_2.czi

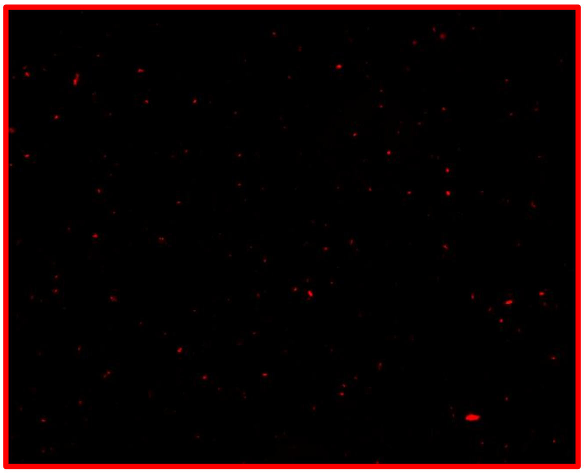

150K_DS_5_GP_AB_3.czi

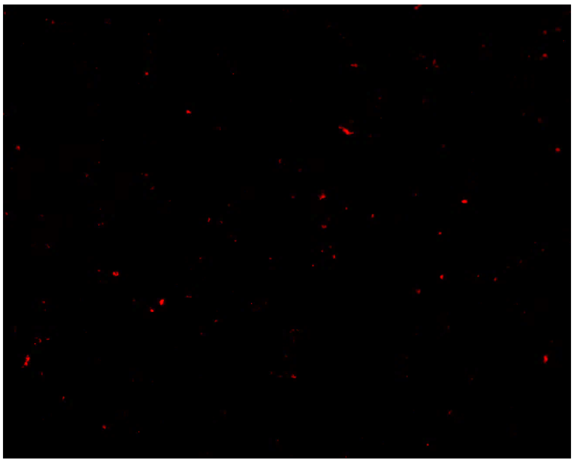

150K_DS_5_GP_AB_4.czi

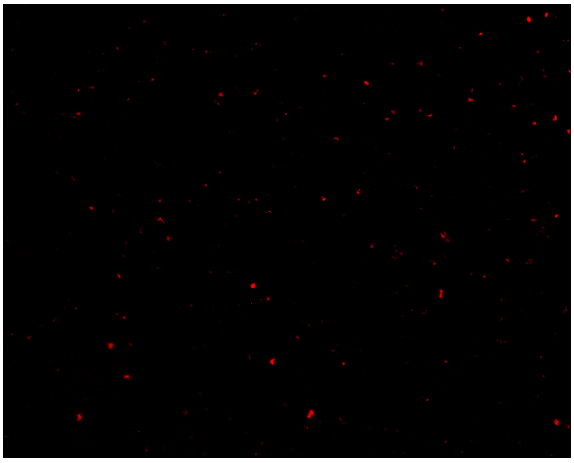

150K_DS_50_GP_AB_1.czi

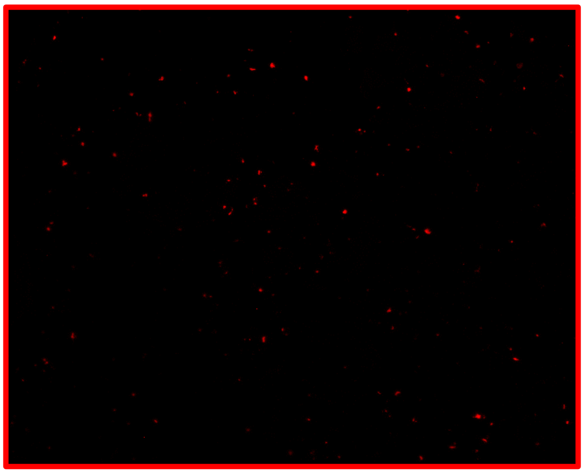

150K_DS_50_GP_AB_2.czi

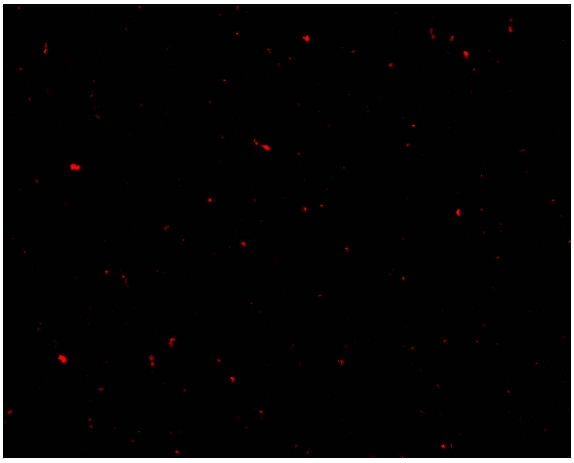

150K_DS_50_GP_AB_3.czi

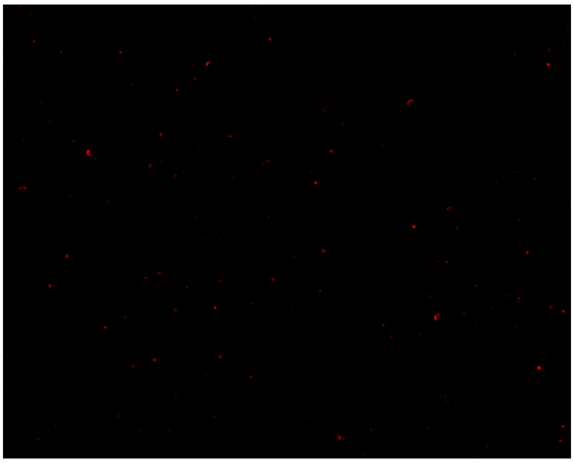

150K_DS_50_GP_AB_4.czi

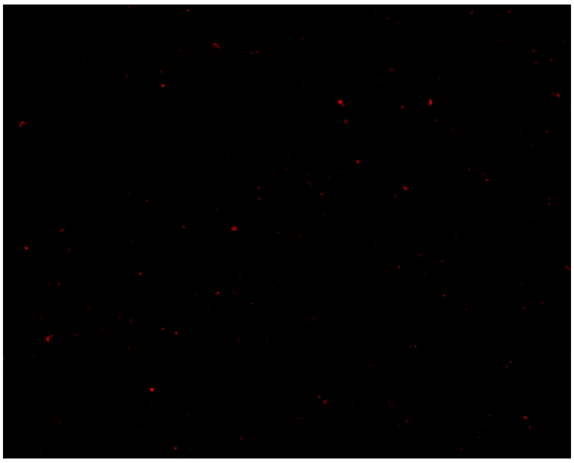

150K_DS_500_GP_AB_1.czi

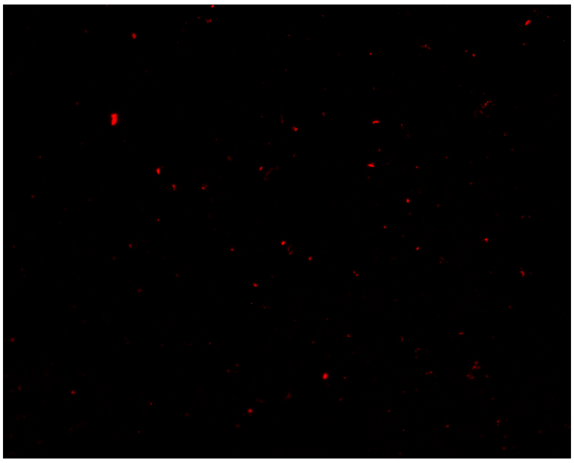

150K_DS_500_GP_AB_2.czi

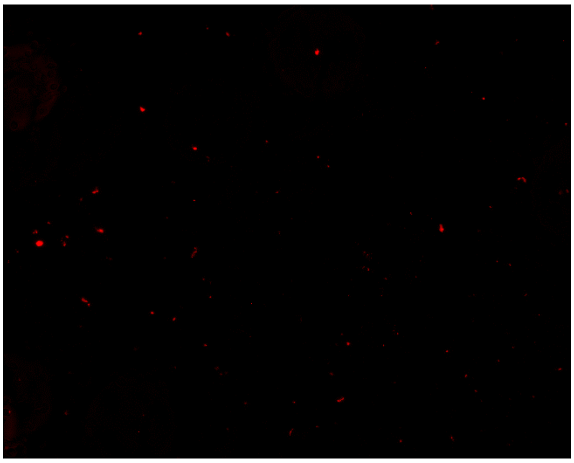

150K_DS_500_GP_AB_3.czi

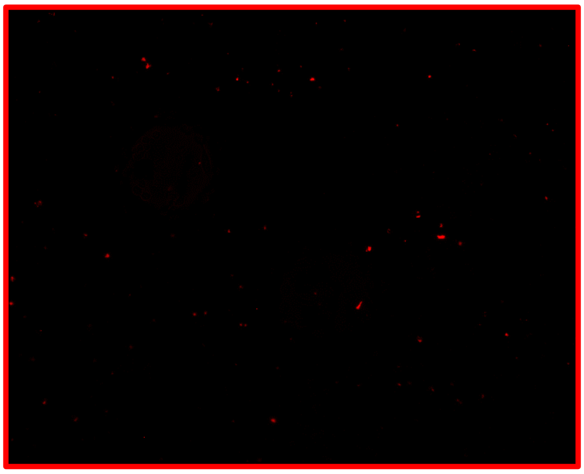

150K_DS_500_GP_AB_4.czi

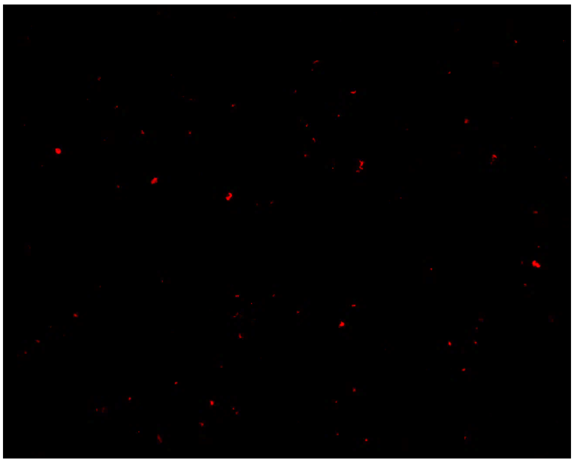

Dextran sulfate experiment -228072025: MC57: Dextran MEDIA

150K_MEDIA_GP_AB_1.czi

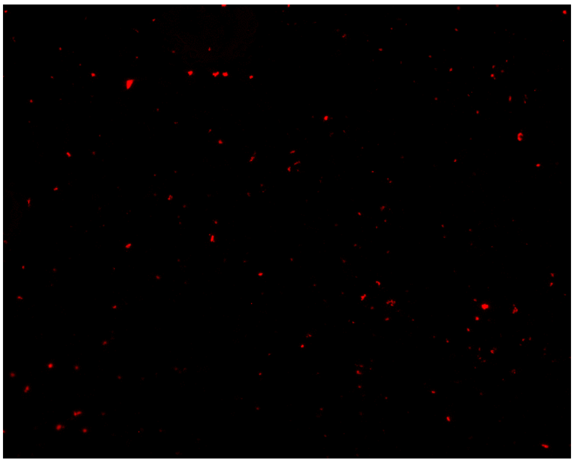

150K_MEDIA_GP_AB_2.czi

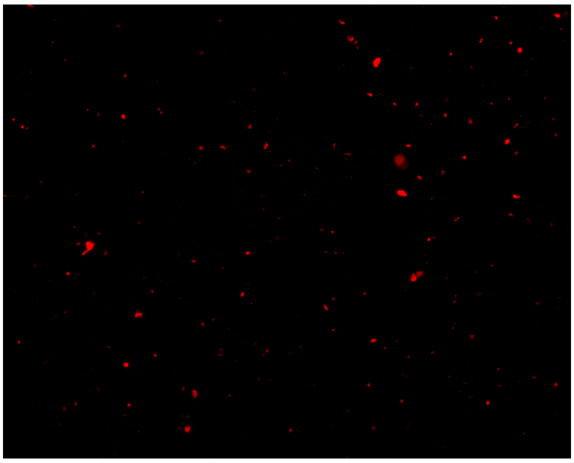

150K_MEDIA_GP_AB_3.czi

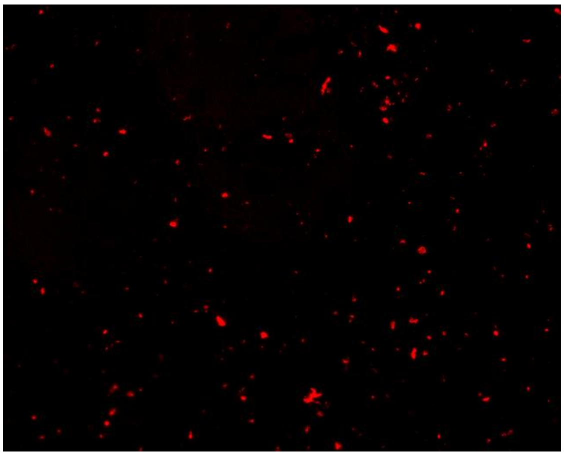

150K_MEDIA_GP_AB_4.czi

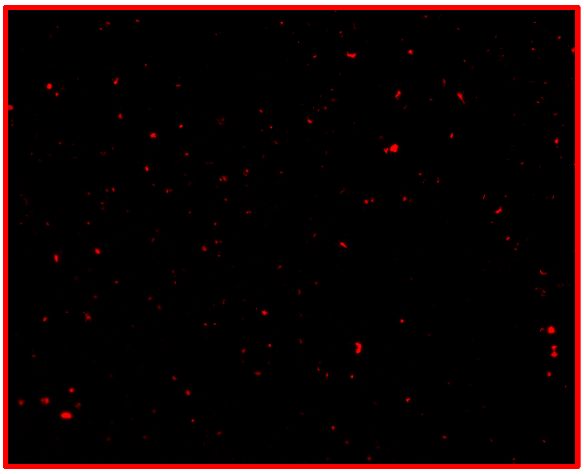

150K_MEDIA_NOGP_AB_1.czi

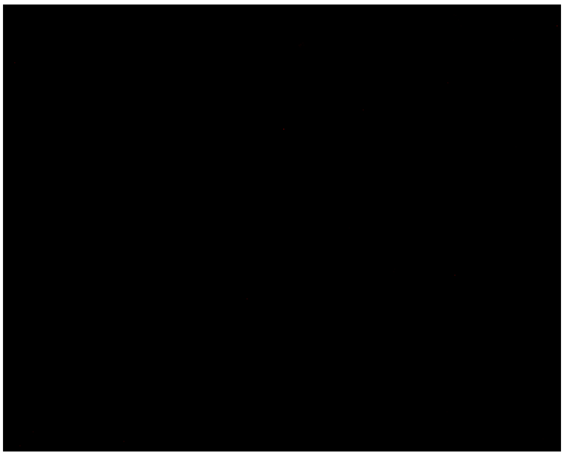

150K_MEDIA_NOGP_AB_2.czi

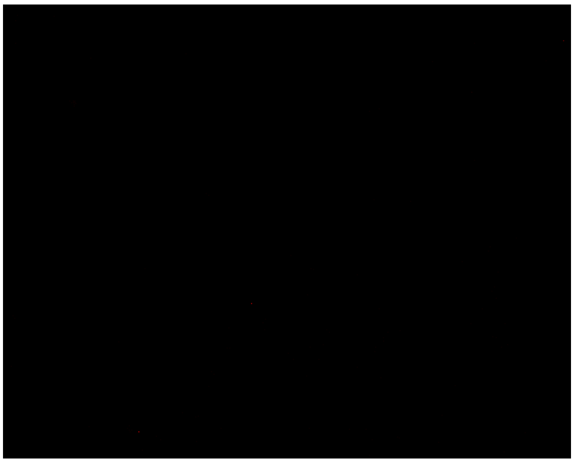

Supplement: Supplementary file 5 — Source data Fig. 3 [file 44321_2026_387_MOESM5_ESM.zip › Fig. 3/Fig. 3F/Summary.pdf]
