# Supplementary material for: Sulfated glycosaminoglycans inhibit LCMV entry and modulate antiviral immunity and pathology
Source: EMBO Mol Med. 2026 Feb 23;18(4):1235–64. doi: 10.1038/s44321-026-00387-8 (PMC13083911; doi:10.1038/s44321-026-00387-8)

D1 Vehicle R Sp 20X and rep. images 10X N=4, 2 ROI

DAPI CD169 LCMVNP F4/80

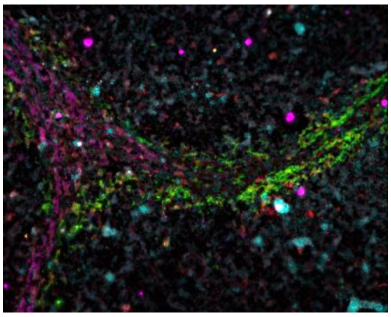

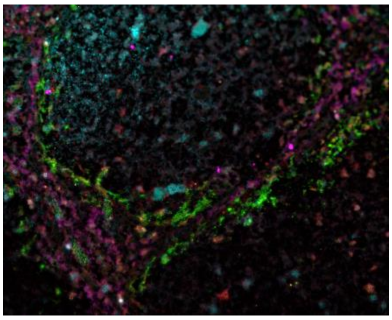

NN Sp

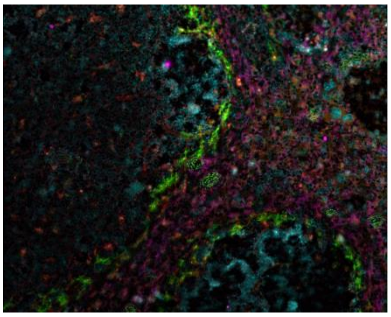

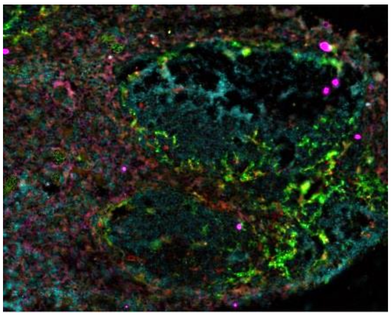

LL Sp

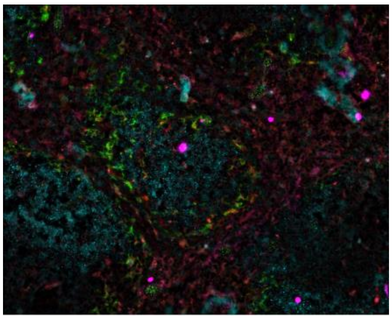

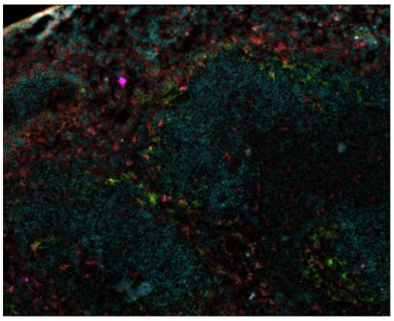

L Sp

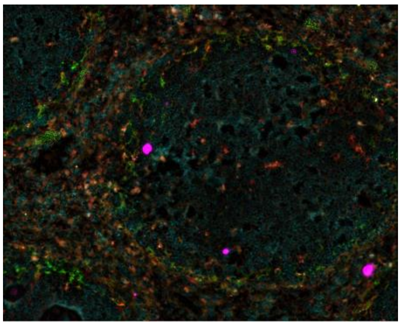

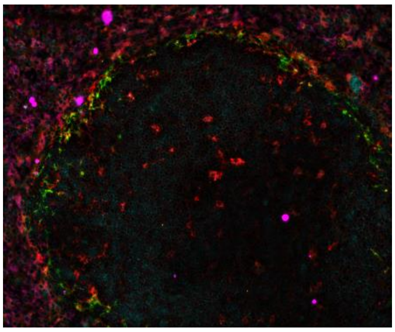

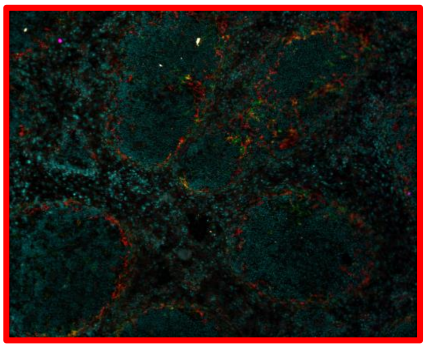

D1 +Dextran sulphate; 20X and rep. images 10X N=4, 2 ROI

L ds Sp

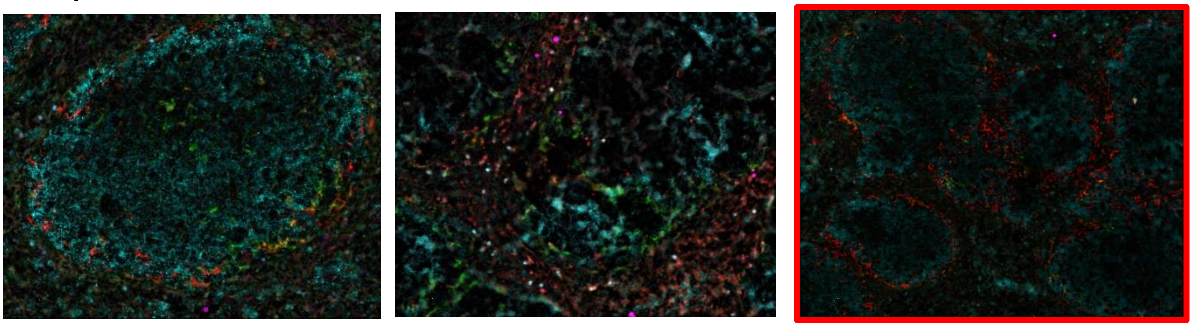

NN ds Sp

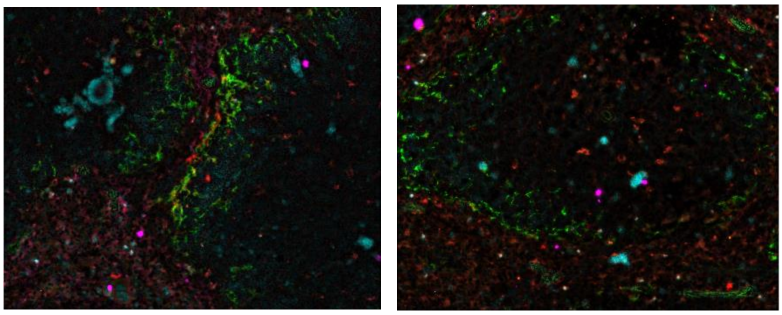

LL ds Sp

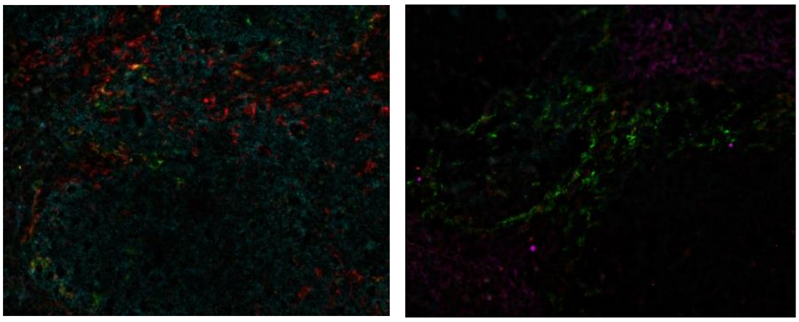

R ds Sp

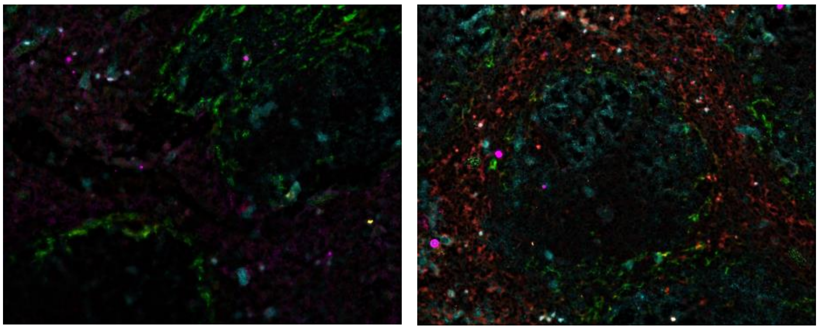

Supplement: Supplementary file 7 — Source data Fig. 5 [file 44321_2026_387_MOESM7_ESM.zip › Fig. 5/Fig. 5C/Fig. 5C_all.pdf]
