# Supplementary material for: Sulfated glycosaminoglycans inhibit LCMV entry and modulate antiviral immunity and pathology
Source: EMBO Mol Med. 2026 Feb 23;18(4):1235–64. doi: 10.1038/s44321-026-00387-8 (PMC13083911; doi:10.1038/s44321-026-00387-8)
Supplement: Supplementary file 8 — Source data Fig. 6 [file 44321_2026_387_MOESM8_ESM.zip › Fig. 6/Fig. 6C-D/Fig. 6C.pdf]

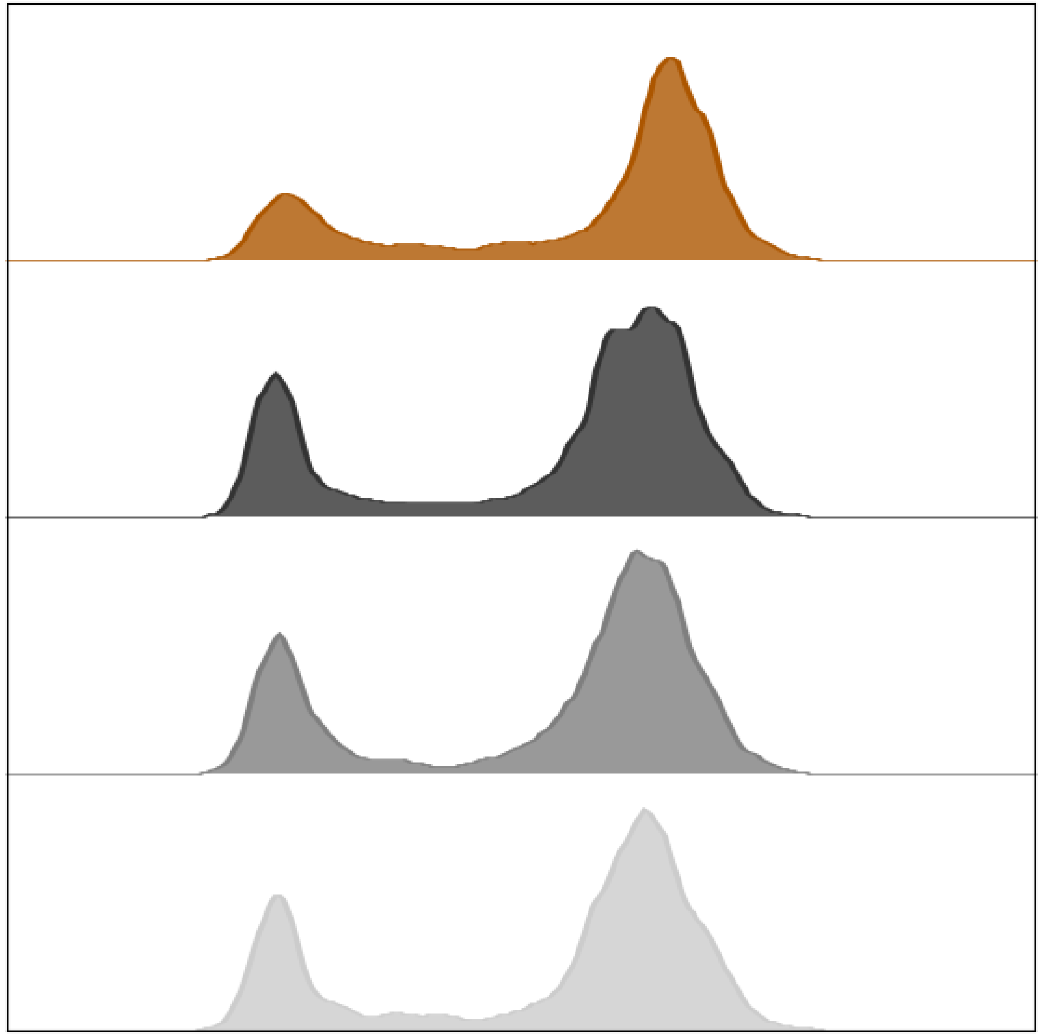

	Sample Name	Mean : Comp-Alexa Fluor 700-A
	mouse_001_1,3a,10_LCMV_001.fcs	6542
	mouse_001_1,3a,10_Dextran_005_007.fcs	5180
	mouse_001_1,3a,10_Dextran_003_005.fcs	5196
	mouse_001_1,3a,10_Dextran_001_003.fcs	5346

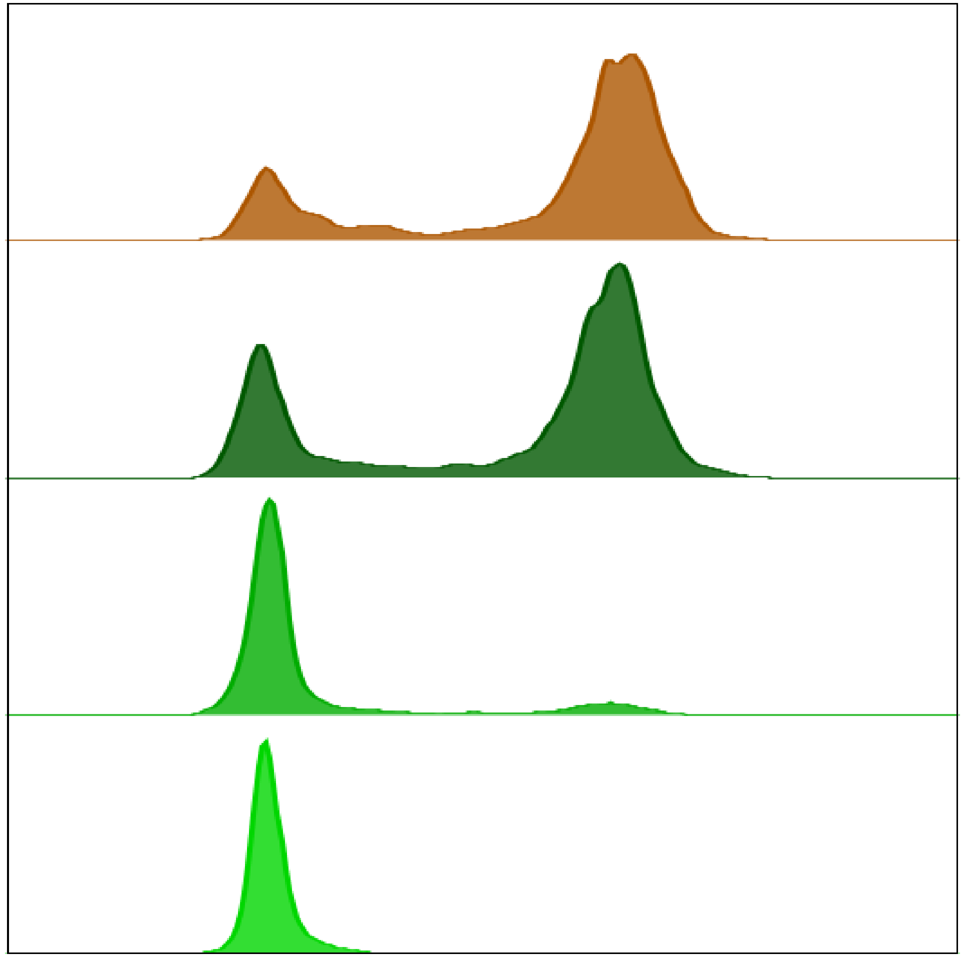

	Sample Name	Mean : Comp-Alexa Fluor 700-A
	mouse_002_1,3a,10_LCMV_015.fcs	6928
	mouse_001_1,3a,10_Dextran Sulfate_005_013.fcs	5595
	mouse_001_1,3a,10_Dextran Sulfate_003_011.fcs	1052
	mouse_001_1,3a,10_Dextran Sulfate_001_009.fcs	287
