# Supplementary material for: Sulfated glycosaminoglycans inhibit LCMV entry and modulate antiviral immunity and pathology
Source: EMBO Mol Med. 2026 Feb 23;18(4):1235–64. doi: 10.1038/s44321-026-00387-8 (PMC13083911; doi:10.1038/s44321-026-00387-8)
Supplement: Supplementary file 8 — Source data Fig. 6 [file 44321_2026_387_MOESM8_ESM.zip › Fig. 6/Fig. 6B/Fig. 6B.pdf]

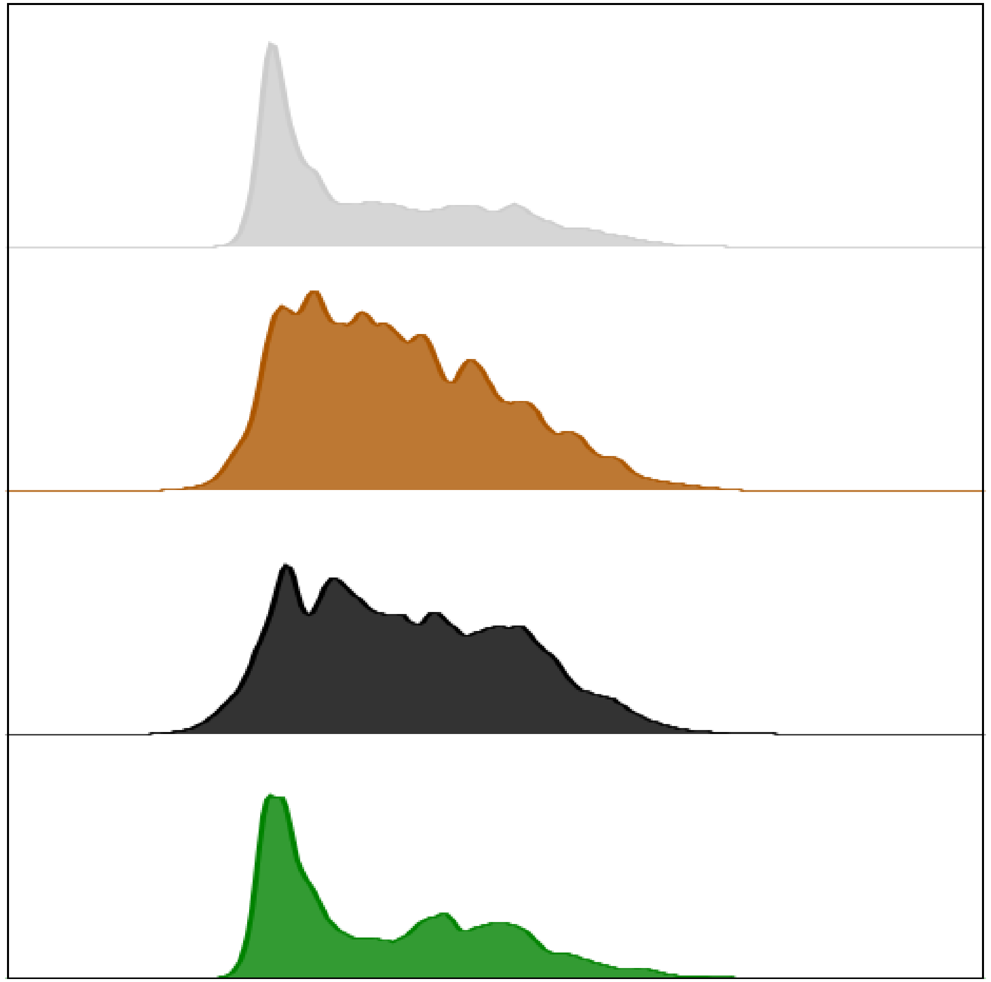

	Sample Name
(light grey)	Control_Medium_mouse_003_006.fcs
(orange)	Control_WE_mouse_003_009.fcs
(black)	BMDC_003_Dextran_002_035.fcs
(green)	BMDC_003_Dextran Sulfate_002_041.fcs

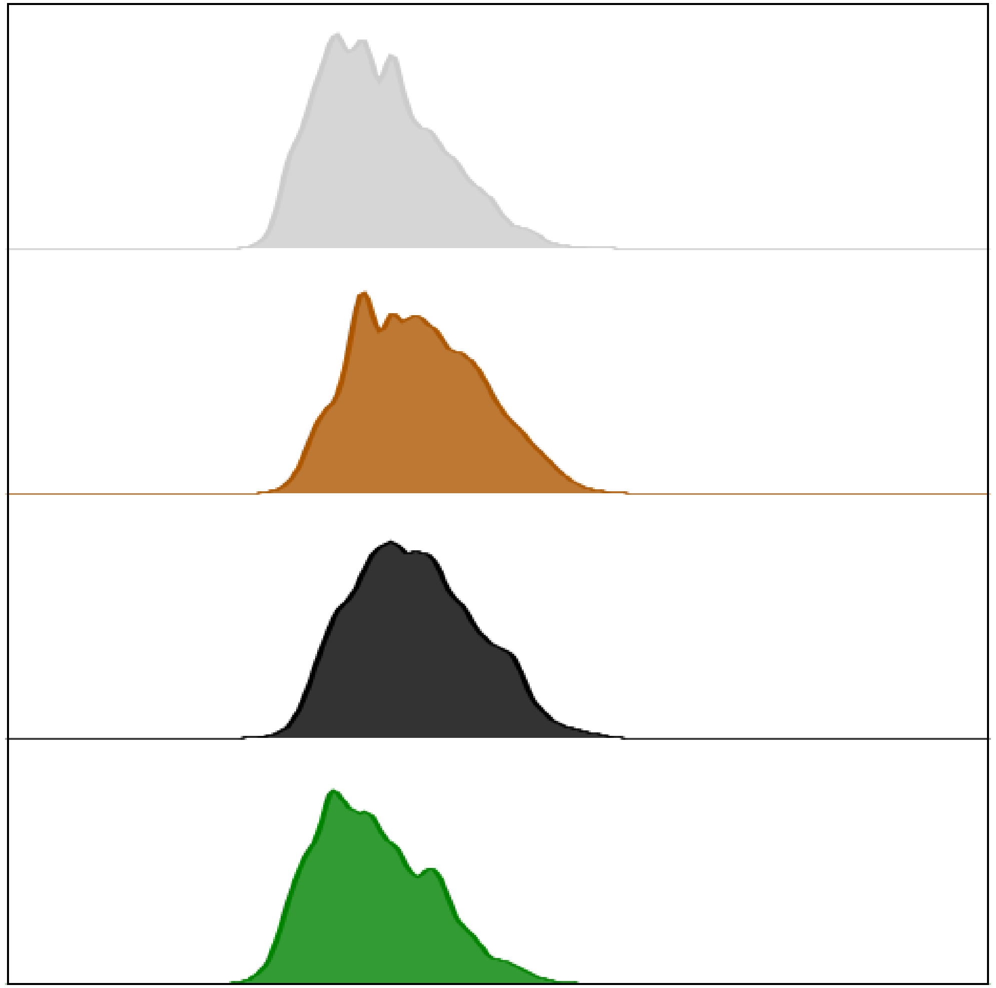

	Sample Name
	Control_Medium_mouse_003_006.fcs
	Control_WE_mouse_003_009.fcs
	BMDC_003_Dextran_002_035.fcs
	BMDC_003_Dextran Sulfate_002_041.fcs

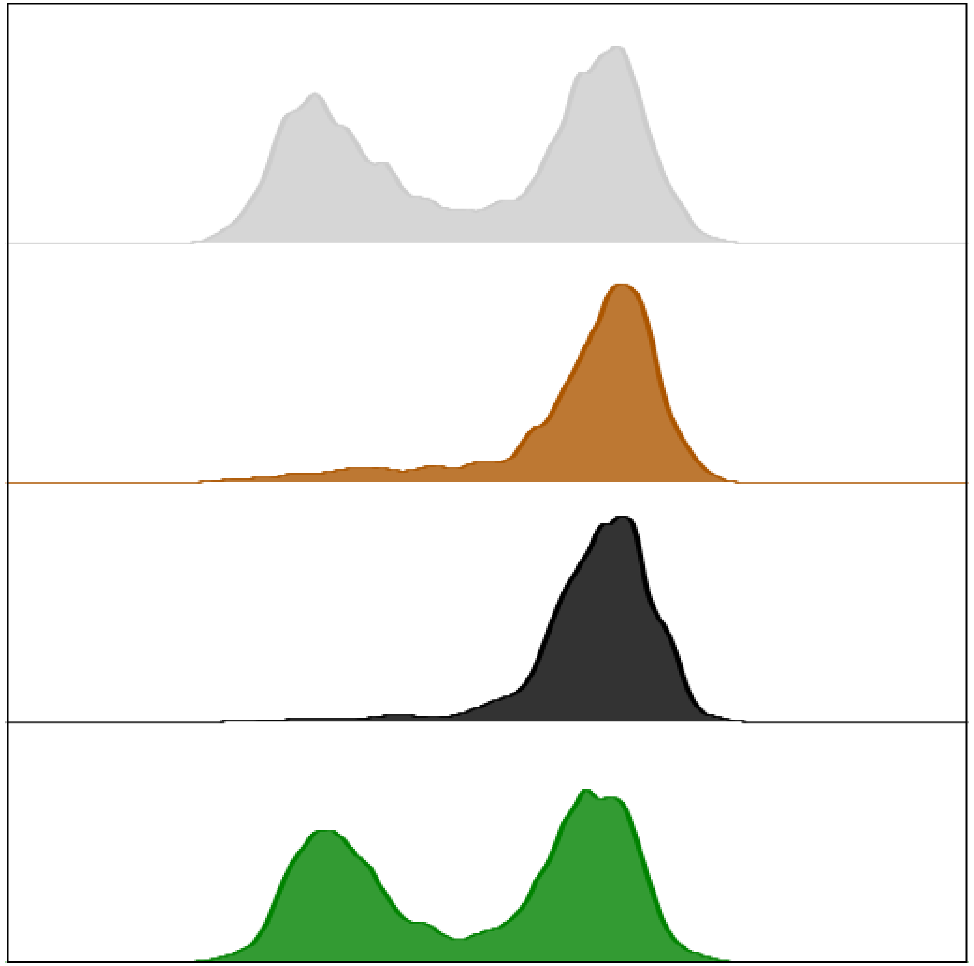

	Sample Name
	Control_Medium_mouse_003_006.fcs
	Control_WE_mouse_001_007.fcs
	BMDC_003_Dextran_002_035.fcs
	BMDC_003_Dextran Sulfate_002_041.fcs
