# Supplementary material for: Sulfated glycosaminoglycans inhibit LCMV entry and modulate antiviral immunity and pathology
Source: EMBO Mol Med. 2026 Feb 23;18(4):1235–64. doi: 10.1038/s44321-026-00387-8 (PMC13083911; doi:10.1038/s44321-026-00387-8)

D8 Vehicle, 20x N=4, 4 ROI **DAPI F4/80 LCMVNP Collagen 1**

RL blue Lv

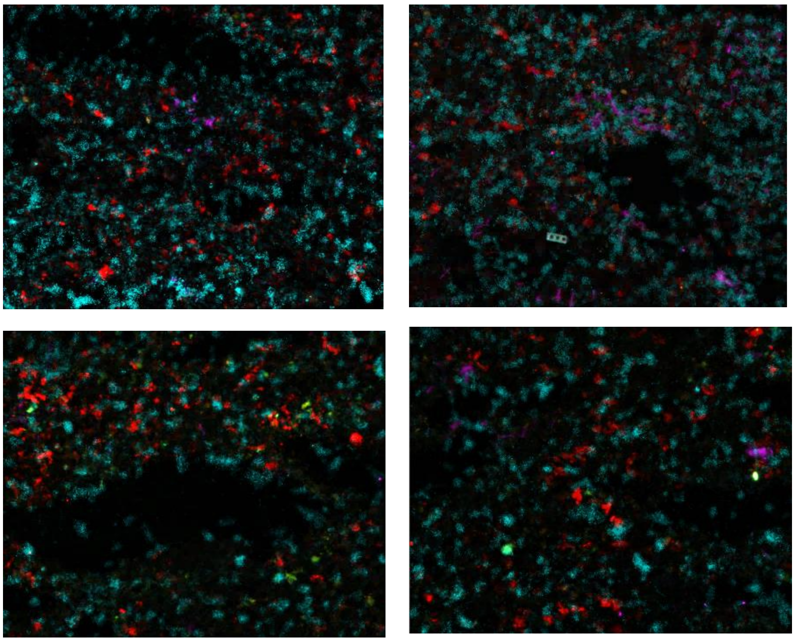

RR Lv

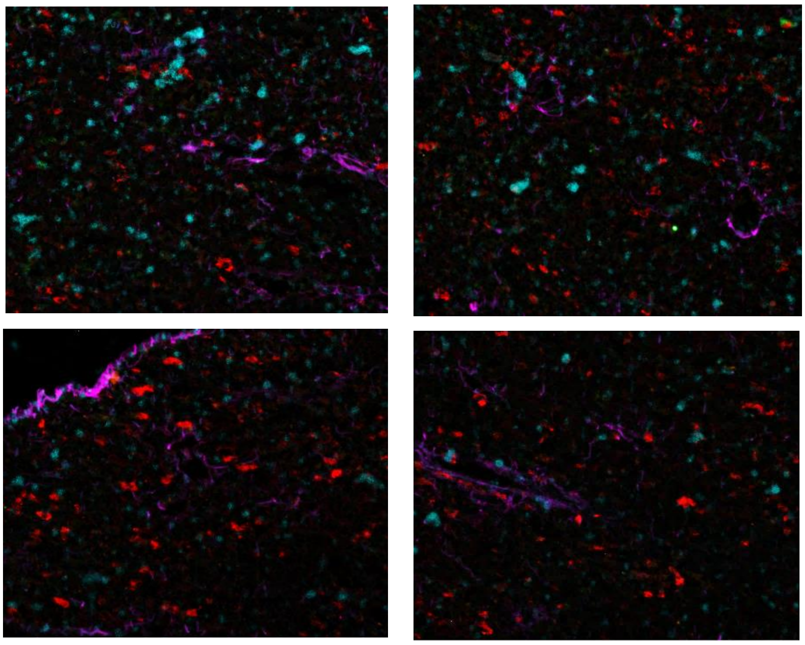

RL Lv

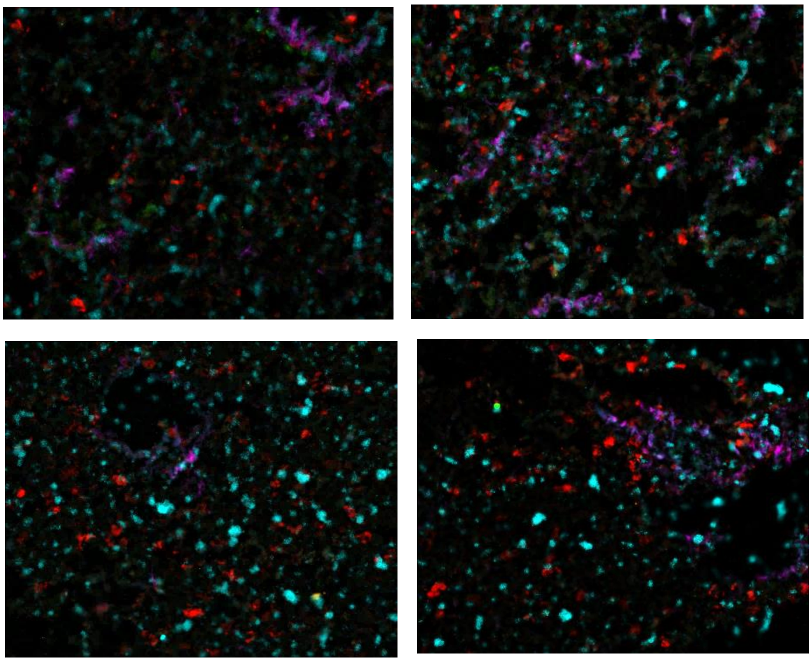

LL Lv

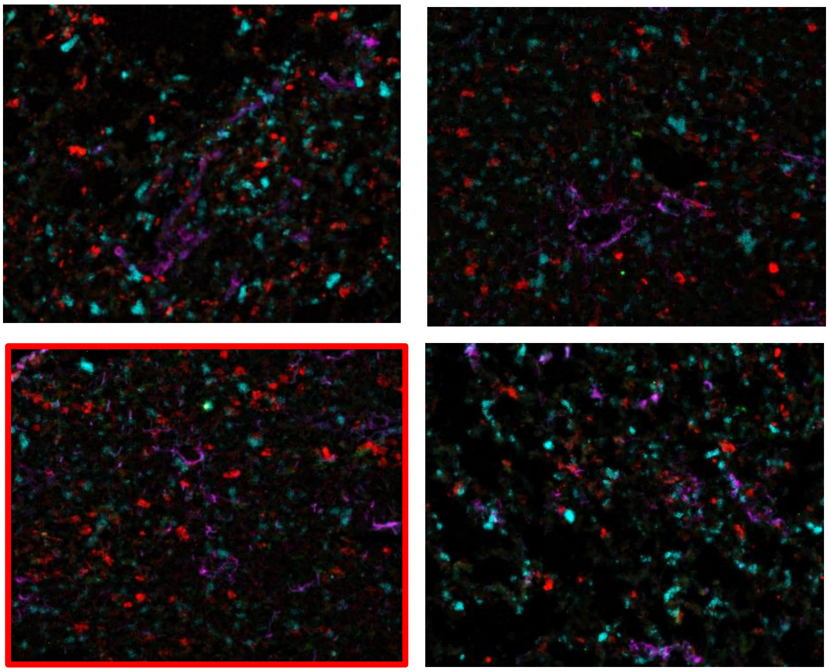

D8 +Dextran sulphate; 20x N=4, 4 ROI

RL ds Lv blue

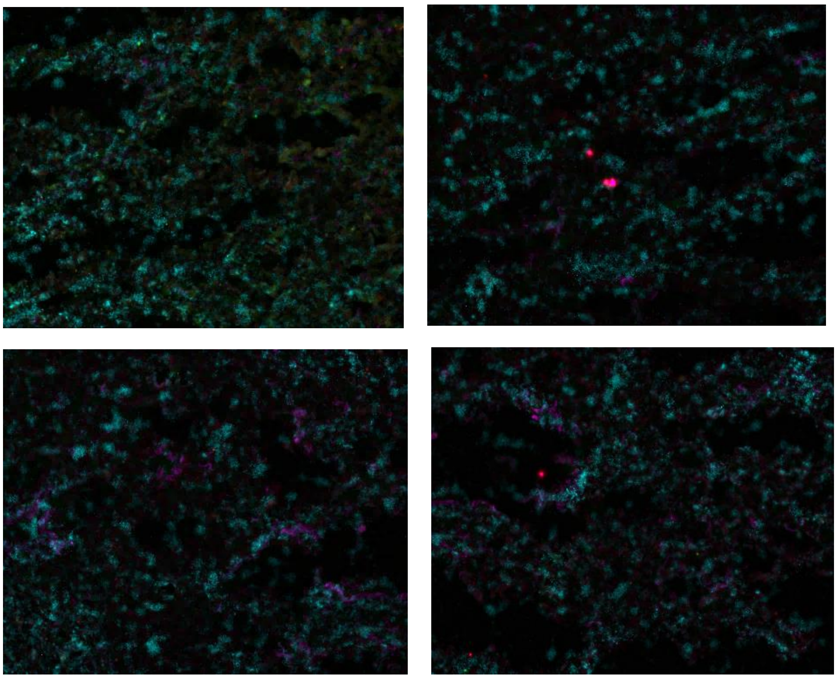

RR ds Lv

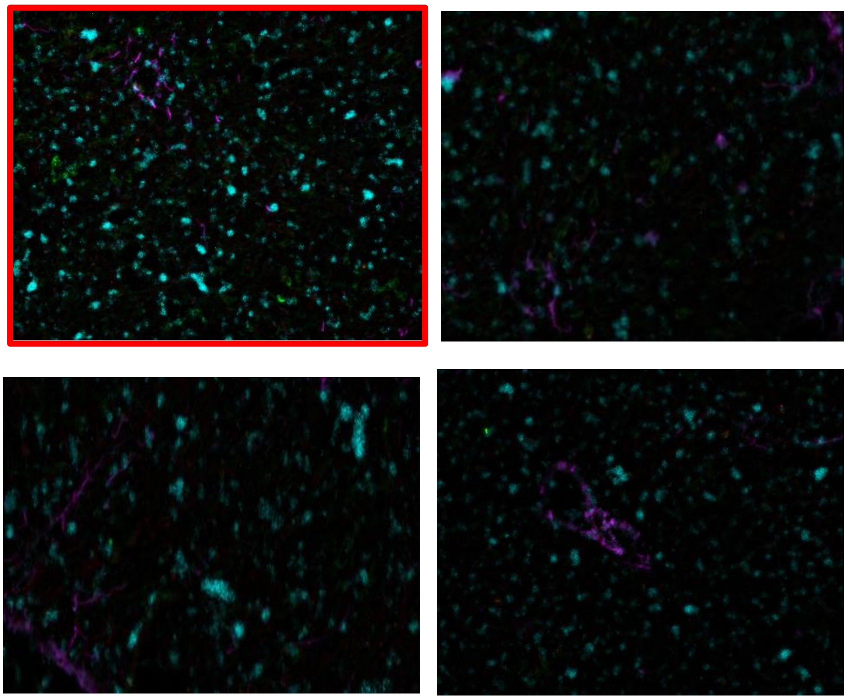

RL Ds Lv

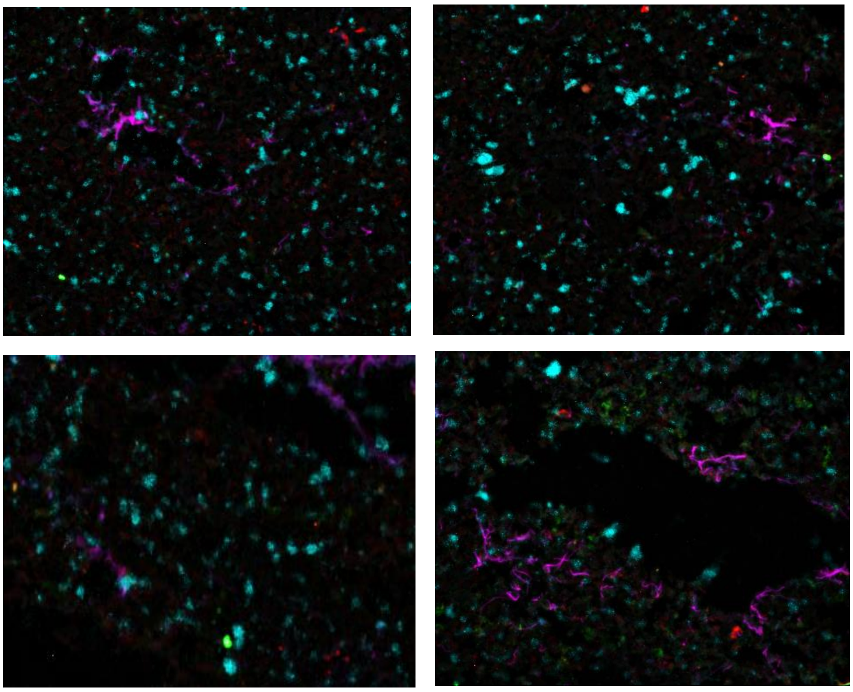

L Ds Lv

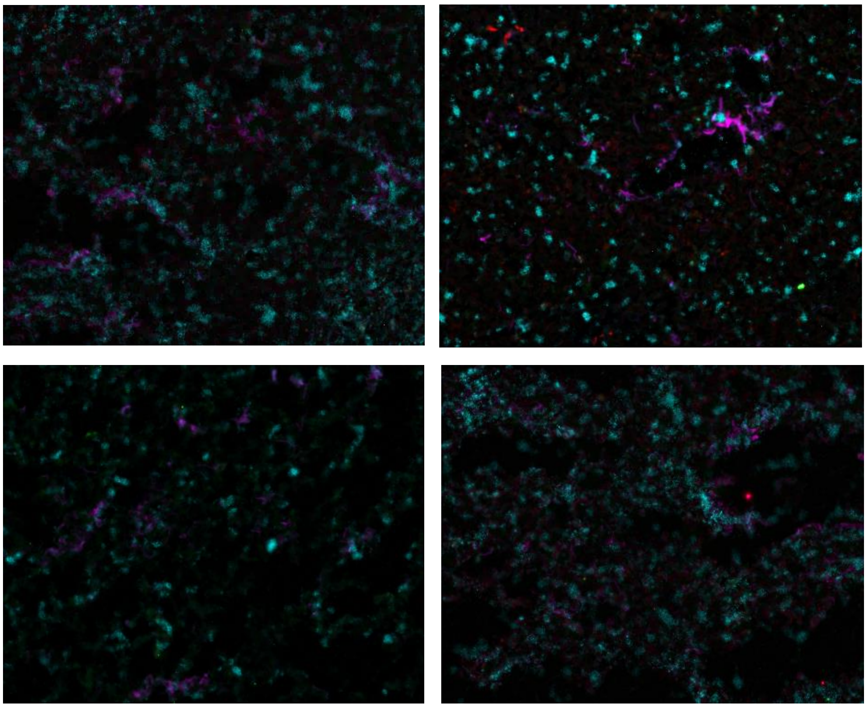

Supplement: Supplementary file 9 — Source data Fig. 7 [file 44321_2026_387_MOESM9_ESM.zip › Fig. 7/Fig. 7K/Fig. 7K_all.pdf]
