# Supplementary material for: Sulfated glycosaminoglycans inhibit LCMV entry and modulate antiviral immunity and pathology
Source: EMBO Mol Med. 2026 Feb 23;18(4):1235–64. doi: 10.1038/s44321-026-00387-8 (PMC13083911; doi:10.1038/s44321-026-00387-8)

D8 Vehicle, 20x N=4, 4 ROI **DAPI Cd169 LCMVNP F4/80**

LL Sp

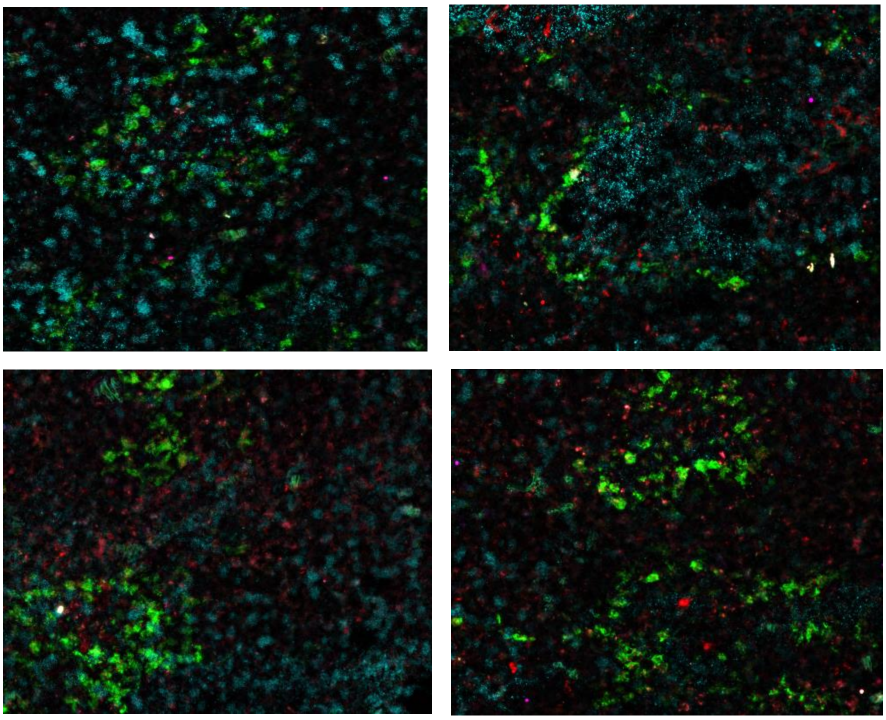

RL Sp

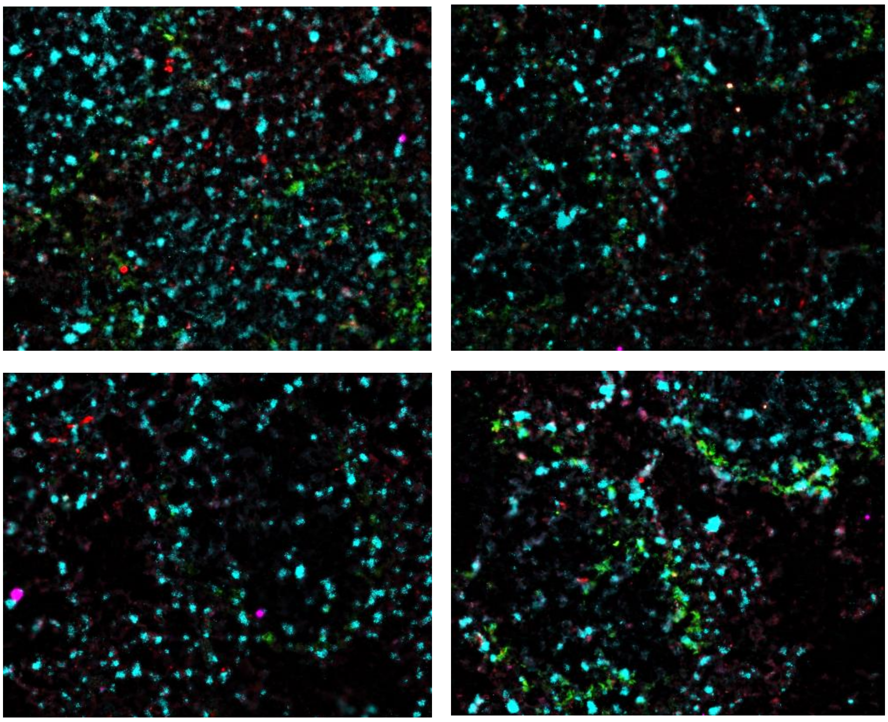

RR Sp

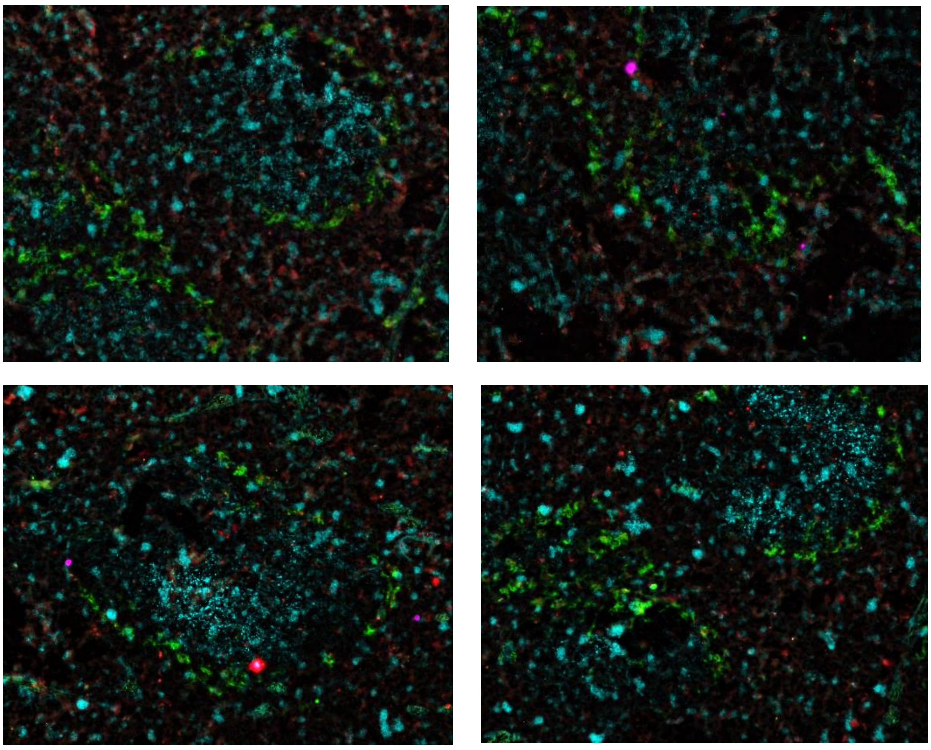

L Sp

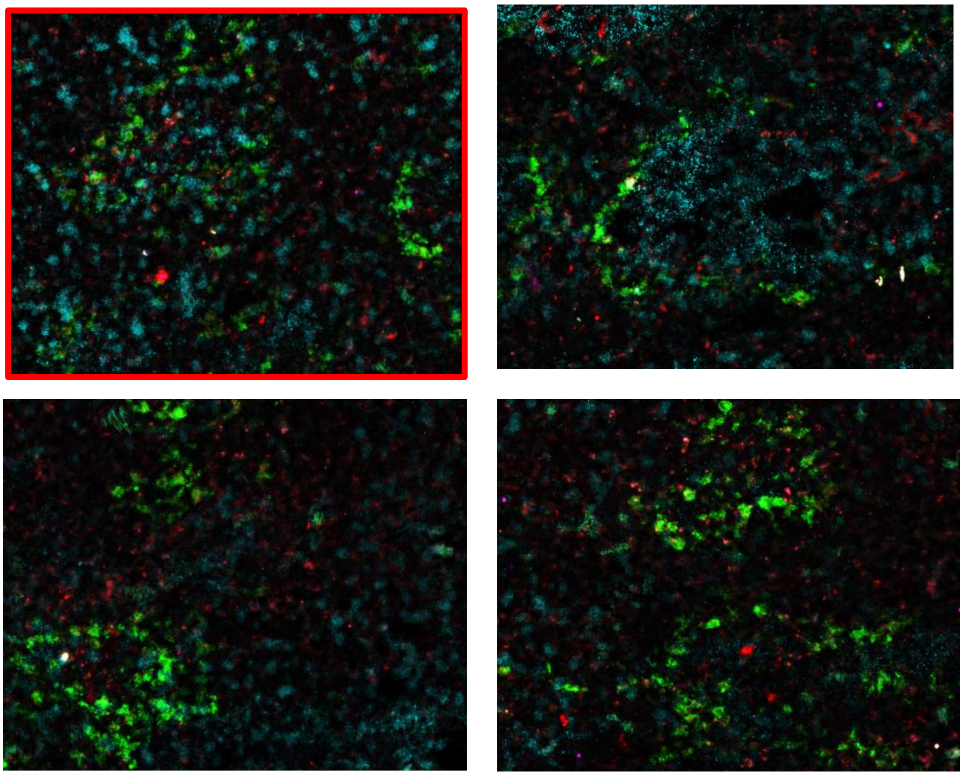

D8 +Dextran sulphate, 20x N=4, 4 ROI **DAPI Cd169 LCMVNP F4/80**

L Ds Sp

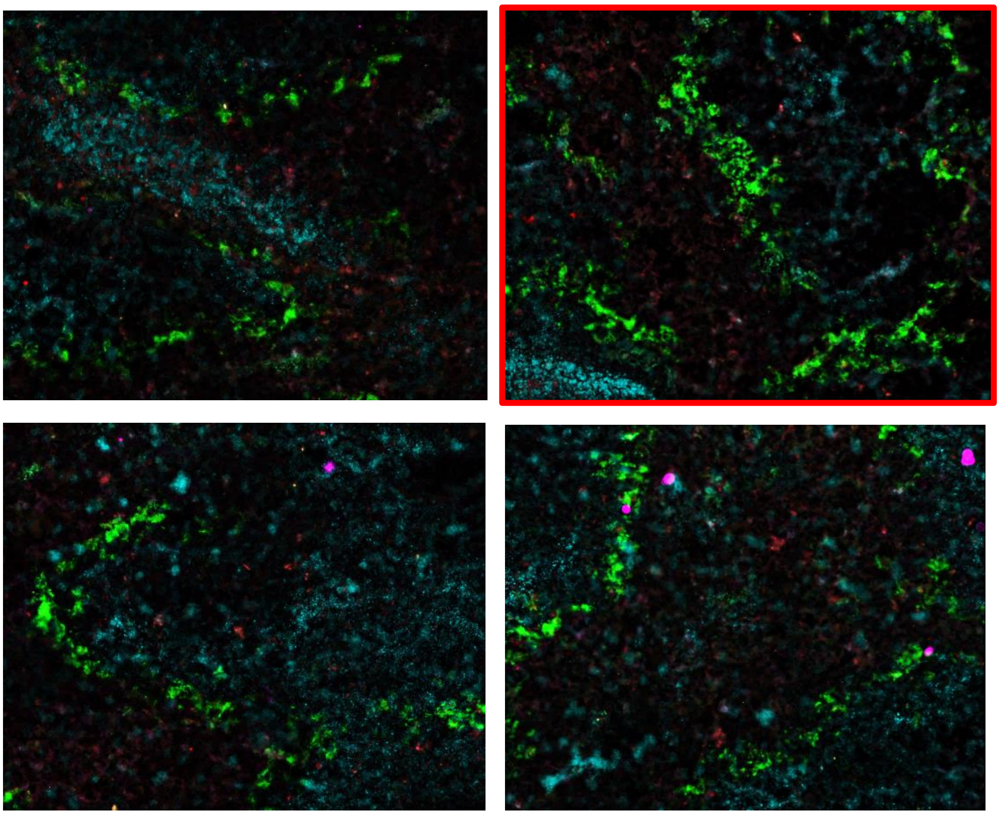

RR Ds Sp

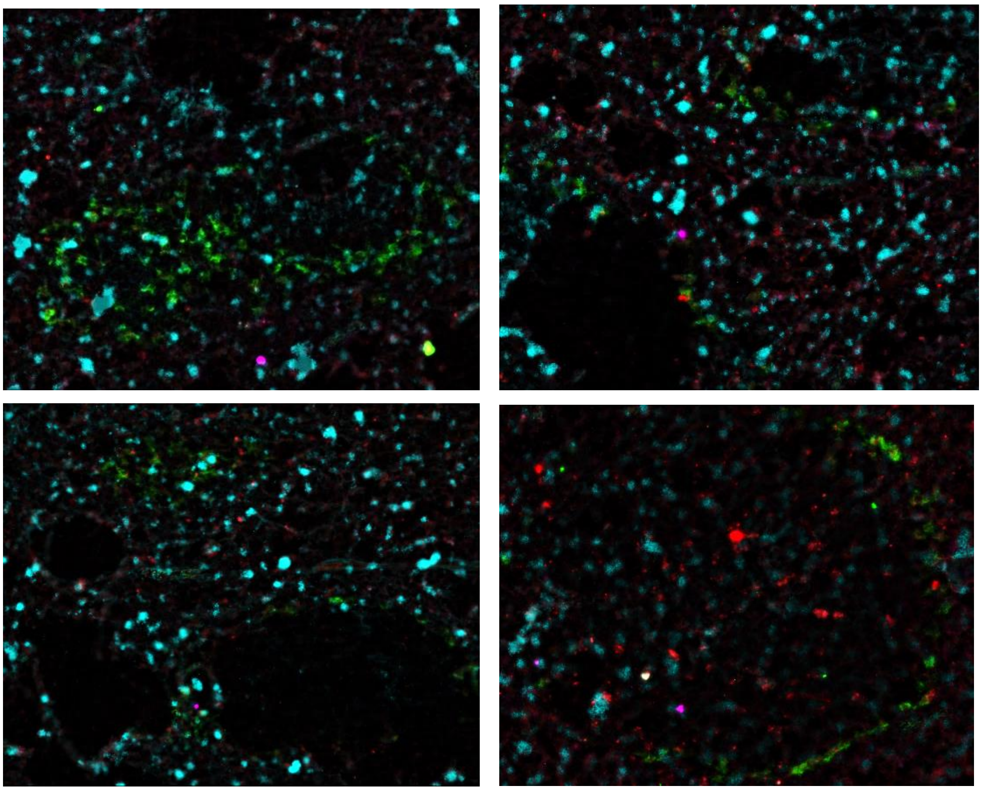

RL Ds Sp

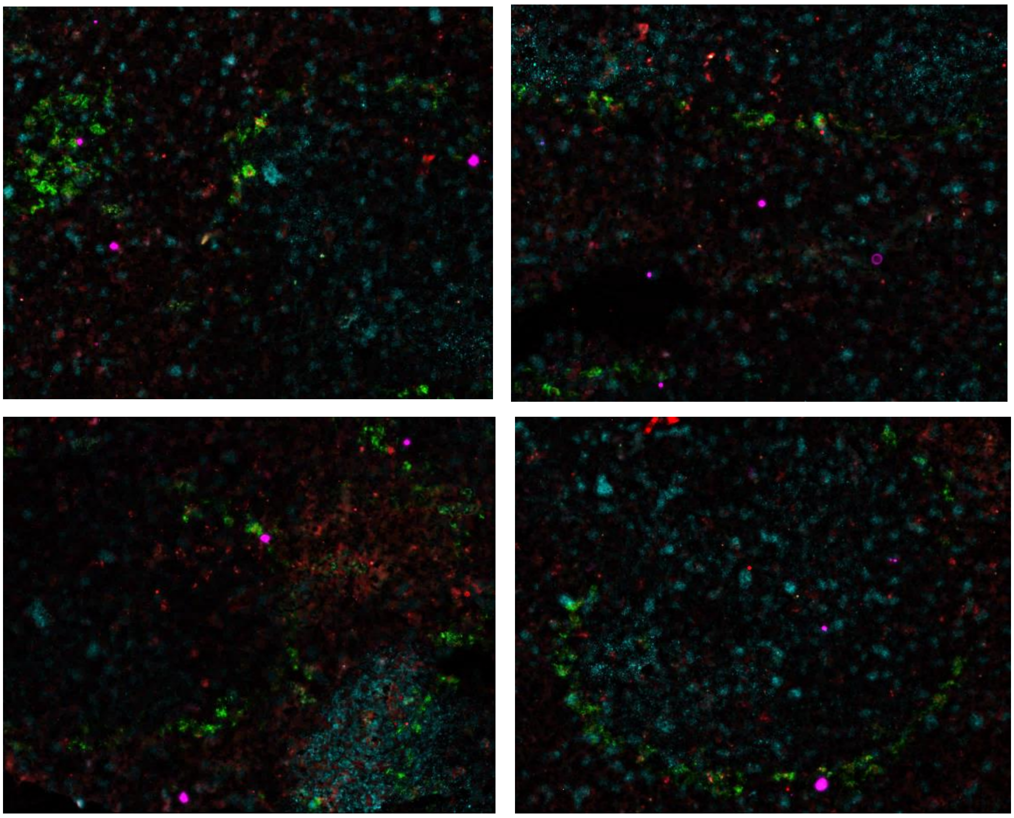

L Ds Sp

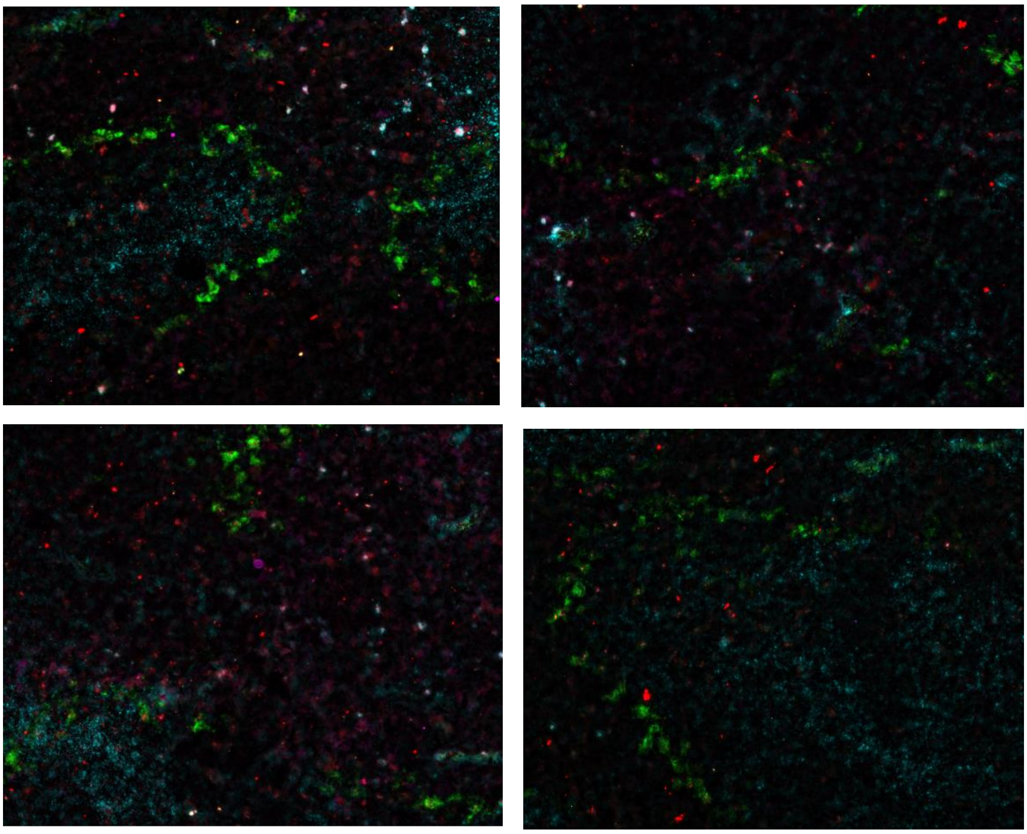

Supplement: Supplementary file 9 — Source data Fig. 7 [file 44321_2026_387_MOESM9_ESM.zip › Fig. 7/Fig. 7J/Fig. 7J_all.pdf]
