# Supplementary material for: Sulfated glycosaminoglycans inhibit LCMV entry and modulate antiviral immunity and pathology
Source: EMBO Mol Med. 2026 Feb 23;18(4):1235–64. doi: 10.1038/s44321-026-00387-8 (PMC13083911; doi:10.1038/s44321-026-00387-8)
Supplement: Supplementary file 11 — Figure EV2 Source Data [file 44321_2026_387_MOESM11_ESM.zip › Fig. EV2/Fig. EV2D.pdf]

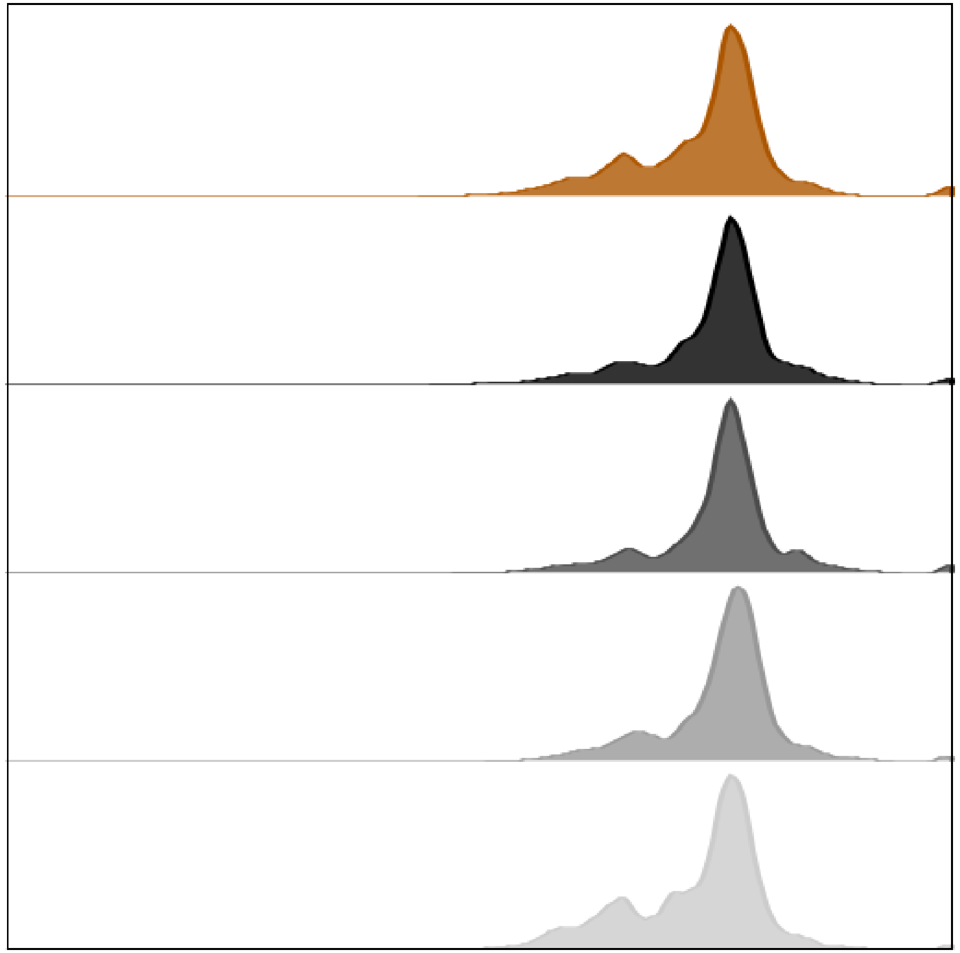

	Sample Name	Mean : Comp-APC-A
	Dextran 48h_ctrl - 2_067.fcs	25248
	Dextran 48h_005 - 2_070.fcs	26847
	Dextran 48h_05 - 2_073.fcs	27758
	Dextran 48h_5 - 3_077.fcs	27377
	Dextran 48h_50- 1_078.fcs	22483

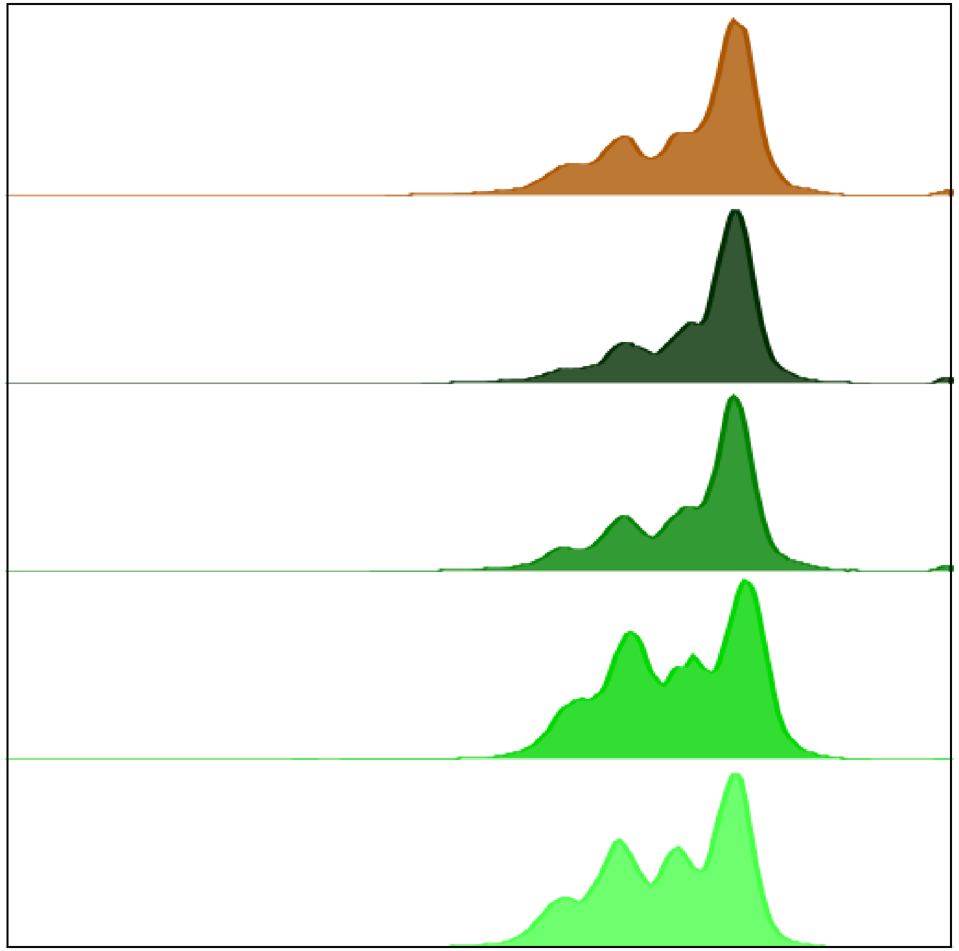

	Sample Name	Mean : Comp-APC-A
	Dextran sulfate 48h_ctrl - 2_046.fcs	21903
	Dextran sulfate 48h_005 - 2_049.fcs	23516
	Dextran sulfate 48h_05 - 2_052.fcs	22525
	Dextran sulfate 48h_5 - 3_056.fcs	19427
	Dextran sulfate 48h_50- 1_057.fcs	17526
