# Supplementary material for: Sulfated glycosaminoglycans inhibit LCMV entry and modulate antiviral immunity and pathology
Source: EMBO Mol Med. 2026 Feb 23;18(4):1235–64. doi: 10.1038/s44321-026-00387-8 (PMC13083911; doi:10.1038/s44321-026-00387-8)
Supplement: Supplementary file 11 — Figure EV2 Source Data [file 44321_2026_387_MOESM11_ESM.zip › Fig. EV2/Fig. EV2A-B/Fig. EV2A.pdf]

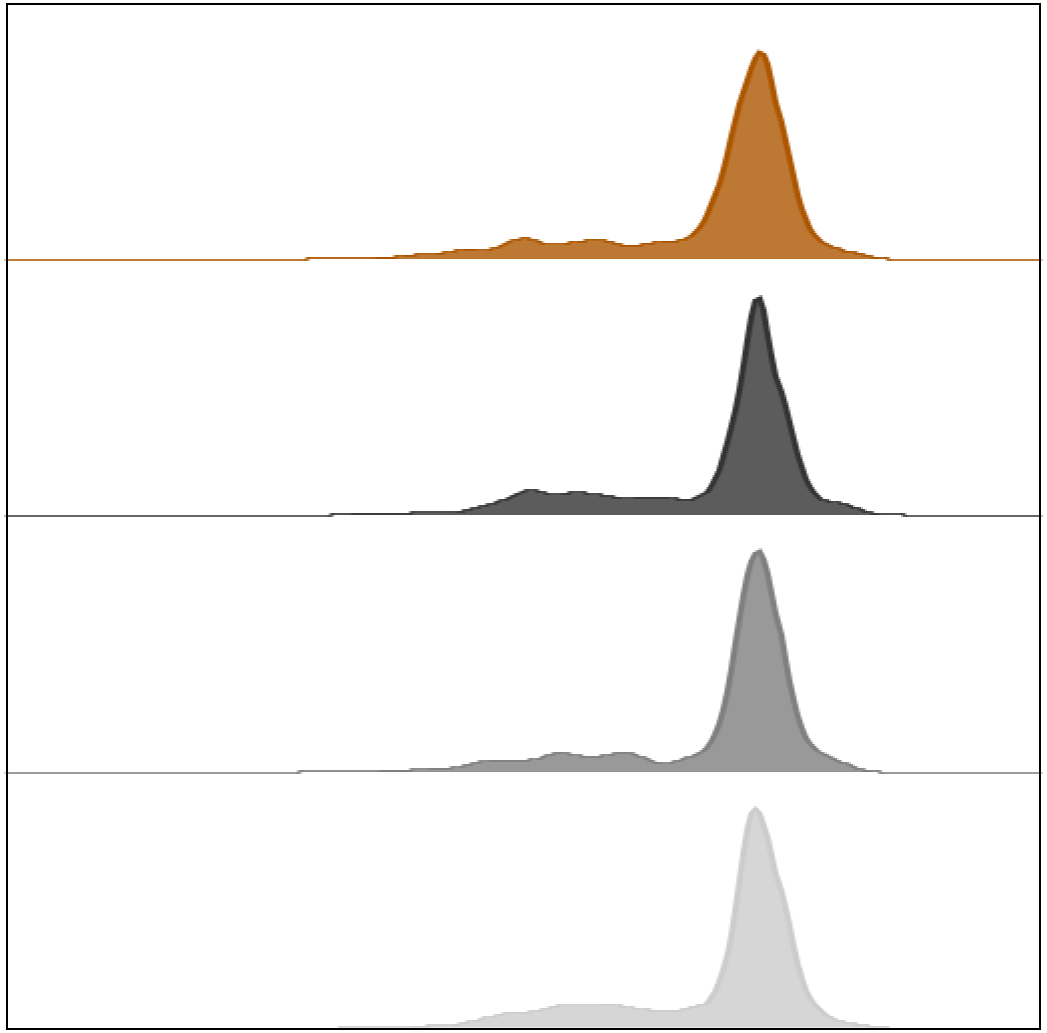

	Sample Name	Mean : Comp-APC-A
	BMDC_mouse_2_1,3a,10_72h_LCMV_099.fcs	15520
	BMDC_mouse_2_1,3a,10_72h_Dextran_005_105.fcs	15596
	BMDC_mouse_2_1,3a,10_72h_Dextran_003_103.fcs	15788
	BMDC_mouse_2_1,3a,10_72h_Dextran_001_101.fcs	15403

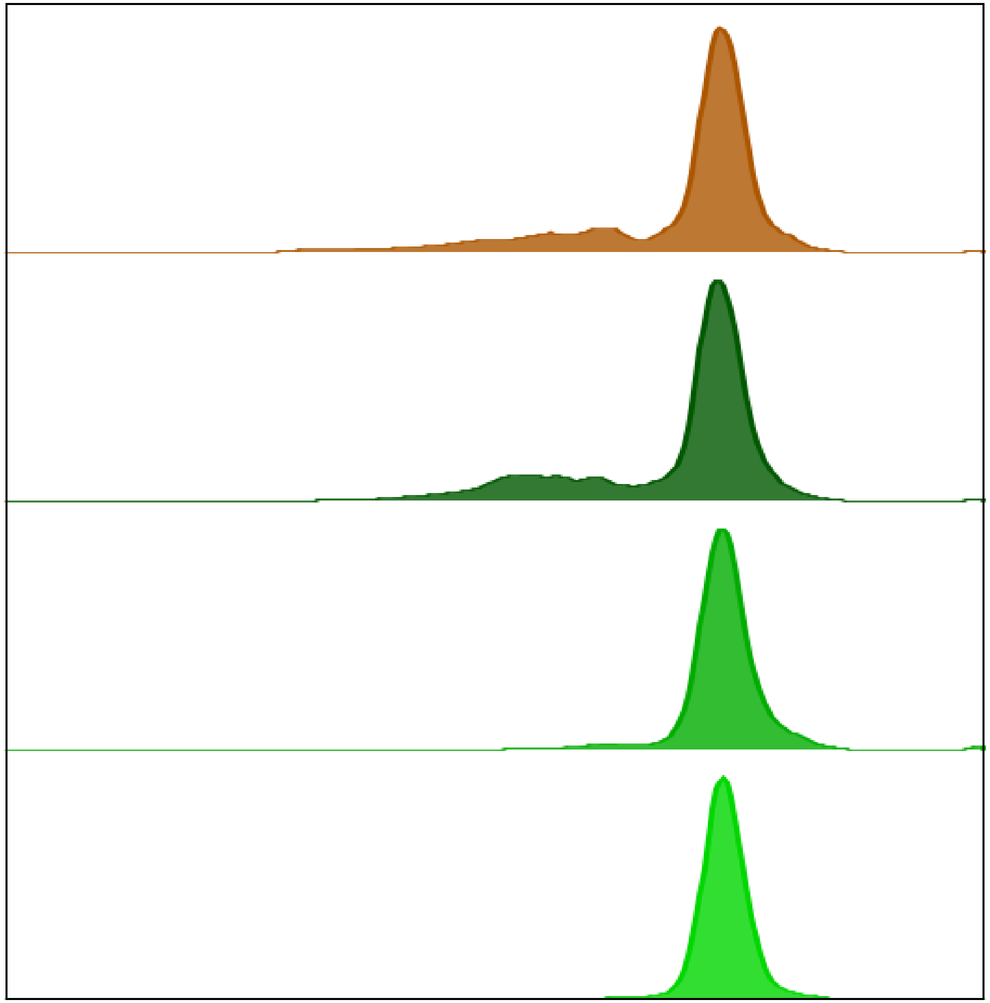

	Sample Name	Mean : Comp-APC-A
	BMDC_mouse_1_1,3a,10_72h_LCMV_085.fcs	16961
	BMDC_mouse_1_1,3a,10_72h_Dextran sulfate_005_097.fcs	16287
	BMDC_mouse_1_1,3a,10_72h_Dextran sulfate_003_095.fcs	20916
	BMDC_mouse_1_1,3a,10_72h_Dextran sulfate_001_093.fcs	19770
