# Supplementary material for: Sulfated glycosaminoglycans inhibit LCMV entry and modulate antiviral immunity and pathology
Source: EMBO Mol Med. 2026 Feb 23;18(4):1235–64. doi: 10.1038/s44321-026-00387-8 (PMC13083911; doi:10.1038/s44321-026-00387-8)

D1 Vehicle NN Lv 20x N=4, 4 ROI   **DAPI CD31 LCMVNP F4/80**

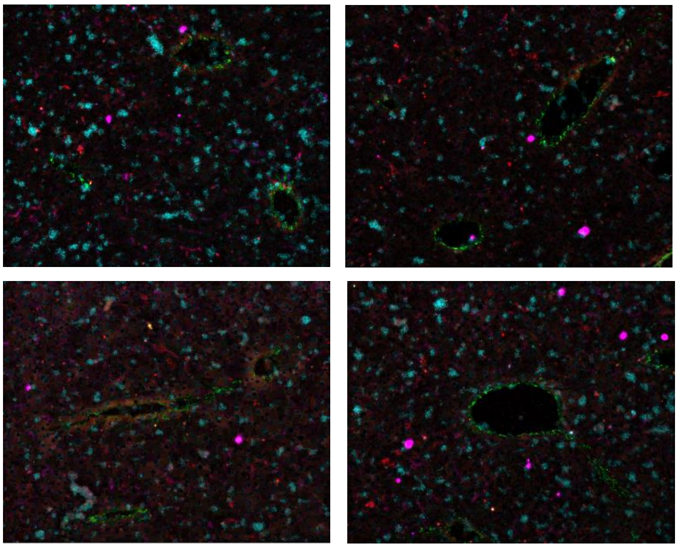

L Lv

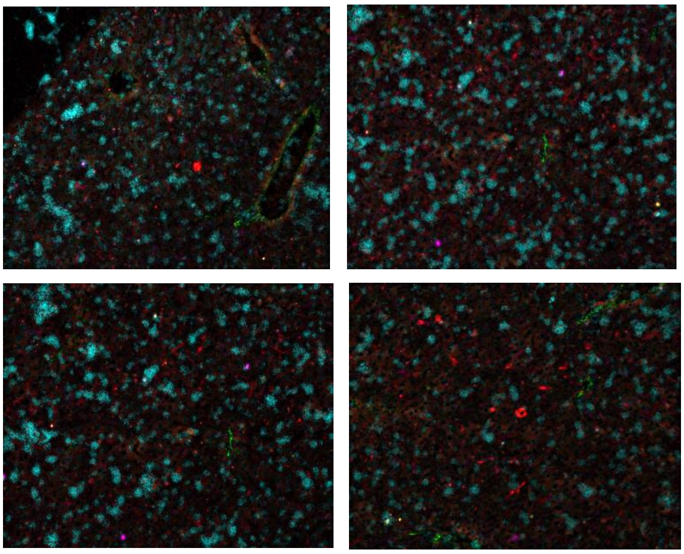

R Lv

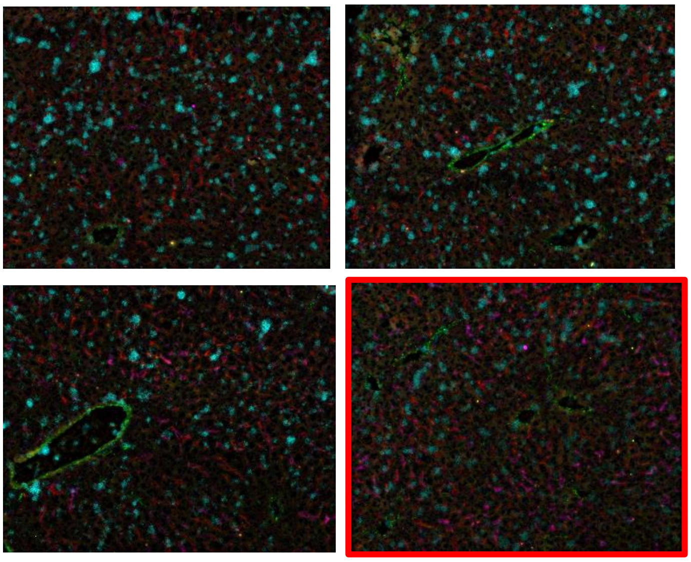

LL Lv

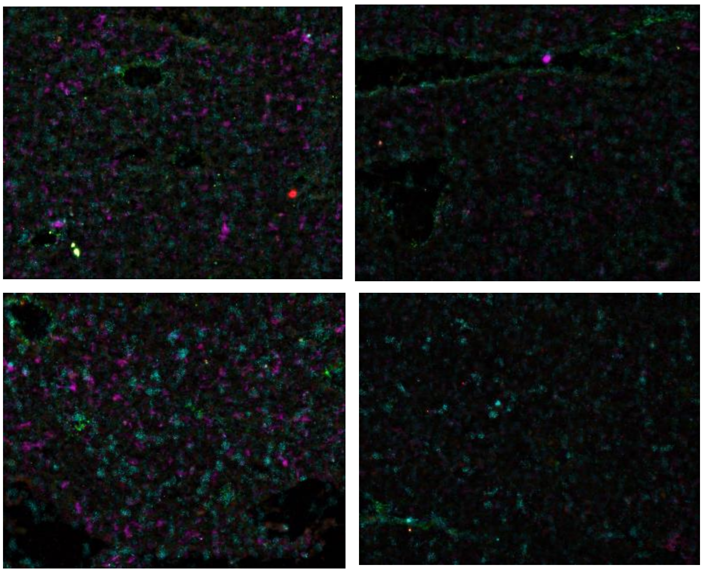

D1 +Dextran sulphate; 20x N=4, 4 ROI

R Ds Lv

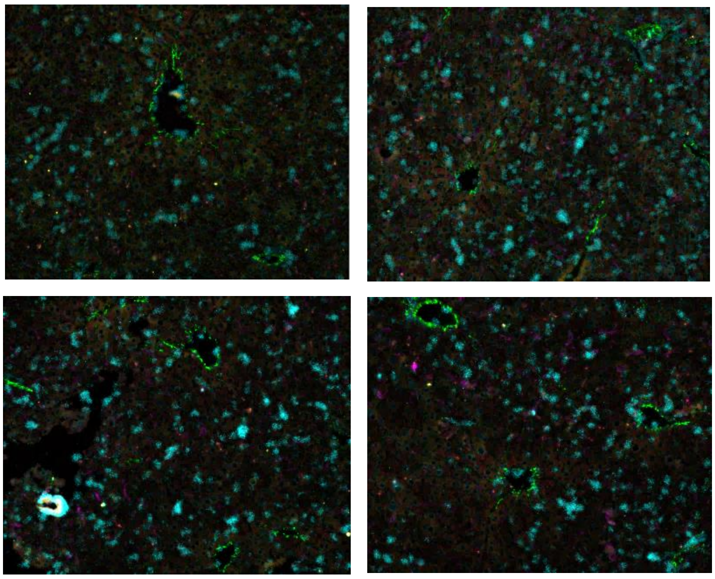

L Ds Lv

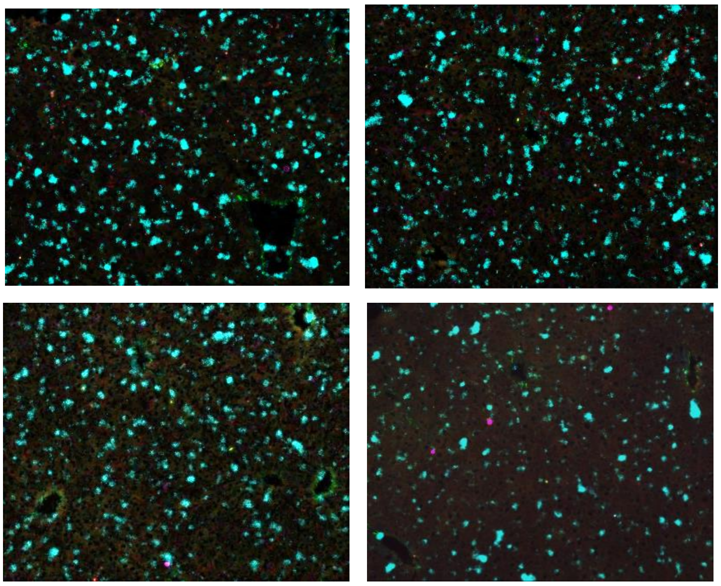

NN Ds Lv

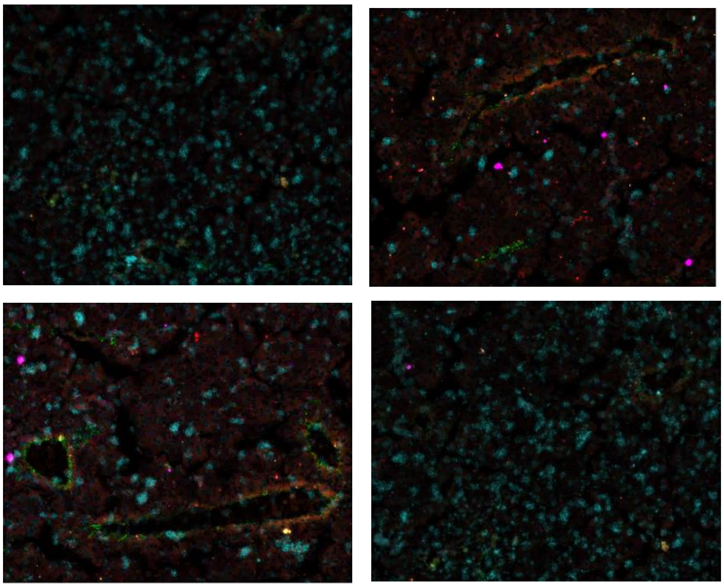

RR Ds Lv

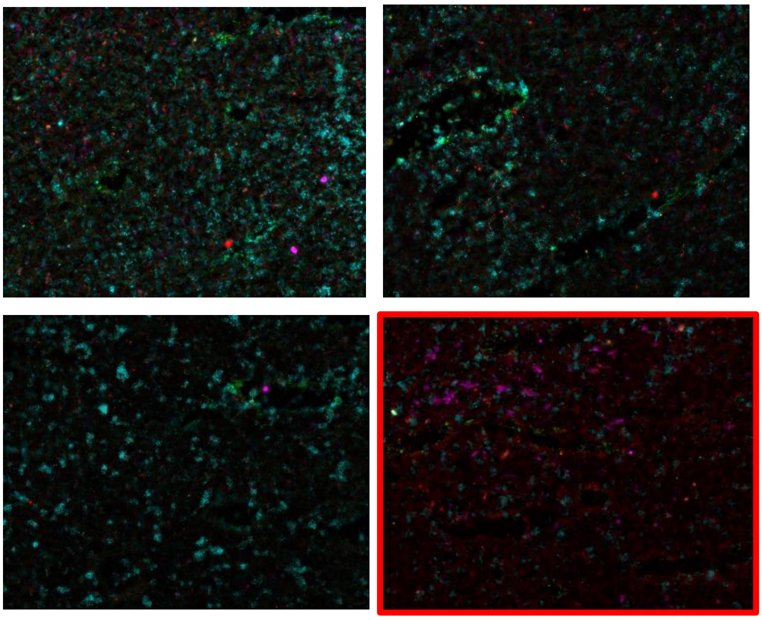

Supplement: Supplementary file 15 — Appendix Figure Source Data [file 44321_2026_387_MOESM15_ESM.zip › Appendix Fig. S6/Fig. S6A/Fig. S6A_all.pdf]
