# Supplementary material for: Sulfated glycosaminoglycans inhibit LCMV entry and modulate antiviral immunity and pathology
Source: EMBO Mol Med. 2026 Feb 23;18(4):1235–64. doi: 10.1038/s44321-026-00387-8 (PMC13083911; doi:10.1038/s44321-026-00387-8)
Supplement: Supplementary file 16 — Expanded View Figures [file 44321_2026_387_MOESM16_ESM.pdf]

Expanded View Figures

Figure EV1. Inhibition of Arenavirus infection by GAGs depends on their structure and level of sulfation.

(**A**) Chemical structures of GAGs used in the study. (**B**, **C**) MC57G cells were treated with dextran and dextran sulfate (500 μg/ml) for 1 h. Next, cells were infected with LCMV WE (MOI 0.01) for 3 h. After the infection, cells were washed three times with PBS and fresh medium containing studied compounds was re-added to the cells. The cells were collected after 8 (**B**) and 24 h (**C**) for RNA isolation and RT-PCR. Data presented as number of cognate mRNA copies per copy of mRNA for reference housekeeping gene, mean ± SEM, $n = 3$. *$P < 0.05$, **$P < 0.01$ compared to samples treated with dextran. Statistical significance was assessed by Student's *t* test. (**D**, **E**) MC57G cells were treated with dextran and dextran sulfate (500 μg/ml) for 1 h. Next, cells were infected with LCMV WE (MOI 0.1) for 3 h. After the infection, cells were washed three times with PBS and fresh medium containing studied compounds was re-added to the cells. The cells were collected after 8 (**D**) and 24 h (**E**) for RNA isolation and RT-PCR. Data presented as number of cognate mRNA copies per copy of mRNA for reference housekeeping gene, mean ± SEM, $n = 3$. *$P < 0.05$, **$P < 0.01$, ***$P < 0.001$ compared to samples treated with dextran. Statistical significance was assessed by Student's *t* test. (**F**) BHK cells were treated with Man70 or Man70-sulf for 1 h. Next, cells were infected with LCMV WE (MOI 0.5) for 1 h at 4 °C. After the infection, cells were incubated at 37 °C for 2.5 h before adding monensin to block the Golgi apparatus and intracellular protein transport. Cells were then stained for viability and LCMV NP. Data presented as mean ± SEM, $n = 9$–12. **$P < 0.01$, ***$P < 0.001$ compared to vehicle control. Statistical significance was assessed by one-way ANOVA. (**G**) MC57G cells were treated with heparin, dextran, or dextran sulfate and infected with Tacaribe (TCRV), Parana (PARV), and Morogoro (MORV) viruses (BSL2) at MOI of 0.001. Cell culture supernatant was harvested after two days and virus titers were determined by immunofocus assay. Data presented as focus-forming units (ffu) per ml, mean ± SEM $n = 3$. *$P < 0.05$, **$P < 0.01$, ***$P < 0.01$ compared to dextran treatment. Statistical significance was assessed by two-way ANOVA. Source data are available online for this figure.

 © The Author(s)

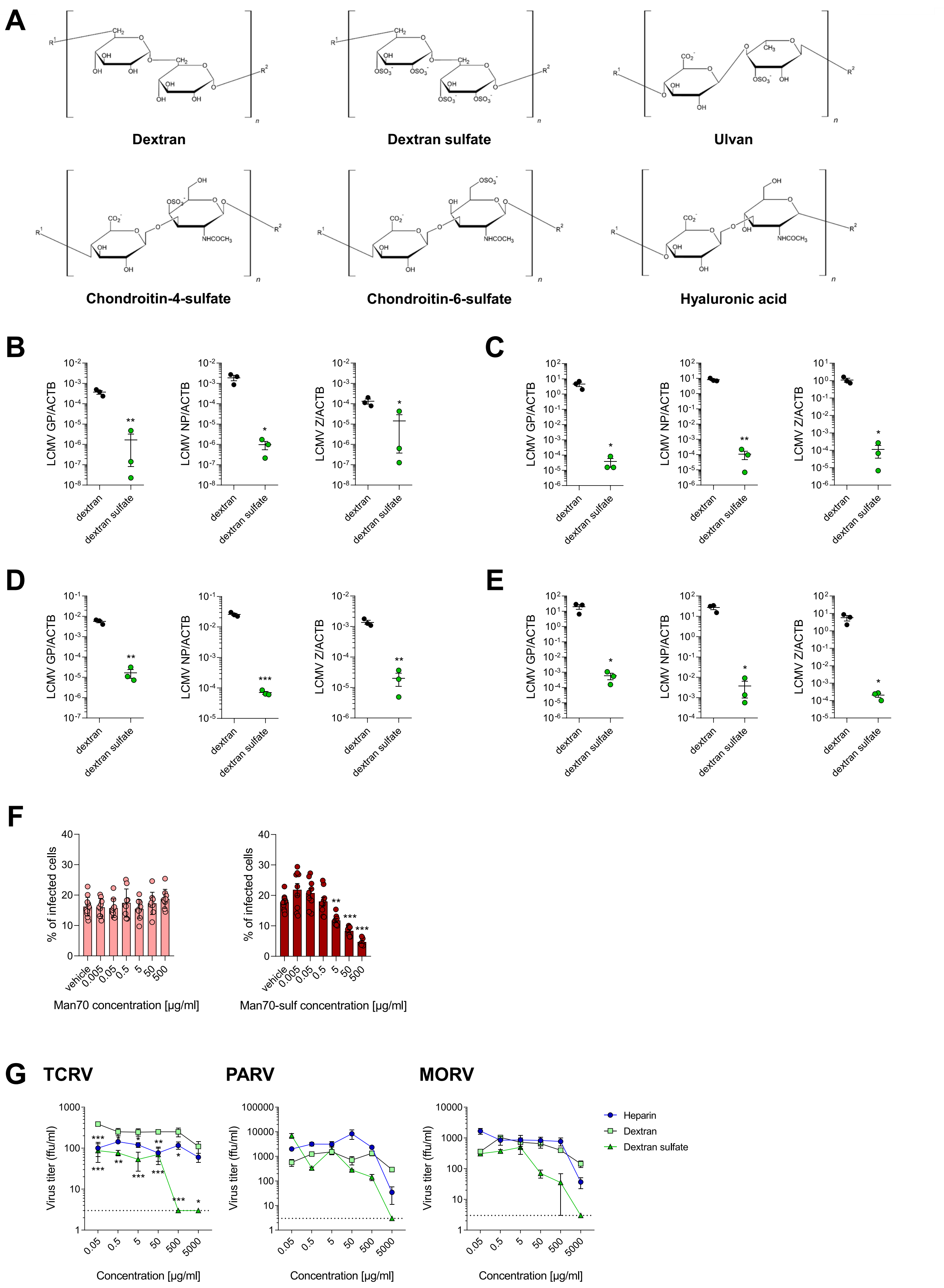
A
Dextran
Dextran sulfate
Ulvan
Chondroitin-4-sulfate
Chondroitin-6-sulfate
Hyaluronic acid
B
C
D
E
LCMV GP/ACTB
LCMV NP/ACTB
LCMV Z/ACTB
dextran
dextran sulfate
F
% of infected cells
vehicle
Man70 concentration [μg/ml]
Man70-sulf concentration [μg/ml]
G
TCRV
PARV
MORV
Virus titer (ffu/ml)
Concentration [μg/ml]
Heparin
Dextran
Dextran sulfate

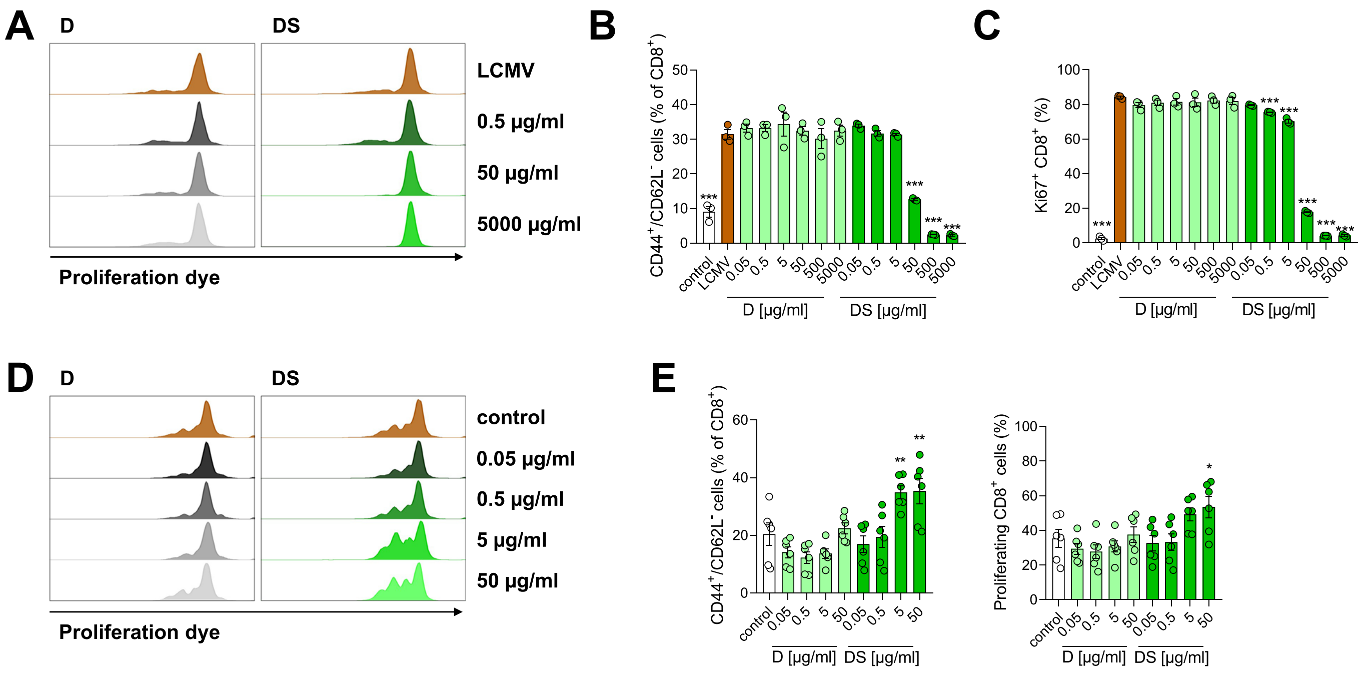

Figure EV2. Dextran sulfate decreases infectivity in BMDCs causing impaired antigen presentation and T-cell function.

(**A**, **B**) BMDCs were treated with dextran/dextran sulfate for 1 h. Cells were subsequently infected with LCMV WE (MOI 0.5) for 1 h. Then the dextrans and virus were removed by washing. $CD8^+$ $P14^+$ T cells were isolated, stained with proliferation dye (10 min, 37 °C) and mixed with BMDCs at a ratio of 10:1. After 72 h of incubation, T cells were analyzed for proliferation (**A**) and effector populations (**B**). For proliferation, representative histograms are shown ($n = 3$). For effector populations, data presented as mean ± SEM, $n = 3$. ***$P < 0.001$ compared to LCMV-infected control. Statistical significance was assessed by one-way ANOVA. (**C**) BMDCs were treated with dextran/dextran sulfate for 1 h. Cells were subsequently infected with LCMV WE (MOI 0.5) for 1 h. Then the dextrans and virus were removed by washing. $CD8^+$ $P14^+$ T cells were isolated and mixed with BMDCs at 1:10 ratio (BMDCs:T cells). After 72 h of incubation, T cells were analyzed for Ki67 expression. Data presented as mean ± SEM, $n = 3$. ***$P < 0.001$ compared to LCMV-infected control. Statistical significance was assessed by one-way ANOVA. (**D**, **E**) $CD8^+$ T cells were isolated from spleen and lymph nodes of C57BL/6 J mice, activated with anti-CD3/CD28 antibodies, and at the same time treated with dextran (**D**) or dextran sulfate (DS). For proliferation analysis, the cells were additionally stained with cell proliferation dye for 10 min at 37 °C. After 48 h, the cells were incubated with anti-CD8, -CD62L, -CD44 antibodies for 30 min at 4 °C. Proliferation and T-cell populations were analyzed by flow cytometry. (**D**) Representative proliferation histograms are shown. (**E**) Data presented as mean ± SEM, $n = 6$. *$P < 0.05$, **$P < 0.01$, compared to non-treated control. Statistical significance was assessed by one-way ANOVA. Source data are available online for this figure.

 © The Author(s)

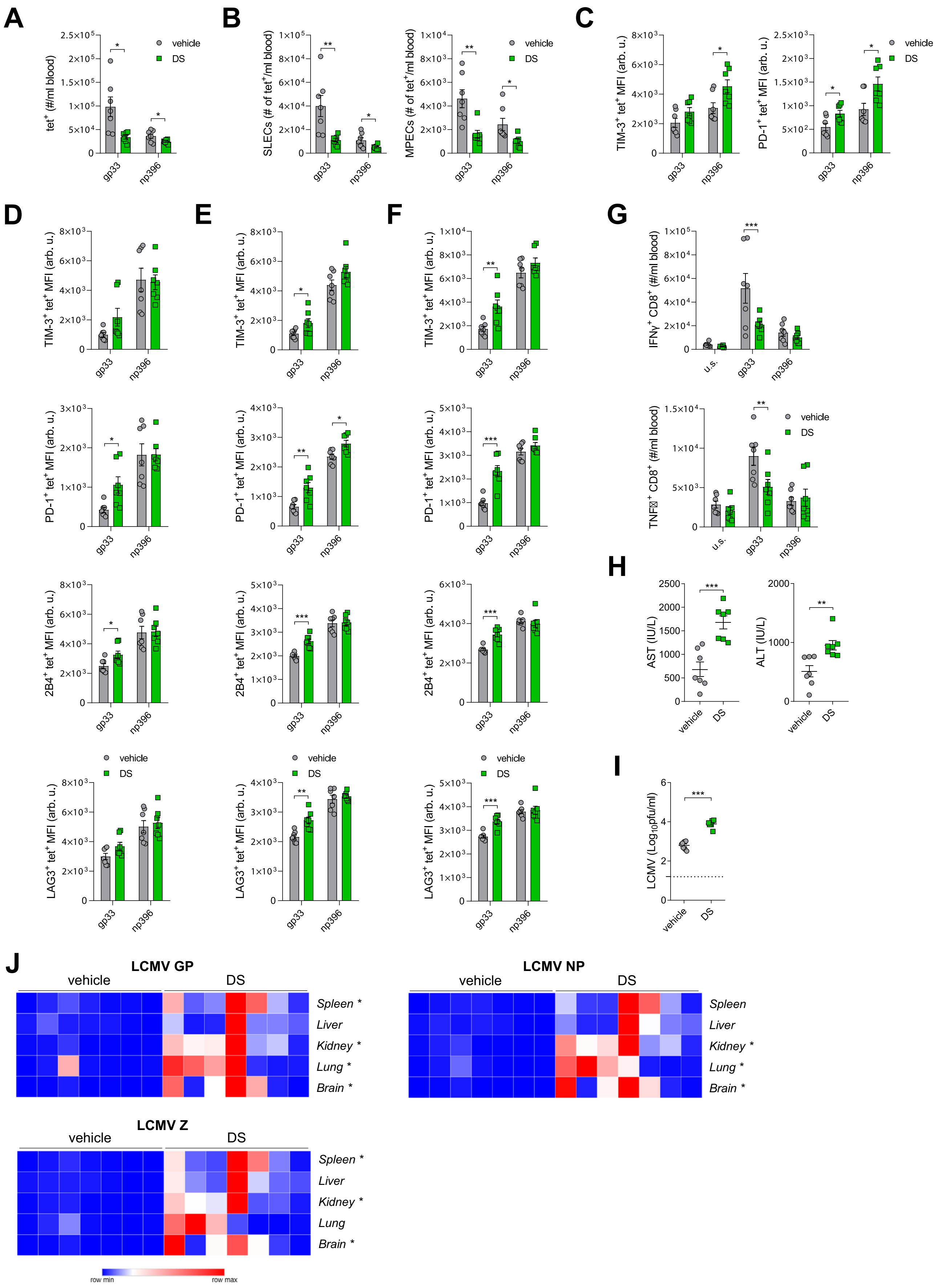
A
B
C
D
E
F
G
H
I
J
vehicle
DS
gp33
np396
u.s.
tet+ (#/ml blood)
SLECs (# of tet+/ml blood)
MPECs (# of tet+/ml blood)
TIM-3+ tet+ MFI (arb. u.)
PD-1+ tet+ MFI (arb. u.)
2B4+ tet+ MFI (arb. u.)
LAG3+ tet+ MFI (arb. u.)
IFNγ+ CD8+ (#/ml blood)
CD8+ (#/ml blood)
AST (IU/L)
ALT (IU/L)
LCMV (Log10pfu/ml)
LCMV GP
LCMV NP
LCMV Z
Spleen *
Liver
Kidney *
Lung *
Brain *
Spleen
Lung
row min
row max

Figure EV3. Treatment with dextran sulfate at the beginning of infection leads to virus persistence and pathology.

LCMV WE (5×10^6 pfu) was pretreated with dextran sulfate (DS, 500 μg/ml) for 30 min, and then injected i.v. into C57BL/6J mice (10^6 pfu per mouse), while control mice were infected with LCMV WE. Mice were re-injected i.v. with dextran sulfate (100 μg per mouse) 6 h p.i., and control mice were injected with PBS. Analyses were carried out on day 8 and 12 p.i. (**A**) Tet-gp33$^+$ and tet-np396$^+$ T cells were determined in blood on day 8 p.i. (**B**) SLECs (IL7R$^-$, KLRG1$^+$) and MPECs (IL7R$^+$, KLRG1$^-$) subsets within tet$^+$ T cells were determined in blood on day 8 p.i. (**C**) Surface expression of exhaustion markers on tet$^+$ T cells was determined in blood on day 8 p.i. (**D–F**) Surface expression of exhaustion markers on tet$^+$ T cells was determined in blood (**D**), spleen (**E**), and liver (**F**) on day 12 p.i. (**G**) IFN-γ and TNF-α production by CD8$^+$ T cells in blood after re-stimulation with LCMV-specific peptides was determined on day 8 p.i. (**H**) ALT and AST activity in the serum of control and DS-treated mice was evaluated on day 8 p.i. (**I**) LCMV titer was determined in blood on day 8 p.i. Tet$^+$ CD8$^+$ T cells, SLECs, MPECs (both populations as subsets of tet$^+$ cells), and cytokine-producing CD8$^+$ T cells are presented as absolute counts: a number of cells per ml of blood, per spleen, or per liver lobe, as indicated. Data presented as mean ± SEM, $n = 7$ mice per condition, *$P < 0.05$, **$P < 0.01$, ***$P < 0.001$. Statistical significance was assessed by Student's *t* test, or two-way ANOVA in case of IFN-γ and TNF-α production. (**J**) mRNA expression of LCMV genes was determined in spleen, liver, kidney, lung and brain tissue. Data presented as heatmaps representing the number of cognate mRNA copies per copy of mRNA for reference housekeeping gene, $n = 7$ mice per condition. *$P < 0.05$ between control and DS-treated groups. Statistical significance was assessed by Student's *t* test. Source data are available online for this figure.

 © The Author(s)

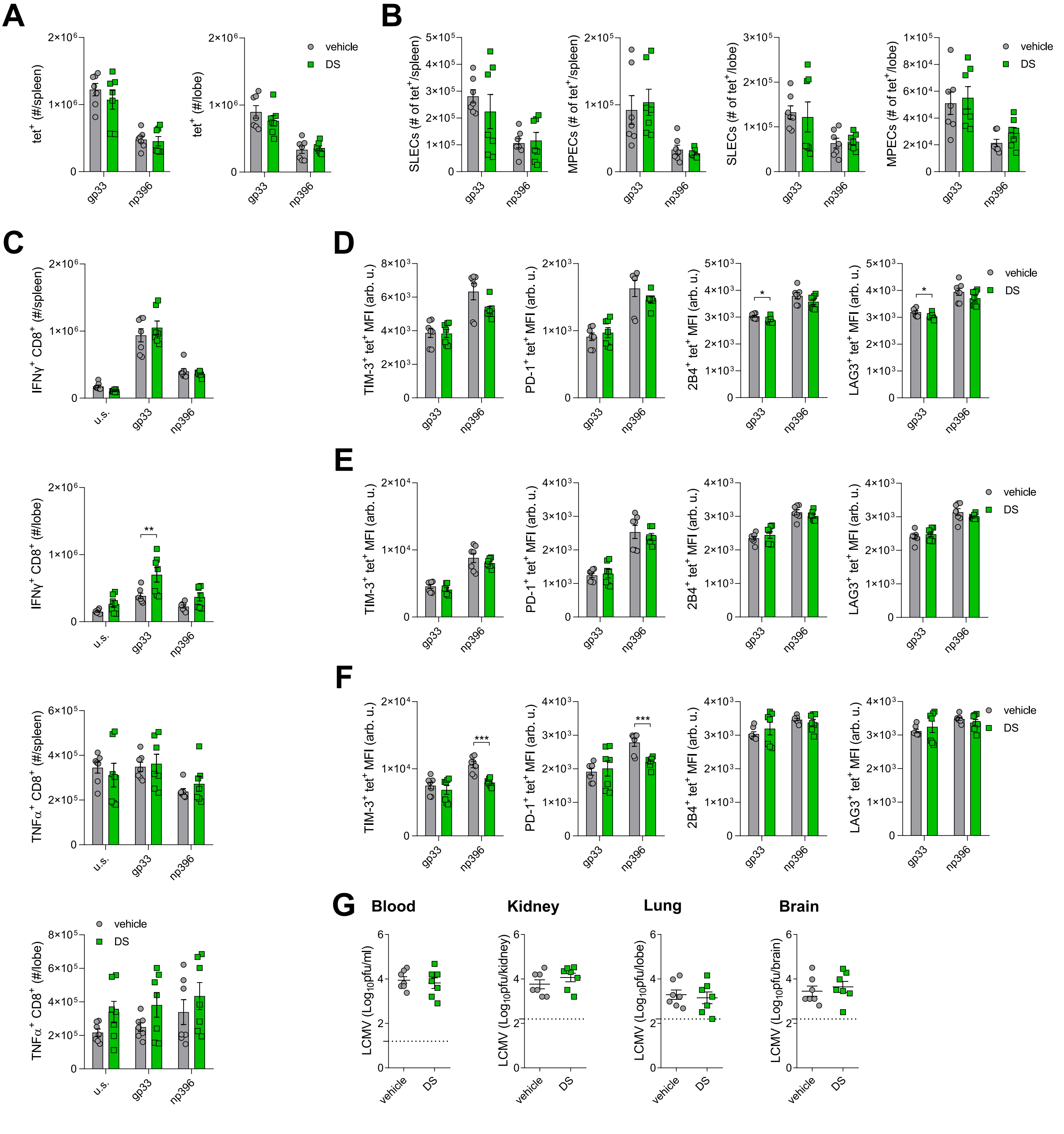

Figure EV4. Treatment with dextran sulfate in the course of active infection leads to increased antiviral immunity.

C57BL/6J mice were infected i.v. with LCMV WE (10^6 pfu per mouse). On day 6 and 7 p.i., mice were injected i.v. with dextran sulfate (DS, 100 µg per mouse), wjile control mice were injected with PBS. Analyses were carried out on day 8 p.i. (**A**) Tet-gp33$^+$ and tet-np396$^+$ T cells were determined in spleen and liver. (**B**) SLECs (IL7R$^-$, KLRG1$^+$) and MPECs (IL7R$^+$, KLRG1$^-$) subsets within tet$^+$ T cells were determined in spleen and liver. (**C**) IFN-γ and TNF-α production by CD8$^+$ T cells in spleen and liver after re-stimulation with LCMV-specific peptides was determined. (**D–F**) Surface expression of exhaustion markers on tet$^+$ T cells was determined in blood (**D**), spleen (**E**), and liver (**F**). (**G**) LCMV titers were determined in blood, kidney, lung and brain. Tet$^+$ CD8$^+$ T cells, SLECs, MPECs (both populations as subsets of tet$^+$ cells), and cytokine-producing CD8$^+$ T cells are presented as absolute counts: number of cells per ml of blood, per spleen, or per liver lobe, as indicated. Data presented as mean ± SEM, $n = 7$ mice per condition, *$P < 0.05$, **$P < 0.01$, ***$P < 0.001$. Statistical significance was assessed by Student's t test, or two-way ANOVA in case of IFN-γ and TNF-α production. Source data are available online for this figure.

© The Author(s)

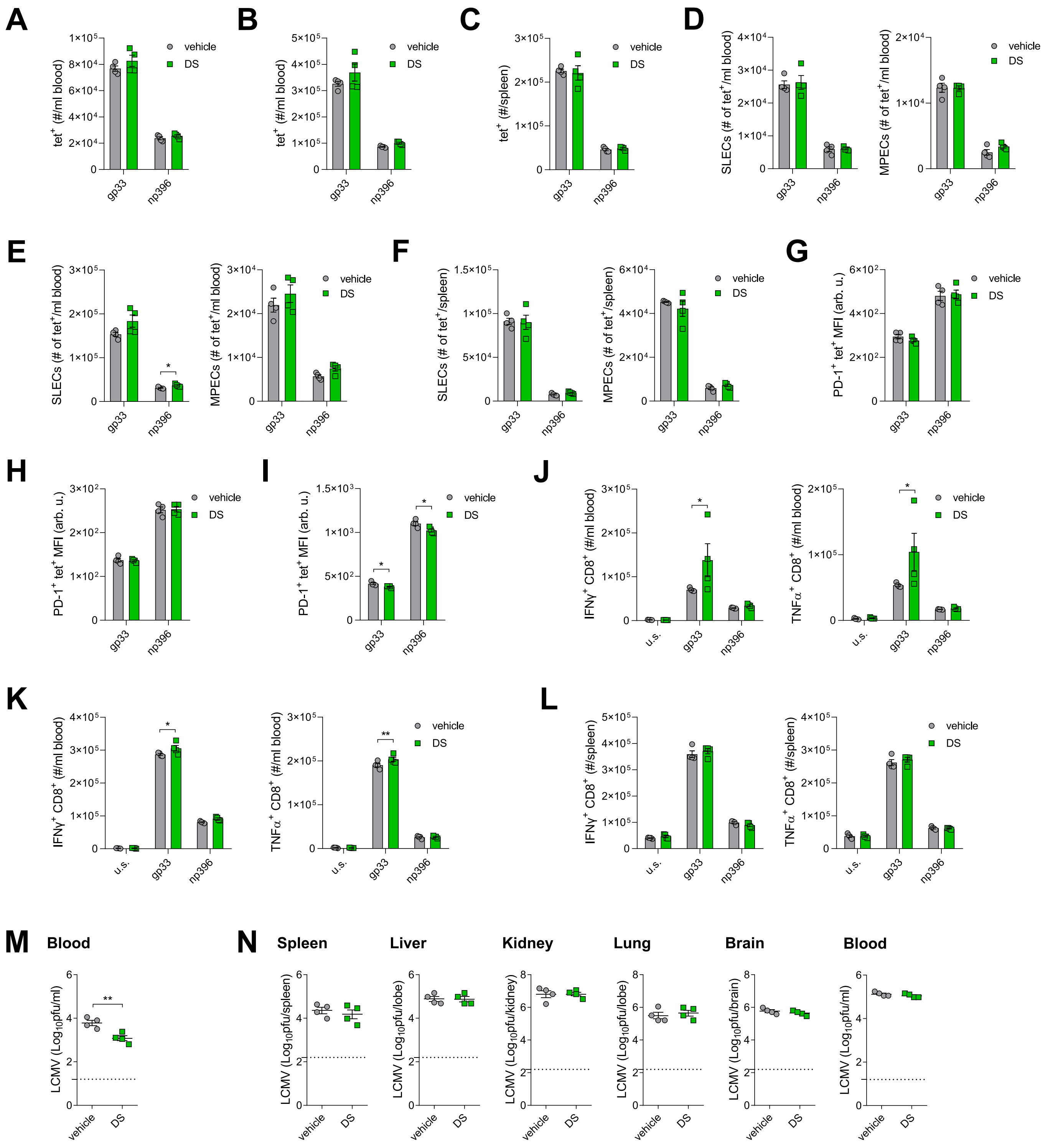
A
B
C
D
E
F
G
H
I
J
K
L
M
N
vehicle
DS
gp33
np396
u.s.
tet+ (#/ml blood)
tet+ (#/spleen)
SLECs (# of tet+/ml blood)
MPECs (# of tet+/ml blood)
SLECs (# of tet+/spleen)
MPECs (# of tet+/spleen)
PD-1+ tet+ MFI (arb. u.)
IFNγ+ CD8+ (#/ml blood)
TNFα+ CD8+ (#/ml blood)
IFNγ+ CD8+ (#/spleen)
TNFα+ CD8+ (#/spleen)
Blood
Spleen
Liver
Kidney
Lung
Brain
LCMV (Log10pfu/ml)
LCMV (Log10pfu/spleen)
LCMV (Log10pfu/lobe)
LCMV (Log10pfu/kidney)
LCMV (Log10pfu/brain)

© The Author(s)

Figure EV5. Treatment with dextran sulfate in the course of LCMV clone 13 infection leads to transiently increased antiviral immunity.

C57BL/6J mice were infected i.v. with LCMV clone 13 (10^6 pfu per mouse). On day 6 and 7 p.i., mice were injected i.v. with dextran sulfate (DS, 100 µg per mouse), while control mice were injected with PBS. Analyses were carried out on day 8, 12 and 20 p.i. (**A–C**) Tet-gp33$^+$ and tet-np396$^+$ T cells were determined in blood on day 8 (**A**), day 12 (**B**), and in spleen on day 20 p.i. (**C–F**) SLECs (IL7R$^-$, KLRG1$^+$) and MPECs (IL7R$^+$, KLRG1$^-$) subsets within tet$^+$ T cells were determined in blood on day 8 (**D**), day 12 (**E**), and in spleen on day 20 p.i. (**F–I**) Surface expression of PD-1 on tet$^+$ T cells was determined in blood on day 8 (**G**), day 12 (**H**), and in spleen on day 20 p.i. (**I–L**) IFN-γ and TNF-α production by CD8$^+$ T cells in blood on day 8 (**J**), day 12 (**K**), and in spleen on day 20 p.i. (**L**) After re-stimulation with LCMV-specific peptides was determined. (**M**) LCMV titers were determined in blood on day 8 p.i. (**N**) LCMV titers were determined in blood, spleen, liver, kidney, lung and brain on day 20 p.i. Tet$^+$ CD8$^+$ T cells, SLECs, MPECs (both populations as subsets of tet$^+$ cells), and cytokine-producing CD8$^+$ T cells are presented as absolute counts: number of cells per ml of blood, or per spleen, as indicated. Data presented as mean ± SEM, $n = 4$ mice per condition, *$P < 0.05$, **$P < 0.01$. Statistical significance was assessed by Student's *t* test, or two-way ANOVA in case of IFN-γ and TNF-α production. Source data are available online for this figure.

© The Author(s)
